# Copper‐Catalyzed Regio‐ and Enantioselective Hydroboration of Difluoroalkyl‐Substituted Internal Alkenes

**DOI:** 10.1002/advs.202304194

**Published:** 2023-10-25

**Authors:** Tao‐Qian Zhao, Hui Xu, Yu‐Chen Tian, Xiaodong Tang, Yanfeng Dang, Shaozhong Ge, Jun‐An Ma, Fa‐Guang Zhang

**Affiliations:** ^1^ Joint School of National University of Singapore and Tianjin University International Campus of Tianjin University Binhai New City Fuzhou 350207 P. R. China; ^2^ Department of Chemistry Tianjin Key Laboratory of Molecular Optoelectronic Sciences Frontiers Science Center for Synthetic Biology (Ministry of Education) Tianjin University Tianjin 300072 P. R. China; ^3^ Department of Chemistry National University of Singapore 3 Science Drive 3 Singapore 117543 Singapore

**Keywords:** copper catalysis, difluoromethylene group, enantioselectivity, hydroboration, regioselectivity

## Abstract

Catalytic asymmetric hydroboration of fluoroalkyl‐substituted alkenes is a straightforward approach to access chiral small molecules possessing both fluorine and boron atoms. However, enantioselective hydroboration of fluoroalkyl‐substituted alkenes without fluorine elimination has been a long‐standing challenge in this field. Herein, a copper‐catalyzed hydroboration of difluoroalkyl‐substituted internal alkenes with high levels of regio‐ and enantioselectivities is reported. The native carbonyl directing group, copper hydride system, and bisphosphine ligand play crucial roles in suppressing the undesired fluoride elimination. This atom‐economic protocol provides a practical synthetic platform to obtain a wide scope of enantioenriched secondary boronates bearing the difluoromethylene moieties under mild conditions. Synthetic applications including functionalization of biorelevant molecules, versatile functional group interconversions, and preparation of difluoroalkylated Terfenadine derivative are also demonstrated.

## Graphical abstract

A copper‐catalyzed regio‐ and enantioselective hydroboration of difluoroalkyl‐substituted internal alkenes without fluorine elimination is realized, thus enabling practical and atom‐economic access to a broad scope of chiral secondary boronates containing the valuable difluoromethylene motifs.

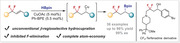

## 1 Introduction

**Figure 1:**
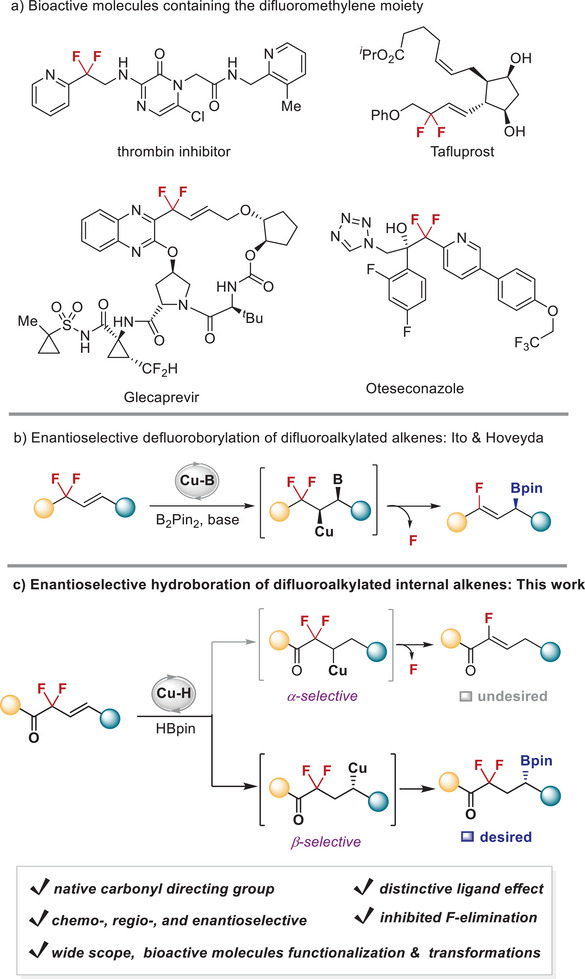
Catalytic asymmetric hydroboration of difluoroalkylated alkenes.

Organofluorine compounds have found numerous applications in a broad range of disciplines, thus continuing to drive the development of new strategies for their efficient and precise synthesis.^[^ ^1^ ^]^ In particular, difluoromethylene‐featured units are greatly desired structures owing to their prevalence as key scaffolds in a large number of bioactive molecules such as glaucoma treat drug Talfuprost and hepatitis C treatment drug Glecaprevir (**Figure** **1**a).^[^ ^2^ ^]^ However, compared with the remarkable progresses in the preparation of fluorinated and trifluoromethylated molecules,^[^ ^3^ ^]^ available synthetic approaches to access difluoromethylene‐containing compounds are still relatively limited, especially in a stereoselective manner.^[^ ^4^ ^]^ Therefore, the development of new catalytic asymmetric transformations to produce diverse difluoromethylene‐containing chiral molecules is still highly sought‐after.

Catalytic asymmetric hydroboration of unsaturated carbon–carbon double bonds represents one of the most renowned reactions for the construction of synthetically useful chiral alkyl boronates.^[^ ^5^ ^]^ In particular, when fluorine‐containing alkenes are employed as the substrates, an attractive type of fluorinated organoboron compounds could be obtained.^[^ ^6^ ^]^ The introduction of boryl group not only provides a powerful synthetic handle for versatile downstream transformations but also highlights the significance of fluorine and boron themselves as important moieties in drug design and development.^[^ ^1^, ^7^ ^]^ Over the past decades, tremendous efforts have been focused on the borylation of trifluoromethyl alkenes.^[^ ^8^, ^9^ ^]^ In sharp contrast, little progress has been made in the manipulation of difluoroalkyl‐substituted alkenes with boron reagents, and only one report by Ito and Hoveyda has addressed the copper‐catalyzed enantioselective defluoroborylation of difluoroalkyl‐substituted alkenes to give monofluoro‐allylic boronates. The proposed mechanism for this selective defluorination stems from the α‐regioselectivity of Cu─B complex across the C═C bond to generate difluoroalkylcopper intermediate that is prone to undergo β‐fluoride elimination (Figure 1b).^[^ ^10^ ^]^ However, to the best of our knowledge, catalytic asymmetric hydroboration of difluoroalkylated internal alkenes while keeping the difluoromethylene motif intact is still not realized. The greatest challenge mainly lies in the regioselectivity issue, that is the α‐selective hydrocupration is electronically favored for Cu–H catalysis,^[^ ^11^ ^]^ but the formed α‐cuprated difluoroalkyl intermediate would easily undergo β‐fluoride elimination (Figure 1c, undesired pathway). Therefore, here we report our results on the copper‐catalyzed β‐regioselective hydroboration of difluoroalkyl‐substituted internal alkenes without defluorination to give the chiral difluoroalkyl boronates with high enantioselectivities (Figure 1c, desired pathway). The key findings to suppress the competing fluoride elimination consists of the use of native carbonyl directing group,^[^ ^12^ ^]^ harnessing Cu–H catalysis system, and the judicious choice of Ph‐BPE as the crucial chiral ligand.

## 2 Results and Discussion

### 2.1 Reaction Optimizations

**Figure 2:**
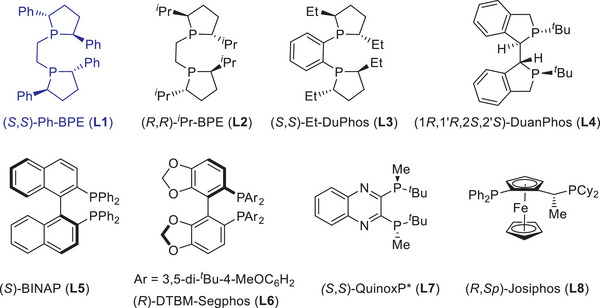
Chiral ligands used in this study.


We began our investigation by using the easily accessible (*E*)−2,2‐difluoro‐4‐phenylbut‐3‐enoate **1a** as the model substrate to undergo hydroboration with HBpin under copper catalysis conditions (**Table** **1**). The screening experiments revealed that the desired hydroboration product **2a** was obtained in 78% isolated yield with 99% ee by utilizing CuOAc in combination with Ph‐BPE as the chiral ligand, while the defluorinative side‐product **2a'** and the reduction side‐products **2a''** were not observed (**2a**/**2a'**/**2a''** > 99:0:0, entry 1). As discussed above, the formation of boronate **2a** was attributable to the β‐regioselective hydrocupration, while monofluoro alkene **2a'** was ascribed to the defluorination process relevant to α‐regioselectivity, thus demonstrating the critical role of β‐regioselectivity on suppressing the undesired fluorine elimination. Notably, the bisphosphine ligand plays a critical role in controlling both the reactivity and enantioselectivity (entries 2–8 and **Figure** **2**). For example, the change of Ph‐BPE into *^i^* Pr‐BPE, Duphos, Duanphos, or BINAP, almost completely failed to catalyze the reaction (entries 2–5). Interestingly, bulky DTBM‐Segphos could promote the reaction of **1a** with HBPin, but defluorinative product **2a'** was observed to be the major product (entry 6). QuinoxP* and Josiphos proved to be effective in enabling the selective formation of benzylic boronate **2a**, albeit in moderate yields with decreased ee values (entries 7 and 8). The replacement of CuOAc with alternative copper salts such as Cu(OAc)_2_ and CuTc resulted in the formation of **2a** with 95%–99% ee, but with a significant drop in yields (entries 9 and 10). In comparison, other earth‐abundant metal catalysts such as cobalt and nickel, proved to be invalid for this transformation (entries 11 and 12). A screening of reaction media confirmed the optimal choice of cyclohexane as the solvent (entries 13–15). In addition, the yield of **2a** was further improved to 85% by operating the reaction at elevated temperatures (50 °C), albeit at the cost of slightly decreased enantioselectivity (95% ee, entry 16). It should be mentioned that subjecting **1a** to the optimal Cu‐B system reported in Ito & Hoveyda's study led to the formation of difluoro‐remaining product **B1a** in 87% yield with 55% ee (equation 1).^[^ ^10^ ^]^

**Table 1:** Reaction development.

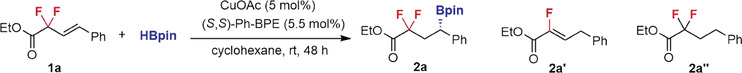				
Entry^a^ ^)^	Derivations from standard conditions	Ratio of **2a**/**2a′**/**2a′′**	Yield of **2a** (%)	ee of **2a** (%)
1	none	>99:0:0	84(78)	99
2	**L2** instead of **L1**	nd	nr	nd
3	**L3** instead of **L1**	nd	nr	nd
4	**L4** instead of **L1**	47:47:6	trace	nd
5	**L5** instead of **L1**	nd	nr	nd
6^b^ ^)^	**L6** instead of **L1**	10:87:38	trace	nd
7	**L7** instead of **L1**	90:8:2	63	90
8	**L8** instead of **L1**	>99:0:0	68	−89
9	Cu(OAc)_2_ instead of CuOAc	72:4:24	18	95
10	CuTc instead of CuOAc	94:0:6	28	99
11	Co(OAc)_2_ instead of CuOAc	0:30:70	nd	nd
12	Ni(cod)_2_ instead of CuOAc	0:48:52	nd	nd
13	THF instead of cyclohexane	92:6:2	86	59
14	dioxane instead of cyclohexane	82:0:18	70	93
15	toluene instead of cyclohexane	88:2:10	70	93
16	50 °C instead of rt	>99:0:0	85	95

^a)^ Reaction conditions unless otherwise noted: **1a** (0.1 mmol), HBpin (1.5 equiv), CuOAc (5 mol%), ligand (5.5 mol%), solvent (1.0 mL), rt, 48 h. Ratios of **2a**:**2a′**:**2a′′** were determined by ^19^F‐NMR of crude reaction mixtures with PhOCF_3_ as the internal standard. Yields of **2a** were determined by ^19^F‐NMR of crude reaction mixtures with PhOCF_3_ as the internal standard, and yield of **2a** in parentheses was determined by the isolated product after column chromatograph. Enantiomeric excess (ee) values of **2a** were determined by chiral HPLC analysis.

^b^ ^)^ The corresponding carboxylic acid of **2a′** was obtained as the major product. “nd” means “not determined”. “nr” means “no reaction”.

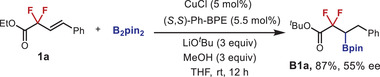


### 2.2 Substrate Scope

**Figure 3:**
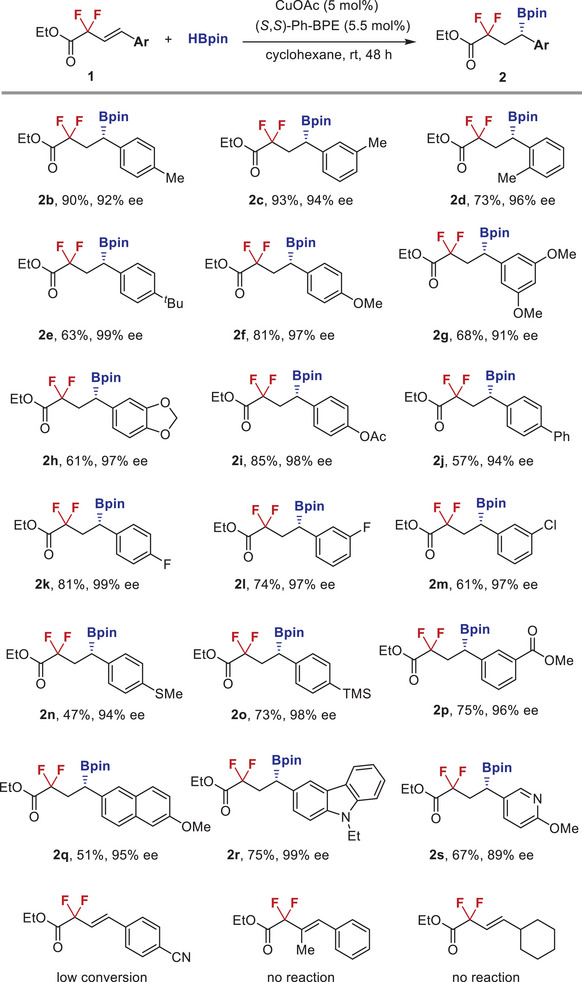
Substrate scope of alkenyl difluoroacetates.

This catalytic enantioselective hydroboration platform proved to be widely compatible to a series of difluoroalkyl‐substituted internal alkenes without the loss of fluorine atom (**Figure** **3**). For example, the aryl moieties substituted by various alkyl groups, alkoxyl groups, acetoxyl group, and phenyl group, at different positions of the benzene ring (*para*, *meta*, *ortho*), were all well tolerated under standard conditions to give the corresponding difluoroalkyl boronates **2b**–**2j** in moderate to high yields with excellent enantioselectivities. Heteroatoms including fluorine, chlorine, sulfur, and silicon, were all compatible in this reaction and the corresponding difluoroalkyl boronates **2k**–**2o** were obtained in good results (94%–99% ee). Incorporation of strong electron‐withdrawing carboxylic ester group at the *meta* position of the benzene ring proved to be viable, affording **2p** in good yield with 96% ee. 2‐Naphthyl‐derived alkenyl difluoroacetate reacted with HBpin to provide **2q** with 95% ee. Furthermore, heteroaromatic groups such as carbazole and pyridine were also accommodated under identical conditions to give hydroboration products **2r** and **2s** in good yields with 99% and 89% ee, respectively. In addition, the scope of this reaction also exhibited some limitations, for example, substrate bearing strong electron‐withdrawing group at the *para* position of phenyl ring, trisubstituted alkene, and alkyl group‐substituted difluoroalkyl alkene were inert under current conditions.

**Figure 4:**
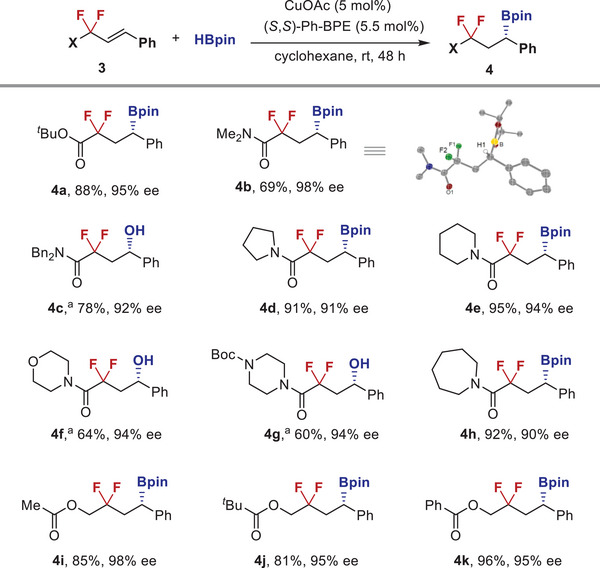
Substrate scope with respect to the difluoroalkyl substituent. *^a^* The corresponding alcohols were obtained via oxidation of the crude boronates by NaBO_3_.

With respect to the difluoroalkyl substituent (**Figure** **4**), switching ethyl group to more sterically hindered *tert*‐butyl group was found to be well acceptable (**4a**). Furthermore, this reaction protocol is not limited to alkenyl difluoroacetates, and a broad range of tertiary difluoroacetamides are also good reaction partners. For example, dimethyl and dibenzyl‐containing amides underwent β‐regioselective hydroboration smoothly (**4b** and **4c**). The absolute configuration of boronate **4b** was determined to be *S* by X‐ray crystallographic analysis, and other products were assigned by analogy. A series of cyclic amides, including pyrrole, piperidine, morpholine, piperazine, and azepane‐containing substrates, reacted with HBpin under the standard conditions to give difluoroalkyl boronates **4d**–**4h** in good yields with high enantioselectivities (90%–94% ee). More importantly, alkenyl difluoroalkyl acetoxyl substrates also underwent the desired hydroboration, giving the corresponding boronates **4i**–**4k** in high yields and high enantioselectivities.

**Figure 5:**
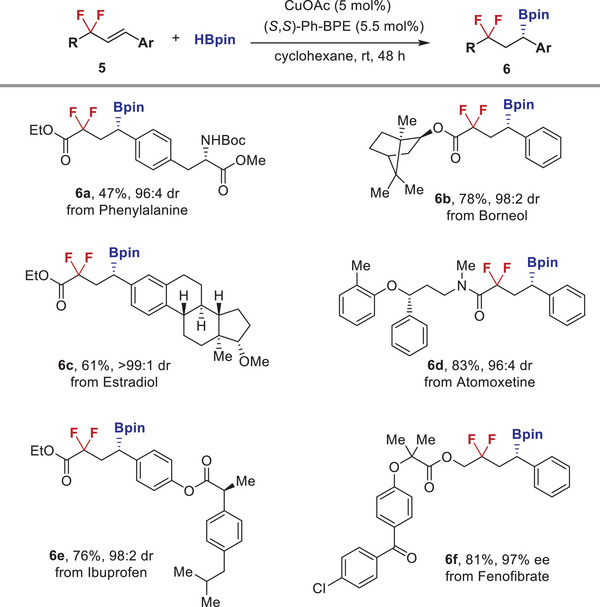
Applications for the functionalization of biorelevant molecules.

The functional group compatibility and potential utility of this method was further demonstrated by the functionalization of biorelevant molecules (**Figure** **5**). Phenylalanine‐derived alkenyl difluoroacetate derivative underwent chemo‐, regio‐ and stereoselective hydroboration without fluorine elimination to provide **6a** in a practical yield with high dr value. Natural product structures including Borneol and Estradiol were amenable to be equipped at the carbonyl and aryl sites, affording boronates **6b** and **6c** with excellent diastereoselectivities. Furthermore, three pharmaceutical molecules including attention deficit hyperactivity disorder drug Atomoxetine, nonsteroidal anti‐inflammatory drug Ibuprofen, and antihyperlipidemic drug Fenofibrate were also successfully decorated with the difluoroalkyl boronate motif (**6d**–**6f**).

### 2.3 Synthetic Transformations

**Figure 6:**
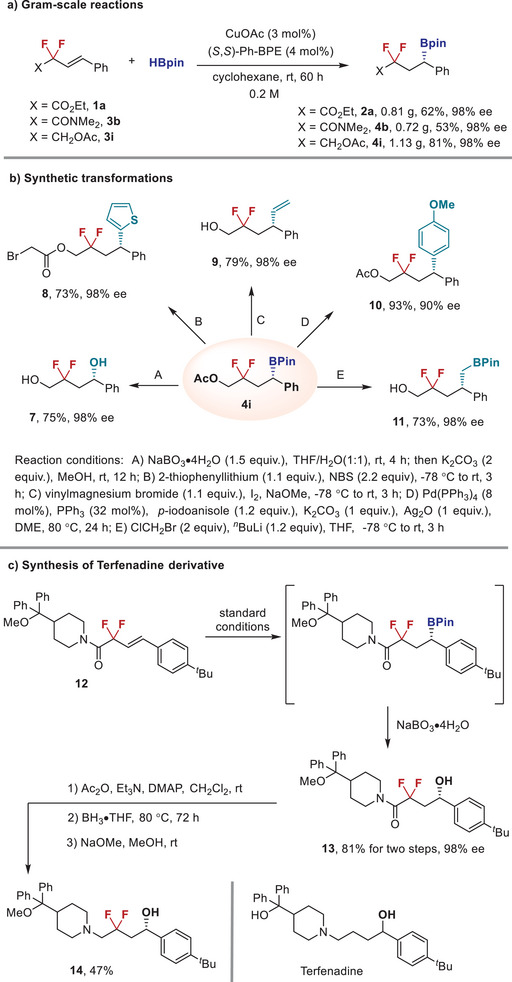
Further demonstration of synthetic utility.

Subsequently, three gram‐scale reactions were conducted to probe the practicality of this transformation to furnish the desired products **2a**, **4b**, and **4i** in good yields with maintained high level of enantioselectivies (**Figure** **6**a). More importantly, the obtained chiral difluoroalkyl boronates could undergo a series of boron‐based stereospecific functional group interconversions (Figure 6b). Among them, oxidation of **4i** by NaBO_3_ led to *gem*‐difluorinated 1,4‐diol **7** in 75% yield with full stereo‐retention.^[^ ^13^ ^]^ Treating **4i** with 2‐thienyllithium in the presence of *N*‐bromosuccinimide provided a thienylated difluoroalkyl ester **8** in 73% yield without loss of ee values.^[^ ^14^ ^]^ Iodine‐promoted vinylation of boronate ester proceeded with conservation of enantiomeric excess to give the corresponding difluorinated alcohol **9** in 79% yield.^[^ ^15^ ^]^ Palladium‐catalyzed Suzuki−Miyaura cross‐coupling reaction of boronate **4i** with iodioanisole was also viable to generate compound **10** in high yield with predominant stereo‐retention at the chiral center.^[^ ^16^ ^]^ Furthermore, Matteson homologation of secondary boronic ester **4i** occurred without erosion of optical purity, thus yielding compound **11** in 73% yield with 98% ee.^[^ ^17^ ^]^ More importantly, this protocol was further applied in the asymmetric synthesis of difluoroalkyl analogue of antihistamine drug Terfenadine (Figure 6c).^[^ ^18^ ^]^ Copper‐catalyzed enantioselective hydroboration of **12** yielded the boronate as an intermediate, which was subsequently oxidized to chiral alcohol **13** in 81% total yield with 98% ee. Further reduction of **13** with borane provided the corresponding amine **14** in 47% total yield through a three‐step procedure. In addition, it should be mentioned that an alternative path to access the compounds in Figure 6b by performing a cascade sequence of enantioselective conjugate addition to enones and deoxyfluorination transformations might also be possible.^[^ ^19^ ^]^

### 2.4 Mechanistic Studies

**Figure 7:**
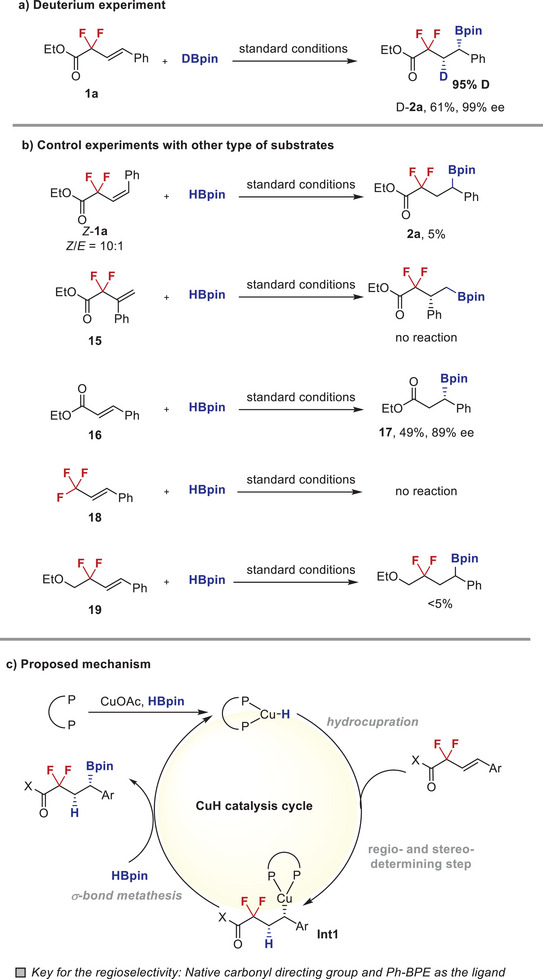
Experimental mechanistic studies.

A series of experiments were performed to gain insight on the mechanism of this reaction (**Figure** **7**). First, isotope labelling experiment with D‐Bpin under otherwise standard conditions generated the corresponding D‐**2a** with 95% deuterium incorporation in 61% yield and 99% ee (Figure 7a). The excellent diastereoselectivity of D‐**2a** suggested an irreversible addition of Cu–H species across the C═C bond and the hydrocupration process is thus estimated to be the regio‐ and stereo‐determining step of this reaction. Subsequently, we conducted a series of control experiments with other types of substrates to understand the influence of alkene structures. As illustrated in Figure 7b, both the *Z*‐configured isomer of model substrate **1a** and the 1,1‐disubstituted alkenyl difluoroacetate **15**, was nearly inert under standard conditions (≤ 5% conversions). Interestingly, substrates without the difluoromethylene moiety (**16**), underwent this copper‐catalyzed hydroboration with constant exclusive β‐regioselectivity, albeit in decreased yield and enantioselectivity to some extent (product **17**). In sharp contrast, both of 1,2‐disubstituted trifluoromethyl styrene **18** and difluoroalkyl ether **19** were found to be almost unreactive under otherwise identical conditions. Collectively, these control experiments clearly demonstrated the crucial role of carbonyl scaffold as the native directing group in improving the reactivity and dictating regioselectivity. Based on these results and previous studies,^[^ ^20^ ^]^ a plausible mechanism is illustrated in Figure 7c. The reaction starts with the formation of ligated copper hydride species (*LCu–H) from copper salt, bisphosphine ligand, and HBpin. β‐Regioselective hydrocupration undergoes stereoselectively to give the chiral alkylcopper complex **Int1**. Final borylation occurs with retention of configuration via σ‐bond metathesis to produce the desired product and regenerates the *Cu–H catalyst. Notably, the use of copper hydride catalysis system, the choice of Ph‐BPE as the chiral ligand, and the presence of native carbonyl directing group all plays indispensable roles in enabling the realization of this atom‐economic reaction.

**Figure 8:**
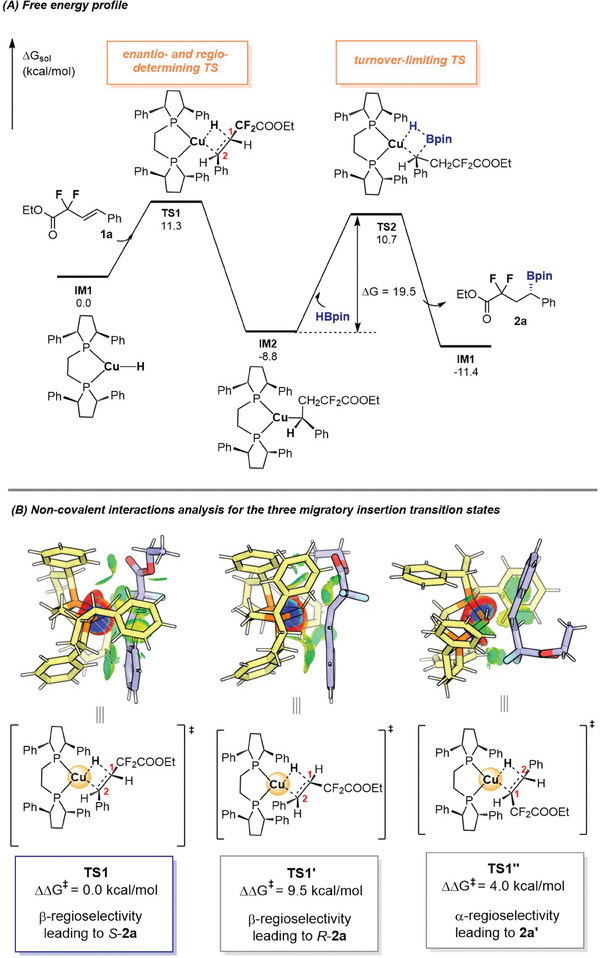
Computational mechanistic studies.

To dig deeper into the mechanism and origin of the regio‐ and stereo‐selectivities, density functional theory calculations have been carried out on the reaction between **1a** and HBpin catalyzed by **L1**‐Cu complex (see Supporting Information for more details). As shown in **Figure** **8**A, copper hydride complex **IM1**, as the zero point, inserts into alkene **1a** to generate **IM2** via **TS1** exogenously by 8.8 kcal mol^−1^ with an activation free energy of 11.3 kcal mol^−1^. And then, σ‐bond metathesis between H−B bond of HBpin and Cu−C bond of **IM2** occurs to release the final product **2a** and regenerate the active Cu‐hydride catalyst **IM1**, which requires an energy barrier of 19.5 kcal mol^−1^ via **TS2**. The calculation results show that the σ‐bond metathesis (**TS2**) is identified as the turnover‐limiting step, and moreover, the irreversible alkene insertion step (**TS1**) exercises control over the regio‐ and stereo‐selectivities, which is consistent with our deuterium studies.

With comprehension of the reaction mechanism, we next sought to scrutinize the observed selectivities. As shown in Figure 8B, the activation barrier for 2,1‐migratory insertion of **1a** via **TS1** (β‐regioselectivity) is 4.0 kcal mol^−1^ more favorable than that for 1,2‐migratory insertion into via **TS1′′** (α‐regioselectivity), which is in accord with the experimentally observed regioselectivity. In addition, the enantioselectivity is controlled by the relative energy (9.5 kcal mol^−1^) of the two diastereomeric transition states **TS1** (leading to *S*‐**2a**) and **TS1′** (leading to *R*‐**2a**) for the insertion of **1a**. Noncovalent interactions (NCIs) using the IGMH analysis has been adopted to comparing the three competing transition states. Obviously, **TS1** has more pronounced C−H···H−C (contributed by the carbonyl group) and C−H···π dispersion interactions between **1a** and Cu catalyst compared to **TS1′** and **TS1′′**, which accounts for the observed selectivities of the Cu‐catalyzed hydroboration reaction.

## 3 Conclusion

In conclusion, we have successfully developed a regio‐ and enantioselective hydroboration of difluoroalkyl‐substituted internal alkenes without the loss of fluorine atom. The key to the success of this reaction relies on the concerted engagement of native carbonyl directing group, copper hydride catalysis system, and Ph‐BPE as the chiral ligand. This atom‐economic protocol provides practical access to a wide range of chiral difluoroalkyl boronates that are viable to versatile further synthetic transformations with high stereochemical fidelity. Combined experimental and computational studies indicate that hydrocupration process is the regio‐ and stereo‐selectivity determining step, and the carbonyl‐containing difluoroalkyl substrates manifest important noncovalent interactions to stabilize the corresponding transition states. We anticipate this unprecedented strategy will find broad applications in both synthetic chemistry and drug development. Further investigation into the mechanism and application to other substrates are currently underway in our laboratories.

[CCDC 2 262 049 (**4b**) contains the supplementary crystallographic data for this paper. These data can be obtained free of charge from The Cambridge Crystallographic Data Centre via www.ccdc.cam.ac.uk/data_request/cif.]

## Conflict of Interest

The authors declare no conflict of interest.

## Supporting information

Supporting Information

## Data Availability

The data that support the findings of this study are available in the supplementary material of this article.
