# Supplementary material for: Copper‐Catalyzed Regio‐ and Enantioselective Hydroboration of Difluoroalkyl‐Substituted Internal Alkenes
Source: Adv Sci (Weinh). 2023 Oct 25;10(35):2304194. doi: 10.1002/advs.202304194 (PMC10724385; doi:10.1002/advs.202304194)
Supplement: Supplementary file 1 — Supporting Information [file ADVS-10-2304194-s001.pdf]

ADVANCED SCIENCE
Open Access

Supporting Information

for *Adv. Sci.*, DOI 10.1002/advs.202304194

Copper-Catalyzed Regio- and Enantioselective Hydroboration of Difluoroalkyl-Substituted Internal Alkenes

Tao-Qian Zhao, Hui Xu, Yu-Chen Tian, Xiaodong Tang, Yanfeng Dang, Shaozhong Ge, Jun-An Ma* and Fa-Guang Zhang**

· Supporting Information

Copper-Catalyzed Regio- and Enantioselective Hydroboration of Difluoroalky-Substituted Internal Alkenes

Tao-Qian Zhao,[a,b,c] Hui Xu,[b] Yu-Chen Tian,[a,b] Xiaodong Tang,[a,b] Yanfeng Dang,[b] Shaozhong Ge,[c],* Jun-An Ma,[a,b],* and Fa-Guang Zhang[b],*

[a] T.-Q. Zhao, Y.-C. Tian, Dr. X.-D. Tang, and Prof. Dr. J.-A. Ma
Joint School of National University of Singapore and Tianjin University, International Campus of Tianjin University, Binhai New City, Fuzhou 350207, P. R. of China.
Email: majun_an68@tju.edu.cn

[b] T.-Q. Zhao, H. Xu, Y.-C. Tian, Dr. X.-D. Tang, Prof. Dr. Y. Dang, Prof. Dr. J.-A. Ma, and Prof. Dr. F.-G. Zhang
Department of Chemistry, Tianjin Key Laboratory of Molecular Optoelectronic Sciences, Frontiers Science Center for Synthetic Biology (Ministry of Education), Tianjin University, Tianjin 300072, P. R. of China.
Email: zhangfg1987@tju.edu.cn

[c] T.-Q. Zhao, Prof. Dr. S. Ge
Department of Chemistry, National University of Singapore, 3 Science Drive 3, Singapore 117543, Singapore.
Email: chmgsh@nus.edu.sg.

Table of content

1. General Information 2
2. General Procedure for the Preparation of Difluoroalkyl-Substituted Internal Alkenes 3
3. General Procedure for the Synthesis of Fluoroalkyl Boronates **2**, **4** and **6**. 13
4. General Procedure for Gram-Scale Reactions and Synthetic Transformations 56
5. Mechanistic Studies 75
6. Details of Computational Studies 81
7. NMR Spectra of the Related Compounds 108
8. X-Ray Crystallographic Data 222
9. References 223

1. General Information

All the manipulations were performed in an argon-filled glovebox, unless mentioned otherwise. ^{1}H, ^{13}C and ^{19}F were recorded on Bruker AV 400 MHz or 500 MHz instrument at 400 MHz (^{1}H NMR) or 500 MHz (^{1}H NMR), 101 MHz (^{13}C NMR) or 126 MHz (^{13}C NMR), as well as 377 MHz (^{19}F NMR) and 470 MHz (^{19}F NMR). Chemical shifts were reported in ppm down field from internal Me_4Si and external CCl_3F, respectively. Multiplicity was indicated as follows: s (singlet), d (doublet), t (triplet), q (quartet), m (multiplet), dd (doublet of doublet), dt (doublet of triplet), ddd (doublet of doublet of doublet), td (triplet of doublet), qd (quartet of doublet), br (broad). Coupling constants were reported in Hertz (Hz). GC analysis was acquired on Agilent 6850 gas chromatograph equipped with a flame-ionization detector. GC-MS analysis was performed on Shimadzu GC-2010 gas chromatograph coupled to a Shimadzu QP2010 mass selective detector. High resolution mass spectrometry (HRMS) spectra were obtained on a Bruker miorOTOF-QII instrument. Optical rotations were determined using an Autopol IV-T polarimeter and reported as follows: $[\alpha]_D^{25}$ (c = g/100 mL, solvent). HPLC analyses were carried out on Shimadzu Model LC-2030C 3D instrument or Shimadzu DGU-20A and was performed with Chiralpak® OD-H, OJ-H, AS-H or IG column (4.6 mm × 250 mm × 5 μm) using a Shimadzu HPLC system and was monitored using a UV-detector. X-ray structural analysis was conducted on the Bruker APEX-II CCD instrument.

Materials: Tetrahydrofuran (THF) and toluene were distilled from sodium; Cyclohexane and 1,4-dioxane were purchased from Energy chemical in high purities. CuOAc was purchased from Strem Chemicals, Pinacolborane (HBpin) was purchased from Oakwood Chemicals, and all the chiral ligands were purchased from Strem Chemicals, Inc. Commercially available reagents were used as received without further purification, unless otherwise stated. Analytical thin layer chromatography was performed on 0.20 mm silica gel plates. Silica gel (200–300 mesh) was used for flash chromatography. All purchased reagents were used in high purity without further purification.

2. General Procedure for the Preparation of Difluoroalkyl-Substituted Internal Alkenes

The internal alkenes **1a-1s**, **3a-3h**, **5a-5e** were prepared according to the general procedure A.[1] The corresponding styrene and functionalized difluoromethyl bromide were either purchased from companies or prepared according to a previously reported literature procedure. The internal alkenes **3i-3k** and **5f** were prepared according to the general procedure B.

General procedure A:

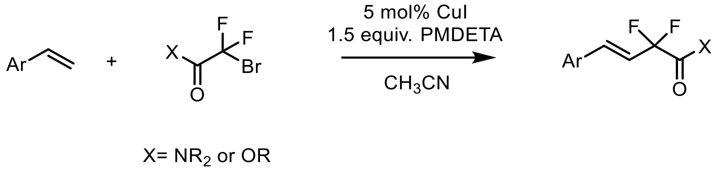

To a solution of styrene (5.00 mmol, 1.0 equiv), CuI (47.7 mg, 0.25 mmol, 5.0 mol%), PMDETA (*N*,*N*,*N′*,*N″*,*N″*-Pentamethyldiethylenetriamine) (1.30 g, 7.50 mmol, 1.5 equiv) and acetonitrile (10 mL) in a Schlenk tube under Ar atmosphere, ethyl bromodifluoroacetate (1.53 g, 7.50 mmol, 1.5 equiv) was added. After stirring at 80 °C in an oil bath for 24 h, the solvent was removed and water (20 mL) was added. The mixture was then extracted with EtOAc (20 mL × 3). The organic layers were combined, washed with brine, dried over anhydrous Na_2SO_4. After removal of solvent, the residue was purified by flash chromatography on silica gel using EtOAc/n-Hexane to give product as a colorless oil or white solid (20-90% yields).

The characterization data of all the new compounds are reported as follows.

Ethyl (*E*)-2,2-difluoro-4-(4-(trimethylsilyl)phenyl)but-3-enoate (1o)

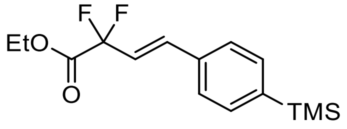

According to general procedure A, with CuI (47.7 mg, 0.25 mmol, 5.0 mol%), product **Styrene-1o** (5.0 mmol, 1.0 equiv), PMDETA (1.30 g, 7.5 mmol, 1.5 equiv) and bromodifluoroacetate (1.5 g, 7.5 mmol, 1.5 equiv) in CH_3CN (20 mL) at 80 °C for 24

h, purified by column chromatography on silica gel with n-Hexane/EtOAc (40:1, v/v) as the eluent. Colorless oil, 1.35 g, 91% yield; **^{1}H NMR** (400 MHz, $CDCl_3$) δ 7.41 (d, *J* = 8.0 Hz, 2H), 7.30 (d, *J* = 8.0 Hz, 2H), 6.96 (dt, *J* = 16.2, 2.6 Hz, 1H), 6.21 (dt, *J* = 16.1, 11.4 Hz, 1H), 4.22 (q, *J* = 7.1 Hz, 2H), 1.23 (t, *J* = 7.1 Hz, 3H), 0.15 (s, 9H). **^{13}C {^{1}H} NMR** (101 MHz, $CDCl_3$) δ 165.1 (t, *J* = 34.9 Hz), 144.0, 138.1 (t, *J* = 9.4 Hz), 135.6, 135.0, 127.8, 120.2 (t, *J* = 25.0 Hz), 114.0 (t, *J* = 248.5 Hz), 64.3, 15.1, 0.0. **^{19}F NMR** (377 MHz, $CDCl_3$) δ -103.22 (dd, *J* = 11.4, 2.6 Hz, 2F); **HRMS (EI)** m/z calcd for $C_{15}H_{20}F_2O_2Si$ $[M+H]^+$: 299.1279; Found: 299.1281.

Methyl (*E*)-3-(4-ethoxy-3,3-difluoro-4-oxobut-1-en-1-yl)benzoate (1p)

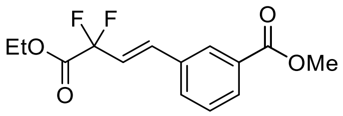

According to general procedure A, with CuI (47.7 mg, 0.25 mmol, 5.0 mol%), product **Styrene-1p** (5.0 mmol, 1.0 equiv), PMDETA (1.30 g, 7.5 mmol, 1.5 equiv) and bromodifluoroacetate (1.5 g, 7.5 mmol, 1.5 equiv) in CH_3CN (20 mL) at 80 °C for 24 h, purified by column chromatography on silica gel with n-Hexane/EtOAc (30:1, v/v) as the eluent. Colorless oil, 1.09 g, 77% yield; **^{1}H NMR** (500 MHz, $CDCl_3$) δ 8.13 (t, *J* = 1.8 Hz, 1H), 8.01 (dt, *J* = 8.0, 1.4 Hz, 1H), 7.63 (dt, *J* = 7.8, 1.5 Hz, 1H), 7.45 (t, *J* = 7.7 Hz, 1H), 7.11 (dt, *J* = 16.2, 2.5 Hz, 1H), 6.40 (dt, *J* = 16.1, 11.3 Hz, 1H), 4.36 (q, *J* = 7.1 Hz, 2H), 3.94 (s, 3H), 1.37 (t, *J* = 7.1 Hz, 3H). **^{13}C {^{1}H} NMR** (126 MHz, $CDCl_3$) δ 166.4, 163.6 (t, *J* = 34.6 Hz), 135.7 (t, *J* = 9.6 Hz), 134.4, 131.7, 130.8, 130.4, 128.9, 128.3, 120.1 (t, *J* = 25.2 Hz), 112.5 (t, *J* = 248.9 Hz), 63.2, 52.2, 13.9. **^{19}F {^{1}H} NMR** (470 MHz, $CDCl_3$) δ -103.43 (s, 2F); **HRMS (EI)** m/z calcd for $C_{14}H_{14}F_2O_4$ $[M+Na]^+$: 307.0758; Found: 307.0762.

Ethyl (*E*)-2,2-difluoro-4-(6-methoxynaphthalen-2-yl)but-3-enoate (1q)

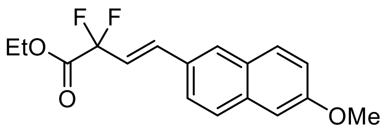

According to general procedure A, with CuI (47.7 mg, 0.25 mmol, 5.0 mol%), product **Styrene-1q** (5.0 mmol, 1.0 equiv), PMDETA (1.30 g, 7.5 mmol, 1.5 equiv) and bromodifluoroacetate (1.5 g, 7.5 mmol, 1.5 equiv) in CH_3CN (20 mL) at

80 °C for 24 h, purified by column chromatography on silica gel with n-Hexane/EtOAc (40:1, v/v) as the eluent. White solid, 1.25 g, 82% yield; **^{1}H NMR** (400 MHz, $CDCl_3$) δ 7.79 (d, *J* = 1.7 Hz, 1H), 7.75 (dd, *J* = 8.7, 4.9 Hz, 2H), 7.59 (dd, *J* = 8.6, 1.8 Hz, 1H), 7.28 – 7.17 (m, 2H), 7.15 (d, *J* = 2.5 Hz, 1H), 6.40 (dt, *J* = 16.2, 11.5 Hz, 1H), 4.40 (q, *J* = 7.2 Hz, 2H), 3.95 (s, 3H), 1.41 (t, *J* = 7.1 Hz, 3H). **^{13}C {^{1}H} NMR** (101 MHz, $CDCl_3$) δ 164.0 (t, *J* = 35.1 Hz), 158.5, 137.0 (t, *J* = 9.5 Hz), 135.2, 129.9, 129.4, 128.6, 128.6, 127.4, 123.9, 119.4, 117.8 (t, *J* = 25.0 Hz), 112.9 (t, *J* = 248.4 Hz), 105.9, 63.1, 55.3, 14.0. **^{19}F NMR** (377 MHz, $CDCl_3$) δ -102.76 (dd, *J* = 11.5, 2.6 Hz, 2F); **HRMS (EI)** m/z calcd for $C_{17}H_{16}F_2O_3$ $[M+Na]^+$: 329.0965; Found: 329.0969.

Ethyl (*E*)-2,2-difluoro-4-(6-methoxypyridin-3-yl)but-3-enoate (1s)

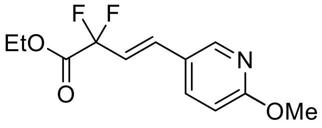

According to general procedure A, with CuI (47.7 mg, 0.25 mmol, 5.0 mol%), product **Styrene-1s** (5.0 mmol, 1.0 equiv), PMDETA (1.30 g, 7.5 mmol, 1.5 equiv) and bromodifluoroacetate (1.5 g, 7.5 mmol, 1.5 equiv) in CH_3CN (20 mL) at 80 °C for 24 h, purified by column chromatography on silica gel with n-Hexane/EtOAc (30:1, v/v) as the eluent. White solid, 0.78 g, 61% yield; **^{1}H NMR** (500 MHz, $CDCl_3$) δ 8.19 (d, *J* = 3.3 Hz, 1H), 7.71 (d, *J* = 8.9 Hz, 1H), 7.02 (dd, *J* = 16.1, 2.9 Hz, 1H), 6.75 (dd, *J* = 8.7, 2.6 Hz, 1H), 6.22 (m, 1H), 4.36 (qd, *J* = 7.1, 2.4 Hz, 2H), 3.95 (d, *J* = 3.0 Hz, 3H), 1.36 (td, *J* = 7.3, 2.6 Hz, 3H). **^{13}C {^{1}H} NMR** (126 MHz, $CDCl_3$) δ 164.8, 163.7 (t, *J* = 34.9 Hz), 147.3, 135.8, 133.1 (t, *J* = 9.6 Hz), 123.3, 117.7 (t, *J* = 25.2 Hz), 112.6 (t, *J* = 248.5 Hz), 111.3, 63.0, 53.5, 13.8. **^{19}F {^{1}H} NMR** (470 MHz, $CDCl_3$) δ -103.01 (s, 2F); **HRMS (EI)** m/z calcd for $C_{12}H_{13}F_2NO_3$ $[M+H]^+$: 258.0942; Found: 258.0939.

(*E*)-2,2-difluoro-*N,N*-dimethyl-4-phenylbut-3-enamide (3b)

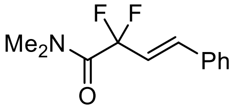

According to general procedure A, with CuI (191.2 mg, 1.0 mmol, 5.0 mol%), Styrene (20.0 mmol, 1.0 equiv), PMDETA (5.19 g, 30

mmol, 1.5 equiv) and *N*,*N*-dimethyl-2-bromo-2,2-difluoroacetamide (6.03 g, 30 mmol, 1.5 equiv) in CH_3CN (50 mL) at 80 °C for 24 h, purified by column chromatography on silica gel with n-Hexane/EtOAc (20:1, v/v) as the eluent. Colorless oil, 1.6 g, 36% yield; **^{1}H NMR** (500 MHz, $CDCl_3$) δ 7.44 (d, *J* = 7.3 Hz, 2H), 7.33 (q, *J* = 8.1, 7.2 Hz, 3H), 6.98 (dt, *J* = 16.3, 2.8 Hz, 1H), 6.45 (dt, *J* = 16.1, 10.9 Hz, 1H), 3.13 (s, 3H), 3.01 (s, 3H). **^{13}C {^{1}H} NMR** (126 MHz, $CDCl_3$) δ 163.3 (t, *J* = 30.0 Hz), 135.4 (t, *J* = 9.7 Hz), 134.3, 129.4, 128.8, 127.3, 120.2 (t, *J* = 24.5 Hz), 115.3 (t, *J* = 248.6 Hz), 37.1 (t, *J* = 5.9 Hz), 36.9. **^{19}F {^{1}H} NMR** (470 MHz, $CDCl_3$) δ -95.62 (s, 2F); **HRMS (EI)** m/z calcd for $C_{12}H_{13}F_2NO$ $[M+Na]^+$: 248.0863; Found: 248.0857.

(*E*)-*N*,*N*-dibenzyl-2,2-difluoro-4-phenylbut-3-enamide (3c)

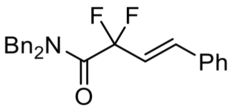

According to general procedure A, with CuI (47.7 mg, 0.25 mmol, 5.0 mol%), Styrene (5.0 mmol, 1.0 equiv), PMDETA (1.30 g, 7.5 mmol, 1.5 equiv) and *N*,*N*-dibenzyl-2-bromo-2,2-difluoroacetamide (2.7 g, 7.5 mmol, 1.5 equiv) in CH_3CN (20 mL) at 80 °C for 24 h, purified by column chromatography on silica gel with n-Hexane/EtOAc (20:1, v/v) as the eluent. Colorless oil, 0.38 g, 20% yield; **^{1}H NMR** (400 MHz, $CDCl_3$) δ 7.46 – 7.32 (m, 11H), 7.30 – 7.23 (m, 4H), 7.04 (dt, *J* = 16.3, 2.8 Hz, 1H), 6.56 (dt, *J* = 16.3, 10.9 Hz, 1H), 4.67 (s, 2H), 4.58 (s, 2H). **^{13}C {^{1}H} NMR** (101 MHz, $CDCl_3$) δ 164.0 (t, *J* = 30.1 Hz), 136.0, 135.8 (t, *J* = 9.7 Hz), 135.6, 134.2, 129.5, 128.8, 128.7, 128.4, 127.9, 127.8, 127.4, 120.2 (t, *J* = 24.6 Hz), 115.5 (t, *J* = 249.5 Hz), 49.8 (t, *J* = 5.2 Hz), 48.3. **^{19}F NMR** (377 MHz, $CDCl_3$) δ -94.08 (dd, *J* = 11.1, 2.7 Hz, 2F); **HRMS (EI)** m/z calcd for $C_{24}H_{21}F_2NO$ $[M+Na]^+$: 400.1489; Found: 400.1485.

Tert-butyl (*E*)-4-(2,2-difluoro-4-phenylbut-3-enoyl)piperazine-1-carboxylate (3g)

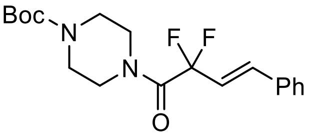

According to general procedure A, with CuI (47.7 mg, 0.25 mmol, 5.0 mol%), Styrene (5.0 mmol, 1.0 equiv), PMDETA (1.30 g, 7.5 mmol, 1.5 equiv) and tert-butyl 4-(2-bromo-2,2-difluoroacetyl)piperazine-1-carboxylate (2.56 g,

7.5 mmol, 1.5 equiv) in CH_3CN (20 mL) at 80 ºC for 24 h, purified by column chromatography on silica gel with n-Hexane/EtOAc (10:1, v/v) as the eluent. Colorless oil, 0.95 g, 52% yield; **^{1}H NMR** (400 MHz, $CDCl_3$) δ 7.42 – 7.35 (m, 2H), 7.34 – 7.25 (m, 3H), 6.93 (dt, *J* = 16.3, 2.7 Hz, 1H), 6.38 (dt, *J* = 16.3, 11.0 Hz, 1H), 3.60 (t, *J* = 5.2 Hz, 4H), 3.41 (qd, *J* = 7.2, 4.6 Hz, 4H), 1.40 (s, 9H). **^{13}C {^{1}H} NMR** (101 MHz, $CDCl_3$) δ 162.1 (t, *J* = 30.5 Hz), 154.4, 135.7 (t, *J* = 9.7 Hz), 134.1, 129.5, 128.8, 127.4, 119.9 (t, *J* = 24.2 Hz), 115.2 (t, *J* = 249.5 Hz), 80.5, 45.8 (t, *J* = 4.9 Hz), 43.1, 28.3. **^{19}F NMR** (377 MHz, $CDCl_3$) δ -95.04 (d, *J* = 11.1 Hz, 2F); **HRMS (EI)** m/z calcd for $C_{19}H_{24}F_2N_2O_3$ $[M+Na]^+$: 389.1653; Found: 389.1658.

(*E*)-1-(azepan-1-yl)-2,2-difluoro-4-phenylbut-3-en-1-one (3h)

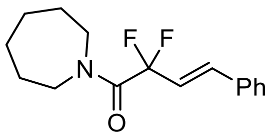

According to general procedure A, with CuI (47.7 mg, 0.25 mmol, 5.0 mol%), Styrene (5.0 mmol, 1.0 equiv), PMDETA (1.30 g, 7.5 mmol, 1.5 equiv) and 1-(azepan-1-yl)-2-bromo-2,2-difluoroethan-1-one (1.91 g, 7.5 mmol, 1.5 equiv) in CH_3CN (20 mL) at 80 ºC for 24 h, purified by column chromatography on silica gel with n-Hexane/EtOAc (10:1, v/v) as the eluent. Colorless oil, 0.77 g, 55% yield; **^{1}H NMR** (400 MHz, $CDCl_3$) δ 7.48 (dd, *J* = 7.8, 1.8 Hz, 2H), 7.43 – 7.32 (m, 3H), 7.02 (dt, *J* = 16.3, 2.8 Hz, 1H), 6.50 (dt, *J* = 16.3, 10.9 Hz, 1H), 3.68 – 3.63 (m, 2H), 3.63 – 3.57 (m, 2H), 1.88 – 1.74 (m, 4H), 1.69 – 1.51 (m, 4H). **^{13}C {^{1}H} NMR** (101 MHz, $CDCl_3$) δ 163.1 (t, *J* = 30.0 Hz), 135.2 (t, *J* = 9.7 Hz), 134.4, 129.3, 128.7, 127.3, 120.6 (t, *J* = 24.8 Hz), 115.4 (t, *J* = 249.2 Hz), 48.2, 48.0 (t, *J* = 4.9 Hz), 29.6, 27.4, 26.5, 26.0. **^{19}F NMR** (377 MHz, $CDCl_3$) δ -95.20 (d, *J* = 10.9 Hz, 2F); **HRMS (EI)** m/z calcd for $C_{16}H_{19}F_2NO$ $[M+Na]^+$: 302.1332; Found: 302.1342.

Ethyl (*S*,*E*)-4-(4-(2-((tert-butoxycarbonyl)amino)-3-methoxy-3-oxopropyl)phenyl)-2,2-difluorobut-3-enoate (5a)

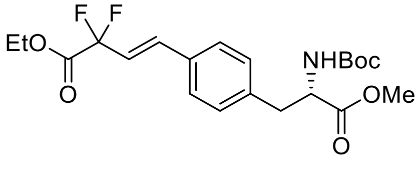

According to general procedure A, with CuI (47.7 mg, 0.25 mmol, 5.0 mol%), **Styrene-5a** (5.0 mmol,

1.0 equiv), PMDETA (1.30 g, 7.5 mmol, 1.5 equiv) and ethyl bromodifluoroacetate (1.5 g, 7.5 mmol, 1.5 equiv) in CH_3CN (20 mL) at 80 °C for 24 h, purified by column chromatography on silica gel with n-Hexane/EtOAc (10:1, v/v) as the eluent. White solid, 1.56 g, 73% yield; **^{1}H NMR** (400 MHz, $CDCl_3$) δ 7.39 (d, *J* = 8.0 Hz, 2H), 7.15 (d, *J* = 7.9 Hz, 2H), 7.05 (dt, *J* = 16.2, 2.5 Hz, 1H), 6.29 (dt, *J* = 16.2, 11.4 Hz, 1H), 5.03 (d, *J* = 8.2 Hz, 1H), 4.60 (q, *J* = 6.6 Hz, 1H), 4.36 (q, *J* = 7.1 Hz, 2H), 3.73 (s, 3H), 3.16 (dd, *J* = 13.8, 5.8 Hz, 1H), 3.06 (dd, *J* = 13.8, 6.2 Hz, 1H), 1.43 (s, 9H), 1.37 (t, *J* = 7.1 Hz, 3H). **^{13}C {^{1}H} NMR** (101 MHz, $CDCl_3$) δ 172.1, 163.8 (t, *J* = 34.9 Hz), 137.9, 136.3 (t, *J* = 9.4 Hz), 132.9, 129.8, 127.6, 118.6 (t, *J* = 24.9 Hz), 112.7 (t, *J* = 248.6 Hz), 80.0, 63.1, 54.3, 52.2, 38.1, 28.2, 13.9. **^{19}F NMR** (377 MHz, $CDCl_3$) δ -103.15 – -103.32 (m, 2F); **HRMS (EI)** m/z calcd for $C_{21}H_{27}F_2NO_6$ $[M+Na]^+$: 450.1704; Found: 450.1709.

(1*R*,2*S*,4*R*)-1,7,7-trimethylbicyclo[2.2.1]heptan-2-yl (*E*)-2,2-difluoro-4-phenylbut-3-enoate (5b)

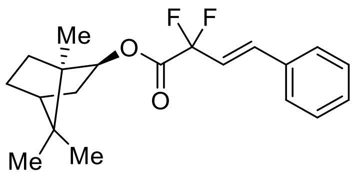

According to general procedure A, with CuI (47.7 mg, 0.25 mmol, 5.0 mol%), **Styrene** (5.0 mmol, 1.0 equiv), PMDETA (1.30 g, 7.5 mmol, 1.5 equiv) and (1*R*,2*S*,4*R*)-1,7,7-trimethylbicyclo[2.2.1]heptan-2-yl 2-bromo-2,2-difluoroacetate (2.3 g, 7.5 mmol, 1.5 equiv) in CH_3CN (20 mL) at 80 °C for 24 h, purified by column chromatography on silica gel with n-Hexane/EtOAc (40:1, v/v) as the eluent. White solid, 1.42 g, 85% yield; **^{1}H NMR** (500 MHz, $CDCl_3$) δ 7.42 (dd, *J* = 7.8, 1.9 Hz, 2H), 7.39 – 7.28 (m, 3H), 7.08 (dt, *J* = 16.3, 2.6 Hz, 1H), 6.32 (dt, *J* = 16.3, 11.2 Hz, 1H), 5.06 (dt, *J* = 10.1, 2.8 Hz, 1H), 2.40 (m, 1H), 1.97 (m, 1H), 1.77 (m, 1H), 1.71 (t, *J* = 4.5 Hz, 1H), 1.40 – 1.30 (m, 1H), 1.29 – 1.20 (m, 1H), 1.07 (dd, *J* = 14.0, 3.4 Hz, 1H), 0.90 (s, 3H), 0.88 (s, 3H), 0.86 (s, 3H). **^{13}C {^{1}H} NMR** (126 MHz, $CDCl_3$) δ 164.1 (t, *J* = 34.6 Hz), 136.8 (t, *J* = 9.2 Hz), 134.2, 129.6, 128.9, 127.4, 119.2 (t, *J* = 25.3 Hz), 112.9 (t, *J* = 248.6 Hz), 83.1, 49.2, 48.0, 44.8, 36.4, 27.9,

27.0, 19.6, 18.8, 13.4. **^{19}F {^{1}H} NMR** (470 MHz, $CDCl_3$) δ -102.79 (s, 2F); **HRMS (EI)** m/z calcd for $C_{20}H_{24}F_2O_2$ $[M+H]^+$: 335.1823 Found:335.1830.

Ethyl (*E*)-2,2-difluoro-4-((8*R*,9*S*,13*S*,14*S*,17*S*)-17-methoxy-13-methyl-7,8,9,11,12,13,14,15,16,17-decahydro-6*H*-cyclopenta[*a*]phenanthren-3-yl)but-3-enoate (5c)

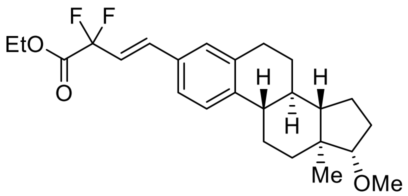

According to general procedure A, with CuI (47.7 mg, 0.25 mmol, 5.0 mol%), **Styrene-5c** (5.0 mmol, 1.0 equiv), PMDETA (1.30 g, 7.5 mmol, 1.5 equiv) and bromodifluoroacetate (1.5 g, 7.5 mmol, 1.5 equiv) in CH_3CN (20 mL) at 80 ^{o}C for 24 h, purified by column chromatography on silica gel with n-Hexane/EtOAc (30:1, v/v) as the eluent. White solid, 1.42 g, 68% yield; **^{1}H NMR** (500 MHz, $CDCl_3$) δ 7.27 (d, *J* = 8.2 Hz, 1H), 7.20 (dd, *J* = 8.1, 1.9 Hz, 1H), 7.14 (s, 1H), 7.01 (dt, *J* = 16.1, 2.4 Hz, 1H), 6.25 (dt, *J* = 15.7, 11.3 Hz, 1H), 4.41 – 4.21 (m, 2H), 3.36 (s, 3H), 3.29 (t, *J* = 8.3 Hz, 1H), 2.84 (dd, *J* = 9.2, 4.2 Hz, 2H), 2.28 (dd, *J* = 13.4, 3.6 Hz, 1H), 2.19 (td, *J* = 11.2, 3.8 Hz, 1H), 2.12 – 2.00 (m, 2H), 1.93 – 1.82 (m, 1H), 1.73 – 1.63 (m, 1H), 1.57 – 1.45 (m, 2H), 1.44 – 1.25 (m, 7H), 1.22 – 1.11 (m, 1H), 0.78 (s, 3H). **^{13}C {^{1}H} NMR** (126 MHz, $CDCl_3$) δ 164.0 (t, *J* = 35.0 Hz), 142.4, 137.2, 136.7 (t, *J* = 9.3 Hz), 131.4, 128.0, 125.9, 124.7, 117.8 (t, *J* = 24.9 Hz), 112.9 (t, *J* = 248.3 Hz), 90.7, 63.0, 57.8, 50.4, 44.4, 43.1, 38.2, 38.0, 29.4, 27.7, 27.0, 26.1, 23.0, 13.9, 11.5. **^{19}F {^{1}H} NMR** (470 MHz, $CDCl_3$) δ -102.84 (s, 2F); **HRMS (EI)** m/z calcd for $C_{25}H_{32}F_2O_3$ $[M+Na]^+$: 441.2217; Found: 441.2208.

(*R*,*E*)-2,2-Difluoro-*N*-methyl-4-phenyl-*N*-(3-phenyl-3-(*o*-tolyloxy)propyl)but-3-enamide (5d)

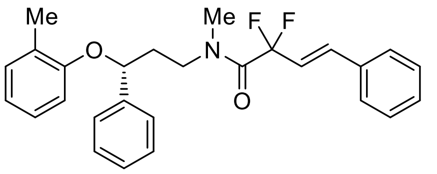

According to general procedure A, with CuI (28.7 mg, 0.15 mmol, 5.0 mol%), **Styrene** (3.0 mmol, 1.0 equiv), PMDETA (0.80 g, 4.5 mmol, 1.5 equiv) and (*R*)-2-bromo-2,2-difluoro-*N*-methyl-*N*-(3-

phenyl-3-(o-tolyloxy)propyl)acetamide (1.85 g, 4.5 mmol, 1.5 equiv) in CH_3CN (20 mL) at 80 °C for 24 h, purified by column chromatography on silica gel with n-Hexane/EtOAc (10:1, v/v) as the eluent. Colorless oil, 1.11 g, 85% yield; **^{1}H NMR** (400 MHz, $CDCl_3$) δ 7.46 (ddd, *J* = 7.5, 6.0, 1.8 Hz, 2H), 7.37 (m, 6H), 7.33 – 7.24 (m, 2H), 7.16 (td, *J* = 7.2, 1.6 Hz, 1H), 7.05 – 6.93 (m, 2H), 6.82 (qd, *J* = 7.3, 1.0 Hz, 1H), 6.59 (dd, *J* = 17.7, 8.2 Hz, 1H), 6.45 (m, 1H), 5.23 (td, *J* = 8.7, 4.0 Hz, 1H), 3.85 – 3.59 (m, 2H), 3.22 – 3.01 (m, 3H), 2.38 (s, 3H), 2.36 – 2.19 (m, 2H). **^{13}C {^{1}H} NMR** (126 MHz, $CDCl_3$) δ 163.2 (t, *J* = 30.2 Hz), 155.6, 155.5, 141.3, 140.9, 135.4 (t, *J* = 10.2 Hz), 134.3, 134.3, 130.8, 130.7, 129.4, 129.4, 128.8, 128.8, 128.7, 127.8, 127.7, 127.4, 126.6, 125.6, 125.5, 120.5, 120.4, 120.1 (t, *J* = 23.9 Hz), 115.3 (t, *J* = 249.2), 115.2 (t, *J* = 249.6), 112.6, 112.5, 47.3, 47.0 (t, *J* = 4.7 Hz), 37.6, 35.8, 35.0, 16.5. **^{19}F NMR** (377 MHz, $CDCl_3$) δ -94.53 – -96.50 (m, 2F); **HRMS (EI)** m/z calcd for $C_{18}H_{25}BF_2O_4$ $[M+Na]^+$: 458.1908; Found: 458.1917.

General procedure B:

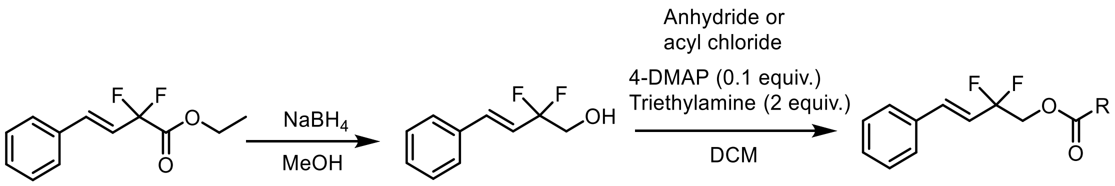

According to the literature procedure,[2] to a stirred solution of **1a** (20 mmol) in MeOH (40 mL) was added $NaBH_4$ (20 mmol) in portions at 0 °C. The resulting solution was stirred at the same temperature for 1 hour, water (8 mL) and saturated NH_4Cl solution (20 mL) were added to quench the reaction. The mixture was then extracted with EtOAc (20 mL × 3). The organic layers were combined, washed with water and brine, dried over anhydrous Na_2SO_4. After removal of the solvent, the residue was purified by flash chromatography on silica gel using EtOAc/n-Hexane (1:5) as the eluent to afford the (*E*)-2,2-difluoro-4-phenylbut-3-en-1-ol as a white solid.

To a stirred solution of the (*E*)-2,2-difluoro-4-phenylbut-3-en-1-ol (5 mmol), 4-dimethylaminopyridine (62 mg, 0.1 equiv) and Et_3N (2 equiv) in DCM (10 mL) at 0 °C, the corresponding anhydride or acyl chloride (1.2 equivalents) were added dropwise to the mixture. After the mixture was stirred at room temperature overnight, the saturated NH_4Cl was added to quench the reaction. The mixture was then extracted with EtOAc (20 mL × 3), washed with water and brine, dried over anhydrous Na_2SO_4. After removal of the solvent, the residue was purified by flash chromatography on silica gel with EtOAc/n-Hexane (1:40) as eluent to afford the desired product (**3i**-**3k**) as a colorless oil or white solid (70-88% yields).

(*E*)-2,2-Difluoro-4-phenylbut-3-en-1-yl acetate (3i)

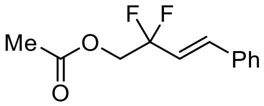

According to general procedure B, with 4-DMAP (61.1 mg, 0.5 mmol, 10.0 mol%), **(*E*)-2,2-difluoro-4-phenylbut-3-en-1-ol** (0.92 g, 5.0 mmol, 1.0 equiv), triethylamine (1.01 g, 10 mmol, 2.0 equiv) and Acetic anhydride (0.76 g, 7.5 mmol, 1.5 equiv) in DCM (20 mL) at room temperature for overnight, purified by column chromatography on silica gel with n-Hexane/EtOAc (30:1, v/v) as the eluent. Colorless oil, 0.79 g, 70% yield; **^{1}H NMR** (400 MHz, $CDCl_3$) δ 7.41 – 7.34 (m, 2H), 7.34 – 7.24 (m, 3H), 6.93 (dt, *J* = 16.2, 2.6 Hz, 1H), 6.12 (dt, *J* = 16.3, 11.4 Hz, 1H), 4.33 (t, *J* = 12.6 Hz, 2H), 2.06 (s, 3H). **^{13}C {^{1}H} NMR** (101 MHz, $CDCl_3$) δ 169.9, 135.8 (t, *J* = 9.4 Hz), 134.4, 129.3, 128.8, 127.2, 120.0 (t, *J* = 25.1 Hz), 118.3 (t, *J* = 240.7 Hz), 64.4 (t, *J* = 33.9 Hz), 20.6. **^{19}F NMR** (377 MHz, $CDCl_3$) δ -103.37 (qd, *J* = 12.4, 2.7 Hz, 2F); **HRMS (EI)** m/z calcd for $C_{12}H_{12}F_2O_2$ $[M+H]^+$: 227.0884; Found: 227.0882.

(*E*)-2,2-difluoro-4-phenylbut-3-en-1-yl pivalate (3j)

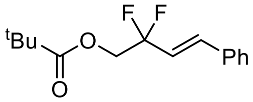

According to general procedure B, with 4-DMAP (61.1 mg, 0.5 mmol, 10.0 mol%), **(*E*)-2,2-difluoro-4-phenylbut-3-en-1-ol** (0.92 g, 5.0 mmol, 1.0 equiv), triethylamine (1.01 g, 10 mmol, 2.0 equiv) and trimethylacetic anhydride (1.91 g, 7.5 mmol, 1.5 equiv) in DCM (20

mL) at room temperature for overnight, purified by column chromatography on silica gel with n-Hexane/EtOAc (30:1, v/v) as the eluent. Colorless oil, 1.14 g, 85% yield; **^{1}H NMR** (400 MHz, $CDCl_3$) δ 7.37 – 7.32 (m, 2H), 7.32 – 7.23 (m, 3H), 6.91 (dt, *J* = 16.2, 2.5 Hz, 1H), 6.10 (dt, *J* = 16.2, 11.3 Hz, 1H), 4.33 (t, *J* = 12.3 Hz, 2H), 1.14 (s, 9H). **^{13}C {^{1}H} NMR** (101 MHz, $CDCl_3$) δ 177.4, 135.7 (t, *J* = 9.4 Hz), 134.5, 129.3, 128.8, 127.2, 120.2 (t, *J* = 25.3 Hz), 118.5 (t, *J* = 240.8 Hz), 64.1 (t, *J* = 34.6 Hz), 38.8, 27.0. **^{19}F NMR** (377 MHz, $CDCl_3$) δ -103.26 (qd, *J* = 12.0, 2.6 Hz, 2F); **HRMS (EI)** m/z calcd for $C_{15}H_{18}F_2O_2$ $[M+H]^+$: 269.1353; Found: 269.1363.

(*E*)-2,2-Difluoro-4-phenylbut-3-en-1-yl benzoate (3k)

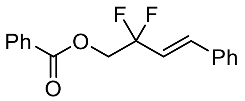

According to general procedure B, with 4-DMAP (61.1 mg, 0.5 mmol, 10.0 mol%), **(*E*)-2,2-difluoro-4-phenylbut-3-en-1-ol** (1.84 g, 5.0 mmol, 1.0 equiv), triethylamine (1.01 g, 10 mmol, 2.0 equiv) and benzoyl chloride (1.05 g, 7.5 mmol, 1.5 equiv) in DCM (20 mL) at room temperature for overnight, purified by column chromatography on silica gel with n-Hexane/EtOAc (30:1, v/v) as the eluent. Colorless oil, 1.27 g, 88% yield; **^{1}H NMR** (400 MHz, $CDCl_3$) δ 7.99 – 7.93 (m, 2H), 7.51 – 7.42 (m, 1H), 7.37 – 7.29 (m, 4H), 7.28 – 7.20 (m, 3H), 6.95 (dt, *J* = 16.3, 2.6 Hz, 1H), 6.17 (dt, *J* = 16.2, 11.3 Hz, 1H), 4.54 (t, *J* = 12.4 Hz, 2H). **^{13}C {^{1}H} NMR** (101 MHz, $CDCl_3$) δ 165.6, 136.0 (t, *J* = 9.4 Hz), 134.5, 133.5, 129.9, 129.3, 129.2, 128.8, 128.5, 127.3, 120.1 (t, *J* = 25.2 Hz), 118.5 (t, *J* = 240.8 Hz), 64.8 (t, *J* = 34.1 Hz). **^{19}F NMR** (377 MHz, $CDCl_3$) δ -102.94 (qd, *J* = 12.2, 2.6 Hz, 2F); **HRMS (EI)** m/z calcd for $C_{17}H_{14}F_2O_2$ $[M+H]^+$: 289.1040; Found: 289.1048.

(*E*)-2,2-Difluoro-4-phenylbut-3-en-1-yl 2-(4-(4-chlorobenzoyl)phenoxy)-2-methylpropanoate (5f)

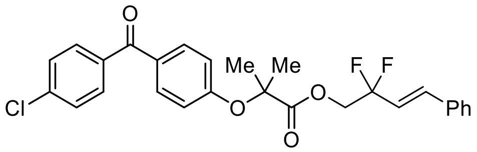

According to the literature procedure,[3] 1-(3-dimethylaminopropyl)-3-ethylcarbodiimide hydrochloride (EDCI) (1.15 g, 6 mmol), *N*-

hydroxybenzotriazole (HOBt) (0.92 g, 6 mmol) and **(*E*)-2,2-difluoro-4-phenylbut-3-en-1-ol** (0.92 g, 5.0 mmol) were added to a solution of fenofibric acid (1.59 g, 5 mmol) in DCM (20 mL) at room temperature. The reaction mixture was stirred overnight at room temperature and purified by column chromatography on silica gel with n-Hexane/EtOAc (30:1, v/v) as the eluent. Colorless oil, 1.14 g, 85% yield; **^{1}H NMR** (400 MHz, $CDCl_3$) δ 7.60 – 7.54 (m, 4H), 7.39 – 7.32 (m, 2H), 7.28 – 7.18 (m, 5H), 6.84 (dt, *J* = 16.2, 2.6 Hz, 1H), 6.78 – 6.72 (m, 2H), 5.99 (dt, *J* = 16.3, 11.4 Hz, 1H), 4.43 (t, *J* = 12.1 Hz, 2H), 1.62 (s, 6H). **^{13}C {^{1}H} NMR** (101 MHz, $CDCl_3$) δ 194.1, 172.7, 159.2, 138.3, 136.3, 136.1 (t, *J* = 9.4 Hz), 134.1, 132.0, 131.1, 130.5, 129.4, 128.8, 128.5, 127.2, 119.5 (t, *J* = 25.0 Hz), 118.0 (t, *J* = 241.2 Hz), 117.4, 79.2, 64.9 (t, *J* = 35.0 Hz), 25.4. **^{19}F NMR** (377 MHz, $CDCl_3$) δ -102.93 (qd, *J* = 12.0, 2.7 Hz, 2F); **HRMS (EI)** m/z calcd for $C_{27}H_{23}ClF_2O_4$ $[M+Na]^+$: 507.1151; Found: 507.1157.

3. General Procedure for the synthesis of fluoroalkyl boronates 2, 4 and 6.

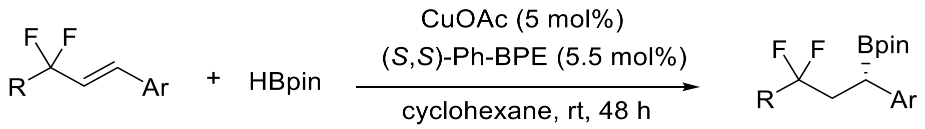

In an Ar-filled dry glovebox, CuOAc (1.2 mg, 10 μmol), (*S*, *S*)-Ph-BPE (5.6 mg, 11 μmol), cyclohexane (1 mL), and a magnetic stirring bar were added to a 4 mL screw-capped vial and stirred for 30 mins. The difluoromethyl alkenes (0.2 mmol) were added and stirred for further 5 min, then HBpin (38.4 mg, 0.3 mmol) was added. The vial was sealed with a cap containing a PTFE septum and removed from the dry box. The reaction mixture was stirred at room temperature for 48 h and the resulting solution was concentrated in vacuum. The residue was purified by silica gel flash column chromatography with EtOAc/n-Hexane as eluent to afford the desired products. The characterization data of these chiral alkylboronates were listed below.

Ethyl (*S*)-2,2-difluoro-4-phenyl-4-(4,4,5,5-tetramethyl-1,3,2-dioxaborolan-2-yl) butanoate (**2a**).

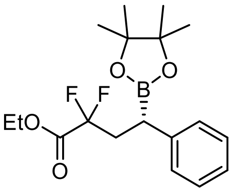

Colorless oil (55.2 mg, 78% yield, 99% ee); **^{1}H NMR** (400 MHz, $CDCl_3$) δ 7.34 – 7.11 (m, 5H), 4.23 – 4.05 (m, 2H), 2.80 – 2.58 (m, 1H), 2.56 – 2.37 (m, 1H), 1.29 (t, *J* = 7.1 Hz, 3H), 1.21 (s, 6H), 1.19 (s, 6H); **^{13}C {^{1}H} NMR** (101 MHz, $CDCl_3$) δ 164.1 (t, *J* = 32.9 Hz), 140.7 , 128.4 , 128.2, 125.8, 116.0 (dd, *J* = 251.6, 249.4 Hz), 83.7, 62.5, 37.0 (t, *J* = 23.0 Hz), 26.9, 24.4 (d, *J* = 3.3 Hz), 13.8; **^{19}F NMR** (377 MHz, $CDCl_3$) δ -102.24 – -103.31 (m, 1F), -106.73 – -107.93 (m, 1F), **^{11}B NMR** (128 MHz, $CDCl_3$) δ 32.37; **HRMS (EI)** m/z calcd for $C_{18}H_{25}BF_2O_4$ $[M+Na]^+$: 377.1712; Found: 377.1711.

Optical rotation: $[\alpha]_D^{25}$: 4.4 (*c* = 0.80, $CHCl_3$).

HPLC condition: Chiral column OD-H, n-Hexane/^{i}PrOH = 99.5:0.5, flow rate = 0.5 mL/min, wavelength = 220 nm, t_R = 10.7 min for the major isomer, t_R = 10.0 min for the minor isomer.

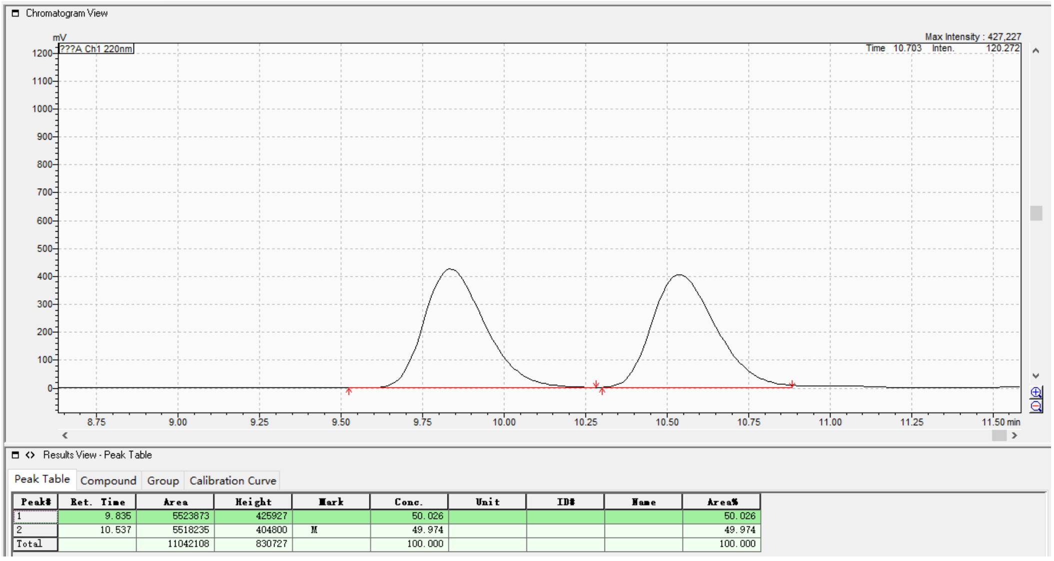

Peak#	Ret. Time	Area	Height	Mark	Conc.	Unit	ID#	Name	Area%
1	9.835	5523873	425927		50.026				50.026
2	10.537	5518235	404800	M	49.974				49.974
Total		11042108	830727		100.000				100.000

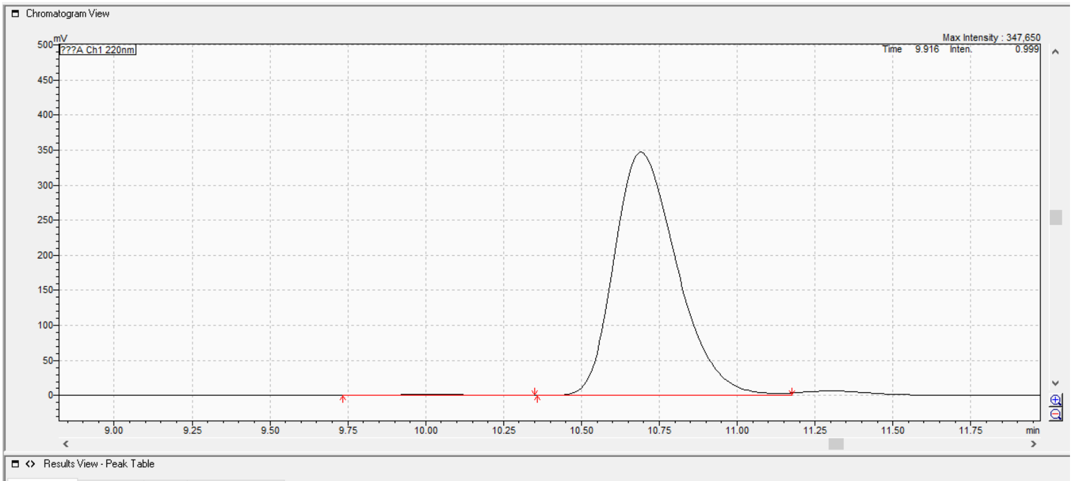

Peak#	Ret. Time	Area	Height	Mark	Conc.	Unit	ID#	Name	Area%
1	10.017	24087	1869		0.493				0.493
2	10.691	4862110	347503	M	99.507				99.507
Total		4886198	349372		100.000				100.000

Ethyl (*S*)-2,2-difluoro-4-(4,4,5,5-tetramethyl-1,3,2-dioxaborolan-2-yl)-4-(*p*-tolyl)butanoate (**2b**).

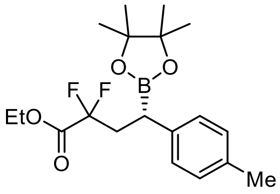

Colorless oil (66.2 mg, 90% yield, 92% ee); **^{1}H NMR** (400 MHz, $CDCl_3$) δ 7.17 – 7.02 (m, 4H), 4.26 – 4.01 (m, 2H), 2.77 – 2.64 (m, 1H), 2.61 (dt, *J* = 8.6, 4.4 Hz, 1H), 2.52 – 2.35 (m, 1H), 2.32 (s, 3H), 1.29 (t, *J* = 7.1 Hz, 3H), 1.21 (s, 6H), 1.19 (s, 6H); **^{13}C {^{1}H} NMR** (101 MHz, $CDCl_3$) δ 164.1 (t, *J* = 32.9 Hz), 137.6, 135.2, 129.1, 128.1, 116.0 (dd, *J* = 251.6, 249.4 Hz), 83.7, 62.5, 37.2 (t, *J* = 22.9 Hz), 24.4, 24.4, 20.9, 13.8; **^{19}F NMR** (377 MHz, $CDCl_3$) δ -102.06 – -103.46 (m, 1F), -106.56 – -107.90 (m, 1F), **^{11}B NMR** (128 MHz, $CDCl_3$) δ 32.70; **HRMS (EI)** m/z calcd for $C_{19}H_{27}BF_2O_4$ $[M+Na]^+$: 391.1868; Found: 391.1867.

Optical rotation: $[\alpha]_D^{25}$: 2.4 (*c* = 1.0, $CHCl_3$).

HPLC condition: Chiral column OD-H, n-Hexane/i-PrOH = 99.5:0.5, flow rate = 0.5 mL/min, wavelength = 220 nm, t_R = 15.4 min for the major isomer, t_R = 14.7 min for the minor isomer.

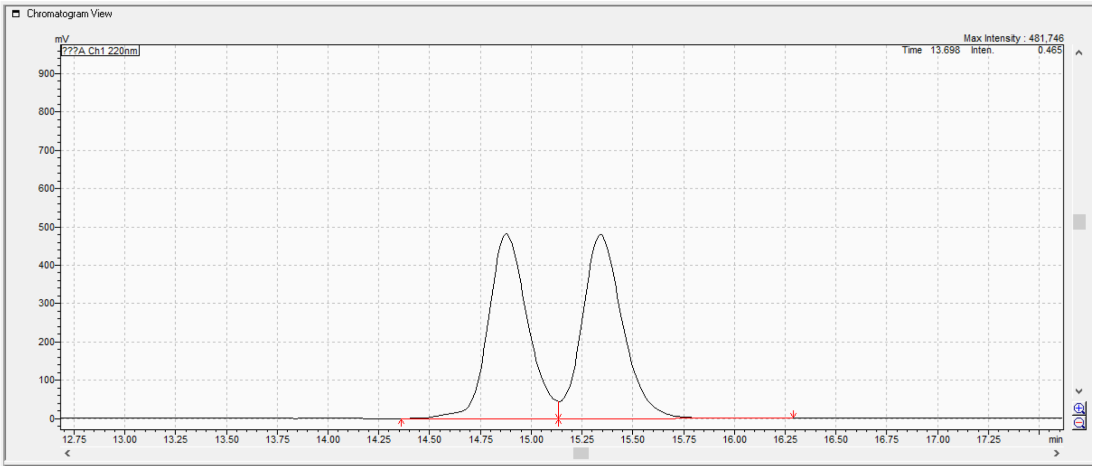

Results View - Peak Table

Peak Table | Compound | Group | Calibration Curve

Peak#	Ret. Time	Area	Height	Mark	Conc.	Unit	ID#	Name	Area%
1	14.878	6472976	481585		49.547				49.547
2	15.341	6591377	479433	V	50.453				50.453
Total		13064354	961018		100.000				100.000

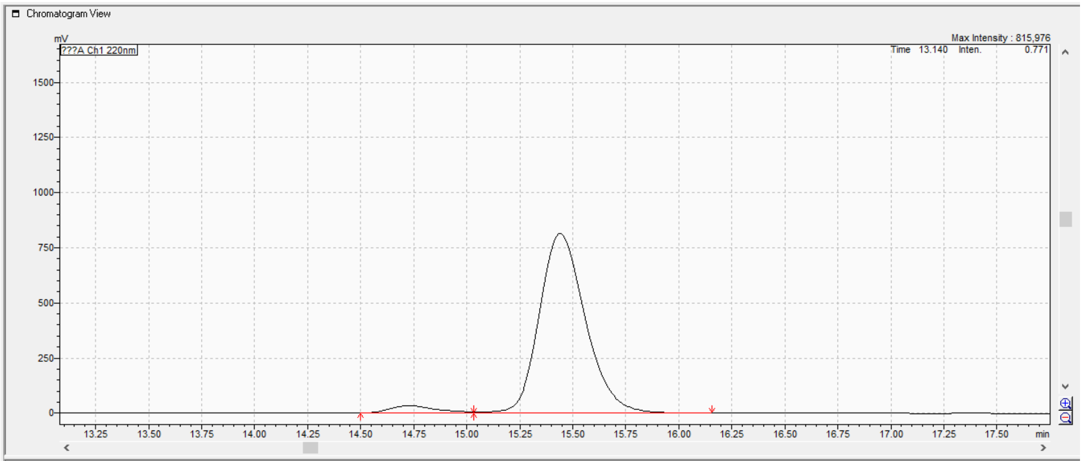

Results View - Peak Table

Peak Table | Compound | Group | Calibration Curve

Peak#	Ret. Time	Area	Height	Mark	Conc.	Unit	ID#	Name	Area%
1	14.725	520998	33794	M	4.085				4.085
2	15.441	12232230	815698	V M	95.915				95.915
Total		12753229	849492		100.000				100.000

Ethyl (*S*)-2,2-difluoro-4-(4,4,5,5-tetramethyl-1,3,2-dioxaborolan-2-yl)-4-(*m*-tolyl) butanoate (**2c**).

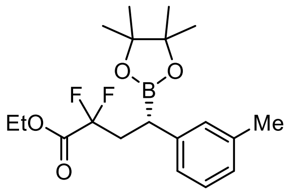

Colorless oil (68.5 mg, 93% yield, 94% ee); ^{1}**H NMR** (400 MHz, $CDCl_3$) δ 7.17 (t, *J* = 7.5 Hz, 1H), 7.09 – 6.95 (m, 3H), 4.26 – 4.06 (m, 2H), 2.80 – 2.64 (m, 1H), 2.67 – 2.56 (m, 1H), 2.45 (tdd, *J* = 18.7, 14.0, 5.6 Hz, 1H), 2.33 (s, 3H), 1.29 (t, *J* = 7.1 Hz, 3H), 1.22 (s, 6H), 1.19 (s, 6H); ^{13}**C {^{1}H} NMR** (101 MHz, $CDCl_3$) δ 164.1 (t, *J* = 32.9 Hz), 140.6, 137.9, 129.1, 128.3, 126.6, 125.2, 116.0 (dd, *J* = 251.5, 249.4 Hz), 83.7, 62.5, 37.1 (t, *J* = 22.9 Hz), 24.4, 24.4, 21.3, 13.8; ^{19}**F NMR** (377 MHz,

$CDCl_3$) δ -102.20 – -103.39 (m, 1F), -106.80 – -107.92 (m, 1F), **^{11}B NMR** (128 MHz, $CDCl_3$) δ 33.31; **HRMS (EI)** m/z calcd for $C_{19}H_{27}BF_2O_4$ $[M+Na]^+$: 391.1868; Found: 391.1877.

Optical rotation: $[\alpha]_D^{25}$: 1.9 (c = 1.1, $CHCl_3$).

HPLC condition: Chiral column OD-H, n-Hexane/i-PrOH = 99.9:0.1, flow rate = 0.5 mL/min, wavelength = 220 nm, t_R = 14.3 min for the major isomer, t_R = 13.9 min for the minor isomer.

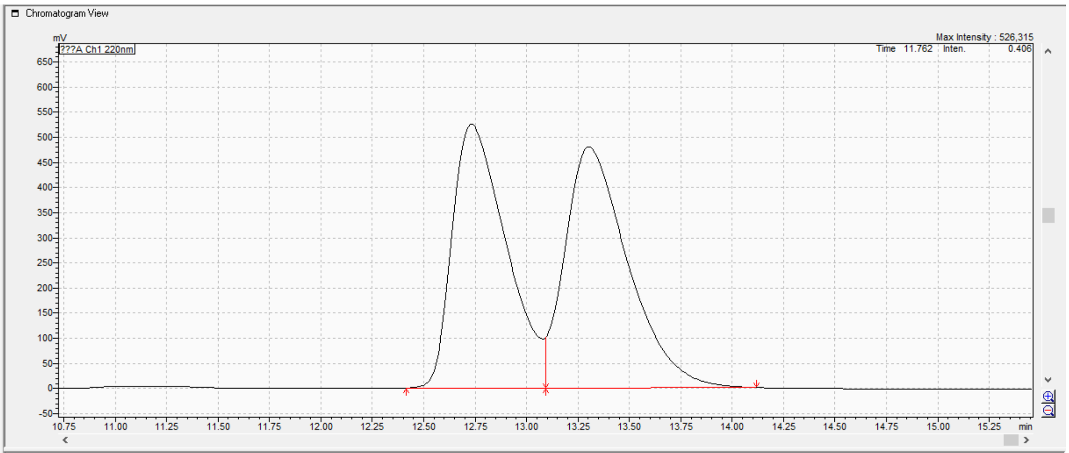

Results View - Peak Table

Peak Table | Compound | Group | Calibration Curve

Peak#	Ret. Time	Area	Height	Mark	Conc.	Unit	ID#	Name	Area%
1	12.732	9595342	525184	M	48.527				48.527
2	13.303	10177713	479709	V M	51.473				51.473
Total		19773056	1004893		100.000				100.000

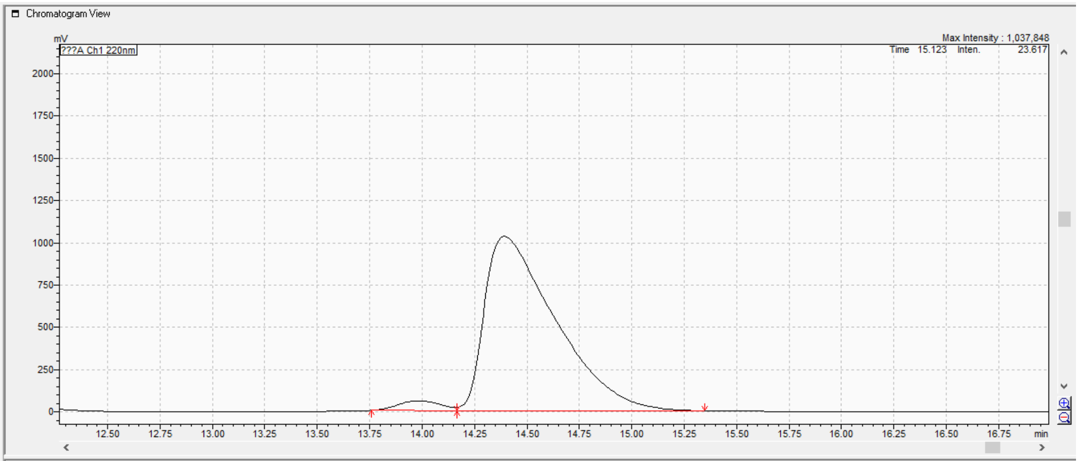

Results View - Peak Table

Peak Table | Compound | Group | Calibration Curve

Peak#	Ret. Time	Area	Height	Mark	Conc.	Unit	ID#	Name	Area%
1	13.981	796727	56585	M	3.193				3.193
2	14.392	24152542	1029771	V M	96.807				96.807
Total		24949269	1086356		100.000				100.000

Ethyl (*S*)-2,2-difluoro-4-(4,4,5,5-tetramethyl-1,3,2-dioxaborolan-2-yl)-4-(*o*-tolyl) butanoate (2d).

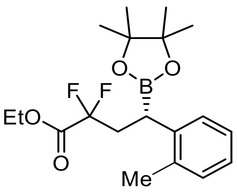

Colorless oil (53.7 mg, 73% yield, 96% ee); **^{1}H NMR** (400 MHz, $CDCl_3$) δ 7.23 – 7.18 (m, 1H), 7.16 – 7.10 (m, 2H), 7.10 – 7.04 (m, 1H), 4.20 – 4.00 (m, 2H), 2.86 (dd, *J* = 7.9, 6.5 Hz, 1H), 2.73 (dddd, *J* = 20.2, 14.7, 10.5, 8.3 Hz, 1H), 2.51 – 2.38 (m, 1H), 2.37 (s, 3H), 1.28 (t, *J* = 7.2 Hz, 3H), 1.21 (s, 6H), 1.18 (s, 6H); **^{13}C {^{1}H} NMR** (101 MHz, $CDCl_3$) δ 164.1 (t, *J* = 33.0 Hz), 139.1, 136.2, 130.4, 127.8, 125.9, 125.7, 116.1 (dd, *J* = 251.3, 249.3 Hz), 83.6, 62.5, 36.7 (t, *J* = 23.0 Hz), 24.4, 24.4, 19.9, 13.7; **^{19}F NMR** (377 MHz, $CDCl_3$) δ -102.83 (ddd, *J* = 258.3, 18.5, 10.5 Hz, 1F), -107.60 (dt, *J* = 258.4, 19.3 Hz, 1F), **^{11}B NMR** (128 MHz, $CDCl_3$) δ 33.72; **HRMS (EI)** m/z calcd for $C_{19}H_{27}BF_2O_4$ $[M+H]^+$: 369.2049; Found: 369.2046.

Optical rotation: $[\alpha]_D^{25}$: -2.4 (*c* = 1.0, $CHCl_3$).

HPLC condition: Chiral column IG, n-Hexane/i-PrOH = 99.5:0.5, flow rate = 0.5 mL/min, wavelength = 220 nm, t_R = 9.1 min for the major isomer, t_R = 9.8 min for the minor isomer.

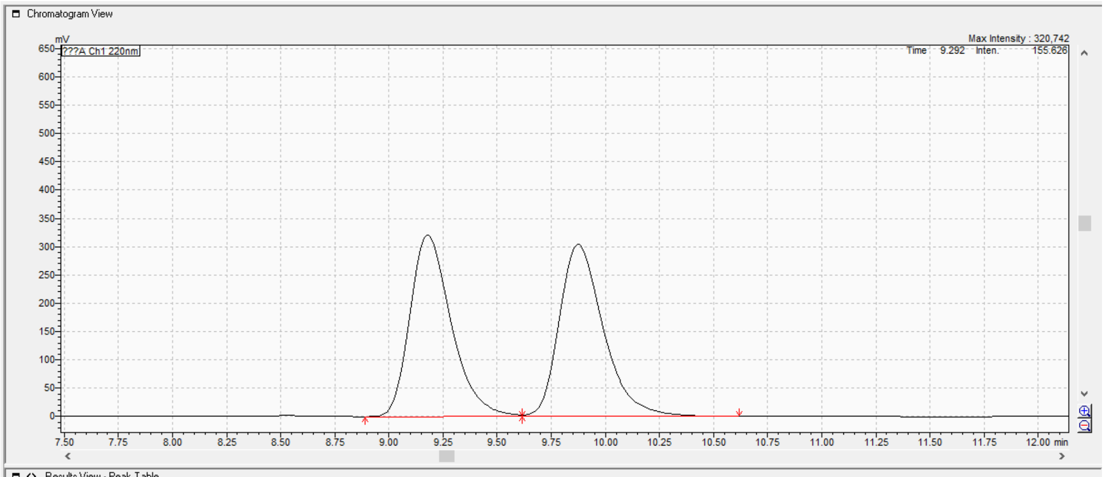

Results View - Peak Table

Peak Table | Compound | Group | Calibration Curve

Peak#	Ret. Time	Area	Height	Mark	Conc.	Unit	ID#	Name	Area%
1	9.179	4229288	320695		49.648				49.648
2	9.874	4289335	303644	V	50.352				50.352
Total		8518623	624340		100.000				100.000

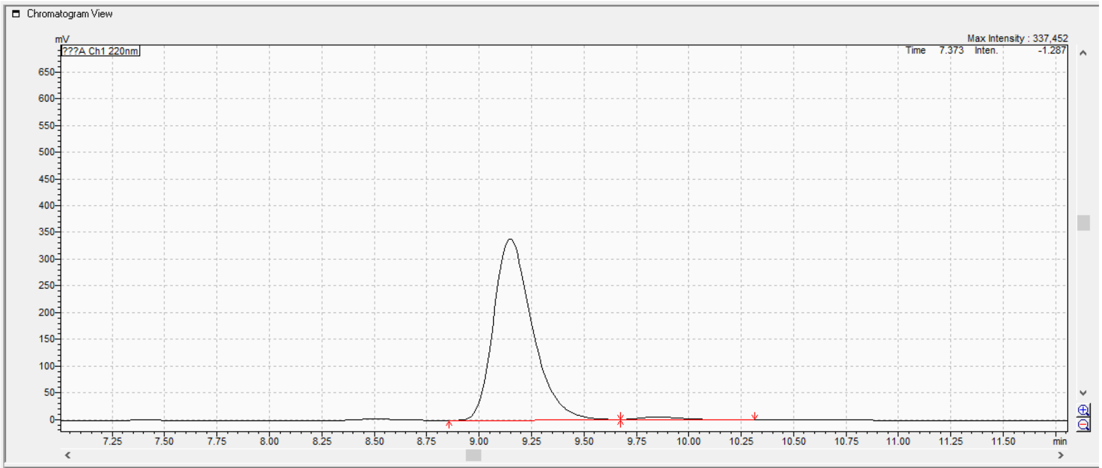

Results View - Peak Table

Peak Table | Compound | Group | Calibration Curve

Peak#	Ret. Time	Area	Height	Mark	Conc.	Unit	ID#	Name	Area%
1	9.148	4363437	338918	M	97.914				97.914
2	9.849	92952	5997	V M	2.086				2.086
Total		4456390	344915		100.000				100.000

Ethyl (*S*)-4-(4-(tert-butyl)phenyl)-2,2-difluoro-4-(4,4,5,5-tetramethyl-1,3,2-dioxaborolan-2-yl) butanoate (2e).

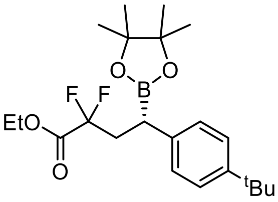

Colorless oil (51.7 mg, 63% yield, 99% ee); **^{1}H NMR** (400 MHz, $CDCl_3$) δ 7.33 – 7.25 (m, 2H), 7.18 – 7.10 (m, 2H), 4.20 – 3.96 (m, 2H), 2.77 – 2.64 (m, 1H), 2.61 (dd, *J* = 8.6, 5.4 Hz, 1H), 2.53 – 2.37 (m, 1H), 1.31 (s, 9H), 1.27 (t, *J* = 7.2 Hz, 3H), 1.22 (s, 6H), 1.20 (s, 6H); **^{13}C {^{1}H} NMR** (101 MHz, $CDCl_3$) δ 164.1 (t, *J* = 32.9 Hz), 148.5, 137.4, 127.9, 125.3, 116.0 (dd, *J* = 251.3, 249.2 Hz), 83.7, 62.5, 37.2 (t, *J* = 23.0 Hz), 34.3, 31.3, 24.5, 24.4, 13.8; **^{19}F NMR** (377 MHz, $CDCl_3$) δ -102.35 (ddd, *J* = 259.0, 18.4, 10.5 Hz, 1F), -107.60 (dt, *J* = 258.9, 19.2 Hz,1F), **^{11}B NMR** (128 MHz, $CDCl_3$) δ 32.61; **HRMS (EI)** m/z calcd for $C_{22}H_{33}BF_2O_4$ $[M+H]^+$: 411.2518; Found: 411.2514.

Optical rotation: $[\alpha]_D^{25}$: -3.4 (*c* = 1.0, $CHCl_3$).

HPLC condition: Chiral column OD-H, n-Hexane/i-PrOH = 99.5:0.5, flow rate = 0.5 mL/min, wavelength = 220 nm, t_R = 10.3 min for the major isomer, t_R = 9.8 min for the minor isomer.

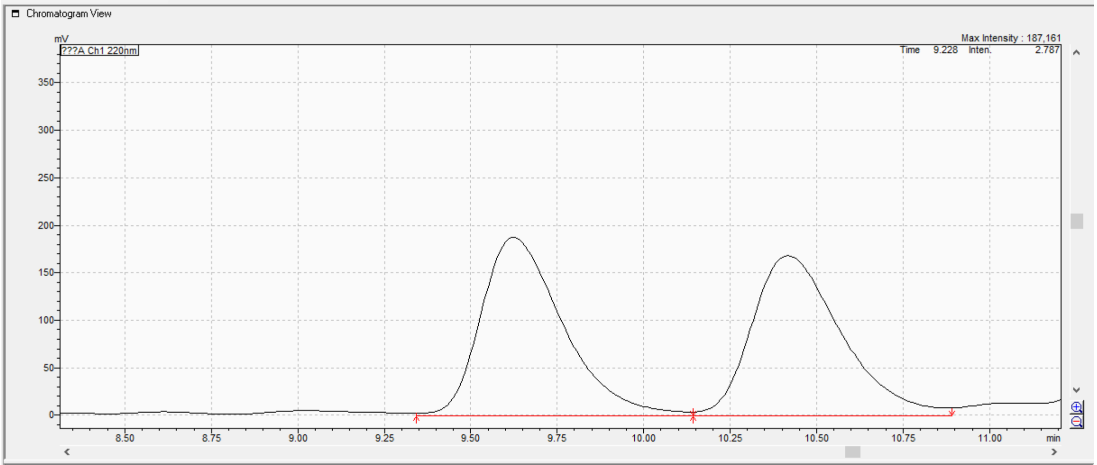

Results View - Peak Table

Peak#	Ret. Time	Area	Height	Mark	Conc.	Unit	ID#	Name	Area%
1	9.623	3090907	187570	M	50.490				50.490
2	10.417	3030863	168578	V M	49.510				49.510
Total		6121770	356148		100.000				100.000

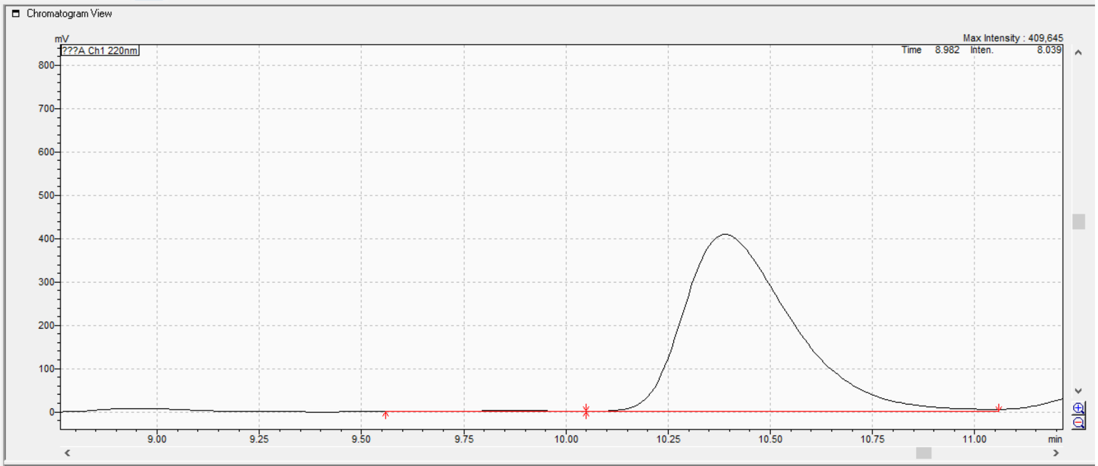

Results View - Peak Table

Peak#	Ret. Time	Area	Height	Mark	Conc.	Unit	ID#	Name	Area%
1	9.881	22518	1518	M	0.301				0.301
2	10.389	7465705	408314	M	99.699				99.699
Total		7488223	409832		100.000				100.000

Ethyl (*S*)-2,2-difluoro-4-(4-methoxyphenyl)-4-(4,4,5,5-tetramethyl-1,3,2-dioxaborolan-2-yl) butanoate (**2f**).

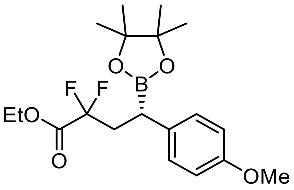

Colorless oil (62.2mg, 81% yield, 97% ee); **^{1}H NMR** (400 MHz, $CDCl_3$) δ 7.17 – 7.11 (m, 2H), 6.86 – 6.80 (m, 2H), 4.24 – 4.08 (m, 2H), 3.79 (s, 3H), 2.75 – 2.62 (m, 1H), 2.59 (dd, *J* = 8.5, 5.7 Hz, 1H), 2.51 – 2.33 (m, 1H), 1.29 (t, *J* = 7.1 Hz, 3H), 1.21 (s, 6H), 1.19 (s, 6H); **^{13}C {^{1}H} NMR** (101 MHz, $CDCl_3$) δ 164.1(t, *J* = 33.0 Hz), 157.8, 132.6, 129.2, 116.07 (dd, *J* = 251.4, 249.3 Hz), 113.9, 83.7, 62.5, 55.1, 37.3 (t, *J* = 22.9 Hz), 24.4, 24.4, 13.8; **^{19}F NMR** (377 MHz, $CDCl_3$) δ -102.75

(ddd, J = 259.0, 18.7, 10.8 Hz, 1F), -107.27 (dt, J = 258.9, 19.2 Hz, 1F), **^{11}B NMR** (128 MHz, $CDCl_3$) δ 33.35; **HRMS (EI)** m/z calcd for $C_{19}H_{27}BF_2O_5$ $[M+H]^+$: 385.1998; Found:385.2004.

Optical rotation: $[\alpha]_D^{25}$: -2.2 (c = 1.0, $CHCl_3$).

HPLC condition: Chiral column OD-H, n-Hexane/i-PrOH = 99:1, flow rate = 0.5 mL/min, wavelength = 220 nm, t_R = 10.8 min for the major isomer, t_R = 10.0 min for the minor isomer.

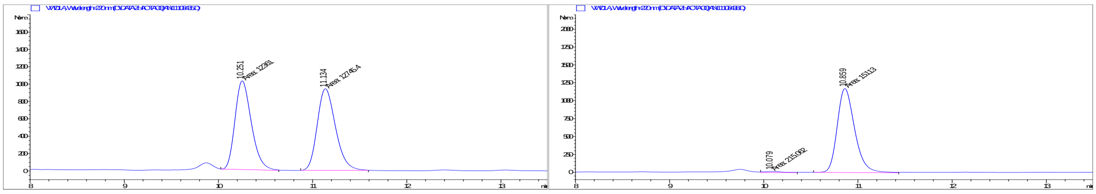

Peak	Time	Start	End	Area	Height	Area%
1	10.251	12361	1022.3	0.2015	0.753	49.234
2	11.134	12745.4	941.6	0.2256	0.772	50.766

Peak	Time	Start	End	Area	Height	Area%
1	10.079	215.1	15.2	0.2352	0.615	1.403
2	10.859	15113	1172	0.2149	0.747	98.597

Ethyl (*S*)-4-(3,5-dimethoxyphenyl)-2,2-difluoro-4-(4,4,5,5-tetramethyl-1,3,2-dioxaborolan-2-yl) butanoate (**2g**).

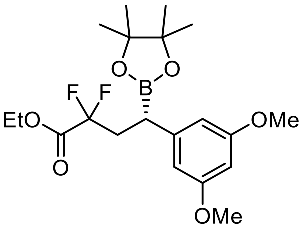

Colorless oil (56.3 mg, 68% yield, 91% ee); **^{1}H NMR** (400 MHz, $CDCl_3$) δ 6.39 (d, J = 2.3 Hz, 2H), 6.29 (t, J = 2.3 Hz, 1H), 4.19 (qq, J = 7.4, 3.6 Hz, 2H), 3.78 (s, 6H), 2.78 – 2.62 (m, 1H), 2.64 – 2.54 (m, 1H), 2.43 (dddd, J = 18.7, 16.6, 14.0, 5.2 Hz, 1H), 1.30 (t, J = 7.1 Hz, 3H), 1.22 (s, 6H), 1.20 (s, 6H); **^{13}C {^{1}H} NMR** (101 MHz, $CDCl_3$) δ 164.1 (t, J = 32.9 Hz), 160.7, 143.1, 115.9 (dd, J = 251.3, 249.6 Hz), 106.3, 98.2, 83.8, 62.6, 55.2, 37.1 (t, J = 23.0 Hz), 24.5, 24.4, 13.8; **^{19}F NMR** (377 MHz, $CDCl_3$) δ -103.00 (ddd, J = 259.4, 19.6, 10.3 Hz, 1F), -107.25 (ddd, J = 259.4, 20.7, 17.4 Hz, 1F), **^{11}B NMR** (128 MHz, $CDCl_3$) δ 33.37; **HRMS (EI)** m/z calcd for $C_{20}H_{29}BF_2O_6$ $[M+H]^+$: 431.2053; Found:431.2051.

Optical rotation: $[\alpha]_D^{25}$: -6.0 (c = 1.0, $CHCl_3$).

HPLC condition: Chiral column OD-H, n-Hexane/i-PrOH = 99:1, flow rate = 0.5 mL/min, wavelength = 220 nm, t_R = 13.1 min for the major isomer, t_R = 13.9 min for the minor isomer.

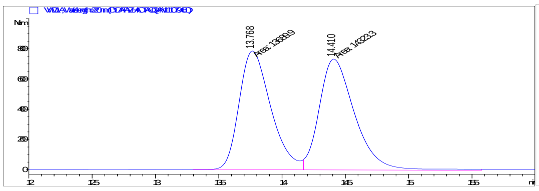

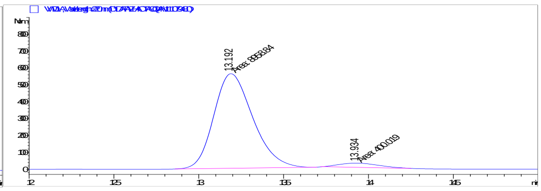

Peak#	Time	Area	Height	Width	Symmetry	Area%
1	13.768	13689.9	787	0.2899	0.672	48.869
2	14.41	14323.3	734.9	0.3248	0.626	51.131

Peak#	Time	Area	Height	Width	Symmetry	Area%
1	13.192	8958.8	561.4	0.266	0.763	95.726
2	13.934	400	25	0.267	0.737	4.274

Ethyl (*S*)-4-(benzo[d][1,3]dioxol-5-yl)-2,2-difluoro-4-(4,4,5,5-tetramethyl-1,3,2-dioxaborolan-2-yl) butanoate (2h).

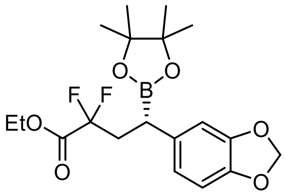

Colorless oil (48.5 mg, 61% yield, 97% ee); **^{1}H NMR** (400 MHz, $CDCl_3$) δ 6.76 – 6.69 (m, 2H), 6.67 (dd, *J* = 7.9, 1.7 Hz, 1H), 5.93 (q, *J* = 1.5 Hz, 2H), 4.20 (qd, *J* = 7.2, 3.1 Hz, 2H), 2.73 – 2.58 (m, 1H), 2.56 (dd, *J* = 8.8, 6.1 Hz, 1H), 2.40 (dddd, *J* = 17.4, 15.8, 13.3, 5.1 Hz, 1H), 1.31 (t, *J* = 7.2 Hz, 3H), 1.22 (s, 6H), 1.20 (s, 6H); **^{13}C {^{1}H} NMR** (101 MHz, $CDCl_3$) δ 164.1 (t, *J* = 32.9 Hz), 147.6, 145.7, 134.5, 121.2, 115.9 (dd, *J* = 251.5, 249.7 Hz), 108.7, 108.2, 100.7, 83.8, 62.6, 37.3 (t, *J* = 22.9 Hz), 24.5, 24.4, 13.8; **^{19}F NMR** (377 MHz, $CDCl_3$) δ -103.01 (ddd, *J* = 259.5, 19.1, 10.6 Hz, 1F), -107.13 (ddd, *J* = 259.6, 20.0, 17.6 Hz, 1F), **^{11}B NMR** (128 MHz, $CDCl_3$) δ 33.28; **HRMS (EI)** m/z calcd for $C_{19}H_{25}BF_2O_6$ $[M+Na]^+$: 421.1610; Found: 421.1614.

Optical rotation: $[\alpha]_D^{25}$: -2.7 (*c* = 1.0, $CHCl_3$).

HPLC condition: Chiral column OD-H, n-Hexane/i-PrOH = 99.5:0.5, flow rate = 0.5 mL/min, wavelength = 220 nm, t_R = 30.0 min for the major isomer, t_R = 26.0 min for the minor isomer.

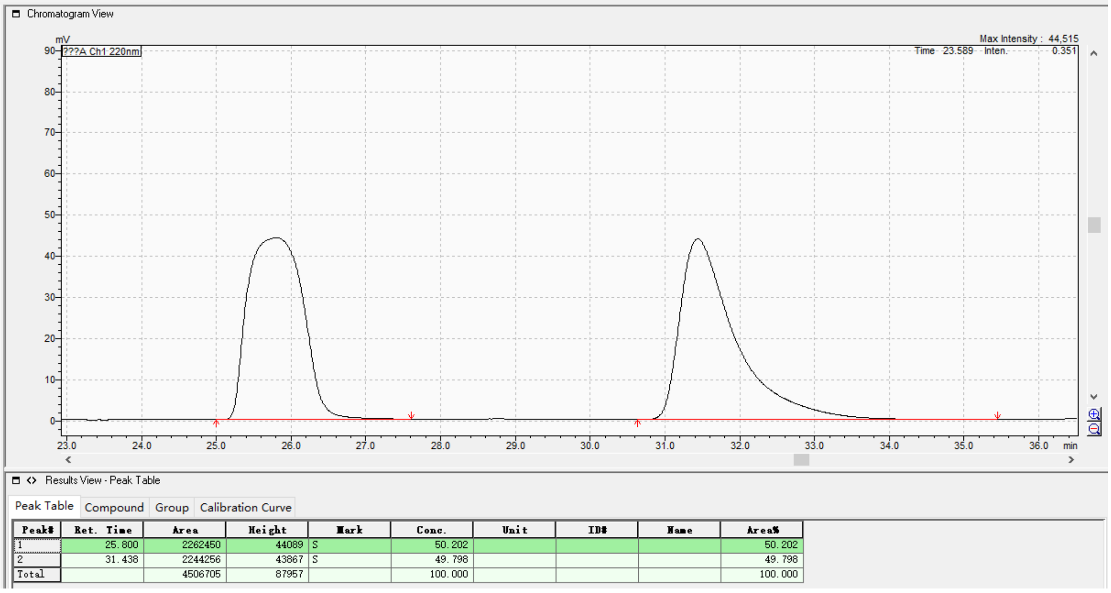

Peak#	Ret. Time	Area	Height	Mark	Conc.	Unit	ID#	Name	Area%
1	25.800	2262450	44089	S	50.202				50.202
2	31.438	2244256	43867	S	49.798				49.798
Total		4506705	87957		100.000				100.000

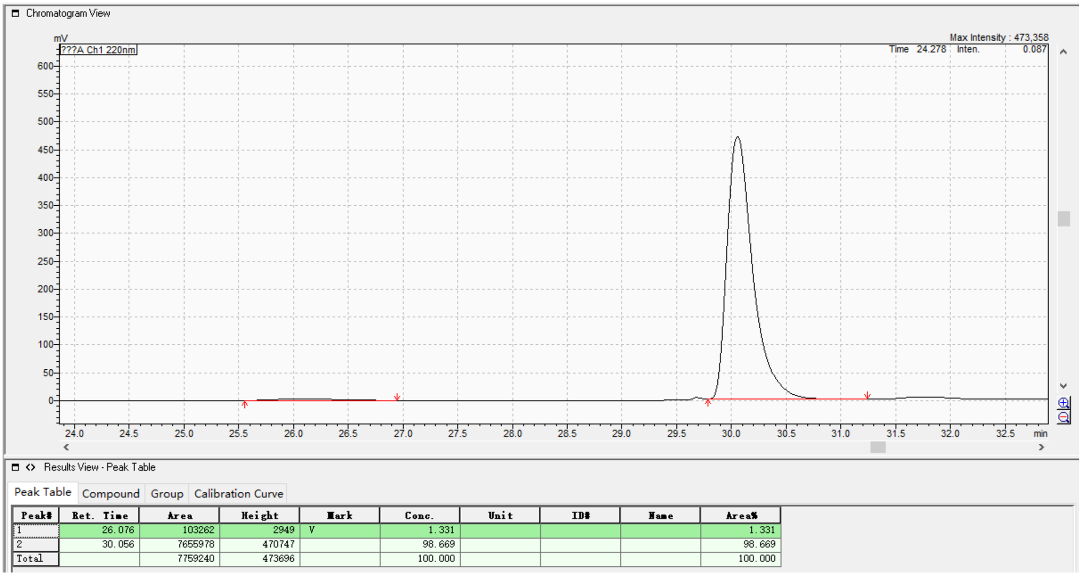

Peak#	Ret. Time	Area	Height	Mark	Conc.	Unit	ID#	Name	Area%
1	26.076	103262	2949	V	1.331				1.331
2	30.056	7655978	470747		98.669				98.669
Total		7759240	473696		100.000				100.000

Ethyl (*S*)-4-(4-acetoxyphenyl)-2,2-difluoro-4-(4,4,5,5-tetramethyl-1,3,2-dioxaborolan-2-yl)butanoate (2i).

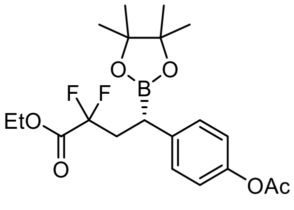

Colorless oil (70.0 mg, 85% yield, 98% ee); **^{1}H NMR** (400 MHz, $CDCl_3$) δ 7.23 (d, *J* = 8.6 Hz, 2H), 7.00 (d, *J* = 8.6 Hz, 2H), 4.25 – 4.05 (m, 2H), 2.78 – 2.58 (m, 2H), 2.54 – 2.34 (m, 1H), 2.29 (s, 3H), 1.29 (t, *J* = 7.1 Hz, 3H), 1.21 (s, 6H), 1.19 (s, 6H); **^{13}C {^{1}H} NMR** (101 MHz, $CDCl_3$) δ 169.3, 164.0 (t, *J* = 32.8 Hz), 148.9, 138.2, 129.1, 121.4, 115.9 (dd, *J* = 251.5, 249.6 Hz), 83.8, 62.7, 37.1 (t, *J* = 23.0 Hz),

24.5, 24.4, 21.0, 13.8; **^{19}F NMR** (377 MHz, $CDCl_3$) δ -102.64 (ddd, J = 259.2, 18.5, 9.6 Hz, 1F), -107.49 (dt, J = 259.3, 18.7 Hz, 1F), **^{11}B NMR** (128 MHz, $CDCl_3$) δ 33.37; **HRMS (EI)** m/z calcd for $C_{20}H_{27}BF_2O_6$ $[M+Na]^+$: 435.1766; Found: 435.1774.

Optical rotation: $[\alpha]_D^{25}$: 1.9 (c = 1.0, $CHCl_3$).

HPLC condition: Chiral column OD-H, n-Hexane/i-PrOH = 99:1, flow rate = 0.5 mL/min, wavelength = 220 nm, t_R = 16.1 min for the major isomer, t_R = 14.9 min for the minor isomer.

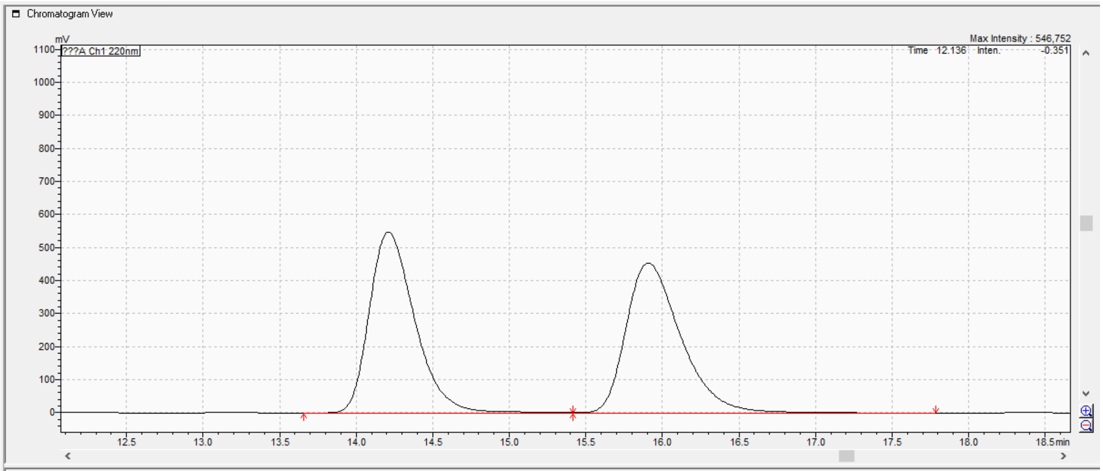

Results View - Peak Table

Peak Table | Compound | Group | Calibration Curve

Peak#	Ret. Time	Area	Height	Mark	Conc.	Unit	ID#	Name	Area%
1	14.211	11282320	547764		50.028				50.028
2	15.908	11269561	453717	V	49.972				49.972
Total		22551881	1001481		100.000				100.000

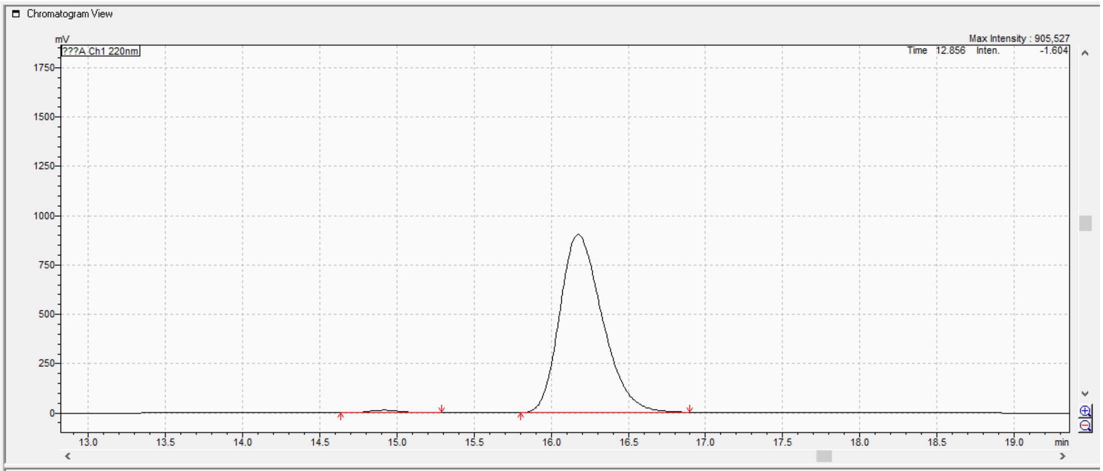

Results View - Peak Table

Peak Table | Compound | Group | Calibration Curve

Peak#	Ret. Time	Area	Height	Mark	Conc.	Unit	ID#	Name	Area%
1	14.915	186736	13333		1.065				1.065
2	16.175	17341058	903276	M	98.935				98.935
Total		17527794	916609		100.000				100.000

Ethyl (*S*)-4-([1,1'-biphenyl]-4-yl)-2,2-difluoro-4-(4,4,5,5-tetramethyl-1,3,2-dioxaborolan-2-yl)butanoate (2j).

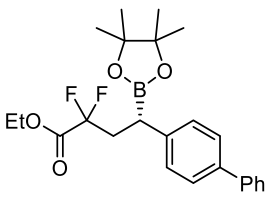

Colorless oil (49.0 mg, 57% yield, 94% ee); **^{1}H NMR** (400 MHz, $CDCl_3$) δ 7.60 (d, *J* = 7.8 Hz, 2H), 7.53 (d, *J* = 8.3 Hz, 2H), 7.45 (t, *J* = 7.6 Hz, 2H), 7.39 – 7.26 (m, 3H), 4.26 – 4.02 (m, 2H), 2.83 – 2.66 (m, 2H), 2.59 – 2.41 (m, 1H), 1.29 (t, *J* = 7.1 Hz, 3H), 1.24 (s, 6H), 1.21 (s, 6H); **^{13}C {^{1}H} NMR** (101 MHz, $CDCl_3$) δ 164.1 (t, *J* = 33.0 Hz), 140.8, 139.9, 138.7, 128.7, 127.1, 127.0, 126.8, 116.0 (dd, *J* = 251.4, 249.6 Hz), 83.8, 62.6, 37.0 (t, *J* = 23.0 Hz), 24.5, 24.4, 13.8; **^{19}F NMR** (377 MHz, $CDCl_3$) δ-101.81 – -103.44 (m, 1F), -106.59 – -108.04 (m, 1F), **^{11}B NMR** (128 MHz, $CDCl_3$) δ 34.39; **HRMS (EI)** m/z calcd for $C_{24}H_{29}BF_2O_4$ $[M+Na]^+$: 453.2025; Found: 453.2029.

Optical rotation: $[\alpha]_D^{25}$: -4.5 (*c* = 1.0, $CHCl_3$).

HPLC condition: Chiral column OD-H, n-Hexane/i-PrOH = 99.5:0.5, flow rate = 0.5 mL/min, wavelength = 220 nm, t_R = 13.4 min for the major isomer, t_R = 12.0 min for the minor isomer.

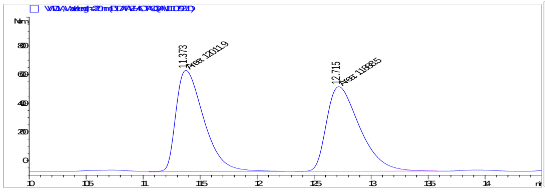

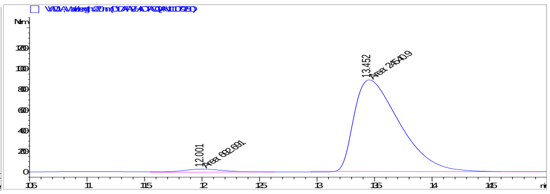

Peak#	Time	Area	Height	Width	Symmetry	Area%
1	11.373	12011.9	706.8	0.2832	0.497	50.258
2	12.715	11888.5	590	0.3359	0.521	49.742

Peak#	Time	Area	Height	Width	Symmetry	Area%
1	12.001	692.7	29	0.3982	1.008	2.745
2	13.452	24540.9	890.7	0.4592	0.504	97.255

Ethyl (*S*)-2,2-difluoro-4-(4-fluorophenyl)-4-(4,4,5,5-tetramethyl-1,3,2-dioxaborolan-2-yl)butanoate (2k).

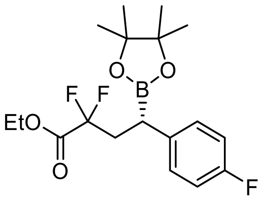

Colorless oil (60.2 mg, 81% yield, 99% ee); **^{1}H NMR** (400 MHz, $CDCl_3$) δ 7.24 – 7.14 (m, 2H), 7.03 – 6.91 (m, 2H), 4.17 (qd, *J* = 7.2, 4.7 Hz, 2H), 2.77 – 2.59 (m, 2H), 2.52 – 2.33 (m, 1H), 1.30 (t, *J* = 7.2 Hz, 3H), 1.21 (s, 6H), 1.19 (s, 6H); **^{13}C {^{1}H} NMR** (101 MHz, $CDCl_3$) δ 164.0 (t, *J* = 32.8 Hz), 161.2 (d, *J* = 243.8 Hz), 136.4

(d, J = 3.3 Hz), 129.6 (d, J = 7.8 Hz), 115.9 (dd, J = 251.5, 249.8 Hz), 115.2 (d, J = 21.2 Hz), 83.8, 62.6, 37.1 (t, J = 23.1 Hz), 24.4, 24.4, 13.8; **^{19}F NMR** (377 MHz, $CDCl_3$) δ -102.28 – -103.72 (m, 1F), -106.60 – -107.76 (m, 1F), -117.50 (tt, J = 8.9, 5.3 Hz, 1F), **^{11}B NMR** (128 MHz, $CDCl_3$) δ 34.13; **HRMS (EI)** m/z calcd for $C_{18}H_{24}BF_3O_4$ $[M+H]^+$: 373.1798; Found: 373.1789.

Optical rotation: $[\alpha]_D^{25}$: -24.1 (c = 1.00, $CHCl_3$).

HPLC condition: Chiral column OD-H, n-Hexane/i-PrOH = 99.5:0.5, flow rate = 0.5 mL/min, wavelength = 220 nm, t_R = 16.4 min for the major isomer, t_R = 15.4 min for the minor isomer.

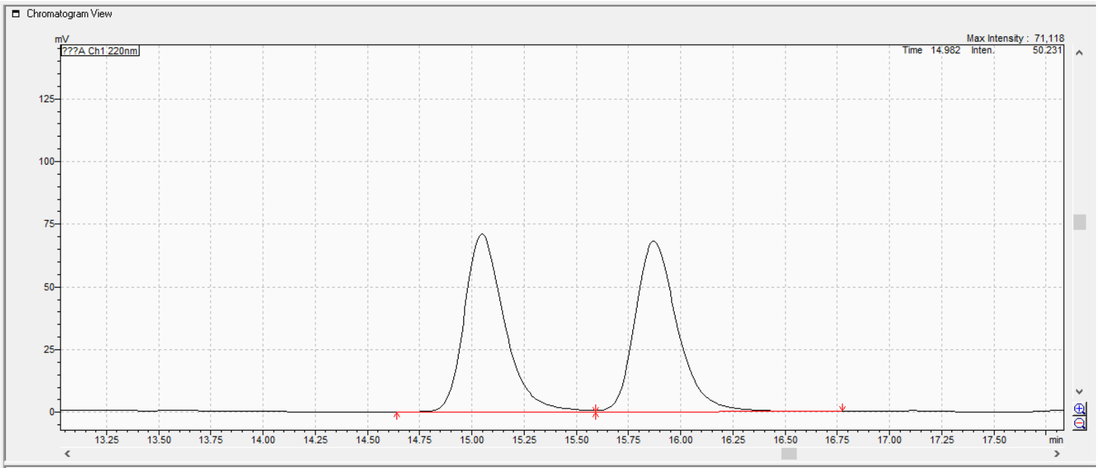

Results View - Peak Table

Peak Table | Compound | Group | Calibration Curve

Peak#	Ret. Time	Area	Height	Mark	Conc.	Unit	ID#	Name	Area%
1	15.048	942049	70858		49.746				49.746
2	15.871	951658	67937	SV	50.254				50.254
Total		1893707	138795		100.000				100.000

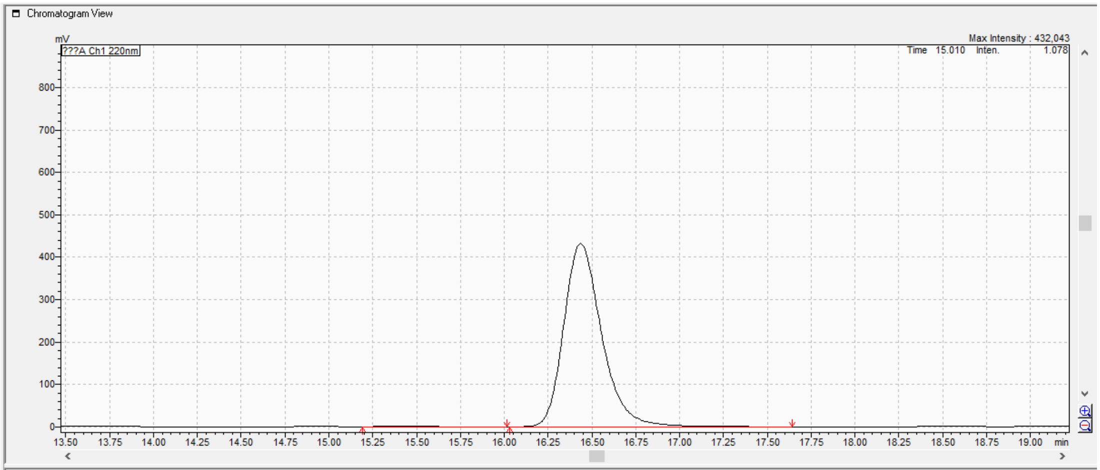

Results View - Peak Table

Peak Table | Compound | Group | Calibration Curve

Peak#	Ret. Time	Area	Height	Mark	Conc.	Unit	ID#	Name	Area%
1	15.416	26362	1962	M	0.406				0.406
2	16.435	6465064	431406		99.594				99.594
Total		6491427	433368		100.000				100.000

Ethyl (*S*)-2,2-difluoro-4-(3-fluorophenyl)-4-(4,4,5,5-tetramethyl-1,3,2-dioxaborolan-2-yl)butanoate (**2l**).

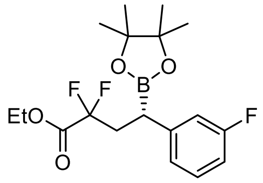

Colorless oil (55.0 mg, 74% yield, 97% ee); **^{1}H NMR** (400 MHz, $CDCl_3$) δ 7.15 (td, *J* = 8.0, 6.1 Hz, 1H), 6.91 (dt, *J* = 7.8, 1.3 Hz, 1H), 6.86 (dt, *J* = 10.2, 2.2 Hz, 1H), 6.79 (tt, *J* = 8.7, 1.8 Hz, 1H), 4.09 (qq, *J* = 7.4, 3.6 Hz, 2H), 2.68 – 2.52 (m, 2H), 2.47 – 2.26 (m, 1H), 1.22 (t, *J* = 7.1 Hz, 3H), 1.12 (s, 6H), 1.10 (s, 6H); **^{13}C {^{1}H} NMR** (101 MHz, $CDCl_3$) δ 164.0 (t, *J* = 32.9 Hz), 162.8 (d, *J* = 245.4 Hz), 143.4 (d, *J* = 7.4 Hz), 129.8 (d, *J* = 8.4 Hz), 123.9 (d, *J* = 2.7 Hz), 115.8 (dd, *J* = 251.9, 249.6 Hz), 115.0 (d, *J* = 21.6 Hz), 112.8 (d, *J* = 21.1 Hz), 84.0, 62.7, 36.7 (t, *J* = 23.0 Hz), 24.4, 24.4, 13.8; **^{19}F NMR** (377 MHz, $CDCl_3$) δ -103.15 (ddd, *J* = 259.5, 19.6, 10.1 Hz, 1F), -106.41 – -107.81 (m, 1F), -113.36 (td, *J* = 10.3, 6.0 Hz, 1F), **^{11}B NMR** (128 MHz, $CDCl_3$) δ 33.47; HRMS m/z (EI) calcd for $C_{18}H_{24}BF_3O_4$: 395.1617; Found: 395.1618.

Optical rotation: $[\alpha]_D^{25}$: 4.8 (*c* = 0.50, $CHCl_3$).

HPLC condition: Chiral column OD-H, n-Hexane/i-PrOH = 99.5:0.5, flow rate = 0.4 mL/min, wavelength = 210 nm, t_R = 11.6 min for the major isomer, t_R = 11.2 min for the minor isomer.

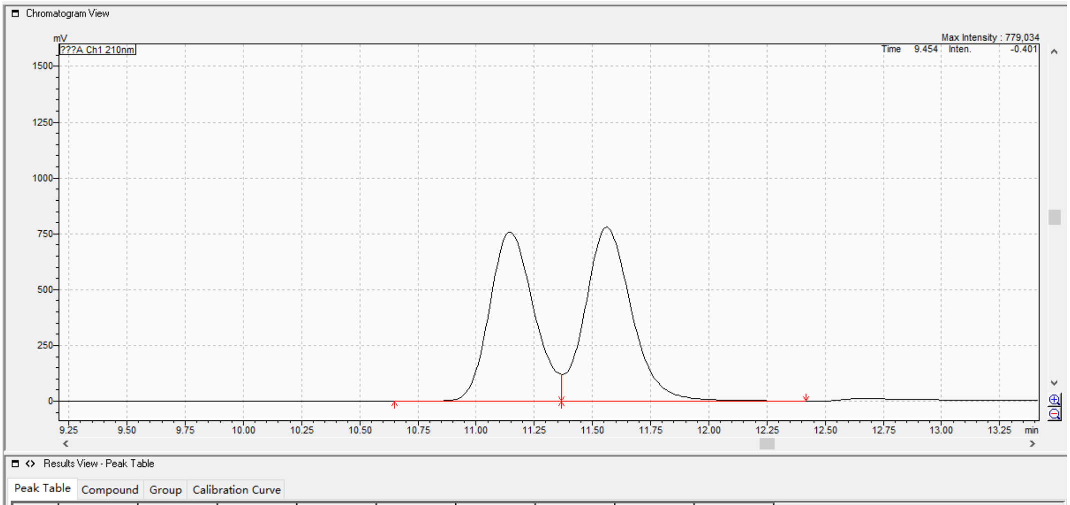

Peak#	Ret. Time	Area	Height	Mark	Conc.	Unit	ID#	Name	Area%
1	11.145	9996926	757965		48.152				48.152
2	11.561	10764320	778721	V	51.848				51.848
Total		20761246	1536687		100.000				100.000

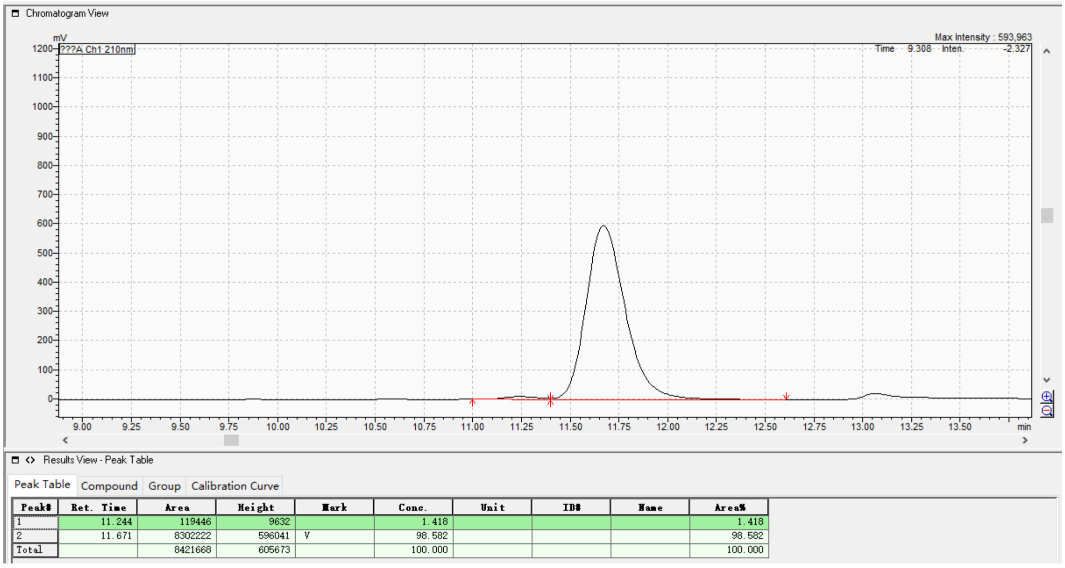

Ethyl (*S*)-4-(3-chlorophenyl)-2,2-difluoro-4-(4,4,5,5-tetramethyl-1,3,2-dioxaborolan-2-yl)butanoate (2m).

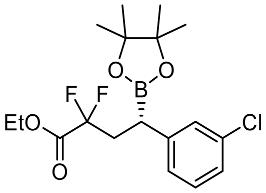

Colorless oil (46.6 mg, 61% yield, 97% ee); **^{1}H NMR** (400 MHz, $CDCl_3$) δ 7.13 (t, *J* = 1.9 Hz, 1H), 7.11 (d, *J* = 7.6 Hz, 1H), 7.07 (dt, *J* = 8.0, 1.6 Hz, 1H), 7.02 (dt, *J* = 7.5, 1.6 Hz, 1H), 4.08 (qq, *J* = 7.5, 3.6 Hz, 2H), 2.69 – 2.49 (m, 2H), 2.44 – 2.24 (m, 1H), 1.22 (t, *J* = 7.2 Hz, 3H), 1.12 (s, 6H), 1.10 (s, 6H); **^{13}C {^{1}H} NMR** (101 MHz, $CDCl_3$) δ 164.0 (t, *J* = 32.7 Hz), 142.9, 134.2, 129.7, 128.3, 126.5, 126.1, 115.8 (dd, *J* = 251.8, 249.7 Hz), 84.0, 62.7, 36.7 (t, *J* = 23.1 Hz), 24.4, 24.4, 13.8; **^{19}F NMR** (377 MHz, $CDCl_3$) δ -102.37 – -103.48 (m, 1F), -107.16 (ddd, *J* = 259.6, 19.6, 16.6 Hz, 1F), **^{11}B NMR** (128 MHz, $CDCl_3$) δ 32.71; **HRMS (EI)** m/z calcd for $C_{18}H_{24}BClF_2O_4$ $[M+Na]^+$: 411.1322; Found: 411.1319 .

Optical rotation: $[\alpha]_D^{25}$: 12.7 (*c* = 1.00, $CHCl_3$).

HPLC condition: Chiral column OD-H, n-Hexane/i-PrOH = 99.5:0.5, flow rate = 0.5 mL/min, wavelength = 220 nm, t_R = 11.1 min for the major isomer, t_R = 10.4 min for the minor isomer.

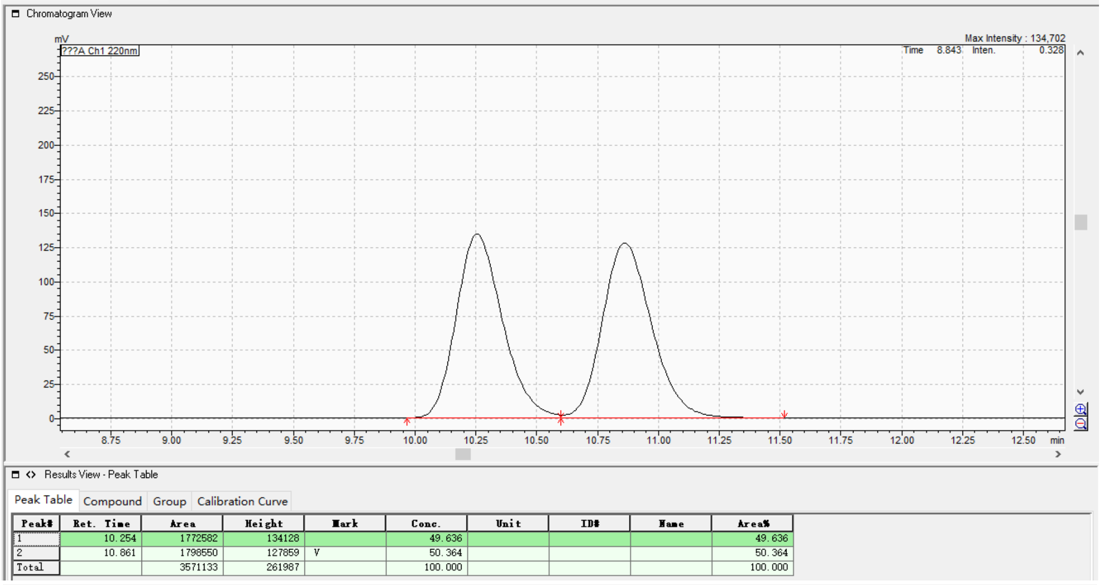

Peak#	Ret. Time	Area	Height	Mark	Conc.	Unit	ID#	Name	Area%
1	10.254	1772582	134128		49.636				49.636
2	10.861	1798550	127859	V	50.364				50.364
Total		3571133	261987		100.000				100.000

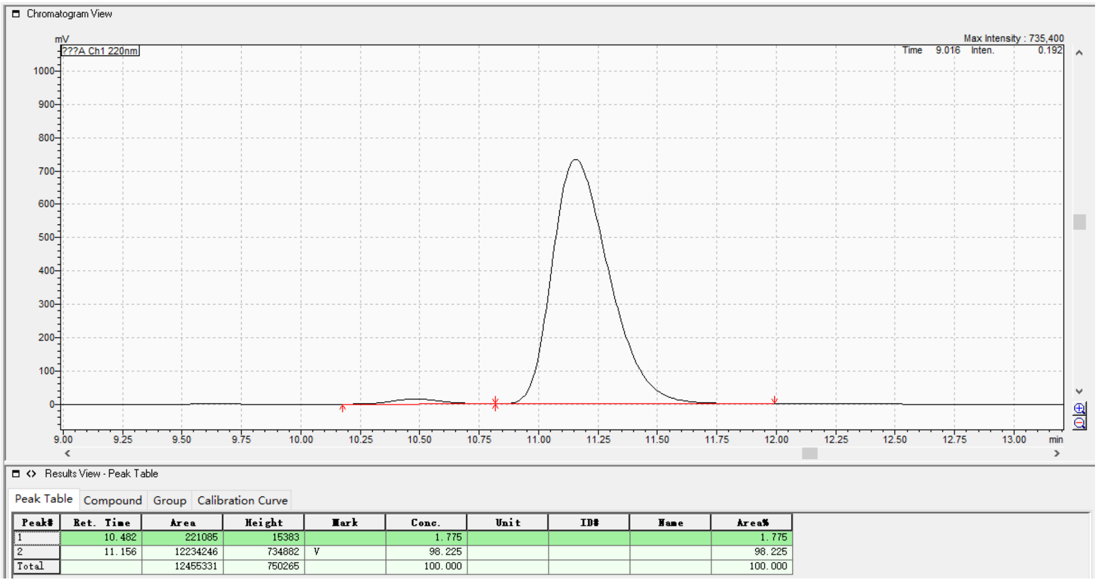

Peak#	Ret. Time	Area	Height	Mark	Conc.	Unit	ID#	Name	Area%
1	10.482	221085	15383		1.775				1.775
2	11.156	12234246	734882	V	98.225				98.225
Total		12455331	750265		100.000				100.000

Ethyl (*S*)-2,2-difluoro-4-(4-(methylthio)phenyl)-4-(4,4,5,5-tetramethyl-1,3,2-dioxaborolan-2-yl)butanoate (**2n**).

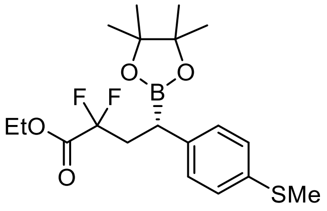

Colorless oil (37.6 mg, 47% yield, 94% ee); **^{1}H NMR** (400 MHz, $CDCl_3$) δ 7.19 (d, *J* = 8.5 Hz, 2H), 7.15 (d, *J* = 8.5 Hz, 2H), 4.15 (qd, *J* = 7.1, 5.5 Hz, 2H), 2.77 – 2.56 (m, 2H), 2.47 (s, 3H), 2.52 – 2.31 (m, 1H), 1.29 (t, *J* = 7.1 Hz, 3H), 1.21 (s, 6H), 1.19 (s, 6H); **^{13}C {^{1}H} NMR** (101 MHz, $CDCl_3$) δ 164.0 (t, *J* = 32.8 Hz), 137.8, 135.5, 128.8, 127.1, 115.9 (dd, *J* = 251.5, 249.6 Hz), 83.8, 62.6, 37.0 (t, *J* = 23.0 Hz), 24.5, 24.4, 16.1, 13.8; **^{19}F NMR** (377 MHz, $CDCl_3$) δ -102.89 (ddd, *J* =

259.3, 19.2, 10.6 Hz, 1F), -107.25 (ddd, J = 259.1, 19.8, 17.4 Hz, 1F), **^{11}B NMR** (128 MHz, $CDCl_3$) δ 33.35; **HRMS (EI)** m/z calcd for $C_{19}H_{27}BF_2O_4S$ $[M+Na]^+$: 423.1589; Found: 423.1588.

Optical rotation: $[\alpha]_D^{25}$: 6.6 (c = 1.0, $CHCl_3$).

HPLC condition: Chiral column OD-H, n-Hexane/i-PrOH = 99.5:0.5, flow rate = 0.4 mL/min, wavelength = 210 nm, t_R = 17.9 min for the major isomer, t_R = 13.5 min for the minor isomer.

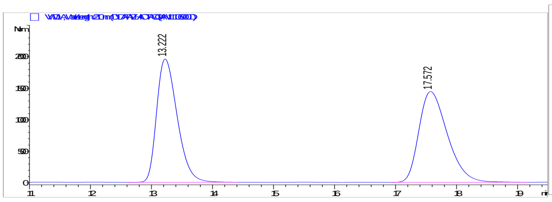

Peak	Time	Start	End	Area	Height	Area%
1	13.222	45811.8	1956	0.3635	0.666	49.503
2	17.572	46732.6	1440.1	0.5053	0.661	50.497

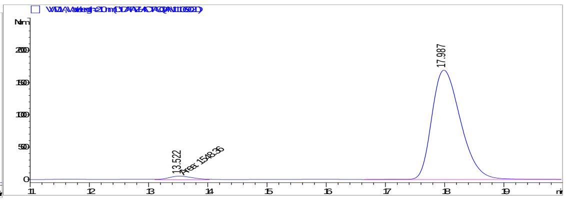

Peak	Time	Start	End	Area	Height	Area%
1	13.522	1548.4	54.8	0.4706	0.779	2.634
2	17.987	57236.6	1693	0.5342	0.689	97.366

Ethyl (*S*)-2,2-difluoro-4-(4,4,5,5-tetramethyl-1,3,2-dioxaborolan-2-yl)-4-(4-(trimethylsilyl)phenyl)butanoate (**2o**).

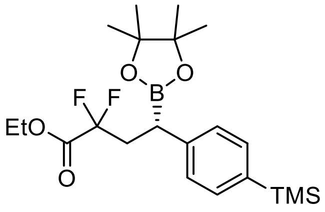

Colorless oil (62.2 mg, 73% yield, 98% ee); **^{1}H NMR** (400 MHz, $CDCl_3$) δ 7.43 (d, J = 7.9 Hz, 2H), 7.21 (d, J = 7.9 Hz, 2H), 4.10 (qq, J = 10.7, 7.1 Hz, 2H), 2.80 – 2.64 (m, 1H), 2.66 – 2.59 (m, 1H), 2.56 – 2.34 (m, 1H), 1.26 (t, J = 7.1 Hz, 3H), 1.22 (s, 6H), 1.20 (s, 6H), 0.26 (s, 9H); **^{13}C {^{1}H} NMR** (101 MHz, $CDCl_3$) δ 164.1 (t, J = 33.0 Hz), 141.3, 137.4, 133.5, 127.7, 116.0 (dd, J = 251.3, 249.4 Hz), 83.8, 62.5, 37.1 (t, J = 23.0 Hz), 24.5, 24.4, 13.8, -1.1; **^{19}F NMR** (377 MHz, $CDCl_3$) δ-102.47 (ddd, J = 259.1, 18.4, 10.4 Hz, 1F), -107.48 (dt, J = 259.0, 19.1 Hz, 1F), **^{11}B NMR** (128 MHz, $CDCl_3$) δ 33.62; **HRMS (EI)** m/z calcd for $C_{21}H_{33}BF_2O_4Si$ $[M+Na]^+$: 449.2107; Found: 449.2103.

Optical rotation: $[\alpha]_D^{25}$: 5.6 (c = 1.2, $CHCl_3$).

HPLC condition: Chiral column OD-H, n-Hexane/i-PrOH = 99.5:0.5, flow rate = 0.5 mL/min, wavelength = 220 nm, t_R = 14.1 min for the major isomer, t_R = 13.7 min for

the minor isomer.

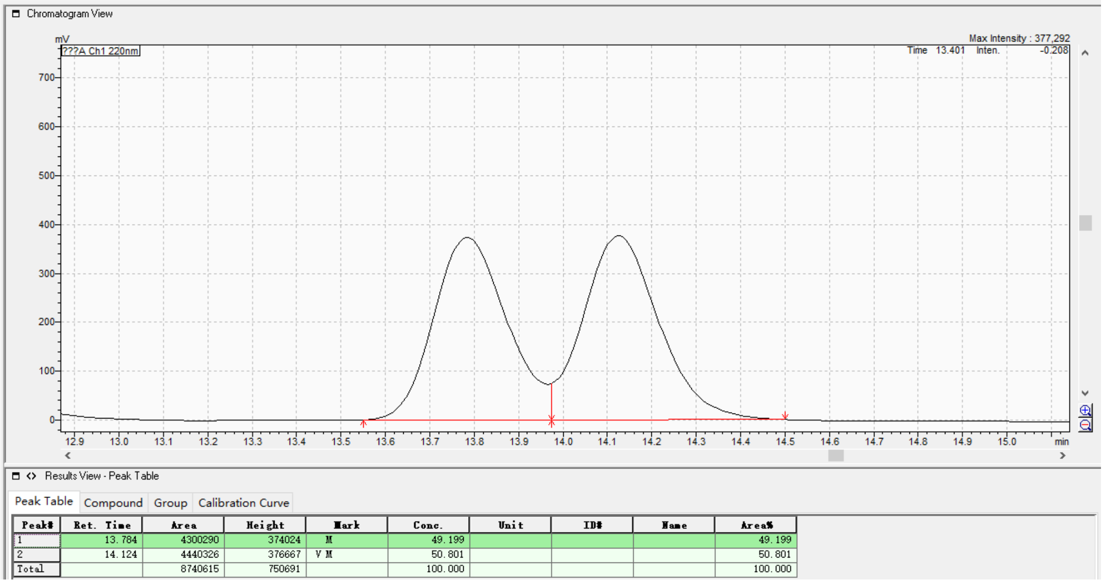

Peak#	Ret. Time	Area	Height	Mark	Conc.	Unit	ID#	Name	Area%
1	13.784	4300290	374024	M	49.199				49.199
2	14.124	4440326	376667	V M	50.801				50.801
Total		8740615	750691		100.000				100.000

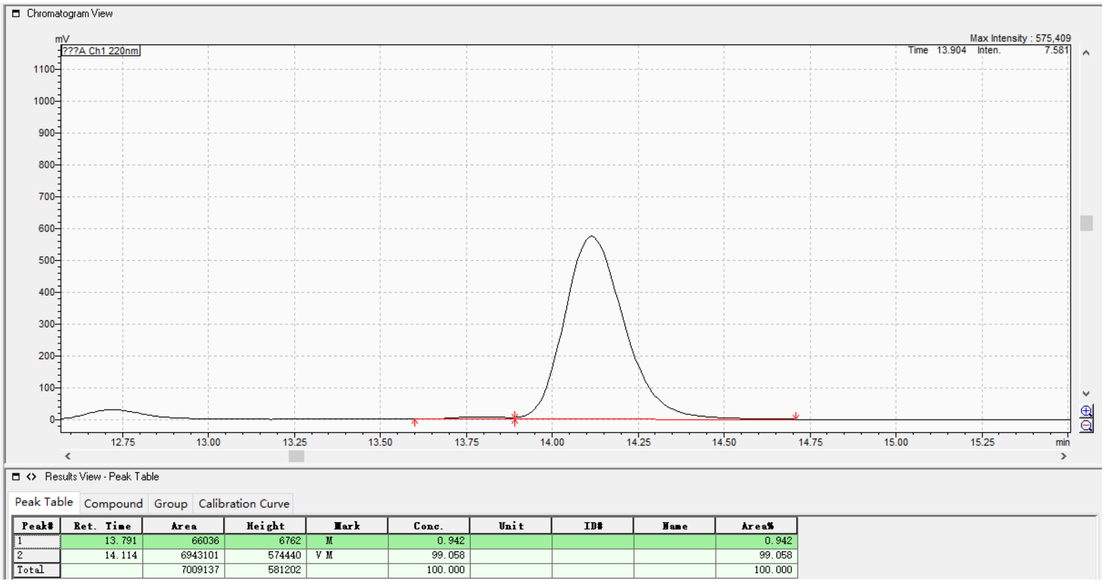

Peak#	Ret. Time	Area	Height	Mark	Conc.	Unit	ID#	Name	Area%
1	13.791	66036	6762	M	0.942				0.942
2	14.114	6943101	574440	V M	99.058				99.058
Total		7009137	581202		100.000				100.000

Methyl (*S*)-3-(4-ethoxy-3,3-difluoro-4-oxo-1-(4,4,5,5-tetramethyl-1,3,2-dioxaborolan-2-yl)butyl)benzoate (**2p**).

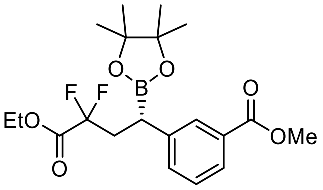

Colorless oil (61.8 mg, 75% yield, 96% ee); **^{1}H NMR** (400 MHz, $CDCl_3$) δ 7.94 – 7.89 (m, 1H), 7.86 (dt, *J* = 7.6, 1.5 Hz, 1H), 7.43 (dt, *J* = 7.8, 1.6 Hz, 1H), 7.36 (t, *J* = 7.7 Hz, 1H), 4.16 (qd, *J* = 7.2, 4.9 Hz, 2H), 3.92 (s, 3H), 2.83 – 2.64 (m, 2H), 2.46 (dddd, *J* = 23.0, 18.4, 13.2, 7.8 Hz, 1H), 1.29 (t, *J* = 7.1 Hz, 3H), 1.20 (s, 6H), 1.18 (s, 6H); **^{13}C {^{1}H} NMR** (101 MHz, $CDCl_3$) δ 167.0, 164.0 (t, *J* =

32.8 Hz), 141.3, 132.9, 130.4, 129.2, 128.5, 127.2, 115.9 (dd, J = 251.7, 250.1 Hz), 83.9, 62.7, 52.0, 36.8 (t, J = 23.0 Hz), 24.4, 24.4, 13.8; **^{19}F NMR** (377 MHz, $CDCl_3$) δ -102.34 – -104.00 (m, 1F), -106.21 – -107.46 (m, 1F), **^{11}B NMR** (128 MHz, $CDCl_3$) δ 32.73; **HRMS (EI)** m/z calcd for $C_{20}H_{27}BF_2O_6$ $[M+Na]^+$: 435.1766; Found: 435.1772.

Optical rotation: $[\alpha]_D^{25}$: 4.0 (c = 1.0, $CHCl_3$).

HPLC condition: Chiral column OD-H, n-Hexane/i-PrOH = 99:1, flow rate = 0.4 mL/min, wavelength = 210 nm, t_R = 23.2 min for the major isomer, t_R = 20.3 min for the minor isomer.

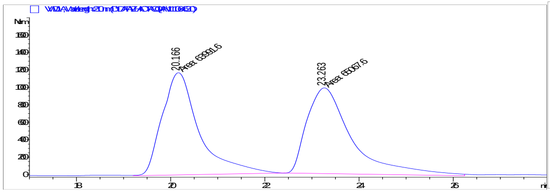

Peak#	Time	Area	Height	Width	Symmetry	Area%
1	20.166	63991.6	1172.5	0.9096	0.748	49.583
2	23.263	65067.6	983.2	1.1029	0.602	50.417

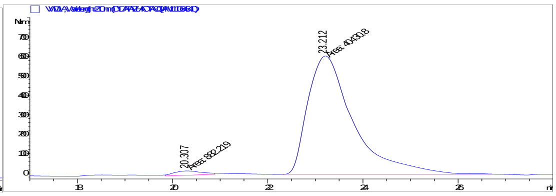

Peak#	Time	Area	Height	Width	Symmetry	Area%
1	20.307	882.2	21.9	0.6702	0.922	2.135
2	23.212	40430.8	608.4	1.1076	0.602	97.865

Ethyl (*S*)-2,2-difluoro-4-(6-methoxynaphthalen-2-yl)-4-(4,4,5,5-tetramethyl-1,3,2-dioxaborolan-2-yl)butanoate (**2q**).

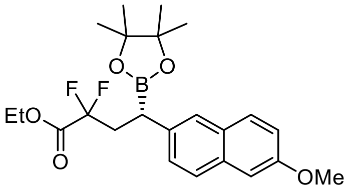

Colorless oil (44.3 mg, 51% yield, 95% ee); **^{1}H NMR** (400 MHz, $CDCl_3$) δ 7.68 (dd, J = 8.6, 3.7 Hz, 2H), 7.59 (d, J = 1.8 Hz, 1H), 7.34 (dd, J = 8.5, 1.8 Hz, 1H), 7.13 (dd, J = 8.8, 2.6 Hz, 1H), 7.11 (d, J = 2.5 Hz, 1H), 4.13 – 3.95 (m, 2H), 3.93 (s, 3H), 2.88 – 2.69 (m, 2H), 2.57 (ddt, J = 18.4, 13.2, 6.4 Hz, 1H), 1.22 (t, J = 7.0 Hz, 3H), 1.21 (s, 6H), 1.18 (s, 6H); **^{13}C {^{1}H} NMR** (101 MHz, $CDCl_3$) δ 164.1 (t, J = 32.9 Hz), 157.2, 135.8, 132.9, 129.1, 128.9, 127.4, 126.9, 126.3, 118.6, 116.1 (dd, J = 251.6, 249.4 Hz), 105.6, 83.8, 62.5, 55.2, 37.0 (t, J = 23.0 Hz), 24.5, 24.4, 13.7; **^{19}F NMR** (377 MHz, $CDCl_3$) δ -102.51 (d, J = 258.9 Hz, 1F), -107.43 (d, J = 259.0 Hz, 1F), **^{11}B NMR** (128 MHz, $CDCl_3$) δ 33.78; **HRMS (EI)** m/z calcd for $C_{23}H_{29}BF_2O_5$ $[M+K]^+$: 473.1713; Found: 473.1718.

Optical rotation: $[\alpha]_D^{25}$: -6.8 (c = 1.0, $CHCl_3$).

HPLC condition: Chiral column OD-H, n-Hexane/i-PrOH = 99:1, flow rate = 1.0 mL/min, wavelength = 210 nm, t_R = 9.3 min for the major isomer, t_R = 7.7 min for the minor isomer.

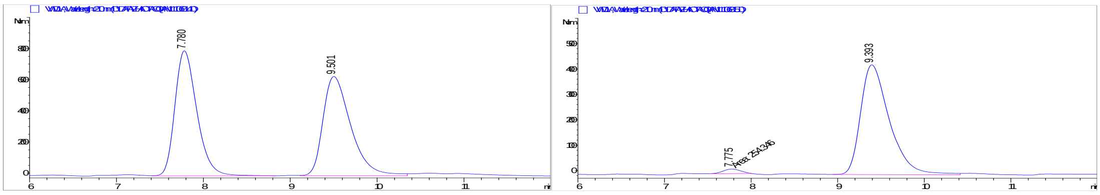

Peak#	Time	Area	Height	Width	Symmetry	Area%
1	7.78	14141.1	803.1	0.271	0.71	49.883
2	9.501	14207.6	637.7	0.3415	0.598	50.117

Peak#	Time	Area	Height	Width	Symmetry	Area%
1	7.775	254.3	17.9	0.2365	0.973	2.485
2	9.393	9980.9	432.1	0.3496	0.593	97.515

Ethyl (*S*)-4-(9-ethyl-9*H*-carbazol-3-yl)-2,2-difluoro-4-(4,4,5,5-tetramethyl-1,3,2-dioxaborolan-2-yl)butanoate (**2r**).

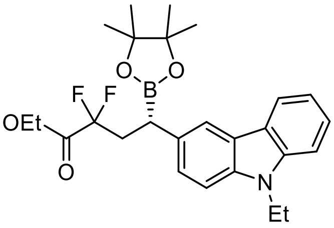

Colorless oil (70.6 mg, 75% yield, 99% ee); **^{1}H NMR** (400 MHz, $CDCl_3$) δ 8.10 (d, J = 7.7 Hz, 1H), 7.97 (s, 1H), 7.47 (ddd, J = 8.1, 7.0, 1.2 Hz, 1H), 7.40 (d, J = 8.1 Hz, 1H), 7.38 – 7.31 (m, 2H), 7.24 (t, J = 7.4 Hz, 1H), 4.36 (q, J = 7.2 Hz, 2H), 4.16 – 3.97 (m, 2H), 2.92 – 2.73 (m, 2H), 2.60 (tdd, J = 18.8, 13.5, 7.4 Hz, 1H), 1.44 (t, J = 7.2 Hz, 3H), 1.26 – 1.18 (m, 15H); **^{13}C {^{1}H} NMR** (101 MHz, $CDCl_3$) δ 164.2 (t, J = 33.0 Hz), 140.1, 138.5, 130.9, 126.3, 125.5, 123.1, 122.7, 120.3, 119.8, 118.6, 116.2 (dd, J = 251.2, 249.3 Hz), 108.4, 108.3, 83.7, 62.5, 37.9 (t, J = 22.8 Hz), 37.5, 24.5, 24.4, 13.8, 13.7; **^{19}F NMR** (377 MHz, $CDCl_3$) δ -102.06 – -103.00 (m, 1F), -106.52 – -108.27 (m, 1F), **^{11}B NMR** (128 MHz, $CDCl_3$) δ 33.56; **HRMS (EI)** m/z calcd for $C_{26}H_{32}BF_2NO_4$ $[M+Na]^+$: 494.2290; Found:494.2280.

Optical rotation: $[\alpha]_D^{25}$: 0.9 (c = 0.3, $CHCl_3$).

HPLC condition: Chiral column OD-H, n-Hexane/i-PrOH = 98:2, flow rate = 1.0 mL/min, wavelength = 210 nm, t_R = 7.6 min for the major isomer, t_R = 7.0 min for the minor isomer.

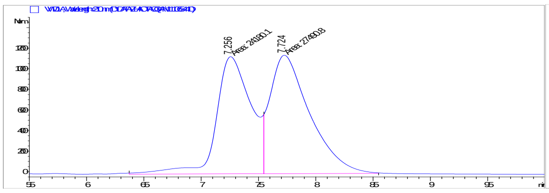

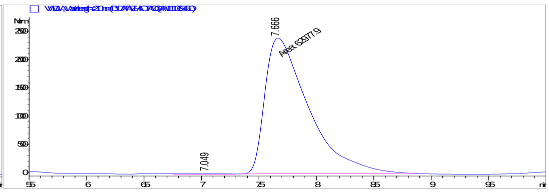

Peak#	Time	Area	Height	Width	Symmetry	Area%
1	7.256	24180.1	1137.6	0.3543	0	46.796
2	7.724	27490.8	1150	0.3984	0.526	53.204

Peak#	Time	Area	Height	Width	Symmetry	Area%
1	7.049	272.4	12.4	0.3019	1.433	0.431
2	7.666	62977.9	2400.6	0.4372	0.398	99.569

Ethyl (*S*)-2,2-difluoro-4-(6-methoxypyridin-3-yl)-4-(4,4,5,5-tetramethyl-1,3,2-dioxaborolan-2-yl)butanoate (2s).

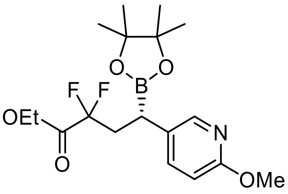

Colorless oil (51.6 mg, 67% yield, 89% ee); **^{1}H NMR** (400 MHz, $CDCl_3$) δ 8.01 (d, *J* = 2.5 Hz, 1H), 7.47 (dd, *J* = 8.5, 2.5 Hz, 1H), 6.68 (d, *J* = 8.5 Hz, 1H), 4.19 (qd, *J* = 7.2, 1.7 Hz, 2H), 3.92 (s, 3H), 2.77 – 2.59 (m, 1H), 2.61 – 2.54 (m, 1H), 2.49 – 2.28 (m, 1H), 1.31 (t, *J* = 7.1 Hz, 3H), 1.22 (s, 6H), 1.20 (s, 6H); **^{13}C {^{1}H} NMR** (101 MHz, $CDCl_3$) δ 164.0 (t, *J* = 32.7 Hz), 162.6, 146.0, 138.8, 128.9, 115.8 (dd, *J* = 251.7, 250.2 Hz), 110.5, 84.0, 62.7, 53.3, 37.0 (t, *J* = 23.1 Hz), 24.5, 24.5, 13.8; **^{19}F NMR** (377 MHz, $CDCl_3$) δ -103.23 (ddd, *J* = 260.4, 19.6, 10.7 Hz, 1F), -106.93 (ddd, *J* = 260.3, 19.8, 16.5 Hz, 1F), **^{11}B NMR** (128 MHz, $CDCl_3$) δ 33.29; **HRMS (EI)** m/z calcd for $C_{18}H_{26}BF_2NO_5$ $[M+H]^+$: 386.1950; Found: 386.1956.

Optical rotation: $[\alpha]_D^{25}$: 7.2 (*c* = 1.0, $CHCl_3$).

HPLC condition: Chiral column OD-H, n-Hexane/i-PrOH = 99:1, flow rate = 1.0 mL/min, wavelength = 210 nm, t_R = 6.2 min for the major isomer, t_R = 5.7 min for the minor isomer.

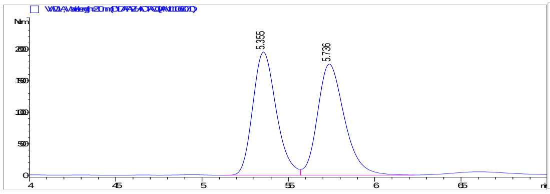

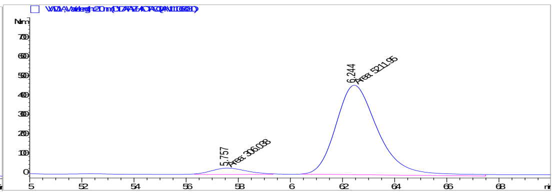

Peak#	Time	Area	Height	Width	Symmetry	Area%
1	5.355	18463.1	1953.8	0.1466	0.794	49.111
2	5.736	19131.3	1763.3	0.1659	0.732	50.889

Peak#	Time	Area	Height	Width	Symmetry	Area%
1	5.757	306	32.7	0.156	0.792	5.546
2	6.244	5211.9	463.1	0.1876	0.738	94.454

Tert-butyl (*S*)-2,2-difluoro-4-phenyl-4-(4,4,5,5-tetramethyl-1,3,2-dioxaborolan-2-yl)butanoate (**4a**).

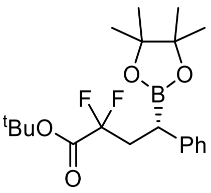

Colorless oil (67.2 mg, 88% yield, 95% ee); **^{1}H NMR** (400 MHz, $CDCl_3$) δ 7.33 – 7.21 (m, 4H), 7.18 (t, *J* = 7.0 Hz, 1H), 2.78 – 2.60 (m, 2H), 2.48 – 2.27 (m, 1H), 1.49 (s, 9H), 1.21 (s, 6H), 1.19 (s, 6H); **^{13}C {^{1}H} NMR** (101 MHz, $CDCl_3$) δ 163.1 (t, *J* = 32.3 Hz), 141.2, 128.5, 128.1, 125.8, 115.9 (dd, *J* = 252.0, 250.2 Hz), 84.1, 83.7, 37.0 (t, *J* = 22.9 Hz), 27.6, 24.4; **^{19}F NMR** (377 MHz, $CDCl_3$) δ -102.88 – -104.09 (m, 1F), -107.18 (ddd, *J* = 256.1, 21.2, 15.8 Hz, 1F), **^{11}B NMR** (128 MHz, $CDCl_3$) δ 32.65; **HRMS (EI)** m/z calcd for $C_{20}H_{29}BF_2O_4$ $[M+Na]^+$: 405.2025; Found: 405.2013.

Optical rotation: $[\alpha]_D^{25}$: 7.1 (*c* = 1.0, $CHCl_3$).

HPLC condition: Chiral column OD-H, n-Hexane/i-PrOH = 99:1, flow rate = 0.4 mL/min, wavelength = 210 nm, t_R = 12.2 min for the major isomer, t_R = 9.9 min for the minor isomer.

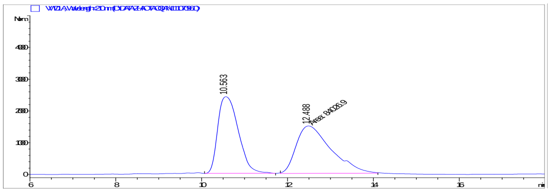

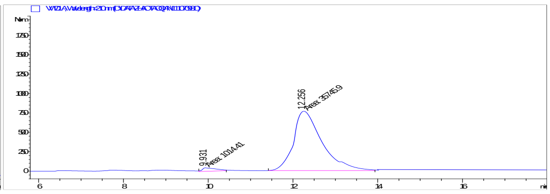

Peak#	Time	Area	Height	Width	Symmetry	Area%
1	10.563	80191.2	2414.4	0.5229	0.622	48.832
2	12.488	84026.9	1494.5	0.9371	0.534	51.168

Peak#	Time	Area	Height	Width	Symmetry	Area%
1	9.931	1014.4	45	0.3757	0.283	2.760
2	12.256	35745.9	764.5	0.7793	0.562	97.240

(*S*)-2,2-Difluoro-*N*,*N*-dimethyl-4-phenyl-4-(4,4,5,5-tetramethyl-1,3,2-dioxaborolan-2-yl)butanamide (**4b**).

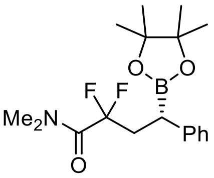

Colorless oil (48.7 mg, 69% yield, 98% ee); **^{1}H NMR** (400 MHz, $CDCl_3$) δ 7.30 – 7.24 (m, 4H), 7.22 – 7.12 (m, 1H), 3.16 (t, *J* = 2.0 Hz, 3H), 2.98 (s, 3H), 2.96 – 2.78 (m, 1H), 2.72 (dd, *J* = 10.1, 4.2 Hz, 1H), 2.45 (dtd, *J* = 22.1, 15.3, 4.2 Hz, 1H), 1.19 (s, 6H), 1.17 (s, 6H); **^{13}C {^{1}H} NMR** (101 MHz, $CDCl_3$) δ 163.4 (t, *J* = 29.2 Hz), 141.8, 128.3, 128.1, 125.5, 119.2 (t, *J* = 254.0 Hz), 83.5, 37.3 (t, *J* = 22.7 Hz), 36.9 (t, *J* = 7.0 Hz), 36.8, 24.4, 24.4; **^{19}F NMR** (377 MHz, $CDCl_3$) δ -98.60 – -99.56 (m, 1F), -99.56 – -100.50 (m, 1F), **^{11}B NMR** (128 MHz, $CDCl_3$) δ 32.51; **HRMS (EI)** m/z calcd for

$C_{18}H_{26}BF_2NO_3$ $[M+Na]^+$: 376.1872; Found: 376.1881.

Optical rotation: $[\alpha]_D^{25}$: 6.2 (c = 1.0, $CHCl_3$).

HPLC condition: Chiral column OD-H, n-Hexane/i-PrOH = 95:5, flow rate = 1.0 mL/min, wavelength = 210 nm, t_R = 5.3 min for the major isomer, t_R = 5.0 min for the minor isomer.

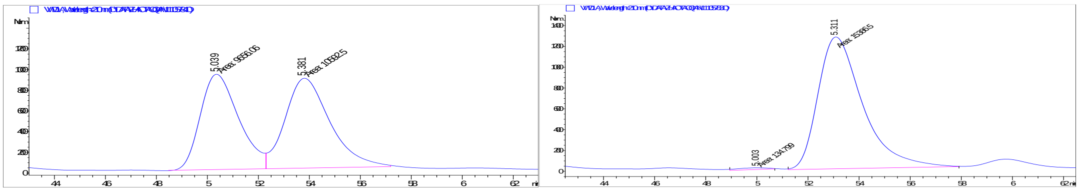

Peak#	Time	Area	Height	Width	Symmetry	Area%
1	5.039	9656.1	923.2	0.1743	0.8	47.688
2	5.381	10592.5	870.7	0.2027	0.645	52.312

Peak#	Time	Area	Height	Width	Symmetry	Area%
1	5.003	134.8	15.9	0.141	2.138	0.868
2	5.311	15386.5	1266.9	0.2024	0.645	99.132

(S)-*N,N*-dibenzyl-2,2-difluoro-4-hydroxy-4-phenylbutanamide (**4c**).

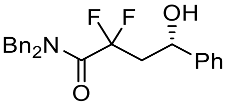

A subsequent oxidation of boronate was performed with $NaBO_3 \cdot 4H_2O$ (2.0 equiv) in THF/H_2O (1/1, 2 mL) at rt for 4 h. Colorless oil (55.3 mg, 70% yield, 92% ee); **^{1}H NMR** (400 MHz, $CDCl_3$) δ 7.51 – 7.30 (m, 11H), 7.29 – 7.25 (m, 2H), 7.25 – 7.19 (m, 2H), 5.16 (d, J = 9.1 Hz, 1H), 4.66 (dq, J = 69.8, 15.5, 14.8 Hz, 4H), 2.93 (ddt, J = 25.4, 15.3, 9.5 Hz, 1H), 2.60 (dtd, J = 17.6, 15.7, 2.3 Hz, 1H); **^{13}C {^{1}H} NMR** (126 MHz, $CDCl_3$) δ 164.8 (t, J = 29.4 Hz), 143.8, 135.5, 135.4, 128.9, 128.8, 128.6, 128.2, 128.0, 127.8, 127.6, 127.6, 125.6, 119.2 (t, J = 256.3 Hz), 68.3 (dd, J = 7.9, 3.8 Hz), 49.8 (t, J = 6.3 Hz), 48.4, 45.5 (t, J = 21.9 Hz); **^{19}F NMR** (377 MHz, $CDCl_3$) δ -90.28 (ddd, J = 288.9, 16.5, 9.7 Hz, 1F), -97.63 (ddd, J = 288.8, 26.1, 13.6 Hz, 1F); **HRMS** m/z (EI) calcd for $C_{24}H_{23}F_2NO_2$ $[M+Na]^+$: 418.1595; Found: 418.1599.

Optical rotation: $[\alpha]_D^{25}$: 22.3 (c = 1.00, $CHCl_3$).

HPLC condition: Chiral column OJ-H, n-Hexane/i-PrOH = 95:5, flow rate = 1.0 mL/min, wavelength = 220 nm, t_R = 42.3 min for the major isomer, t_R = 30.9 min for the minor isomer.

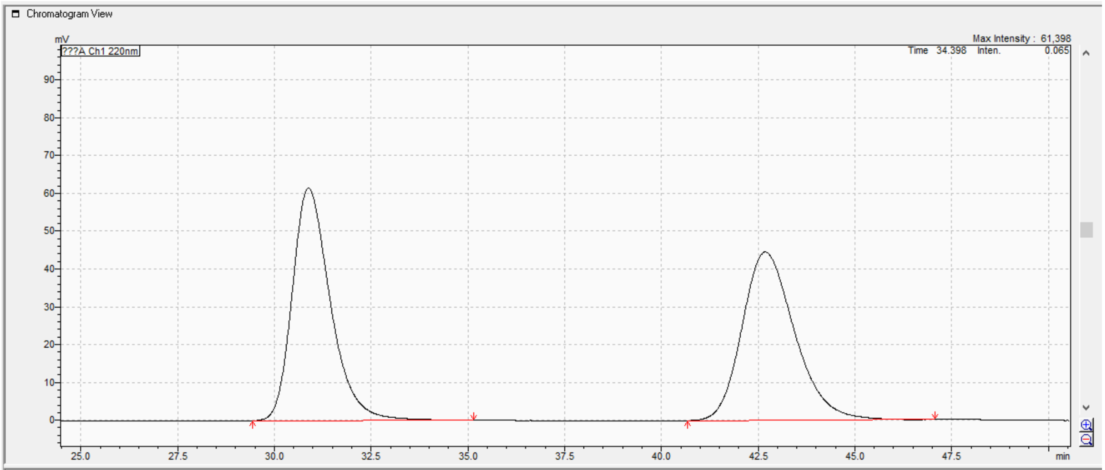

Results View - Peak Table

Peak Table | Compound | Group | Calibration Curve

Peak#	Ret. Time	Area	Height	Mark	Conc.	Unit	ID#	Name	Area%
1	30.883	4216421	61525	SV	50.077				50.077
2	42.685	4203438	44530	SV	49.923				49.923
Total		8419860	106055		100.000				100.000

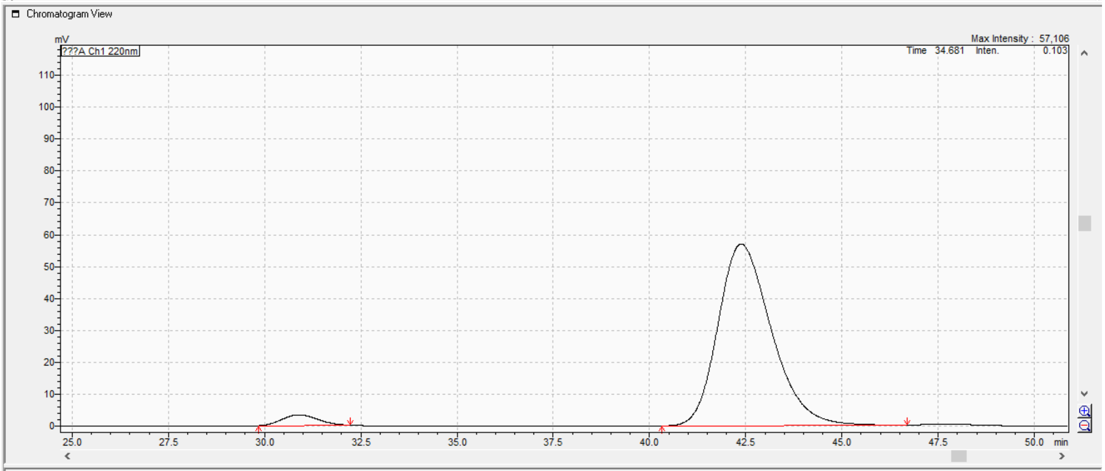

Results View - Peak Table

Peak Table | Compound | Group | Calibration Curve

Peak#	Ret. Time	Area	Height	Mark	Conc.	Unit	ID#	Name	Area%
1	30.910	215006	3359	M	3.780				3.780
2	42.381	5473436	56876	M	96.220				96.220
Total		5688442	60235		100.000				100.000

(*S*)-2,2-Difluoro-4-phenyl-1-(pyrrolidin-1-yl)-4-(4,4,5,5-tetramethyl-1,3,2-dioxaborolan-2-yl)butan-1-one (**4d**).

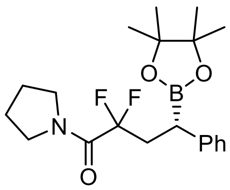

Colorless oil (68.9 mg, 91% yield, 91% ee); **^{1}H NMR** (400 MHz, $CDCl_3$) δ 7.32 – 7.22 (m, 4H), 7.16 (ddd, *J* = 10.0, 5.8, 3.0 Hz, 1H), 3.72 – 3.58 (m, 2H), 3.48 (t, *J* = 7.0 Hz, 2H), 2.92 – 2.74 (m, 1H), 2.71 (dd, *J* = 9.7, 4.5 Hz, 1H), 2.48 (dtd, *J* = 22.8, 14.5, 4.5 Hz, 1H), 1.93 (p, *J* = 6.6 Hz, 2H), 1.83 (p, *J* = 6.4 Hz, 2H), 1.19 (s, 6H), 1.17 (s, 6H); **^{13}C {^{1}H} NMR** (101 MHz, $CDCl_3$) δ 162.2 (t, *J* = 29.9 Hz), 141.6, 128.3, 128.1, 125.5, 118.9 (t, *J* = 253.0 Hz), 83.5, 47.2, 46.5 (t, *J* = 6.4 Hz), 36.8 (t, *J* = 22.7 Hz),

26.4, 24.4, 24.4, 23.2; **^{19}F NMR** (377 MHz, $CDCl_3$) δ -101.80 (ddd, J = 270.6, 23.1, 10.8 Hz, 1F), -103.48 (ddd, J = 270.7, 23.5, 14.8 Hz, 1F), **^{11}B NMR** (128 MHz, $CDCl_3$) δ 32.45; **HRMS (EI)** m/z calcd for $C_{20}H_{28}BF_2NO_3$ $[M+Na]^+$: 402.2028; Found: 402.2031.

Optical rotation: $[\alpha]_D^{25}$: 3.0 (c = 1.0, $CHCl_3$).

HPLC condition: Chiral column OD-H, n-Hexane/i-PrOH = 95:5, flow rate = 0.5 mL/min, wavelength = 220 nm, t_R = 23.3 min for the major isomer, t_R = 22.5 min for the minor isomer.

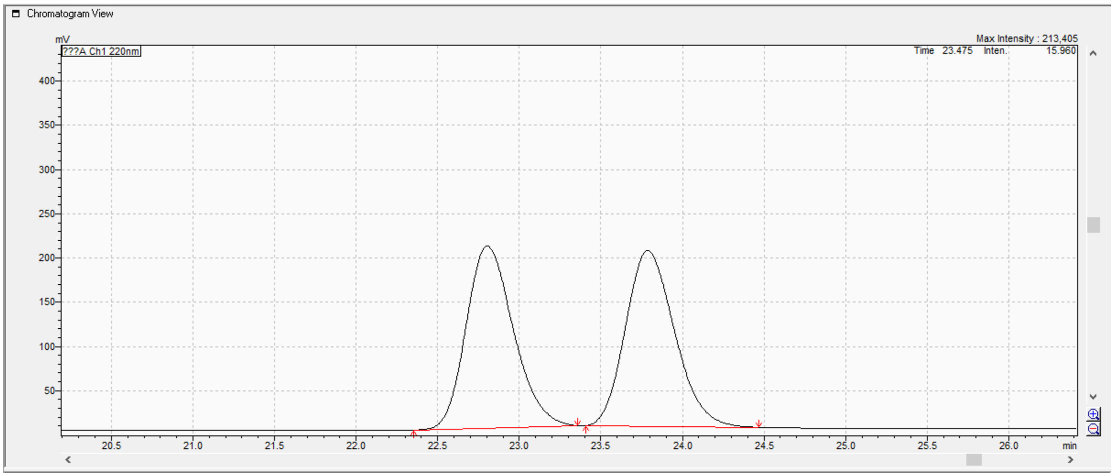

Results View - Peak Table

Peak Table | Compound | Group | Calibration Curve

Peak#	Ret. Time	Area	Height	Mark	Conc.	Unit	ID#	Name	Area%
1	22.803	4149663	205712	M	49.977				49.977
2	23.788	4153430	198342	M	50.023				50.023
Total		8303093	404054		100.000				100.000

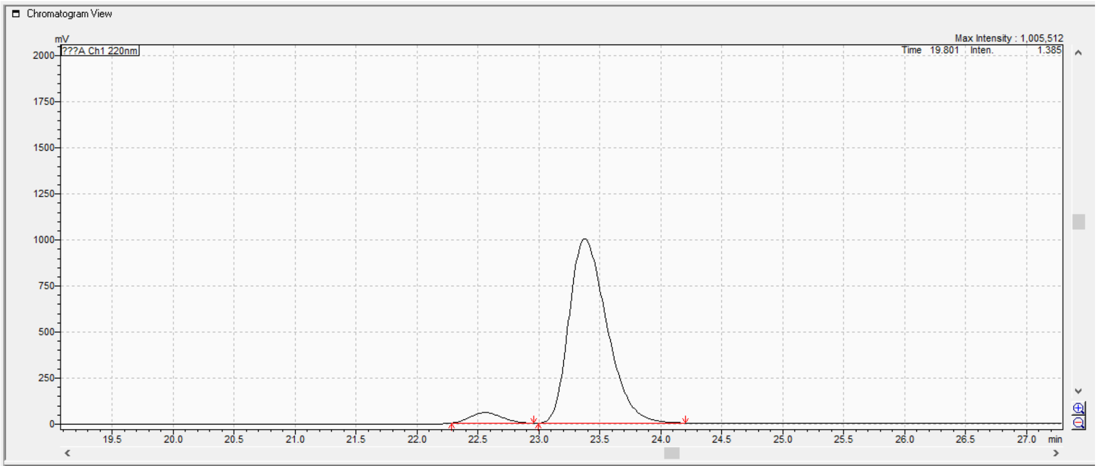

Results View - Peak Table

Peak Table | Compound | Group | Calibration Curve

Peak#	Ret. Time	Area	Height	Mark	Conc.	Unit	ID#	Name	Area%
1	22.556	1039679	57407	M	4.590				4.590
2	23.375	21612937	1000058	M	95.410				95.410
Total		22652616	1057465		100.000				100.000

(*S*)-2,2-Difluoro-4-phenyl-1-(piperidin-1-yl)-4-(4,4,5,5-tetramethyl-1,3,2-dioxaborolan-2-yl)butan-1-one (**4e**).

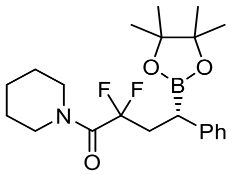

Colorless oil (74.6 mg, 95% yield, 94% ee); **^{1}H NMR** (400 MHz, $CDCl_3$) δ 7.32 – 7.23 (m, 4H), 7.22 – 7.11 (m, 1H), 3.64 (ddt, *J* = 25.1, 12.6, 5.8 Hz, 3H), 3.51 (dt, *J* = 12.7, 5.4 Hz, 1H), 2.88 (dddd, *J* = 19.7, 16.0, 14.6, 10.1 Hz, 1H), 2.73 (dd, *J* = 10.1, 4.1 Hz, 1H), 2.43 (dddd, *J* = 21.9, 18.3, 14.6, 4.1 Hz, 1H), 1.67 (tt, *J* = 7.6, 4.6 Hz, 2H), 1.60 (p, *J* = 5.4 Hz, 4H), 1.19 (s, 6H), 1.18 (s, 6H); **^{13}C {^{1}H} NMR** (101 MHz, $CDCl_3$) δ 162.5 – 160.5 (m), 141.9, 128.4, 128.1, 125.5, 119.3 (t, *J* = 254.0 Hz), 83.5, 46.8 (t, *J* = 6.5 Hz), 44.3, 37.6 (t, *J* = 22.6 Hz), 26.4, 25.5, 24.4, 24.4; **^{19}F NMR** (377 MHz, $CDCl_3$) δ -99.16 – -99.36 (m, 2F), **^{11}B NMR** (128 MHz, $CDCl_3$) δ 33.60; **HRMS (EI)** m/z calcd for $C_{21}H_{30}BF_2NO_3$ $[M+Na]^+$: 416.2185; Found: 416.2195.

Optical rotation: $[\alpha]_D^{25}$: 4.1 (*c* = 1.0, $CHCl_3$).

HPLC condition: Chiral column OD-H, n-Hexane/i-PrOH = 95:5, flow rate = 0.5 mL/min, wavelength = 220 nm, t_R = 18.1 min for the major isomer, t_R = 17.4 min for the minor isomer.

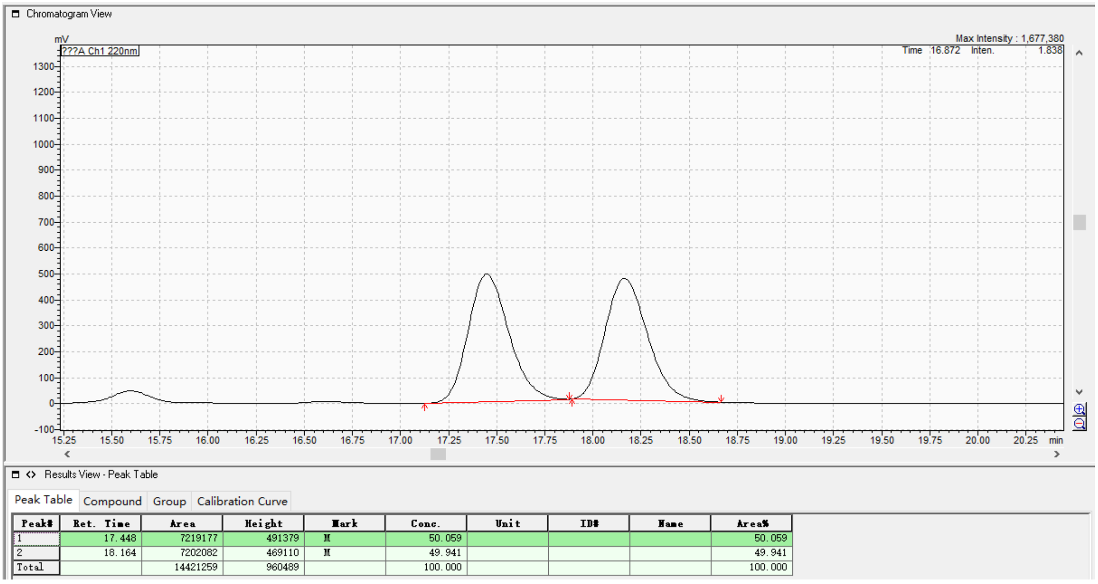

Peak#	Ret. Time	Area	Height	Mark	Conc.	Unit	ID#	Name	Area%
1	17.448	7219177	491379	M	50.059				50.059
2	18.164	7202082	469110	M	49.941				49.941
Total		14421259	960489		100.000				100.000

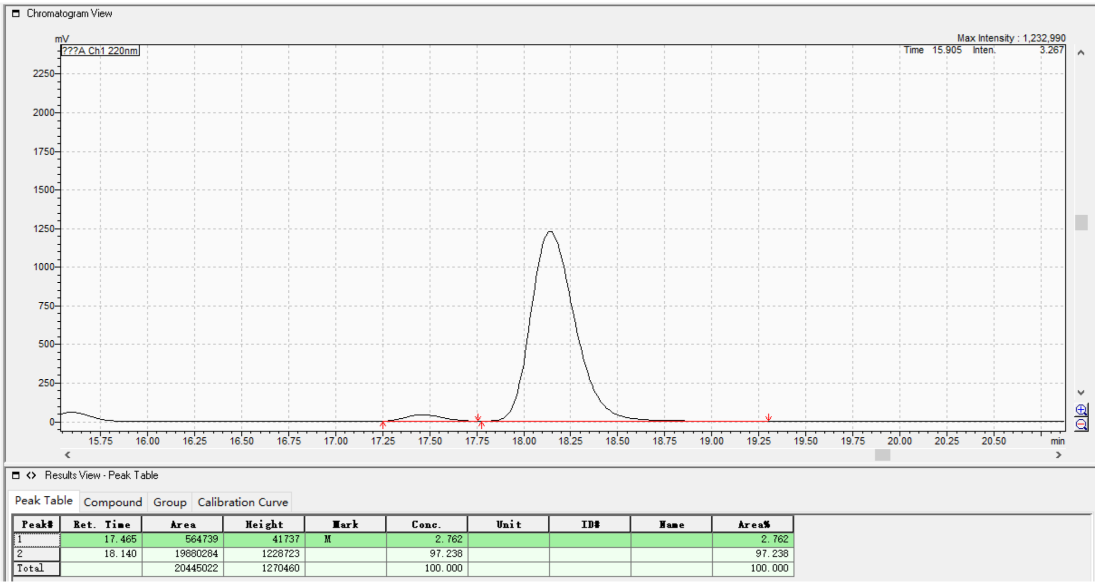

Peak#	Ret. Time	Area	Height	Mark	Conc.	Unit	ID#	Name	Area%
1	17.465	564739	41737	M	2.762				2.762
2	18.140	19880284	1228723		97.238				97.238
Total		20445022	1270460		100.000				100.000

(*S*)-2,2-Difluoro-4-hydroxy-1-morpholino-4-phenylbutan-1-one (4f).

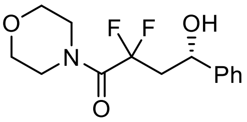

A subsequent oxidation of boronate was performed with $NaBO_3{\cdot}4H_2O$ (2.0 equiv) in THF/H_2O (1/1) at rt for 4 h. Colorless oil (36.4 mg, 64% yield, 94% ee); **^{1}H NMR** (400 MHz, $CDCl_3$) δ 7.46 – 7.34 (m, 4H), 7.33 – 7.25 (m, 1H), 5.05 (d, J = 9.1 Hz, 1H), 4.08 (s, 1H), 3.74 (ddt, J = 22.3, 18.1, 5.0 Hz, 9H), 2.76 (ddt, J = 25.4, 15.3, 9.6 Hz, 1H), 2.49 (tdd, J = 16.1, 14.0, 2.4 Hz, 1H); **^{13}C {^{1}H} NMR** (101 MHz, $CDCl_3$) δ 162.7 (t, J = 29.6 Hz), 143.7, 128.5, 127.6, 125.6, 118.8 (dd), 68.1 (dd, J = 8.2, 3.8 Hz), 66.6, 66.6, 46.6 (t, J = 6.2 Hz), 45.2 (t, J = 21.9 Hz), 43.6; **^{19}F NMR** (377 MHz, $CDCl_3$) δ -92.02 (ddd, J = 289.3, 16.4, 9.9 Hz, 1F), -98.62 (ddd, J = 289.2, 26.0, 14.0 Hz, 1F); **HRMS (EI)** m/z calcd for $C_{14}H_{17}F_2NO_3$ $[M+Na]^+$: 308.1074; Found: 308.1078.

Optical rotation: $[\alpha]_D^{25}$: 6.0 (c = 1.0, $CHCl_3$).

HPLC condition: Chiral column OJ-H, n-Hexane/i-PrOH = 90:10, flow rate = 1.0 mL/min, wavelength = 220 nm, t_R = 31.4 min for the major isomer, t_R = 23.8 min for the minor isomer.

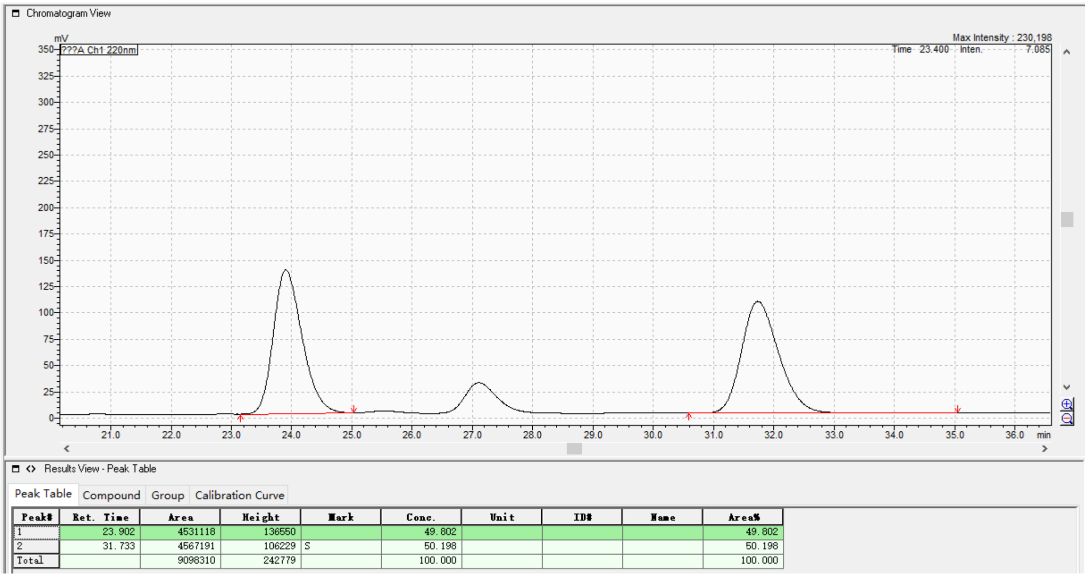

Peak#	Ret. Time	Area	Height	Mark	Conc.	Unit	ID#	Name	Area%
1	23.902	4531118	136550		49.802				49.802
2	31.733	4567191	106229	S	50.198				50.198
Total		9098310	242779		100.000				100.000

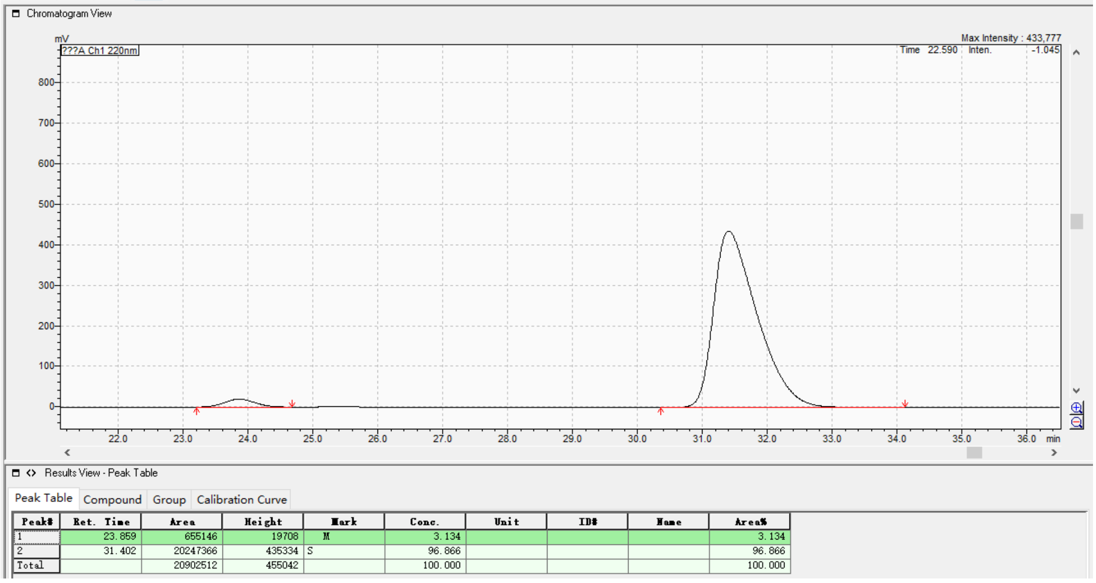

Peak#	Ret. Time	Area	Height	Mark	Conc.	Unit	ID#	Name	Area%
1	23.859	655146	19708	M	3.134				3.134
2	31.402	20247366	435334	S	96.866				96.866
Total		20902512	455042		100.000				100.000

Tert-butyl (*S*)-4-(2,2-difluoro-4-hydroxy-4-phenylbutanoyl)piperazine-1-carboxylate (4g).

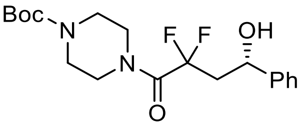

A subsequent oxidation of boronate was performed with $NaBO_3 \cdot 4H_2O$ (2.0 equiv) in THF/H_2O (1/1) at rt for 4 h. Colorless oil (46.1 mg, 60% yield, 94% ee); **^{1}H NMR** (400 MHz, $CDCl_3$) δ 7.37 – 7.24 (m, 4H), 7.24 – 7.17 (m, 1H), 4.95 (d, *J* = 9.2 Hz, 1H), 3.94 (d, *J* = 2.9 Hz, 1H), 3.61 (dt, *J* = 36.7, 5.2 Hz, 4H), 3.43 (dd, *J* = 5.9, 3.7 Hz, 4H), 2.66 (ddt, *J* = 25.3, 15.3, 9.6 Hz, 1H), 2.40 (tdd, *J* = 15.9, 14.1, 2.4 Hz, 1H), 1.40 (s, 9H); **^{13}C {^{1}H} NMR** (101 MHz, $CDCl_3$) δ 162.8 (t, *J* = 29.6 Hz), 154.4, 143.7, 128.5,

127.6, 125.6, 118.8 (t, J = 256.3 Hz), 80.5, 68.1 (dd, J = 8.1, 3.7 Hz), 45.8 (t, J = 6.1 Hz), 45.2 (t, J = 21.9 Hz), 43.2, 28.3; **^{19}F NMR** (377 MHz, $CDCl_3$) δ -91.08 – -92.78 (m, 1F), -97.70 – -99.46 (m, 1F); **HRMS (EI)** m/z calcd for $C_{19}H_{26}F_2N_2O_4$ $[M+Na]^+$: 407.1758; Found: 407.1768.

Optical rotation: $[\alpha]_D^{25}$: 3.9 (c = 1.0, $CHCl_3$).

HPLC condition: Chiral column OJ-H, n-Hexane/i-PrOH = 90:10, flow rate = 1.0 mL/min, wavelength = 220 nm, t_R = 25.6 min for the major isomer, t_R = 17.7 min for the minor isomer.

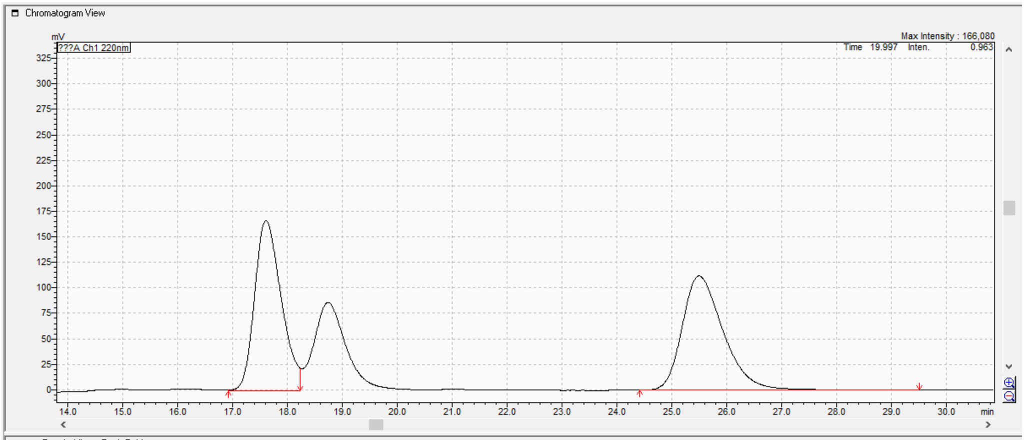

Results View - Peak Table

Peak Table | Compound | Group | Calibration Curve

Peak#	Ret. Time	Area	Height	Mark	Conc.	Unit	ID#	Name	Area%
1	17.604	5607958	166721	M	49.108				49.108
2	25.497	5811606	111826	S	50.892				50.892
Total		11419565	278547		100.000				100.000

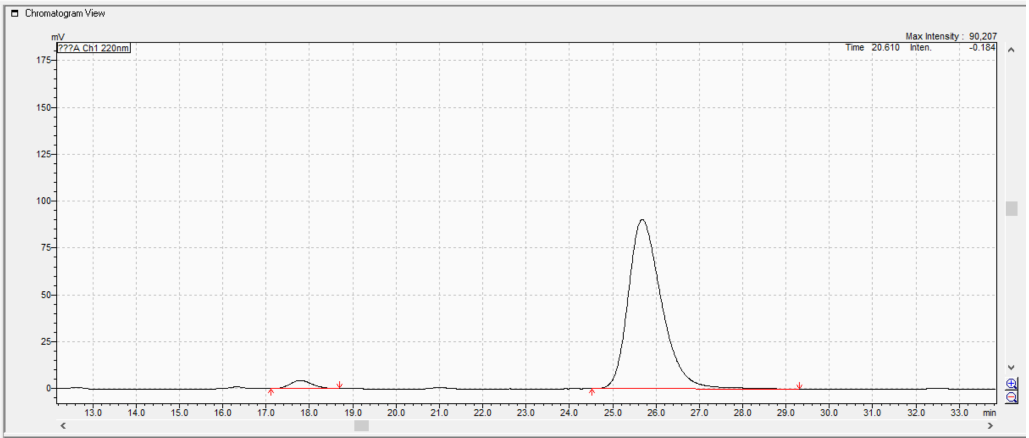

Results View - Peak Table

Peak Table | Compound | Group | Calibration Curve

Peak#	Ret. Time	Area	Height	Mark	Conc.	Unit	ID#	Name	Area%
1	17.786	152848	4337		3.060				3.060
2	25.681	4842703	90316		96.940				96.940
Total		4995551	94653		100.000				100.000

(*S*)-1-(azepan-1-yl)-2,2-Difluoro-4-phenyl-4-(4,4,5,5-tetramethyl-1,3,2-dioxaborolan-2-yl)butan-1-one (**4h**).

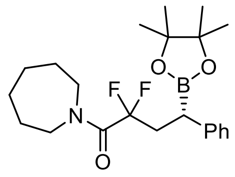

Colorless oil (74.9 mg, 92% yield, 90% ee); **^{1}H NMR** (400 MHz, $CDCl_3$) δ 7.30 – 7.24 (m, 4H), 7.16 (dt, *J* = 8.7, 4.1 Hz, 1H), 3.75 – 3.42 (m, 4H), 2.88 (dtd, *J* = 21.9, 14.2, 10.2 Hz, 1H), 2.73 (dd, *J* = 10.2, 4.1 Hz, 1H), 2.46 (dddd, *J* = 22.1, 15.9, 14.6, 4.1 Hz, 1H), 1.84 – 1.71 (m, 4H), 1.65 – 1.54 (m, 4H), 1.19 (s, 6H), 1.18 (s, 6H); **^{13}C {^{1}H} NMR** (101 MHz, $CDCl_3$) δ 163.1 (t, *J* = 28.8 Hz), 141.8, 128.3, 128.1, 125.5, 119.5 (t, *J* = 254.3 Hz), 83.5, 48.2, 47.8 (t, *J* = 6.0 Hz), 37.6 (t, *J* = 22.8 Hz), 29.7, 27.3, 26.4, 26.0, 24.4, 24.4; **^{19}F NMR** (377 MHz, $CDCl_3$) δ -98.36 – -99.23 (m, 1F), -99.22 – -100.15 (m, 1F), **^{11}B NMR** (128 MHz, $CDCl_3$) δ 33.45; **HRMS (EI)** m/z calcd for $C_{22}H_{32}BF_2NO_3$ $[M+Na]^+$: 430.2341; Found: 430.2347.

Optical rotation: $[\alpha]_D^{25}$: 3.7 (*c* = 1.0, $CHCl_3$).

HPLC condition: Chiral column OD-H, n-Hexane/i-PrOH = 95:5, flow rate = 0.5 mL/min, wavelength = 220 nm, t_R = 17.7 min for the major isomer, t_R = 17.0 min for the minor isomer.

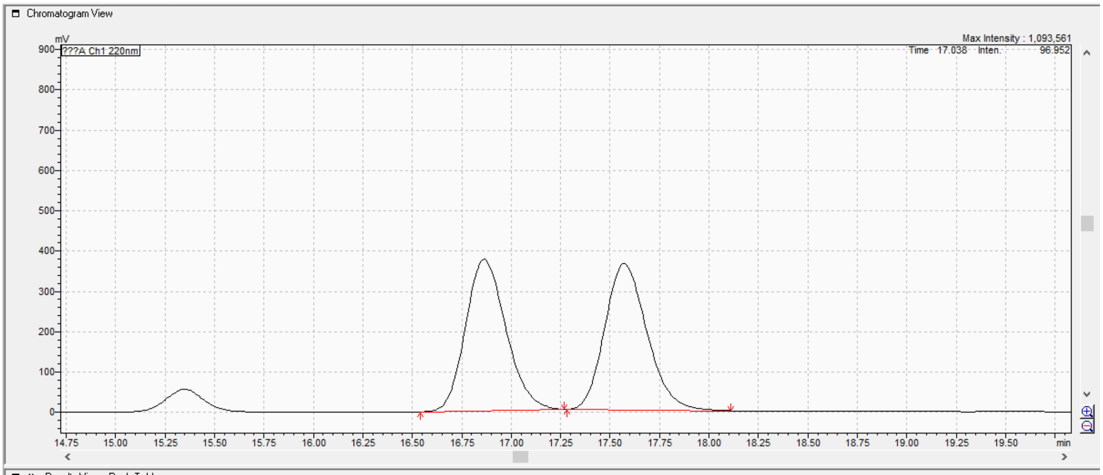

Results View - Peak Table

Peak Table | Compound | Group | Calibration Curve

Peak#	Ret. Time	Area	Height	Mark	Conc.	Unit	ID#	Name	Area%
1	16.863	5348807	375695	M	49.806				49.806
2	17.570	5390557	362754	M	50.194				50.194
Total		10739364	738449		100.000				100.000

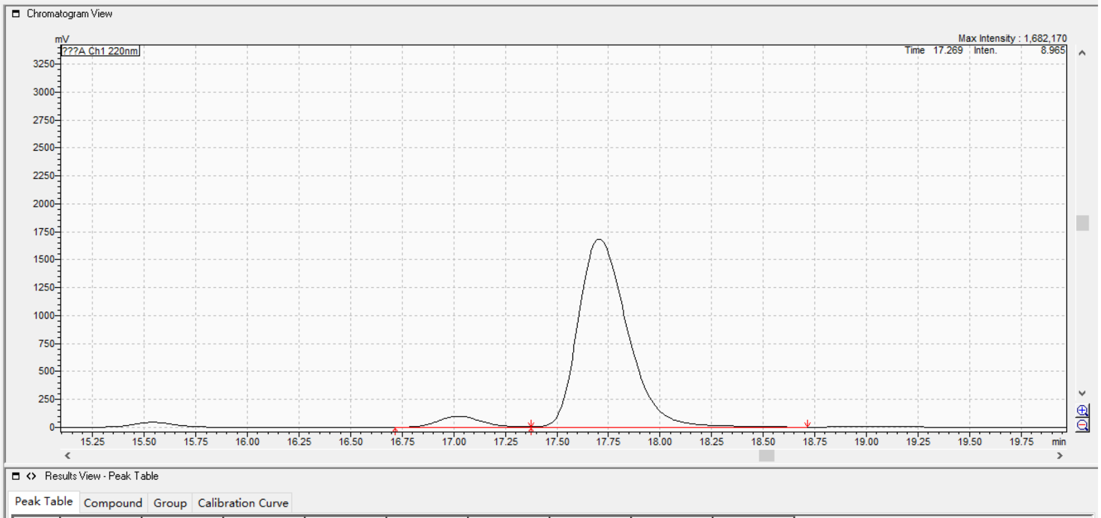

Peak#	Ret. Time	Area	Height	Mark	Conc.	Unit	ID#	Name	Area%
1	17.021	1415189	99197		4.852				4.852
2	17.705	27749972	1680835	V	95.148				95.148
Total		29165160	1780033		100.000				100.000

(*S*)-2,2-Difluoro-4-phenyl-4-(4,4,5,5-tetramethyl-1,3,2-dioxaborolan-2-yl)butyl acetate (**4i**).

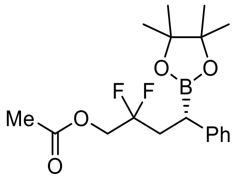

Colorless oil (60.2 mg, 85% yield, 98% ee); **^{1}H NMR** (400 MHz, $CDCl_3$) δ 7.32 – 7.22 (m, 4H), 7.21 – 7.15 (m, 1H), 4.20 (ddd, *J* = 14.4, 11.7, 9.6 Hz, 2H), 2.73 – 2.49 (m, 2H), 2.34 – 2.13 (m, 1H), 2.11 (s, 3H), 1.20 (s, 6H), 1.18 (s, 6H); **^{13}C {^{1}H} NMR** (101 MHz, $CDCl_3$) δ 169.7, 141.4, 128.5, 128.0, 125.8, 121.4 (t, *J* = 242.8 Hz), 83.6, 64.0 (dd, *J* = 33.8, 32.0 Hz), 36.6 (t, *J* = 23.4 Hz), 24.4, 24.4, 20.5; **^{19}F NMR** (377 MHz, $CDCl_3$) δ -102.35 – -103.77 (m, 1F), -106.41 (m, 1F), **^{11}B NMR** (128 MHz, $CDCl_3$) δ 33.34; **HRMS (EI)** m/z calcd for $C_{18}H_{25}BF_2O_4$ $[M+Na]^+$: 377.1712; Found: 377.1710.

Optical rotation: $[\alpha]_D^{25}$: -2.1 (*c* = 1.0, $CHCl_3$).

HPLC condition: Chiral column OD-H, n-Hexane/i-PrOH = 99.5:0.5, flow rate = 0.5 mL/min, wavelength = 220 nm, t_R = 14.6 min for the major isomer, t_R = 16.9 min for the minor isomer.

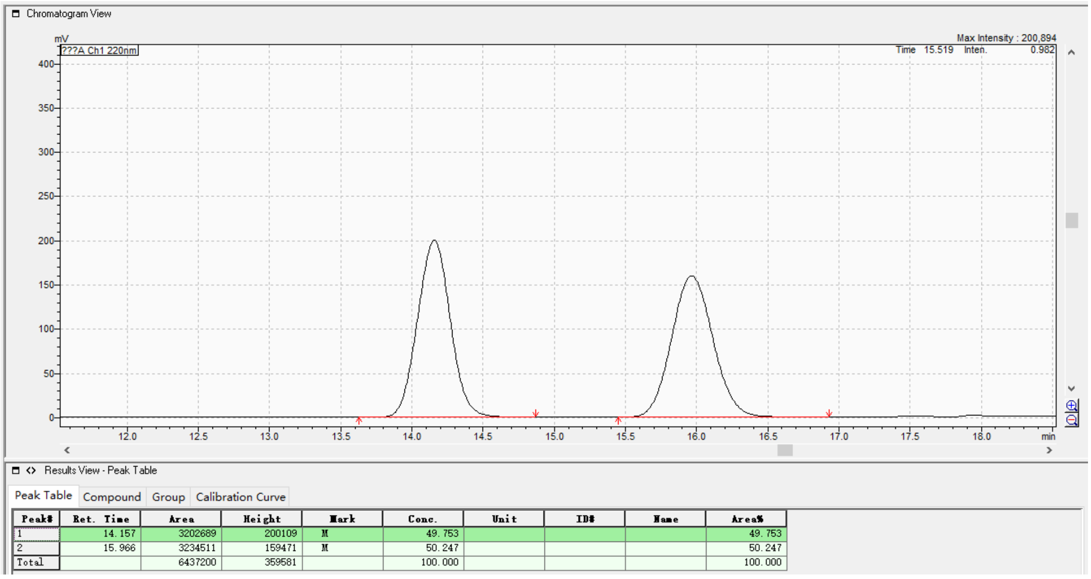

Peak#	Ret. Time	Area	Height	Mark	Conc.	Unit	ID#	Name	Area%
1	14.157	3202689	200109	M	49.753				49.753
2	15.966	3234511	159471	M	50.247				50.247
Total		6437200	359581		100.000				100.000

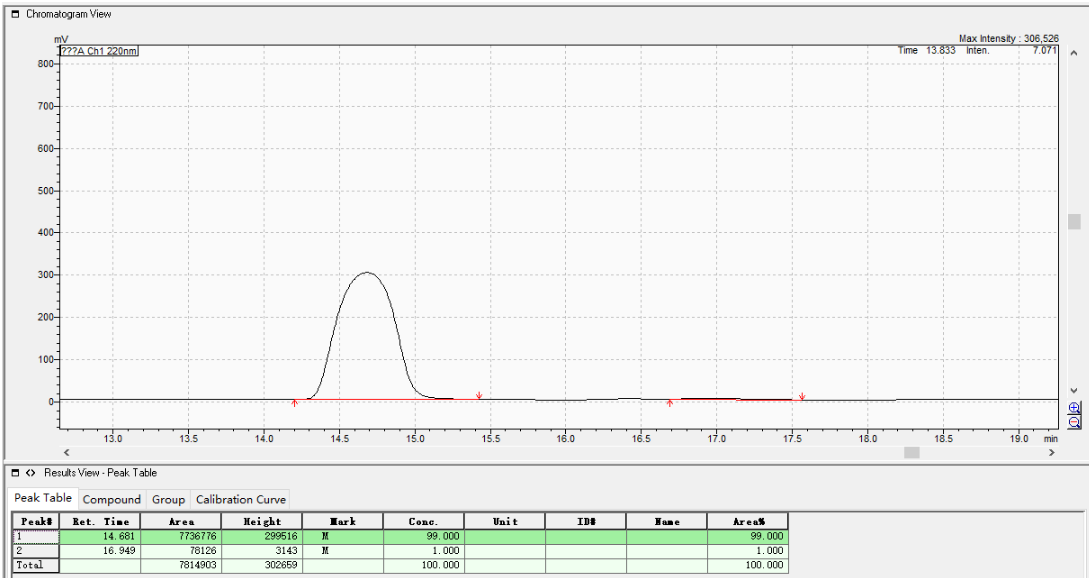

Peak#	Ret. Time	Area	Height	Mark	Conc.	Unit	ID#	Name	Area%
1	14.681	7736776	299516	M	99.000				99.000
2	16.949	78126	3143	M	1.000				1.000
Total		7814903	302659		100.000				100.000

(*S*)-2,2-Difluoro-4-phenyl-4-(4,4,5,5-tetramethyl-1,3,2-dioxaborolan-2-yl)butyl pivalate (4j).

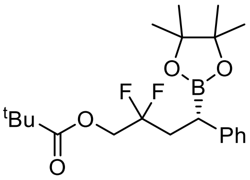

Colorless oil (64.1 mg, 81% yield, 95% ee); **^{1}H NMR** (400 MHz, $CDCl_3$) δ 7.33 – 7.21 (m, 4H), 7.21 – 7.14 (m, 1H), 4.32 – 4.09 (m, 2H), 2.73 – 2.50 (m, 2H), 2.32 – 2.11 (m, 1H), 1.23 (s, 9H), 1.20 (s, 6H), 1.18 (s, 6H); **^{13}C {^{1}H} NMR** (101 MHz, $CDCl_3$) δ 177.3, 141.4, 128.5, 128.0, 125.7, 121.6 (t, *J* = 242.9 Hz), 83.6, 63.7 (dd, *J* = 34.8, 31.9 Hz), 38.8, 36.6 (t, *J* = 23.4 Hz), 27.0, 24.4, 24.4; **^{19}F NMR** (377 MHz, $CDCl_3$) δ -102.48 – -103.73 (m, 1F), -105.73 – -106.95 (m, 1F), **^{11}B NMR** (128 MHz,

$CDCl_3$) δ 33.48; **HRMS (EI)** m/z calcd for $C_{21}H_{31}BF_2O_4$ $[M+Na]^+$: 419.2181; Found: 419.2171.

Optical rotation: $[\alpha]_D^{25}$: 3.8 (*c* = 1.0, $CHCl_3$).

HPLC condition: Chiral column OJ-H, n-Hexane/i-PrOH = 99.5:0.5, flow rate = 0.5 mL/min, wavelength = 220 nm, tR = 26.7 min for the major isomer, tR = 21.9 min for the minor isomer.

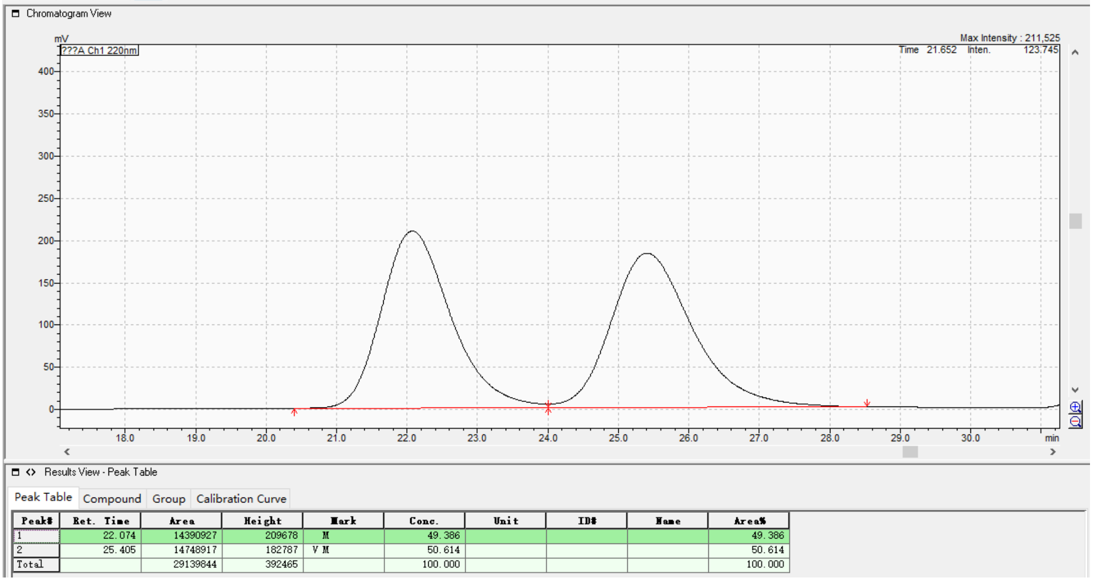

Peak#	Ret. Time	Area	Height	Mark	Conc.	Unit	ID#	Name	Area%
1	22.074	14390927	209678	M	49.386				49.386
2	25.405	14748917	182787	V M	50.614				50.614
Total		29139844	392465		100.000				100.000

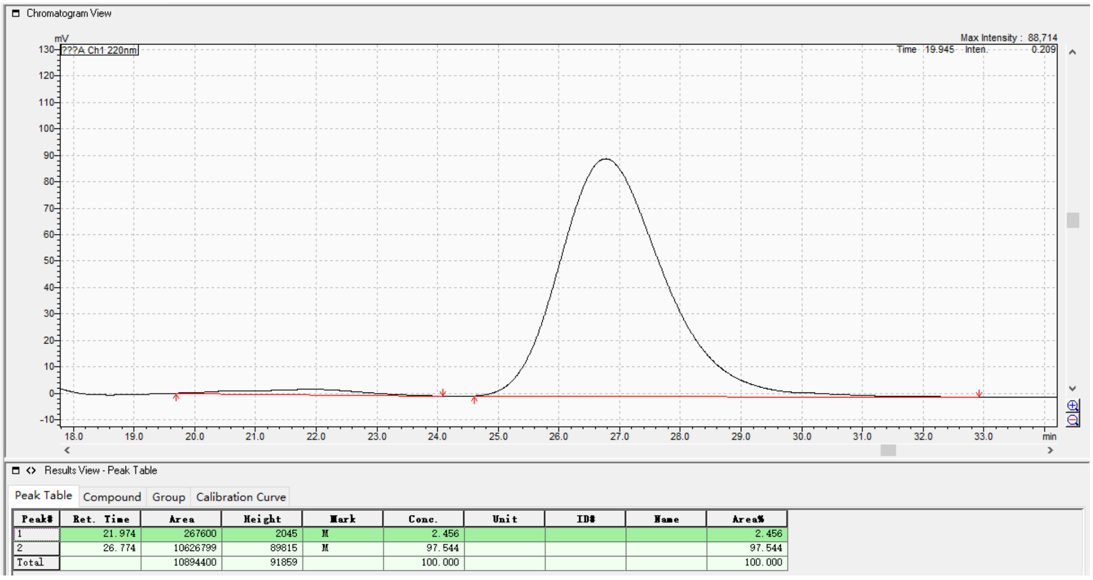

Peak#	Ret. Time	Area	Height	Mark	Conc.	Unit	ID#	Name	Area%
1	21.974	267600	2045	M	2.456				2.456
2	26.774	10626799	89815	M	97.544				97.544
Total		10894400	91859		100.000				100.000

(*S*)-2,2-Difluoro-4-phenyl-4-(4,4,5,5-tetramethyl-1,3,2-dioxaborolan-2-yl)butyl benzoate (**4k**).

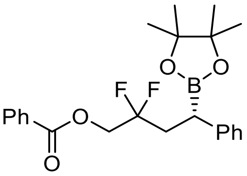

Colorless oil (79.9 mg, 96% yield, 95% ee); **^{1}H NMR** (400 MHz, $CDCl_3$) δ 8.12 – 8.04 (m, 2H), 7.66 – 7.56 (m, 1H), 7.48 (t, *J* = 7.8 Hz, 2H), 7.28 (d, *J* = 4.3 Hz, 4H), 7.22 – 7.13 (m, 1H), 4.44 (dt, *J* = 26.3, 12.1 Hz, 2H), 2.84 – 2.61 (m, 2H), 2.45 – 2.21 (m, 1H), 1.21 (s, 6H), 1.19 (s, 6H); **^{13}C {^{1}H} NMR** (101 MHz, $CDCl_3$) δ 165.4, 141.4, 133.3, 129.8, 129.2, 128.5, 128.4, 128.0, 125.8, 121.6 (t, *J* = 242.9 Hz), 83.7, 64.3 (dd, *J* = 34.4, 32.3 Hz), 36.8 (t, *J* = 23.4 Hz), 24.4, 24.4; **^{19}F NMR** (377 MHz, $CDCl_3$) δ -102.25 – -103.47 (m, 1F), -105.30 – -106.62 (m, 1F), **^{11}B NMR** (128 MHz, $CDCl_3$) δ 33.74; **HRMS (EI)** m/z calcd for $C_{23}H_{27}BF_2O_4$ $[M+Na]^+$: 439.1868; Found: 439.1862.

Optical rotation: $[\alpha]_D^{25}$: 1.3 (*c* = 1.0, $CHCl_3$).

HPLC condition: Chiral column OD-H, n-Hexane/i-PrOH = 99.5:0.5, flow rate = 0.5 mL/min, wavelength = 220 nm, t_R = 14.4 min for the major isomer, t_R = 13.9 min for the minor isomer.

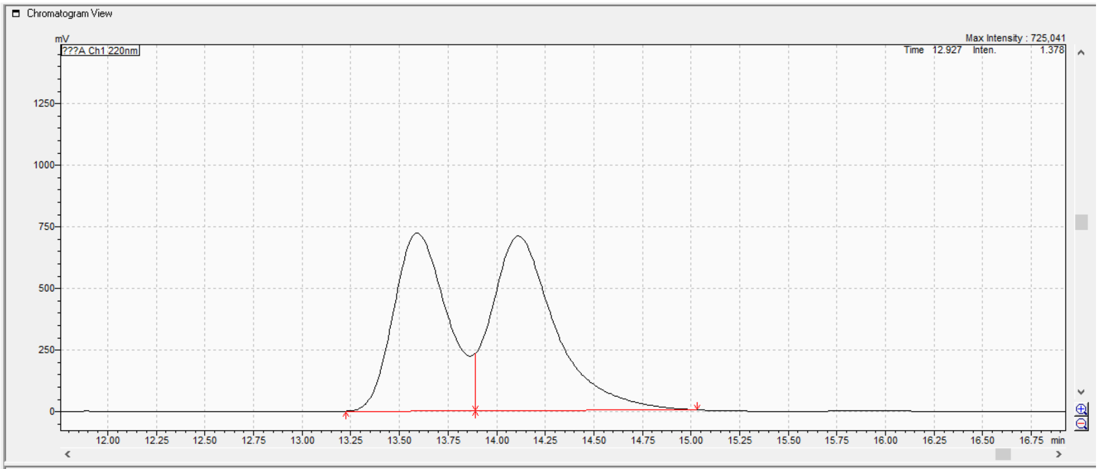

Results View - Peak Table

Peak Table | Compound | Group | Calibration Curve

Peak#	Ret. Time	Area	Height	Mark	Conc.	Unit	ID#	Name	Area%
1	13.591	13888133	721529	M	46.479				46.479
2	14.111	15992451	707461	V M	53.521				53.521
Total		29880584	1428989		100.000				100.000

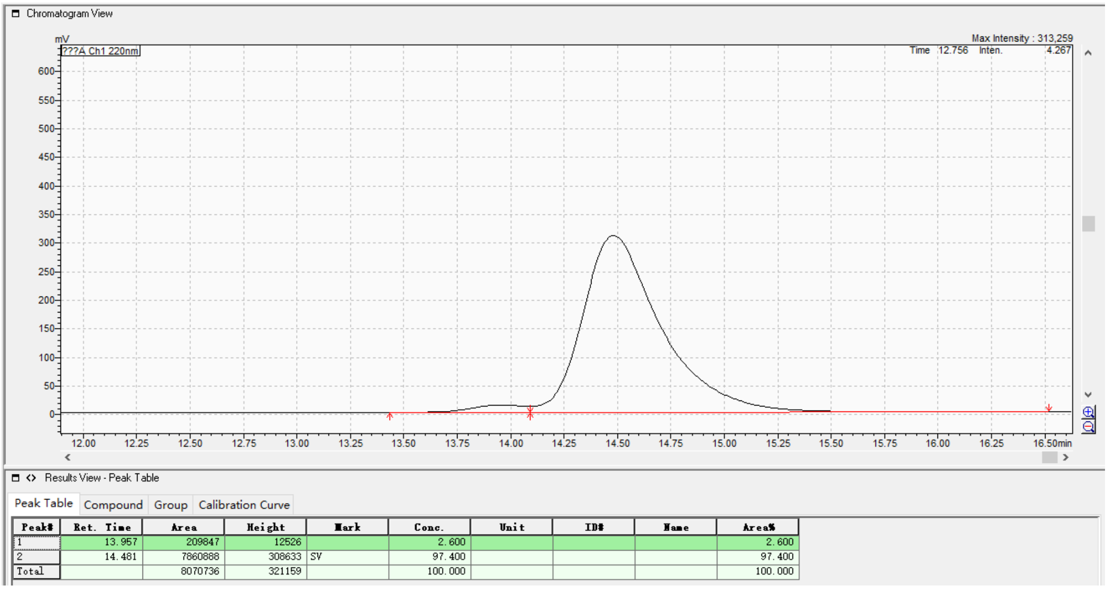

Peak#	Ret. Time	Area	Height	Mark	Conc.	Unit	ID#	Name	Area%
1	13.957	209847	12526		2.600				2.600
2	14.481	7860888	308633	SV	97.400				97.400
Total		8070736	321159		100.000				100.000

Ethyl (*S*)-4-(4-((*S*)-2-((tert-butoxycarbonyl)amino)-3-methoxy-3-oxopropyl)phenyl)-2,2-difluoro-4-(4,4,5,5-tetramethyl-1,3,2-dioxaborolan-2-yl)butanoate (**6a**).

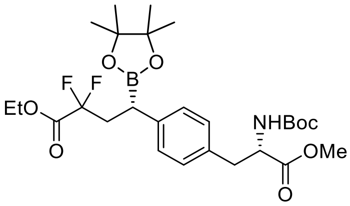

Colorless oil (52.2 mg, 47% yield, 96:4 dr); **^{1}H NMR** (400 MHz, $CDCl_3$) δ 7.20 – 7.10 (m, 2H), 7.03 (d, *J* = 7.8 Hz, 2H), 4.96 (d, *J* = 8.3 Hz, 1H), 4.56 (q, *J* = 6.7 Hz, 1H), 4.24 – 4.07 (m, 2H), 3.70 (s, 3H), 3.04 (qd, *J* = 14.0, 5.9 Hz, 2H), 2.84 – 2.64 (m, 1H), 2.64 – 2.56 (m, 1H), 2.41 (dddd, *J* = 25.1, 21.7, 13.8, 7.4 Hz, 1H), 1.43 (s, 9H), 1.30 (t, *J* = 7.1 Hz, 3H), 1.19 (s, 6H), 1.17 (s, 6H); **^{13}C {^{1}H} NMR** (101 MHz, $CDCl_3$) δ 172.3, 164.1 (t, *J* = 32.9 Hz), 155.0, 139.4, 133.5, 129.4, 128.4, 115.9 (dd, *J* = 251.3, 249.7 Hz), 83.8, 79.9, 62.6, 54.4, 52.1, 37.8, 36.9 (t, *J* = 23.0 Hz), 28.3, 24.4, 24.4, 13.8; **^{19}F NMR** (377 MHz, $CDCl_3$) δ -103.13 (ddd, *J* = 258.9, 19.7, 10.1 Hz, 1F), -107.23 (dt, *J* = 258.9, 19.0 Hz, 1F), **^{11}B NMR** (128 MHz, $CDCl_3$) δ 33.08; **HRMS (EI)** m/z calcd for $C_{27}H_{40}BF_2NO_8$ $[M+Na]^+$: 578.2713; Found: 578.2716.

Optical rotation: $[\alpha]_D^{25}$: 11.7 (*c* = 1.10, $CHCl_3$).

Diastereomeric ratios were determined by crude ^{1}H NMR and ^{19}F NMR spectrum

that integrate at least two pairs of signals corresponding to the two diastereomers present in the sample.

(1*R*,2*S*,4*R*)-1,7,7-Trimethylbicyclo[2.2.1]heptan-2-yl (*S*)-2,2-difluoro-4-phenyl-4-(4,4,5,5-tetramethyl-1,3,2-dioxaborolan-2-yl)butanoate (**6b**).

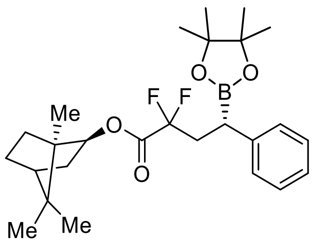

Colorless oil (72.1 mg, 78% yield, 98:2 dr); **^{1}H NMR** (400 MHz, $CDCl_3$) δ 7.32 – 7.22 (m, 5H), 7.21 – 7.14 (m, 1H), 4.96 (dt, *J* = 10.2, 2.6 Hz, 1H), 2.76 (ddt, *J* = 20.1, 14.0, 10.0 Hz, 1H), 2.64 (dd, *J* = 9.5, 5.0 Hz, 1H), 2.53 – 2.31 (m, 2H), 1.94 (ddd, *J* = 13.1, 9.2, 4.3 Hz, 1H), 1.78 (dp, *J* = 11.6, 3.9 Hz, 1H), 1.72 (t, *J* = 4.5 Hz, 1H), 1.38 – 1.23 (m, 2H), 1.21 (s, 6H), 1.19 (s, 6H), 0.99 (dd, *J* = 14.0, 3.5 Hz, 1H), 0.92 (s, 3H), 0.90 (s, 3H), 0.87 (s, 3H); **^{13}C {^{1}H} NMR** (101 MHz, $CDCl_3$) δ 164.3 (t, *J* = 32.8 Hz), 141.0, 128.5, 128.1, 125.8, 113.7 (dd, *J* = 251.3, 250.1 Hz), 83.7, 82.7, 49.0, 47.9, 44.8, 37.1 (t, *J* = 22.8 Hz), 36.3, 27.8, 26.9, 24.4, 24.4, 19.6, 18.8, 13.4; **^{19}F NMR** (377 MHz, $CDCl_3$) δ -102.97 (ddd, *J* = 258.3, 19.2, 10.3 Hz, 1F), -106.52 (ddd, *J* = 258.3, 20.6, 17.2 Hz, 1F), **^{11}B NMR** (128 MHz, $CDCl_3$) δ 32.85; **HRMS (EI)** m/z calcd for $C_{26}H_{37}BF_2O_4$ $[M+Na]^+$: 485.2651; Found: 485.2658.

Optical rotation: $[\alpha]_D^{25}$: -5.9 (*c* = 1.0, $CHCl_3$).

Diastereomeric ratios were determined by crude ^{1}H NMR and ^{19}F NMR spectrum that integrate at least two pairs of signals corresponding to the two diastereomers present in the sample.

Ethyl (*S*)-2,2-difluoro-4-((8*R*,9*S*,13*S*,14*S*,17*S*)-17-methoxy-13-methyl-7,8,9,11,12,13,14,15,16,17-decahydro-6H-cyclopenta[a]phenanthren-3-yl)-4-(4,4,5,5-tetramethyl-1,3,2-dioxaborolan-2-yl)butanoate (6c).

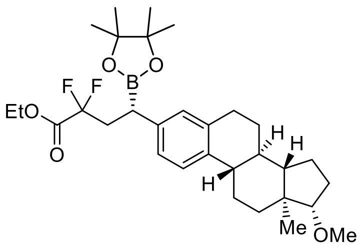

Colorless oil (66.6 mg, 61% yield, >99:1 dr); **^{1}H NMR** (400 MHz, $CDCl_3$) δ 7.19 (d, *J* = 8.0 Hz, 1H), 6.99 (dd, *J* = 8.1, 2.0 Hz, 1H), 6.93 (d, *J* = 1.9 Hz, 1H), 4.16 (qd, *J* = 7.2, 2.5 Hz, 2H), 3.40 (s, 3H), 3.33 (t, *J* = 8.3 Hz, 1H), 2.93 – 2.76 (m, 2H), 2.78 – 2.59 (m, 1H), 2.56 (dd, *J* = 9.2, 5.3 Hz, 1H), 2.50 – 2.26 (m, 2H), 2.21 (td, *J* = 10.9, 4.1 Hz, 1H), 2.16 – 2.01 (m, 2H), 1.89 (ddt, *J* = 12.4, 5.6, 2.6 Hz, 1H), 1.71 (dddd, *J* = 12.2, 9.7, 6.9, 3.1 Hz, 1H), 1.60 – 1.31 (m, 6H), 1.29 (t, *J* = 7.1 Hz, 3H), 1.23 (s, 6H), 1.21 (s, 6H), 0.81 (s, 3H); **^{13}C {^{1}H} NMR** (101 MHz, $CDCl_3$) δ 164.1 (t, *J* = 32.9 Hz), 137.8, 137.8, 136.7, 128.7, 125.5, 125.4, 116.0 (dd, *J* = 251.3, 249.5 Hz), 90.8, 83.7, 62.5, 57.8, 50.4, 44.2, 43.2, 38.4, 38.1, 37.4 (t, *J* = 22.8 Hz), 29.5, 27.7, 27.2, 26.2, 24.5, 24.4, 23.0, 13.8, 11.5; **^{19}F NMR** (377 MHz, $CDCl_3$) δ -102.79 (ddd, *J* = 259.0, 19.4, 10.4 Hz, 1F), -107.28 (ddd, *J* = 259.0, 20.4, 17.6 Hz, 1F), **^{11}B NMR** (128 MHz, $CDCl_3$) δ 33.17; **HRMS (EI)** m/z calcd for $C_{31}H_{45}BF_2O_5$ $[M+Na]^+$: 569.3226; Found: 569.3215.

Optical rotation: $[\alpha]_D^{25}$: 1.5 (*c* = 1.0, $CHCl_3$).

Diastereomeric ratios were determined by crude ^{1}H NMR and ^{19}F NMR spectrum that integrate at least two pairs of signals corresponding to the two diastereomers present in the sample.

(*S*)-2,2-Difluoro-*N*-methyl-4-phenyl-*N*-((*R*)-3-phenyl-3-(*o*-tolyloxy)propyl)-4-(4,4,5,5-tetramethyl-1,3,2-dioxaborolan-2-yl)butanamide (6d).

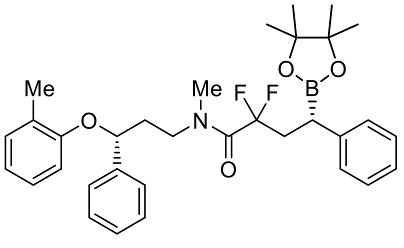

Colorless oil (93.4 mg, 83% yield, 96:4 dr); **^{1}H NMR** (400 MHz, $CDCl_3$) δ 7.45 – 7.32 (m, 4H), 7.33 – 7.25 (m, 5H), 7.24 – 7.12 (m, 2H), 6.99 (t, *J* = 7.8 Hz, 1H), 6.83 (q, *J* = 6.8 Hz, 1H), 6.61 (dd, *J* =

14.1, 8.2 Hz, 1H), 5.29 – 5.11 (m, 1H), 3.88 – 3.69 (m, 1H), 3.61 (qdd, *J* = 13.5, 8.7, 5.6 Hz, 1H), 3.16 (s, 1.5H), 2.98 (s, 1.5H), 2.95 – 2.83 (m, 1H), 2.74 (dt, *J* = 8.2, 3.8 Hz, 1H), 2.58 – 2.43 (m, 1H), 2.39 (s, 3H), 2.24 (ttd, *J* = 18.0, 9.4, 7.8, 4.8 Hz, 2H), 1.22 (s, 6H), 1.20 (s, 6H); **^{13}C {^{1}H} NMR** (101 MHz, $CDCl_3$) δ 163.4 (t, *J* = 29.3 Hz), 163.2 (t, *J* = 29.1 Hz), 155.7, 155.6, 141.8, 141.3, 141.1, 130.7, 130.6, 128.7, 128.6, 128.4, 128.1, 128.1, 127.7, 127.6, 126.9, 126.9, 126.6, 126.5, 125.7, 125.6, 125.5, 120.5, 120.4, 119.5 (t, *J* = 254.7 Hz), 119.3 (t, *J* = 254.1 Hz), 112.7, 112.5, 83.5, 47.1, 46.9, 37.9, 37.4 (q, *J* = 23.2 Hz), 35.7, 35.6 (t, *J* = 6.9 Hz), 24.5, 24.5, 16.5; **^{19}F NMR** (377 MHz, $CDCl_3$) δ -97.70 – -100.73 (m, 2F), **^{11}B NMR** (128 MHz, $CDCl_3$) δ 33.38; **HRMS (EI)** m/z calcd for $C_{33}H_{40}BF_2NO_4$ $[M+Na]^+$: 586.2916; Found: 586.2922.
Optical rotation: $[\alpha]_D^{25}$: 15.4 (*c* = 1.00, $CHCl_3$).
Diastereomeric ratios were determined by crude ^{1}H NMR and ^{19}F NMR spectrum that integrate at least two pairs of signals corresponding to the two diastereomers present in the sample.

Ethyl (*S*)-2,2-difluoro-4-(4-(((*S*)-2-(4-isobutylphenyl)propanoyl)oxy)phenyl)-4-(4,4,5,5-tetramethyl-1,3,2-dioxaborolan-2-yl)butanoate (6e).

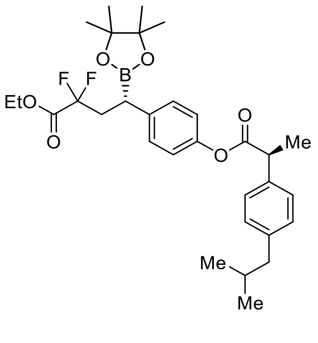

Colorless oil (84.8 mg, 76% yield, 98:2 dr); **^{1}H NMR** (400 MHz, $CDCl_3$) δ 7.31 (d, *J* = 8.0 Hz, 2H), 7.23 – 7.13 (m, 4H), 6.97 – 6.87 (m, 2H), 4.23 – 4.06 (m, 2H), 3.93 (q, *J* = 7.1 Hz, 1H), 2.76 – 2.57 (m, 2H), 2.54 – 2.33 (m, 3H), 1.89 (dp, *J* = 12.8, 6.4 Hz, 1H), 1.61 (d, *J* = 7.1 Hz, 3H), 1.28 (t, *J* = 7.1 Hz, 3H), 1.20 (s, 6H), 1.18 (s, 6H), 0.94 (s, 3H), 0.93 (s, 3H); **^{13}C {^{1}H} NMR** (101 MHz, $CDCl_3$) δ 173.1, 164.0 (t, *J* = 32.7 Hz), 149.1, 140.7, 138.1, 137.3, 137.2, 129.4, 129.0, 127.1, 121.3, 115.9 (dd, *J* = 251.5, 249.5 Hz), 83.8, 62.7, 45.2, 45.0, 37.0 (t, *J* = 22.8 Hz), 30.1, 24.4, 24.4, 22.3, 18.4, 13.8; **^{19}F NMR** (377 MHz, $CDCl_3$) δ -102.68 (ddd, *J* = 259.1, 18.6, 9.8 Hz, 1F), -106.88 – -108.16 (m, 1F), **^{11}B NMR**

(128 MHz, $CDCl_3$) δ 33.53; **HRMS (EI)** m/z calcd for $C_{31}H_{41}BF_2O_6$ $[M+Na]^+$: 581.2862; Found: 581.2866.

Optical rotation: $[\alpha]_D^{25}$: -6.2 (*c* = 1.0, $CHCl_3$).

Diastereomeric ratios were determined by crude ^{1}H NMR and ^{19}F NMR spectrum that integrate at least two pairs of signals corresponding to the two diastereomers present in the sample.

(*S*)-2,2-difluoro-4-phenyl-4-(4,4,5,5-tetramethyl-1,3,2-dioxaborolan-2-yl)butyl 2-(4-(4-chlorobenzoyl)phenoxy)-2-methylpropanoate (**6f**).

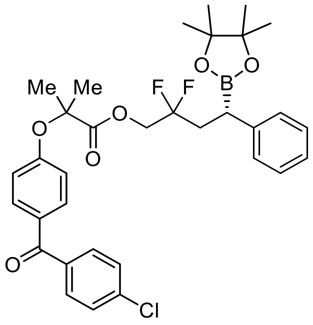

Colorless oil (49.6 mg, 81% yield, 97% ee); **^{1}H NMR** (400 MHz, $CDCl_3$) δ 7.66 – 7.58 (m, 4H), 7.41 – 7.35 (m, 2H), 7.19 – 7.12 (m, 2H), 7.10 – 7.02 (m, 3H), 6.81 – 6.74 (m, 2H), 4.21 (ddt, *J* = 24.0, 14.1, 12.2 Hz, 2H), 2.53 (dd, *J* = 9.9, 4.9 Hz, 1H), 2.41 (ddt, *J* = 23.8, 14.5, 9.0 Hz, 1H), 2.02 (ddt, *J* = 20.0, 14.5, 4.8 Hz, 1H), 1.51 (s, 6H), 1.08 (s, 6H), 1.06 (s, 6H); **^{13}C {^{1}H} NMR** (101 MHz, $CDCl_3$) δ 194.1, 172.6, 159.3, 141.1, 138.3, 136.3, 132.0, 131.1, 130.5, 128.6, 128.5, 127.9, 125.8, 121.2 (t, *J* = 243.3 Hz), 117.4, 83.7, 79.2, 64.5 (dd, *J* = 33.9, 31.8 Hz), 36.5 (t, *J* = 23.2 Hz), 25.5, 25.3, 24.4; **^{19}F NMR** (377 MHz, $CDCl_3$) δ -102.16 – -103.83 (m, 1F), -105.39 – -107.20 (m, 1F), **^{11}B NMR** (128 MHz, $CDCl_3$) δ 34.17; **HRMS (EI)** m/z calcd for $C_{33}H_{36}BClF_2O_6$ $[M+Na]^+$: 635.2159; Found: 635.2157.

Optical rotation: $[\alpha]_D^{25}$: 6.5 (*c* = 1.0, $CHCl_3$).

HPLC condition: Chiral column OD-H, n-Hexane/i-PrOH = 98:2, flow rate = 0.5 mL/min, wavelength = 220 nm, t_R = 30.1 min for the major isomer, t_R = 33.8 min for the minor isomer.

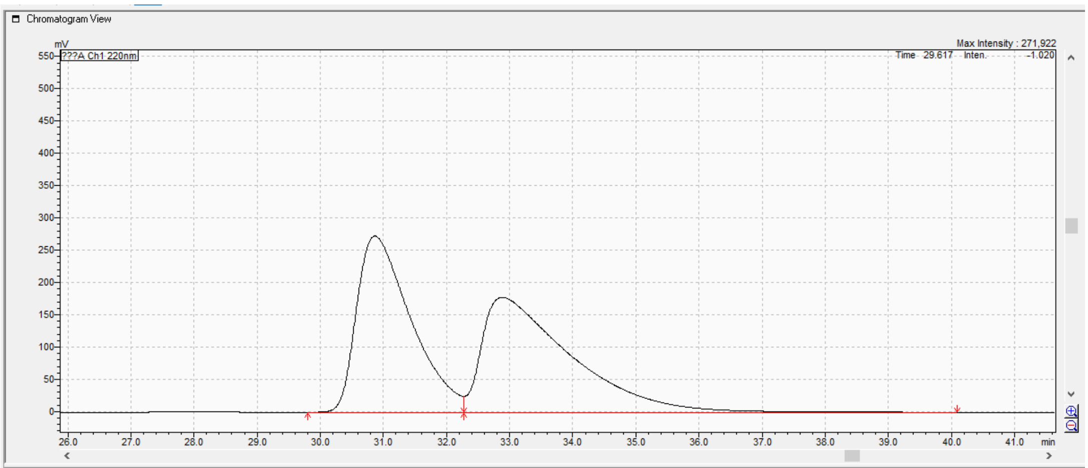

Results View - Peak Table

Peak Table | Compound | Group | Calibration Curve

Peak#	Ret. Time	Area	Height	Mark	Conc.	Unit	ID#	Name	Area%
1	30.866	16326147	272964	V	48.539				48.539
2	32.883	17308956	178026	SV	51.461				51.461
Total		33635103	450990		100.000				100.000

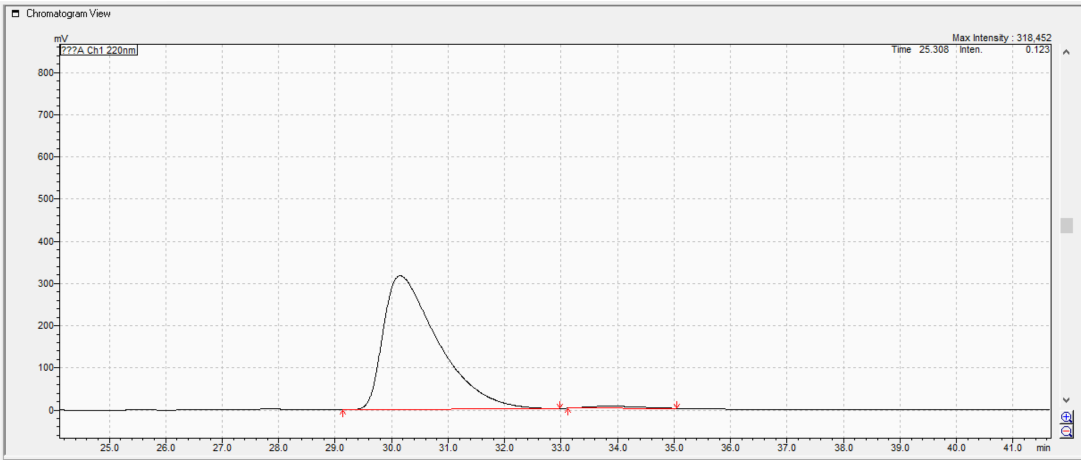

Results View - Peak Table

Peak Table | Compound | Group | Calibration Curve

Peak#	Ret. Time	Area	Height	Mark	Conc.	Unit	ID#	Name	Area%
1	30.150	21321303	316705	M	98.578				98.578
2	33.880	307545	4834	M	1.422				1.422
Total		21628848	321539		100.000				100.000

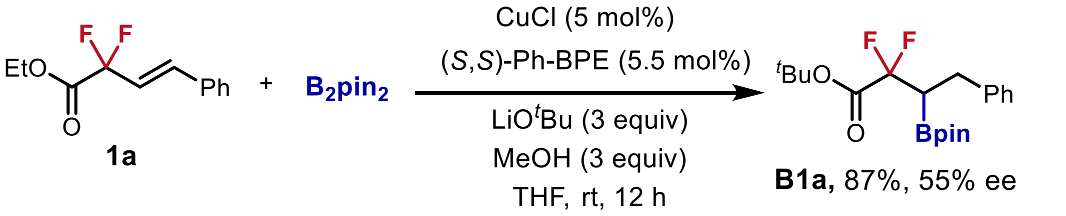

In an Ar-filled dry glovebox, CuCl (1.2 mg, 10 μmol), (*S*, *S*)-Ph-BPE (5.6 mg, 11 μmol), THF (1 mL), and a magnetic stirring bar were added to a 4 mL screw-capped vial and stirred for 5 mins before LiO^{t}Bu (48.0 mg, 0.6 mmol) and B_2pin_2 (76.2 mg, 0.3 mmol) were added in sequence. The resulting solution was stirred for another 10

min and alkene **1a** (45.2 mg, 0.200 mmol) and MeOH (12.8 mg, 0.4 mmol) were added. The vial was sealed with a cap containing a PTFE septum and removed from the dry box. The reaction mixture was stirred at room temperature for 24 h and the resulting solution was concentrated in vacuum and purified by flash column chromatography using EtOAc/n-Hexane (1:60) as eluent yielding product **B1a** as a colorless oil.

***tert*-butyl 2,2-difluoro-4-phenyl-3-(4,4,5,5-tetramethyl-1,3,2-dioxaborolan-2-yl)butanoate** (**B1a**).

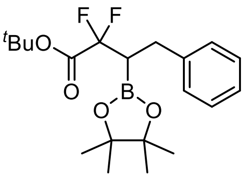

Colorless oil (66.5 mg, 87% yield, 55% ee); **^{1}H NMR** (400 MHz, $CDCl_3$) δ 7.31 – 7.24 (m, 4H), 7.20 (ddd, *J* = 8.5, 3.6, 2.3 Hz, 1H), 3.02 (dd, *J* = 13.6, 4.7 Hz, 1H), 2.89 (dd, *J* = 13.6, 11.2 Hz, 1H), 2.36 – 2.19 (m, 1H), 1.56 (s, 9H), 1.15 (s, 6H), 1.10 (s, 6H); **^{13}C {^{1}H} NMR** (101 MHz, $CDCl_3$) δ 163.2 (t, *J* = 32.7 Hz), 139.8, 129.0, 128.2, 126.2, 117.0 (t, *J* = 253.3 Hz), 84.2, 84.0, 29.8 (t, *J* = 5.3 Hz), 27.7, 24.6, 24.5; **^{19}F NMR** (377 MHz, $CDCl_3$) δ -103.14 (t, *J* = 18.4 Hz); **^{11}B NMR** (128 MHz, $CDCl_3$) δ 32.57; **HRMS (EI)** m/z calcd for $C_{20}H_{29}BF_2O_4$ $[M+Na]^+$: 405.2025; Found: 405.2037.
Optical rotation: $[\alpha]_D^{25}$: 25.8 (*c* = 1.00, $CHCl_3$).
HPLC condition (after oxidation with 2 equiv $NaBO_3$): Chiral column OD-H, n-Hexane/i-PrOH = 98:2, flow rate = 1.0 mL/min, wavelength = 220 nm, t_R = 8.4 min for the major isomer, t_R = 7.8 min for the minor isomer.

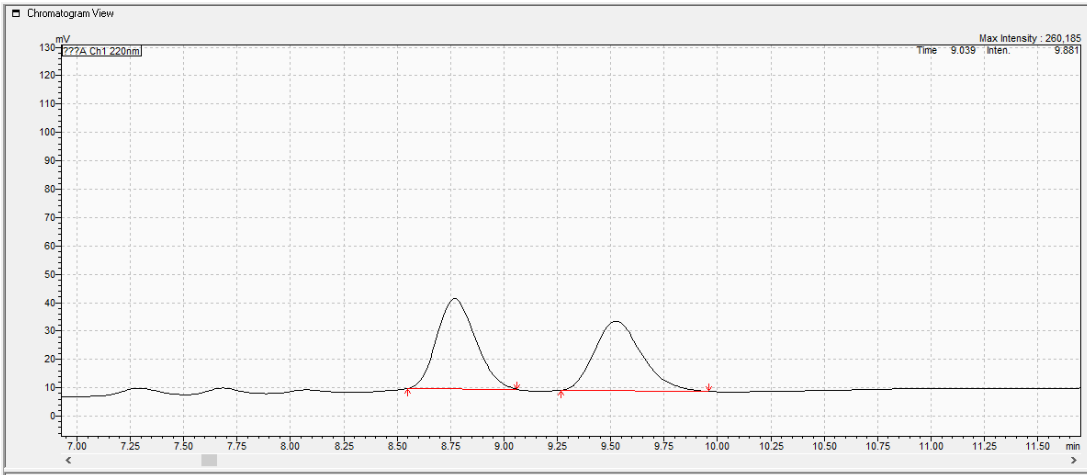

Results View - Peak Table

Peak Table | Compound | Group | Calibration Curve

Peak#	Ret. Time	Area	Height	Mark	Conc.	Unit	ID#	Name	Area%
1	8.769	401088	31762	M	51.679				51.679
2	9.523	375027	24455	M	48.321				48.321
Total		776115	56217		100.000				100.000

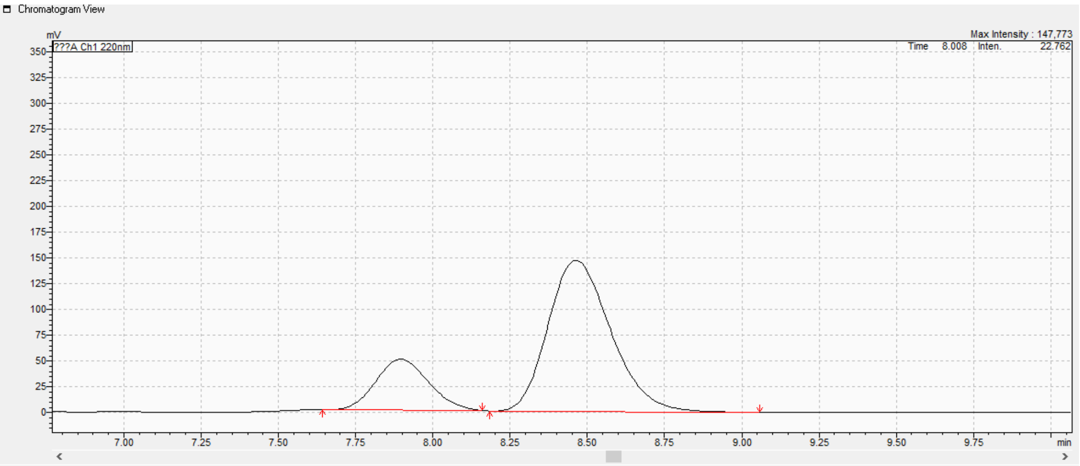

Results View - Peak Table

Peak Table | Compound | Group | Calibration Curve

Peak#	Ret. Time	Area	Height	Mark	Conc.	Unit	ID#	Name	Area%
1	7.895	589458	49275	M	22.158				22.158
2	8.463	2070794	146490	M	77.842				77.842
Total		2660251	195765		100.000				100.000

4. General procedure for gram-scale reactions and synthetic transformations

Gram Scale Reactions

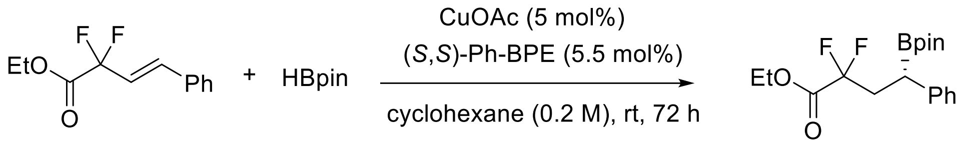

In an Ar-filled dry glovebox, CuOAc (22.6 mg, 0.185 mmol), (*S*, *S*)-Ph-BPE (103.0 mg, 0.203 mmol), cyclohexane (18.5 mL), and a magnetic stirring bar were added to a 50-mL single Schlenk bottle and stirred for 30 mins. The corresponding difluoroalkylated alkene **1a** (0.836 g, 3.70 mmol) were added and stirred for further 5 min, then HBpin (0.710 g, 5.55 mmol) was added. The bottle was sealed with a cap containing a PTFE septum and removed from the dry box. The reaction mixture was stirred at room temperature for 72 h and the resulting solution was concentrated in vacuum. The residue was purified by silica gel flash column chromatography with EtOAc/n-Hexane (1:40) as eluent yielding **2a** (0.81 g, 62% yield, 98% ee) as colorless oil.

HPLC condition: Chiral column OD-H, n-Hexane/^{i}PrOH = 99.5:0.5, flow rate = 0.5 mL/min, wavelength = 220 nm, t_R = 11.3 min for the major isomer, t_R = 10.4 min for the minor isomer.

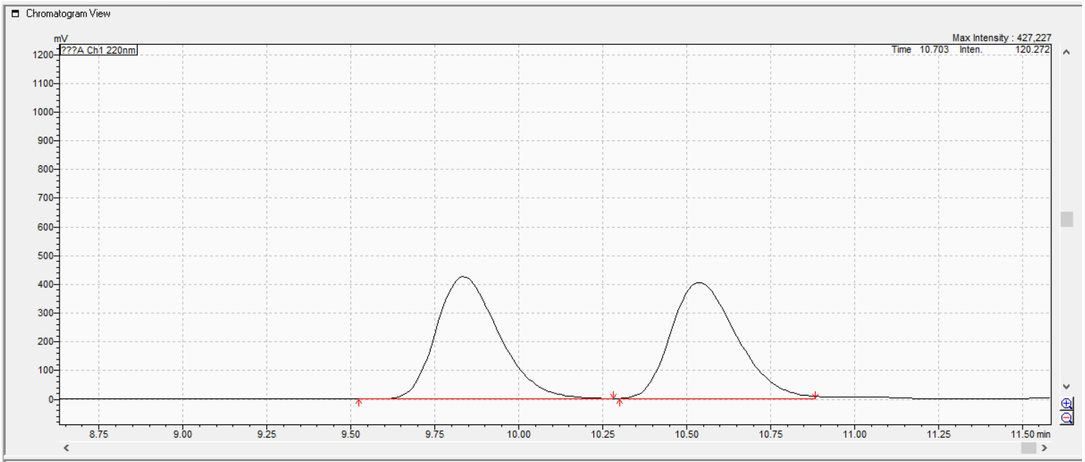

Results View - Peak Table

Peak Table | Compound | Group | Calibration Curve

Peak#	Ret. Time	Area	Height	Mark	Conc.	Unit	ID#	Name	Area%
1	9.835	5523873	425927		50.026				50.026
2	10.537	5518235	404800	M	49.974				49.974
Total		11042108	830727		100.000				100.000

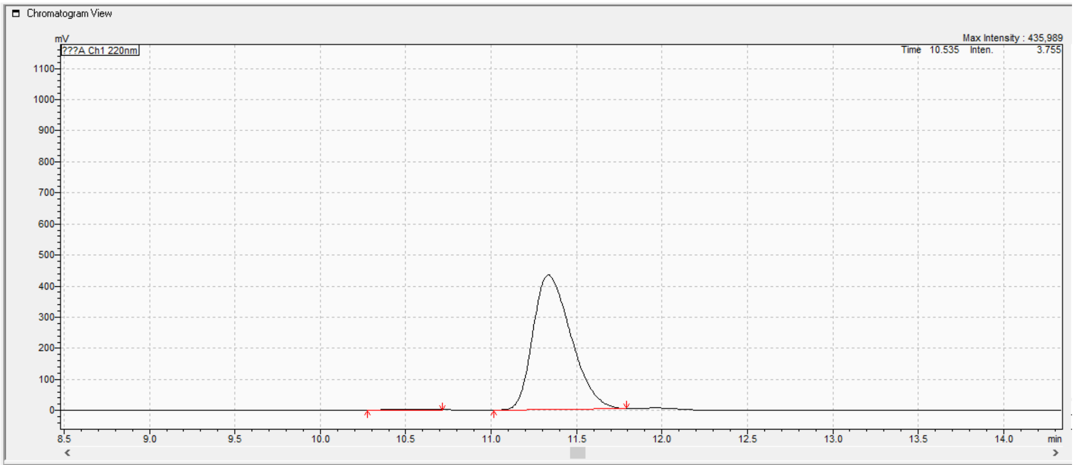

Results View - Peak Table

Peak Table | Compound | Group | Calibration Curve

Peak#	Ret. Time	Area	Height	Mark	Conc.	Unit	ID#	Name	Area%
1	10.483	80635	4249	M	1.189				1.189
2	11.335	6698282	432556	M	98.811				98.811
Total		6778916	436805		100.000				100.000

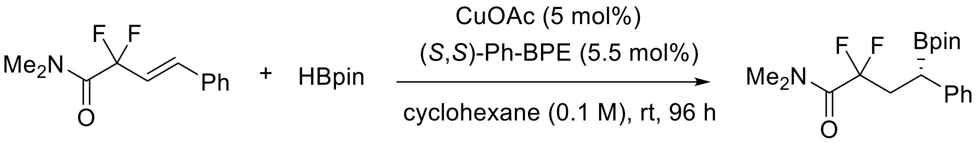

In an Ar-filled dry glovebox, CuOAc (23.5 mg, 0.193 mmol), (*S*, *S*)-Ph-BPE (107.1 mg, 0.212 mmol), cyclohexane (38.5 mL), and a magnetic stirring bar were added to a 100-mL single Schlenk bottle and stirred for 30 mins. The corresponding difluoroalkylated alkene **3b** (0.865 g, 3.85 mmol) were added and stirred for further 5 min, then HBpin (0.739 g, 5.55 mmol) was added. The bottle was sealed with a cap containing a PTFE septum and removed from the dry box. The reaction mixture was

stirred at room temperature for 72 h and the resulting solution was concentrated in vacuum. The residue was purified by silica gel flash column chromatography with EtOAc/n-Hexane (1:40) as eluent yielding **4b** (0.72 g, 53% yield, 98% ee) as white solid.

HPLC condition: Chiral column OD-H, n-Hexane/i-PrOH = 95:5, flow rate = 1.0 mL/min, wavelength = 210 nm, t_R = 5.4 min for the major isomer, t_R = 5.0 min for the minor isomer.

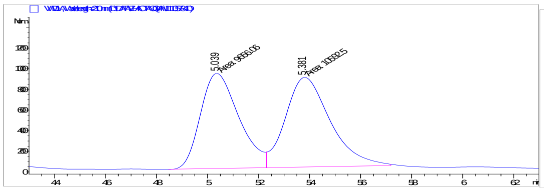

Peak#	Time	Area	Height	Width	Symmetry	Area%
1	5.039	9656.1	923.2	0.1743	0.8	47.688
2	5.381	10592.5	870.7	0.2027	0.645	52.312

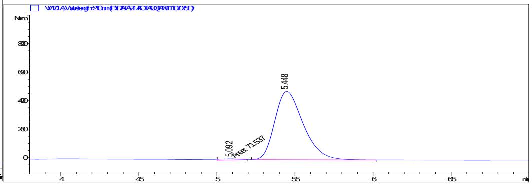

Peak#	Time	Area	Height	Width	Symmetry	Area%
1	5.092	71.5	7.7	0.1547	1.37	1.163
2	5.448	6079.4	479.3	0.1946	0.682	98.837

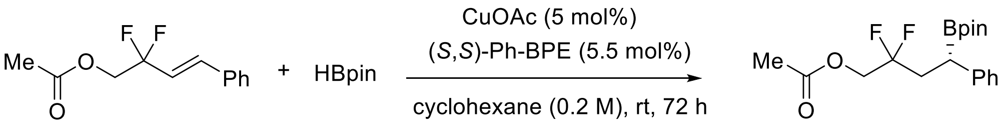

In an Ar-filled dry glovebox, CuOAc (24.0 mg, 0.197 mmol), (*S*, *S*)-Ph-BPE (109.7 mg, 0.217 mmol), cyclohexane (19.7 mL), and a magnetic stirring bar were added to a 50-mL single Schlenk bottle and stirred for 30 mins. The corresponding difluoroalkylated alkene **3i** (0.890 g, 3.94 mmol) were added and stirred for further 5 min, then HBpin (0.756 g, 5.91 mmol) was added. The bottle was sealed with a cap containing a PTFE septum and removed from the dry box. The reaction mixture was stirred at room temperature for 72 h and the resulting solution was concentrated in vacuum. The residue was purified by silica gel flash column chromatography with EtOAc/n-Hexane (1:40) as eluent yielding **4i** (1.13 g, 81% yield, 98% ee) as colorless oil.

HPLC condition: Chiral column OD-H, n-Hexane/i-PrOH = 99.5:0.5, flow rate = 0.5 mL/min, wavelength = 220 nm, t_R = 13.8 min for the major isomer, t_R = 15.6 min for the minor isomer.

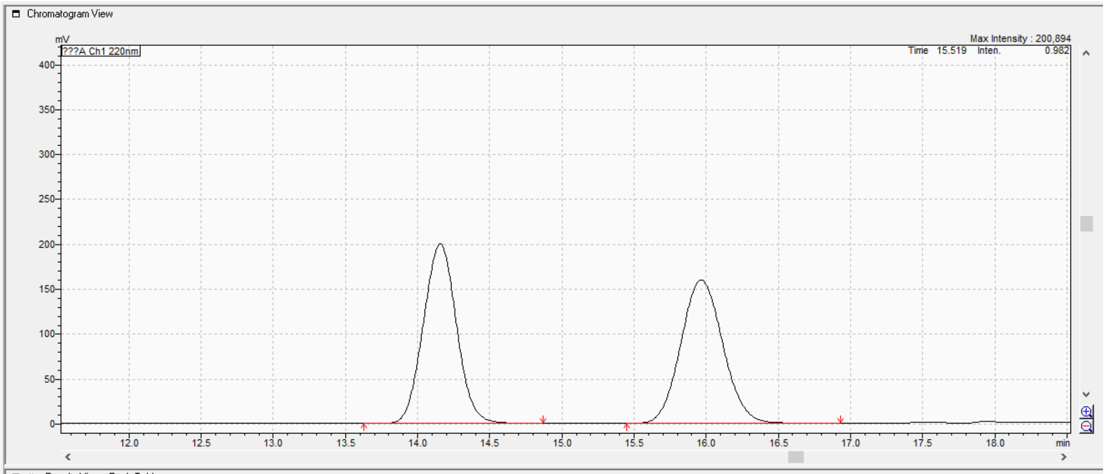

Results View - Peak Table

Peak Table | Compound | Group | Calibration Curve

Peak#	Ret. Time	Area	Height	Mark	Conc.	Unit	ID#	Name	Area%
1	14.157	3202689	200109	M	49.753				49.753
2	15.966	3234511	159471	M	50.247				50.247
Total		6437200	359581		100.000				100.000

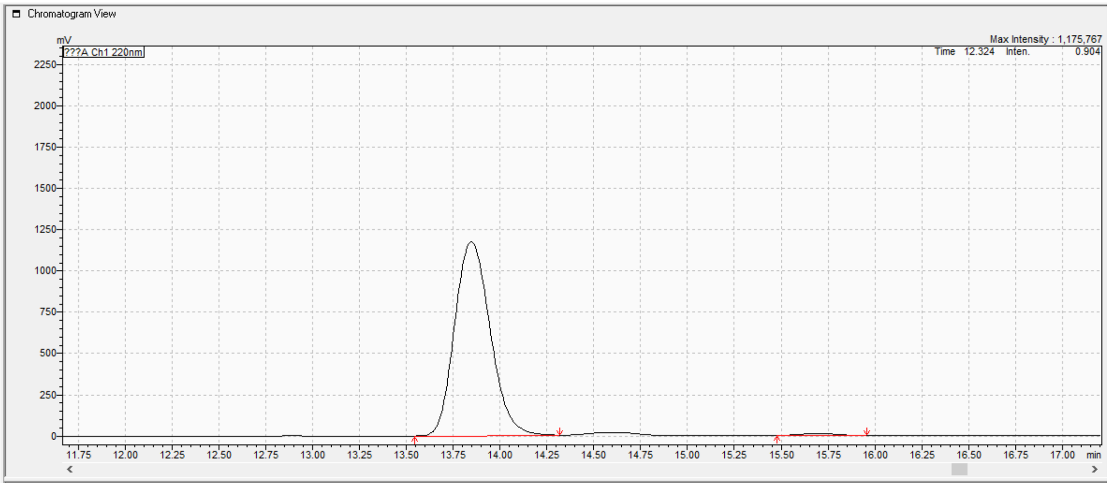

Results View - Peak Table

Peak Table | Compound | Group | Calibration Curve

Peak#	Ret. Time	Area	Height	Mark	Conc.	Unit	ID#	Name	Area%
1	13.846	15509470	1172875	M	98.858				98.858
2	15.697	179161	12825	M	1.142				1.142
Total		15688632	1185700		100.000				100.000

Transformations of difluoroalkylated boronate Products

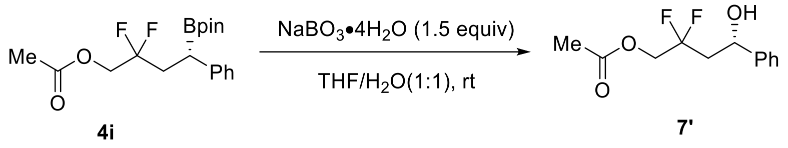

Prepared according to a previous reported method,[4] to a solution of boronate **4i** (70.8 mg, 0.2 mmol, ee = 98%) in THF/H_2O (1:1; 2.0 mL) was added $NaBO_3 \cdot 4H_2O$ (46.2 mg). The reaction mixture was stirred vigorously at room temperature for 4 h. After the reaction was complete, the mixture was diluted with H_2O and extracted with EtOAc (4 mL). The combined organic phases were dried with anhydrous Na_2SO_4 and concentrated under reduced pressure. The residue was purified by flash column chromatography using EtOAc/n-Hexane (1:10) as eluent to afford the product **7'** as a colorless oil.

(*S*)-2,2-Difluoro-4-hydroxy-4-phenylbutyl acetate (7')

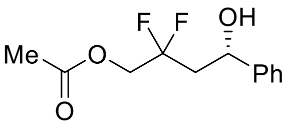

Colorless oil (38.1 mg, 78% yield, 98% ee); **^{1}H NMR** (400 MHz, $CDCl_3$) δ 7.41 – 7.31 (m, 5H), 6.13 (dd, *J* = 9.1, 3.9 Hz, 1H), 3.78 (td, *J* = 12.7, 7.0 Hz, 2H), 2.68 (dtd, *J* = 16.8, 15.1, 9.1 Hz, 1H), 2.43 (dtd, *J* = 18.2, 14.9, 3.9 Hz, 1H), 2.10 (s, 3H); **^{13}C {^{1}H} NMR** (101 MHz, $CDCl_3$) δ 169.9, 139.7, 128.7, 128.4, 126.3, 121.7 (t, *J* = 243.6 Hz), 70.3 (t, *J* = 5.1 Hz), 64.2 (t, *J* = 31.8 Hz), 39.8 (t, *J* = 23.9 Hz), 21.1; **^{19}F NMR** (377 MHz, $CDCl_3$) δ -104.94 – -106.15 (m, 1F), -106.23 – -107.40 (m, 1F); **HRMS (EI)** m/z calcd for $C_{12}H_{14}F_2O_3$ [M+Na]$^+$: 267.0809; Found: 267.0819.

Optical rotation: $[\alpha]_D^{25}$: -51.1 (*c* = 0.500, $CHCl_3$).

HPLC condition: Chiral column OD-H, n-Hexane/i-PrOH = 95:5, flow rate = 1.0 mL/min, wavelength = 220 nm, t_R = 19.6 min for the major isomer, t_R = 13.9 min for the minor isomer.

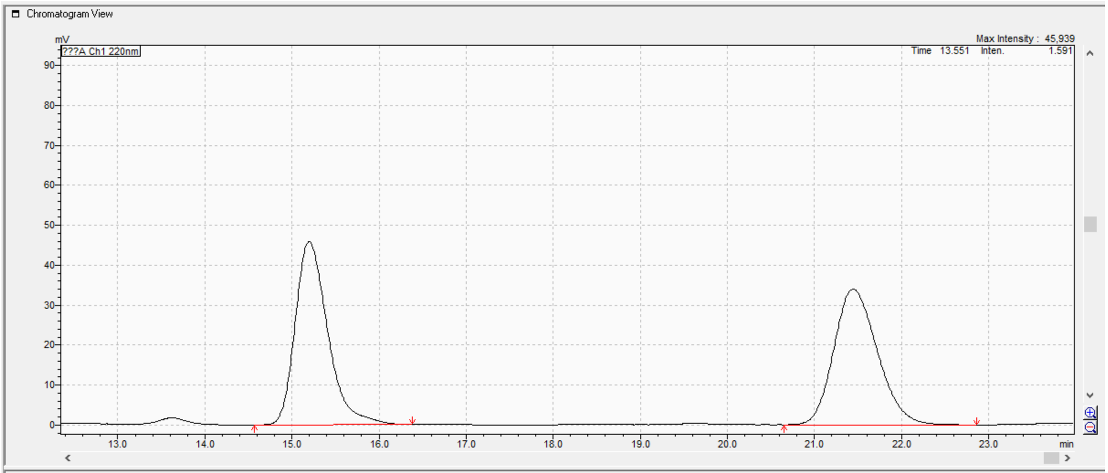

Results View - Peak Table

Peak Table | Compound | Group | Calibration Curve

Peak#	Ret. Time	Area	Height	Mark	Conc.	Unit	ID#	Name	Area%
1	15.198	1190037	45872	S	50.189				50.189
2	21.445	1181054	33961	S	49.811				49.811
Total		2371091	79833		100.000				100.000

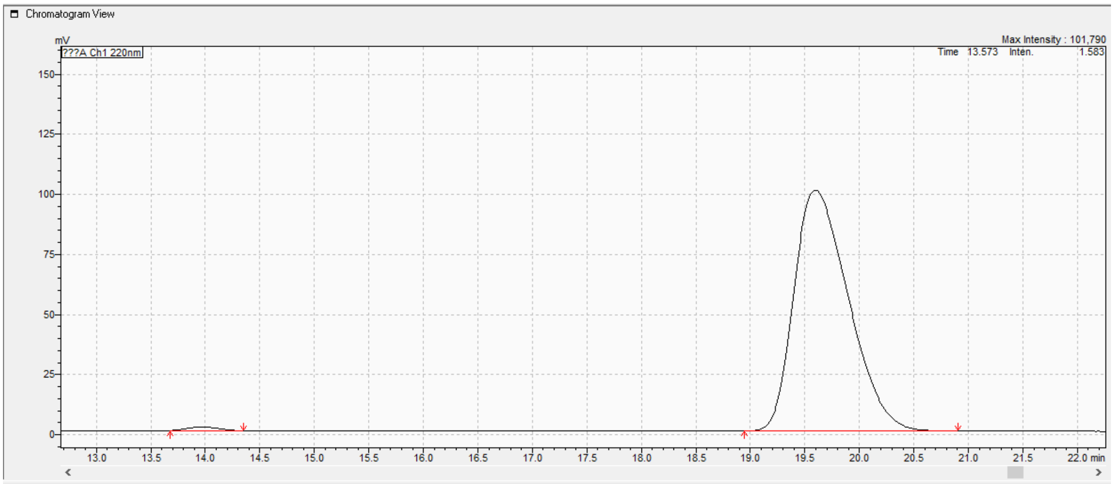

Results View - Peak Table

Peak Table | Compound | Group | Calibration Curve

Peak#	Ret. Time	Area	Height	Mark	Conc.	Unit	ID#	Name	Area%
1	13.961	29957	1515	M	0.850				0.850
2	19.596	3493587	100323	M	99.150				99.150
Total		3523544	101839		100.000				100.000

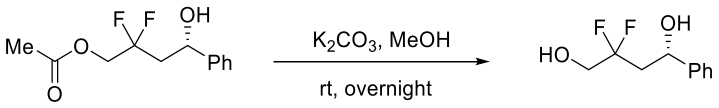

7' **7**

Prepared according to a previous reported method,[5] K_2CO_3 (55.2 mg, 0.4 mmol) was added to a solution of **7’** (48.8 mg, 0.2 mmol, 98% ee) in MeOH (2 mL). The mixture was stirred at room temperature overnight, then a 2 M solution of HCl (2.0 mL) was added. The resulting solution was extracted with EA, washed with brine (10 mL), and dried over anhydrous Na_2SO_4. After filtration and evaporation of the solvent, the

residue was purified by flash column chromatography with EtOAc/n-Hexane (1:3) to give the title compound **7** as colorless oil.

(*S*)-3,3-Difluoro-1-phenylbutane-1,4-diol (7)

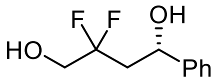

Colorless oil (39.6 mg, 98% yield, 98% ee); **^{1}H NMR** (400 MHz, $CDCl_3$) δ 7.43 – 7.30 (m, 5H), 5.01 (dd, *J* = 10.3, 1.8 Hz, 1H), 3.98 – 3.71 (m, 2H), 3.23 – 3.01 (br, 2H), 2.49 (ddt, *J* = 25.9, 15.2, 9.9 Hz, 1H), 2.25 (q, *J* = 14.5, 14.1 Hz, 1H); **^{13}C {^{1}H} NMR** (101 MHz, $CDCl_3$) δ 143.0, 128.8, 128.2, 125.5, 122.5 (t, *J* = 243.4 Hz), 69.5 (dd, *J* = 10.3, 2.2 Hz), 64.3 (dd, *J* = 34.2, 32.3 Hz), 43.4 (t, *J* = 24.5 Hz); **^{19}F NMR** (377 MHz, $CDCl_3$) δ -98.38 – -100.07 (m, 1F), -106.37 – -108.11 (m, 1F); **HRMS (EI)** m/z calcd for $C_{10}H_{12}F_2O_2$ $[M+Na]^+$: 225.0703; Found: 225.0705.

Optical rotation: $[\alpha]_D^{25}$: -51.6 (*c* = 1.00, $CHCl_3$).

HPLC condition: Chiral column OJ-H, n-Hexane/i-PrOH = 95:5, flow rate = 1.0 mL/min, wavelength = 220 nm, t_R = 23.8 min for the major isomer, t_R = 22.9 min for the minor isomer.

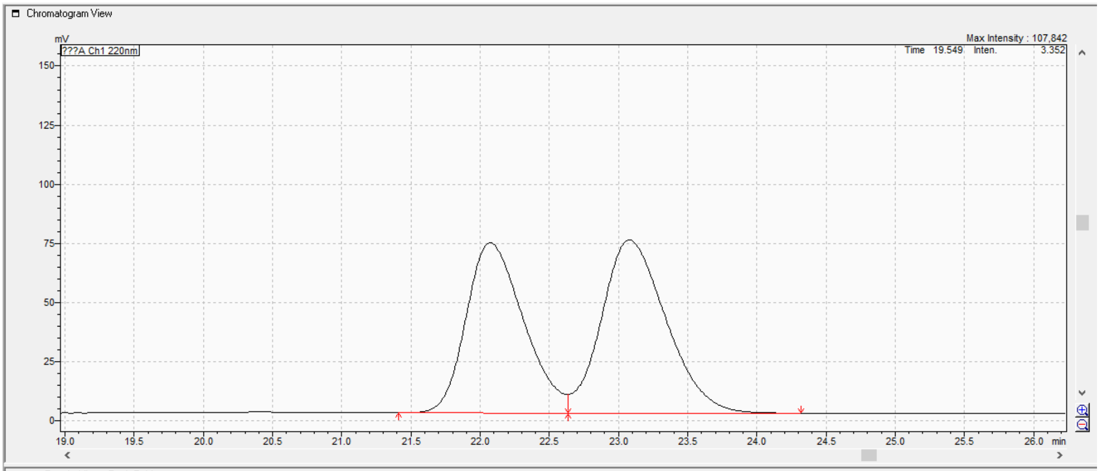

Results View - Peak Table

Peak#	Ret. Time	Area	Height	Mark	Conc.	Unit	ID#	Name	Area%
1	22.072	2078853	71970	V	46.688				46.688
2	23.079	2373756	73189	V	53.312				53.312
Total		4452609	145158		100.000				100.000

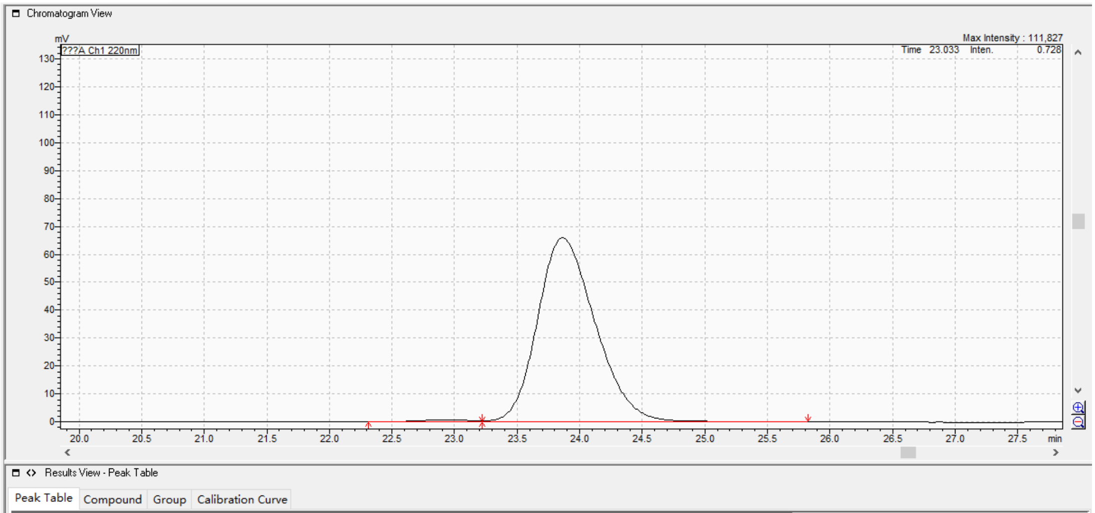

Peak#	Ret. Time	Area	Height	Mark	Conc.	Unit	ID#	Name	Area%
1	22.930	20116	739	V	0.943				0.943
2	23.863	2113485	65820	SV	99.057				99.057
Total		2133601	66559		100.000				100.000

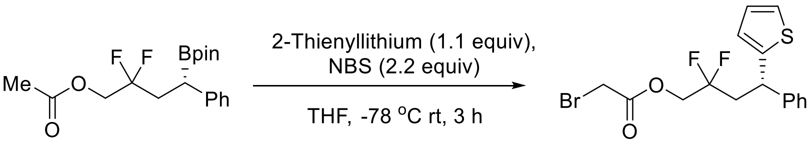

Prepared according to a previous reported method:[6] In an argon-filled glovebox, to a solution of **4i** (70.8 mg, 0.2 mmol, ee = 98%) in anhydrous THF (2 mL) was added 2-thienyllithium (0.22 mmol, 0.22 mL of a 1.0 M solution in THF) dropwise at -78 ˚C. The mixture was stirred for 2 h before the solution was warmed to room temperature. *N*-Bromosuccinimide (0.44 mmol, 0.1 M in MeOH) was added dropwise. The reaction was stirred at room temperature for another 1 h, after which, a saturated solution of $Na_2S_2O_3$ (5 mL) was added and the mixture was diluted with Et_2O (5 mL). The organic layer was separated and the aqueous layer was extracted with Et_2O. The combined organic layers were washed with brine and dried over Na_2SO_4. The solvent was removed under reduced pressure and the crude product was purified by flash column chromatography with EtOAc/n-Hexane (1:40) as eluent to afford the desired product **8** as colorless oil.

(*S*)-2,2-Difluoro-4-phenyl-4-(thiophen-2-yl)butyl 2-bromoacetate (8)

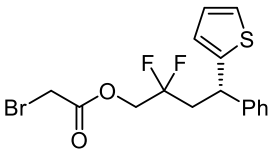

Colorless oil (56.6 mg, 73% yield, 98% ee); **^{1}H NMR** (400 MHz, $CDCl_3$) δ 7.25 (q, *J* = 2.9 Hz, 4H), 7.20 – 7.14 (m, 1H), 7.12 – 7.06 (m, 1H), 6.86 – 6.82 (m, 1H), 6.80 (dt, *J* = 3.5, 1.1 Hz, 1H), 4.47 (t, *J* = 7.2 Hz, 1H), 4.11 – 3.86 (m, 2H), 3.76 (s, 2H), 2.84 – 2.62 (m, 2H); **^{13}C {^{1}H} NMR** (101 MHz, $CDCl_3$) δ 165.9, 147.6, 142.9, 128.8, 127.5, 127.2, 126.7, 124.2, 124.2, 120.5 (t, *J* = 243.8 Hz), 64.8 (dd, *J* = 34.0 Hz, 33.0 Hz), 41.2 (t, *J* = 23.6 Hz), 40.7 (t, *J* = 4.9 Hz), 24.8; **^{19}F NMR** (377 MHz, $CDCl_3$) δ-101.07 – -102.13 (m, 1F), -102.61 – -103.54 (m, 1F); **HRMS (EI)** m/z calcd for $C_{16}H_{15}BrF_2O_2S$ $[M+Na]^+$: 410.9842; Found: 410.9844.

Optical rotation: $[\alpha]_D^{25}$: 3.7 (*c* = 0.50, $CHCl_3$).

HPLC condition: Chiral column OD-H, n-Hexane/i-PrOH = 99:1, flow rate = 1.0 mL/min, wavelength = 220 nm, t_R = 24.2 min for the major isomer, t_R = 32.9 min for the minor isomer.

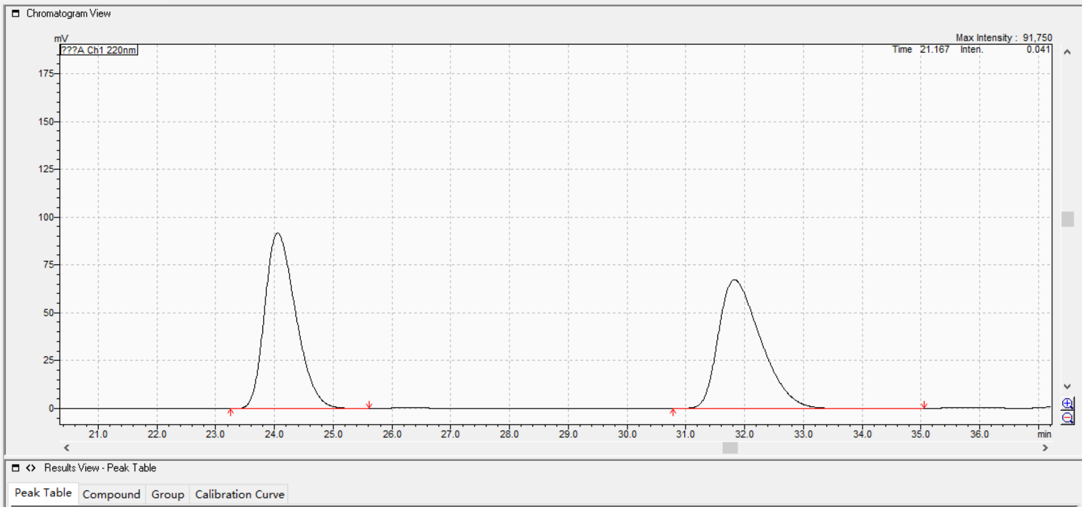

Peak#	Ret. Time	Area	Height	Mark	Conc.	Unit	ID#	Name	Area%
1	24.054	3380610	91902	SV	50.196				50.196
2	31.828	3354270	67239	S	49.804				49.804
Total		6734880	159141		100.000				100.000

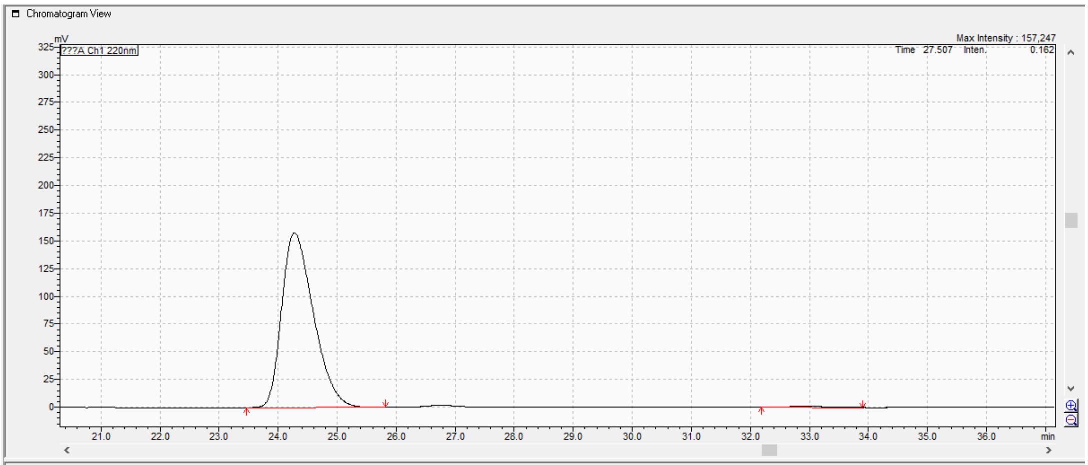

Results View - Peak Table

Peak Table | Compound | Group | Calibration Curve

Peak#	Ret. Time	Area	Height	Mark	Conc.	Unit	ID#	Name	Area%
1	24.276	5901473	157539		99.111				99.111
2	32.905	52943	1159	V	0.889				0.889
Total		5954417	158698		100.000				100.000

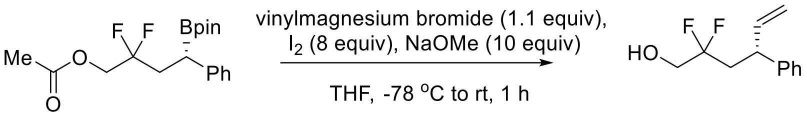

4i **9**

Prepared according to a previous reported method,[7] to a stirred solution of **4i** (70.8 mg, 0.2 mmol, ee = 98%) in dry THF (2 mL) at -78 °C, vinylmagnesium bromide (0.22 mL of a 1.0 M solution; 0.22 mmol) was added dropwise to the reaction mixture. The resulting solution was stirred at -78 °C for 5 minutes and then was stirred at room temperature for another 1 hour. The reaction mixture was re-cool to -78 °C and a solution of I_2 (406 mg, 1.60 mmol, 8.00 equivalents) in methanol (4.0 mL) was added dropwise. After the reaction mixture was stirred at the same temperature for further 30 minutes, a solution of sodium methoxide (108 mg, 2.00 mmol, 10.0 equivalents) in methanol (4.0 mL) was added dropwise. The reaction mixture was warmed up to room temperature and stirred for a total of 1 hour. The solvent was removed under reduced pressure and a 10 mL saturated solution of $Na_2S_2O_3$ and 10 mL of EtOAc were added to the mixture. The organic layer was separated and the aqueous layer was extracted with EtOAc. The combined organic layers were washed with brine and dried over Na_2SO_4. After the removal of the solvent, the residue was purified by flash

chromatography over silica gel with EtOAc/n-Hexane (1:10) to afford the desired product as a colorless oil.

(*R*)-2,2-Difluoro-4-phenylhex-5-en-1-ol (9)

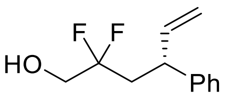

Colorless oil (33.5 mg, 79% yield, 97% ee); **^{1}H NMR** (400 MHz, $CDCl_3$) δ 7.39 – 7.31 (m, 2H), 7.28 – 7.21 (m, 3H), 6.04 (ddd, *J* = 17.4, 10.0, 7.4 Hz, 1H), 5.09 (ddt, *J* = 14.2, 4.1, 1.3 Hz, 2H), 3.81 – 3.48 (m, 3H), 2.45 (tdd, *J* = 16.7, 7.1, 2.0 Hz, 2H); **^{13}C {^{1}H} NMR** (101 MHz, $CDCl_3$) δ 143.1, 141.3, 128.8, 127.5, 126.8, 122.9 (t, *J* = 242.9 Hz), 114.8, 64.2 (dd, *J* = 32.4, 31.2 Hz), 43.7 (dd, *J* = 4.9, 4.2 Hz), 38.7 (t, *J* = 23.5 Hz); **^{19}F NMR** (377 MHz, $CDCl_3$) δ -103.32 – -104.76 (m, 1F), -105.37 – -106.76 (m, 1F); **HRMS (EI)** m/z calcd for $C_{12}H_{14}F_2O$ $[M+H]^+$: 213.1091; Found: 213.1097.

Optical rotation: $[\alpha]_D^{25}$: -9.2 (*c* = 1.0, $CHCl_3$).

HPLC condition: Chiral column OJ-H, n-Hexane/i-PrOH = 98:2, flow rate = 1.0 mL/min, wavelength = 220 nm, t_R = 37.2 min for the major isomer, t_R = 33.4 min for the minor isomer.

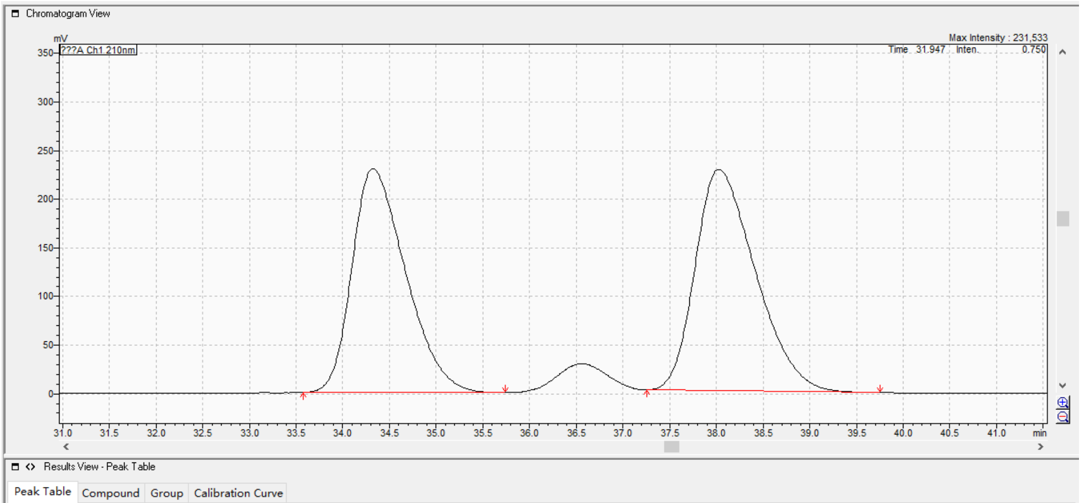

Peak#	Ret. Time	Area	Height	Mark	Conc.	Unit	ID#	Name	Area%
1	34.323	9212918	230354	M	47.370				47.370
2	38.025	10235797	227823	M	52.630				52.630
Total		19448716	458177		100.000				100.000

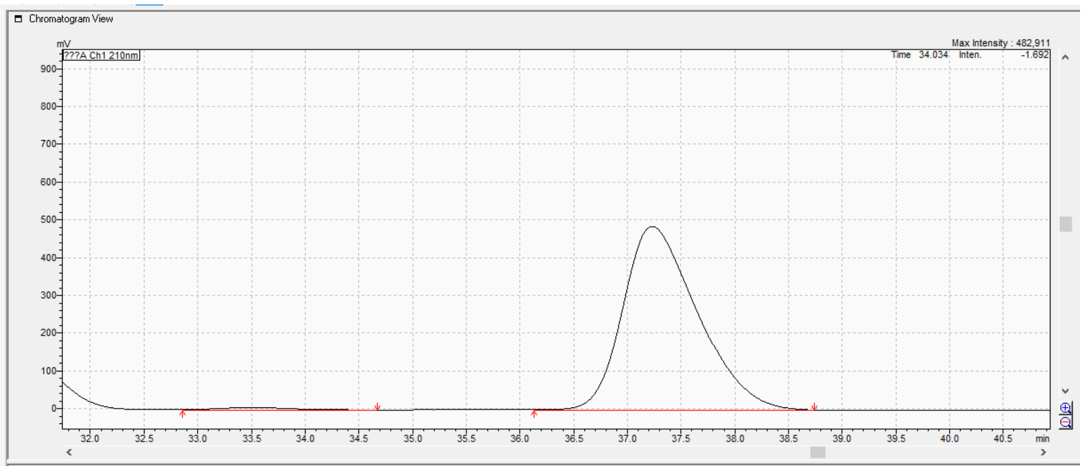

Peak#	Ret. Time	Area	Height	Mark	Conc.	Unit	ID#	Name	Area%
1	33.475	323308	6929	M	1.357				1.357
2	37.231	23501752	486372	M	98.643				98.643
Total		23825060	493301		100.000				100.000

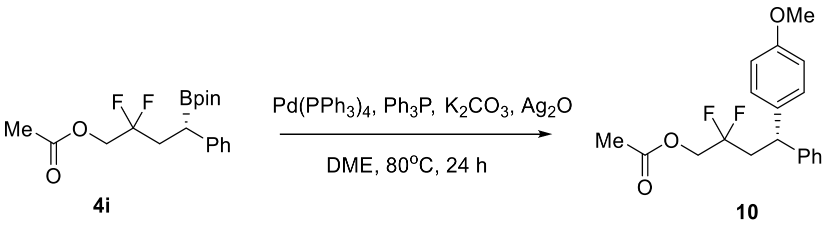

Prepared according to a previous reported method,[8] in an Ar-filled glovebox, 4-iodoanisole (70.2 mg, 0.3 mmol), **4i** (70.8 mg, 0.2 mmol, ee = 98%), Ag_2O (46.2 mg, 0.2 mmol), K_2CO_3 (27.6 mg, 0.2 mmol), $Pd(PPh_3)_4$ (18.5 mg, 0.016 mmol) and Ph_3P (16.8 mg, 0.064 mmol) were weighed into a dried vial and taken up in DME (3 mL). The reaction vessel was sealed and the contents stirred at 80 °C for 24 h. The mixture was cooled and filtered through Celite. The desired product **10** was isolated as a clear oil in 93% yield after column chromatography over silica gel with EtOAc/n-Hexane (1:20).

(*S*)-2,2-Difluoro-4-(4-methoxyphenyl)-4-phenylbutyl acetate (10)

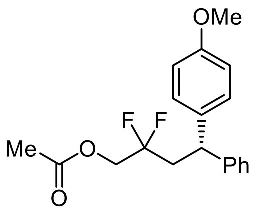

Colorless oil (62.1 mg, 93% yield, 90% ee); **^{1}H NMR** (400 MHz, $CDCl_3$) δ 7.26 – 7.14 (m, 4H), 7.11 (dq, *J* = 9.2, 2.6, 2.1 Hz, 3H), 6.79 – 6.73 (m, 2H), 4.18 (t, *J* = 7.2 Hz, 1H), 3.93 (t, *J* = 13.0 Hz, 2H), 3.69 (s, 3H), 2.66 (td, *J* = 16.1, 7.2 Hz, 2H),

1.98 (s, 3H); **^{13}C {^{1}H} NMR** (101 MHz, $CDCl_3$) δ 169.6, 158.2, 144.0, 135.7, 128.6, 128.5, 127.4, 126.6, 121.2 (t, J = 243.0 Hz), 114.0, 63.7 (t, J = 33.0 Hz), 55.2, 44.2 (t, J = 4.5 Hz), 39.8 (t, J = 23.3 Hz), 20.5; **^{19}F NMR** (377 MHz, $CDCl_3$) δ -102.51 (m, 2F); **HRMS (EI)** m/z calcd for $C_{19}H_{20}F_2O_3$ $[M+H]^+$: 335.1459; Found: 335.1465.

Optical rotation: $[\alpha]_D^{25}$: -21.5 (c = 1.00, $CHCl_3$).

HPLC condition: Chiral column AS-H, n-Hexane/i-PrOH = 99:1, flow rate = 1.0 mL/min, wavelength = 220 nm, t_R = 10.2 min for the major isomer, t_R = 9.1 min for the minor isomer.

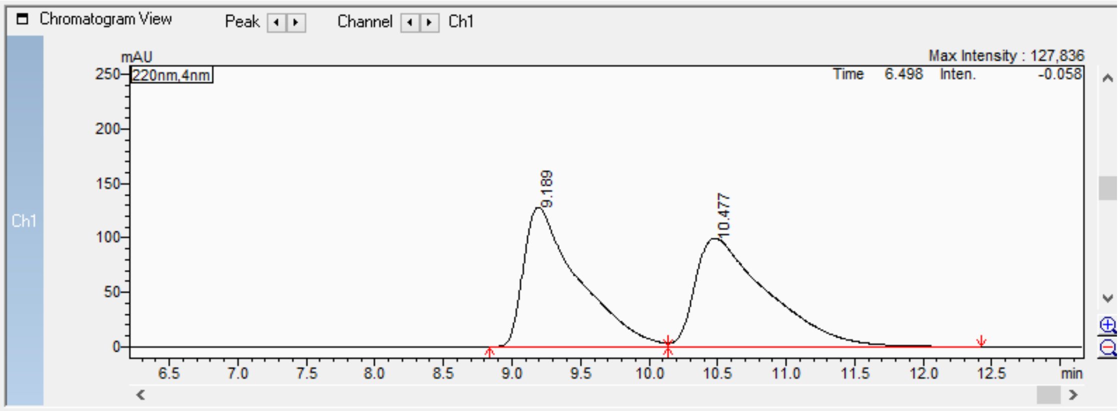

Results View - Peak Table

Peak Table | Compound | Group | Calibration Curve

Peak#	Ret. Time	Peak Start	Peak End	Area	Height	Mark	Conc.	Area%
1	9.189	8.837	10.139	3575978	127894		0.00000	49.404
2	10.477	10.139	12.421	3662236	99903	V	0.00000	50.596
Total				7238214	227798		0.00000	100.000

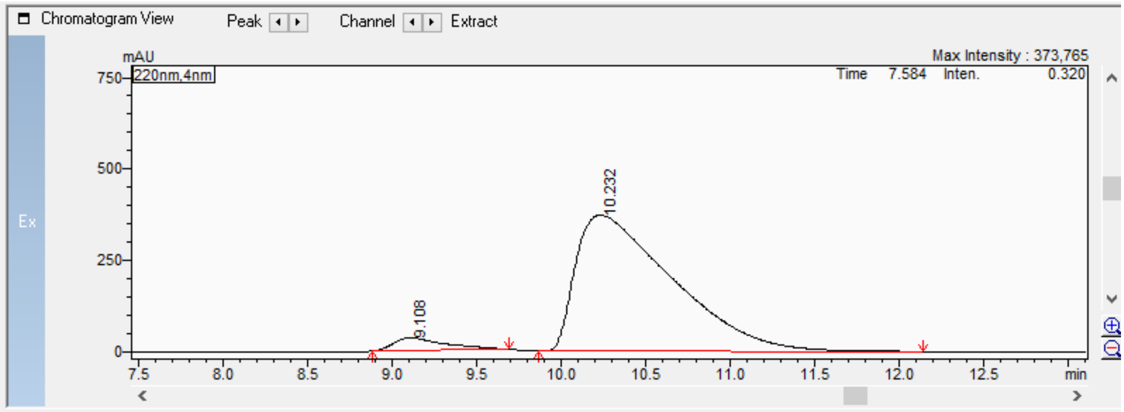

Results View - Peak Table

Peak Table | Compound | Group | Calibration Curve

Peak#	Ret. Time	Peak Start	Peak End	Area	Height	Mark	Conc.	Area%
1	9.108	8.885	9.696	749464	34689	M	4.82957	4.830
2	10.232	9.872	12.144	14768768	371345	M	95.17043	95.170
Total				15518232	406033		100.00000	100.000

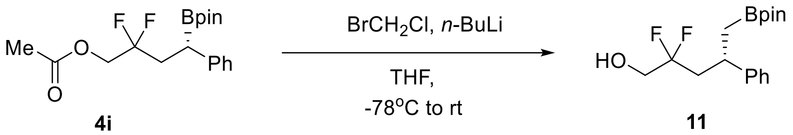

Prepared according to a previous reported method.[9] To an 8 mL vial containing a stirring bar were added **4i** (70.8 mg, 0.2 mmol, ee = 98%), distilled bromochloromethane (52.0 mg, 0.401 mmol), and dry THF (2.0 mL). The vial was sealed with a septum and cooled to –78 °C, at which time n-BuLi (0.12 mL, 0.24 mmol, 2.0 M in cyclohexane) was slowly added. The resulting mixture was stirred for 5 min at –78 °C and then warmed to room temperature. After stirred at room temperature for 4 h, the reaction was quenched with a saturated NH_4Cl solution (10 mL), and the resulting mixture was extracted with Et_2O (10 mL x 3). The combined organic layers were washed with brine, dried over Na_2SO_4, filtered, and concentrated in vacuo. The residue was purified by flash column chromatography (EtOAc:n-Hexanes=1:10) to provide **11** as a colorless oil.

(*S*)-2,2-Difluoro-4-phenyl-5-(4,4,5,5-tetramethyl-1,3,2-dioxaborolan-2-yl)pentan-1-ol (11)

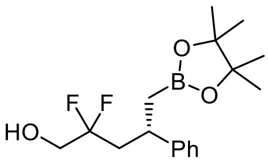

Colorless oil (47.6 mg, 73% yield, 98% ee); **^{1}H NMR** (400 MHz, $CDCl_3$) δ 7.26 – 7.14 (m, 4H), 7.14 – 7.07 (m, 1H), 3.53 (q, *J* = 12.8 Hz, 1H), 3.48 – 3.33 (m, 1H), 3.12 (qd, *J* = 8.2, 5.1 Hz, 1H), 2.38 – 2.12 (m, 2H), 1.33 – 1.07 (m, 2H), 1.04 (s, 12H); **^{13}C {^{1}H} NMR** (101 MHz, $CDCl_3$) δ 146.2, 128.4, 127.2, 126.4, 123.1 (t, *J* = 243.0 Hz), 83.3, 64.0 (t, *J* = 31.9 Hz), 41.7 (t, *J* = 23.1 Hz), 35.7 (t, *J* = 4.4 Hz), 24.6, 24.6; **^{19}F NMR** (377 MHz, $CDCl_3$) δ-103.07 – -104.26 (m, 1F), -105.50 – -106.68 (m, 1F), **^{11}B NMR** (128 MHz, $CDCl_3$) δ 33.79; **HRMS (EI)** m/z calcd for $C_{17}H_{25}BF_2O_3$ $[M+Na]^+$: 349.1763; Found: 349.1766.

Optical rotation: $[\alpha]_D^{25}$: -21.8 (*c* = 0.500, $CHCl_3$).

HPLC condition: Chiral column OD-H, n-Hexane/i-PrOH = 98:2, flow rate = 1.0 mL/min, wavelength = 220 nm, t_R = 18.2 min for the major isomer, t_R = 17.0 min for the minor isomer.

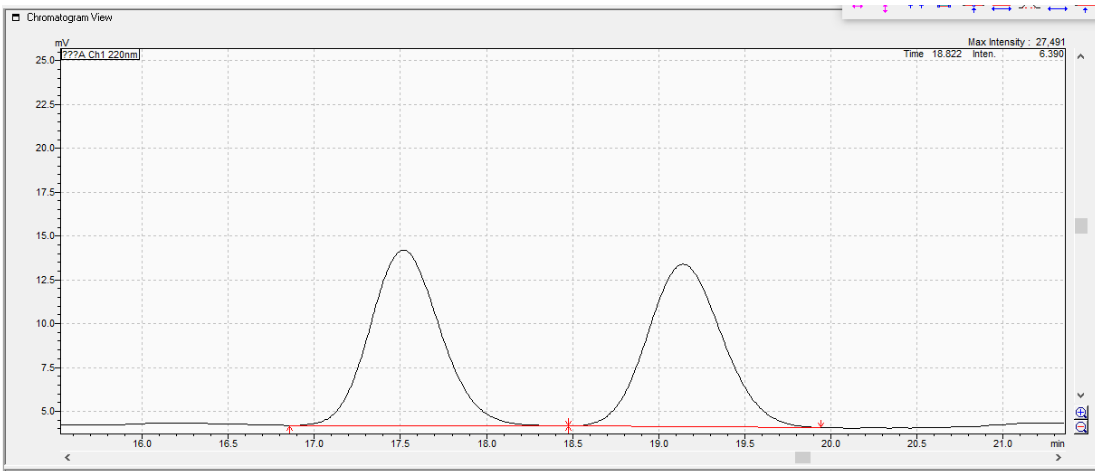

Results View - Peak Table

Peak Table | Compound | Group | Calibration Curve

Peak#	Ret. Time	Area	Height	Mark	Conc.	Unit	ID#	Name	Area%
1	17.518	285352	10026		50.315				50.315
2	19.142	281774	9278		49.685				49.685
Total		567126	19304		100.000				100.000

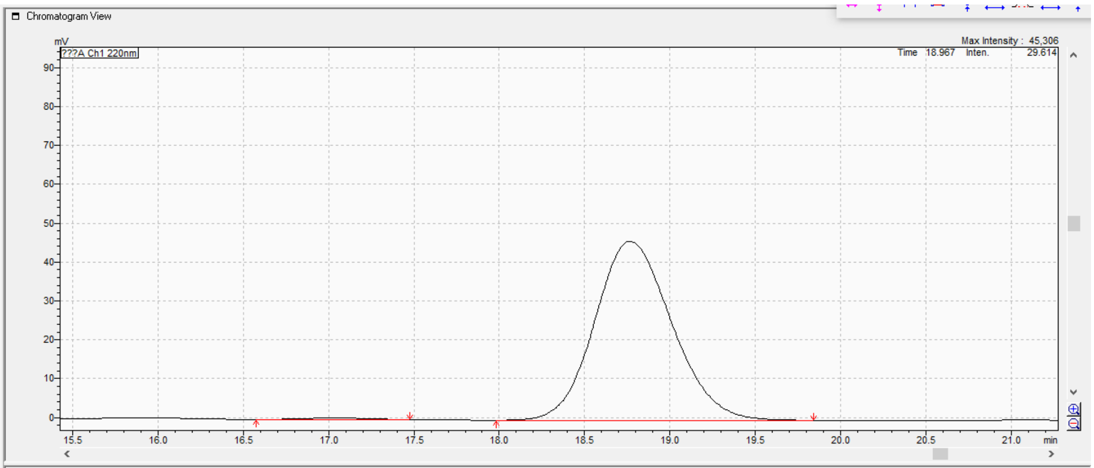

Results View - Peak Table

Peak Table | Compound | Group | Calibration Curve

Peak#	Ret. Time	Area	Height	Mark	Conc.	Unit	ID#	Name	Area%
1	17.051	9597	404	M	0.660				0.660
2	18.766	1445254	46019		99.340				99.340
Total		1454850	46423		100.000				100.000

Synthesis of Terfenadine derivatives.

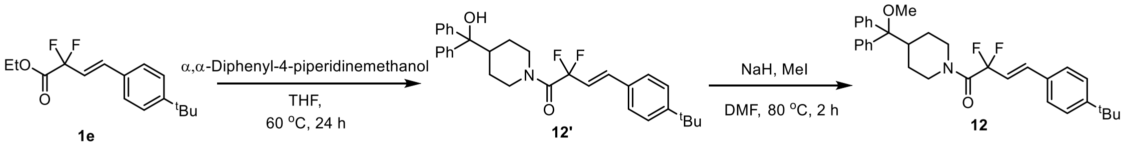

Compound **1e** (0.564 g, 2 mmol), α,α-Diphenyl-4-piperidinemethanol (0.640 g, 2.4 mmol), and dry THF (2.0 mL) were added to a flame-dried flask and purged with argon. After stirring at 80 °C in an oil bath for 24 h, the solvent was removed and the residue was purified by flash chromatography on silica gel with EtOAc/n-Hexane (1:5) as the eluent to give product **12'** as a white solid (0.483 g, 48% yield). The intermediate **12'** was then dissolved in DMF and NaH (0.160 g, 60 % dispersion in mineral oil, 4 mmol) was added in portion at room temperature. After stirring the mixture at room temperature for 30 min, MeI (0.567 g, 4mmol) was added dropwise via syringe. The resulting suspension was heated to 80 °C and stirred for further 2 h until TLC indicated the completion of the reaction. The reaction was quenched with H_2O (10 mL), and the resulting mixture was extracted with EtOAc (20 mL x 3). The combined organic layers were washed with brine, dried over Na_2SO_4 and concentrated in vacuo. The residue was purified by flash column chromatography (EtOAc:n-Hexanes=1:10) to provide **12** as a colorless oil (0.282 g, 55% yield). **^{1}H NMR** (400 MHz, $CDCl_3$) δ 7.43 – 7.21 (m, 14H), 6.80 (dt, *J* = 16.3, 2.8 Hz, 1H), 6.29 (dt, *J* = 16.3, 10.3 Hz, 1H), 4.66 (d, *J* = 13.1 Hz, 1H), 4.19 (d, *J* = 13.7 Hz, 1H), 3.11 (t, *J* = 13.0 Hz, 1H), 2.89 (s, 3H), 2.85 – 2.69 (m, 2H), 1.95 (dd, *J* = 26.5, 13.3 Hz, 2H), 1.36 (s, 9H), 1.04 – 0.83 (m, 2H); **^{13}C {^{1}H} NMR** (101 MHz, $CDCl_3$) δ 161.6 (t, *J* = 29.7 Hz), 152.8, 139.7, 135.4 (t, *J* = 9.8 Hz), 131.4, 129.3, 127.5, 127.5, 127.4, 127.1, 125.6, 119.4 (t, *J* = 25.3 Hz), 115.3, 85.7, 50.9, 46.5 (t, *J* = 5.0 Hz), 43.8, 42.8, 34.7, 31.2, 27.9, 27.5; **^{19}F NMR** (377 MHz, $CDCl_3$) δ -92.64 – -95.60 (m, 2F); **HRMS (EI)** m/z calcd for $C_{33}H_{37}F_2NO_2$ $[M+Na]^+$: 540.2690; Found: 540.2693.

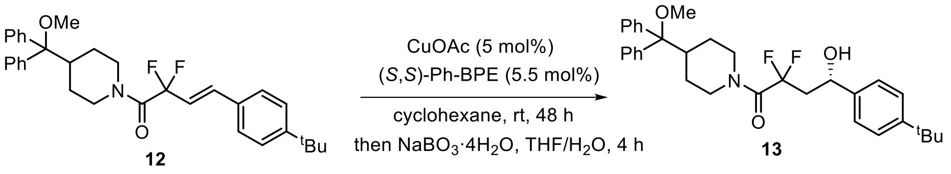

In an Ar-filled dry glovebox, CuOAc (1.2 mg, 10 μmol), (*S*, *S*)-Ph-BPE (5.6 mg, 11 μmol), cyclohexane (1 mL), and a magnetic stirring bar were added to a 4 mL screw-capped vial and stirred for 30 mins. The alkene **12** (103.4 mg, 0.200 mmol) were added and stirred for further 5 min, then HBpin (38.4 mg, 0.3 mmol) was added. The vial was sealed with a cap containing a PTFE septum and removed from the dry box. The reaction mixture was stirred at room temperature for 48 h and the resulting solution was concentrated in vacuum and used for oxidation without further purification.

To a stirred solution of above mixture in THF/H_2O (1:1; 2.0 mL) was added $NaBO_3 \cdot 4H_2O$ (61.6 mg). The reaction mixture was stirred vigorously at room temperature for 4 h. After that the mixture was diluted with H_2O and extracted with EtOAc (4 mL). The combined organic phases were dried over Na_2SO_4 and concentrated under reduced pressure. The residue was purified by flash column chromatography using EtOAc/n-Hexane (1:5) as eluent yielding product **13** as a colorless oil.

(*S*)-4-(4-(tert-butyl)phenyl)-2,2-Difluoro-4-hydroxy-1-(4-(methoxydiphenyl methyl)piperidin-1-yl)butan-1-one (13)

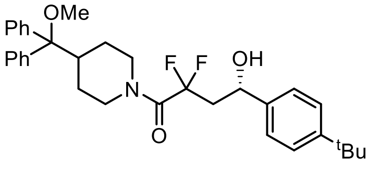

Colorless oil (86.7 mg, 81% yield, 97% ee); **^{1}H NMR** (400 MHz, $CDCl_3$) δ 7.36 – 7.09 (m, 14H), 4.82 (d, *J* = 7.6 Hz, 1H), 4.49 (d, *J* = 13.3 Hz, 1H), 4.26 (d, *J* = 13.7 Hz, 1H), 4.00 (d, *J* = 29.8 Hz, 1H), 3.06 (tt, *J* = 13.2, 2.6 Hz, 1H), 2.80 (s, 3H), 2.77 – 2.46 (m, 3H), 2.35 – 2.20 (m, 1H), 1.89 (d, *J* = 13.0 Hz, 2H), 1.22 (s, 9H), 0.98 – 0.72 (m, 2H); **^{13}C {^{1}H} NMR** (126 MHz, $CDCl_3$) δ 162.4 (td, *J* = 29.5, 7.9 Hz), 150.5, 140.9, 140.8, 139.7, 139.7, 129.3, 127.6, 127.6,

127.5, 127.5, 125.4, 125.3, 118.9 (td, J = 256.0, 6.9 Hz), 85.7, 67.8, 67.8, 51.0, 51.0, 46.3 (q, J = 6.1 Hz), 45.4 (t, J = 22.0 Hz), 44.1, 44.0, 42.9, 42.9, 31.3, 28.1, 28.0, 27.3; **^{19}F NMR** (377 MHz, $CDCl_3$) δ -89.44 – -92.71 (m, 1F), -96.84 – -100.13 (m, 1F); **HRMS (EI)** m/z calcd for $C_{33}H_{39}F_2NO_3$ $[M+Na]^+$: 558.2796; Found: 558.2793.

Optical rotation: $[\alpha]_D^{25}$: 2.7 (c = 1.0, $CHCl_3$).

HPLC condition: Chiral column OD-H, n-Hexane/i-PrOH = 95:5, flow rate = 0.5 mL/min, wavelength = 220 nm, t_R = 31.2 min for the major isomer, t_R = 33.0 min for the minor isomer.

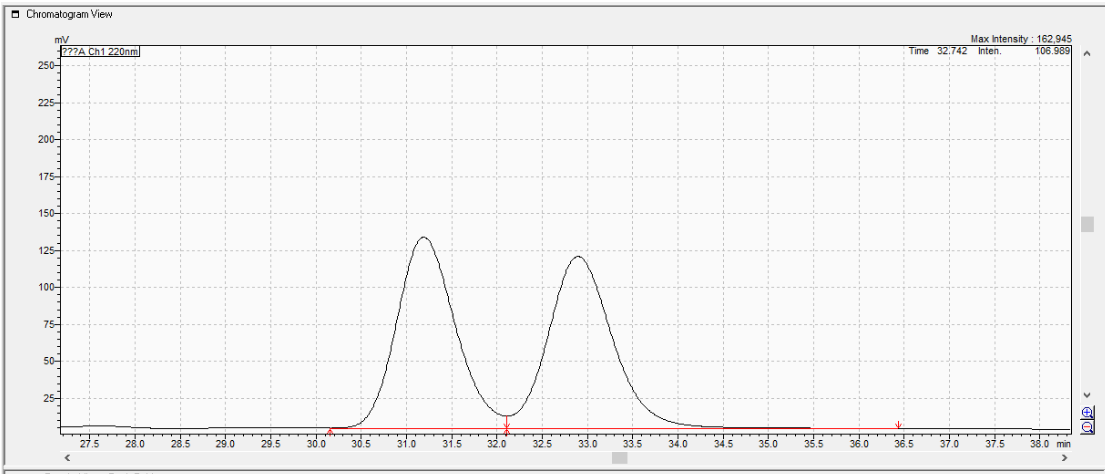

Results View - Peak Table

Peak Table | Compound | Group | Calibration Curve

Peak#	Ret. Time	Area	Height	Mark	Conc.	Unit	ID#	Name	Area%
1	31.191	5801485	129113		50.231				50.231
2	32.898	5748095	116155	SV	49.769				49.769
Total		11549580	245267		100.000				100.000

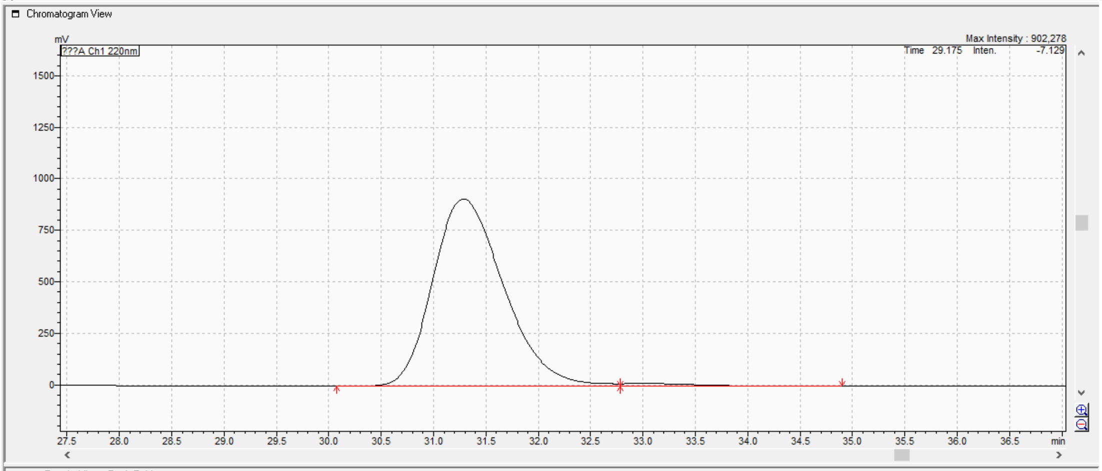

Results View - Peak Table

Peak Table | Compound | Group | Calibration Curve

Peak#	Ret. Time	Area	Height	Mark	Conc.	Unit	ID#	Name	Area%
1	31.286	42889199	909071	M	98.617				98.617
2	33.053	601470	12357	V M	1.383				1.383
Total		43490668	921428		100.000				100.000

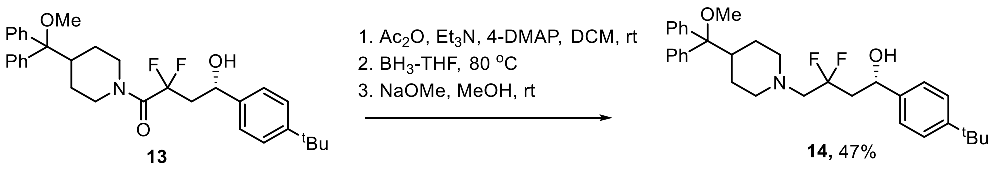

1. To a stirred solution of **13** (107.6 mg, 0.200 mmol) mixture in DCM (2 mL) was added Et_3N (40.4 mg, 0.400 mmol) 4-DMAP (2.4 mg, 0.020 mmol) at 0 °C. Then Ac_2O was added dropwise at the same temperature. The reaction mixture was stirred at room temperature for 12 h. After that the solvent was evaporated under reduced pressure. The residue was purified by flash column chromatography using EtOAc/n-Hexane (1:20) as eluent yielding the intermediate as a colorless oil.
2. To a stirred solution of above intermediate in THF (0.5 mL) was added BH_3-THF (1.0 M, 4 mL) at 0 °C. Then the reaction mixture was warmed to 80 °C and stirred for 72 h. After that MeOH (2 mL) was added to quench the reaction. The solvent was evaporated under reduced pressure and the residue was used without further purification.
3. To a stirred solution of above crude mixture in MeOH was added NaOMe (21.6 mg, 0.400 mmol). The reaction mixture was stirred at room temperature for 12 h. After that the mixture was diluted with H_2O and extracted with EtOAc (4 mL). The combined organic phases were dried over Na_2SO_4 and concentrated under reduced pressure. The residue was purified by flash column chromatography using EtOAc/n-Hexane (1:5) as eluent yielding product **14** as a colorless oil.

(*S*)-1-(4-(*tert*-butyl)phenyl)-3,3-difluoro-4-(4-(methoxydiphenylmethyl)piperidin-1-yl)butan-1-ol (14)

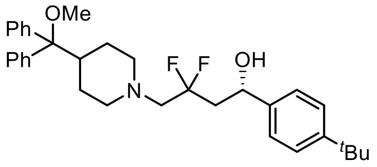

Colorless oil (49.0 mg, 47% yield); **^{1}H NMR** (400 MHz, $CDCl_3$) δ 7.31 – 7.20 (m, 12H), 7.17 – 7.09 (m, 2H), 4.75 – 4.65 (m, 1H), 3.16 (d, *J* = 11.7 Hz, 1H), 2.98 (dd, *J* = 9.2, 4.9 Hz, 1H), 2.81 (s, 3H), 2.89 – 2.66 (m, 2H), 2.47 (t, *J* = 11.9 Hz, 1H), 2.39 – 2.15 (m, 4H), 1.85 – 1.73 (m, 2H), 1.22 (s,

9H), 1.13 – 0.99 (m, 2H); **^{13}C {^{1}H} NMR** (126 MHz, $CDCl_3$) δ 150.1, 143.2, 140.8, 140.3, 129.2, 127.5, 127.3, 127.3, 125.2, 125.2, 123.5 (dd, *J* = 242.1, 239.9 Hz), 85.6, 67.7 (dd, *J* = 10.3, 2.4 Hz), 55.2, 50.9, 47.0 (t, *J* = 24.7 Hz), 42.2, 34.4, 31.3, 29.4; **^{19}F {^{1}H} NMR** (377 MHz, $CDCl_3$) δ -84.94 (d, *J* = 262.8 Hz, 1F), -95.79 (d, *J* = 258.3 Hz, 1F); **HRMS (EI)** m/z calcd for $C_{33}H_{41}F_2NO_2$ $[M+Na]^+$: 544.3003; Found: 544.3011.

Optical rotation: $[\alpha]_D^{25}$: 21.4 (*c* = 1.00, $CHCl_3$)

Since the racemic samples are difficult to obtain, please refer to amide compound **13** for ee value.

5. Mechanistic Studies

Deuterium labe lling experiment.

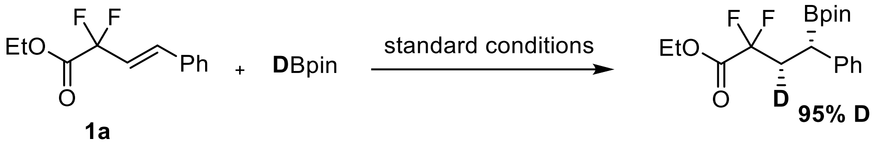

1a

D-**2a**, 61%, 99% ee

In an Ar-filled dry glovebox, CuOAc (0.6 mg, 5 μmol), (*S*, *S*)-Ph-BPE (2.8 mg, 5.5 μmol), cyclohexane (0.5 mL), and a magnetic stirring bar were added to a 4 mL screw-capped vial and stirred for 30 mins. The difluoromethyl alkenes (0.1 mmol) were added and stirred for further 5 min, then D-Bpin (19.3 mg, 0.15 mmol) ws added. The vial was sealed with a cap containing a PTFE septum and removed from the dry box. The reaction mixture was stirred at room temperature for 48 h and the resulting solution was concentrated in vacuum. The residue was purified by silica gel flash column chromatography with EtOAc/n-Hexane (1:40) as eluent to afford the desired products **D-2a** as a colorless oil. Deuterium incorporation was quantified by ^{1}H NMR analysis and ^{19}F NMR analysis.

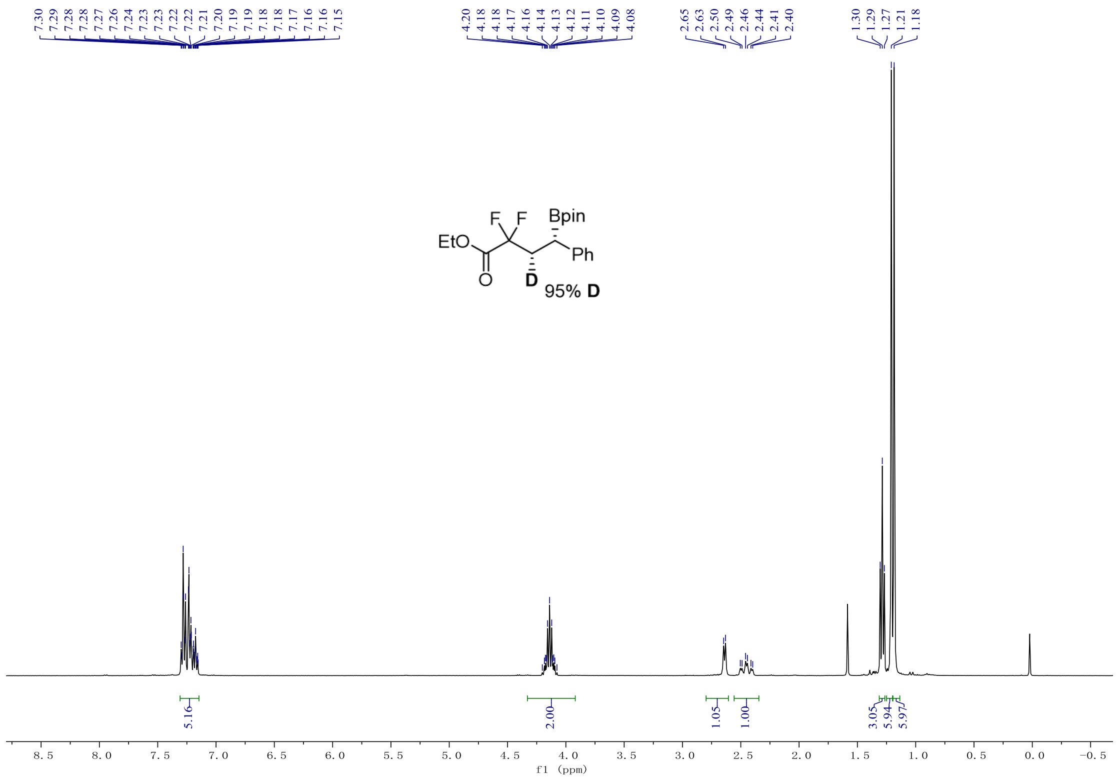
7.30 7.29 7.28 7.28 7.27 7.26 7.24 7.23 7.23 7.22 7.22 7.21 7.20 7.19 7.19 7.18 7.18 7.17 7.16 7.16 7.15
4.20 4.18 4.18 4.17 4.16 4.14 4.13 4.12 4.11 4.10 4.09 4.08
2.65 2.63 2.50 2.49 2.46 2.44 2.41 2.40
1.30 1.29 1.27 1.21 1.18
EtO
F F
Bpin
Ph
O
D
95% D
5.16
2.00
1.05
1.00
3.05
5.94
5.97
f1 (ppm)

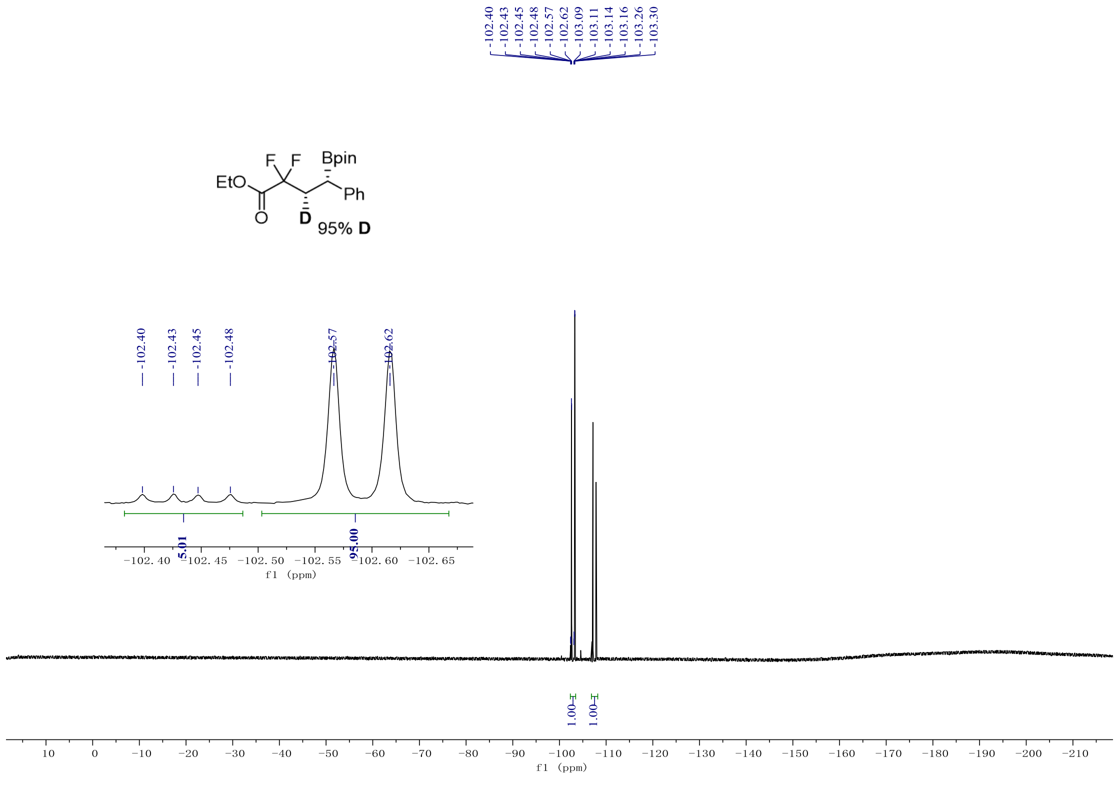
-102.40 -102.43 -102.45 -102.48 -102.57 -102.62 -103.09 -103.11 -103.14 -103.16 -103.26 -103.30
EtO
F F
Bpin
Ph
O
D
95% D
5.01
95.00
f1 (ppm)
1.00
1.00
f1 (ppm)

Control experiments with other type of substrates.

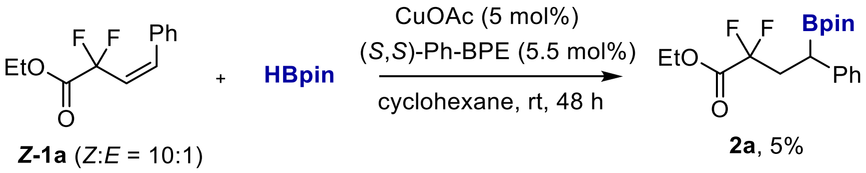

In an Ar-filled dry glovebox, CuOAc (0.6 mg, 5 μmol), (*S*, *S*)-Ph-BPE (2.8 mg, 5.5 μmol), cyclohexane (0.5 mL), and a magnetic stirring bar were added to a 4 mL screw-capped vial and stirred for 30 mins. Then **Z-1a** (0.1 mmol, prepared according to the previously reported procedure[10] with a Z/E ratio of 10:1) were added and stirred for further 5 min, HBpin (19.3 mg, 0.15 mmol) were added. The vial was sealed with a cap containing a PTFE septum and removed from the dry box. The reaction mixture was stirred at room temperature for 48 h and the resulting solution was concentrated in vacuum. The yield was determined by ^{19}F-NMR of crude reaction mixtures with $PhOCF_3$ as the internal standard.

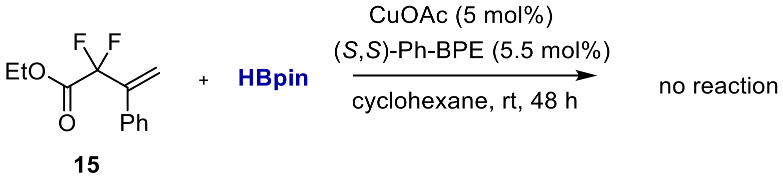

In an Ar-filled dry glovebox, CuOAc (0.6 mg, 5 μmol), (*S*, *S*)-Ph-BPE (2.8 mg, 5.5 μmol), cyclohexane (0.5 mL), and a magnetic stirring bar were added to a 4 mL screw-capped vial and stirred for 30 mins. Then **15** (0.1 mmol, prepared according to the previously reported procedure[11]) were added and stirred for further 5 min, HBpin (19.3 mg, 0.15 mmol) were added. The vial was sealed with a cap containing a PTFE septum and removed from the dry box. The reaction mixture was stirred at room temperature for 48 h and analyzed by GC/MS.

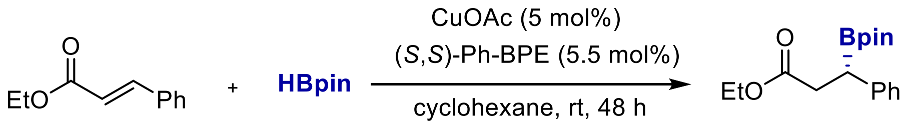

In an Ar-filled dry glovebox, CuOAc (1.2 mg, 10 µmol), (*S*, *S*)-Ph-BPE (5.6 mg, 11 µmol), cyclohexane (1 mL), and a magnetic stirring bar were added to a 4 mL screw-capped vial and stirred for 30 mins. The cinnamate **16** (0.2 mmol) were added and stirred for further 5 min, then HBpin (38.4 mg, 0.3 mmol) were added. The vial was sealed with a cap containing a PTFE septum and removed from the dry box. The reaction mixture was stirred at room temperature for 48 h and the resulting solution was concentrated in vacuum. The residue was purified by silica gel flash column chromatography with EtOAc/n-Hexane (1:30) as eluent to afford the desired products **17** as a colorless oil.

ethyl (*S*)-3-phenyl-3-(4,4,5,5-tetramethyl-1,3,2-dioxaborolan-2-yl)propanoate (17)

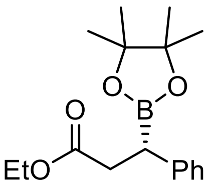

Colorless oil (29.8 mg, 49% yield, 89% ee); **^{1}H NMR** (400 MHz, $CDCl_3$) δ 7.31 – 7.21 (m, 4H), 7.21 – 7.14 (m, 1H), 4.22 – 4.04 (m, 2H), 2.90 (dd, *J* = 16.0, 9.9 Hz, 1H), 2.76 (dd, *J* = 9.8, 6.0 Hz, 1H), 2.67 (dd, *J* = 16.0, 6.0 Hz, 1H), 1.26 – 1.16 (m, 15H); **^{13}C {^{1}H} NMR** (101 MHz, $CDCl_3$) δ 173.3, 141.3, 128.4, 128.1, 125.6, 83.5, 60.3, 37.3, 24.5, 24.4, 14.2, **^{11}B NMR** (128 MHz, $CDCl_3$) δ 33.41; **HRMS (EI)** m/z calcd for $C_{17}H_{25}BO_4$ $[M+Na]^+$: 327.1744; Found: 327.1748.

Optical rotation: $[\alpha]_D^{25}$: 18.0 (*c* = 1.00, C HCl_3).

HPLC condition: Chiral column OD-H, n-Hexane/i-PrOH = 99:1, flow rate = 1.0 mL/min, wavelength = 220 nm, t_R = 10.7 min for the major isomer, t_R = 6.4 min for the minor isomer.

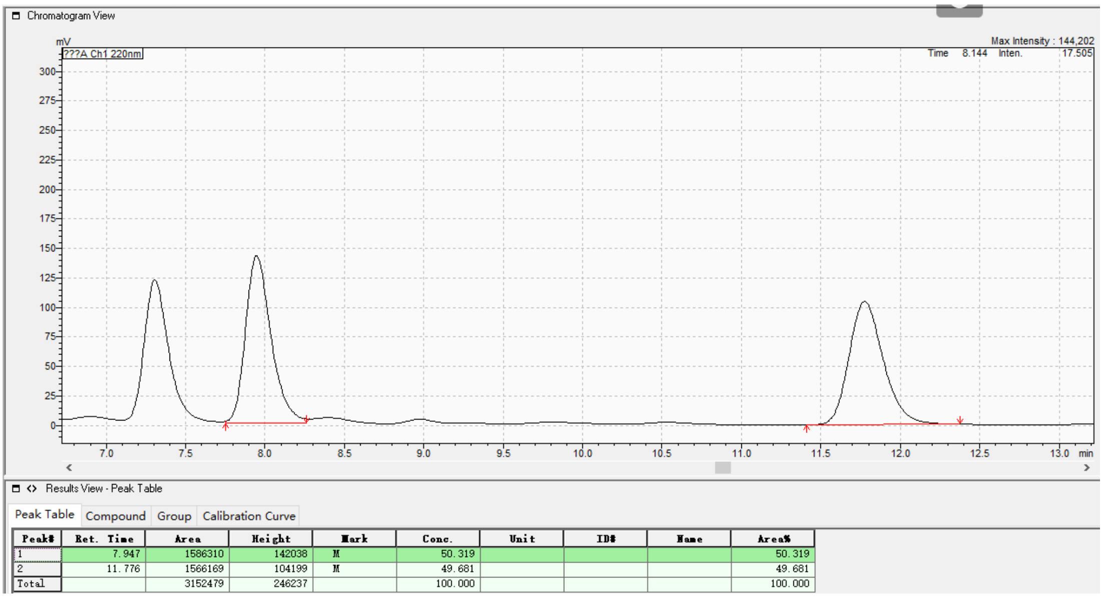

Peak#	Ret. Time	Area	Height	Mark	Conc.	Unit	ID#	Name	Area%
1	7.947	1586310	142038	M	50.319				50.319
2	11.776	1566169	104199	M	49.681				49.681
Total		3152479	246237		100.000				100.000

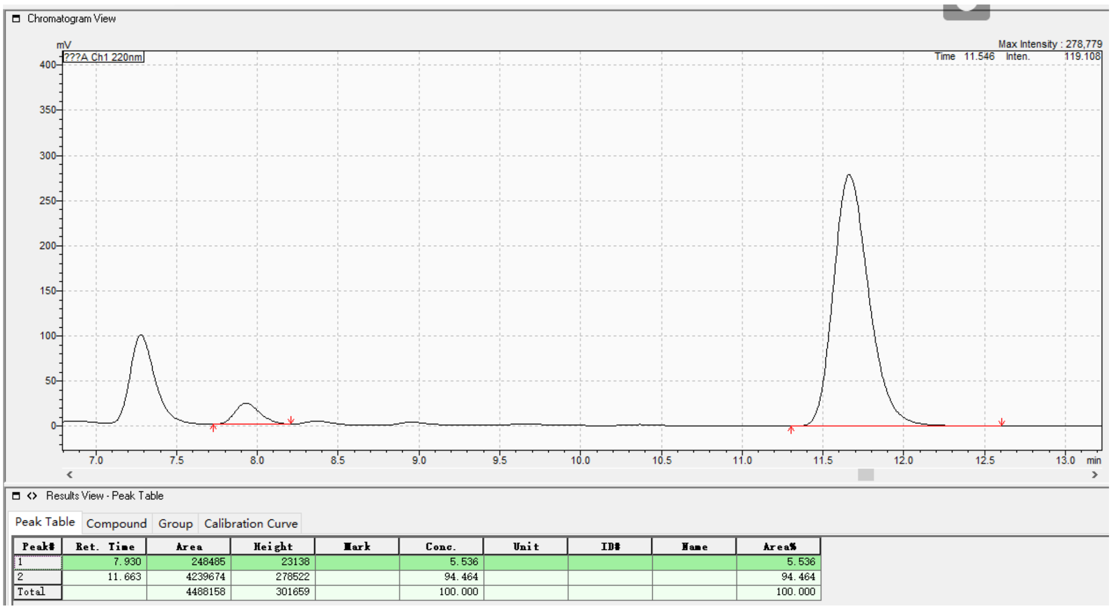

Peak#	Ret. Time	Area	Height	Mark	Conc.	Unit	ID#	Name	Area%
1	7.930	248485	23138		5.536				5.536
2	11.663	4239674	278522		94.464				94.464
Total		4488158	301659		100.000				100.000

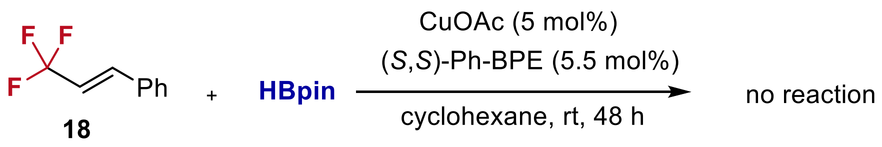

In an Ar-filled dry glovebox, CuOAc (0.6 mg, 5 μmol), (*S*, *S*)-Ph-BPE (2.8 mg, 5.5 μmol), cyclohexane (0.5 mL), and a magnetic stirring bar were added to a 4 mL screw-capped vial and stirred for 30 mins. Compound **18** (0.1 mmol, prepared according to the previously reported procedure[1]) were added and stirred for further 5

min, then HBpin (19.3 mg, 0.15 mmol) was added. The vial was sealed with a cap containing a PTFE septum and removed from the dry box. The reaction mixture was stirred at room temperature for 48 h and analyzed by GC/MS.

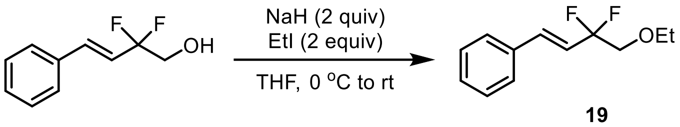

19

To a stirred solution of the (*E*)-2,2-difluoro-4-phenylbut-3-en-1-ol (3 mmol) in THF (10 mL) at 0 °C, NaH (0.240 g, 60 % dispersion in mineral oil, 6 mmol) was added in portion at the same temperature. After stirring the mixture at 0 °C for 30 min, EtI (0.93 g, 4mmol) was added dropwise via syringe. The resulting suspension was stirred at room temperature for overnight until TLC indicated the completion of the reaction. The reaction was quenched with H_2O (10 mL), and the resulting mixture was extracted with EtOAc (20 mL x 3). The combined organic layers were washed with brine, dried over Na_2SO_4 and concentrated in vacuo. The residue was purified by flash column chromatography (EtOAc:n-Hexanes=1:50) to provide **19** as a colorless oil (0.394 g, 62% yield). **^{1}H NMR** (400 MHz, $CDCl_3$) δ 7.52 – 7.45 (m, 2H), 7.43 – 7.31 (m, 3H), 7.03 (dt, *J* = 16.3, 2.7 Hz, 1H), 6.32 (dt, *J* = 16.2, 11.4 Hz, 1H), 3.79 (t, *J* = 12.5 Hz, 2H), 3.68 (q, *J* = 7.0 Hz, 2H), 1.28 (t, *J* = 7.0 Hz, 3H); **^{13}C {^{1}H} NMR** (101 MHz, $CDCl_3$) δ 134.9, 134.8 (t, *J* = 9.5 Hz), 129.0, 128.7, 127.2, 121.1 (t, *J* = 25.1 Hz), 119.5 (t, *J* = 240.6 Hz), 72.3 (t, *J* = 32.7 Hz), 67.8, 15.0; **^{19}F NMR** (377 MHz, $CDCl_3$) δ -102.80 (qd, *J* = 12.3, 2.7 Hz, 2F); **HRMS (EI)** m/z calcd for $C_{12}H_{14}F_2O$ $[M+H]^+$: 213.1091; Found: 213.1098.

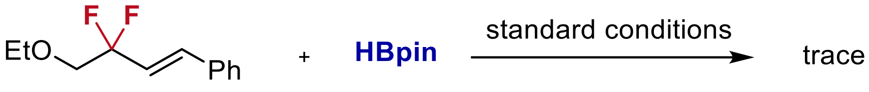

19

In an Ar-filled dry glovebox, CuOAc (1.2 mg, 10 µmol), (*S*, *S*)-Ph-BPE (5.6 mg, 11 µmol), cyclohexane (1 mL), and a magnetic stirring bar were added to a 4 mL screw-

capped vial and stirred for 30 mins. Compound **19** (42.4 mg, 0.200 mmol) were added and stirred for further 5 min, then HBpin (38.6 mg, 0.300 mmol) was added. The vial was sealed with a cap containing a PTFE septum and removed from the dry box. The reaction mixture was stirred at room temperature for 48 h and analyzed by TLC and GC/MS.

6. Details of Computational Studies

All calculations were performed with Gaussian 09.[12] Geometry optimizations were conducted with M06-L[13] functional and 6-31G* basis set was used for all atoms frequency analysis was also performed at the same level of theory as geometry optimization to confirm whether optimized stationary points were either local minimum or transition state. Single-point energies were then calculated at the M06-L/def2-TZVP[14] level, and the Truhlar-Cramer universal solvation model based on density (SMD)[15] for cyclohexane (ε: 2.0165). Multiwfn[16] was utilized to analyze noncovalent interactions (NCIs) with the independent gradient model based on Hirshfeld partition (IGMH).[17] Molecular visualizations were performed with PyMOL.[18]

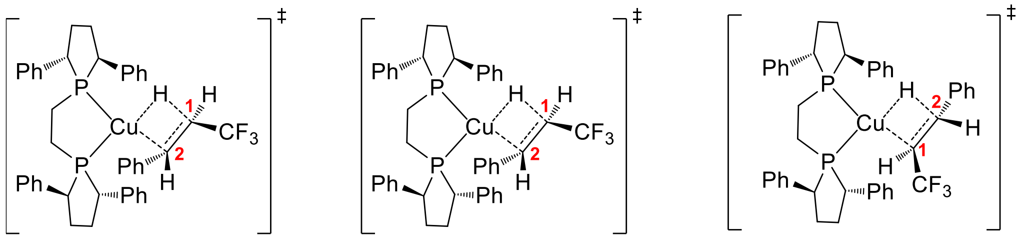

TS1-A 11.9 | TS1'-A 21.4 | TS1''-A 13.6

Figure S1. The three transition states of migratory insertion of substrate **18** into Cu-H bond.

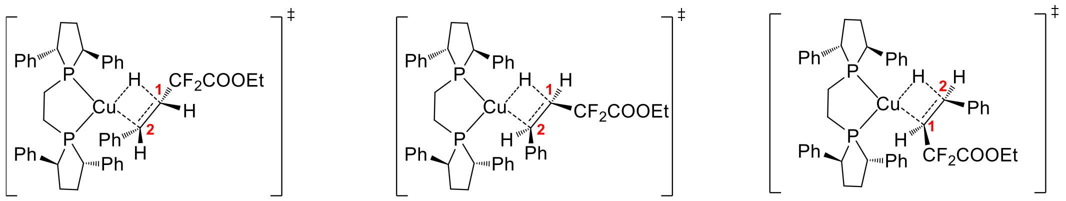

Figure S2. The three transition states of migratory insertion of substrate *Z*-**1a** into Cu-H bond.

IM1

M06-L SCF energy in Solvent: -3641.452279 a.u.

M06-L Free energy in Solvent: -3640.899227 a.u.

C	0.731856	0.763784	1.761999
H	1.222728	1.746217	1.709035
H	0.977506	0.335871	2.744435
C	-0.786963	0.919042	1.629429
H	-1.287219	0.037966	2.057080
H	-1.134931	1.793420	2.201500
P	-1.377291	1.000585	-0.134574
P	1.462611	-0.268559	0.392090
C	3.443905	-0.788933	2.127171
H	3.259363	-0.004040	2.870553
H	4.474953	-1.123544	2.300778
C	2.428098	-1.938848	2.324386
H	2.931820	-2.910844	2.406935
H	1.908421	-1.792104	3.278335
Cu	0.220839	-0.009860	-1.499721
C	-3.693457	2.434189	-0.301540
H	-4.667084	2.681507	0.141140
H	-3.829058	2.456477	-1.393180
C	-2.596158	3.409430	0.097258

H	-2.795349	4.433788	-0.246929
H	-2.526379	3.450524	1.195643
C	1.419723	-1.974861	1.160642
H	1.824578	-2.618015	0.363754
C	3.296884	-0.168999	0.733086
H	3.590218	0.890718	0.743190
C	-3.248263	1.019784	0.120684
H	-3.407610	0.918605	1.206054
C	-1.307264	2.850064	-0.496895
H	-1.379611	2.908091	-1.594288
C	-0.004791	-2.387888	1.430652
C	-0.874224	-2.510672	0.333363
C	-0.536262	-2.567046	2.710027
C	-2.226790	-2.772616	0.508493
H	-0.477621	-2.378961	-0.678533
C	-1.894258	-2.837542	2.889301
H	0.104499	-2.500334	3.587413
C	-2.745240	-2.930641	1.794169
H	-2.881786	-2.839325	-0.359330
H	-2.283825	-2.974076	3.897262
H	-3.806238	-3.131926	1.934067
C	-3.988547	-0.085013	-0.581559
C	-3.691943	-0.427930	-1.906359
C	-5.003938	-0.783030	0.077569
C	-4.385282	-1.449709	-2.547086
H	-2.882903	0.085337	-2.429586
C	-5.701826	-1.804770	-0.561553
H	-5.236781	-0.530421	1.113347
C	-5.391677	-2.143581	-1.876321

H	-4.129109	-1.711695	-3.572036
H	-6.485662	-2.341370	-0.028617
H	-5.929472	-2.947203	-2.376277
C	0.011460	3.425238	-0.073615
C	1.158567	3.094605	-0.812620
C	0.165284	4.198195	1.080381
C	2.420111	3.502709	-0.394868
H	1.048936	2.489998	-1.718216
C	1.430556	4.610257	1.499026
H	-0.709185	4.482371	1.664716
C	2.562554	4.257506	0.770060
H	3.297027	3.234373	-0.983107
H	1.528470	5.211383	2.401747
H	3.549098	4.579573	1.098764
C	4.024127	-0.879223	-0.383561
C	4.059706	-0.299168	-1.658819
C	4.627495	-2.128289	-0.209721
C	4.695030	-0.934797	-2.717775
H	3.557939	0.656080	-1.821774
C	5.264377	-2.769448	-1.270746
H	4.605134	-2.614694	0.765257
C	5.303374	-2.174444	-2.526653
H	4.703380	-0.465661	-3.699913
H	5.728676	-3.741326	-1.111218
H	5.798563	-2.675749	-3.356163
H	0.257053	-0.149595	-3.050702

1a

M06-L SCF energy in Solvent: -814.840443 a.u.

M06-L Free energy in Solvent: -814.666922 a.u.

C	-0.106509	-0.926643	0.028256
C	-1.022871	-0.211898	-0.638936
H	-0.293757	-1.369839	1.005255
H	-0.729322	0.203982	-1.604189
C	1.278139	-1.141459	-0.474317
F	1.572539	-2.480216	-0.484118
F	1.424238	-0.685037	-1.748421
C	2.300680	-0.480634	0.461165
O	2.487382	-0.883273	1.586150
O	2.883880	0.578283	-0.102382
C	3.817648	1.277243	0.755179
H	4.555594	0.555032	1.122737
H	3.266134	1.650674	1.626340
C	4.435260	2.379124	-0.058923
H	3.673074	3.074335	-0.425566
H	5.147385	2.943662	0.552069
H	4.971477	1.975181	-0.923706
C	-2.388520	0.076655	-0.221050
C	-3.209257	0.835087	-1.069481
C	-2.924641	-0.364439	0.999785
C	-4.517355	1.144725	-0.715514
H	-2.805322	1.182814	-2.020396
C	-4.229790	-0.055748	1.354143
H	-2.310713	-0.954200	1.678820
C	-5.032310	0.700546	0.498903
H	-5.135710	1.734841	-1.389392
H	-4.627076	-0.406030	2.305048
H	-6.055691	0.941384	0.780415

TS1

M06-L SCF energy in Solvent: -4456.305268 a.u.

M06-L Free energy in Solvent: -4455.548083 a.u.

C	0.248231	-0.394560	2.821034
H	-0.720264	-0.825948	3.110643
H	0.693062	0.039444	3.728851
C	1.167441	-1.480002	2.260490
H	2.186403	-1.077750	2.147451
H	1.233189	-2.330906	2.955976
P	0.679109	-2.043775	0.558847
P	-0.092997	0.931213	1.561568
C	-1.116870	2.406443	3.574045
H	-1.224915	1.660110	4.371006
H	-1.760911	3.248427	3.861191
C	0.365947	2.838079	3.469591
H	0.467714	3.930101	3.523699
H	0.911909	2.452271	4.338170
C	1.593776	-4.568960	0.066033
H	2.395884	-5.302360	0.216278
H	1.159785	-4.778586	-0.921407
C	0.500444	-4.675575	1.123556
H	-0.018162	-5.643300	1.091237
H	0.953009	-4.591657	2.123957
C	0.997578	2.328962	2.163216
H	0.876183	3.093406	1.378660
C	-1.606248	1.772487	2.264402
H	-2.355594	0.992997	2.461601
C	2.151335	-3.136432	0.098718

H	2.851260	-3.052934	0.944220
C	-0.454941	-3.512975	0.871625
H	-0.954253	-3.669597	-0.097938
C	2.435397	1.885958	2.161963
C	3.067849	1.745102	0.918019
C	3.144695	1.515041	3.308422
C	4.364215	1.256730	0.821682
H	2.525586	2.023741	0.014766
C	4.445913	1.020921	3.212947
H	2.692133	1.613614	4.294488
C	5.061004	0.886834	1.971577
H	4.823053	1.165581	-0.162446
H	4.982156	0.745141	4.120021
H	6.078764	0.505395	1.902177
C	2.834393	-2.636984	-1.142249
C	2.273489	-2.826619	-2.409741
C	4.002234	-1.875693	-1.040032
C	2.867695	-2.280294	-3.542358
H	1.341607	-3.381678	-2.512106
C	4.600262	-1.327369	-2.171081
H	4.439191	-1.701415	-0.055184
C	4.036303	-1.529962	-3.428272
H	2.405049	-2.429610	-4.516753
H	5.511114	-0.738081	-2.069466
H	4.500089	-1.098922	-4.314107
C	-1.490594	-3.169978	1.898015
C	-2.577122	-2.366578	1.516627
C	-1.368090	-3.522072	3.246123
C	-3.488694	-1.904161	2.459350

H	-2.684967	-2.097470	0.465181
C	-2.286365	-3.064848	4.190071
H	-0.545082	-4.158999	3.568969
C	-3.342567	-2.243751	3.804151
H	-4.321812	-1.276061	2.142599
H	-2.172979	-3.352733	5.234231
H	-4.056255	-1.883540	4.542932
C	-2.140558	2.721002	1.223779
C	-2.928248	2.221728	0.176349
C	-1.810211	4.079680	1.210635
C	-3.353231	3.048801	-0.857713
H	-3.174940	1.158026	0.160414
C	-2.249856	4.914460	0.184274
H	-1.196122	4.495902	2.009783
C	-3.014928	4.401856	-0.857744
H	-3.937835	2.630678	-1.676663
H	-1.979778	5.969273	0.196152
H	-3.342625	5.049013	-1.668974
H	-1.595567	-0.213744	-0.867858
C	0.494711	0.570509	-2.265261
C	-0.923522	0.346735	-2.202879
H	-1.575359	1.225014	-2.193896
H	1.092425	-0.204678	-2.744327
Cu	-0.058006	-0.187737	-0.398090
C	1.108926	1.884023	-2.140548
C	2.430864	2.081718	-2.587434
C	0.456877	2.987243	-1.551282
C	3.067041	3.309373	-2.444817
H	2.958347	1.241565	-3.042198

C 1.097293 4.212001 -1.410529

H -0.559183 2.867303 -1.169818

C 2.409690 4.386985 -1.852216

H 4.091968 3.425053 -2.798021

H 0.561610 5.038509 -0.941683

H 2.910557 5.346089 -1.735110

C -1.517028 -0.753523 -3.045285

C -2.817290 -1.331446 -2.466647

O -2.966731 -2.484688 -2.135580

O -3.755603 -0.374478 -2.383528

C -5.003310 -0.823320 -1.807086

H -4.790866 -1.246103 -0.816235

H -5.393520 -1.637808 -2.428432

C -5.936925 0.352551 -1.742597

H -5.556479 1.130041 -1.071048

H -6.914114 0.031272 -1.366801

H -6.082577 0.795149 -2.733721

F -0.648974 -1.791985 -3.183195

F -1.777777 -0.282884 -4.307332

IM2

M06-L SCF energy in Solvent: -4456.344658 a.u.

M06-L Free energy in Solvent: -4455.580124 a.u.

C 0.095959 0.754241 2.747010

H -0.522169 0.023579 3.286105

H 0.162924 1.648868 3.380742

C 1.497721 0.181048 2.512598

H 2.207720 0.996531 2.314474

H 1.854846 -0.342063 3.412855

P	1.584405	-0.940007	1.029027
P	-0.810990	1.104820	1.152642
C	-2.445018	2.861178	2.349863
H	-1.926393	2.808723	3.318339
H	-3.434881	3.291883	2.557899
C	-1.642712	3.724402	1.375162
H	-2.280961	4.018078	0.530723
H	-1.308233	4.656848	1.847660
C	3.551559	-2.792724	1.128646
H	4.583548	-3.054391	1.393936
H	3.306794	-3.344412	0.209065
C	2.551893	-3.155020	2.220501
H	2.524385	-4.234306	2.424435
H	2.840037	-2.657862	3.160675
C	-0.454542	2.925327	0.798947
H	-0.477751	2.995748	-0.294278
C	-2.541657	1.441650	1.783440
H	-2.729161	0.714116	2.588355
C	3.422782	-1.281285	0.865375
H	3.916030	-0.741093	1.689019
C	1.200501	-2.637759	1.734270
H	0.910835	-3.225364	0.849131
C	0.938281	3.275123	1.240554
C	2.015875	2.914016	0.417287
C	1.220587	3.867435	2.475423
C	3.329478	3.142968	0.812573
H	1.808464	2.444677	-0.547802
C	2.536262	4.094446	2.876065
H	0.401773	4.155202	3.136945

C	3.595607	3.732420	2.047949
H	4.145627	2.859511	0.149502
H	2.731918	4.558299	3.841853
H	4.622360	3.915812	2.360254
C	3.973824	-0.790466	-0.444917
C	3.416392	-1.211227	-1.660636
C	5.021927	0.131845	-0.473142
C	3.897346	-0.715284	-2.867870
H	2.577743	-1.910789	-1.665983
C	5.504843	0.630155	-1.681749
H	5.458039	0.470303	0.468618
C	4.941357	0.208758	-2.882817
H	3.441509	-1.046659	-3.799560
H	6.322857	1.349629	-1.682521
H	5.312543	0.600054	-3.828423
C	0.040387	-2.583524	2.681651
C	-1.231400	-2.281543	2.170330
C	0.170269	-2.746347	4.063064
C	-2.334586	-2.146506	3.004398
H	-1.333091	-2.114794	1.094288
C	-0.933341	-2.606491	4.905096
H	1.142940	-2.987036	4.490990
C	-2.187848	-2.306196	4.382861
H	-3.309407	-1.903943	2.578707
H	-0.808539	-2.736054	5.978933
H	-3.046667	-2.199740	5.042793
C	-3.542903	1.205925	0.674428
C	-3.593719	-0.056733	0.063489
C	-4.418036	2.189256	0.210284

C	-4.484082	-0.330802	-0.966307
H	-2.895148	-0.829661	0.390161
C	-5.311587	1.921968	-0.826872
H	-4.402965	3.184162	0.653839
C	-5.348589	0.664931	-1.420855
H	-4.485728	-1.318438	-1.427479
H	-5.978324	2.709324	-1.175603
H	-6.040645	0.462662	-2.236032
H	-1.989496	-0.874706	-2.811949
C	-0.073682	0.155498	-2.514714
C	-0.981749	-0.819998	-3.247138
H	-1.121567	-0.550043	-4.309780
H	0.933400	0.066527	-2.945935
Cu	0.176384	-0.160911	-0.554420
C	-0.494278	1.572045	-2.598212
C	0.467957	2.586543	-2.779931
C	-1.833312	1.990731	-2.451958
C	0.125951	3.935002	-2.777484
H	1.511352	2.290898	-2.917246
C	-2.177188	3.338480	-2.458292
H	-2.616759	1.244463	-2.310394
C	-1.202331	4.326081	-2.611484
H	0.903832	4.686788	-2.911364
H	-3.224651	3.619206	-2.337579
H	-1.475320	5.379892	-2.613465
C	-0.419461	-2.216477	-3.255785
C	-0.442251	-3.012504	-1.940122
O	0.520828	-3.547992	-1.435970
O	-1.709723	-3.147076	-1.513179

C	-1.928695	-4.191093	-0.537289
H	-1.191994	-4.096105	0.268146
H	-1.747108	-5.151712	-1.035933
C	-3.341013	-4.061014	-0.041466
H	-3.506576	-3.080602	0.421335
H	-3.546373	-4.827039	0.713775
H	-4.061874	-4.177544	-0.857602
F	0.873714	-2.217935	-3.690073
F	-1.127222	-3.000233	-4.144554

HBpin

M06-L SCF energy in Solvent: -411.969868 a.u.

M06-L Free energy in Solvent: -411.810326 a.u.

C	-0.779384	-0.189821	-0.050244
C	0.779384	-0.189822	0.050244
H	-0.000001	3.114370	0.000000
B	0.000000	1.930195	0.000000
C	1.462287	-0.442568	-1.285780
H	2.531337	-0.224856	-1.192667
H	1.349883	-1.484917	-1.606528
H	1.053988	0.204631	-2.070556
C	1.348785	-1.099700	1.119414
H	1.094248	-2.147457	0.914782
H	2.440757	-1.017374	1.138099
H	0.974959	-0.841549	2.114514
C	-1.462286	-0.442569	1.285781
H	-2.531336	-0.224856	1.192668
H	-1.349881	-1.484918	1.606528
H	-1.053986	0.204631	2.070556

C	-1.348786	-1.099702	-1.119414
H	-1.094248	-2.147457	-0.914782
H	-2.440759	-1.017374	-1.138098
H	-0.974961	-0.841549	-2.114515
O	1.071240	1.190175	0.410347
O	-1.071242	1.190175	-0.410347

TS2

M06-L SCF energy in Solvent: -4868.306064 a.u.

M06-L Free energy in Solvent: -4867.359400 a.u.

C	2.806788	-2.003357	0.584935
H	2.899041	-2.047330	1.677389
H	3.782952	-2.291351	0.174722
C	1.746346	-2.991778	0.100436
H	1.852822	-3.149121	-0.984164
H	1.891581	-3.973817	0.576778
P	0.005273	-2.396831	0.351801
P	2.401628	-0.232131	0.178263
C	5.005231	0.356318	0.324241
H	5.279885	-0.704426	0.412611
H	5.876377	0.923480	0.678752
C	4.649330	0.689568	-1.121458
H	4.567733	1.780216	-1.223244
H	5.427692	0.363967	-1.823194
C	-1.678792	-4.486053	0.798773
H	-1.973520	-5.514787	0.555868
H	-2.599431	-3.946689	1.064949
C	-0.720573	-4.406984	1.980896
H	-1.164856	-4.797425	2.906551

H	0.175627	-5.014001	1.777800
C	3.283063	0.064042	-1.478589
H	2.646927	0.807338	-1.972406
C	3.757722	0.615920	1.170181
H	3.813419	0.073930	2.127246
C	-1.005116	-3.803019	-0.397503
H	-0.255917	-4.494531	-0.815491
C	-0.357259	-2.931181	2.113020
H	-1.271223	-2.379312	2.376431
C	3.331532	-1.162409	-2.346219
C	2.299368	-1.422071	-3.258588
C	4.378614	-2.089447	-2.260582
C	2.302310	-2.575732	-4.038342
H	1.484998	-0.701942	-3.356111
C	4.383128	-3.245216	-3.037072
H	5.209153	-1.901778	-1.578220
C	3.340475	-3.497908	-3.924917
H	1.490356	-2.749769	-4.744079
H	5.208831	-3.949867	-2.949741
H	3.344252	-4.399624	-4.534246
C	-1.884836	-3.305754	-1.514303
C	-3.279756	-3.321806	-1.458414
C	-1.270380	-2.719929	-2.629888
C	-4.037911	-2.753960	-2.481615
H	-3.793443	-3.756496	-0.602605
C	-2.022690	-2.159517	-3.653148
H	-0.178802	-2.684734	-2.677646
C	-3.414978	-2.170424	-3.578821
H	-5.122638	-2.737008	-2.395680

H	-1.520299	-1.687674	-4.497560
H	-4.010257	-1.700817	-4.359279
C	0.723974	-2.511182	3.061256
C	0.895734	-1.141307	3.316645
C	1.609920	-3.416729	3.650371
C	1.928548	-0.692853	4.130934
H	0.212130	-0.424360	2.856247
C	2.650862	-2.966222	4.462452
H	1.490692	-4.485933	3.475928
C	2.817688	-1.605232	4.701638
H	2.041426	0.375595	4.317468
H	3.333534	-3.686348	4.910892
H	3.629469	-1.256017	5.337448
C	3.450312	2.068393	1.463925
C	2.237283	2.399141	2.080510
C	4.350356	3.103865	1.193383
C	1.945227	3.709281	2.437909
H	1.499236	1.613678	2.255740
C	4.058210	4.420750	1.545923
H	5.302875	2.892695	0.709939
C	2.857970	4.729172	2.176118
H	0.992069	3.937521	2.914516
H	4.778926	5.207101	1.326132
H	2.628154	5.757406	2.449868
C	-1.681151	1.070755	-0.232470
H	-1.776814	2.138111	-0.435873
Cu	0.078141	-0.199595	-0.184513
C	0.915278	3.483499	-1.969069
C	-0.479036	3.453435	-2.671664

B	-0.097394	1.452981	-1.594530
H	0.304375	0.302480	-1.916759
O	-0.805983	2.050090	-2.658360
O	0.830373	2.376935	-1.048746
C	2.059223	3.196913	-2.935438
H	2.221505	4.019863	-3.642724
H	2.985262	3.057323	-2.362819
H	1.868494	2.283148	-3.513677
C	1.213619	4.741083	-1.177087
H	2.192726	4.648548	-0.692578
H	1.233389	5.625236	-1.828247
H	0.472088	4.905483	-0.389208
C	-0.466902	3.911664	-4.119814
H	-1.480916	3.865799	-4.532895
H	-0.116801	4.949148	-4.203175
H	0.174600	3.276936	-4.738326
C	-1.539144	4.240085	-1.907026
H	-1.394478	5.320545	-2.033889
H	-2.532201	3.976748	-2.282954
H	-1.520675	4.029534	-0.832060
C	-2.694917	0.323120	-1.084982
H	-2.393515	0.384553	-2.137941
H	-2.738606	-0.748648	-0.850617
C	-4.121256	0.847764	-1.064197
F	-4.140117	2.203556	-1.243561
F	-4.812070	0.305692	-2.118573
C	-4.904552	0.469113	0.194040
O	-5.319364	-0.653997	0.382277
O	-5.008165	1.497489	1.037711

C	-5.605215	1.191966	2.316471
H	-6.688744	1.098084	2.169707
H	-5.228008	0.217256	2.647849
C	-5.235708	2.303633	3.257896
H	-5.599944	3.269928	2.893121
H	-5.674473	2.121979	4.245279
H	-4.147240	2.361657	3.371878
C	-1.756806	0.917293	1.247490
C	-1.184511	1.909578	2.075510
C	-2.413085	-0.145640	1.902837
C	-1.258102	1.840441	3.460847
H	-0.673049	2.745597	1.593039
C	-2.500388	-0.203621	3.292303
H	-2.883174	-0.934336	1.311483
C	-1.924493	0.784522	4.088150
H	-0.801697	2.627214	4.063252
H	-3.030876	-1.036295	3.757000
H	-1.992147	0.734826	5.172923

2a

M06-L SCF energy in Solvent: -1226.855669 a.u.

M06-L Free energy in Solvent: -1226.495345 a.u.

C	-0.482654	-1.014055	-0.619180
H	-0.305630	-1.092971	-1.701336
C	-2.208731	2.324437	-0.452251
C	-1.358754	2.354766	0.855139
B	-0.959738	0.451487	-0.298201
O	-0.924647	0.972331	0.969415
O	-1.561013	1.270331	-1.217723

C	-3.641390	1.867475	-0.212988
H	-4.228940	2.632744	0.308106
H	-4.121165	1.661821	-1.176076
H	-3.671965	0.944299	0.379126
C	-2.180392	3.602689	-1.263773
H	-2.785889	3.485897	-2.169134
H	-2.596130	4.439445	-0.687842
H	-1.163796	3.865229	-1.570582
C	-2.129431	2.723840	2.105899
H	-1.462352	2.714404	2.974719
H	-2.553796	3.732176	2.018979
H	-2.944193	2.020361	2.299732
C	-0.103279	3.206040	0.725744
H	-0.341040	4.276573	0.727122
H	0.560065	3.000264	1.572538
H	0.445848	2.973260	-0.192876
C	0.802677	-1.375611	0.119297
H	0.732459	-1.127266	1.184982
H	1.022396	-2.450013	0.063164
C	1.985326	-0.639071	-0.445957
F	2.217423	-1.022663	-1.739939
F	1.728215	0.714388	-0.492069
C	3.284819	-0.837242	0.344434
O	3.372177	-1.534076	1.329692
O	4.278968	-0.143577	-0.211605
C	5.555721	-0.272082	0.456505
H	5.432485	0.047900	1.498026
H	5.828296	-1.334006	0.473267
C	6.544549	0.570696	-0.298443

H	6.242842	1.622929	-0.303200
H	7.531837	0.499605	0.169725
H	6.633496	0.236875	-1.337228
C	-1.657927	-1.904793	-0.273796
C	-2.778176	-1.916720	-1.116436
C	-1.702299	-2.675342	0.892197
C	-3.897475	-2.682325	-0.811278
H	-2.763536	-1.307994	-2.021410
C	-2.823179	-3.444095	1.198936
H	-0.852596	-2.683118	1.573253
C	-3.924664	-3.452845	0.349892
H	-4.752921	-2.679823	-1.485376
H	-2.831139	-4.041052	2.109702
H	-4.799017	-4.055146	0.589821

TS1’

M06-L SCF energy in Solvent: -4456.290093 a.u.

M06-L Free energy in Solvent: -4455.532931 a.u.

C	0.125262	1.500670	2.508958
H	0.877303	2.298758	2.432441
H	-0.032387	1.307802	3.580311
C	-1.192468	1.950722	1.871412
H	-2.030392	1.369481	2.282326
H	-1.388934	3.007272	2.109338
P	-1.282809	1.683373	0.043314
P	0.846857	0.001102	1.681853
C	2.123113	-0.469953	4.006127
H	1.889506	0.486178	4.490952
H	2.973381	-0.889190	4.561522

C	0.895706	-1.399778	4.067823
H	1.197906	-2.433271	4.277276
H	0.262260	-1.112239	4.912543
C	-2.874979	3.341425	-1.358925
H	-3.803495	3.915097	-1.475887
H	-2.654040	2.891582	-2.339919
C	-1.704901	4.200007	-0.898955
H	-1.457429	4.997941	-1.611557
H	-1.981425	4.692059	0.045913
C	0.106165	-1.378287	2.739951
H	0.344747	-2.286411	2.166709
C	2.499977	-0.189391	2.551180
H	3.034224	0.767434	2.456554
C	-3.050575	2.227437	-0.313133
H	-3.417845	2.696213	0.614507
C	-0.523434	3.255361	-0.683548
H	-0.144027	2.934262	-1.666124
C	-1.395165	-1.256274	2.819929
C	-2.167251	-1.644225	1.713652
C	-2.060208	-0.679070	3.908079
C	-3.541230	-1.436395	1.680118
H	-1.673296	-2.085516	0.847601
C	-3.440417	-0.481427	3.884237
H	-1.505700	-0.361827	4.789989
C	-4.185228	-0.847836	2.767213
H	-4.104944	-1.728724	0.794806
H	-3.931047	-0.030551	4.745990
H	-5.261518	-0.683052	2.742605
C	-3.989861	1.120624	-0.698695

C	-3.619331	0.164855	-1.649775
C	-5.256536	1.025870	-0.116344
C	-4.483897	-0.864537	-2.005700
H	-2.628631	0.213374	-2.099704
C	-6.132107	0.004224	-0.477328
H	-5.553788	1.757992	0.636582
C	-5.746519	-0.945956	-1.420073
H	-4.157236	-1.612412	-2.724922
H	-7.115291	-0.055037	-0.011872
H	-6.424578	-1.753066	-1.692568
C	0.646809	3.719493	0.134751
C	1.881906	3.069872	-0.017540
C	0.537859	4.708352	1.118095
C	2.964724	3.389612	0.795020
H	1.987060	2.303830	-0.785285
C	1.620760	5.025554	1.937160
H	-0.402392	5.241904	1.254755
C	2.836244	4.363714	1.784904
H	3.915975	2.878037	0.646068
H	1.511304	5.798320	2.696538
H	3.681965	4.616429	2.422181
C	3.290255	-1.274440	1.863334
C	3.848090	-1.022617	0.602996
C	3.464956	-2.546843	2.417364
C	4.552501	-2.008084	-0.079276
H	3.718528	-0.035932	0.153365
C	4.168282	-3.537507	1.734377
H	3.056647	-2.774819	3.400150
C	4.711557	-3.274851	0.481809

H	4.984536	-1.787239	-1.055029
H	4.290230	-4.519572	2.188889
H	5.261447	-4.046824	-0.053060
H	-0.150322	-1.629057	-0.724981
C	0.673149	0.140918	-2.556487
C	0.565172	-1.225910	-2.146972
H	1.495464	-1.760929	-1.938611
H	-0.211630	0.560173	-3.041788
Cu	0.076623	-0.055248	-0.486010
C	1.908891	0.859129	-2.806170
C	1.862383	2.129069	-3.420813
C	3.184537	0.372763	-2.457061
C	3.010907	2.881731	-3.628598
H	0.893769	2.520250	-3.738586
C	4.330341	1.129721	-2.658042
H	3.269263	-0.617401	-2.012975
C	4.258083	2.398229	-3.236365
H	2.929433	3.859971	-4.101369
H	5.298000	0.720581	-2.364027
H	5.157614	2.990112	-3.392190
C	-0.393836	-2.138735	-2.874624
C	-0.762576	-3.381956	-2.054572
O	-1.870237	-3.649886	-1.654139
O	0.355738	-4.092737	-1.843129
C	0.242675	-5.173134	-0.890996
H	-0.718494	-5.674989	-1.043313
H	1.054987	-5.853609	-1.159932
C	0.384556	-4.637623	0.511506
H	-0.465753	-3.992664	0.762719

H	0.413080	-5.459424	1.236268
H	1.308186	-4.053328	0.612718
F	-1.542281	-1.492669	-3.209286
F	0.186163	-2.540560	-4.049939

TS1”

M06-L SCF energy in Solvent: -4456.300069 a.u.

M06-L Free energy in Solvent: -4455.541816 a.u.

C	2.723927	1.227677	0.390228
H	2.667368	1.648015	1.404821
H	3.529584	1.756962	-0.136747
C	3.036316	-0.264700	0.471944
H	3.314690	-0.643894	-0.522990
H	3.894426	-0.442020	1.137317
P	1.571872	-1.287745	0.978772
P	1.061265	1.585202	-0.368708
C	1.651082	4.140359	-0.966743
H	2.725979	4.035832	-0.756543
H	1.439566	5.218883	-0.958355
C	1.330524	3.505891	-2.320289
H	0.317908	3.800092	-2.628238
H	2.009458	3.862043	-3.106121
C	2.046757	-3.720214	2.029359
H	2.620243	-4.655159	2.064686
H	0.989862	-3.985935	2.185126
C	2.487457	-2.718198	3.093710
H	2.351035	-3.101664	4.113604
H	3.562453	-2.514296	2.969978
C	1.364410	1.971440	-2.199134

H	0.490873	1.540506	-2.705256
C	0.875053	3.393740	0.121074
H	1.379493	3.492917	1.093915
C	2.213537	-3.025702	0.669631
H	3.293917	-2.920354	0.480321
C	1.664701	-1.453872	2.852517
H	0.621971	-1.664375	3.133298
C	2.583767	1.251590	-2.698534
C	2.482639	-0.103973	-3.046537
C	3.842096	1.859755	-2.763222
C	3.603692	-0.823287	-3.448047
H	1.511551	-0.599296	-2.978215
C	4.965016	1.140275	-3.165465
H	3.947835	2.912914	-2.497443
C	4.851139	-0.204507	-3.508370
H	3.497185	-1.873507	-3.716474
H	5.933937	1.635374	-3.212135
H	5.728618	-0.765366	-3.825331
C	1.583072	-3.677907	-0.525703
C	0.202186	-3.903234	-0.587499
C	2.362104	-4.015571	-1.635293
C	-0.375792	-4.459435	-1.722922
H	-0.430249	-3.605349	0.249067
C	1.784801	-4.567731	-2.776660
H	3.438010	-3.833300	-1.603427
C	0.412389	-4.792764	-2.823345
H	-1.452694	-4.623440	-1.752344
H	2.410512	-4.823965	-3.630693
H	-0.042357	-5.223108	-3.714042

C	2.065497	-0.144964	3.460177
C	1.090146	0.858913	3.575001
C	3.385141	0.169667	3.799289
C	1.429997	2.140630	3.994890
H	0.057884	0.615651	3.319516
C	3.725335	1.452738	4.224071
H	4.161120	-0.592521	3.729674
C	2.752961	2.445785	4.314600
H	0.656980	2.904629	4.075513
H	4.758550	1.676475	4.485125
H	3.021810	3.447858	4.644278
C	-0.578414	3.753656	0.308966
C	-1.297954	3.098470	1.320167
C	-1.256818	4.682435	-0.483660
C	-2.649283	3.349172	1.523876
H	-0.790671	2.345528	1.926871
C	-2.613094	4.939510	-0.280334
H	-0.728086	5.216157	-1.272792
C	-3.315812	4.270554	0.716804
H	-3.185642	2.807198	2.302646
H	-3.121960	5.665005	-0.913724
H	-4.376592	4.463450	0.865056
H	-0.486456	-1.009161	-1.348346
C	-2.072289	-0.246443	0.670433
C	-2.038569	-0.793336	-0.668308
H	-2.195890	-1.872302	-0.748387
C	-2.571285	-0.020009	-1.817725
C	-2.581564	1.380970	-1.825671
C	-3.066358	-0.695657	-2.938505

C	-3.072892	2.080595	-2.922275
H	-2.185119	1.928927	-0.969564
C	-3.566864	0.003447	-4.032404
H	-3.046308	-1.787039	-2.948640
C	-3.571103	1.396138	-4.029922
H	-3.067988	3.170301	-2.905225
H	-3.951577	-0.542815	-4.892538
H	-3.958599	1.945116	-4.886577
H	-2.482551	0.755140	0.789271
C	-2.377079	-1.118056	1.827025
F	-2.020547	-0.509226	3.006255
F	-1.682207	-2.313527	1.774131
C	-3.874925	-1.400369	1.895569
O	-4.666108	-0.671588	2.451362
O	-4.187057	-2.463719	1.130994
C	-5.594259	-2.605052	0.834817
H	-6.167733	-2.364911	1.735911
H	-5.717034	-3.666237	0.600428
C	-5.971557	-1.713769	-0.322150
H	-5.791467	-0.661778	-0.073627
H	-7.033986	-1.828805	-0.564635
H	-5.386842	-1.957165	-1.216300
Cu	-0.155137	-0.259647	0.028704

7. NMR Spectra of the Related Compounds

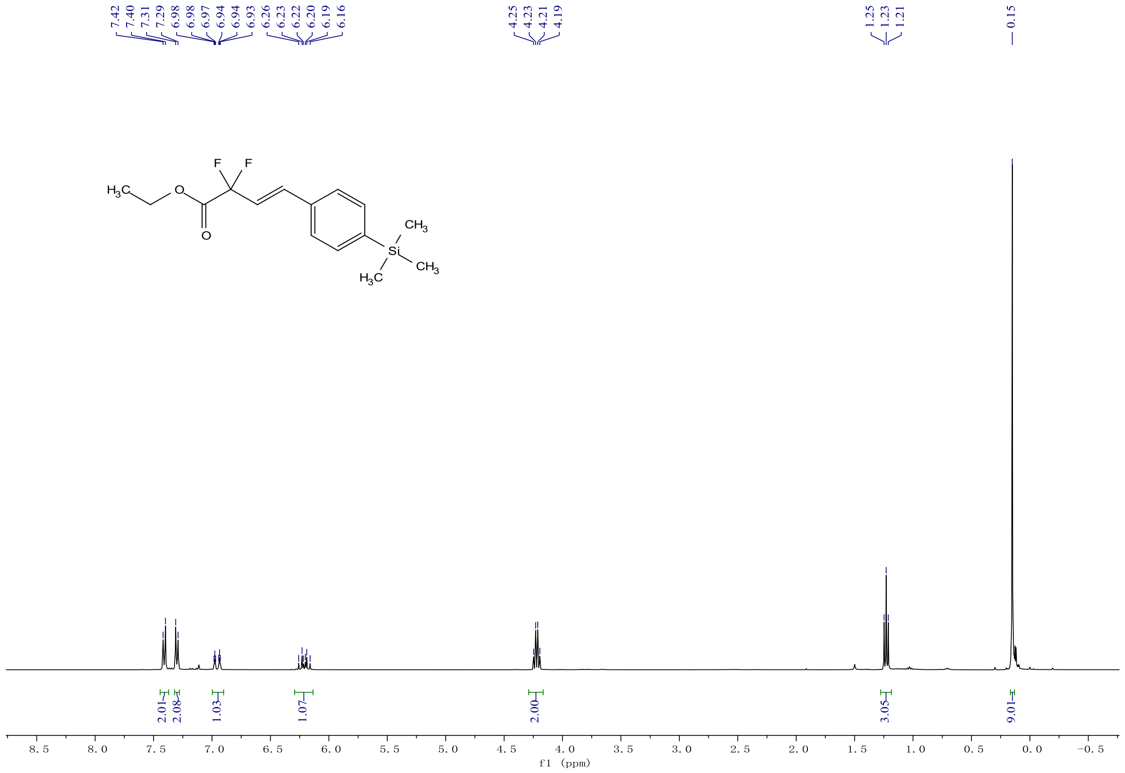

^{1}H-NMR of compound **1o** (400 MHz, $CDCl_3$)

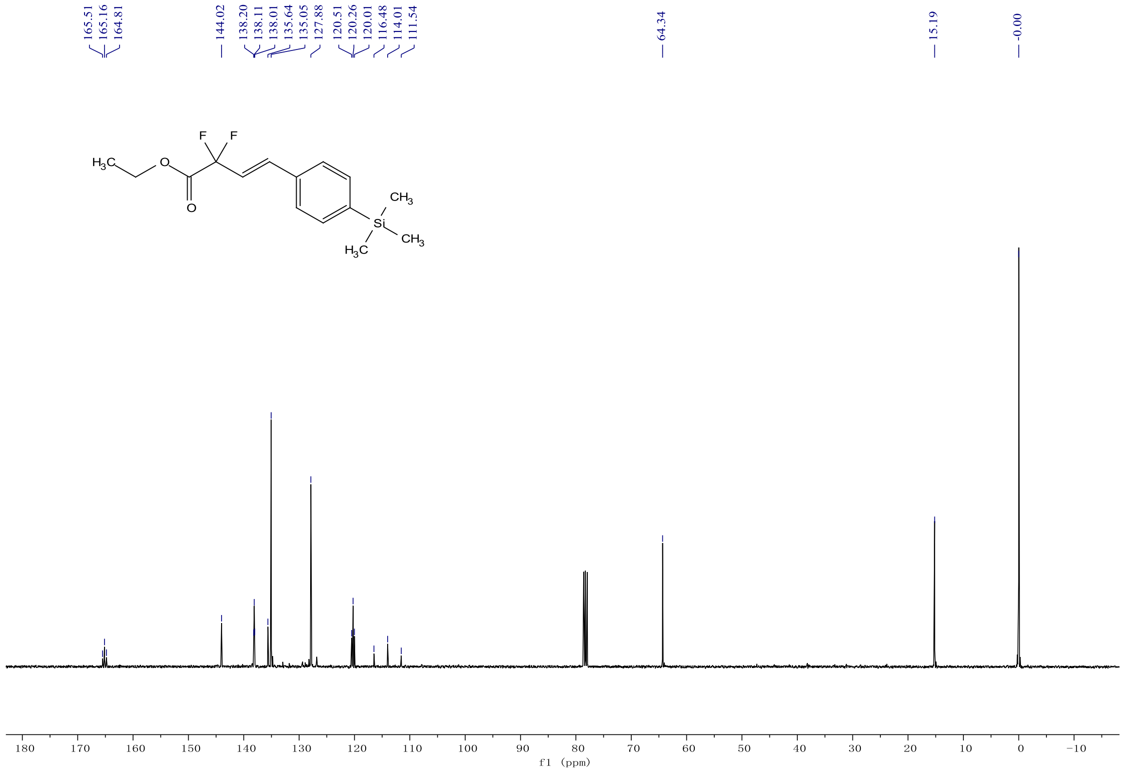

^{13}C{^{1}H}-NMR of compound **1o** (101 MHz, $CDCl_3$)

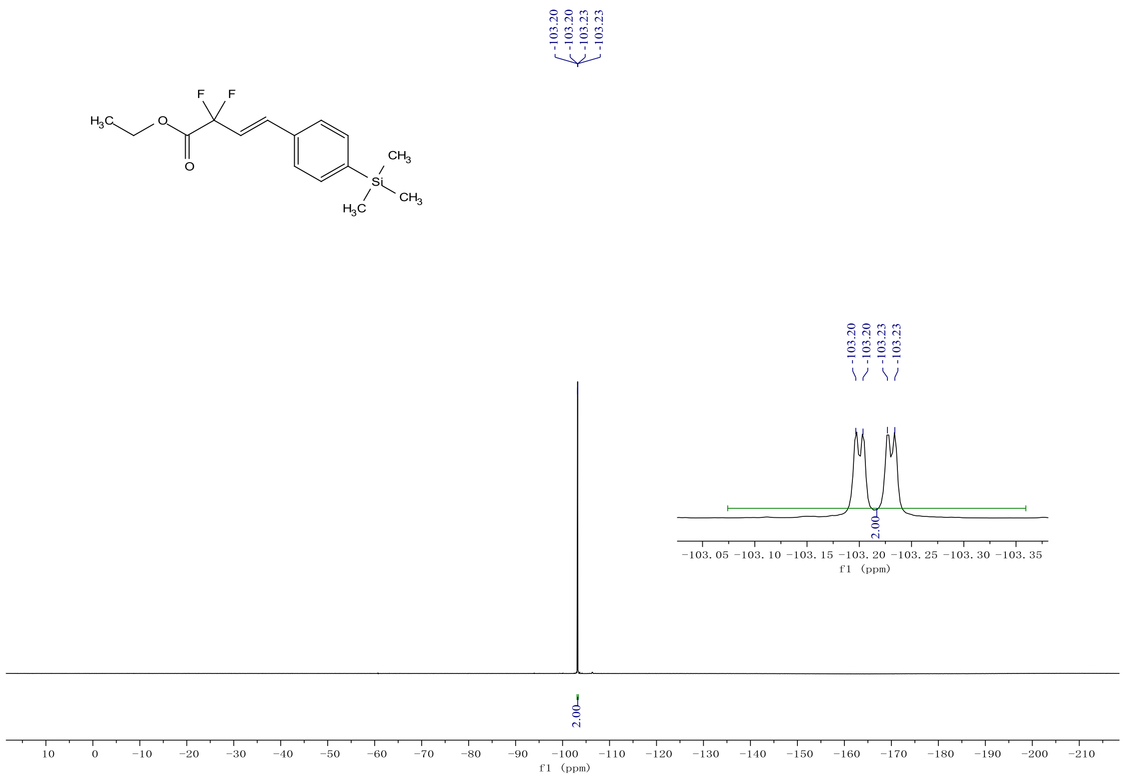

^{19}F-NMR of compound **1o** (377 MHz, $CDCl_3$)

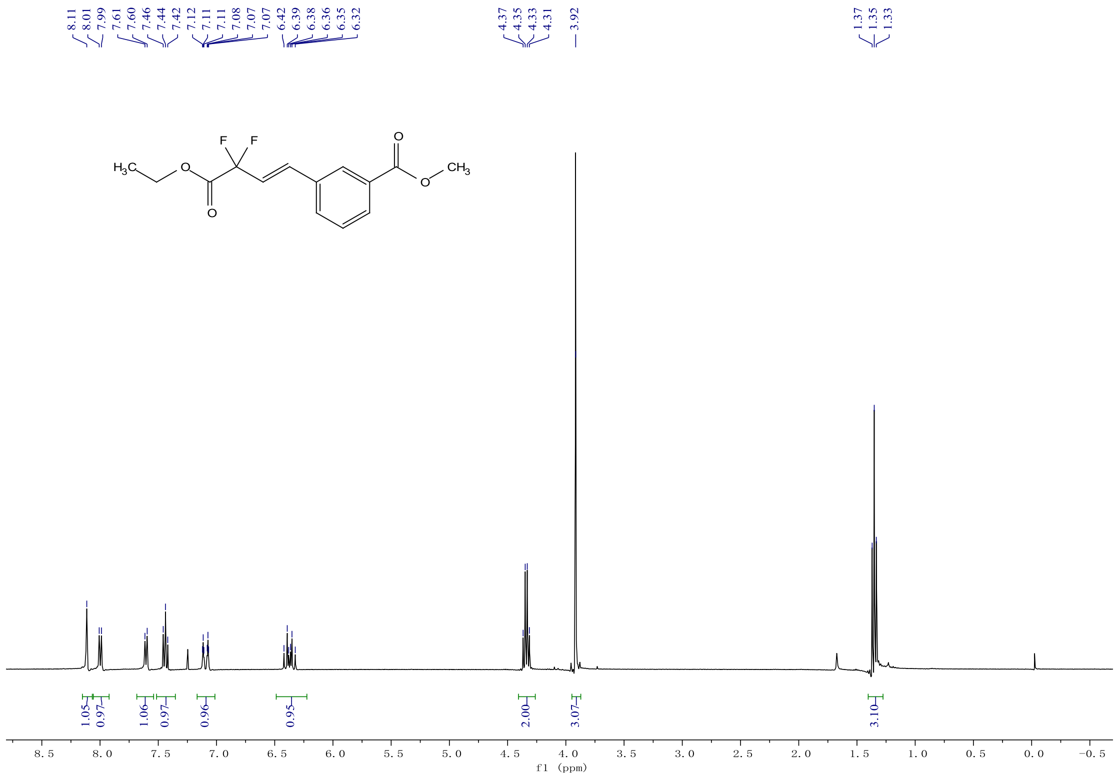

^{1}H-NMR of compound **1p** (500 MHz, $CDCl_3$)

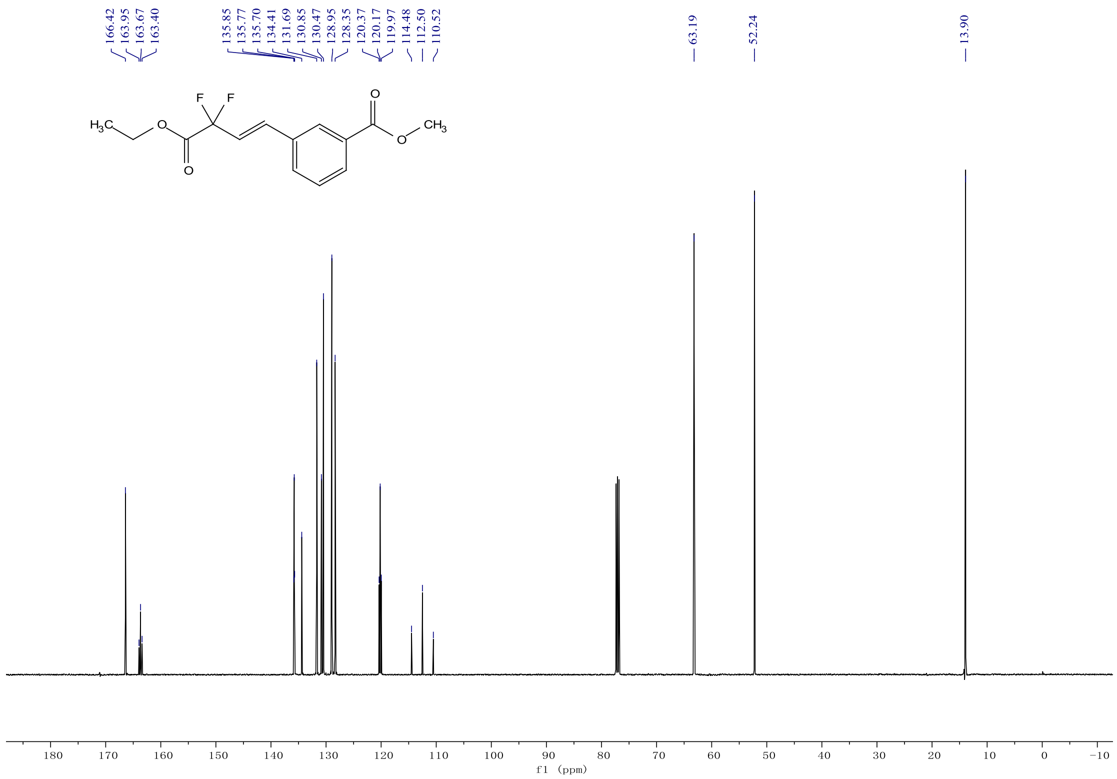

$^{13}C\{^{1}H\}$-NMR of compound **1p** (126 MHz, $CDCl_3$)

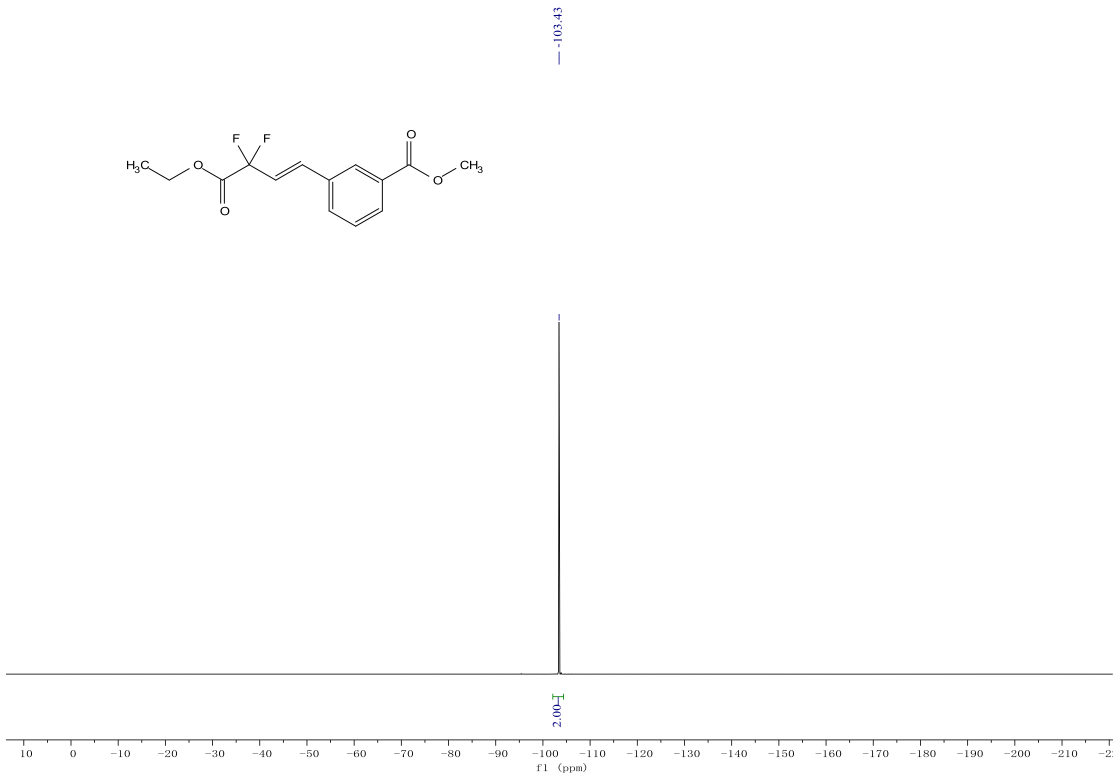

$^{19}F\{^{1}H\}$-NMR of compound **1p** (470 MHz, $CDCl_3$)

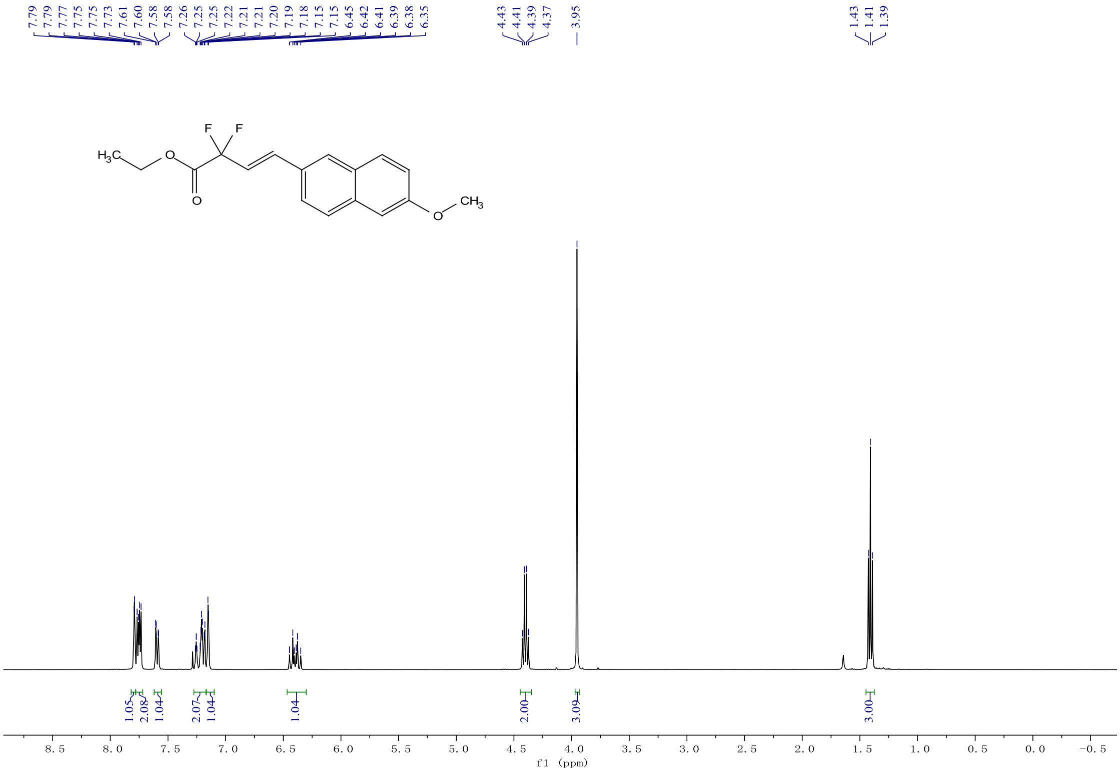

^{1}H-NMR of compound **1q** (400 MHz, $CDCl_3$)

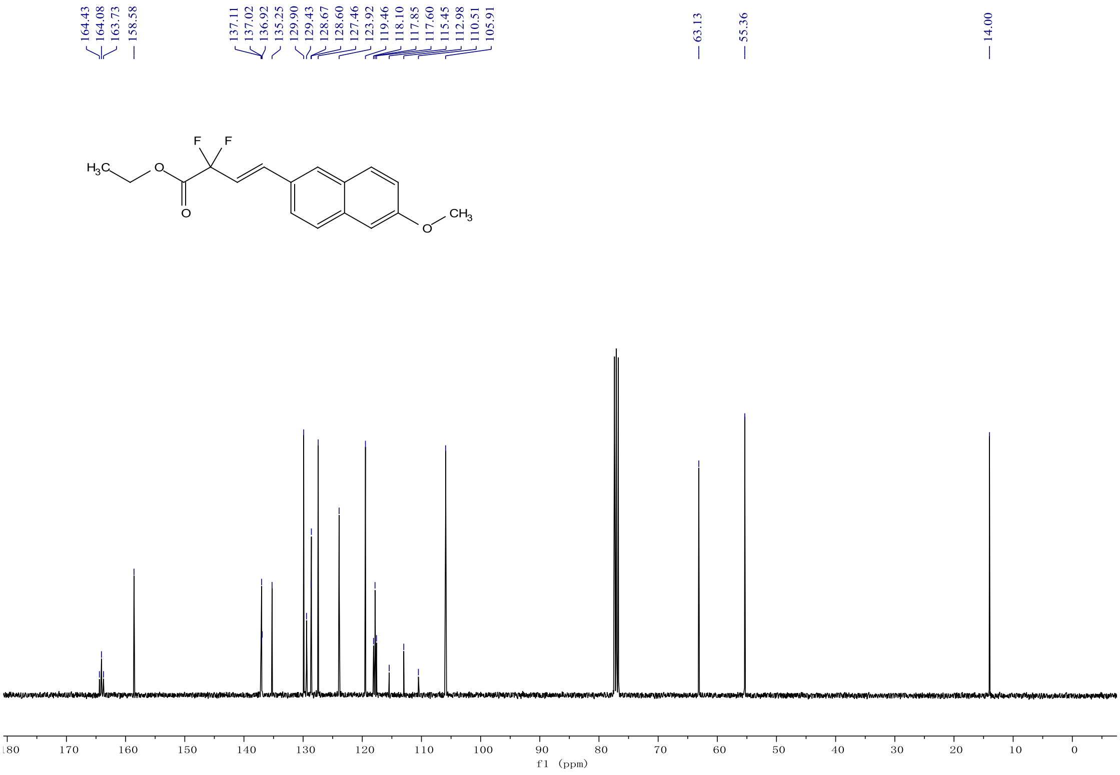

$^{13}C\{^{1}H\}$-NMR of compound **1q** (101 MHz, $CDCl_3$)

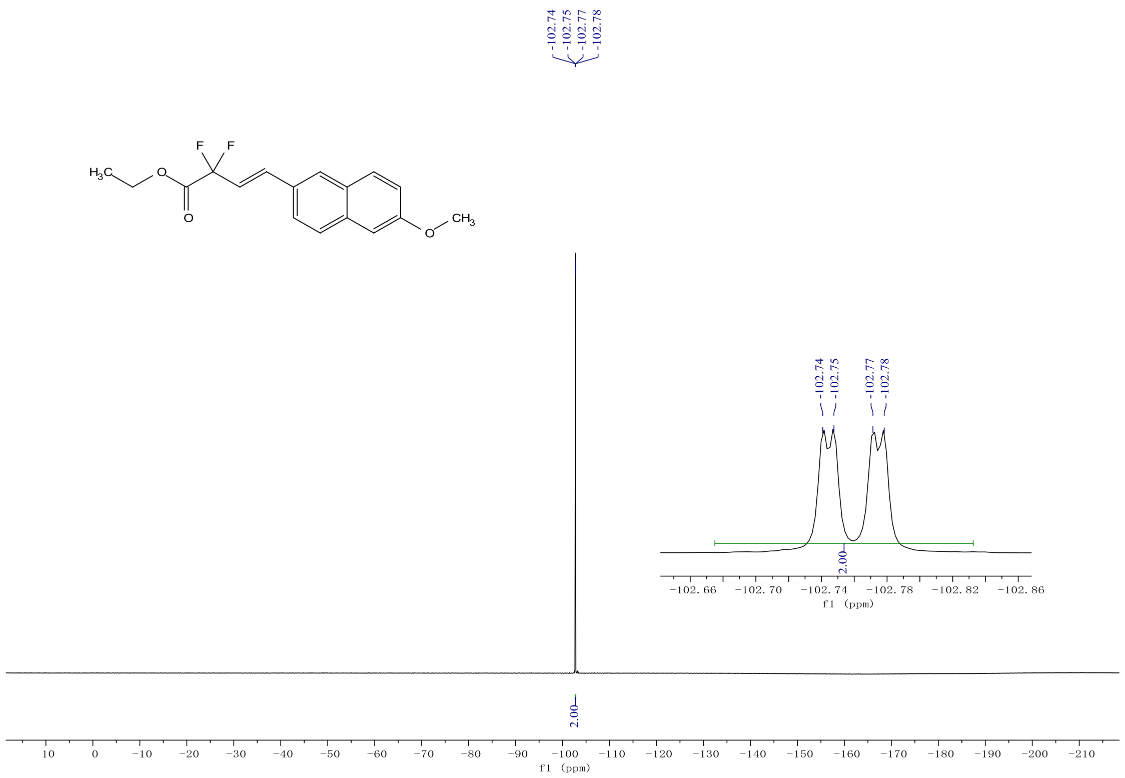

^{19}F-NMR of compound **1q** (377 MHz, $CDCl_3$)

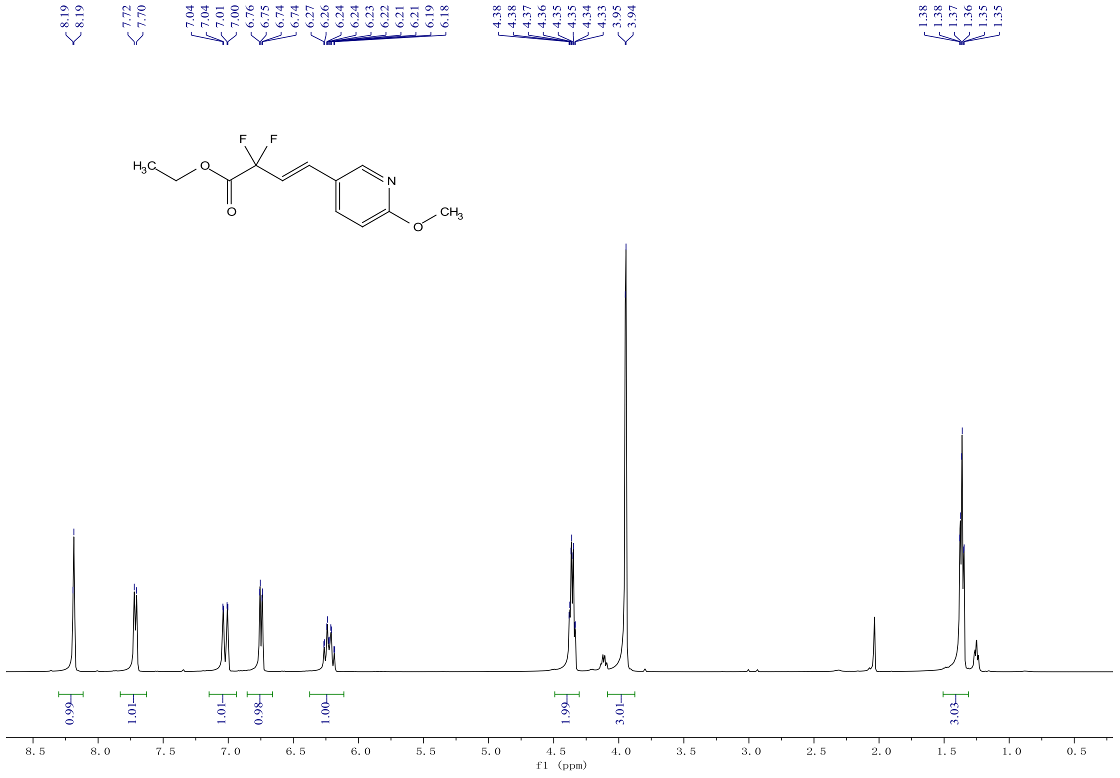

^{1}H-NMR of compound **1s** (500 MHz, $CDCl_3$)

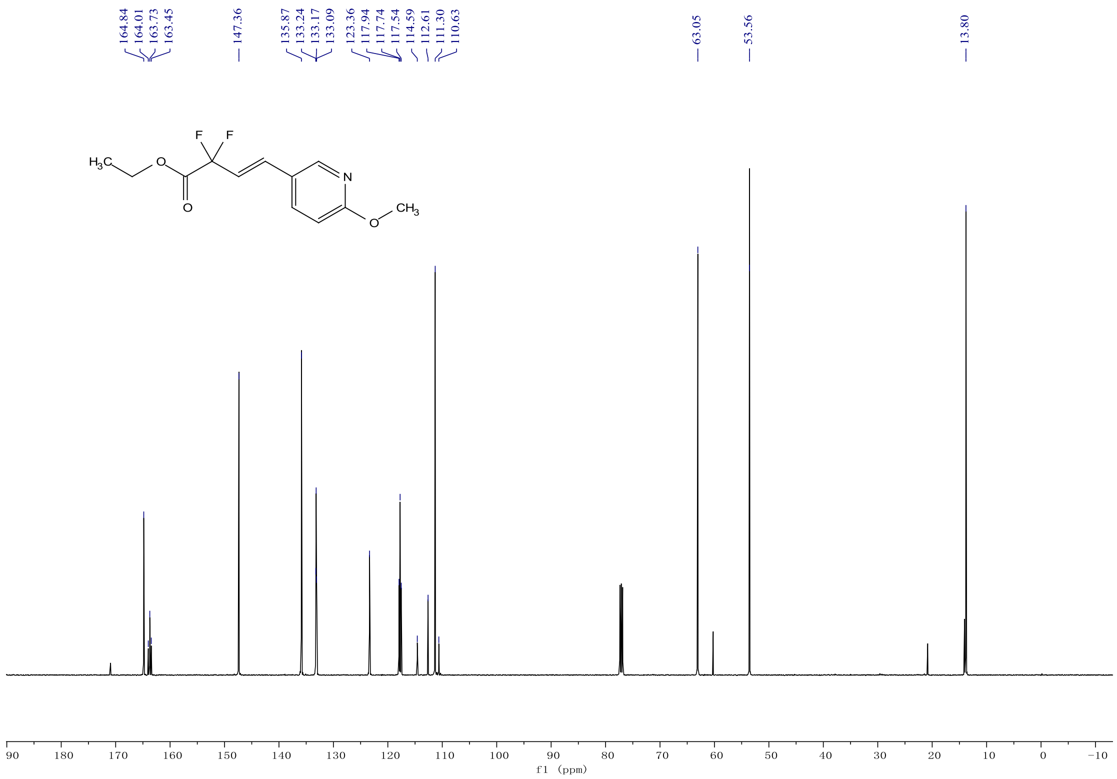

$^{13}C\{^{1}H\}$-NMR of compound **1s** (126 MHz, $CDCl_3$)

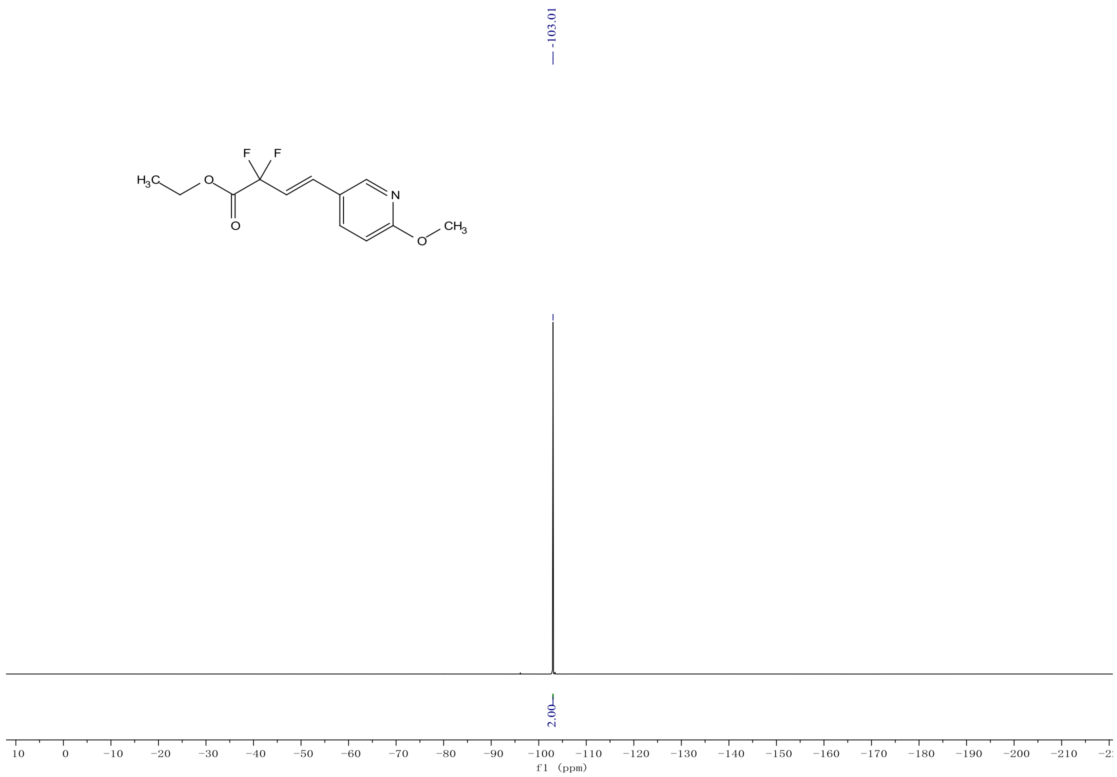

$^{19}F\{^{1}H\}$-NMR of compound **1s** (470 MHz, $CDCl_3$)

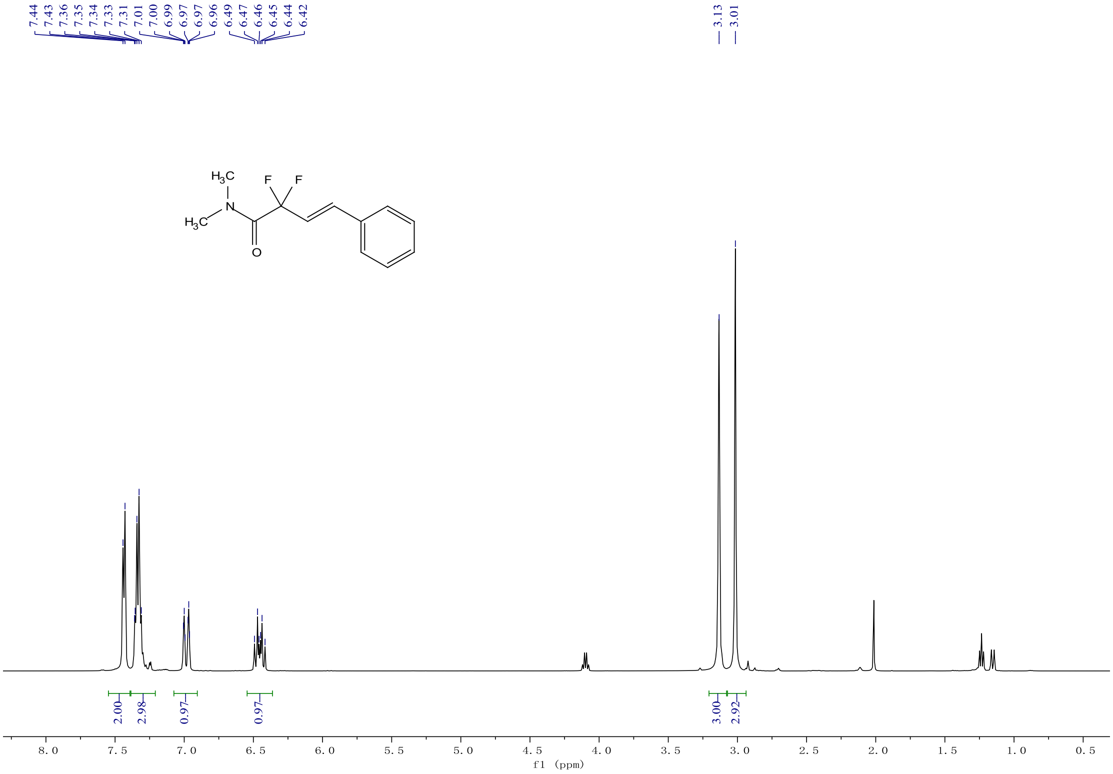

^{1}H-NMR of compound **3b** (500MHz, $CDCl_3$)

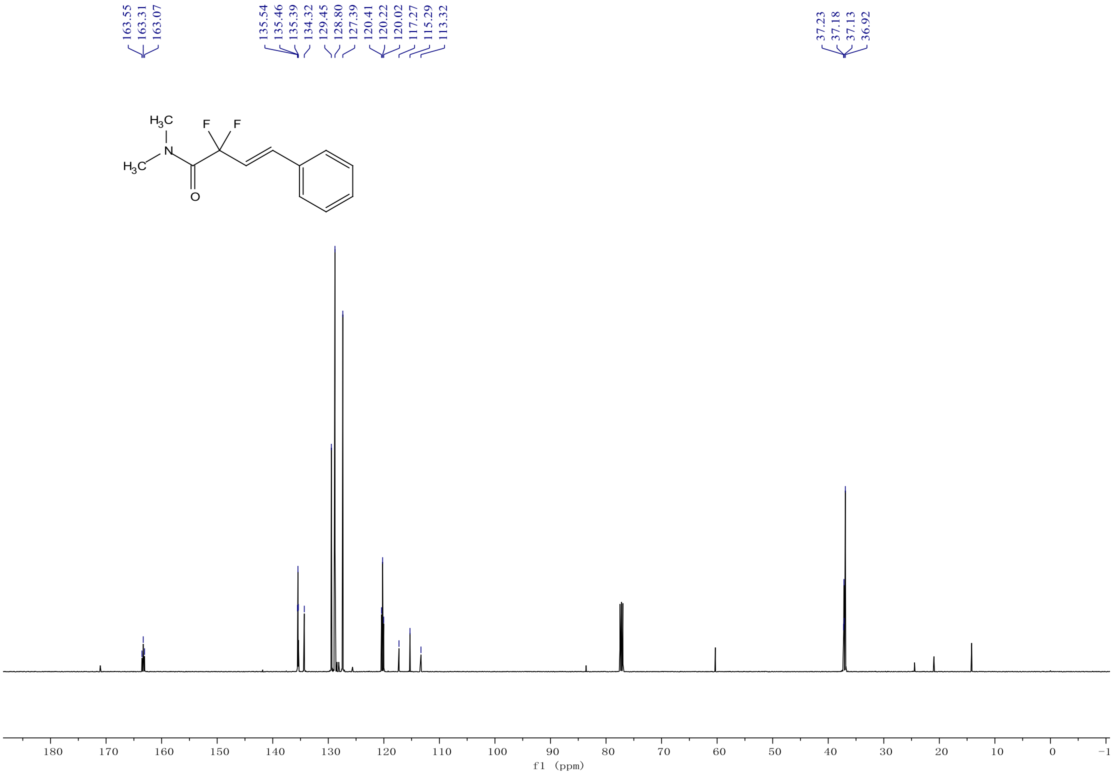

^{13}C{^{1}H}-NMR of compound **3b** (126MHz, $CDCl_3$)

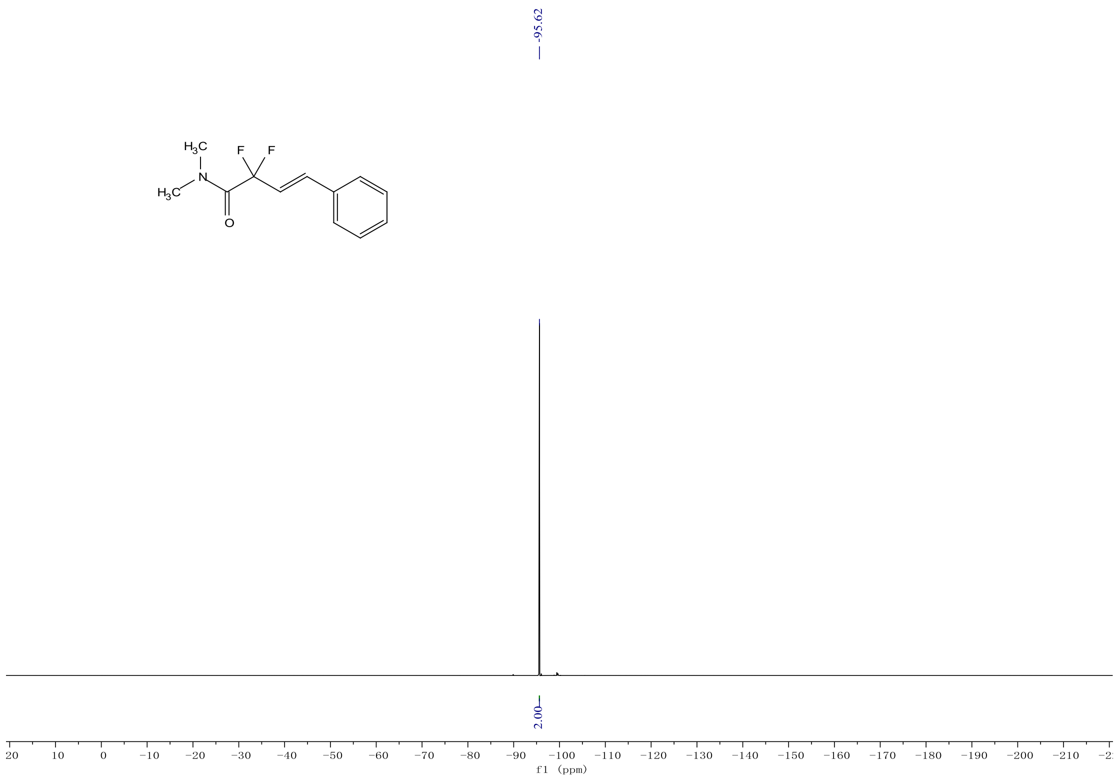

$^{19}F\{^{1}H\}$-NMR of compound **3b** (470MHz, $CDCl_3$)

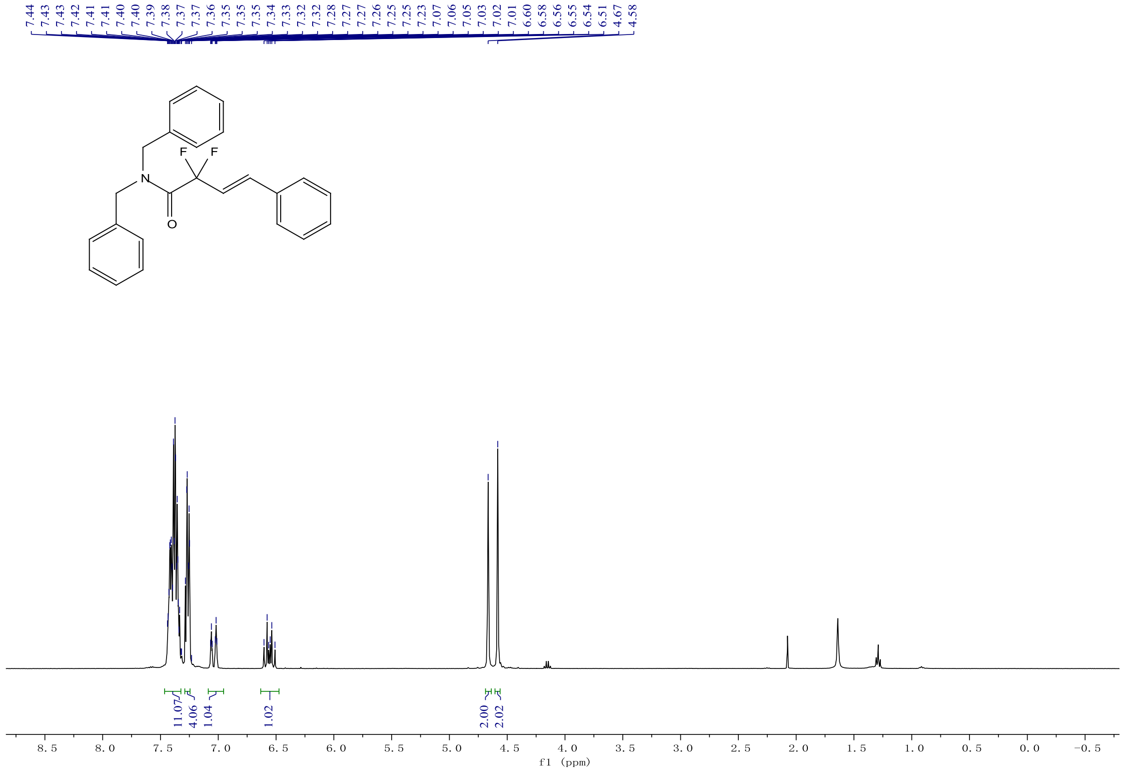

^{1}H-NMR of compound **3c** (400MHz, $CDCl_3$)

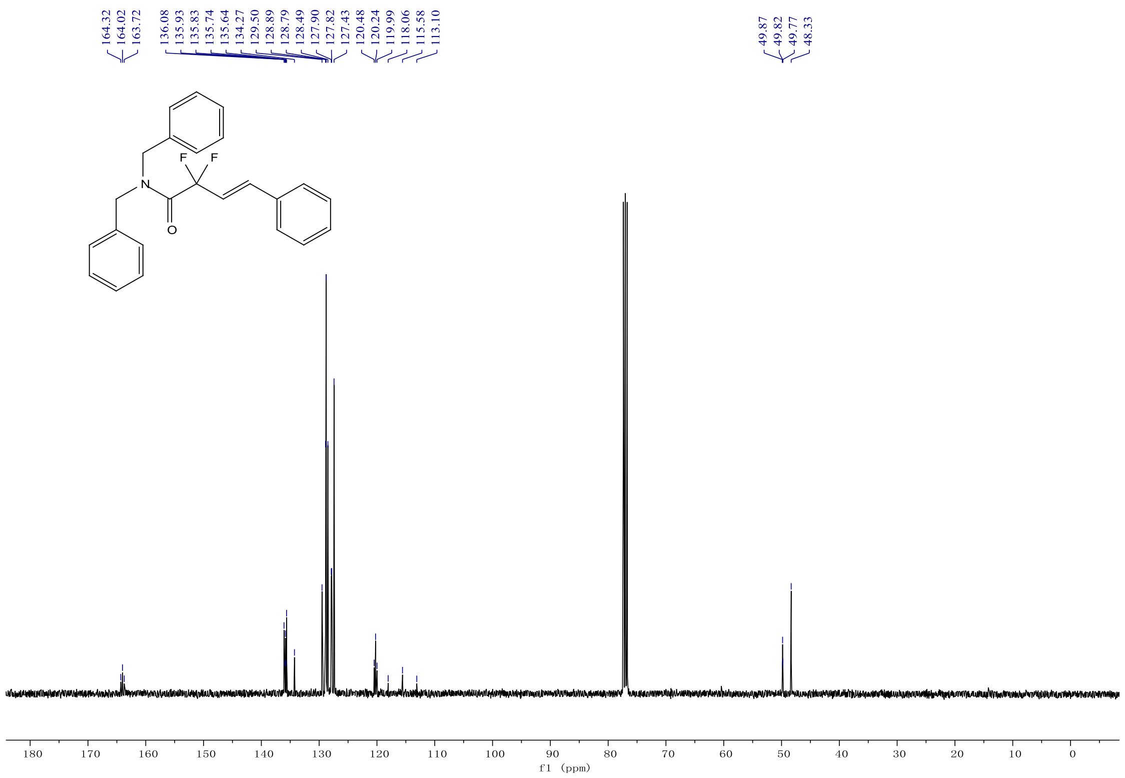

$^{13}C\{^{1}H\}$-NMR of compound **3c** (101MHz, $CDCl_3$)

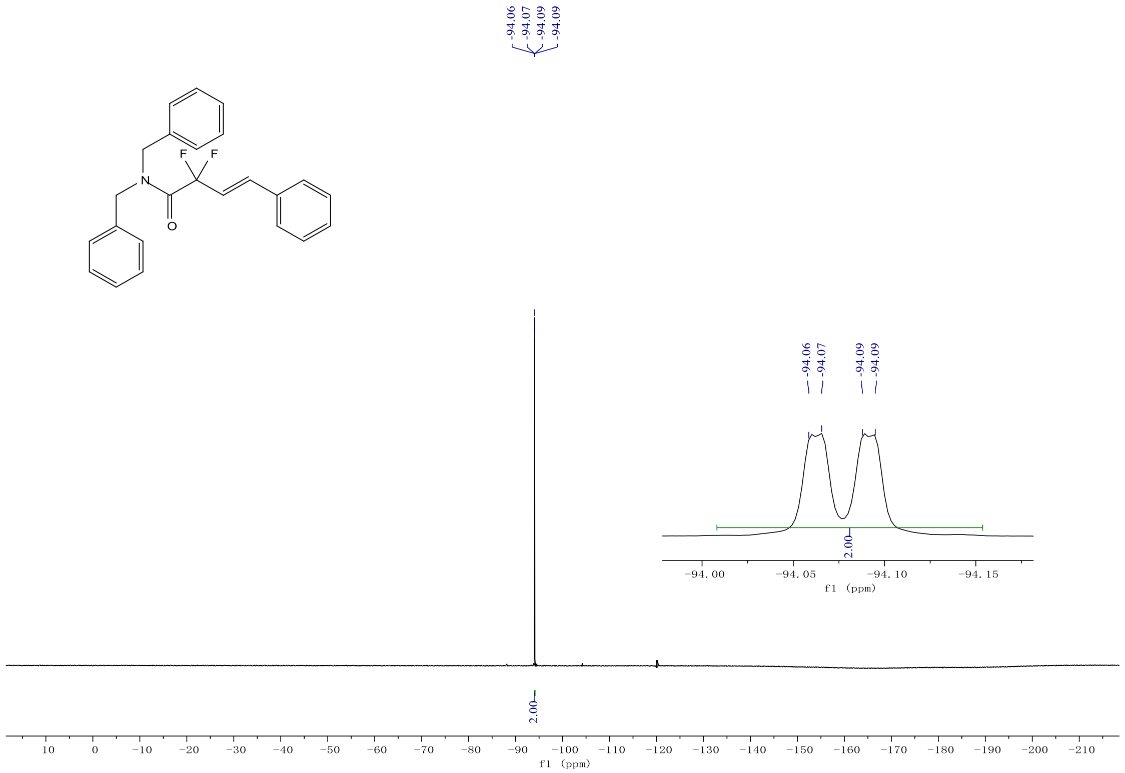

^{19}F-NMR of compound **3c** (377MHz, $CDCl_3$)

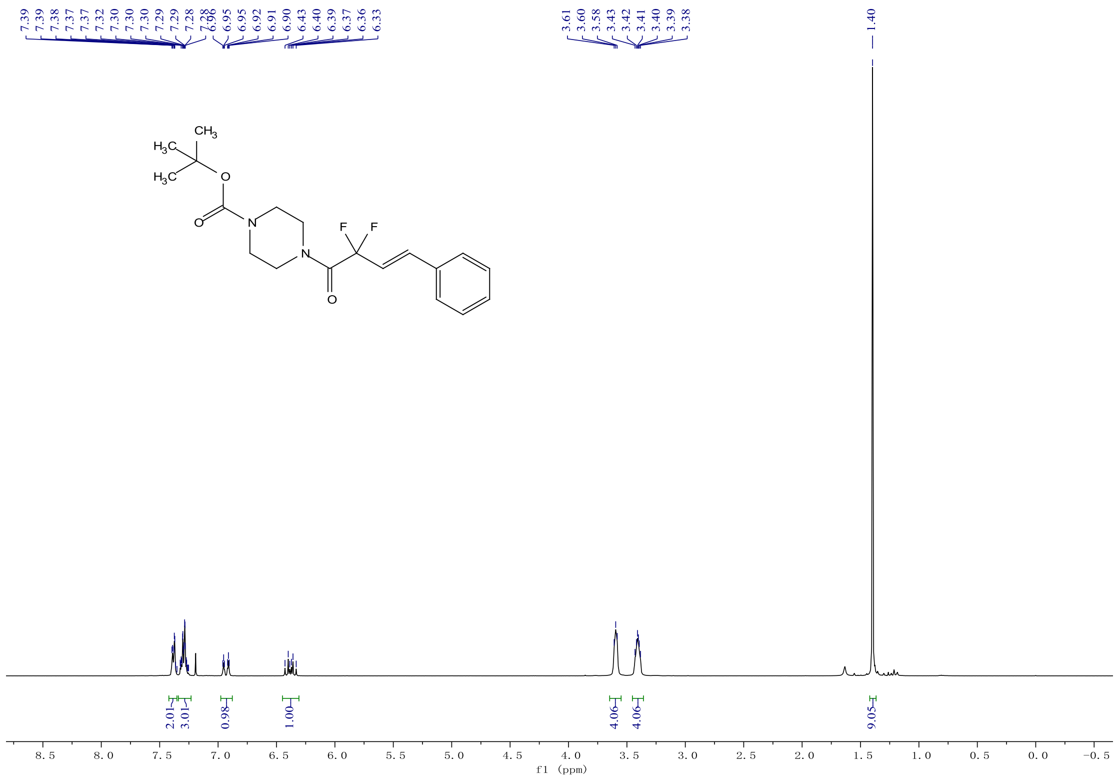

^{1}H-NMR of compound **3g** (400MHz, $CDCl_3$)

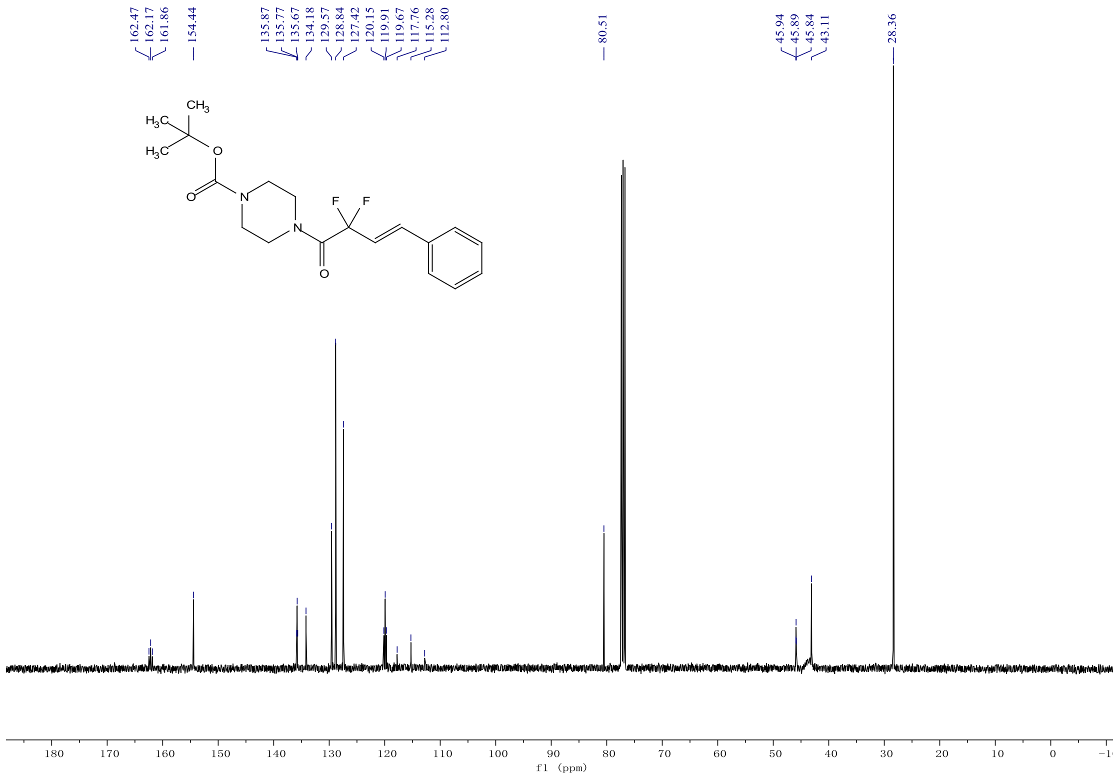

^{13}C{^{1}H}-NMR of compound **3g** (101MHz, $CDCl_3$)

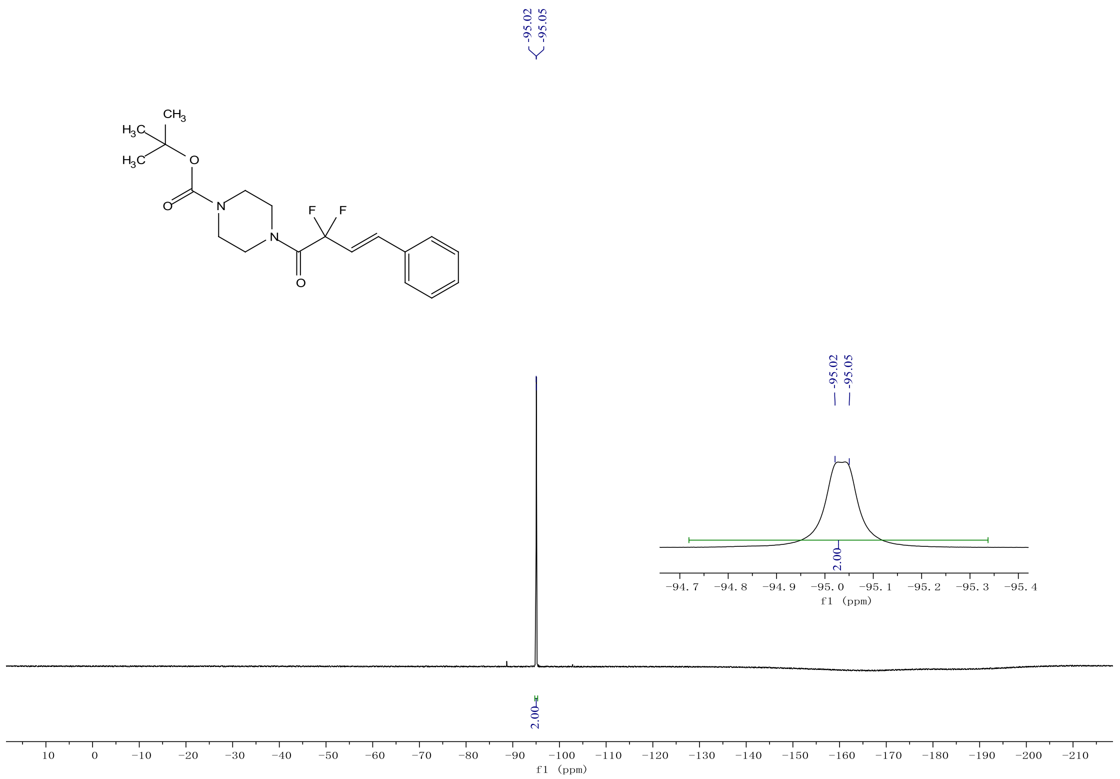

^{19}F-NMR of compound **3g** (377MHz, $CDCl_3$)

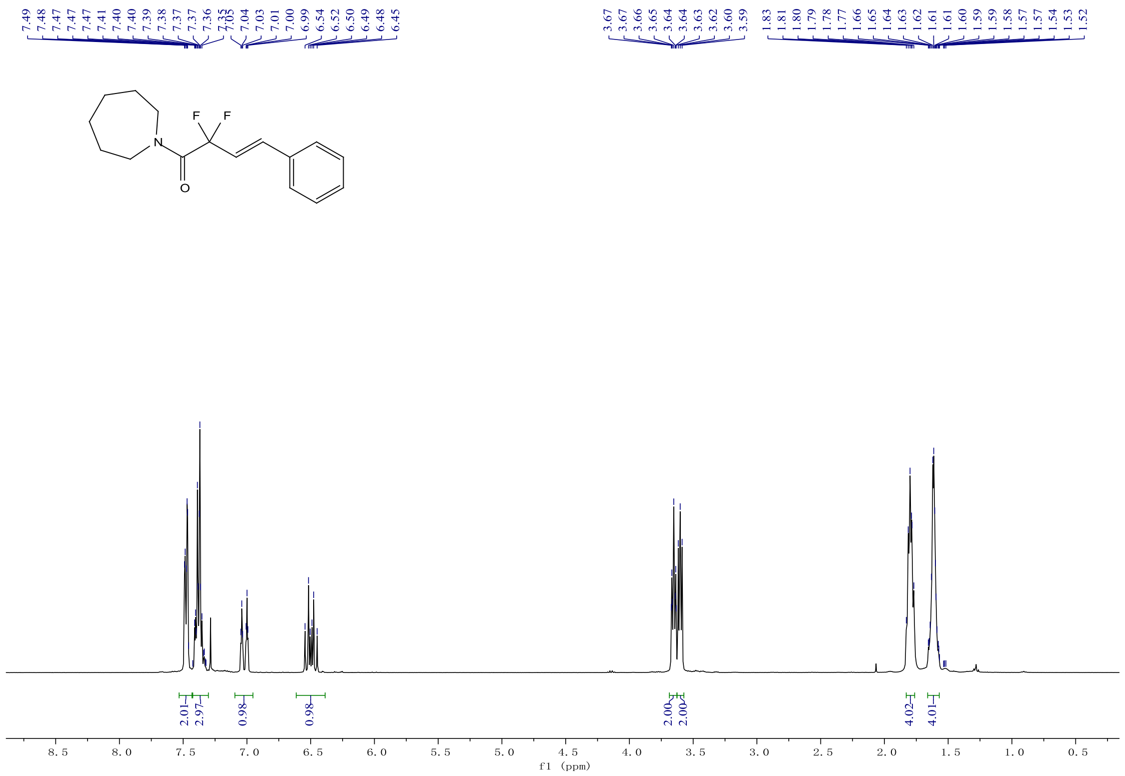

^{1}H-NMR of compound **3h** (400MHz, $CDCl_3$)

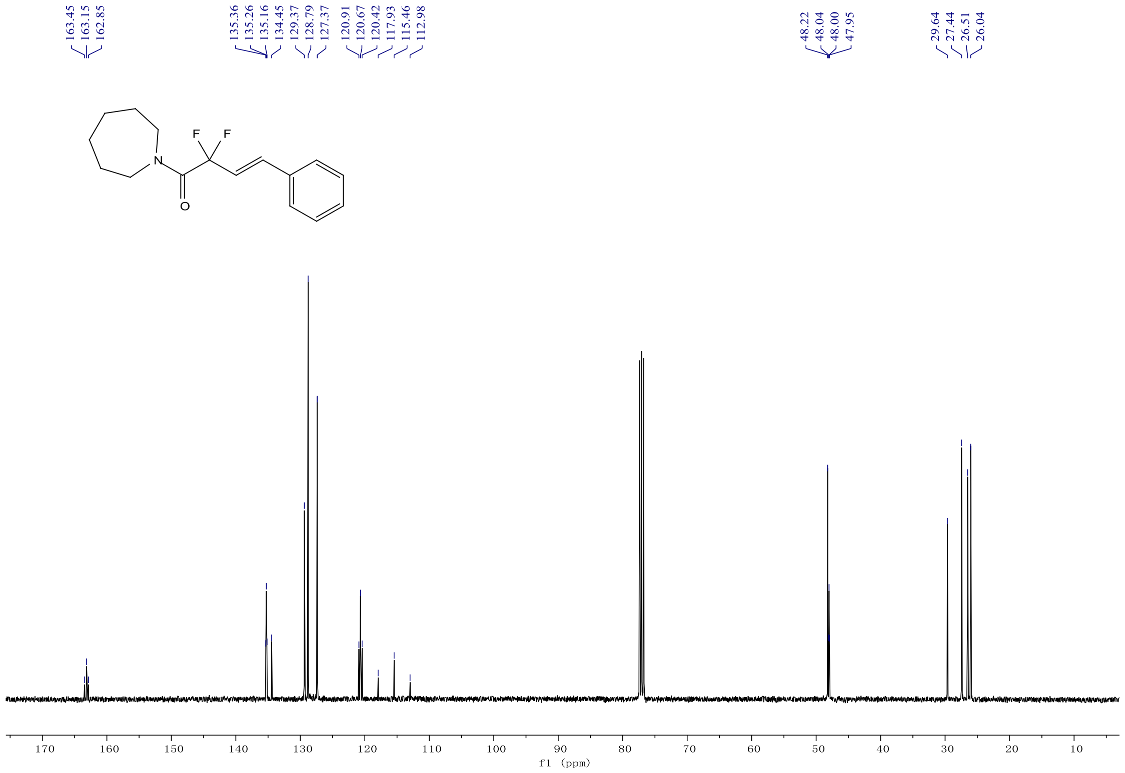

$^{13}C\{^{1}H\}$-NMR of compound **3h** (101MHz, $CDCl_3$)

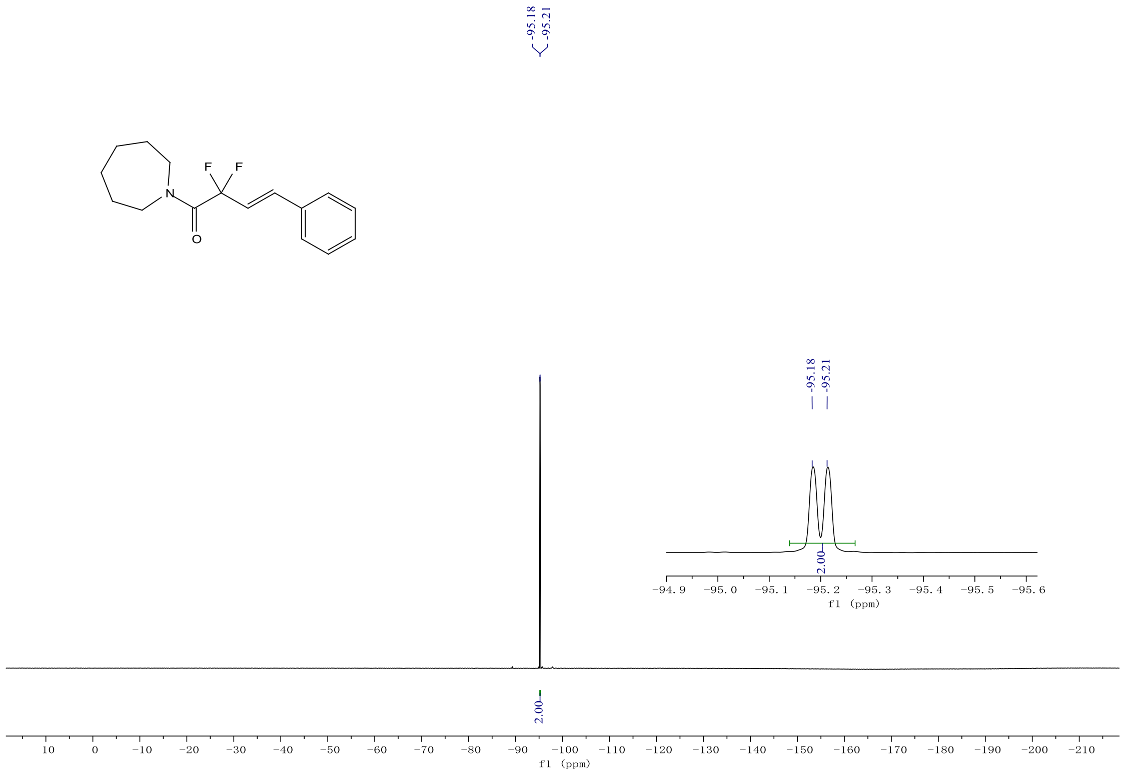

^{19}F-NMR of compound **3h** (377MHz, $CDCl_3$)

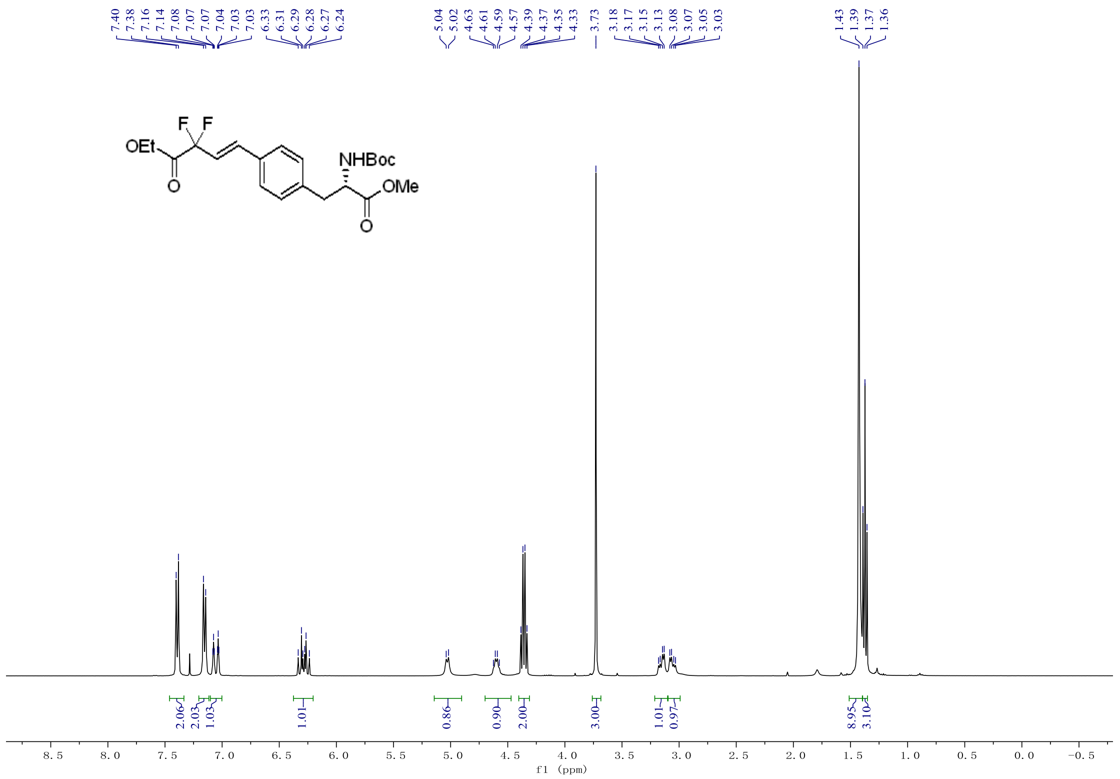

^{1}H-NMR of compound **5a** (400MHz, $CDCl_3$)

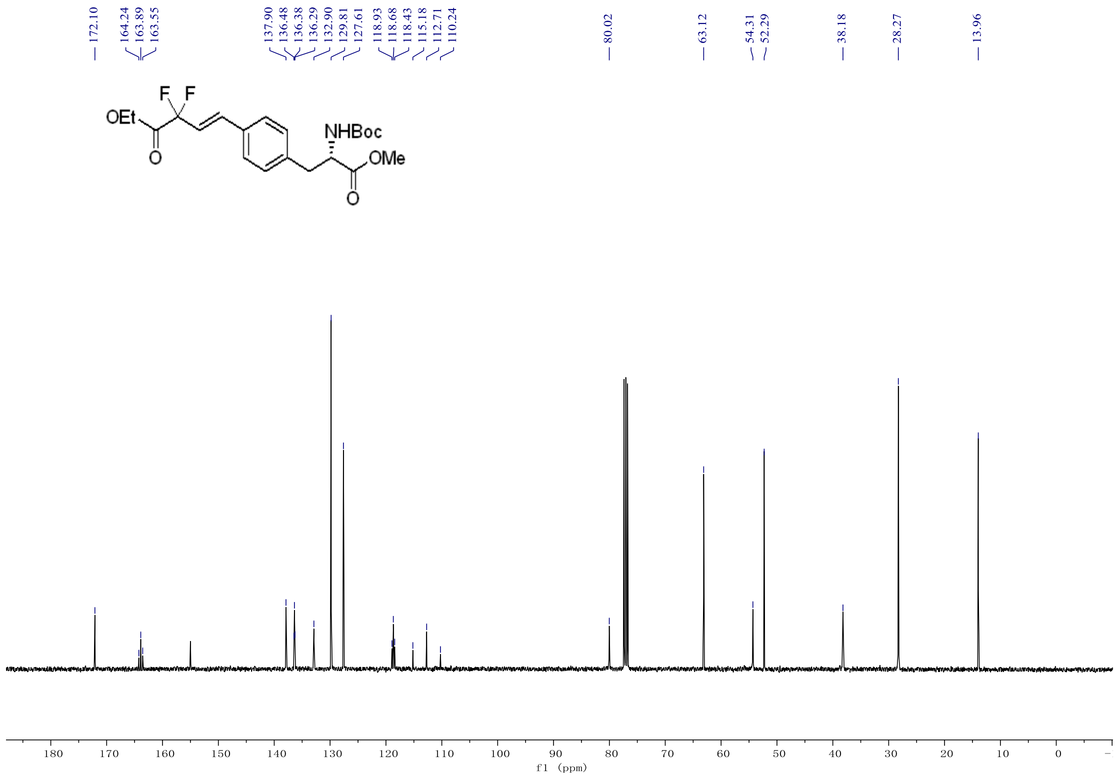

^{13}C{^{1}H}-NMR of compound **5a** (101MHz, $CDCl_3$)

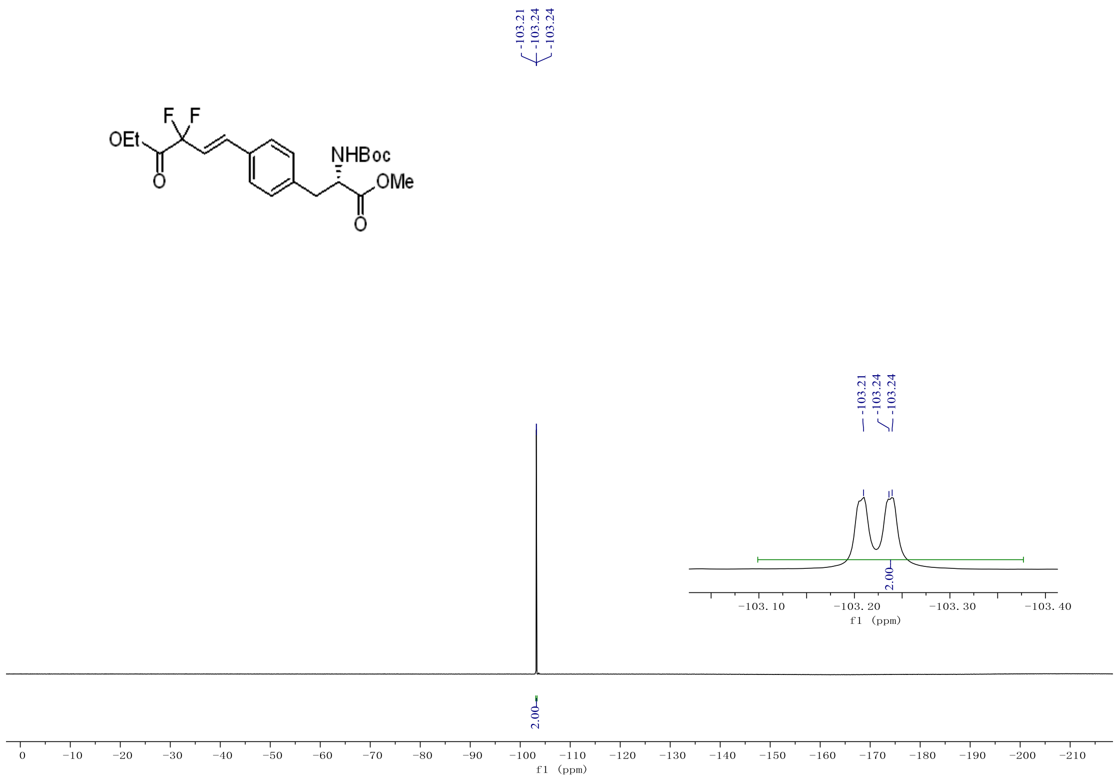

^{19}F-NMR of compound **5a** (377MHz, $CDCl_3$)

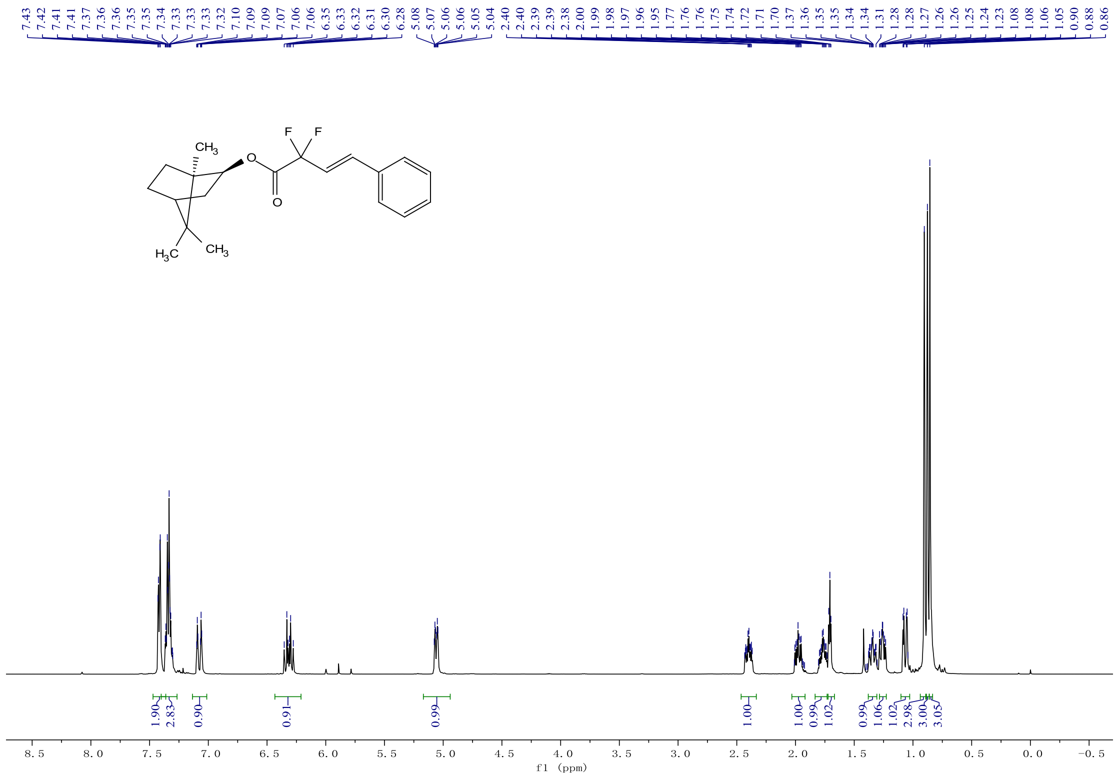

^{1}H-NMR of compound **5b** (500MHz, $CDCl_3$)

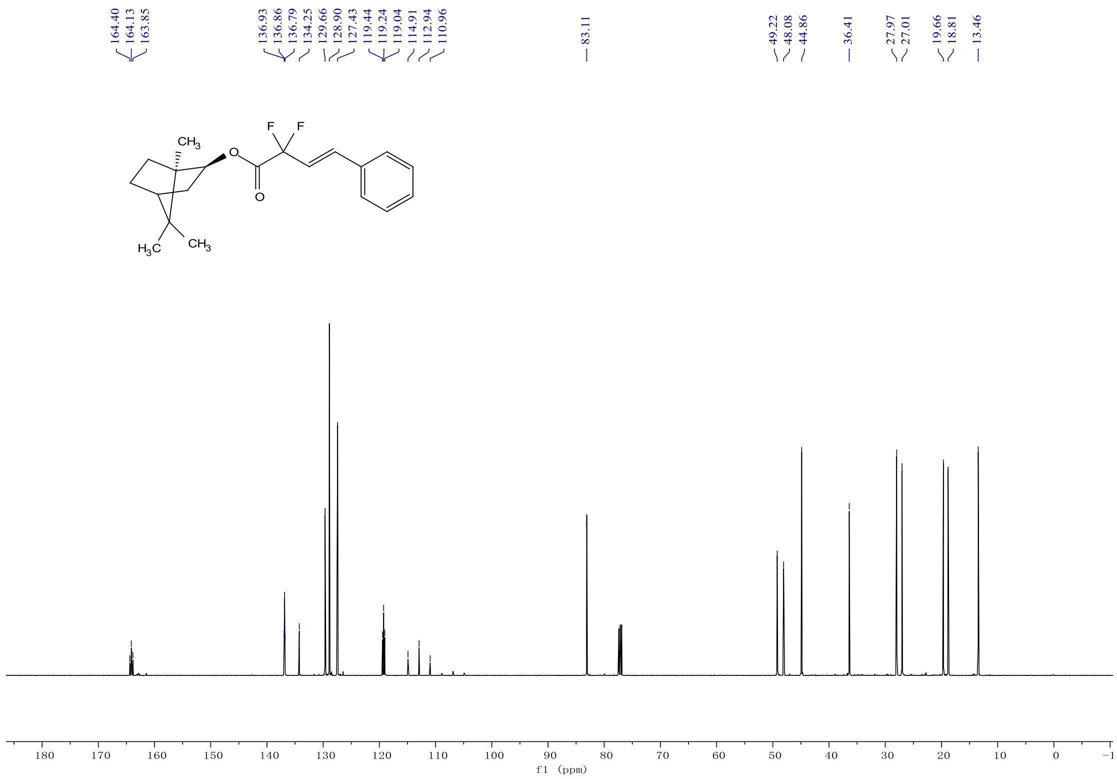

$^{13}C\{^{1}H\}$-NMR of compound **5b** (126MHz, $CDCl_3$)

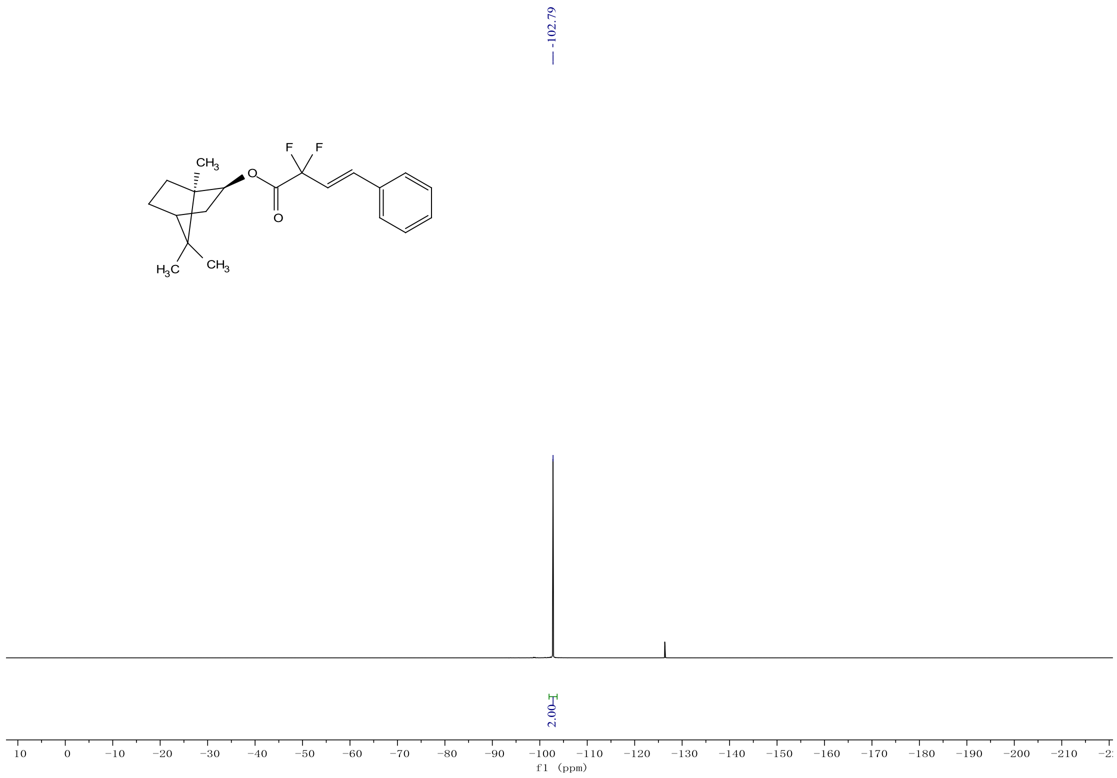

$^{19}F\{^{1}H\}$-NMR of compound **5b** (377MHz, $CDCl_3$)

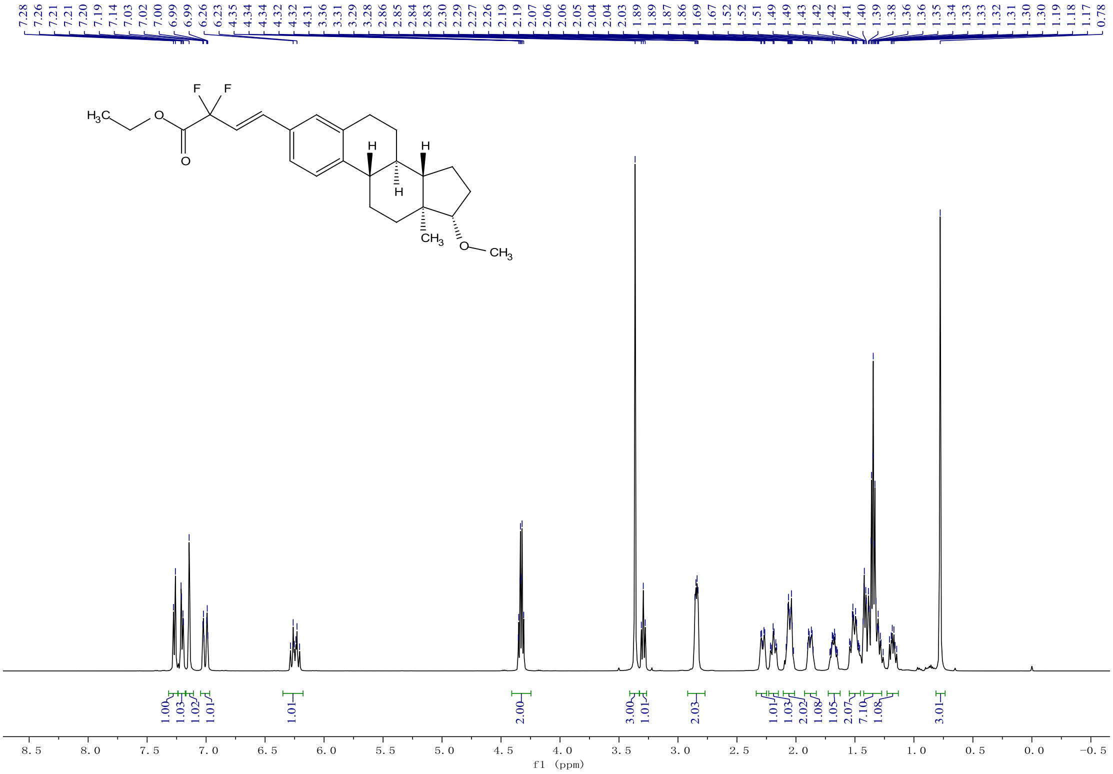

^{1}H-NMR of compound **5c** (500MHz, $CDCl_3$)

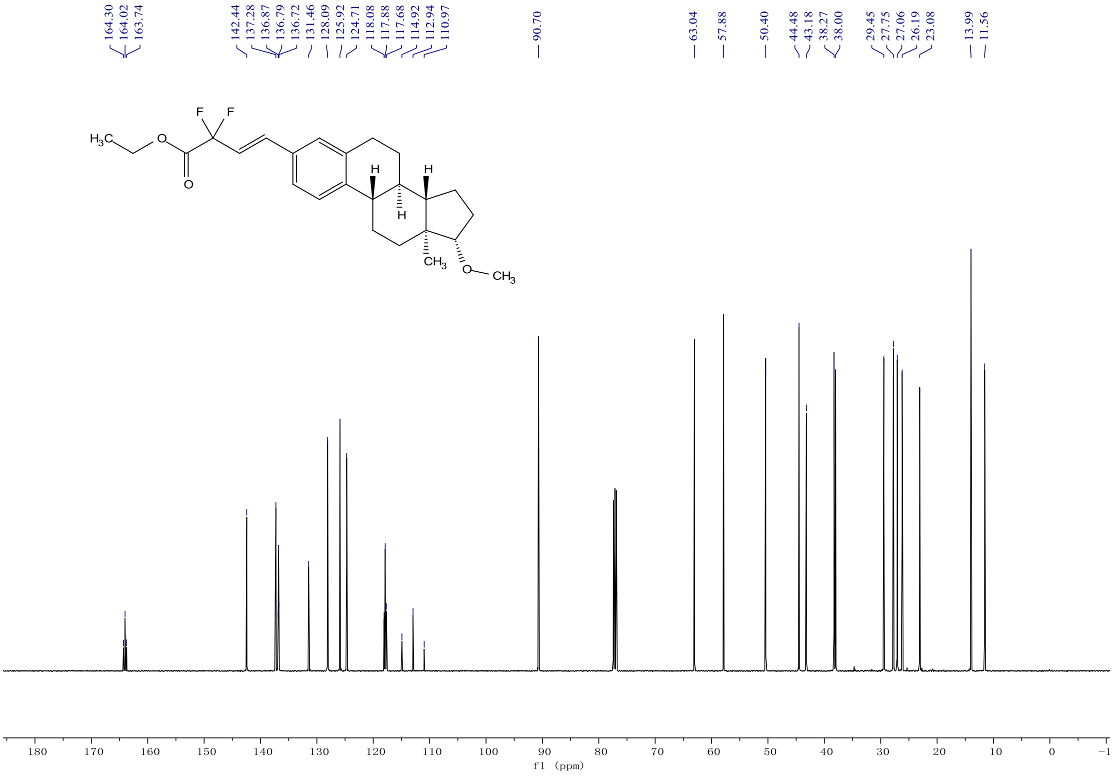

^{13}C{^{1}H}-NMR of compound **5c** (126MHz, $CDCl_3$)

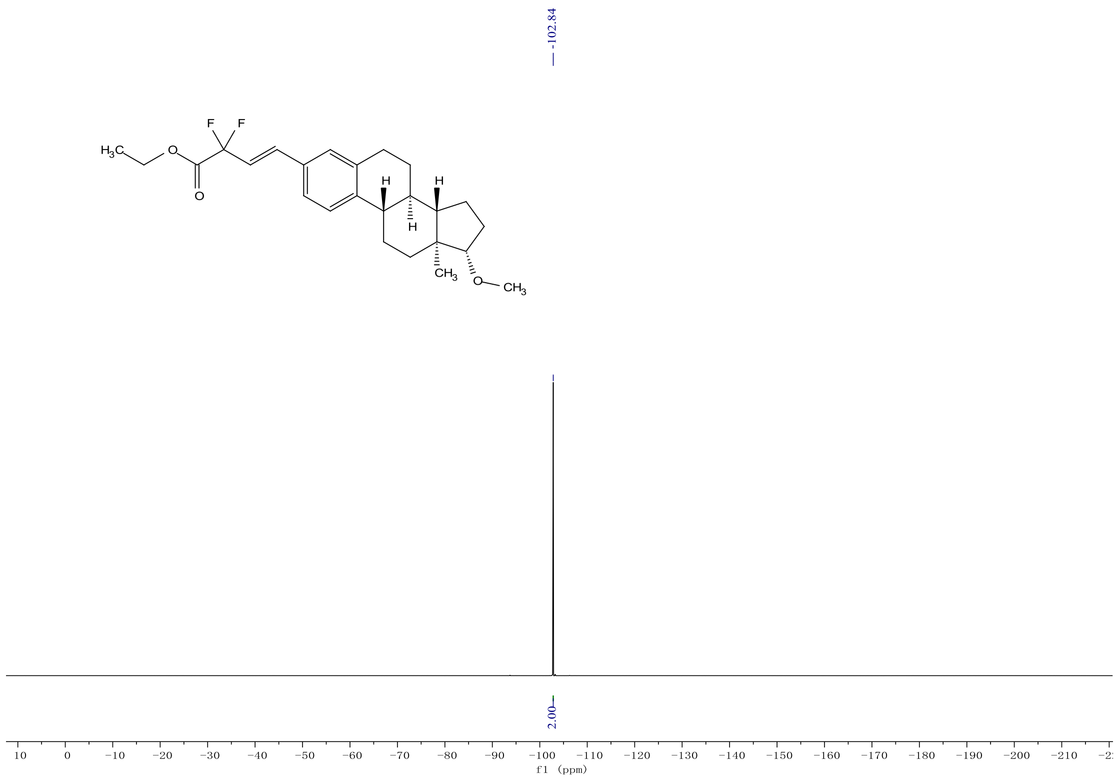

$^{19}F\{^{1}H\}$-NMR of compound **5c** (470MHz, $CDCl_3$)

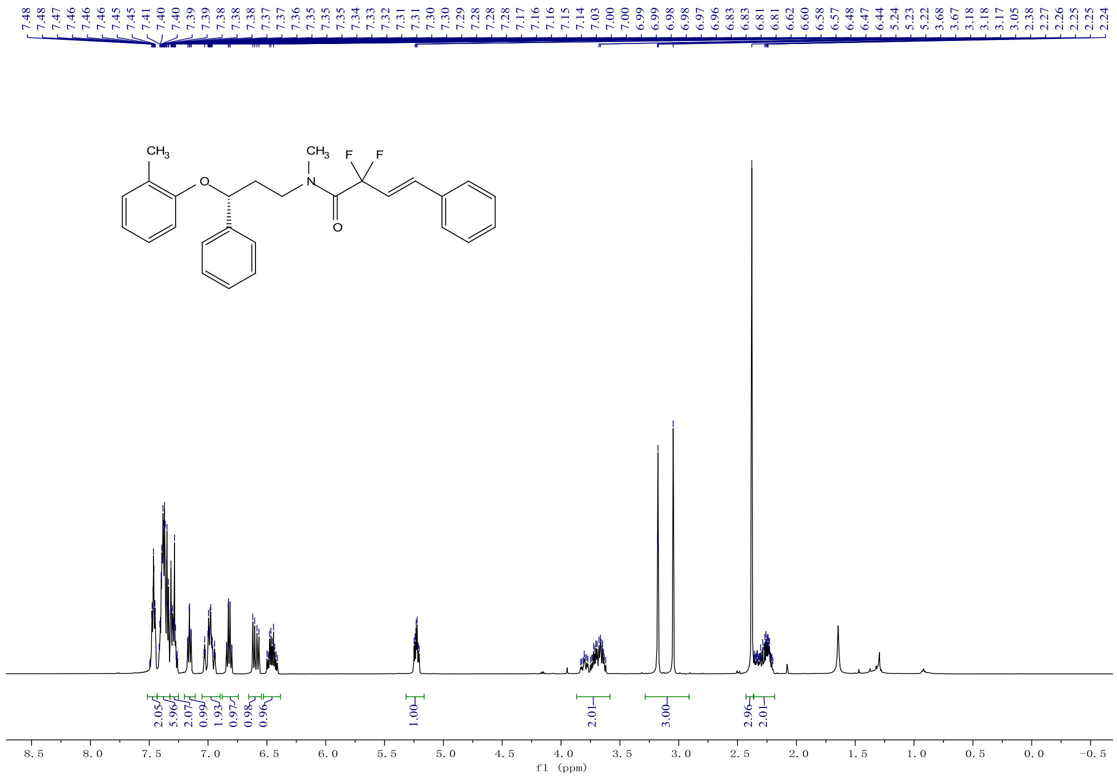

^{1}H-NMR of compound **5d** (400MHz, $CDCl_3$)

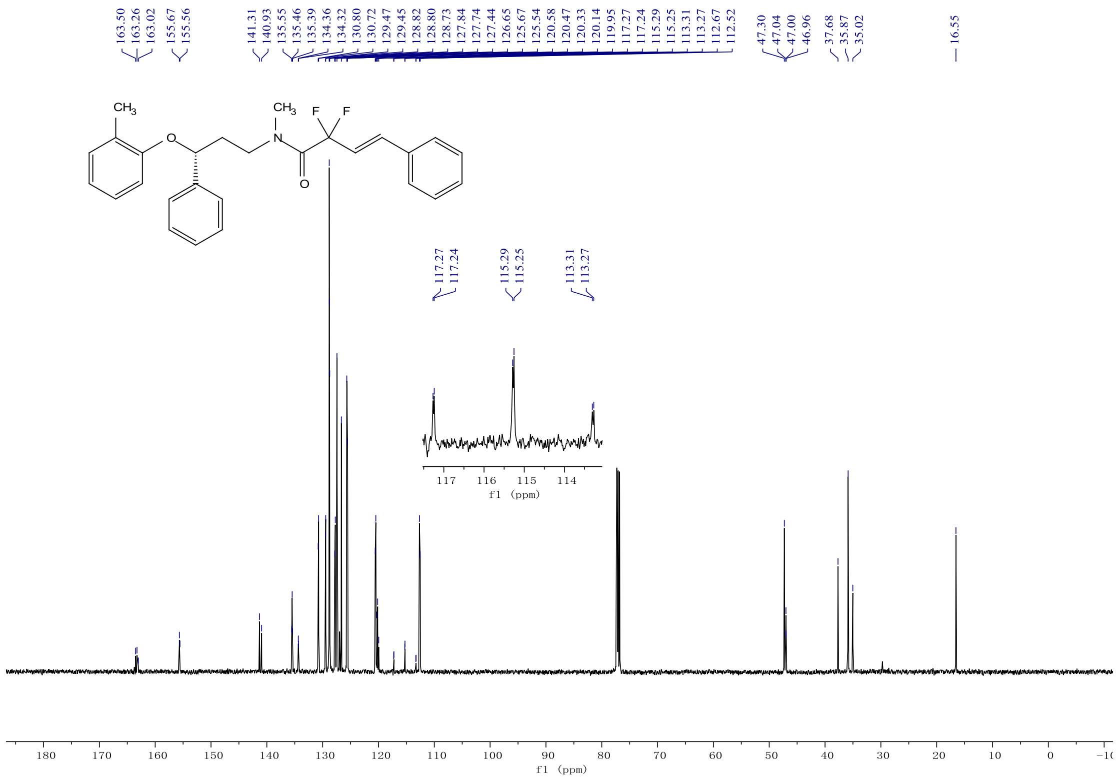

$^{13}C\{^{1}H\}$-NMR of compound **5d** (101MHz, $CDCl_3$)

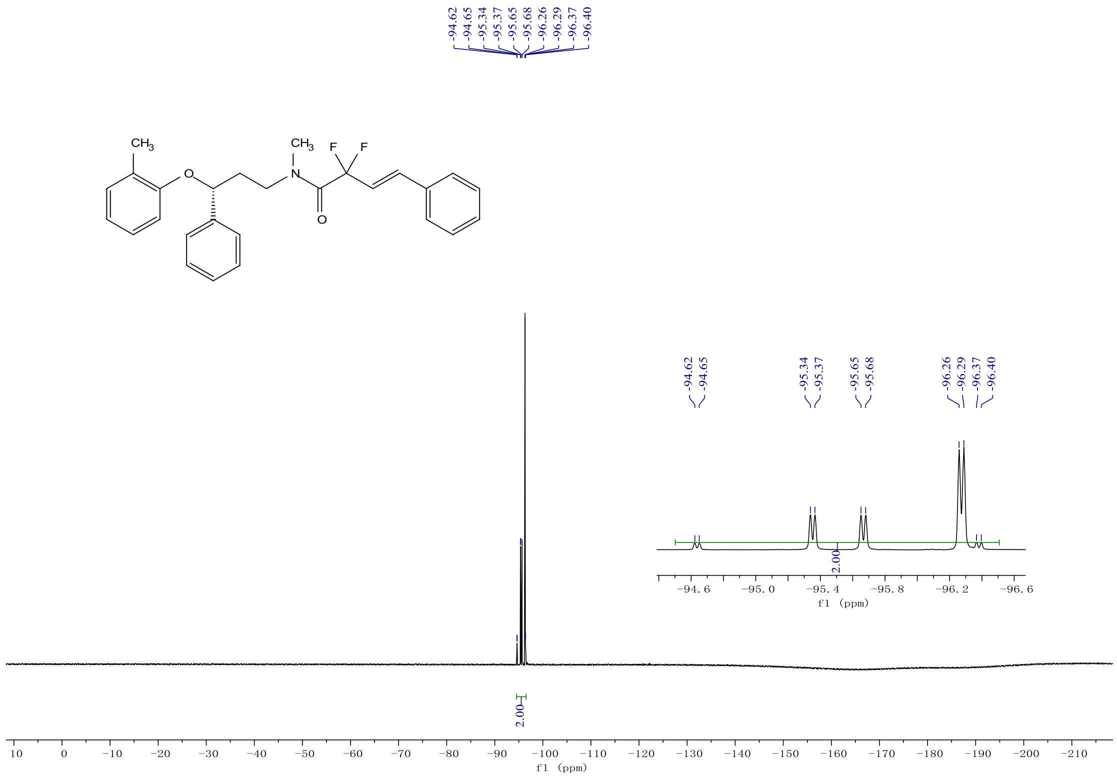

^{19}F-NMR of compound **5d** (377MHz, $CDCl_3$)

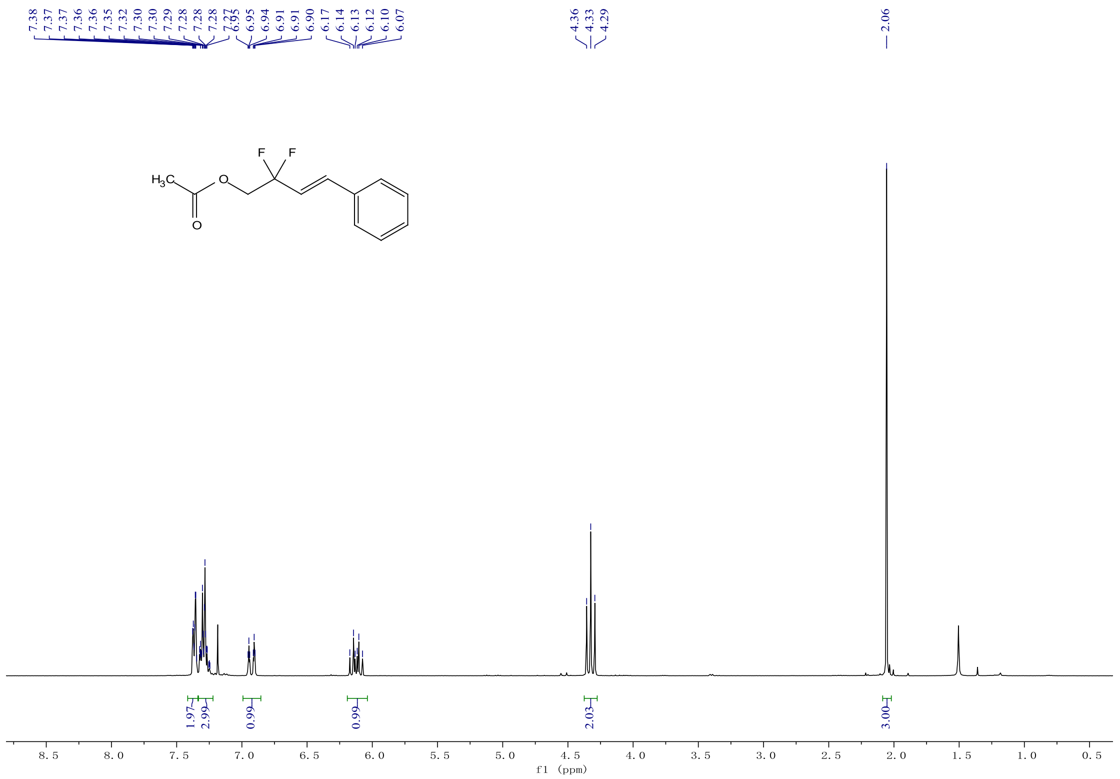

^{1}H-NMR of compound **3i** (400MHz, $CDCl_3$)

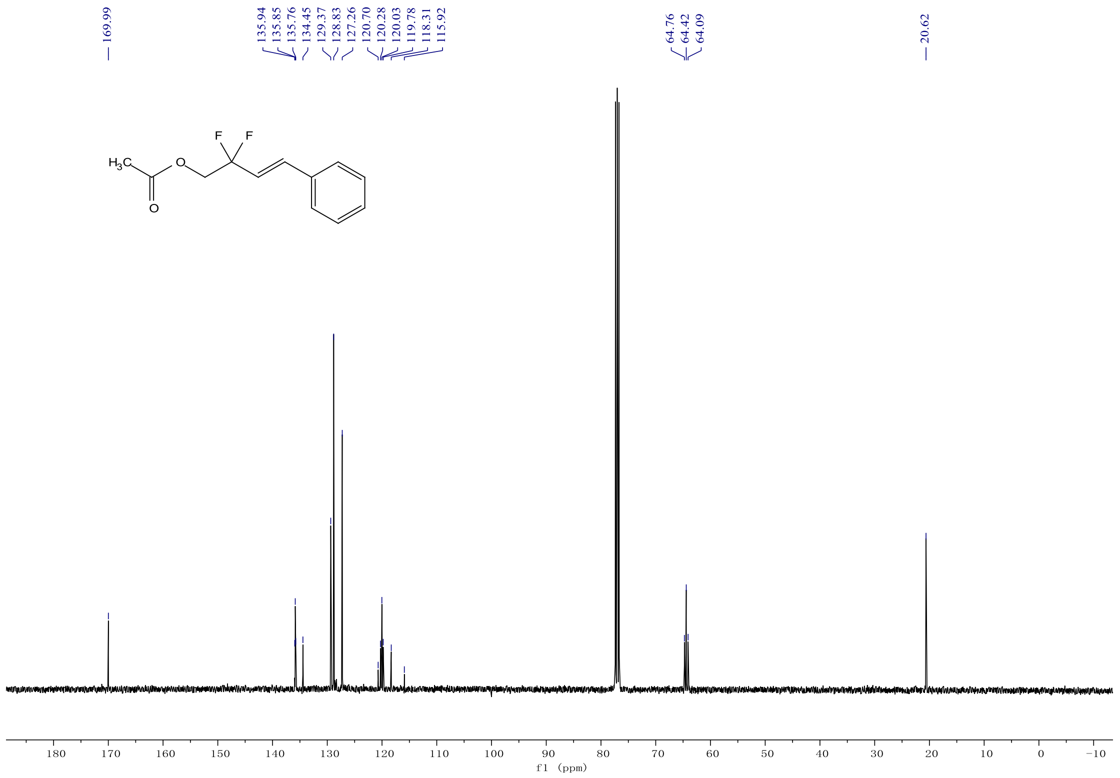

^{13}C{^{1}H}-NMR of compound **3i** (101MHz, $CDCl_3$)

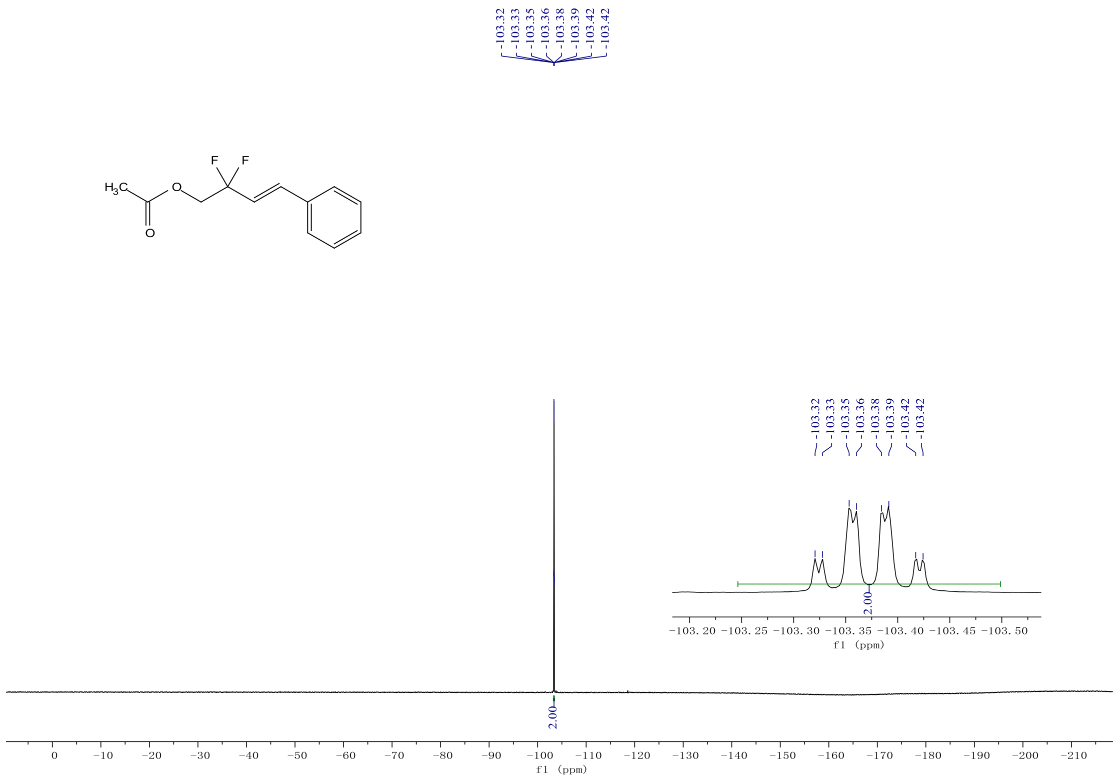

^{19}F-NMR of compound **3i** (377MHz, $CDCl_3$)

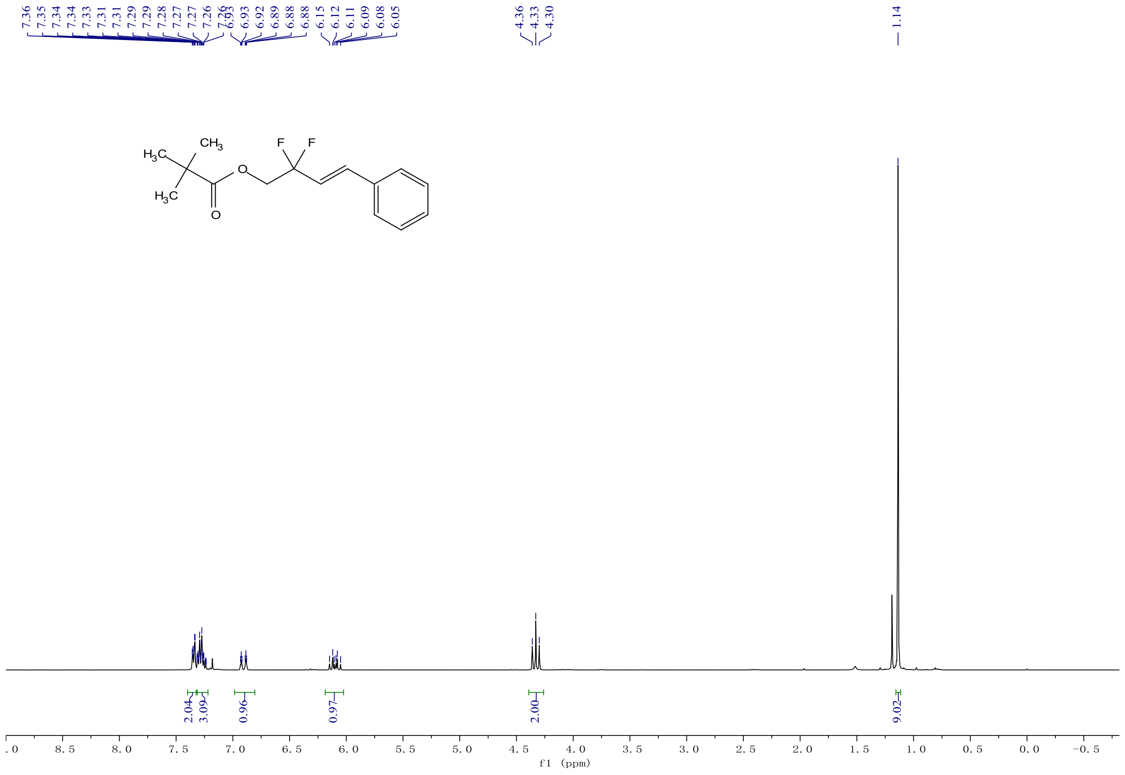

^{1}H-NMR of compound **3j** (400MHz, $CDCl_3$)

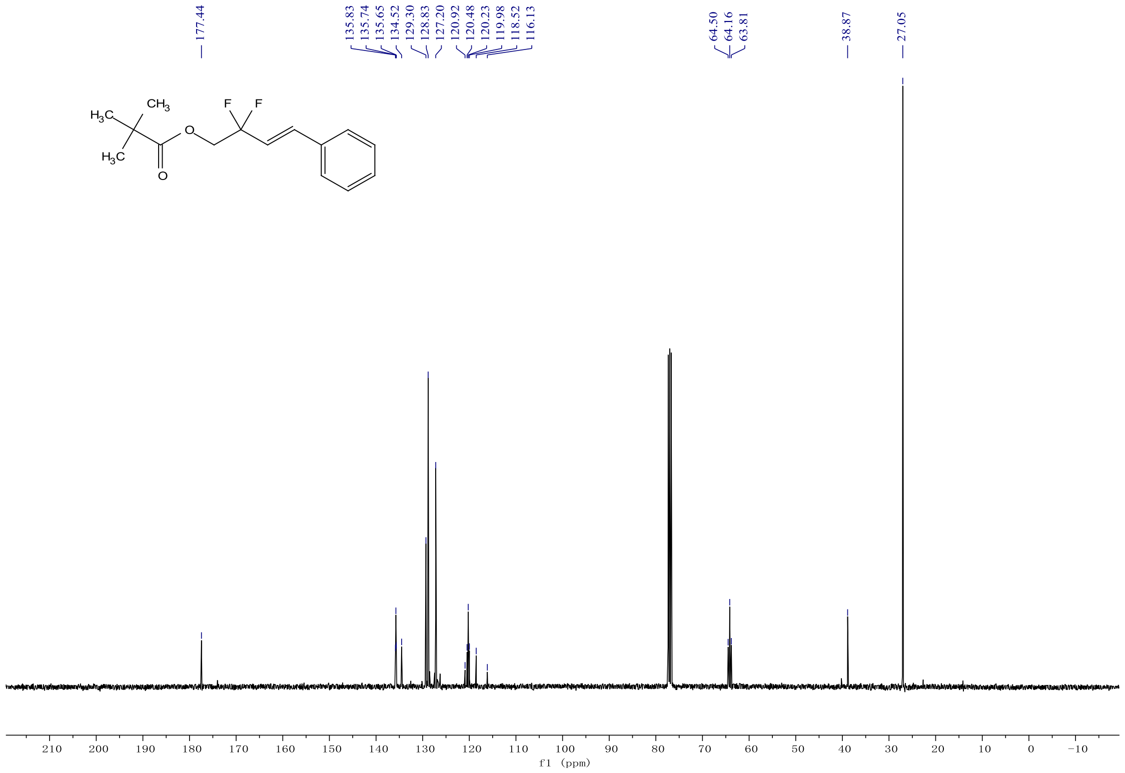

$^{13}C\{^{1}H\}$-NMR of compound **3j** (101MHz, $CDCl_3$)

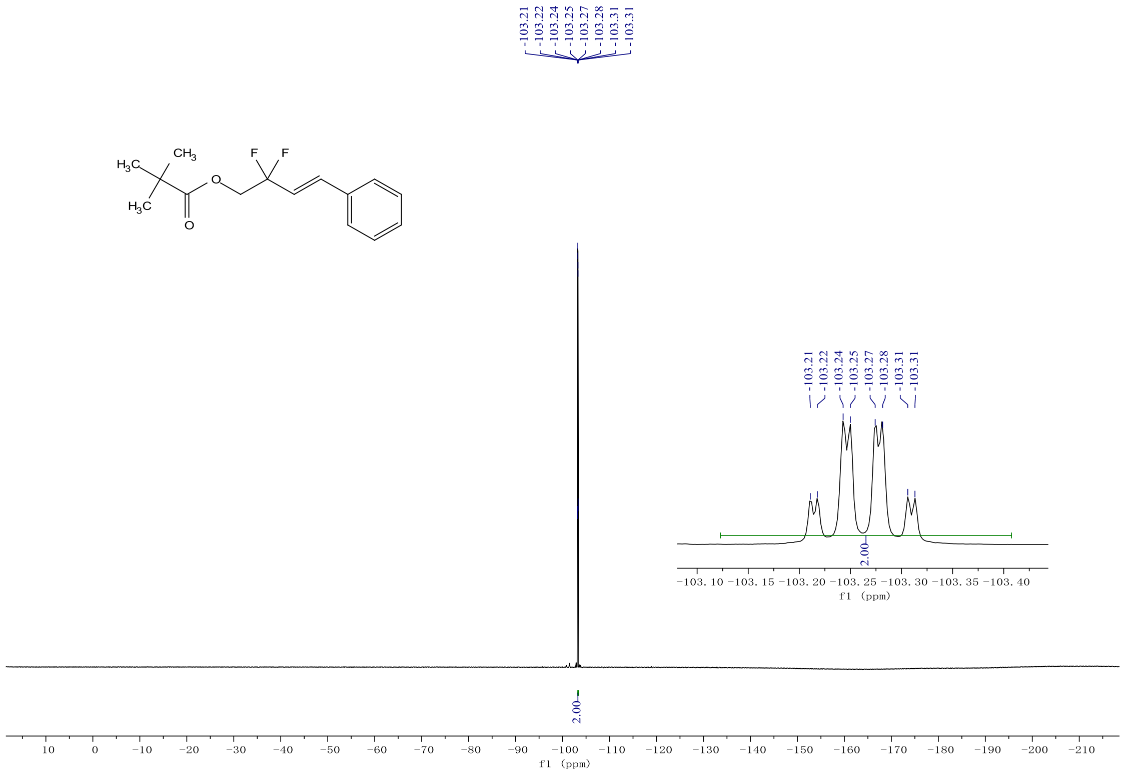

^{19}F-NMR of compound **3j** (377MHz, $CDCl_3$)

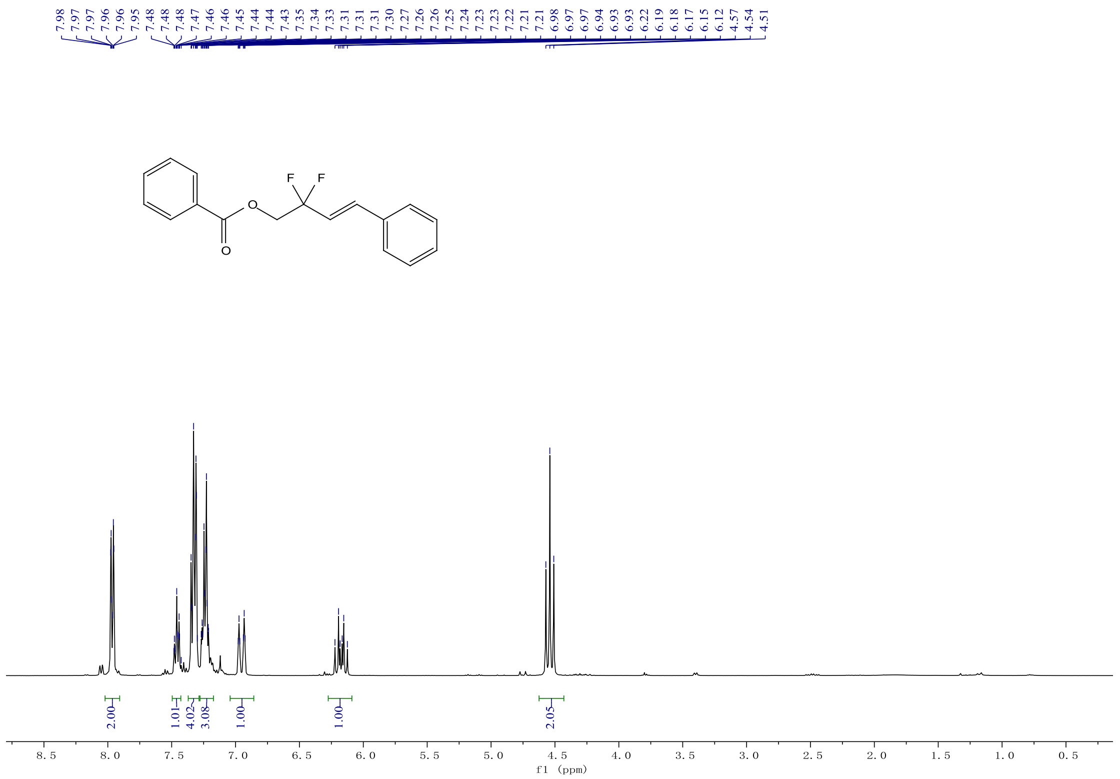

^{1}H-NMR of compound **3k** (400MHz, $CDCl_3$)

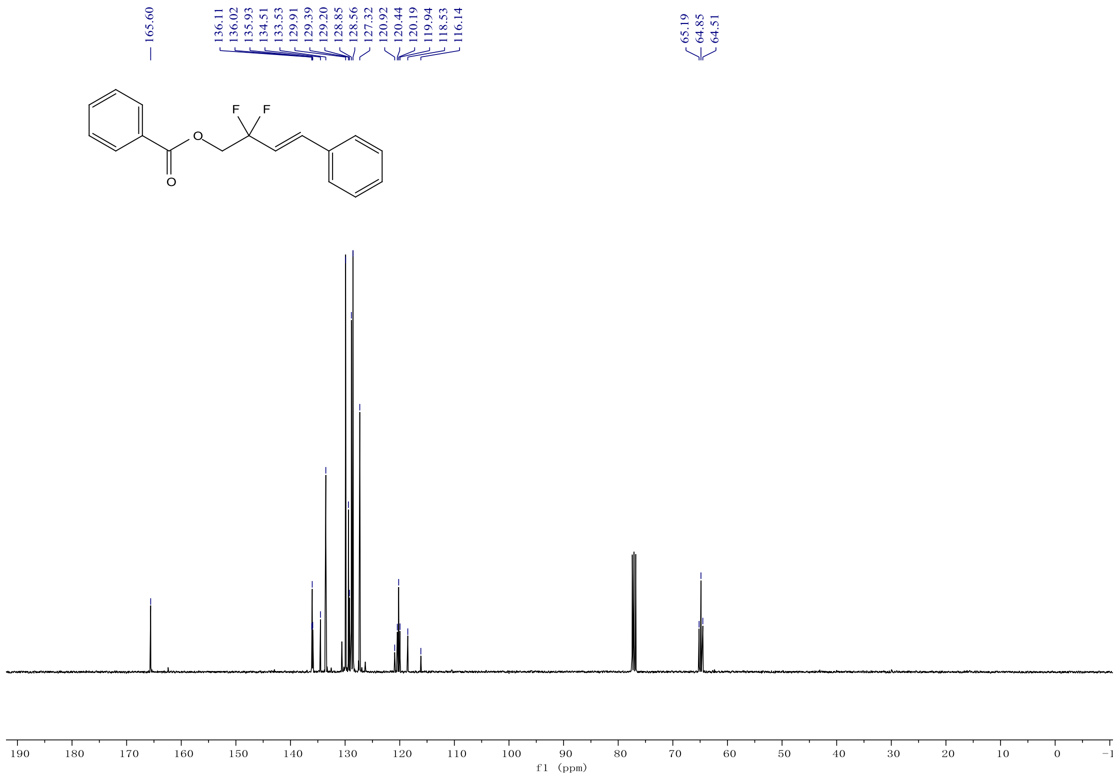

^{13}C{^{1}H}-NMR of compound **3k** (101MHz, $CDCl_3$)

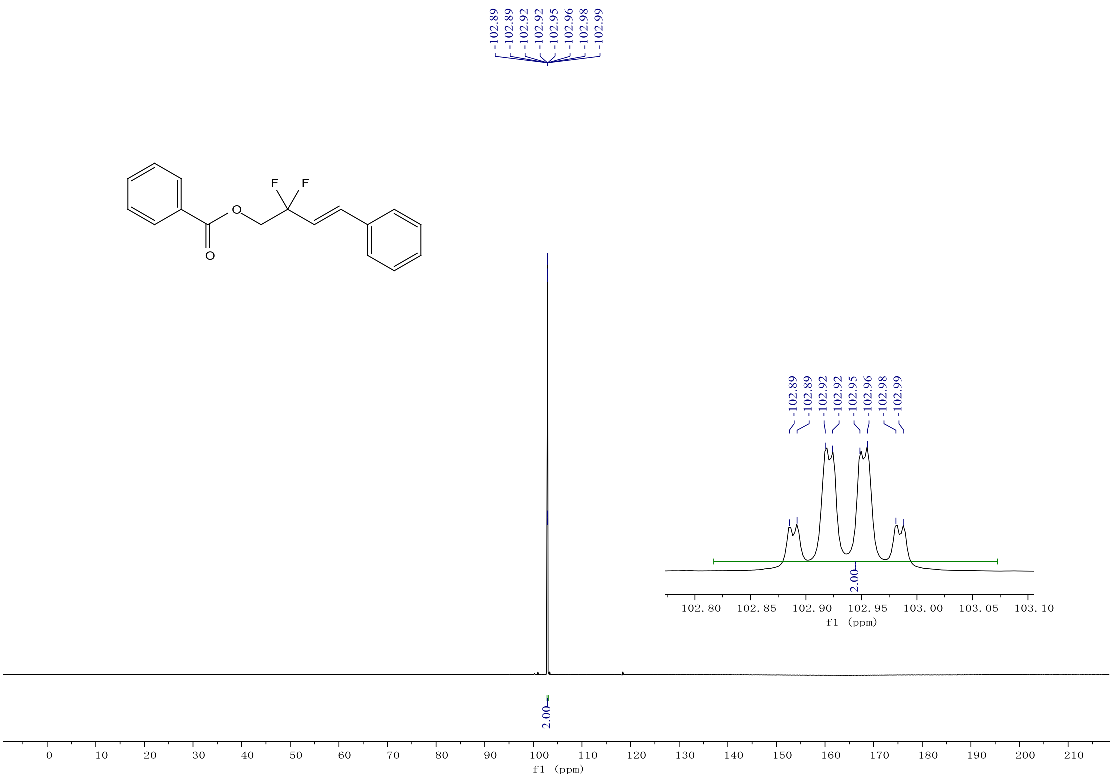

^{19}F-NMR of compound **3k** (377MHz, $CDCl_3$)

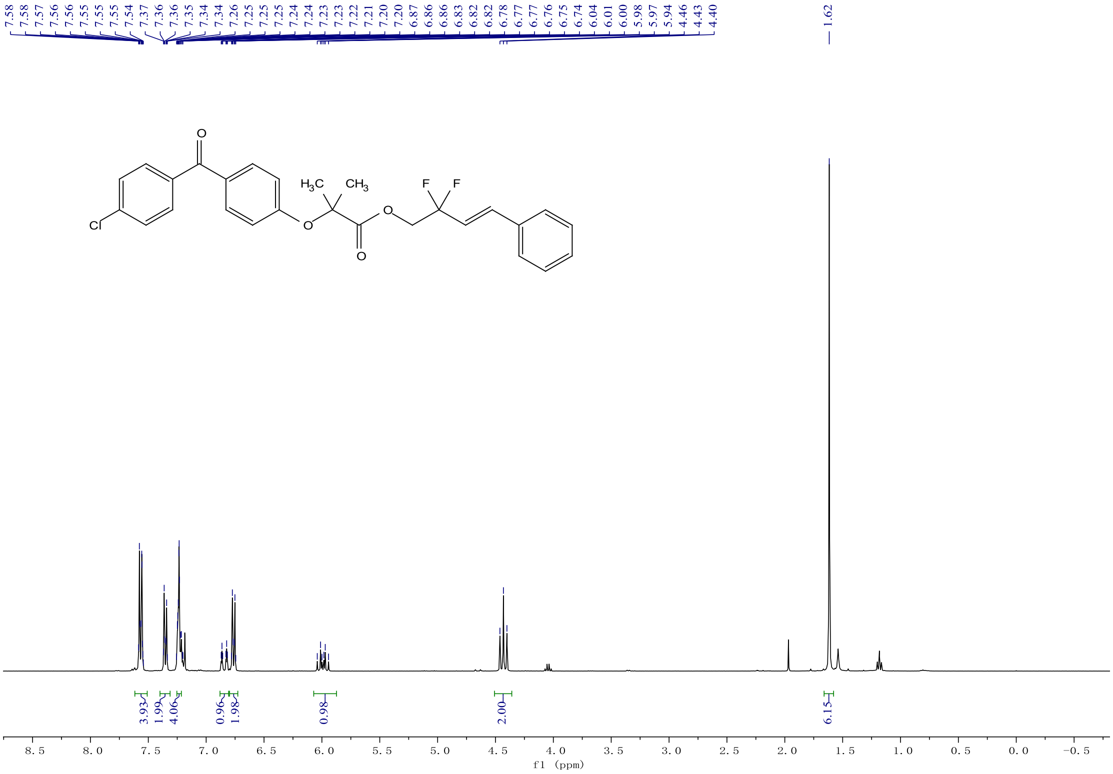

^{1}H-NMR of compound **5f** (400MHz, $CDCl_3$)

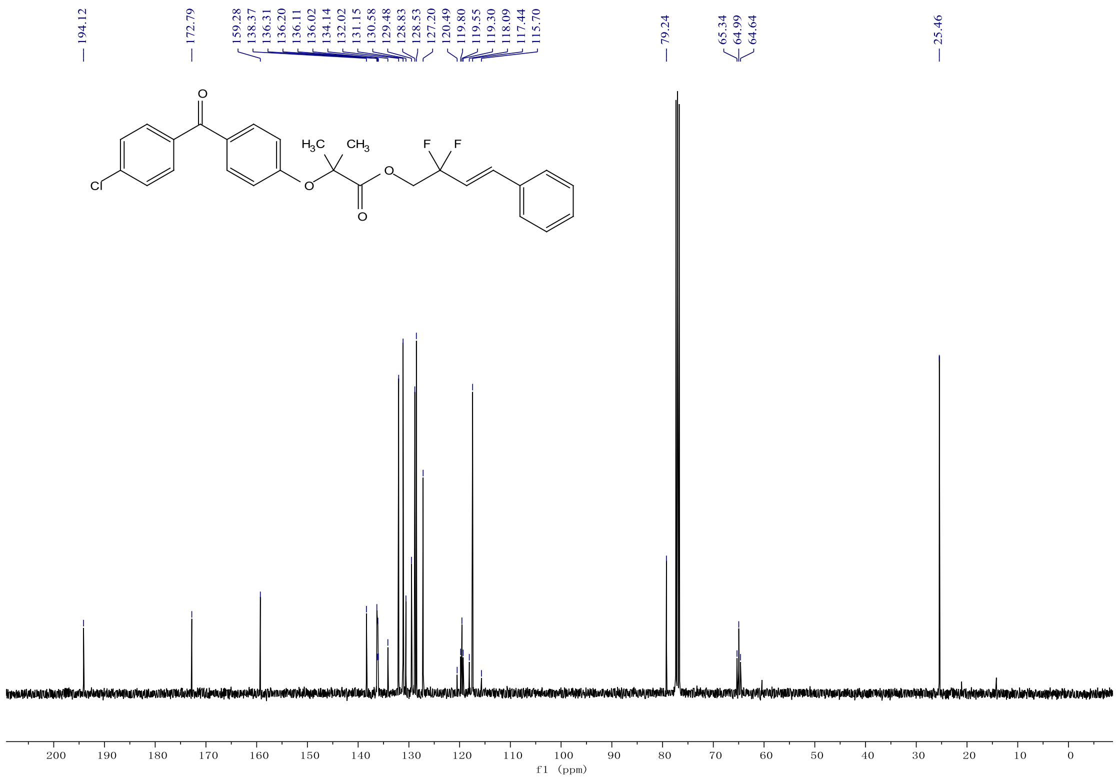

$^{13}C\{^{1}H\}$-NMR of compound **5f** (101MHz, $CDCl_3$)

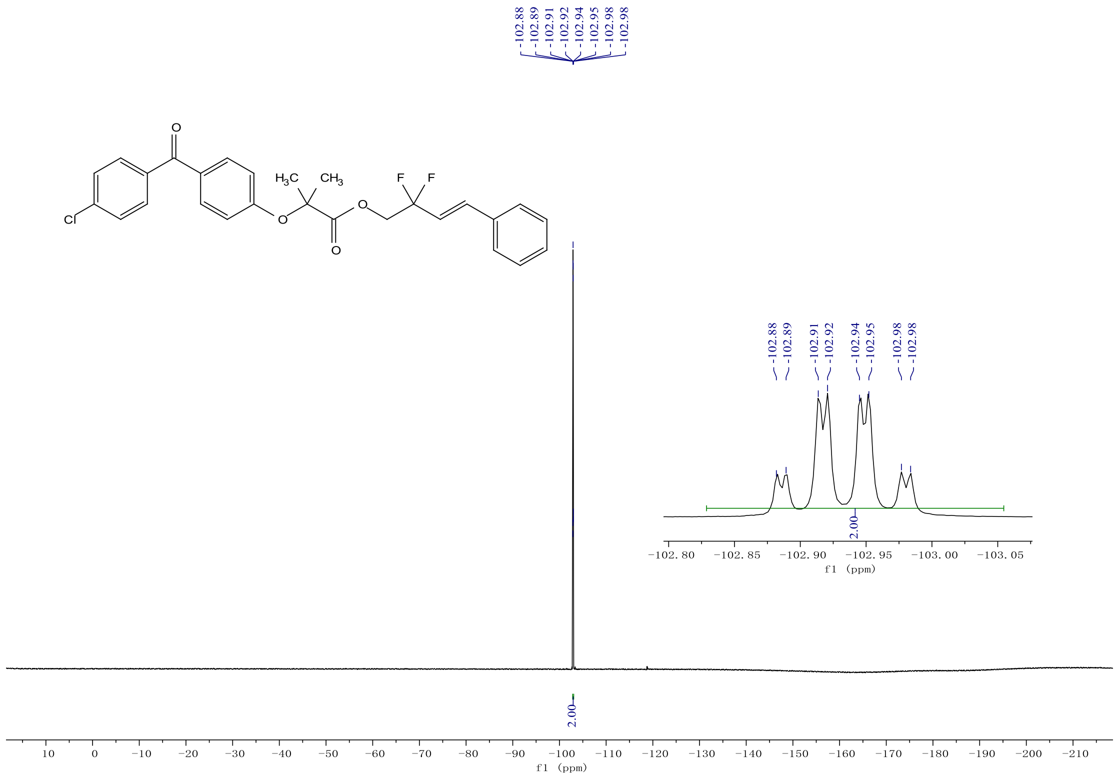

^{19}F-NMR of compound **5f** (377MHz, $CDCl_3$)

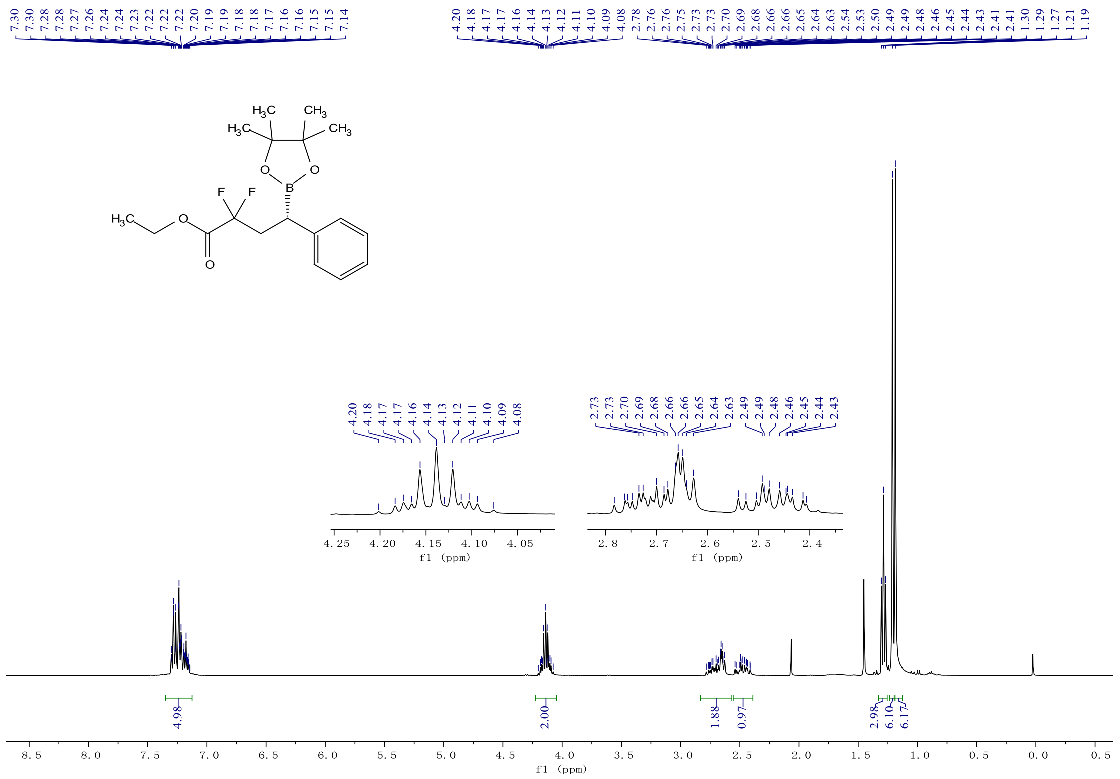

^{1}H-NMR of compound **2a** (400MHz, $CDCl_3$)

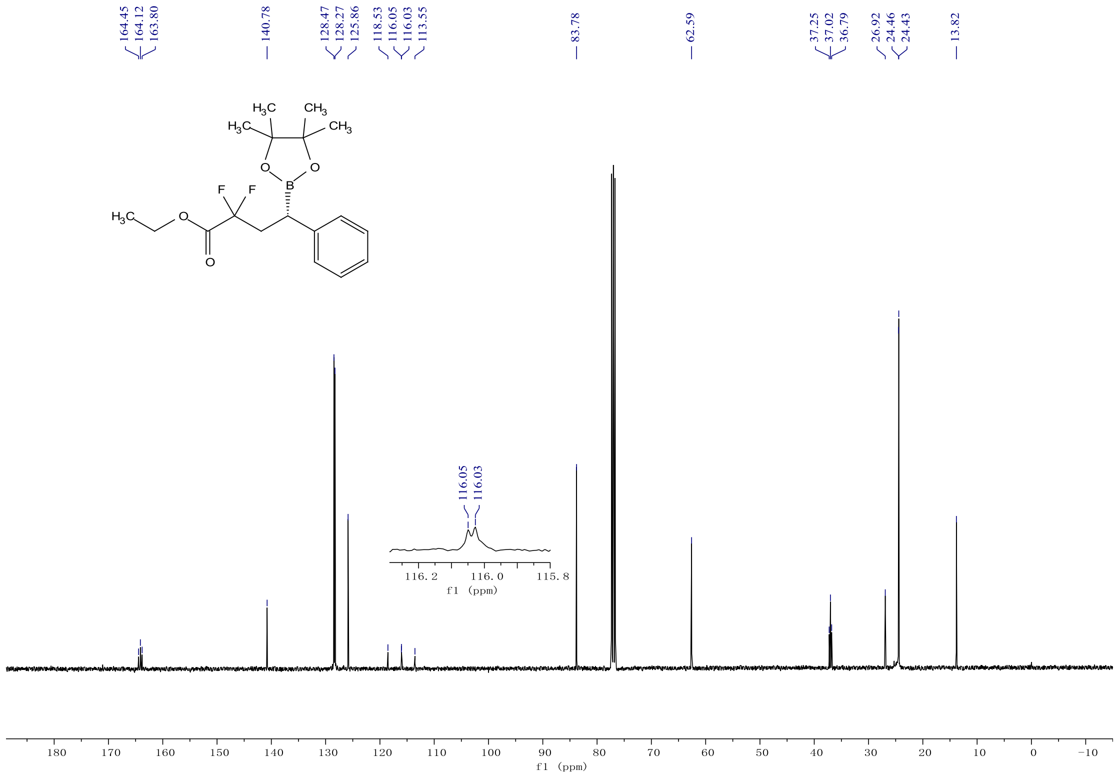

$^{13}C\{^{1}H\}$-NMR of compound **2a** (101MHz, $CDCl_3$)

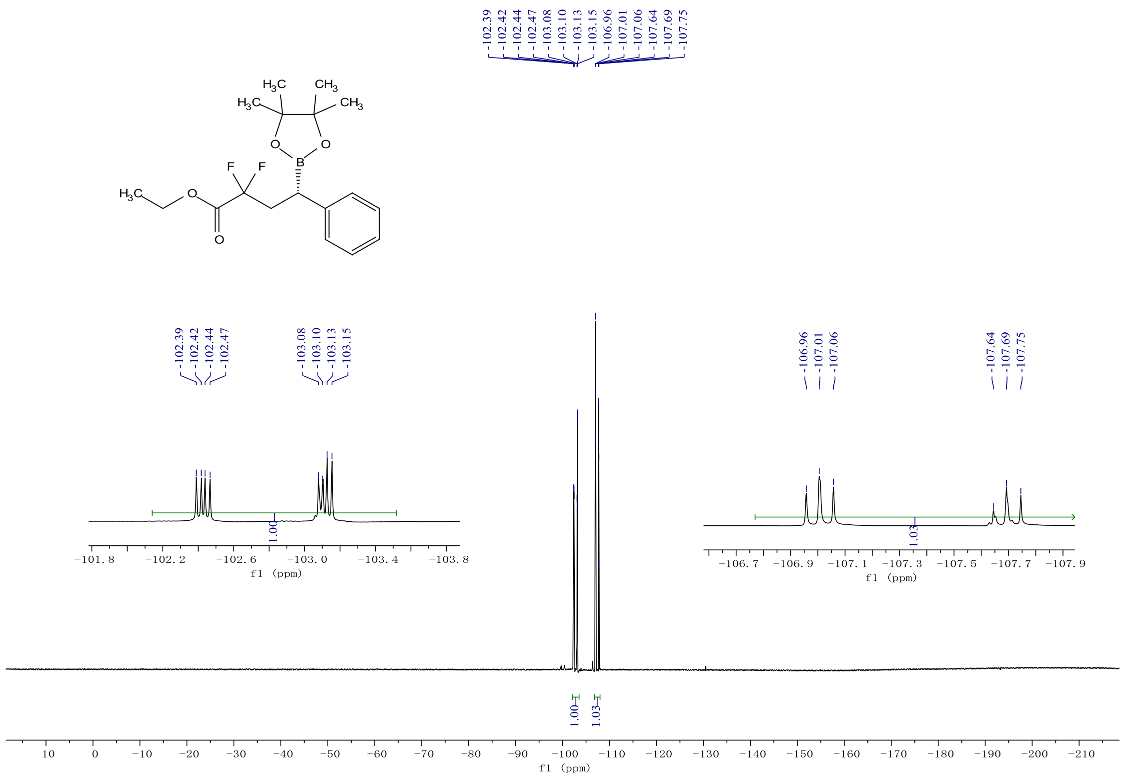

^{19}F-NMR of compound **2a** (377MHz, $CDCl_3$)

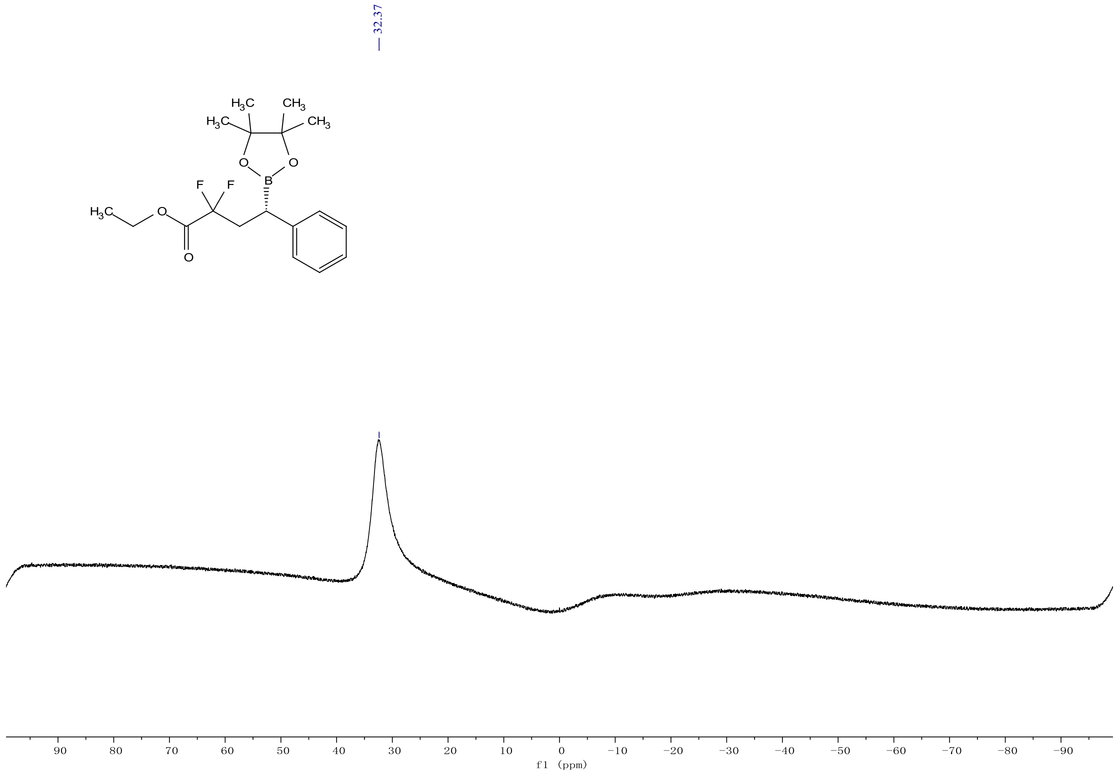

^{11}B-NMR of compound **2a** (128MHz, $CDCl_3$)

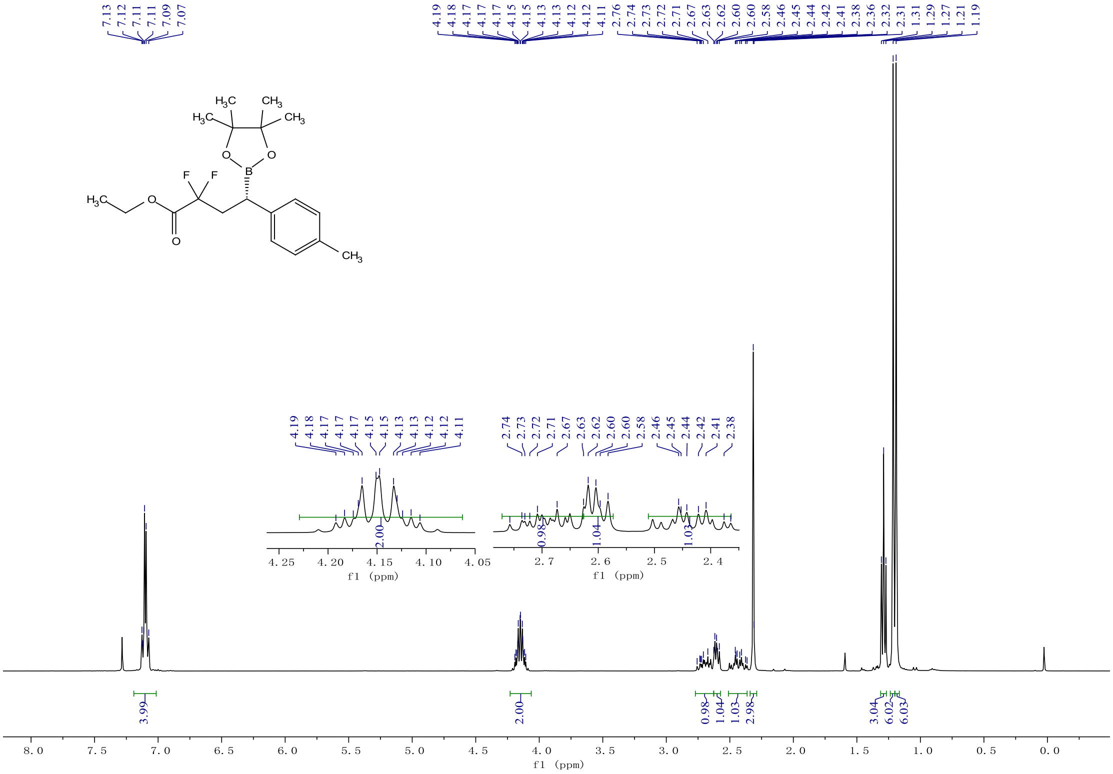

^{1}H-NMR of compound **2b** (400MHz, $CDCl_3$)

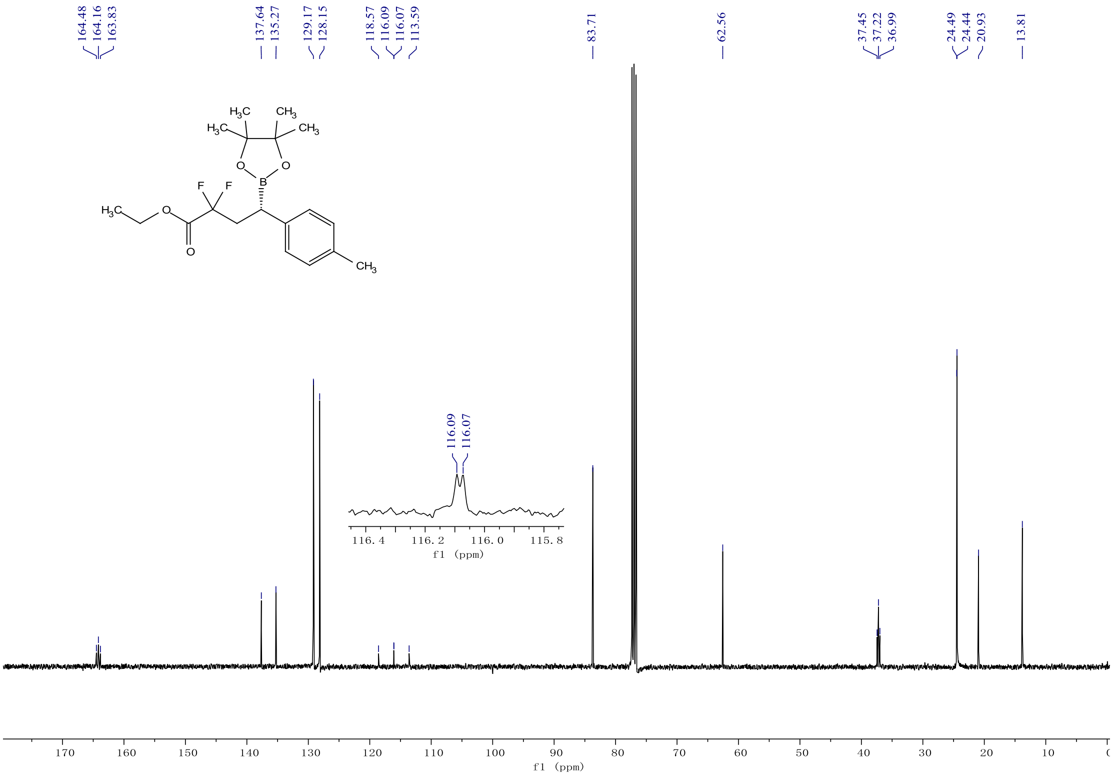

$^{13}C\{^{1}H\}$-NMR of compound **2b** (101MHz, $CDCl_3$)

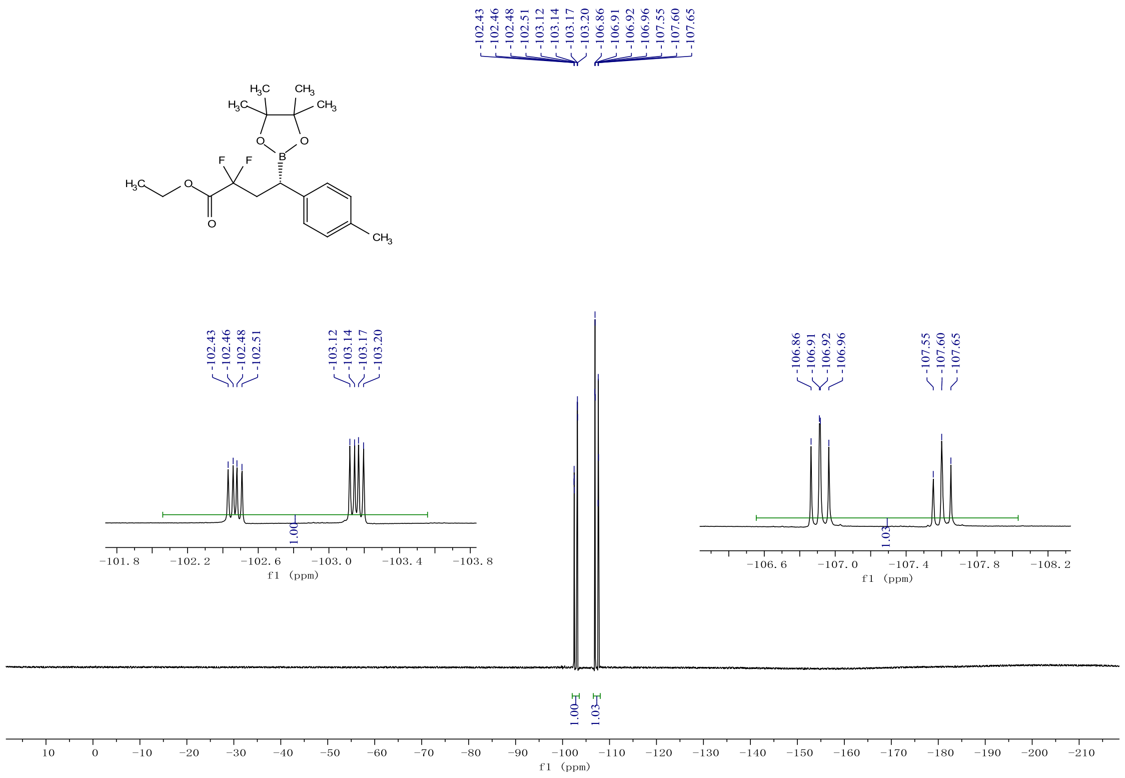

^{19}F-NMR of compound **2b** (377MHz, $CDCl_3$)

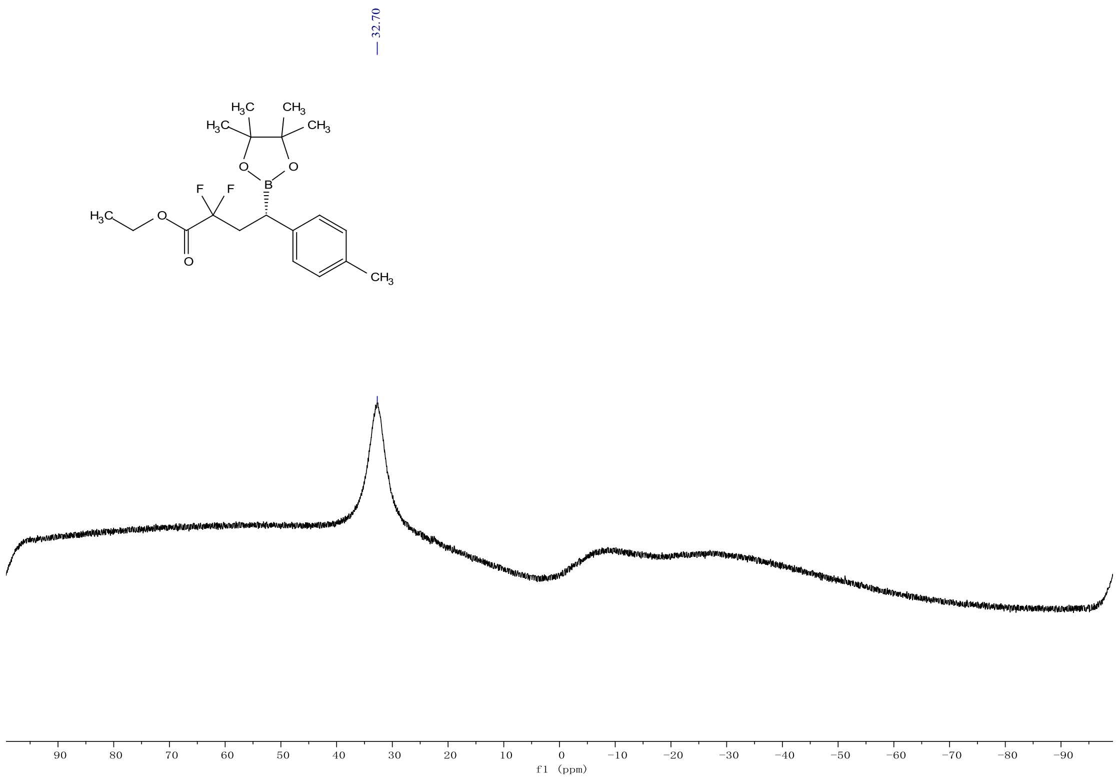

^{11}B-NMR of compound **2b** (128MHz, $CDCl_3$)

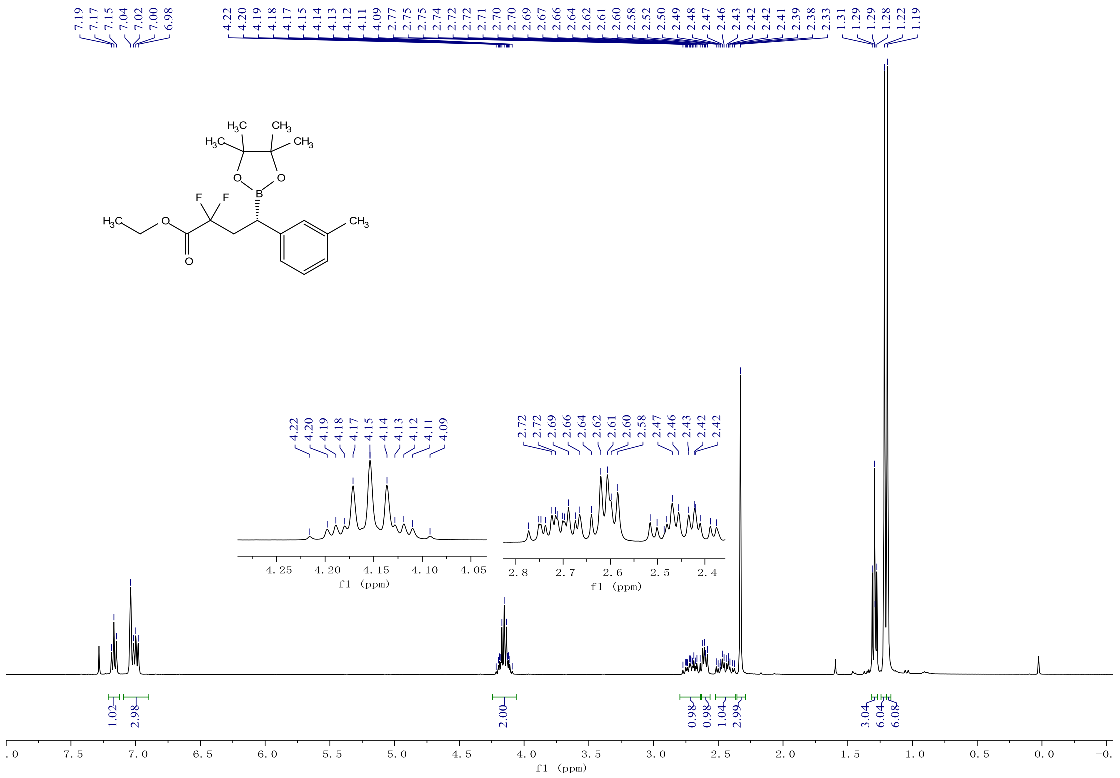

^{1}H-NMR of compound **2c** (400MHz, $CDCl_3$)

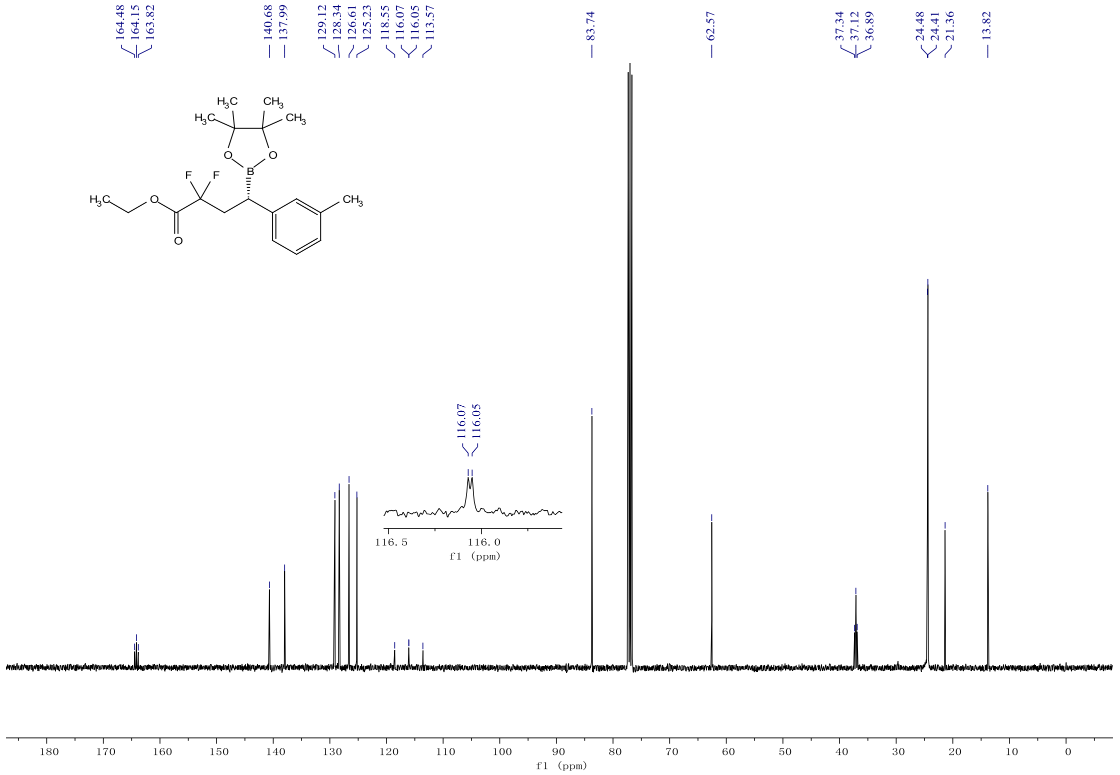

$^{13}C\{^{1}H\}$-NMR of compound **2c** (101MHz, $CDCl_3$)

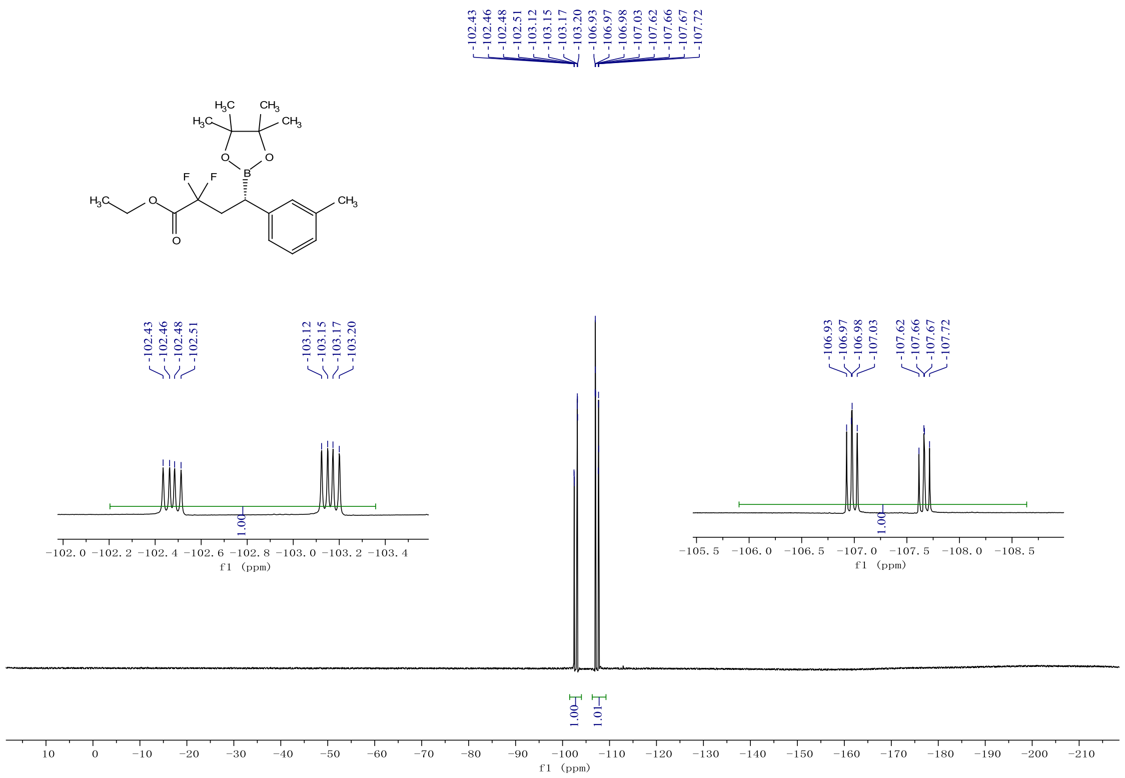

^{19}F-NMR of compound **2c** (377MHz, $CDCl_3$)

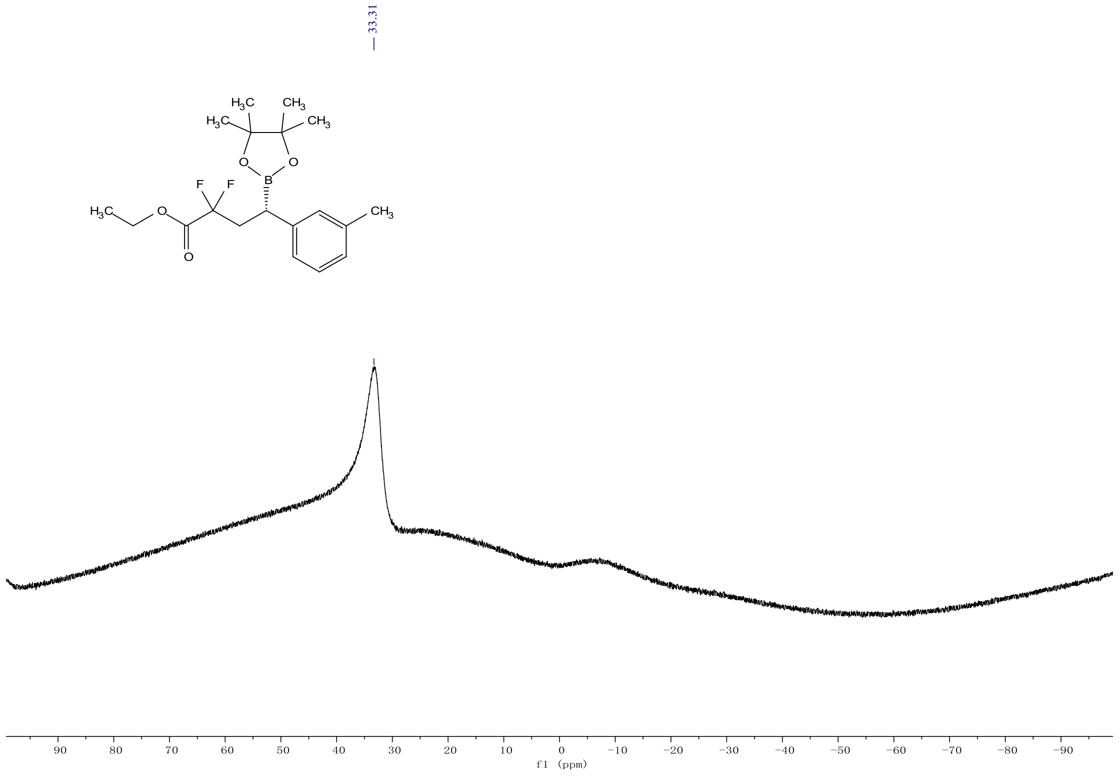

^{11}B-NMR of compound **2c** (128MHz, $CDCl_3$)

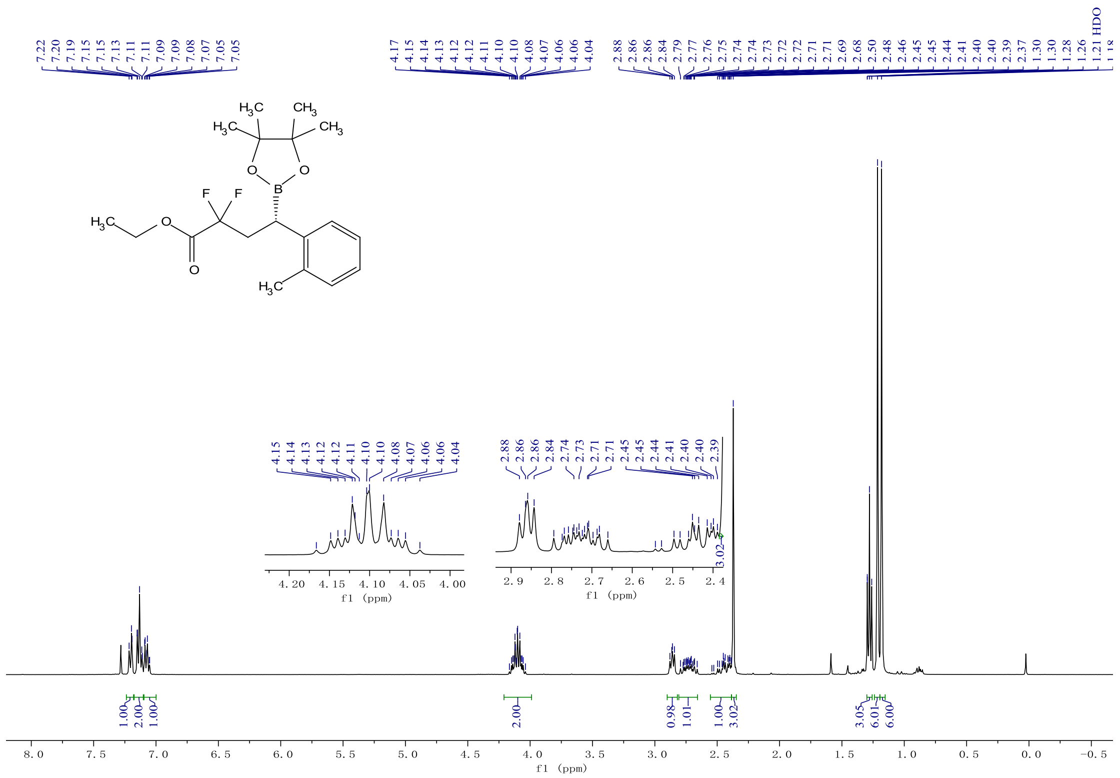

^{1}H-NMR of compound **2d** (400MHz, $CDCl_3$)

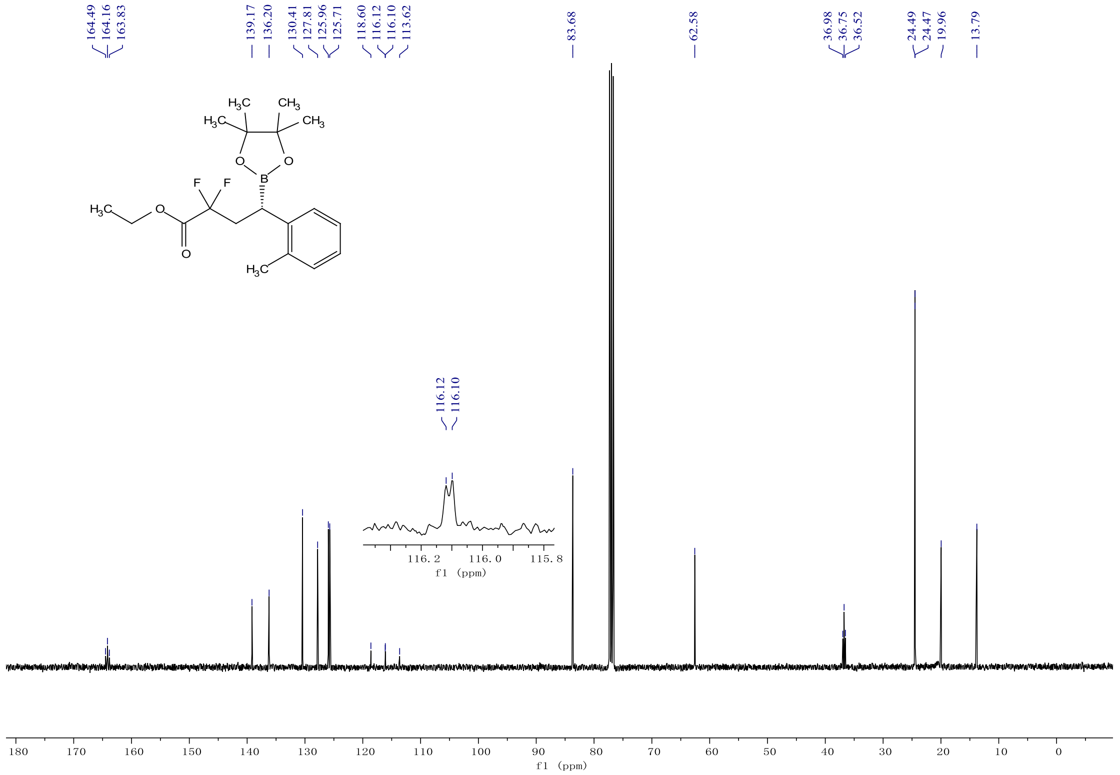

$^{13}C\{^{1}H\}$-NMR of compound **2d** (101MHz, $CDCl_3$)

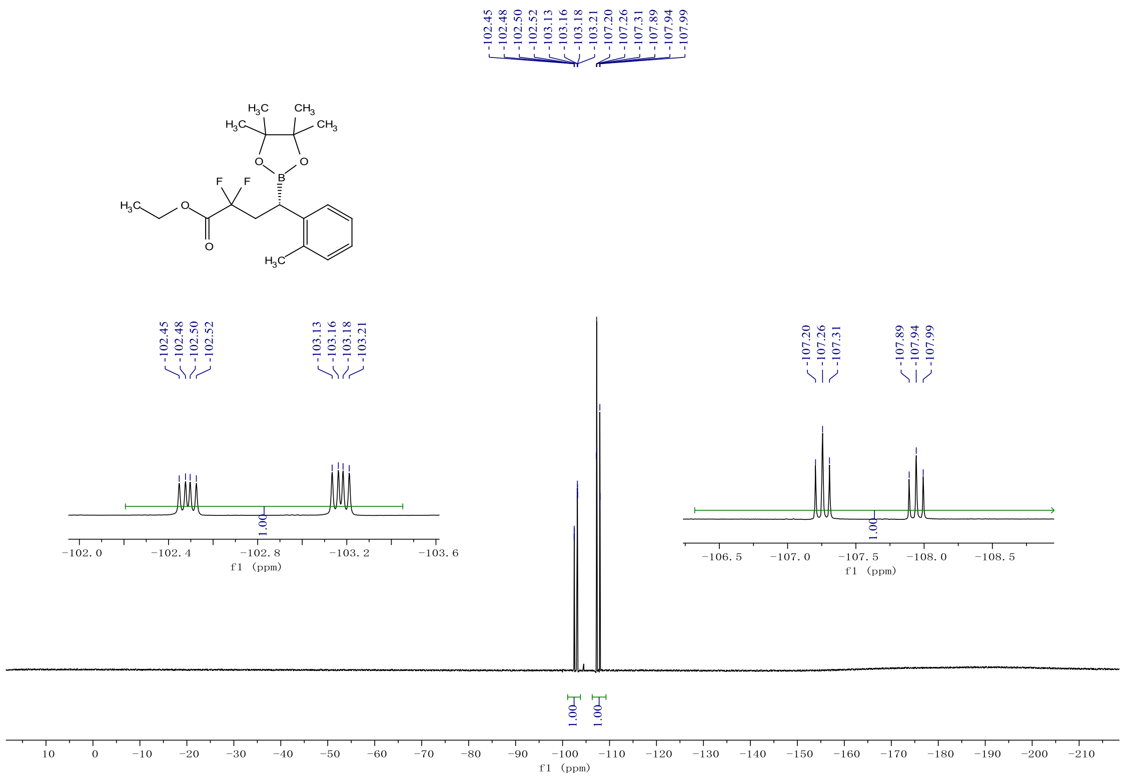

^{19}F-NMR of compound **2d** (377MHz, $CDCl_3$)

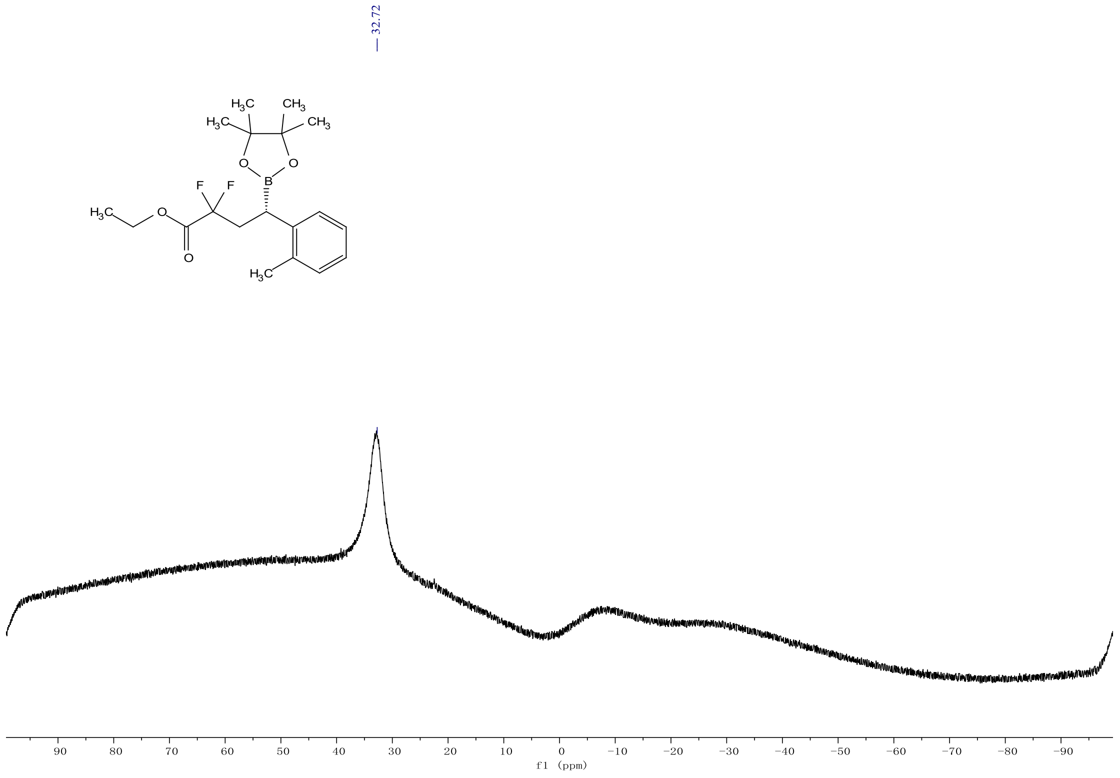

^{11}B-NMR of compound **2d** (128MHz, $CDCl_3$)

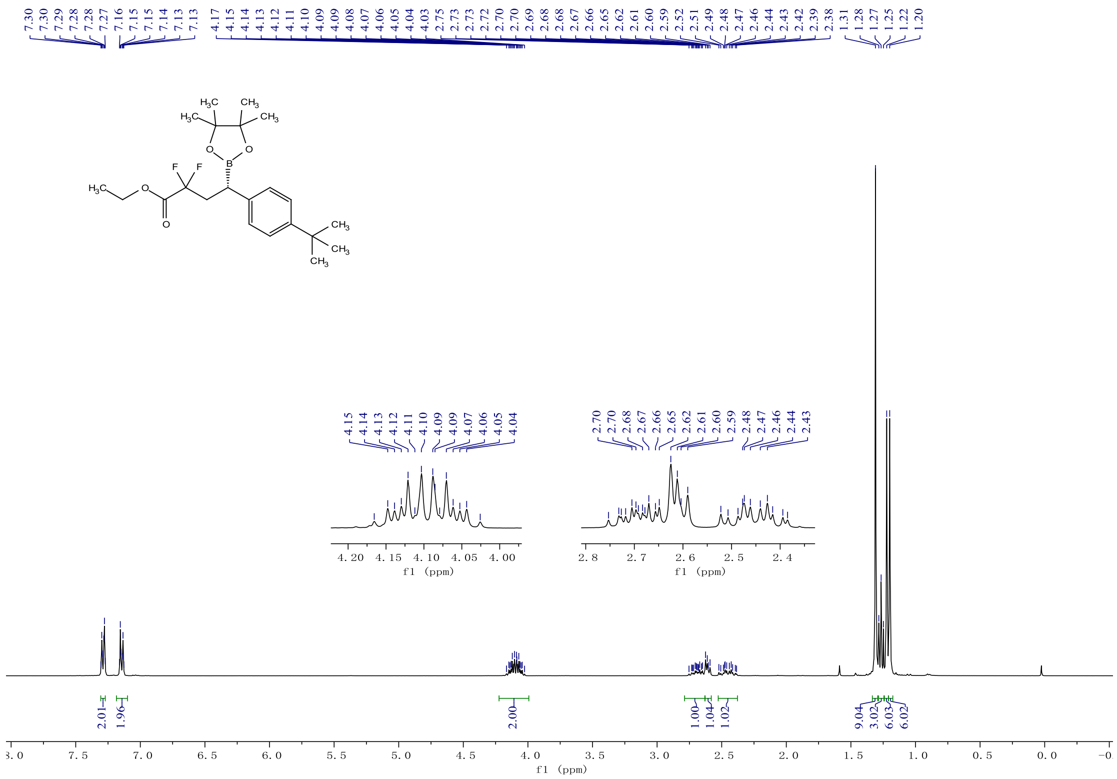

^{1}H-NMR of compound **2e** (400MHz, $CDCl_3$)

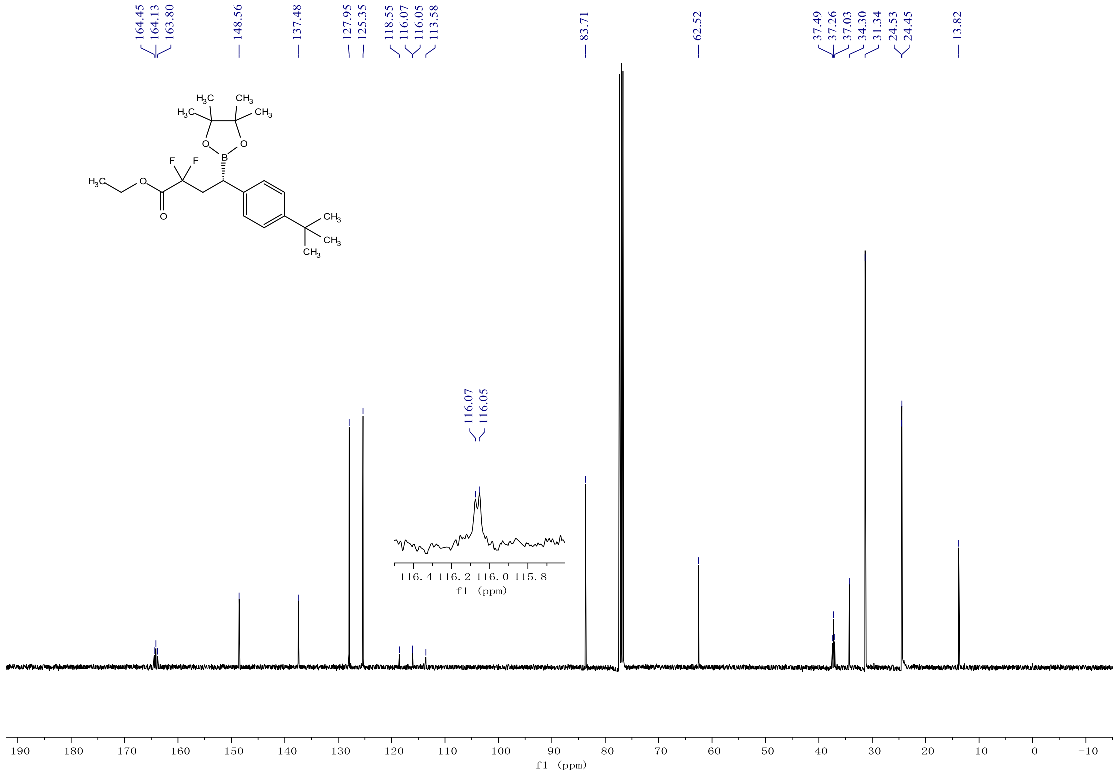

$^{13}C\{^{1}H\}$-NMR of compound **2e** (101MHz, $CDCl_3$)

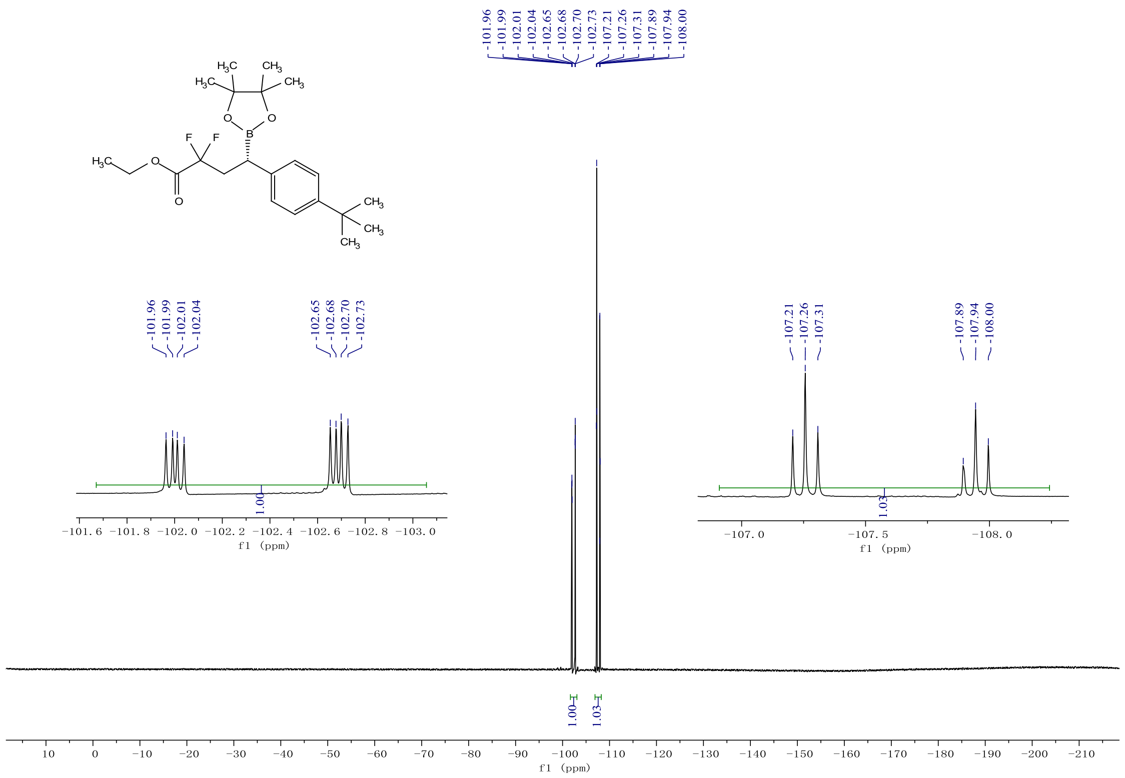

^{19}F-NMR of compound **2e** (377MHz, $CDCl_3$)

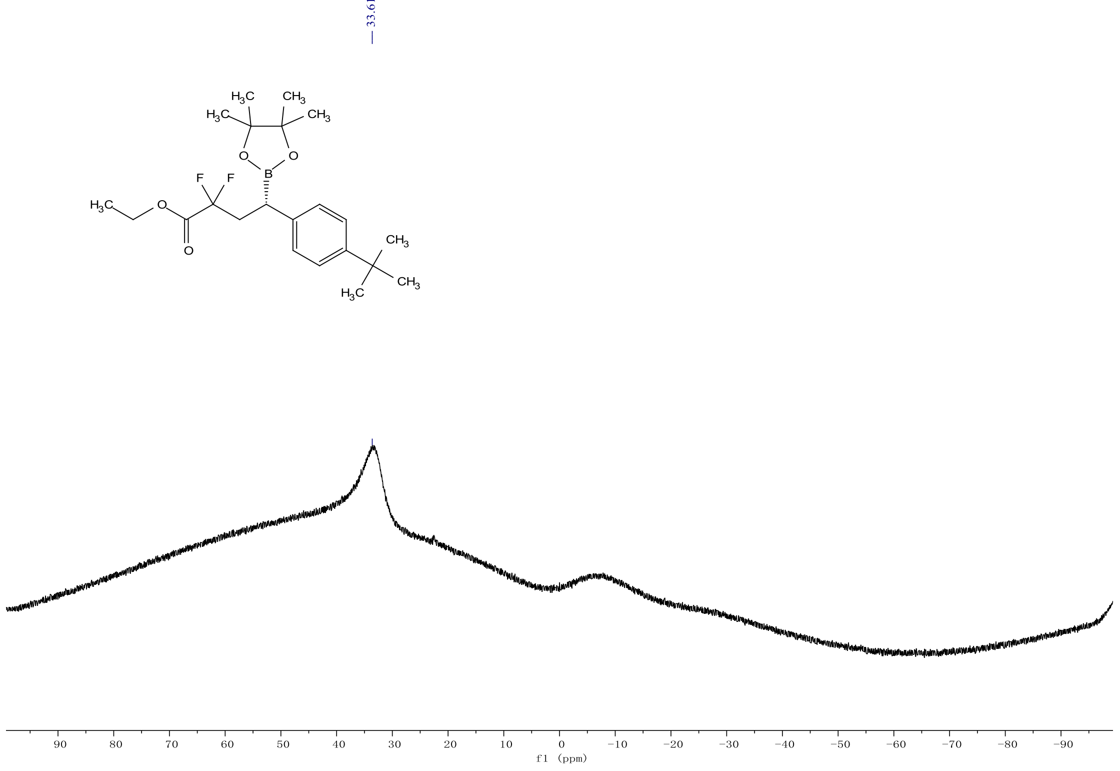

^{11}B-NMR of compound **2e** (128MHz, $CDCl_3$)

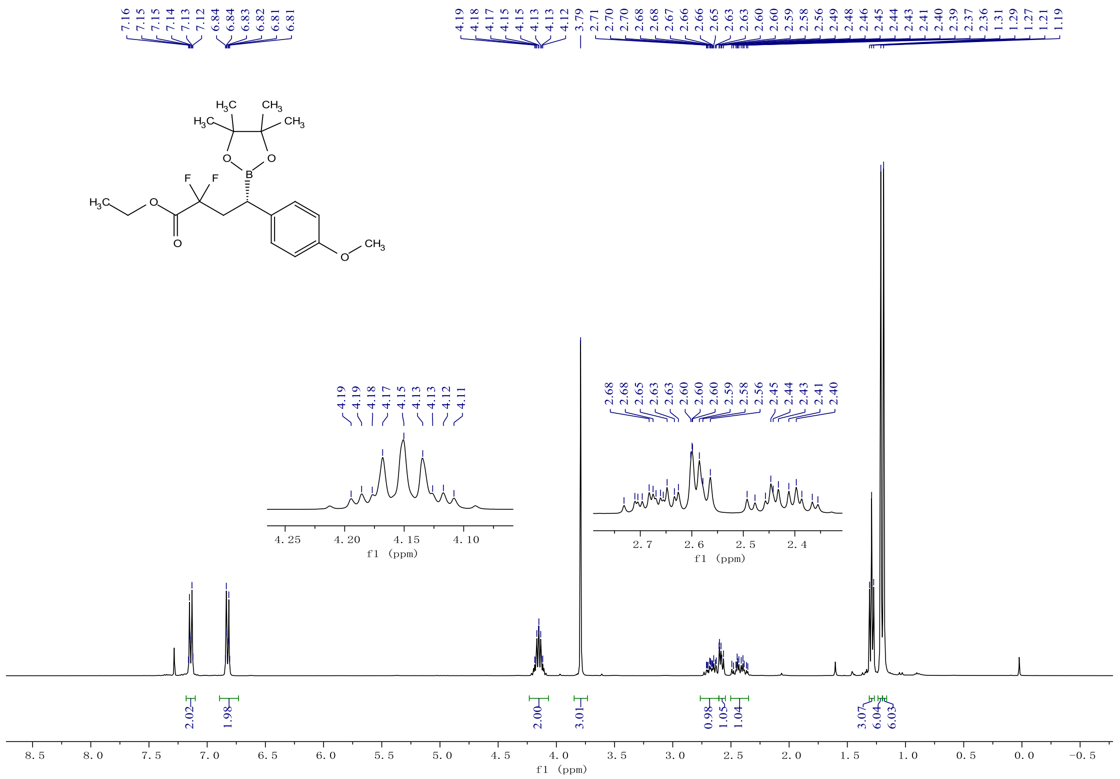

^{1}H-NMR of compound **2f** (400MHz, $CDCl_3$)

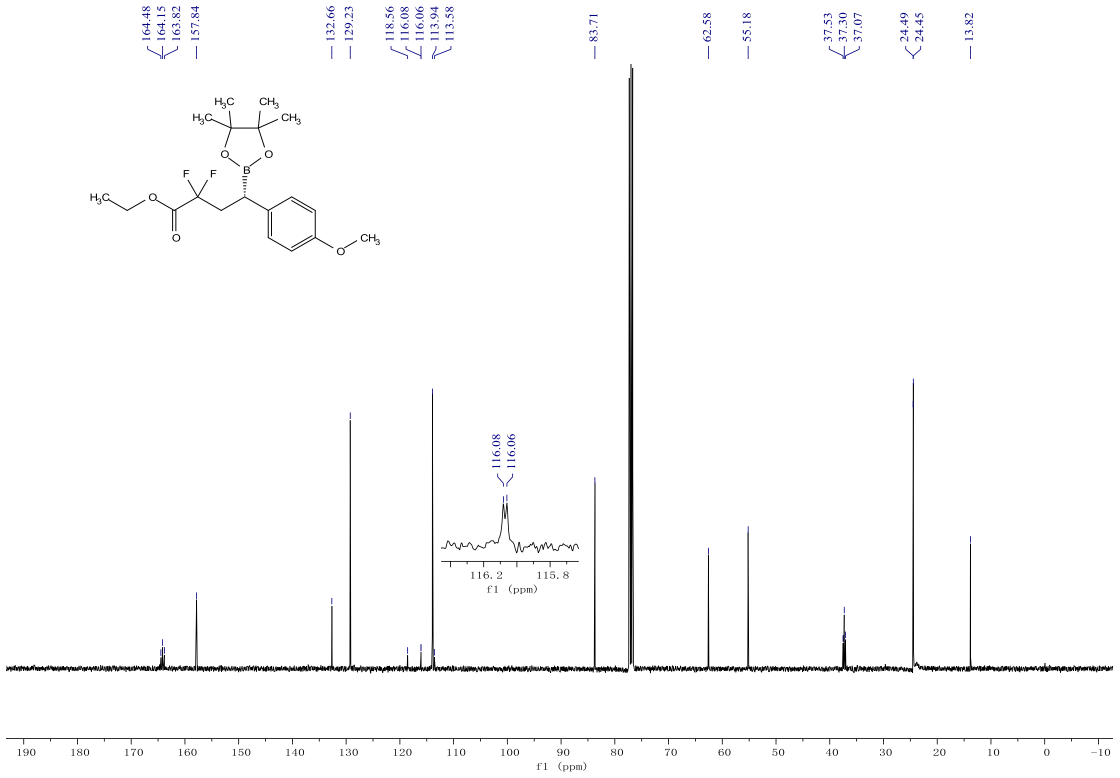

^{13}C{^{1}H}-NMR of compound **2f** (101MHz, $CDCl_3$)

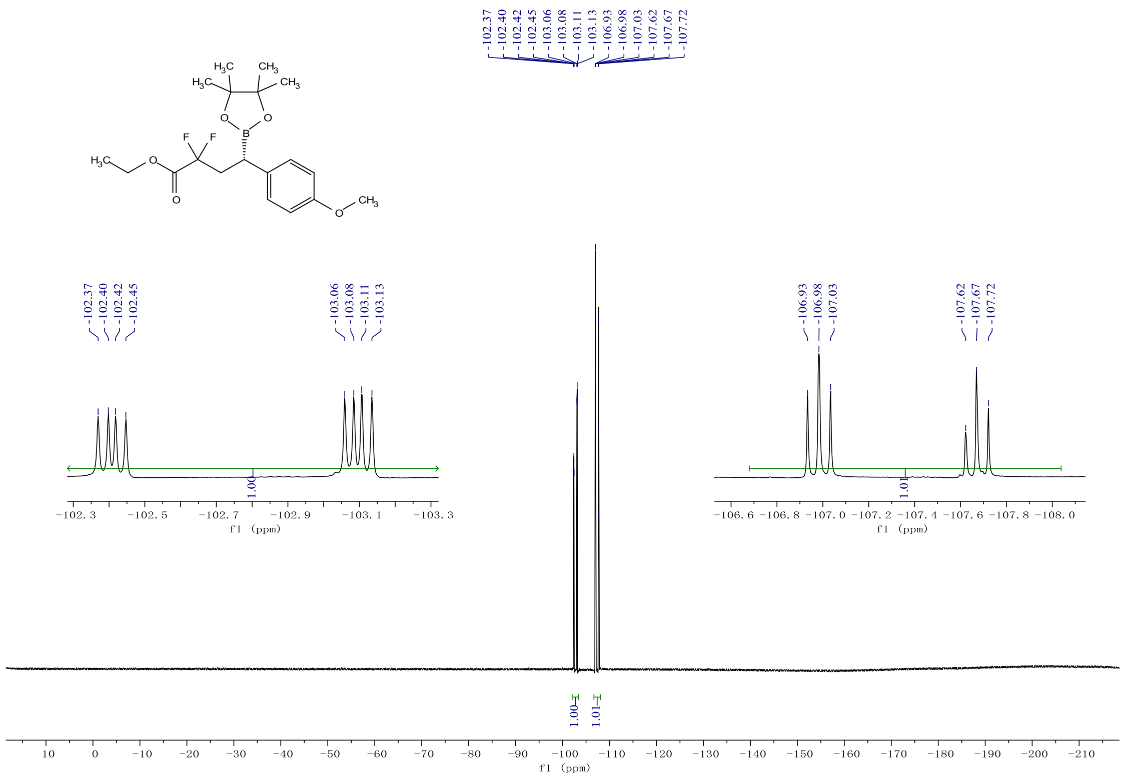

^{19}F-NMR of compound **2f** (377MHz, $CDCl_3$)

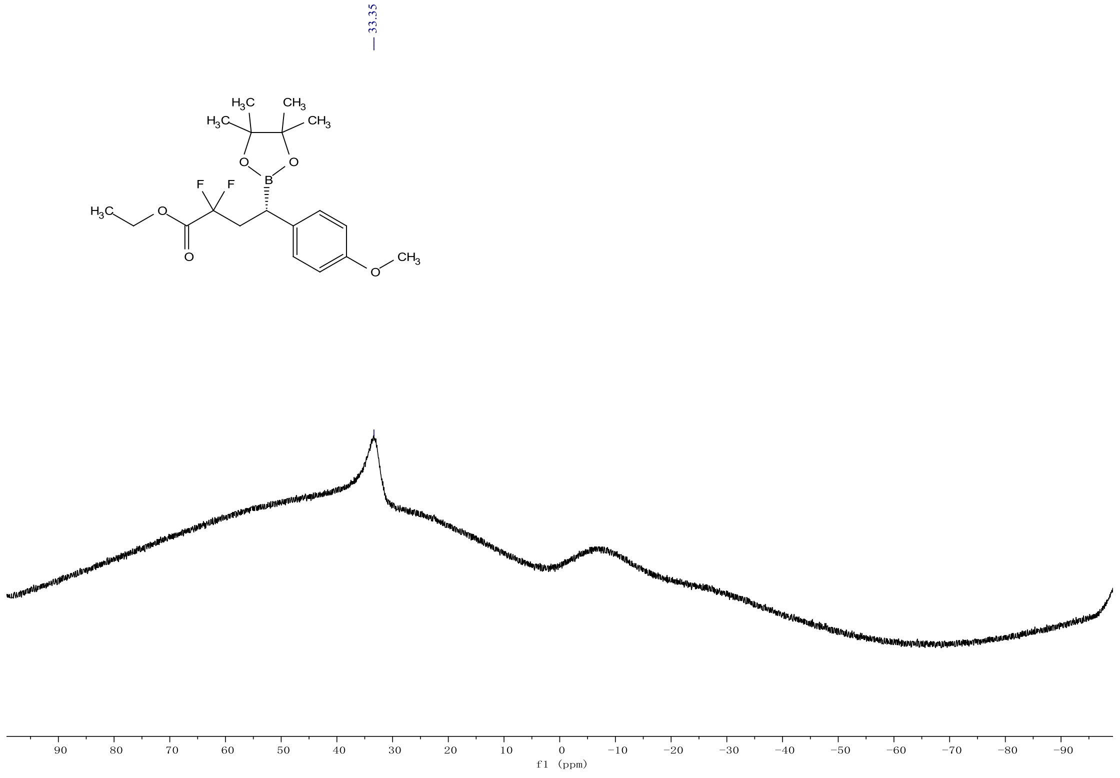

^{11}B-NMR of compound **2f** (128MHz, $CDCl_3$)

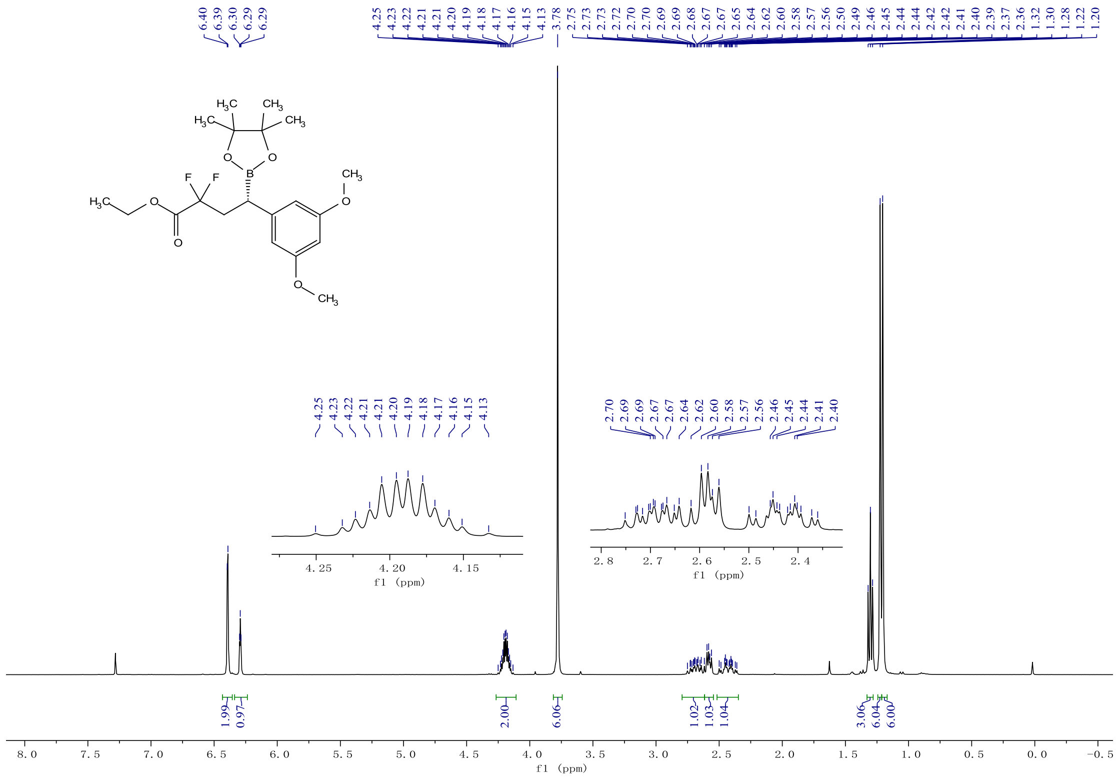

^{1}H-NMR of compound **2g** (400MHz, $CDCl_3$)

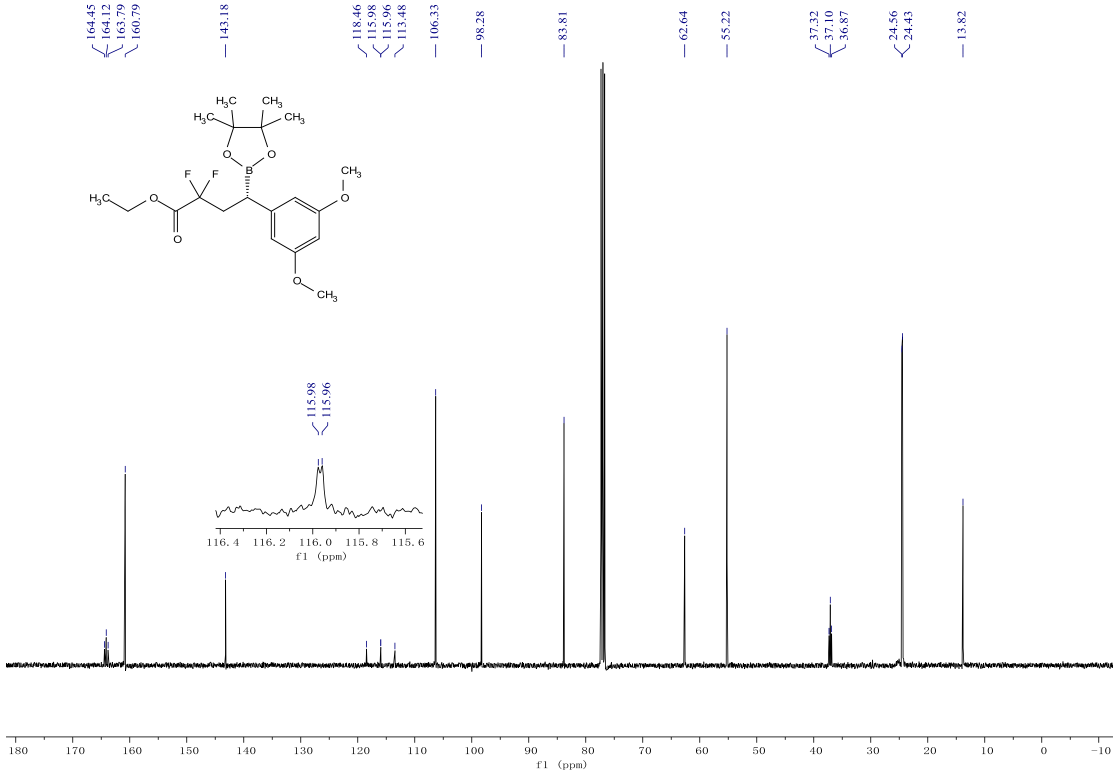

$^{13}C\{^{1}H\}$-NMR of compound **2g** (101MHz, $CDCl_3$)

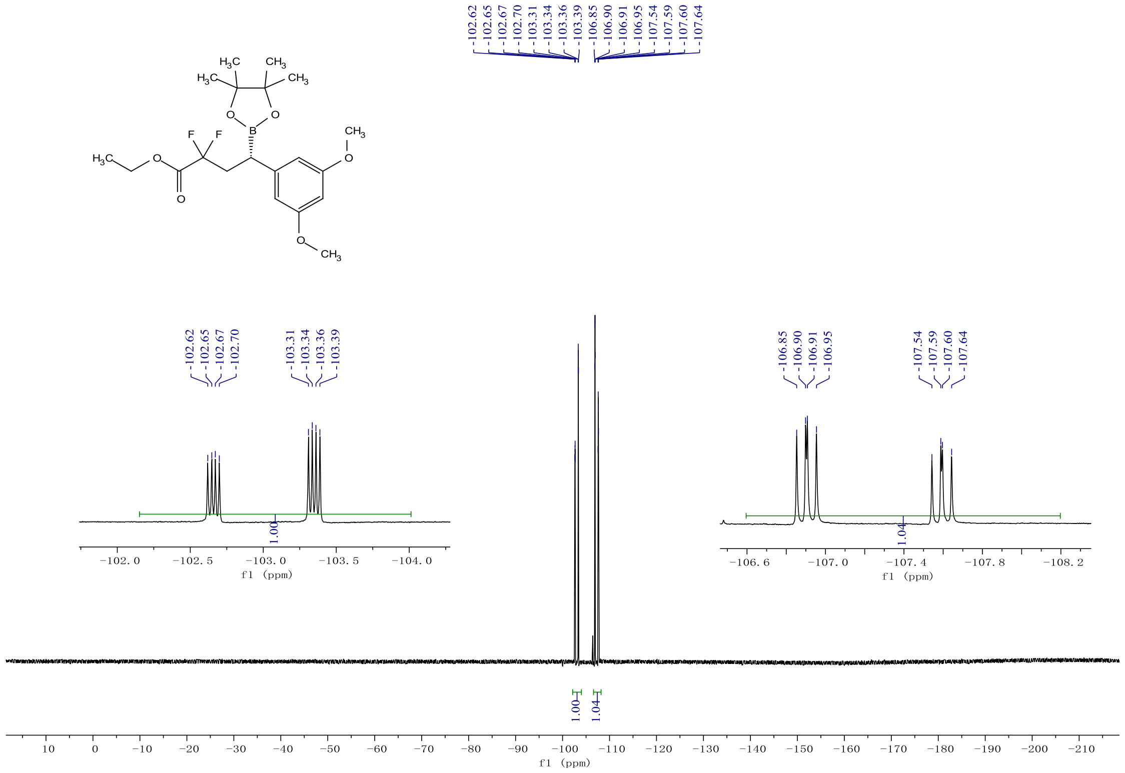

^{19}F-NMR of compound **2g** (377MHz, $CDCl_3$)

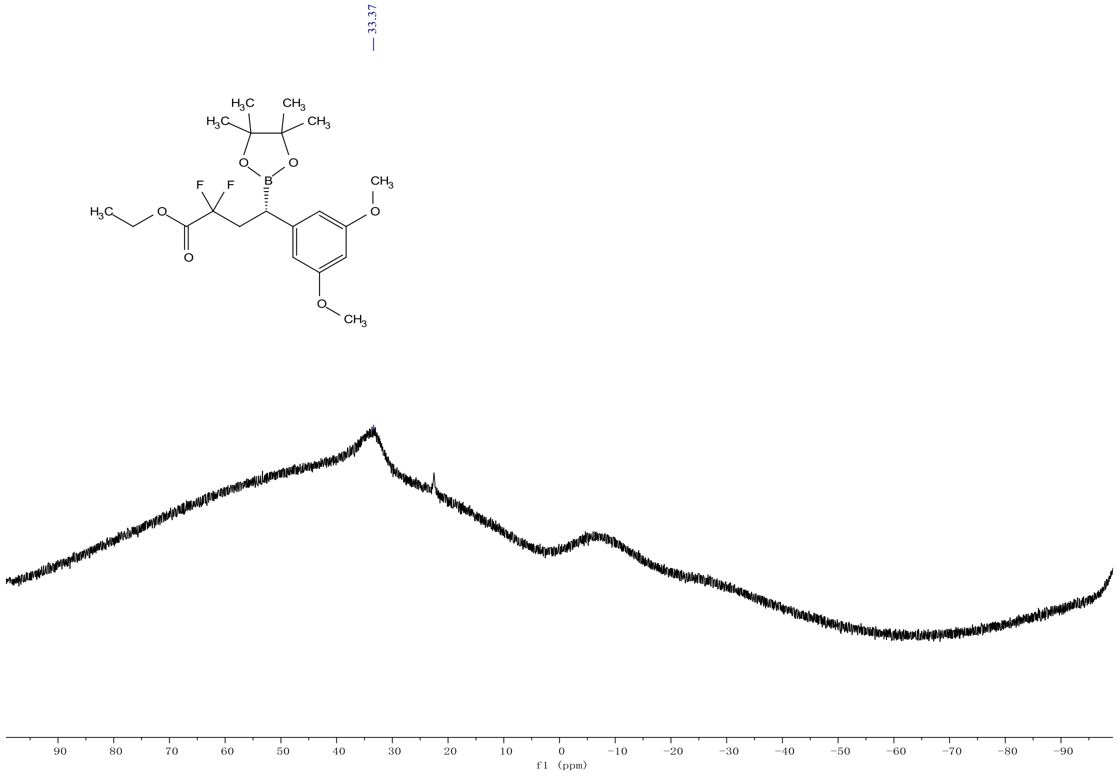

^{11}B-NMR of compound **2g** (128MHz, $CDCl_3$)

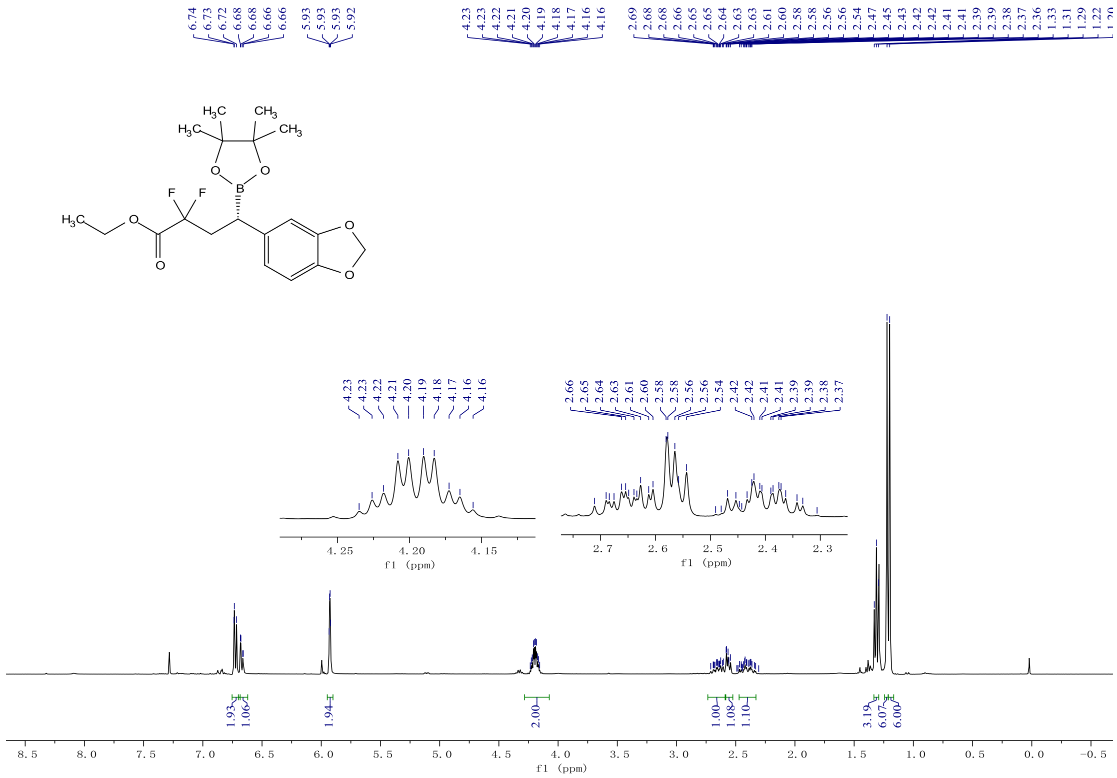

^{1}H-NMR of compound **2h** (400MHz, $CDCl_3$)

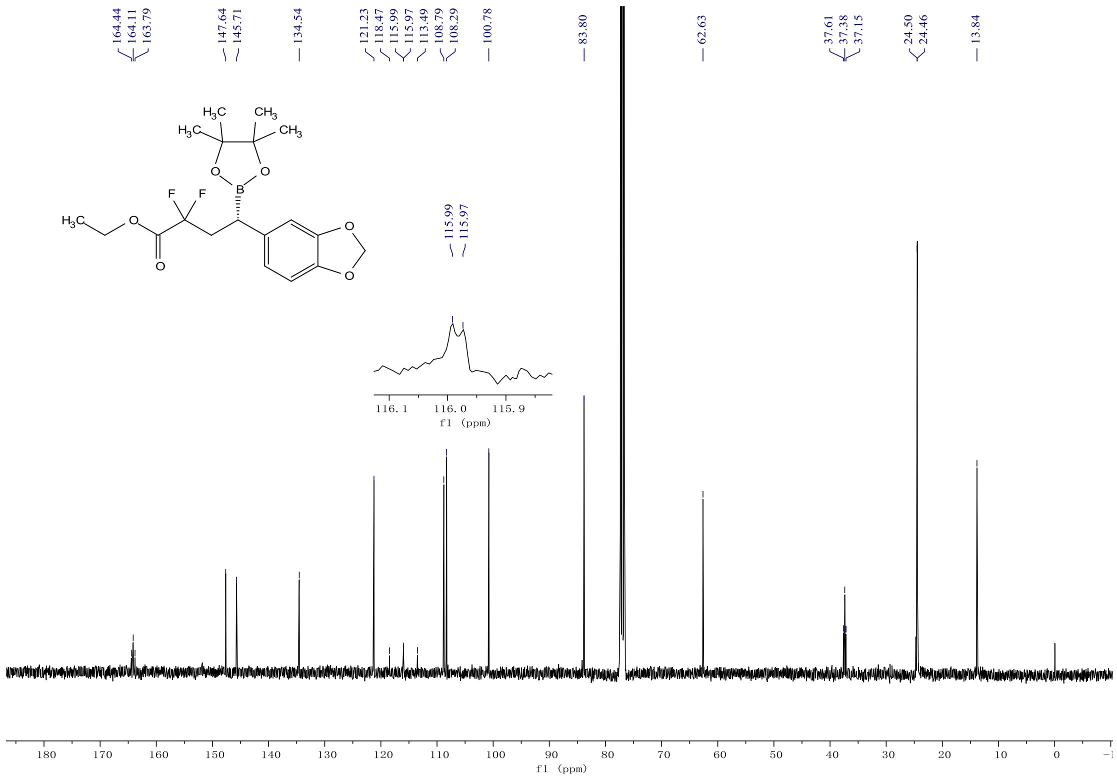

^{13}C{^{1}H}-NMR of compound **2h** (101MHz, $CDCl_3$)

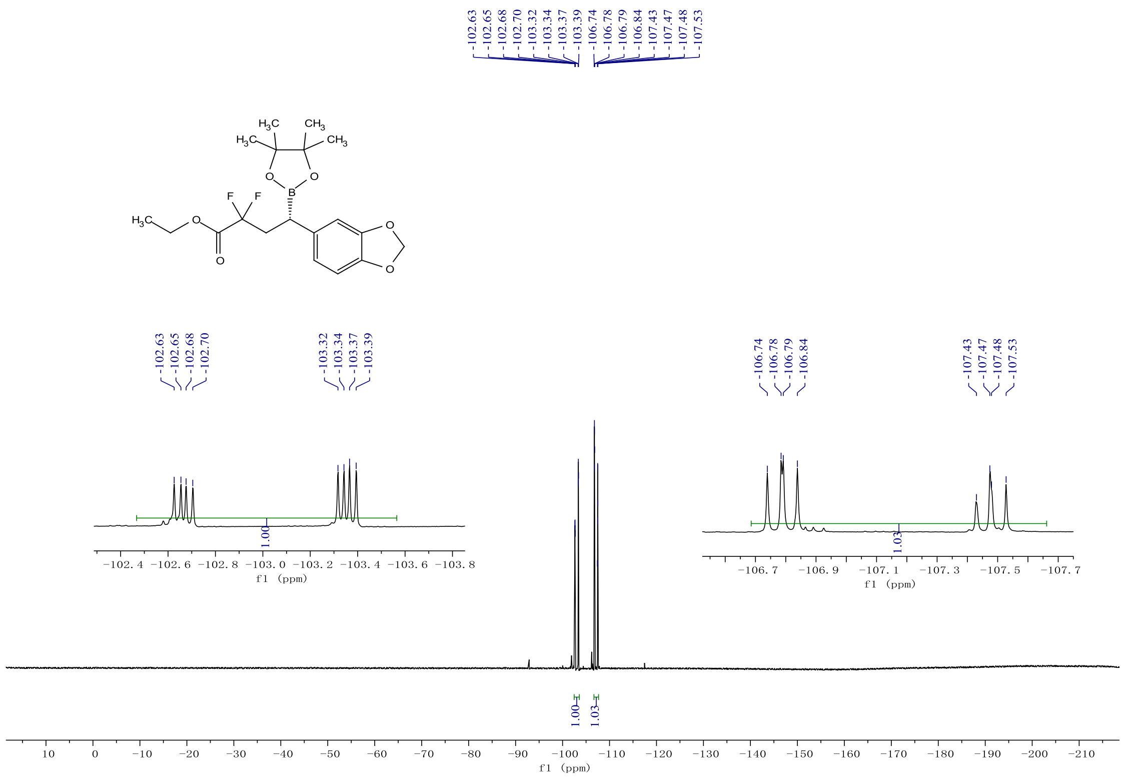

^{19}F-NMR of compound **2h** (377MHz, $CDCl_3$)

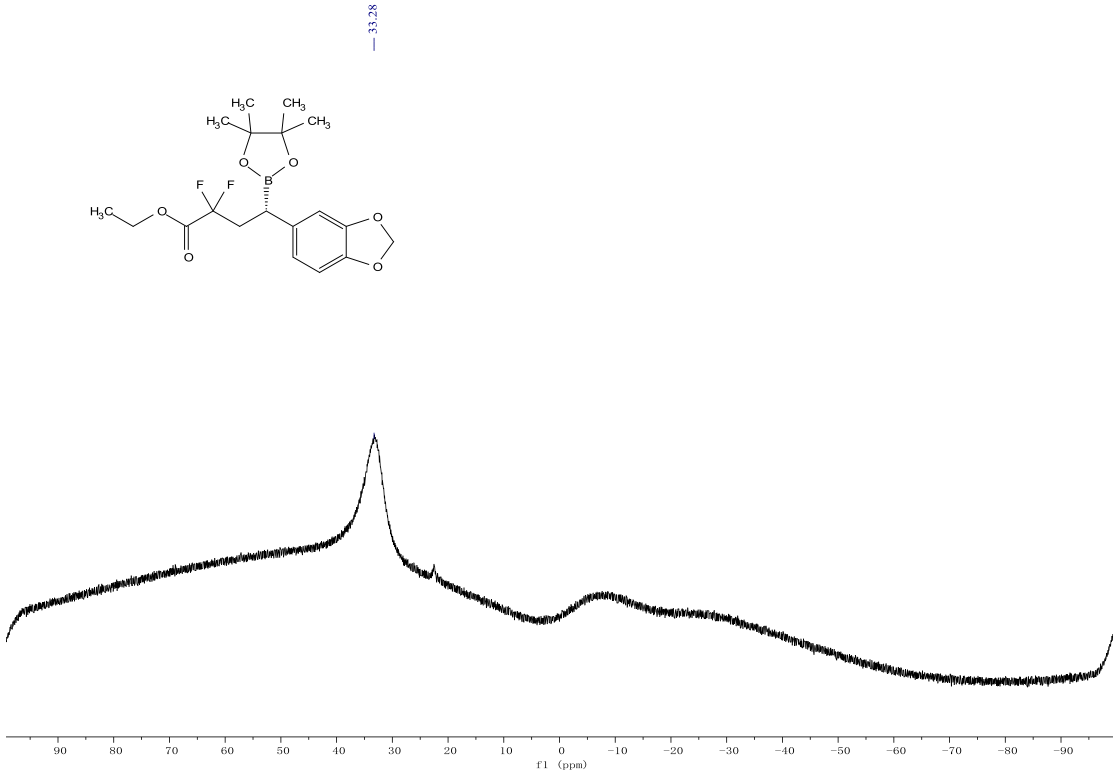

^{11}B-NMR of compound **2h** (128MHz, $CDCl_3$)

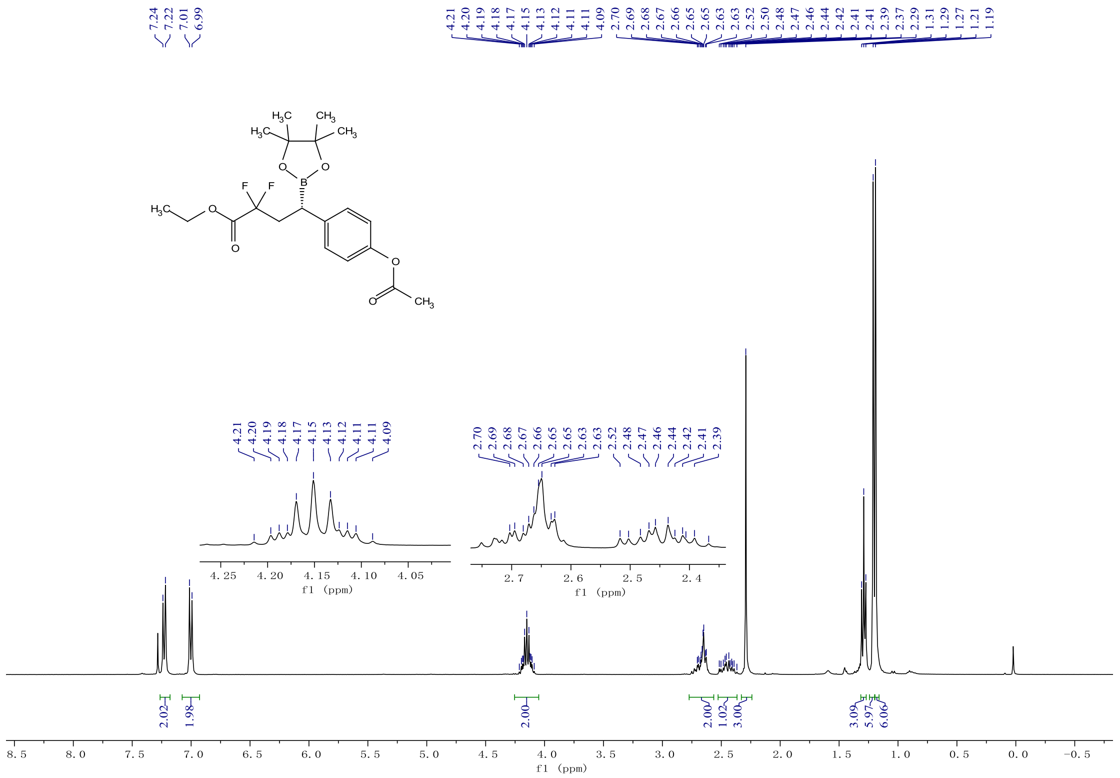

^{1}H-NMR of compound **2i** (400MHz, $CDCl_3$)

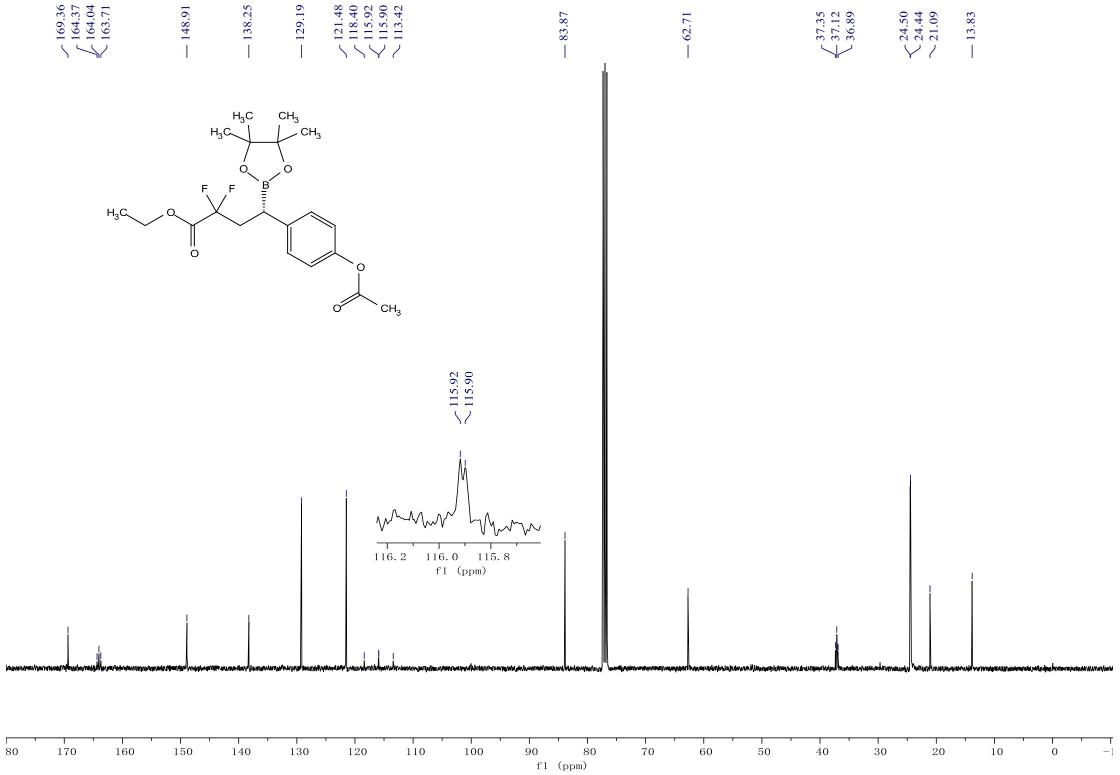

$^{13}C\{^{1}H\}$-NMR of compound **2i** (101MHz, $CDCl_3$)

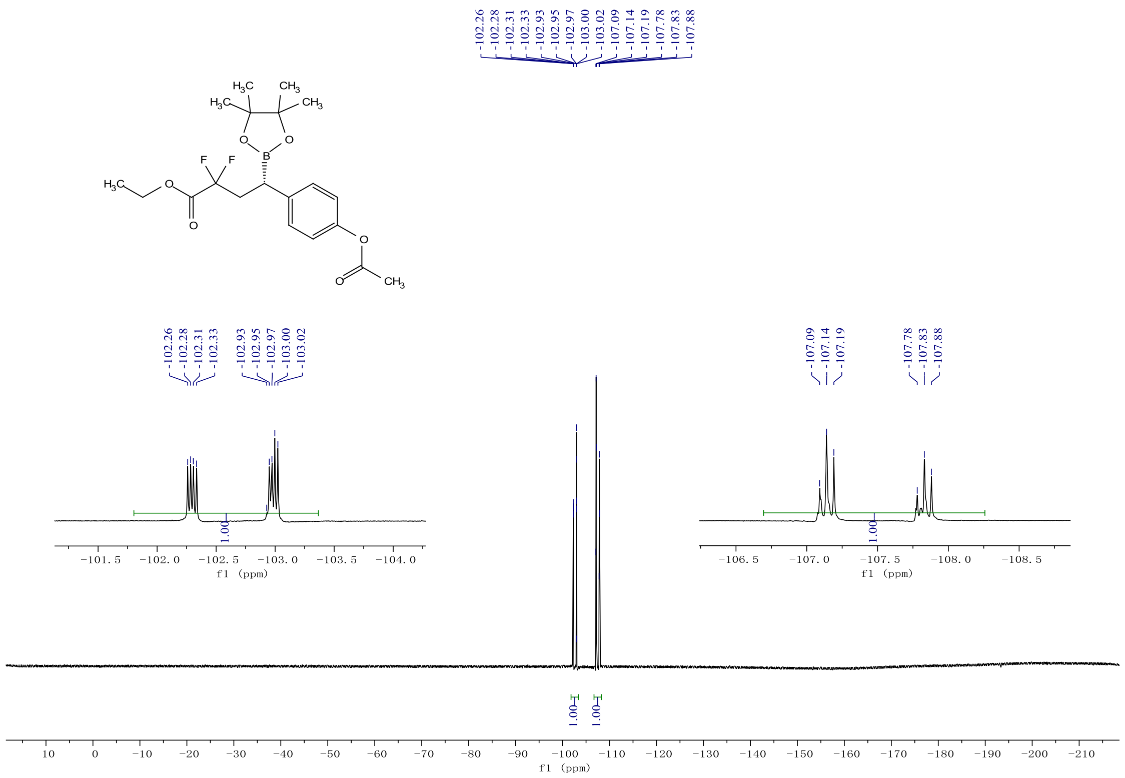

^{19}F-NMR of compound **2i** (377MHz, $CDCl_3$)

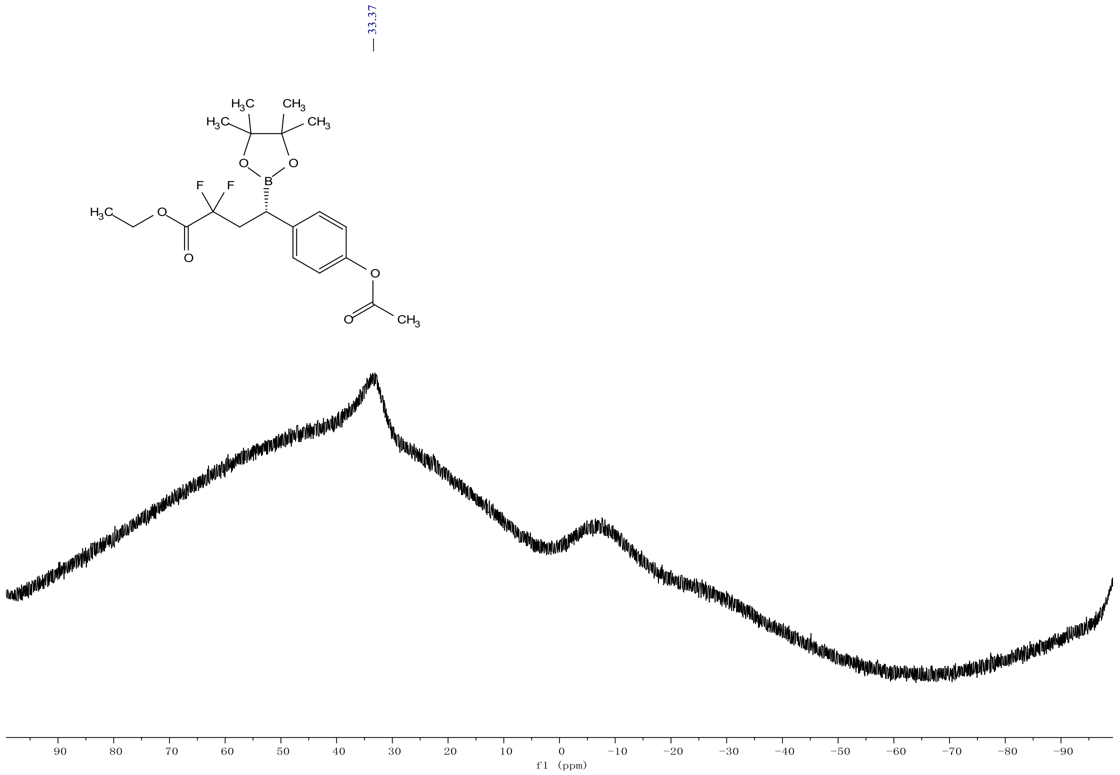

^{11}B-NMR of compound **2i** (128MHz, $CDCl_3$)

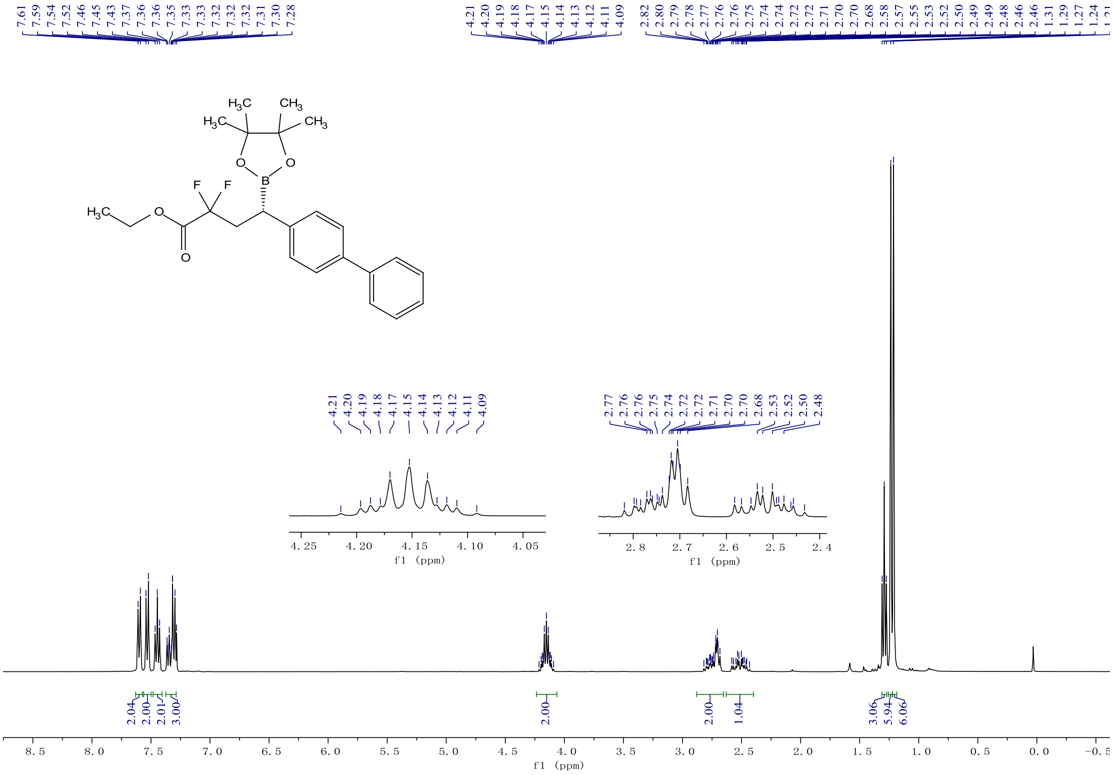

^{1}H-NMR of compound **2j** (400MHz, $CDCl_3$)

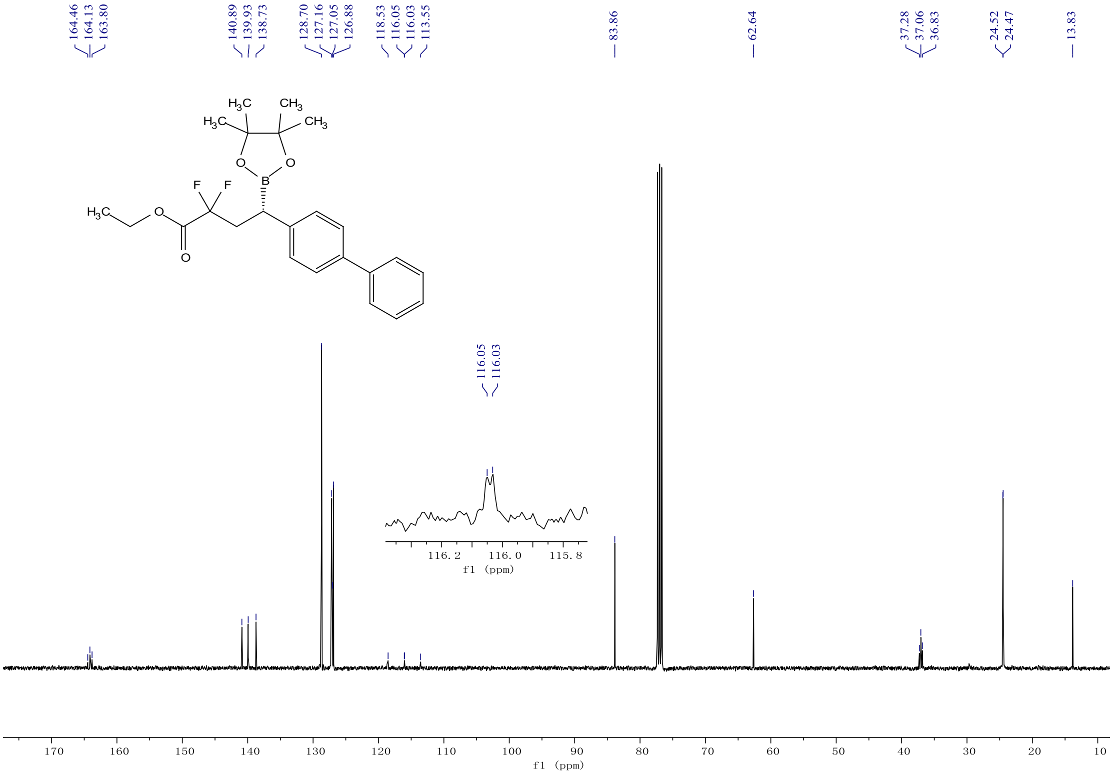

^{13}C{^{1}H}-NMR of compound **2j** (101MHz, $CDCl_3$)

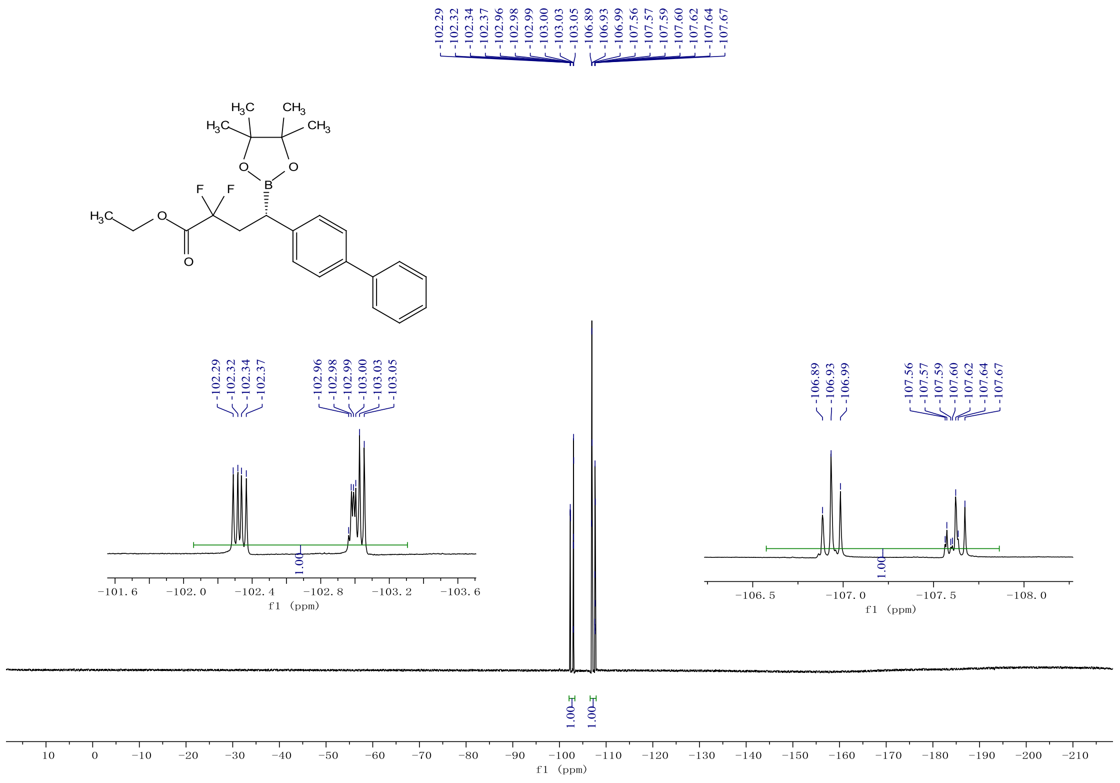

^{19}F-NMR of compound **2j** (377MHz, $CDCl_3$)

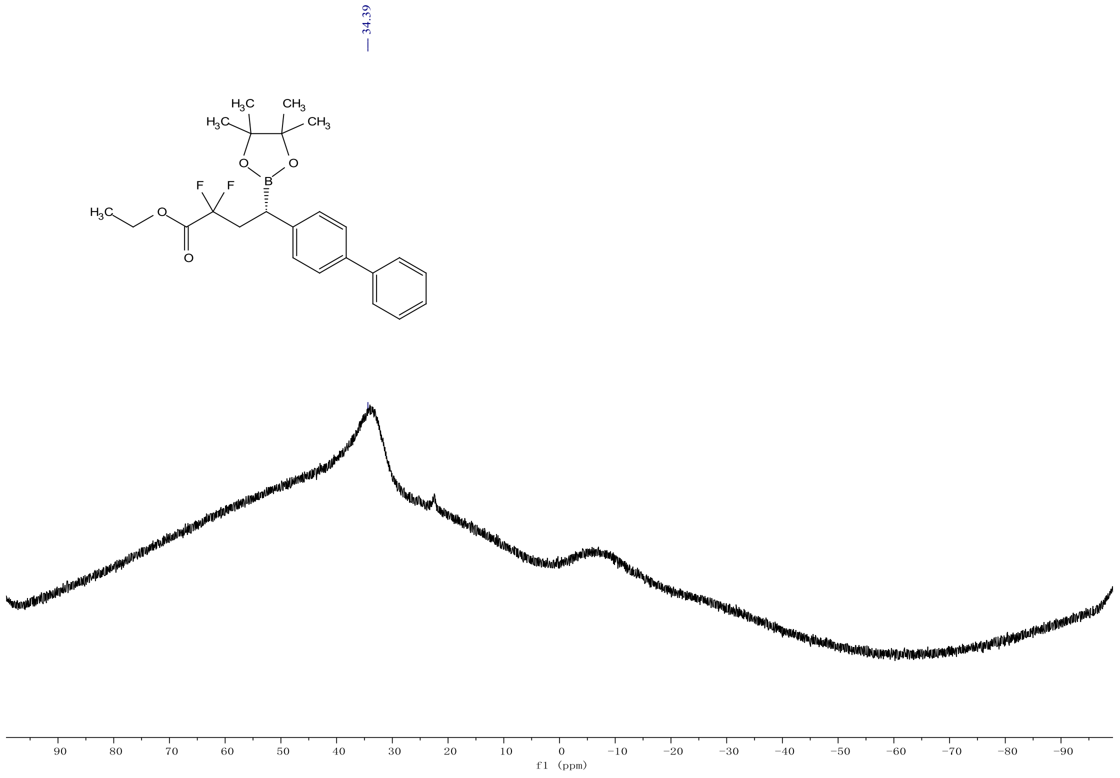

^{11}B-NMR of compound **2j** (128MHz, $CDCl_3$)

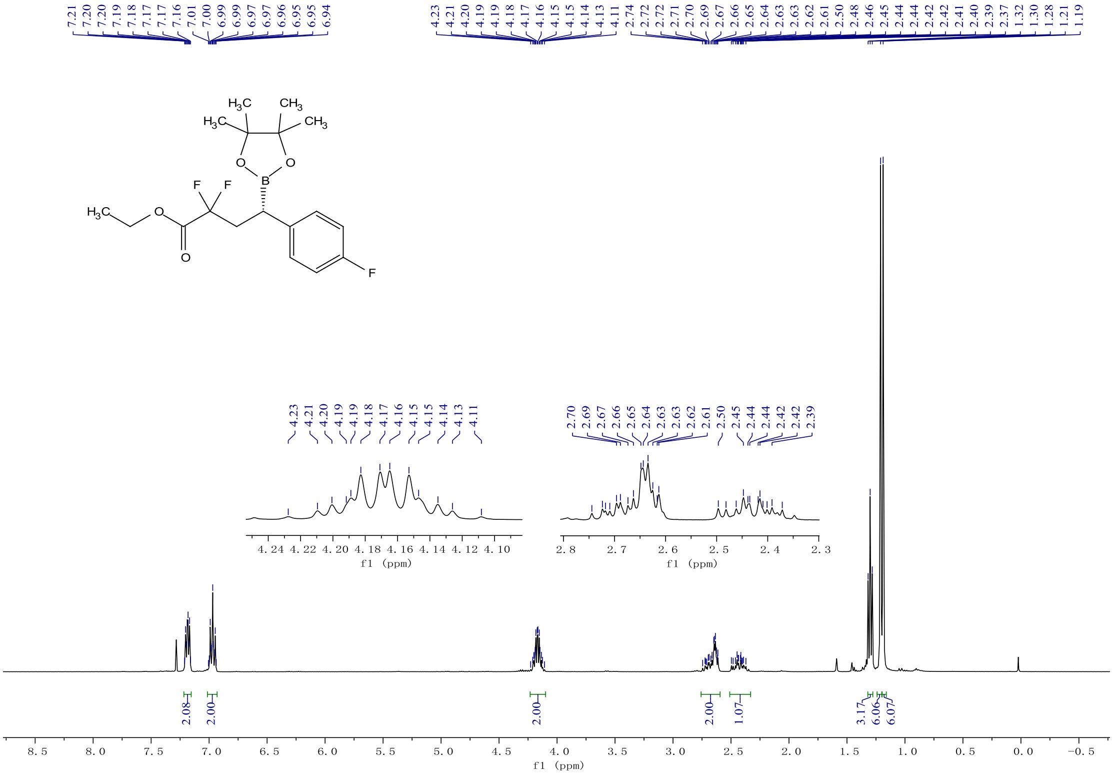

^{1}H-NMR of compound **2k** (400MHz, $CDCl_3$)

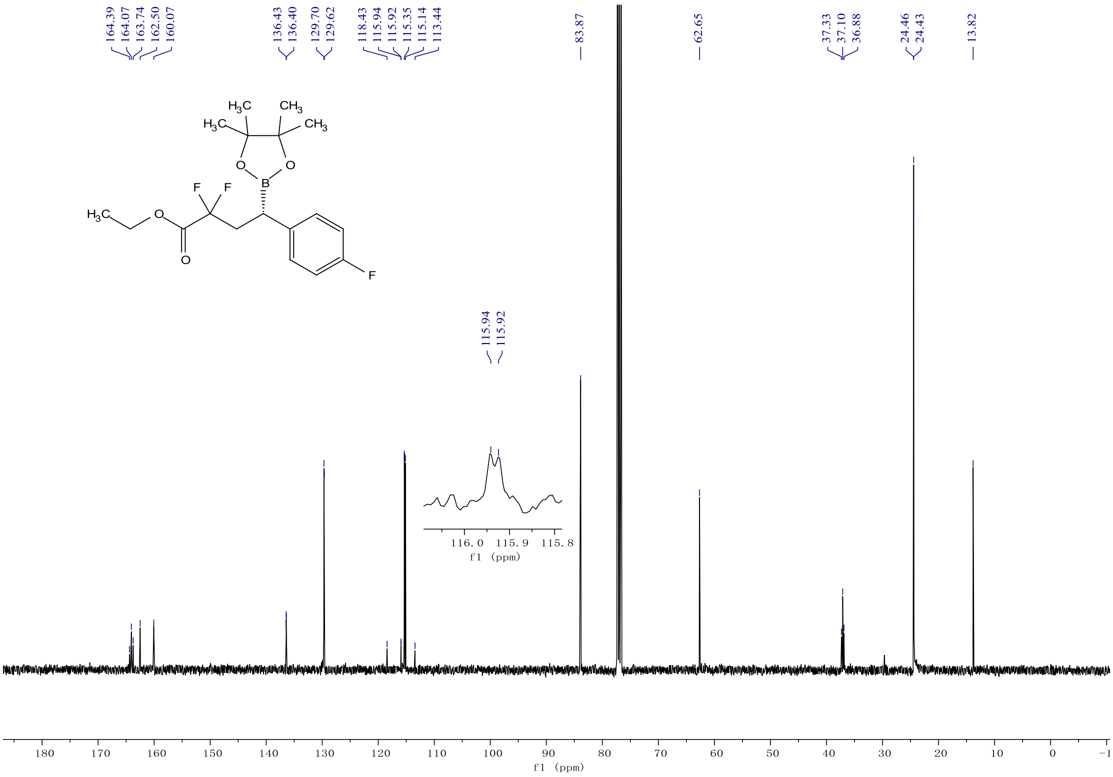

^{13}C{^{1}H}-NMR of compound **2k** (101MHz, $CDCl_3$)

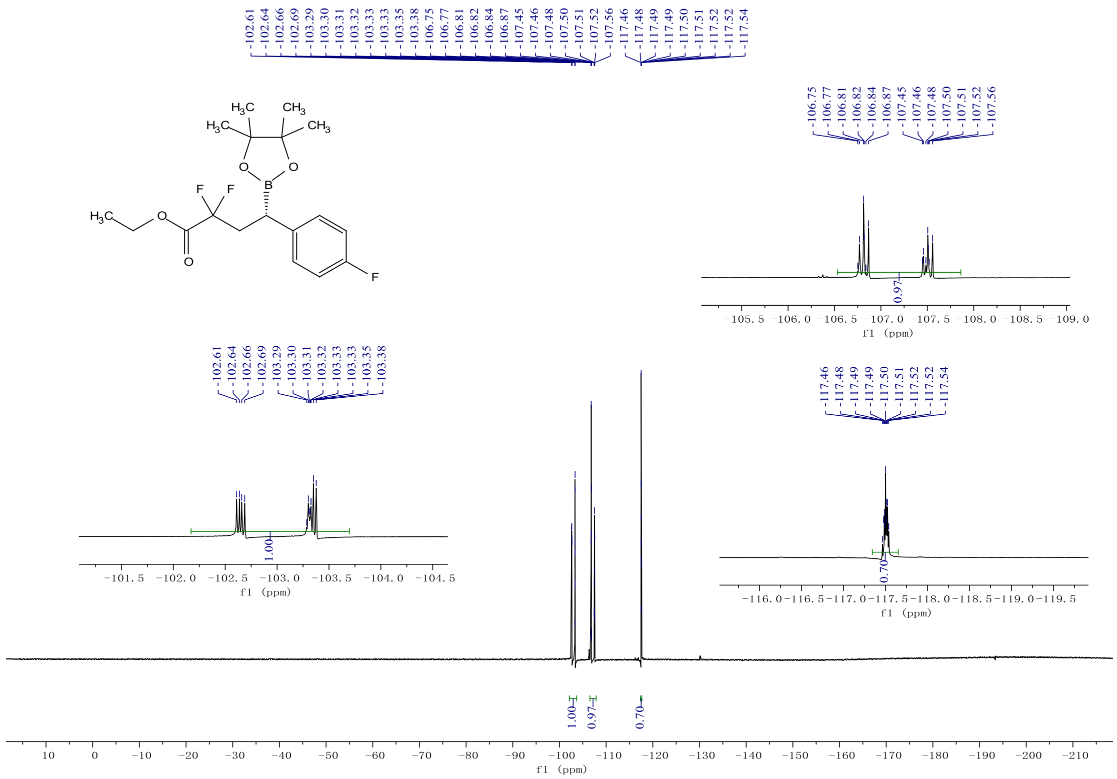

^{19}F-NMR of compound **2k** (377MHz, $CDCl_3$)

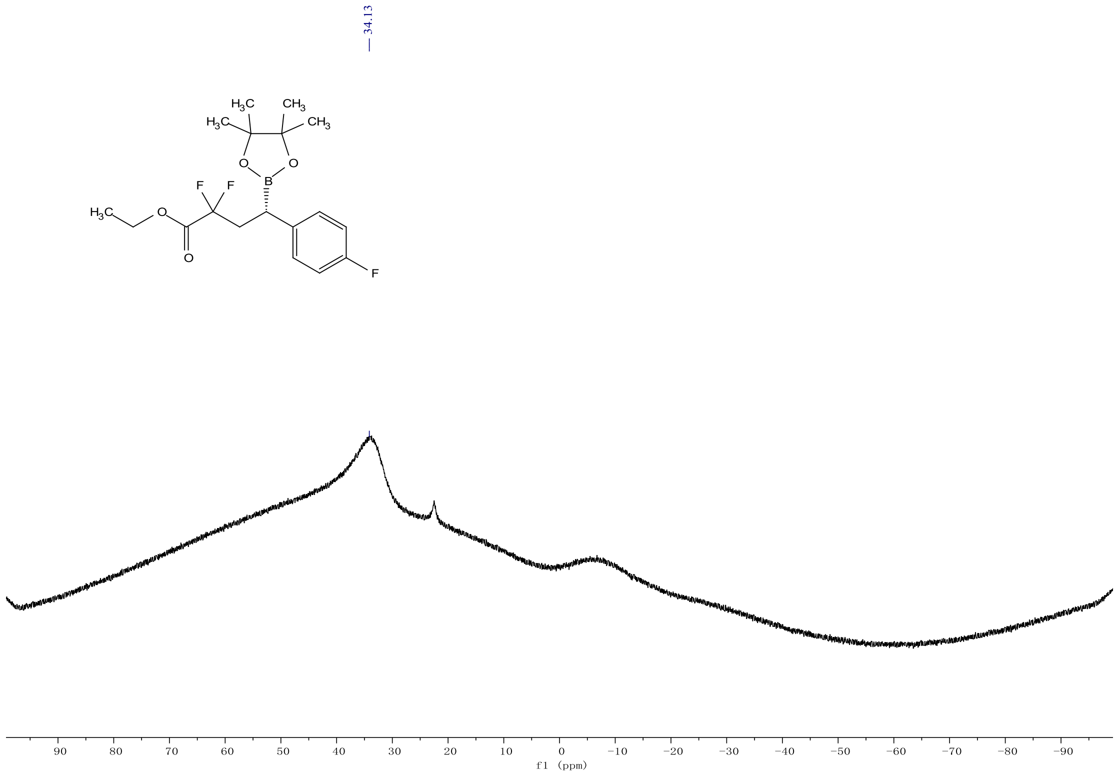

^{11}B-NMR of compound **2k** (128MHz, $CDCl_3$)

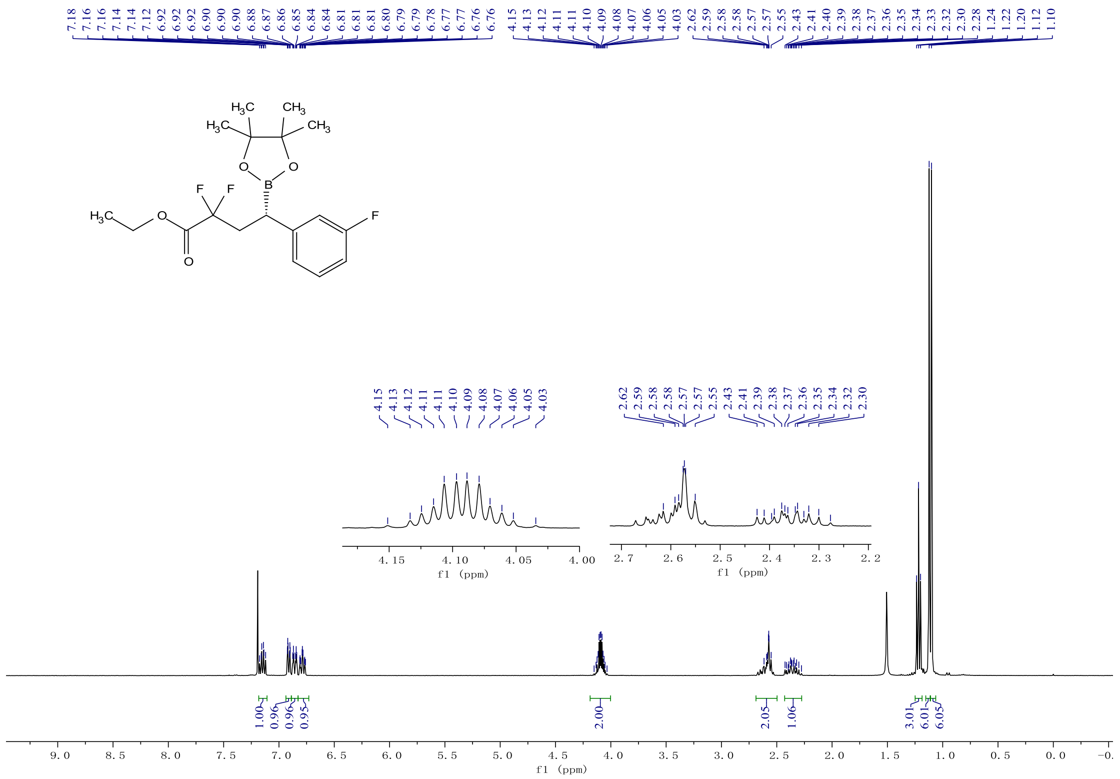

^{1}H-NMR of compound **2l** (400MHz, $CDCl_3$)

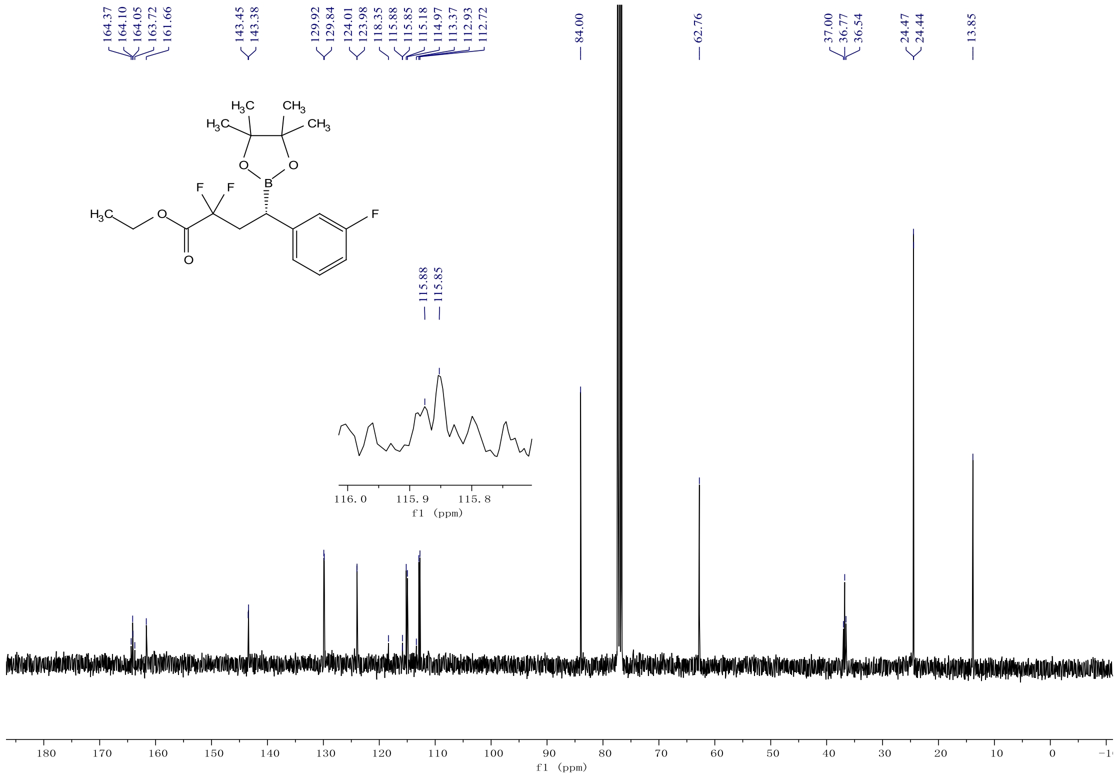

^{13}C{^{1}H}-NMR of compound **2l** (101MHz, $CDCl_3$)

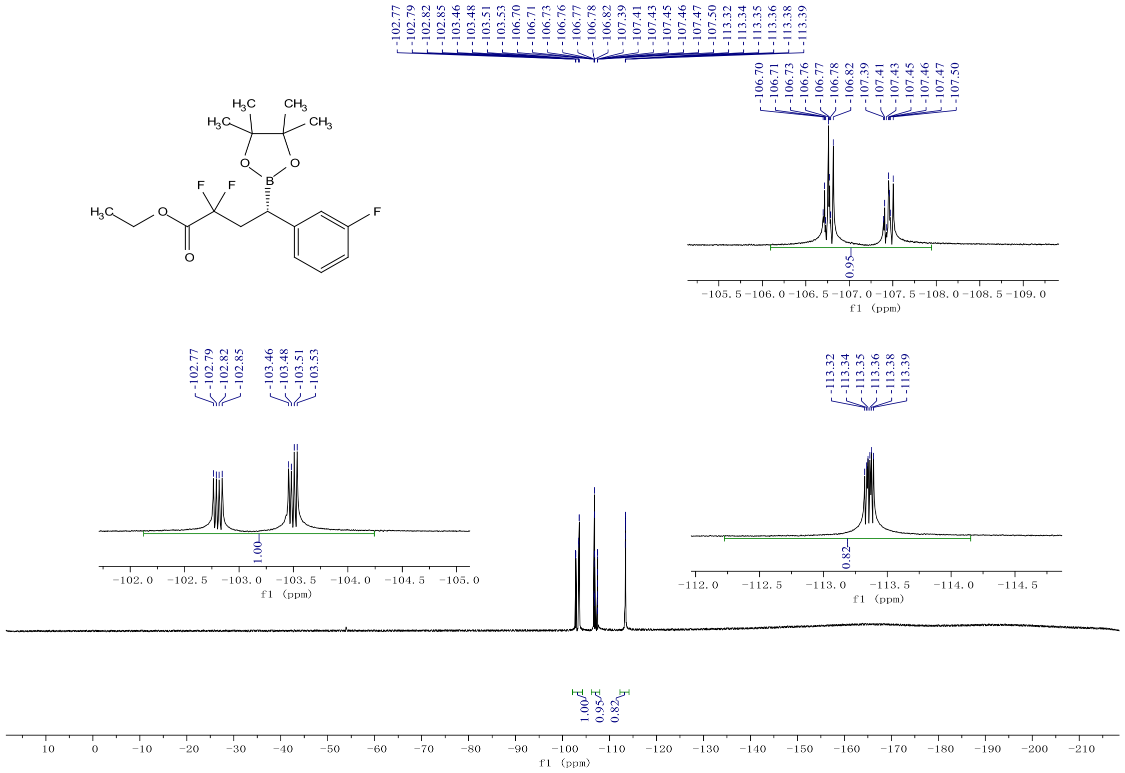

^{19}F-NMR of compound **2l** (377MHz, $CDCl_3$)

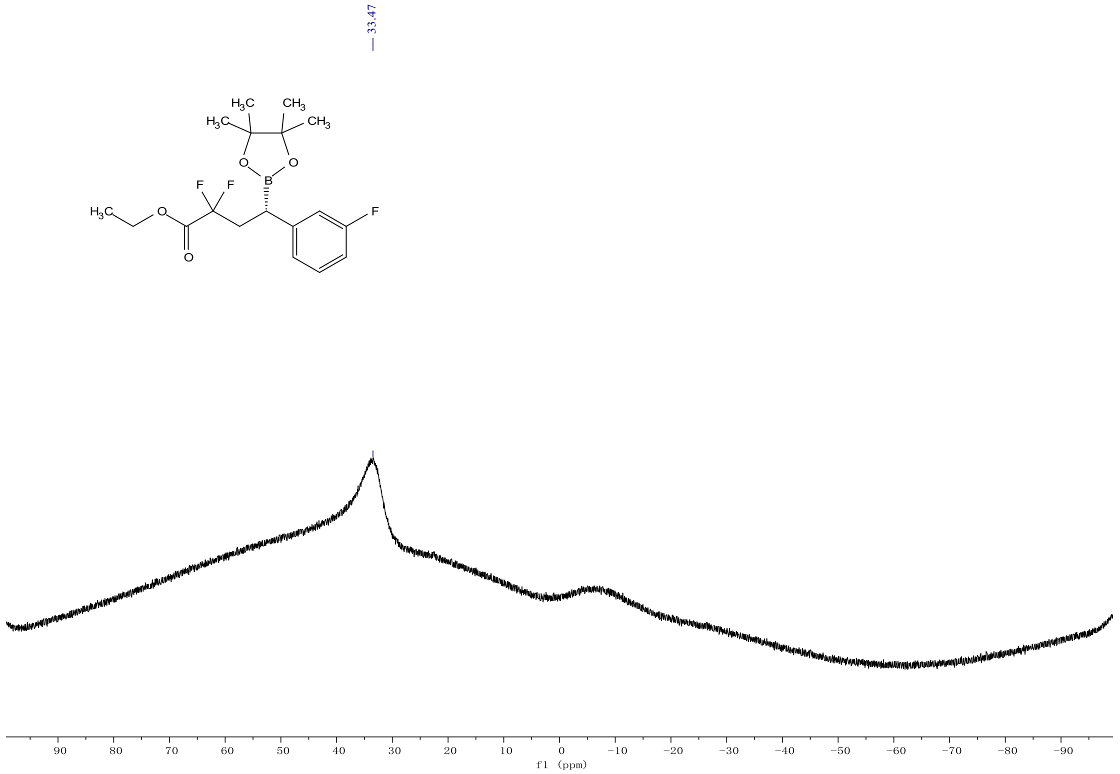

^{11}B-NMR of compound **2l** (128MHz, $CDCl_3$)

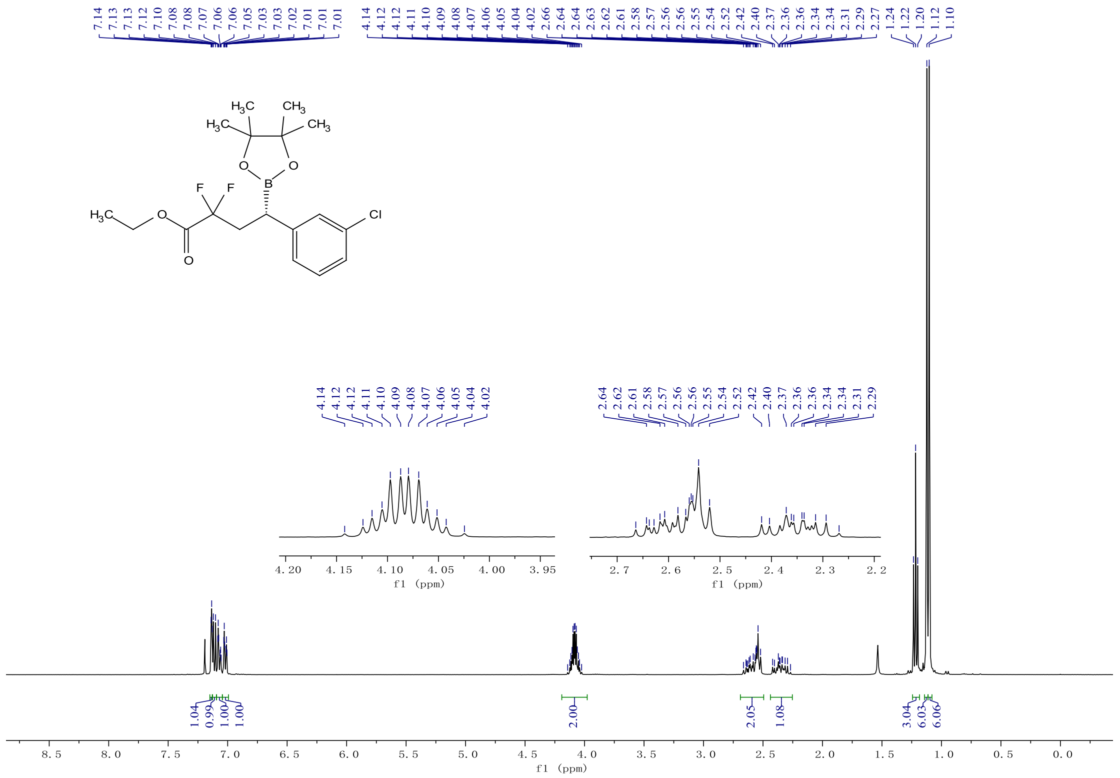

^{1}H-NMR of compound **2m** (400MHz, $CDCl_3$)

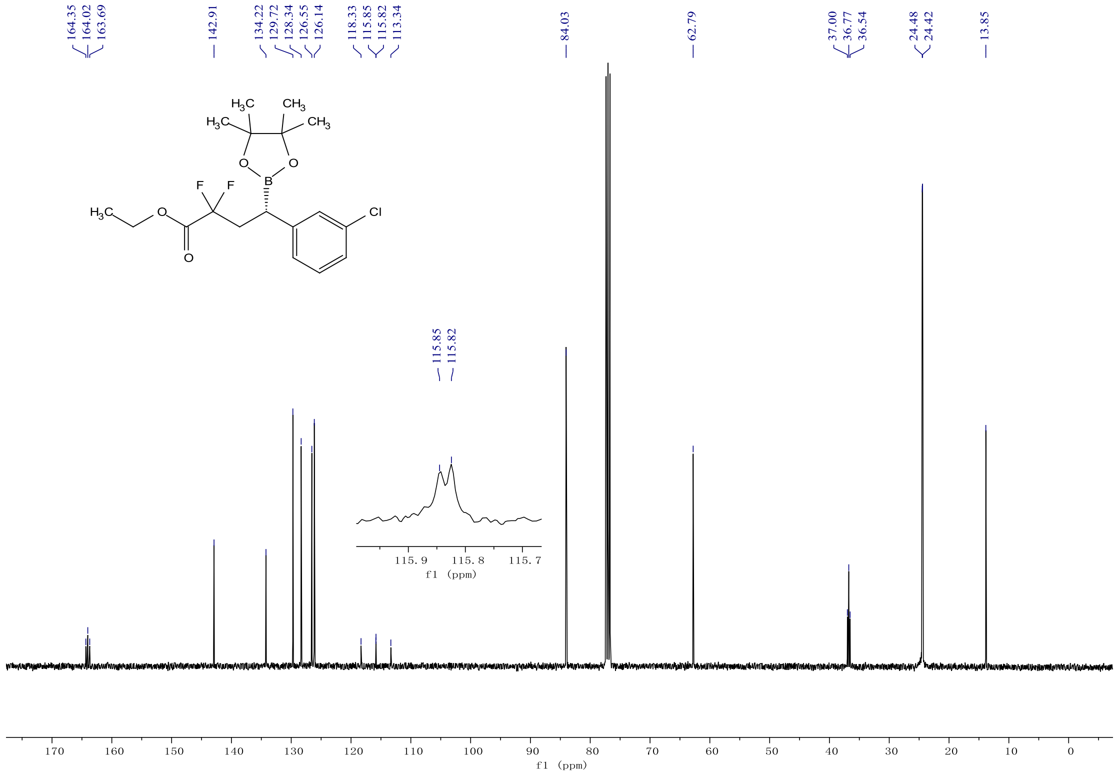

^{13}C{^{1}H}-NMR of compound **2m** (101MHz, $CDCl_3$)

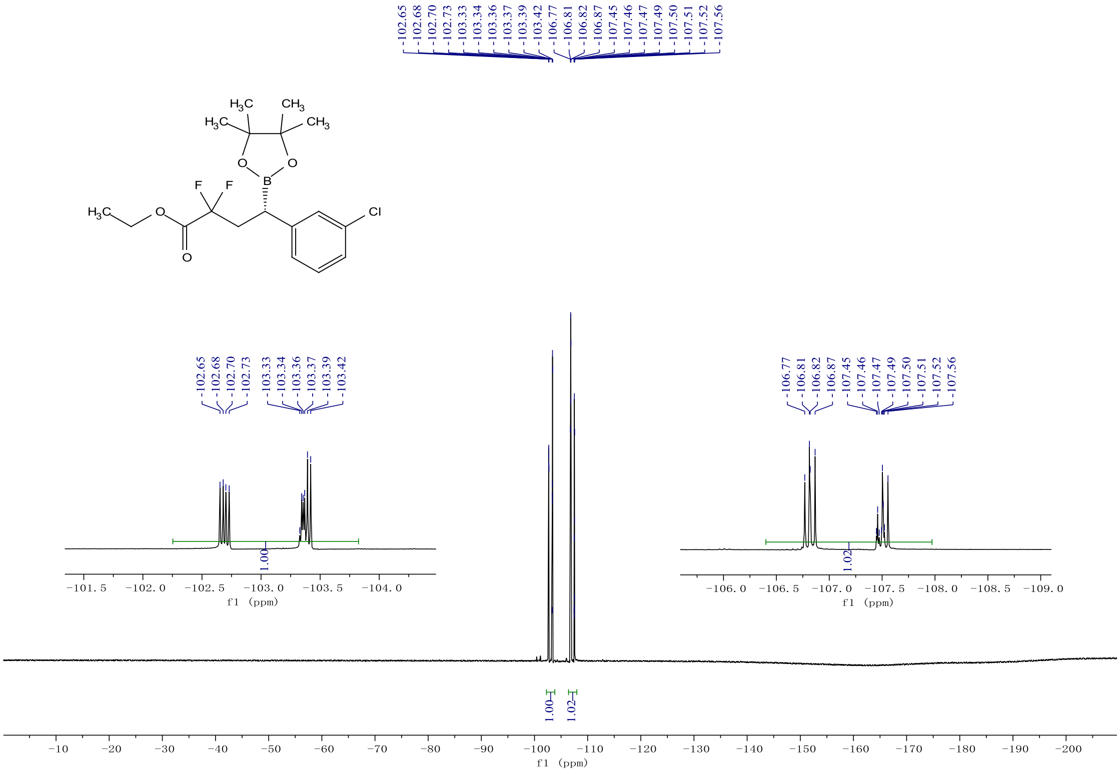

^{19}F-NMR of compound **2m** (377MHz, $CDCl_3$)

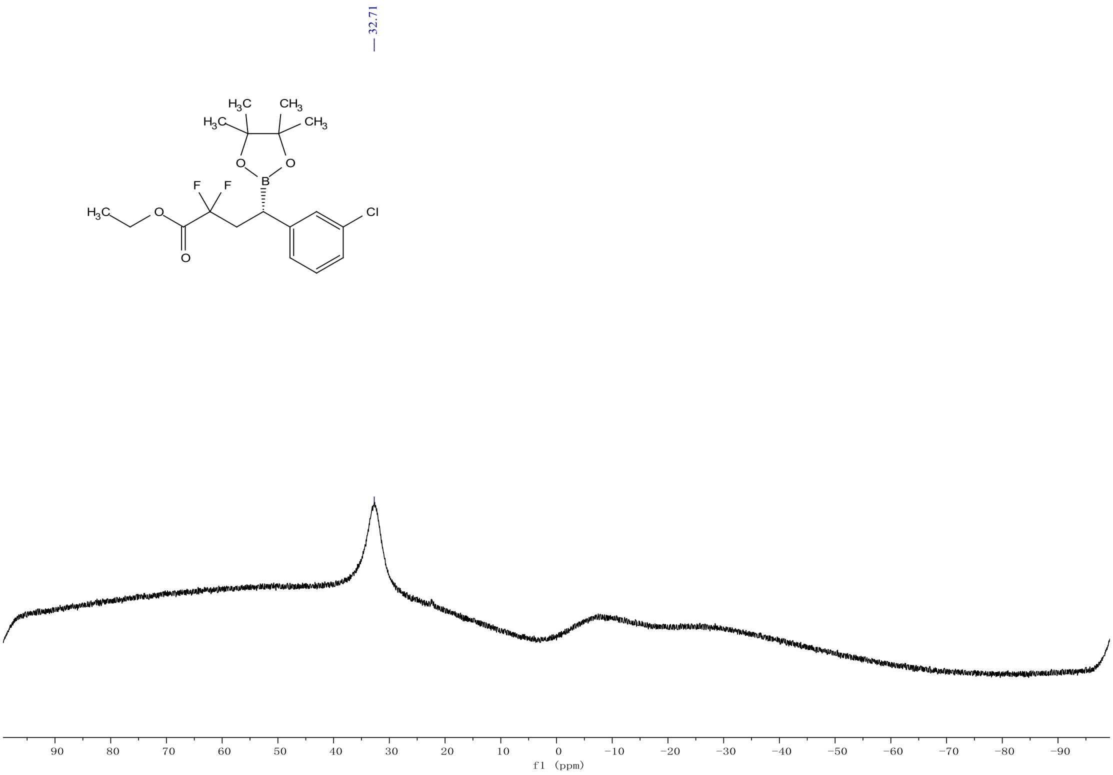

^{11}B-NMR of compound **2m** (128MHz, $CDCl_3$)

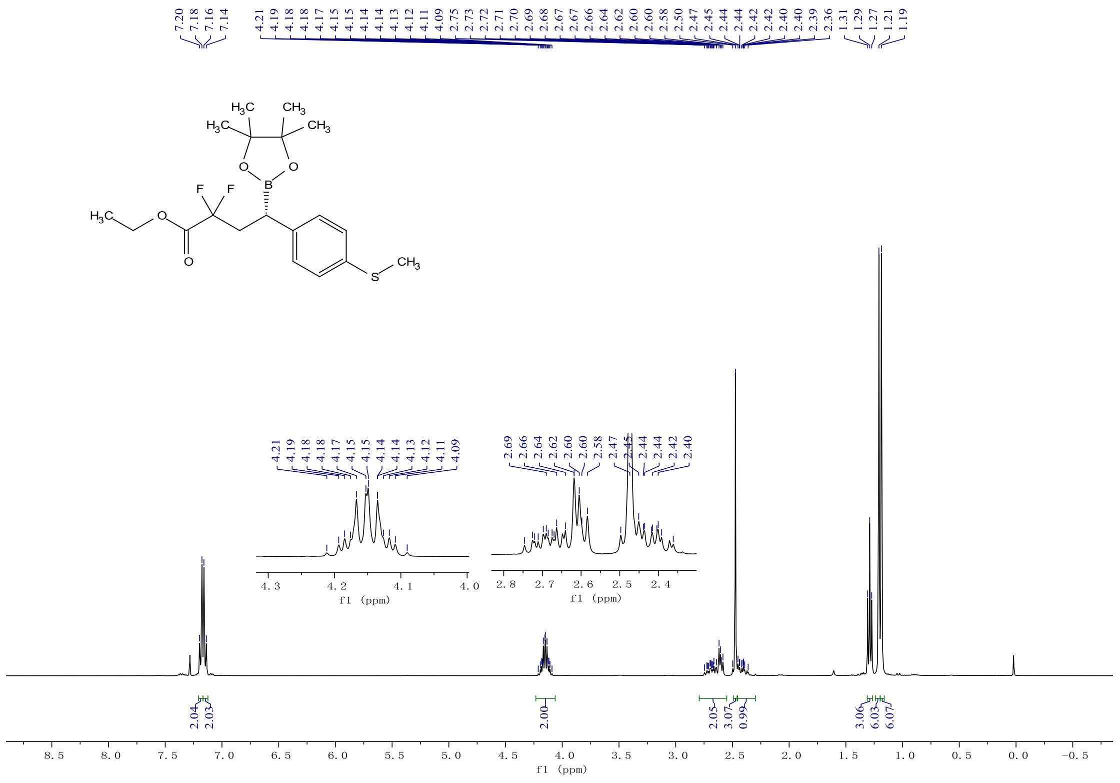

^{1}H-NMR of compound **2n** (400MHz, $CDCl_3$)

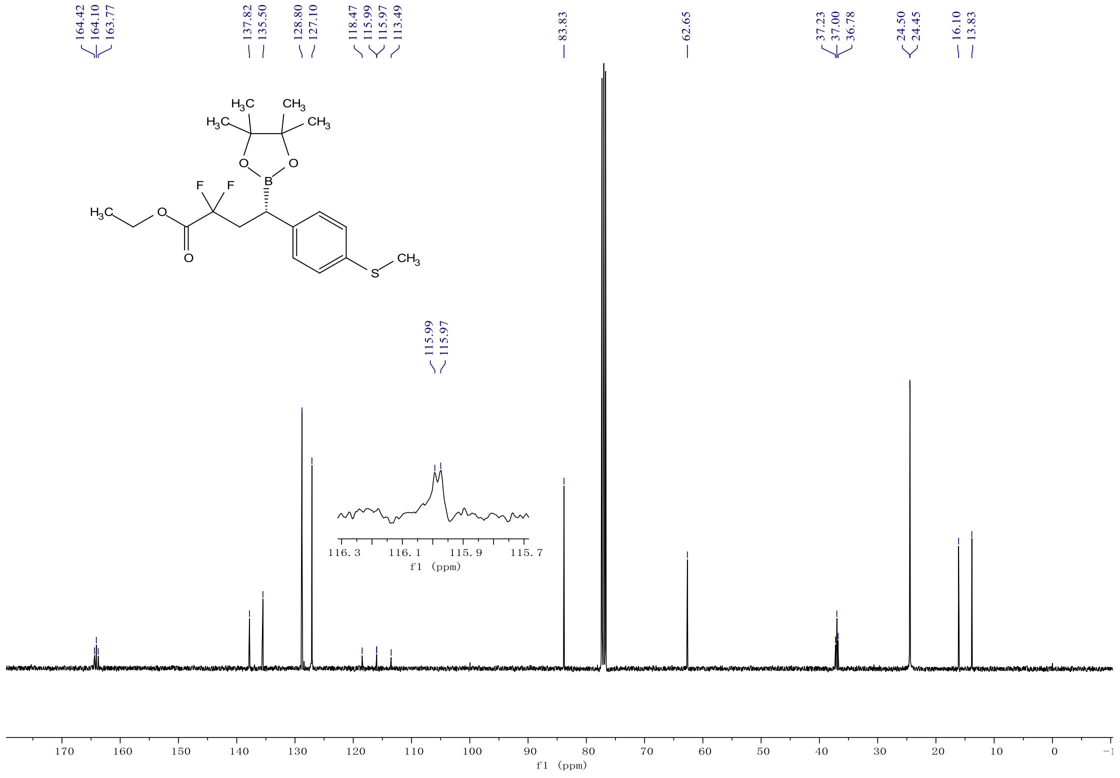

$^{13}C\{^{1}H\}$-NMR of compound **2n** (101MHz, $CDCl_3$)

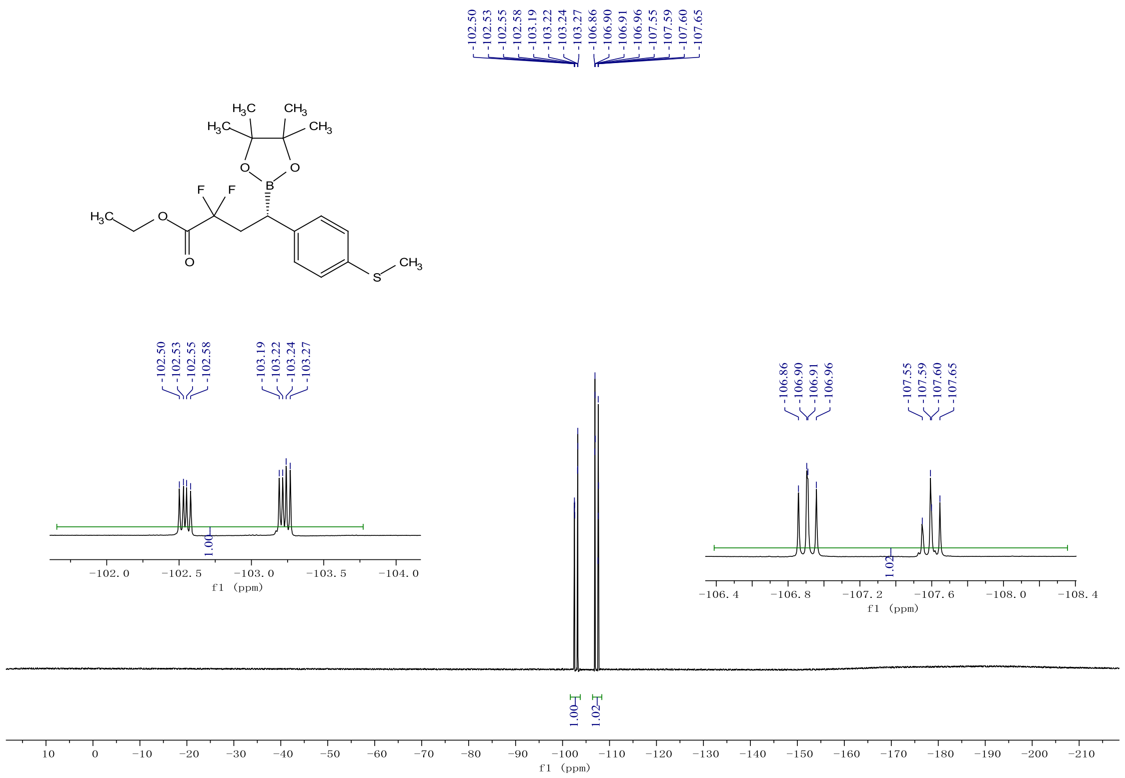

^{19}F-NMR of compound **2n** (377MHz, $CDCl_3$)

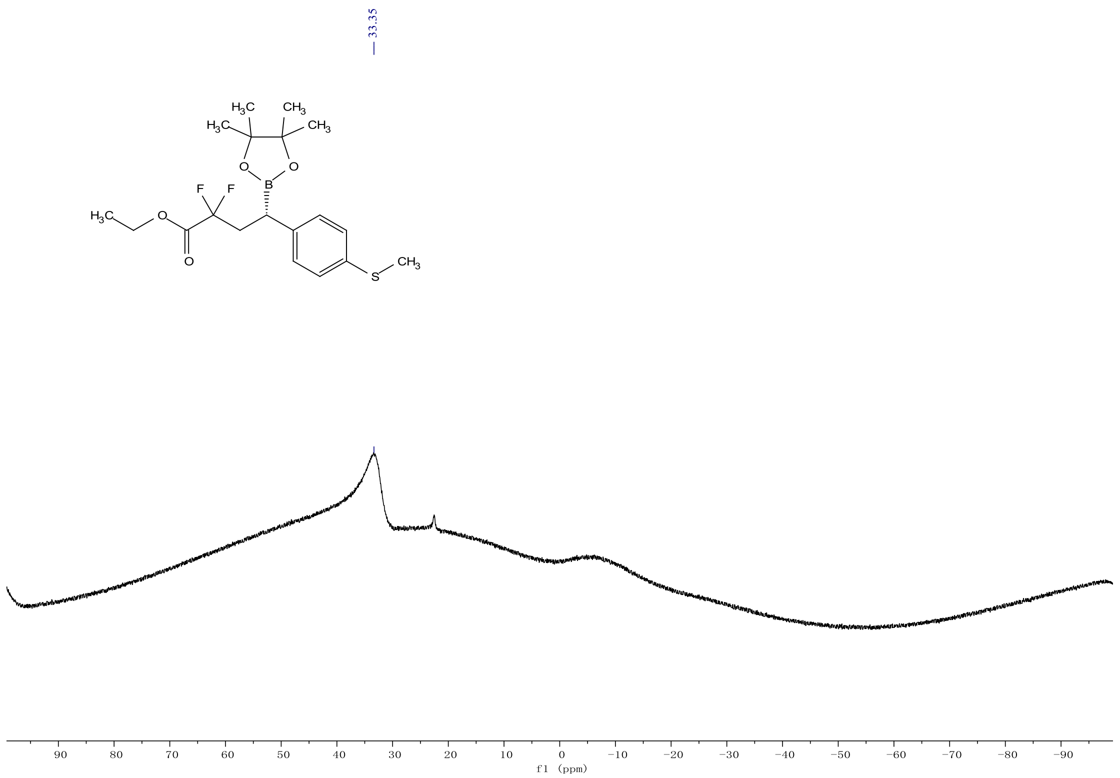

^{11}B-NMR of compound **2n** (128MHz, $CDCl_3$)

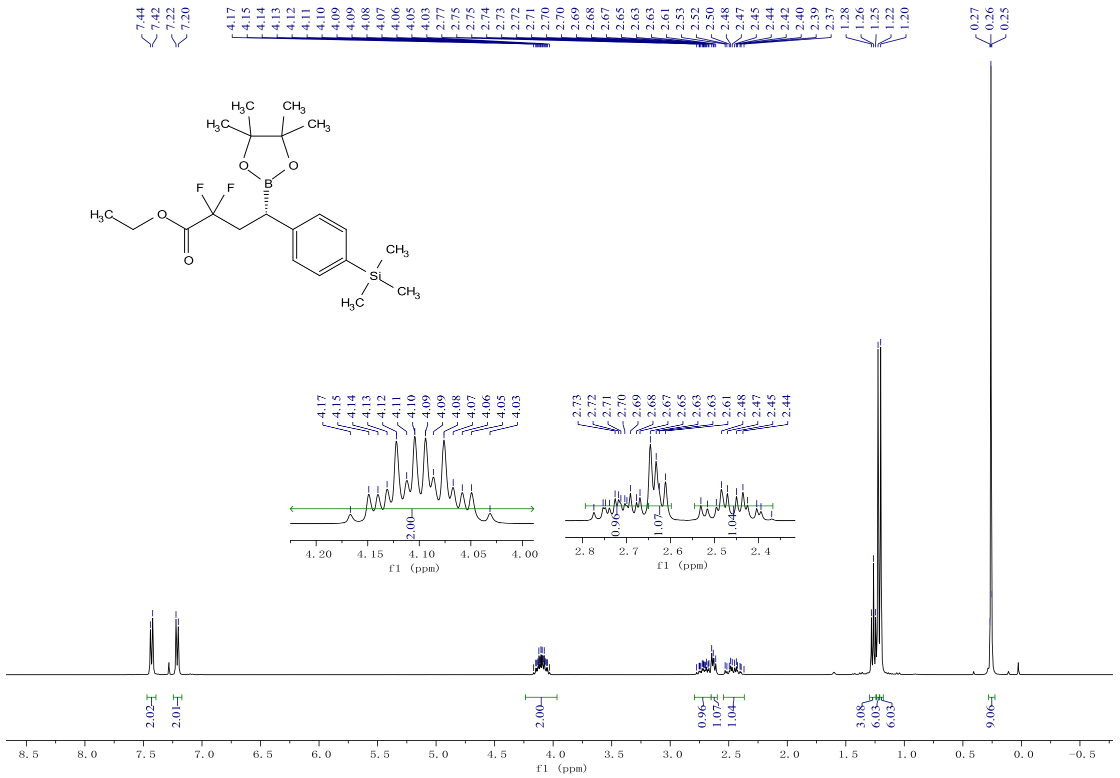

^{1}H-NMR of compound **2o** (400MHz, $CDCl_3$)

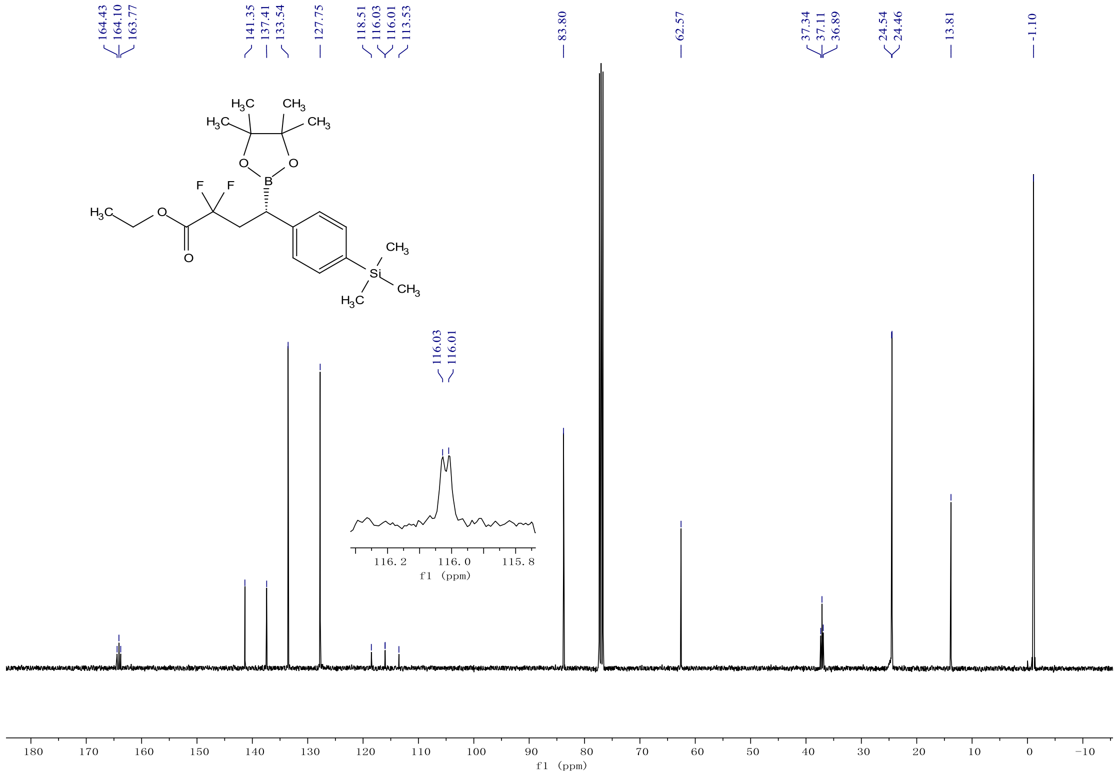

^{13}C{^{1}H}-NMR of compound **2o** (101MHz, $CDCl_3$)

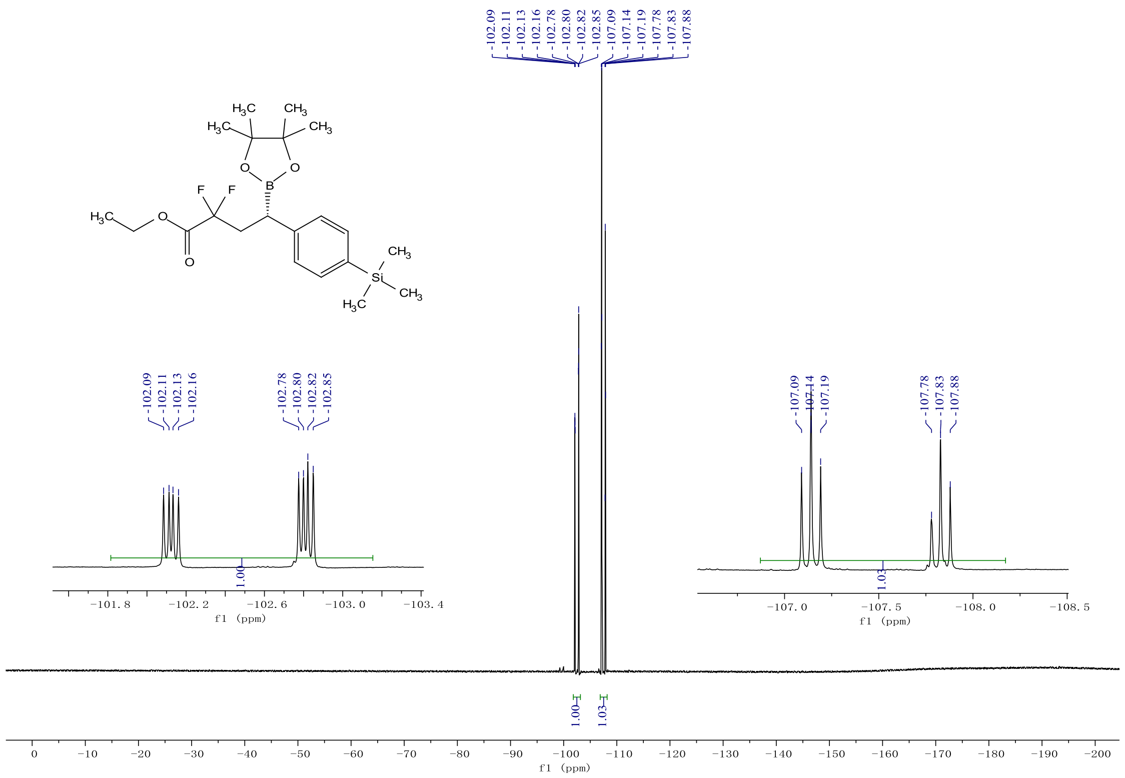

^{19}F-NMR of compound **2o** (377MHz, $CDCl_3$)

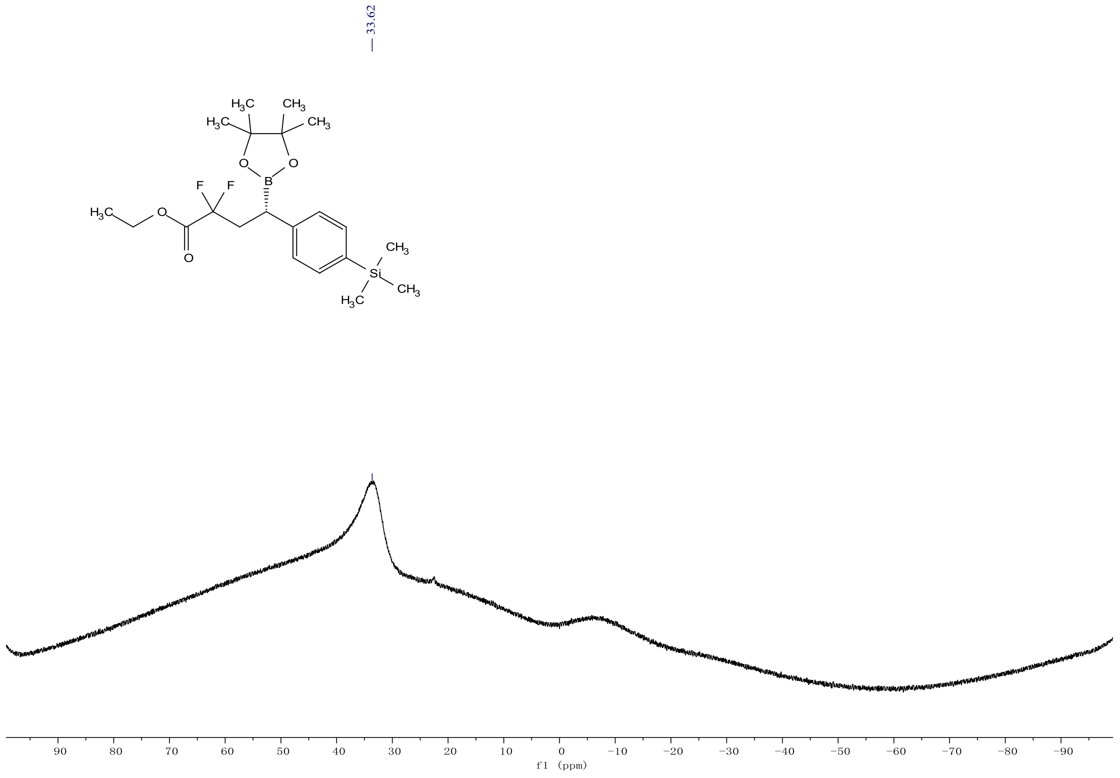

^{11}B-NMR of compound **2o** (128MHz, $CDCl_3$)

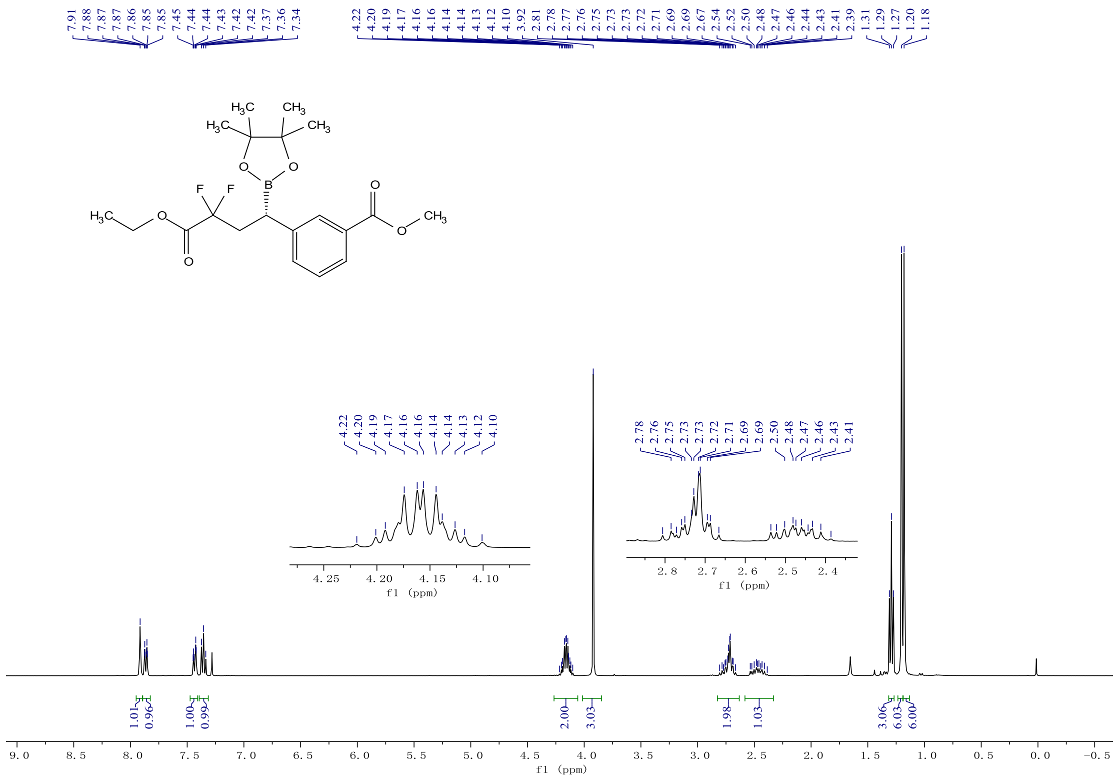

^{1}H-NMR of compound **2p** (400MHz, $CDCl_3$)

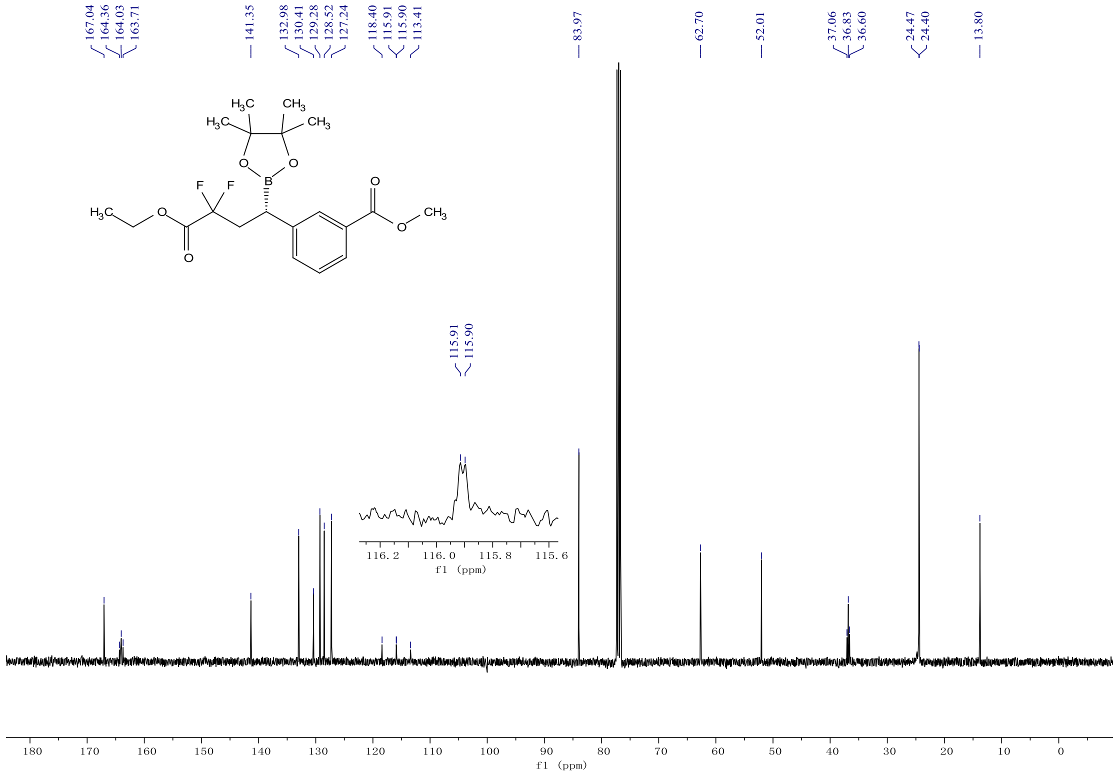

^{13}C{^{1}H}-NMR of compound **2p** (101MHz, $CDCl_3$)

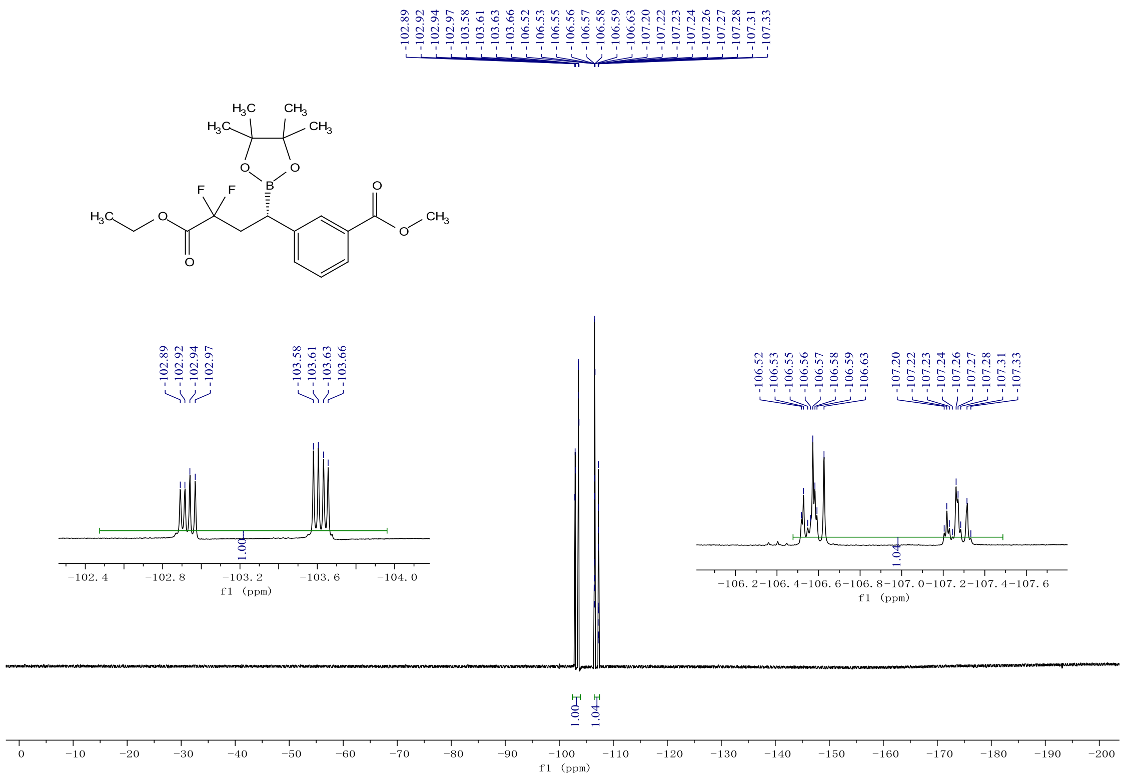

^{19}F-NMR of compound **2p** (377MHz, $CDCl_3$)

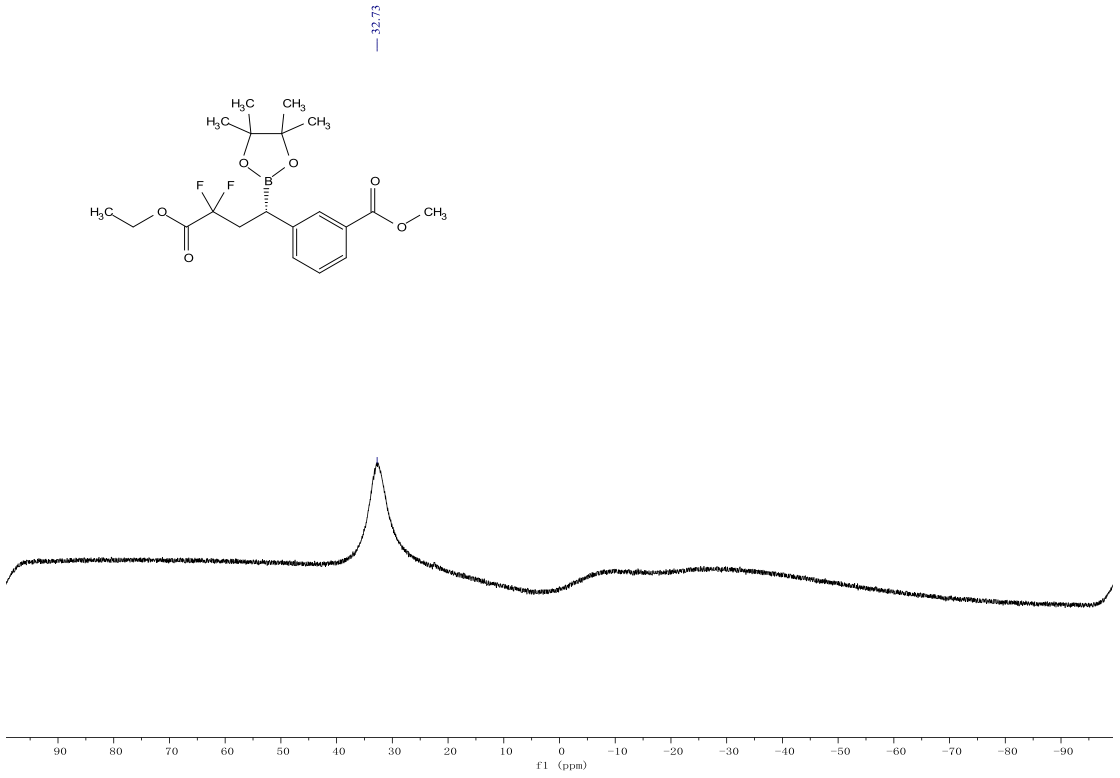

^{11}B-NMR of compound **2p** (128MHz, $CDCl_3$)

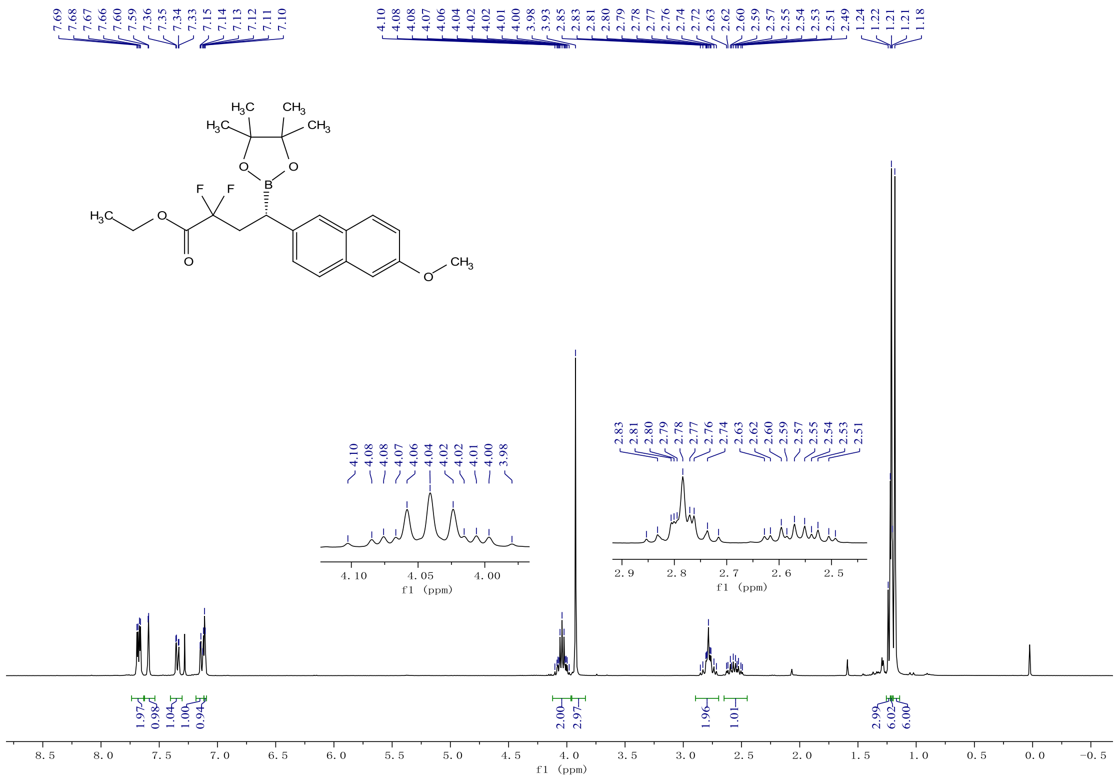

^{1}H-NMR of compound **2q** (400MHz, $CDCl_3$)

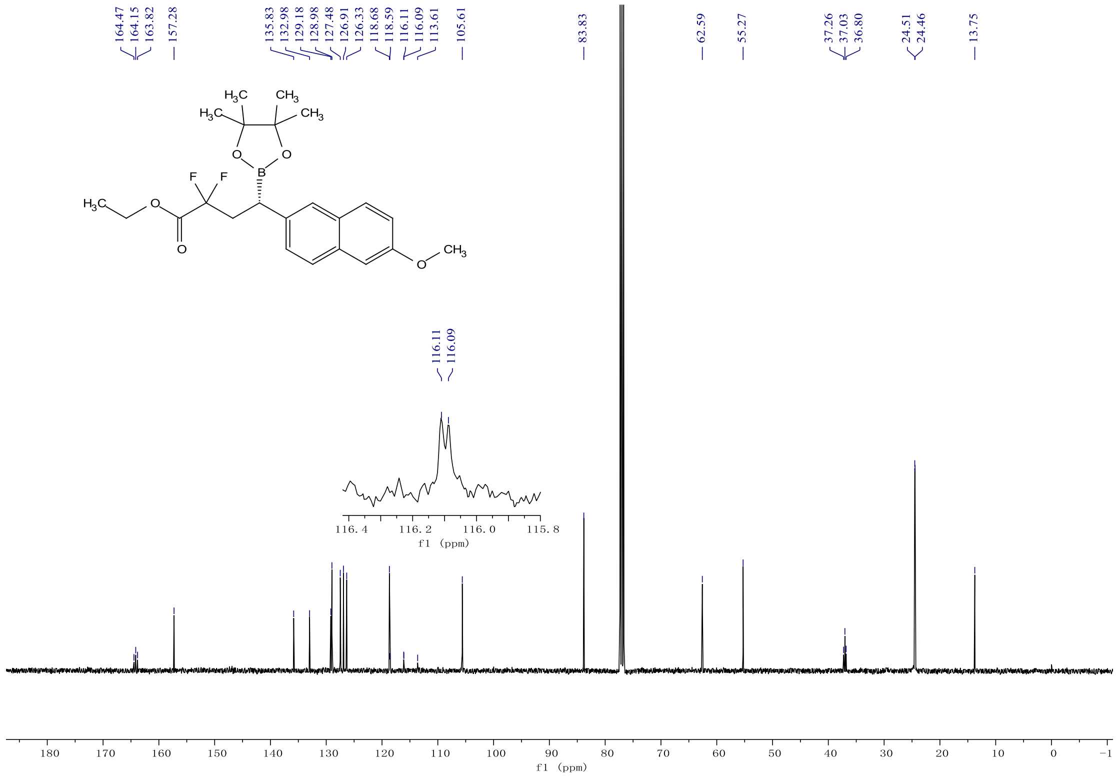

^{13}C{^{1}H}-NMR of compound **2q** (101MHz, $CDCl_3$)

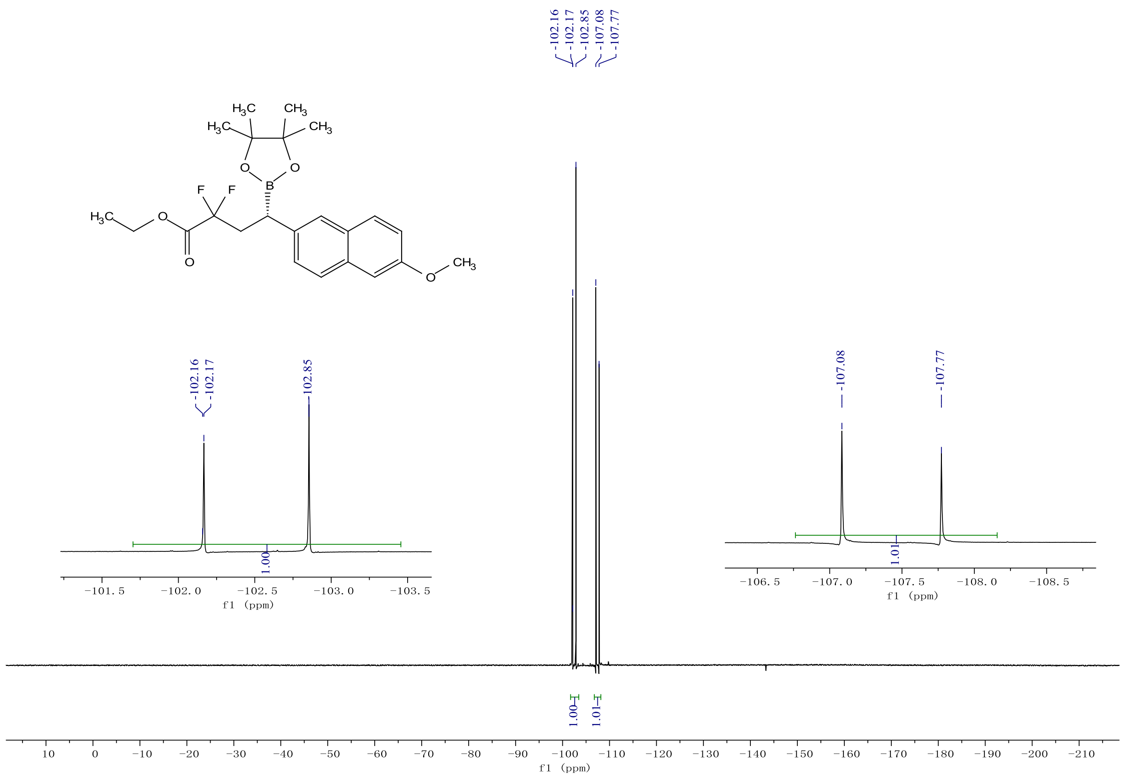

$^{19}F\{^1H\}$-NMR of compound **2q** (377MHz, $CDCl_3$)

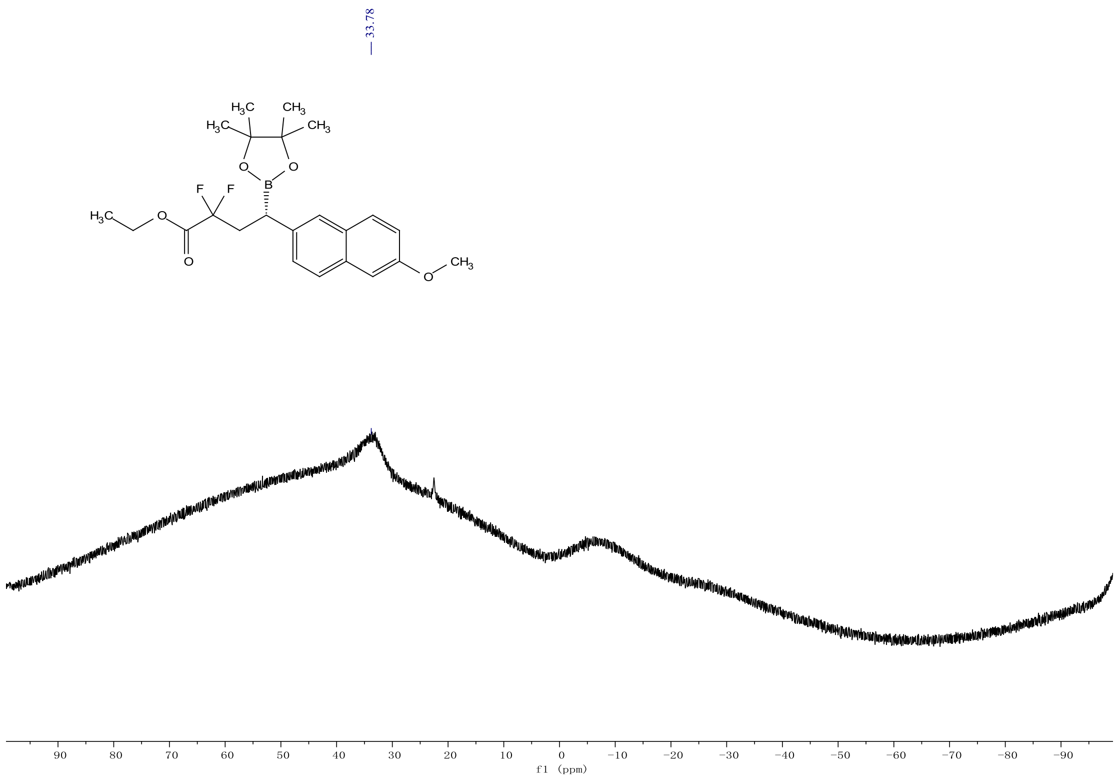

^{11}B-NMR of compound **2q** (128MHz, $CDCl_3$)

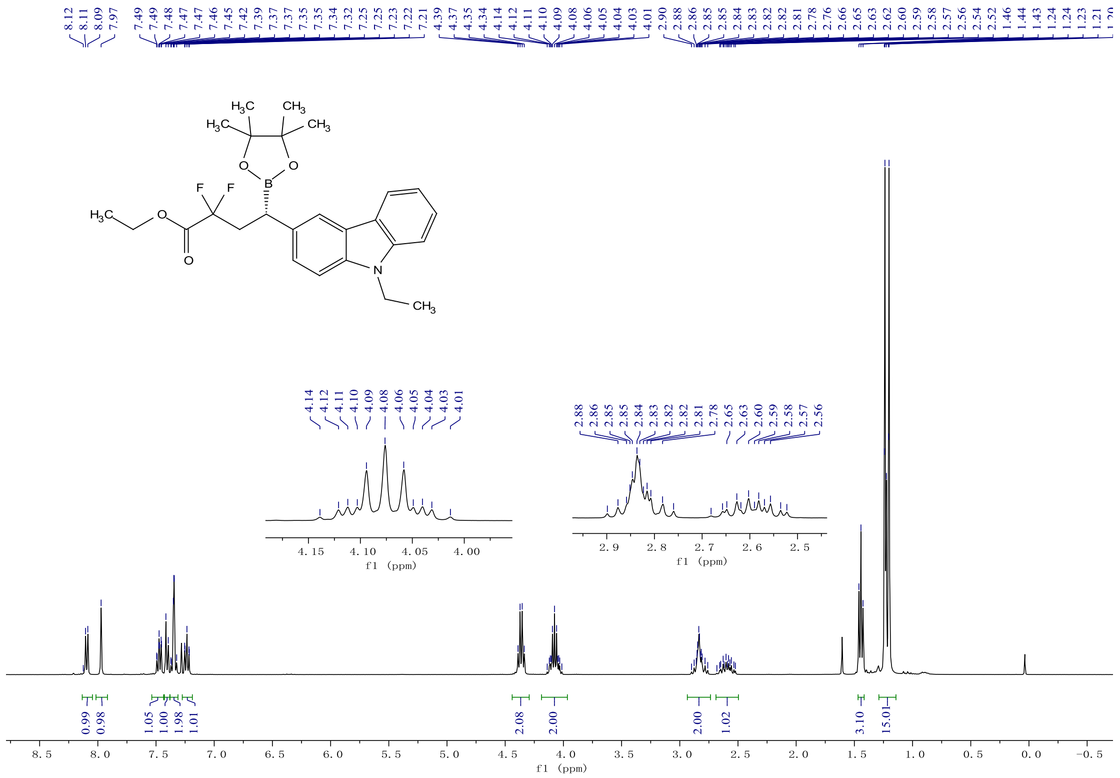

^{1}H-NMR of compound **2r** (400MHz, $CDCl_3$)

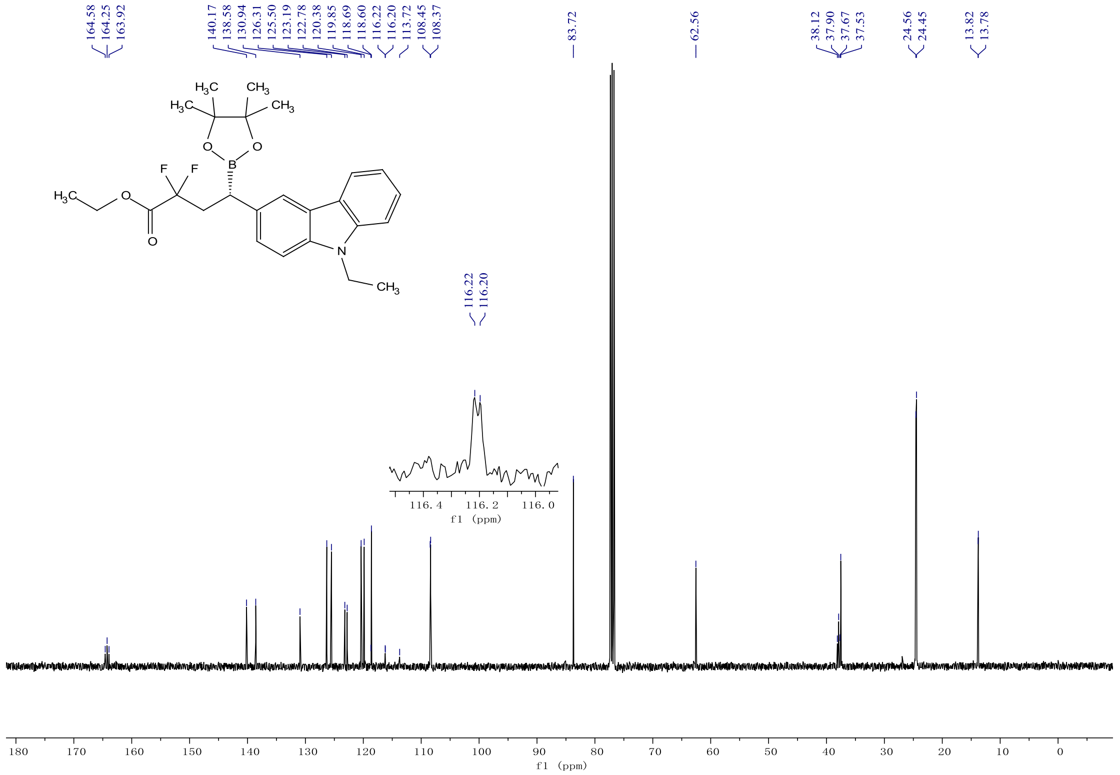

$^{13}C\{^{1}H\}$-NMR of compound **2r** (101MHz, $CDCl_3$)

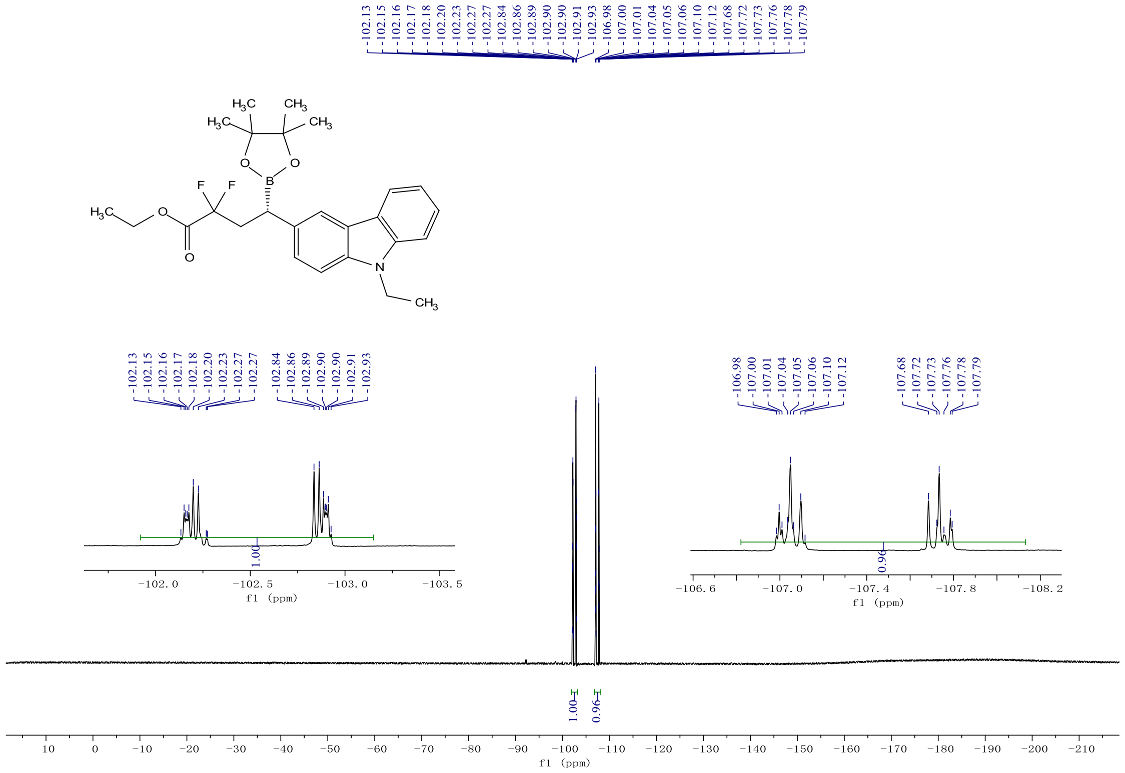

^{19}F-NMR of compound **2r** (377MHz, $CDCl_3$)

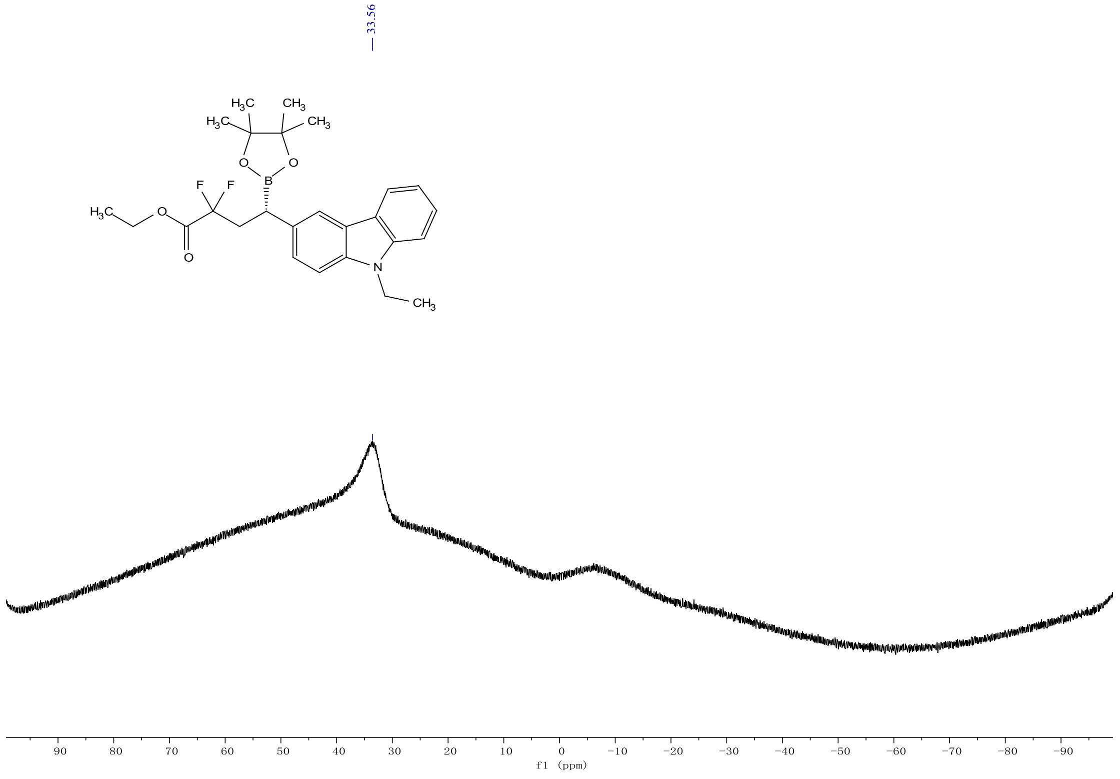

^{11}B-NMR of compound **2r** (128MHz, $CDCl_3$)

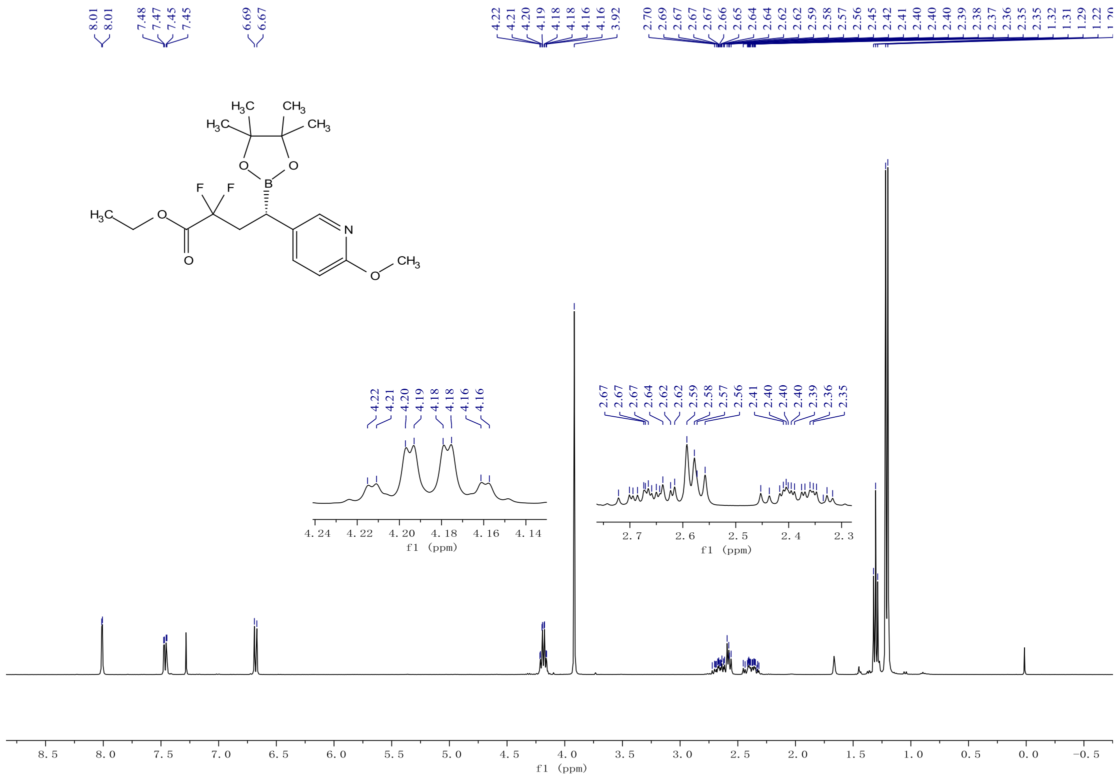

^{1}H-NMR of compound **2s** (400MHz, $CDCl_3$)

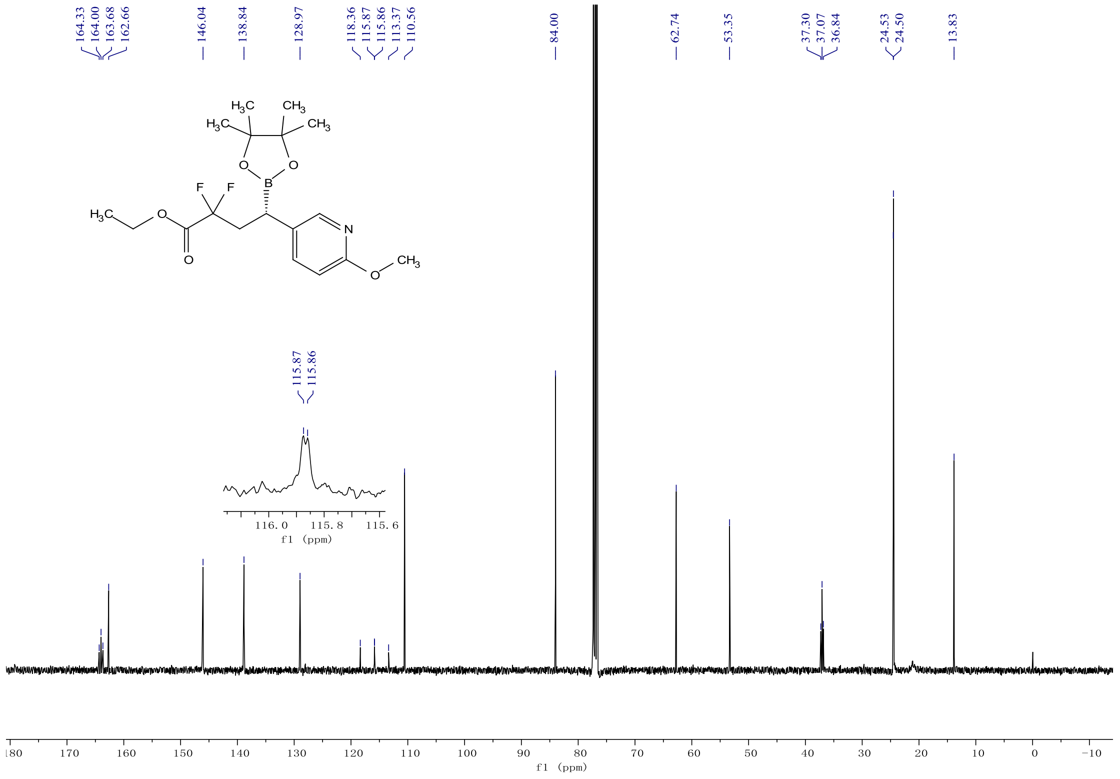

^{13}C{^{1}H}-NMR of compound **2s** (101MHz, $CDCl_3$)

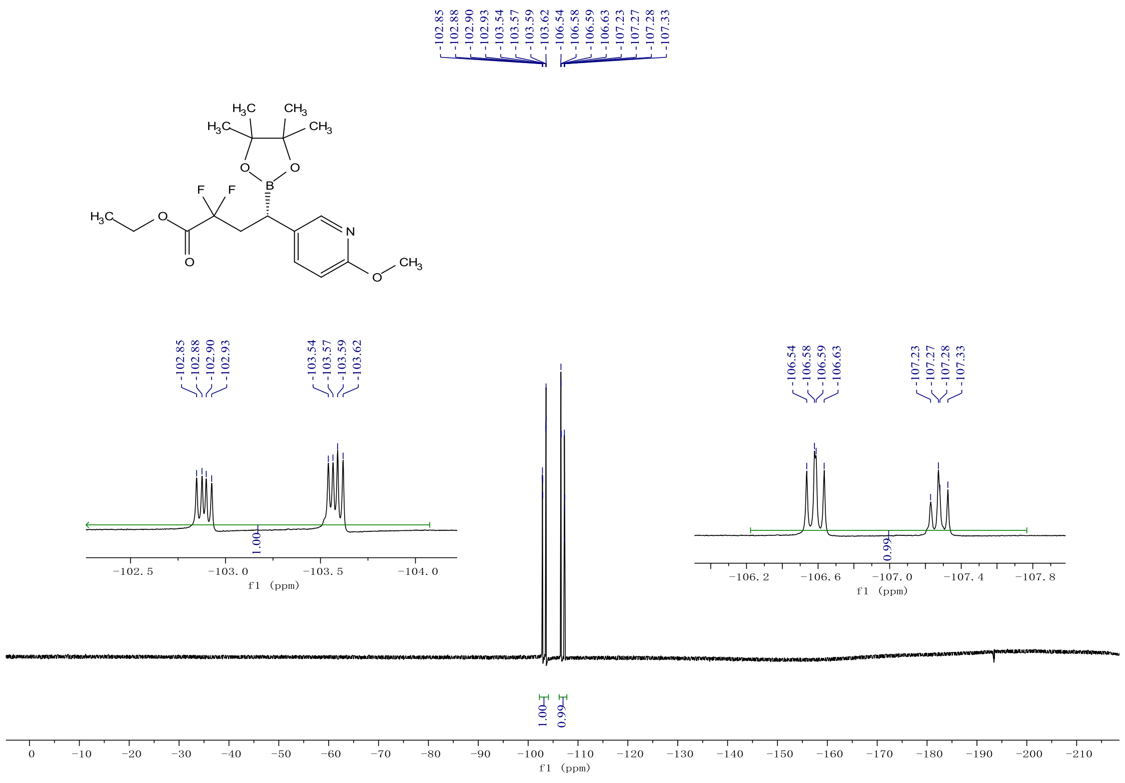

^{19}F-NMR of compound **2s** (377MHz, $CDCl_3$)

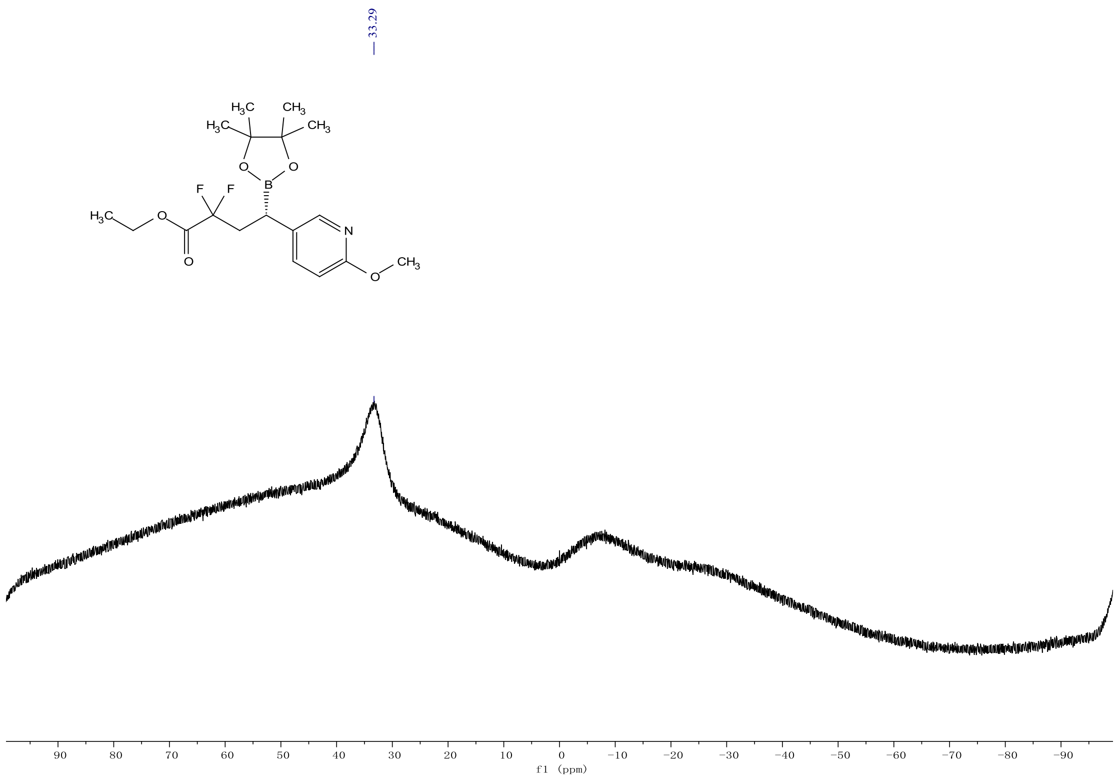

^{11}B-NMR of compound **2s** (128MHz, $CDCl_3$)

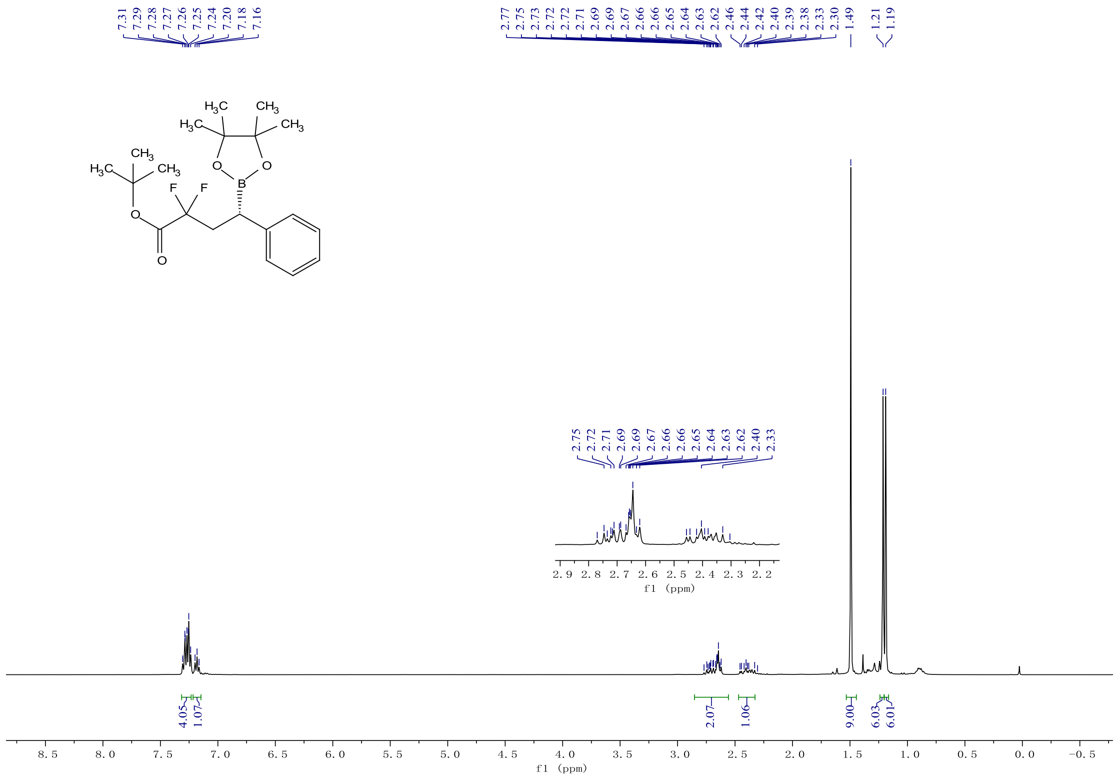

^{1}H-NMR of compound **4a** (400MHz, $CDCl_3$)

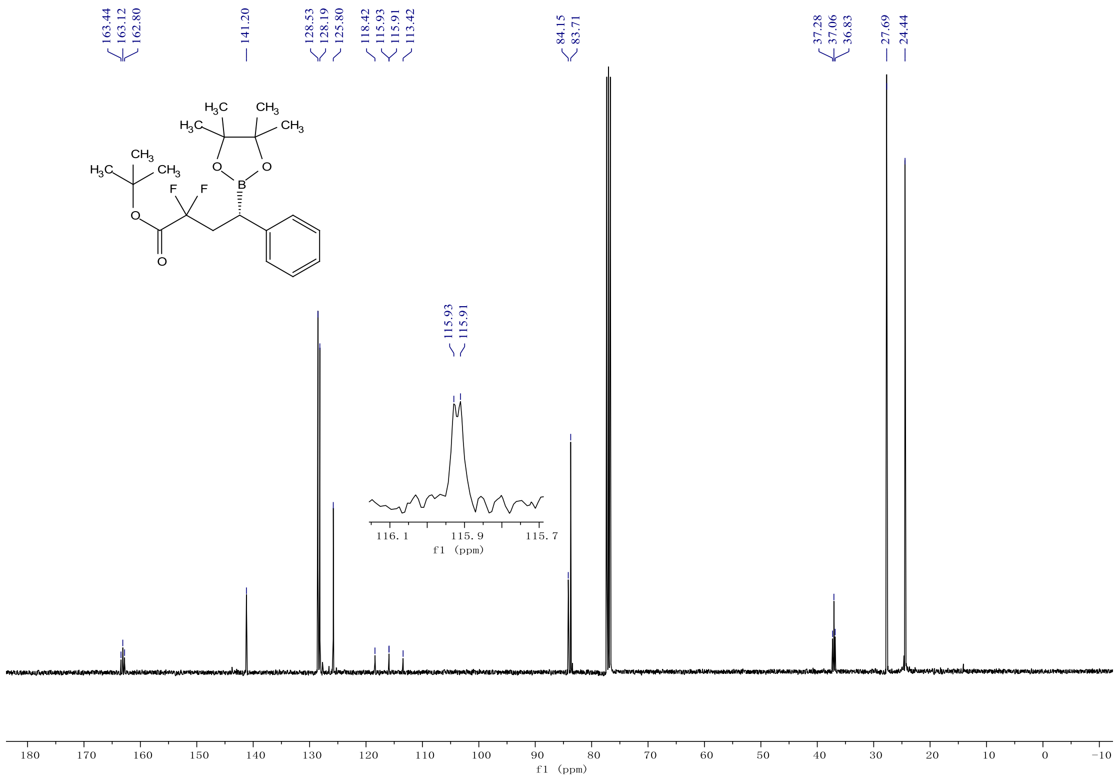

$^{13}C\{^{1}H\}$-NMR of compound **4a** (101MHz, $CDCl_3$)

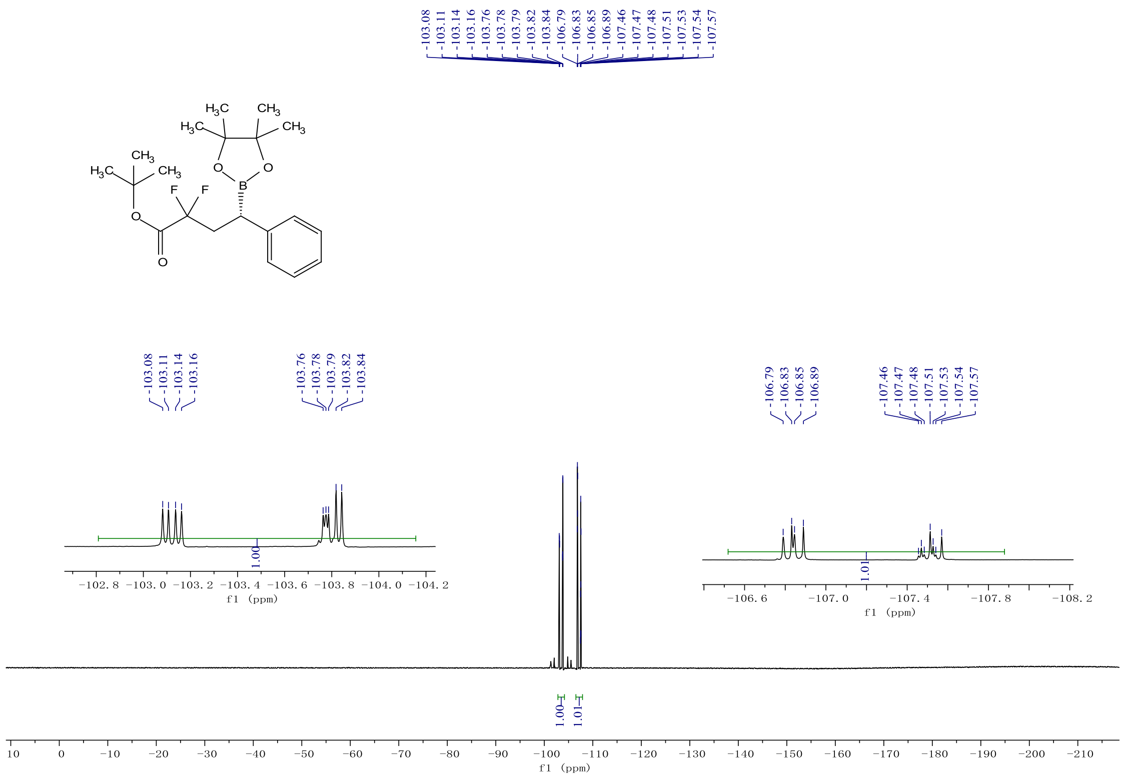

^{19}F-NMR of compound **4a** (377MHz, $CDCl_3$)

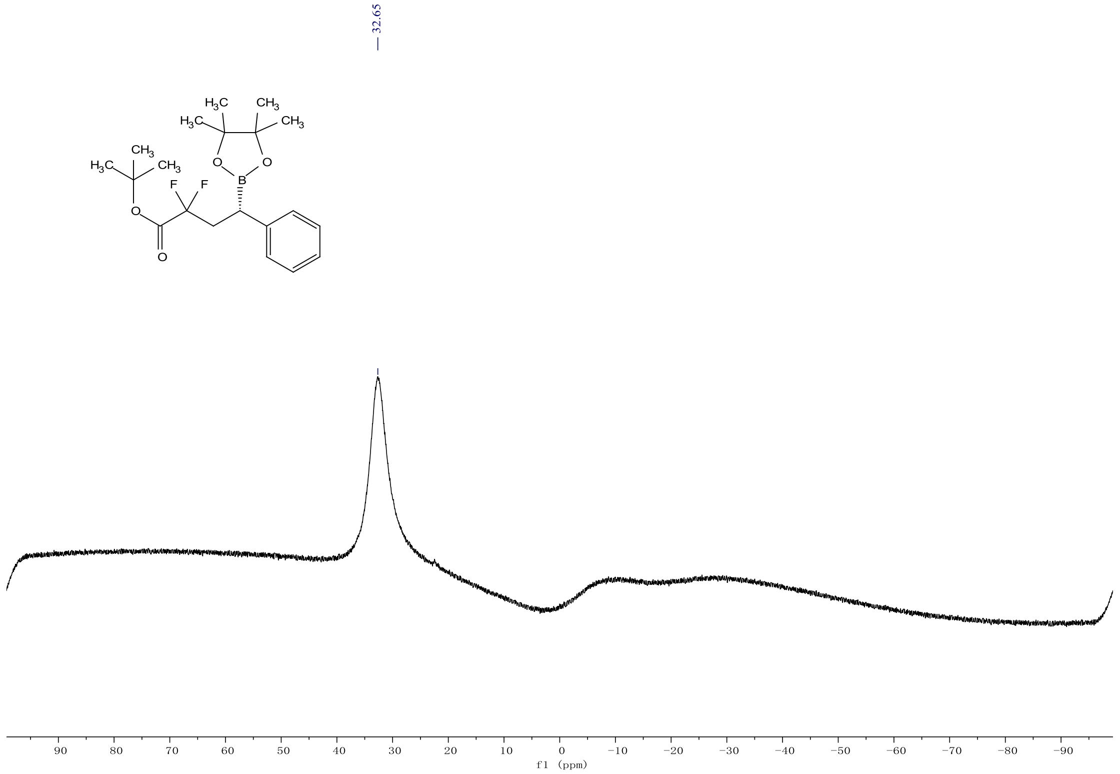

^{11}B-NMR of compound **4a** (128MHz, $CDCl_3$)

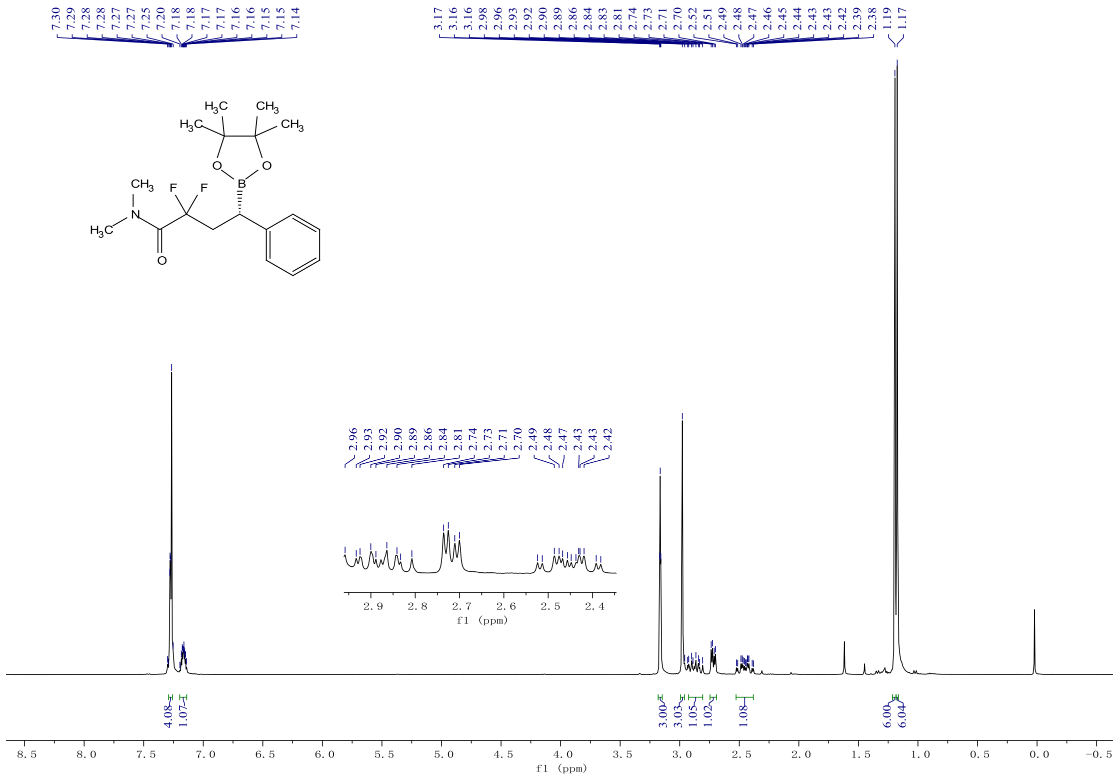

^{1}H-NMR of compound **4b** (400MHz, $CDCl_3$)

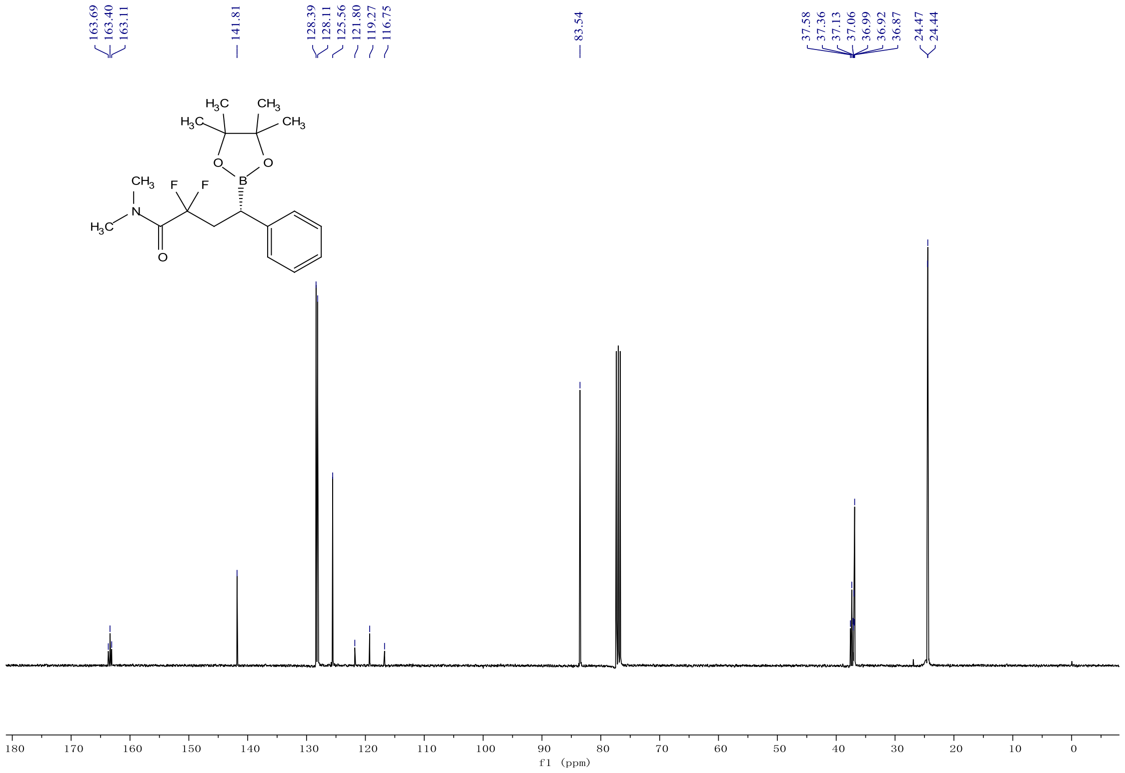

$^{13}C\{^{1}H\}$-NMR of compound **4b** (101MHz, $CDCl_3$)

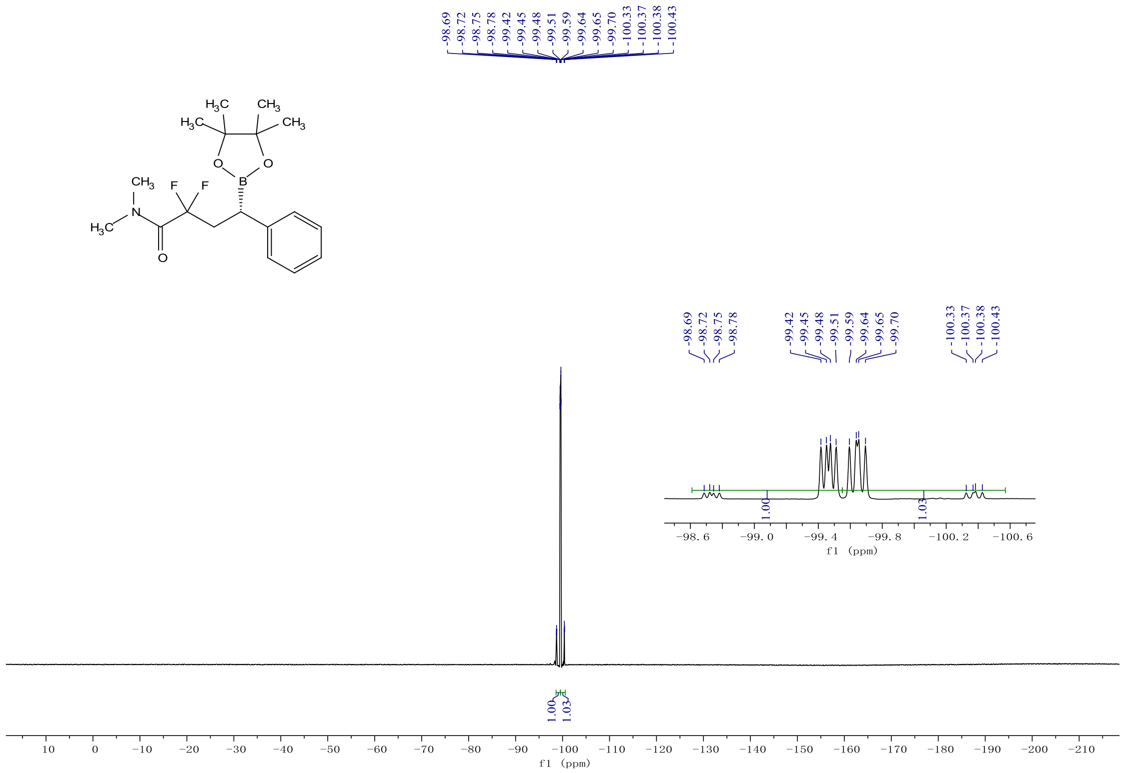

^{19}F-NMR of compound **4b** (377MHz, $CDCl_3$)

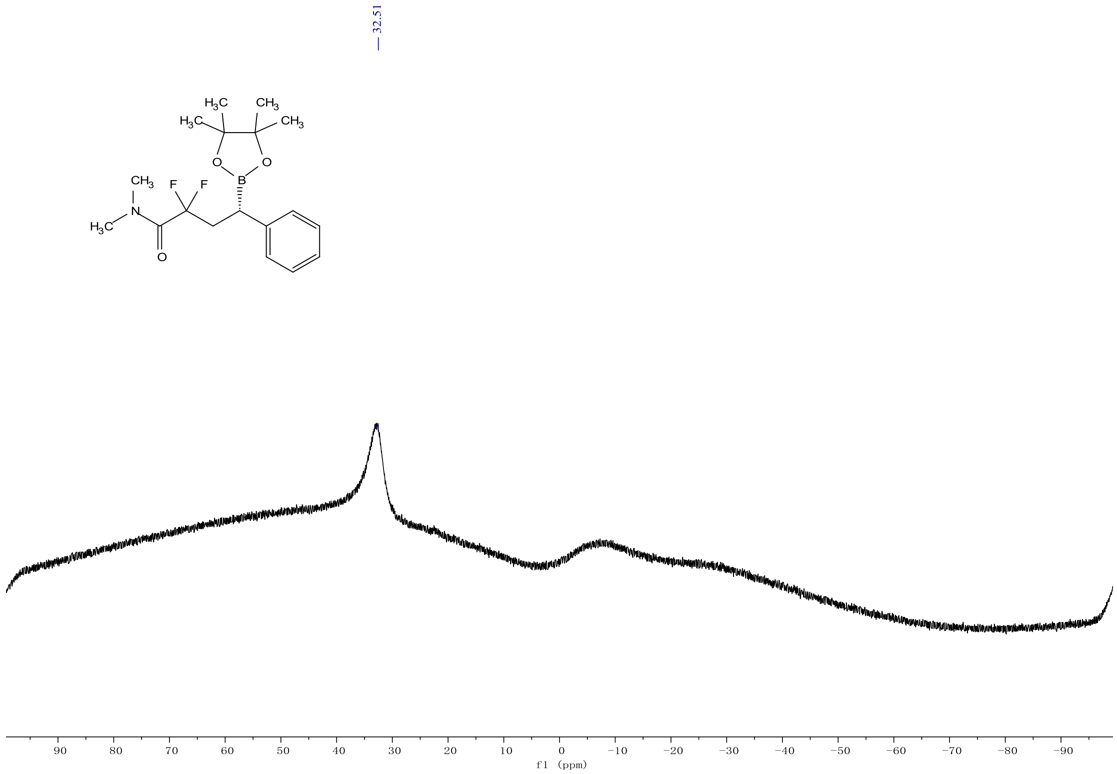

^{11}B-NMR of compound **4b** (128MHz, $CDCl_3$)

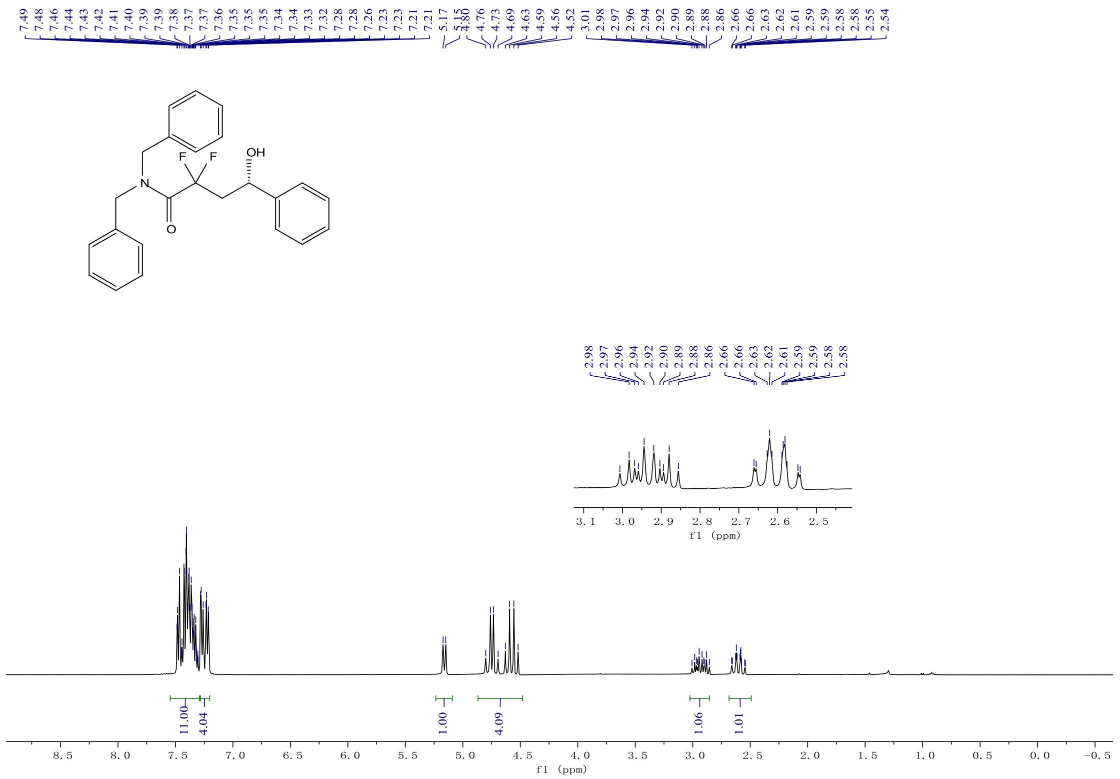

^{1}H-NMR of compound **4c** (400MHz, $CDCl_3$)

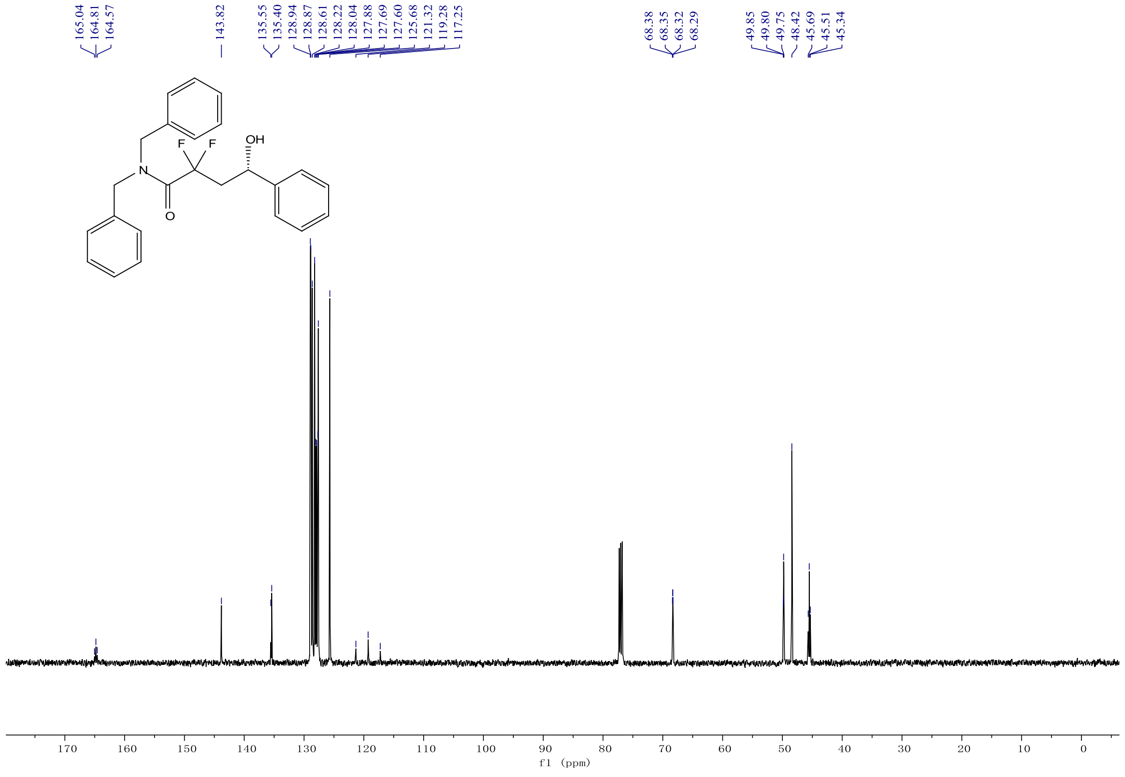

^{13}C{^{1}H}-NMR of compound **4c** (101MHz, $CDCl_3$)

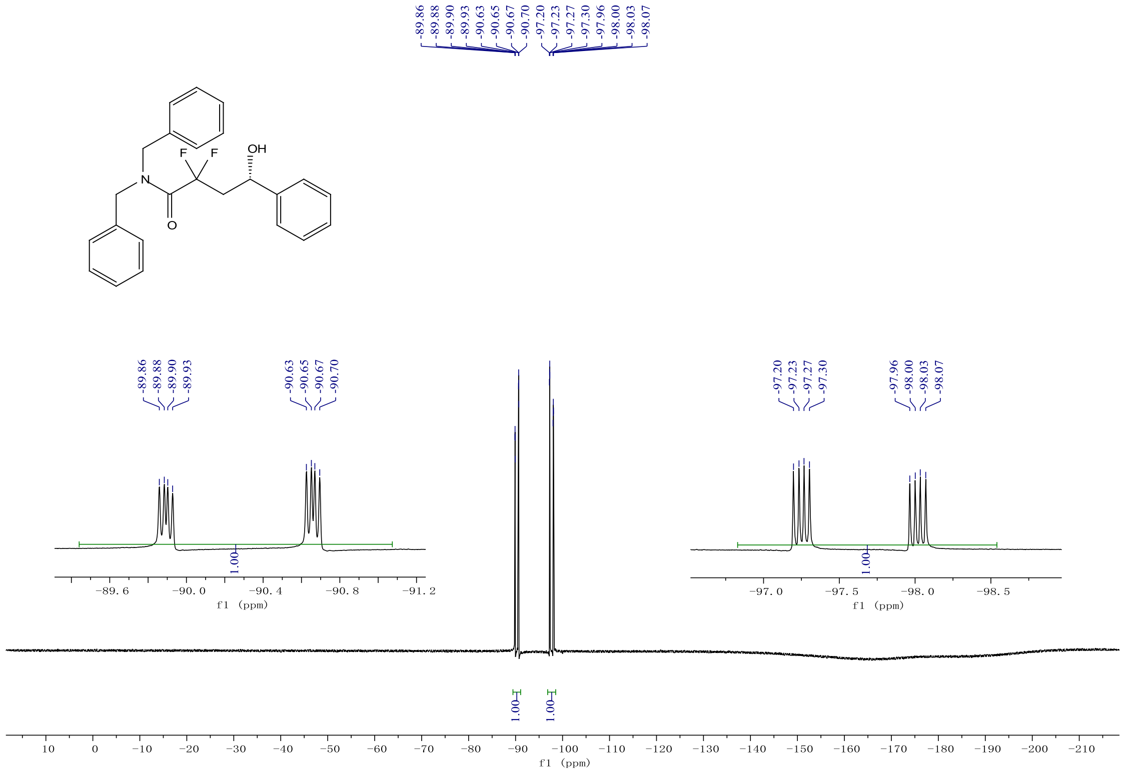

^{19}F-NMR of compound **4c** (377MHz, $CDCl_3$)

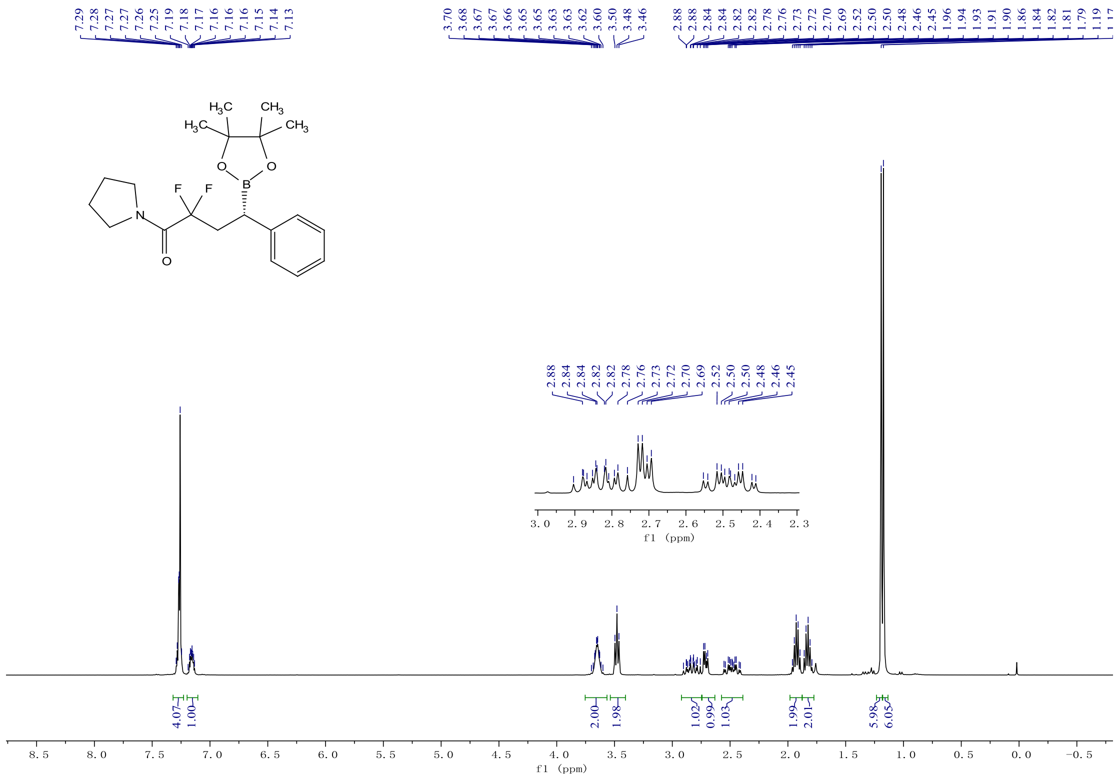

^{1}H-NMR of compound **4d** (400MHz, $CDCl_3$)

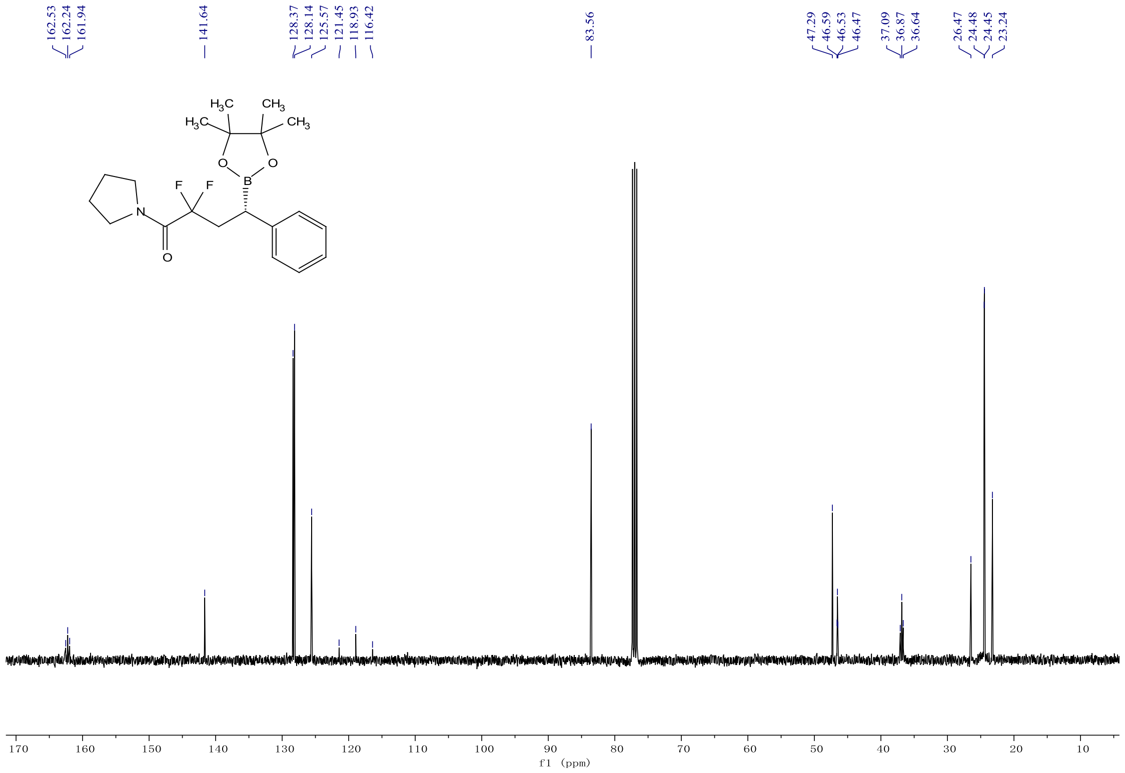

$^{13}C\{^{1}H\}$-NMR of compound **4d** (101MHz, $CDCl_3$)

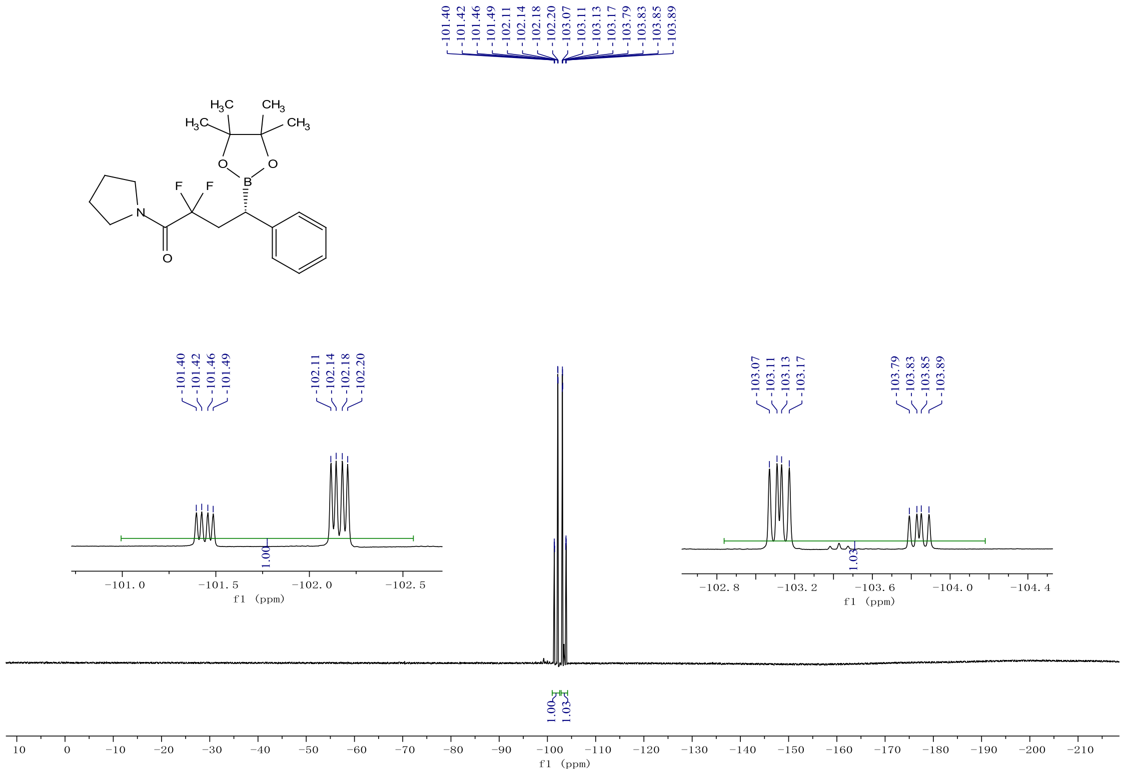

^{19}F-NMR of compound **4d** (377MHz, $CDCl_3$)

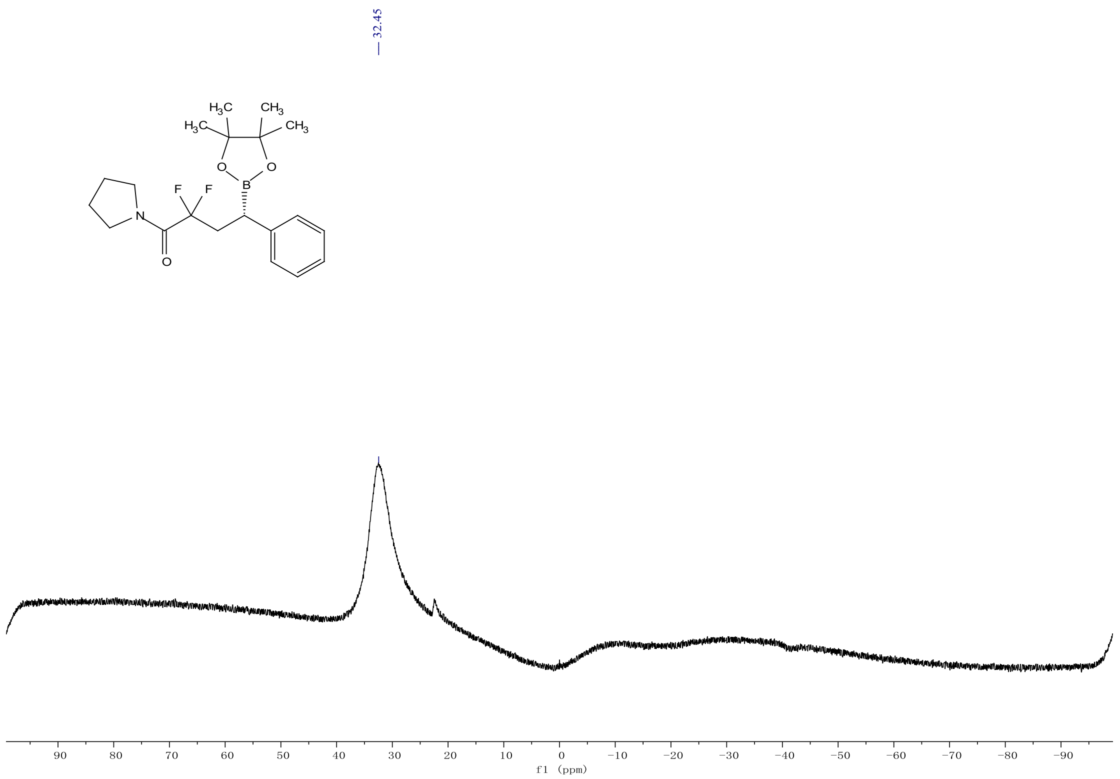

^{11}B-NMR of compound **4d** (128MHz, $CDCl_3$)

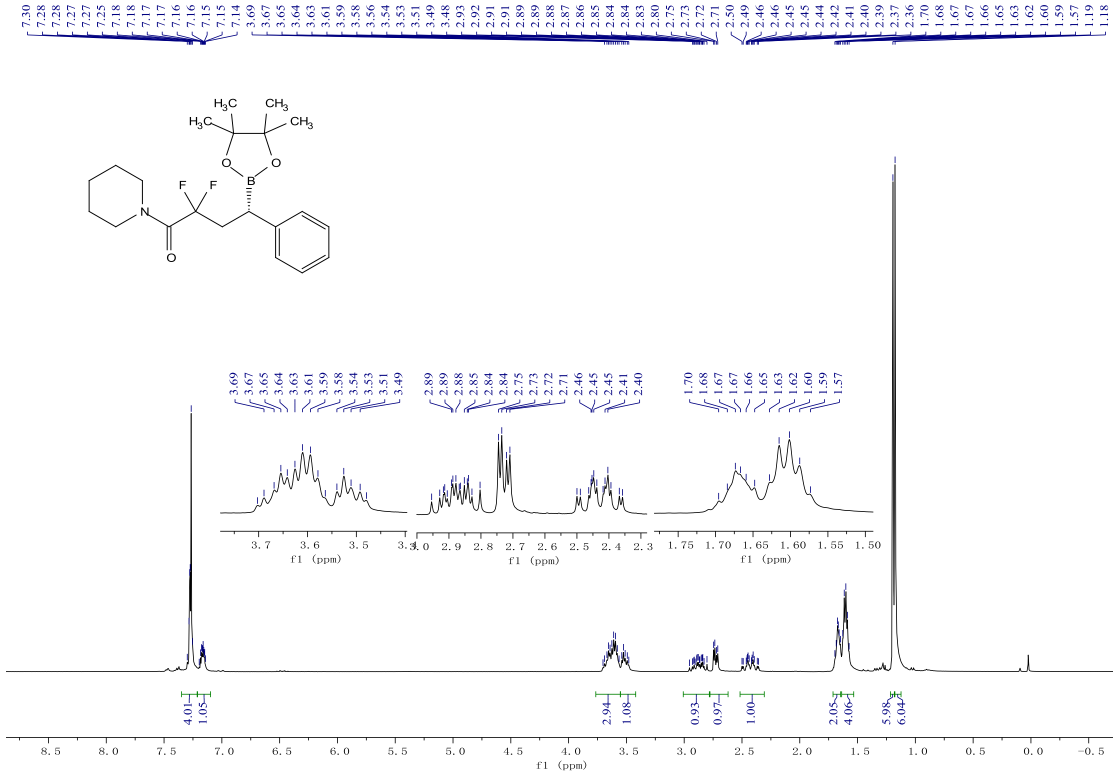

^{1}H-NMR of compound **4e** (400MHz, $CDCl_3$)

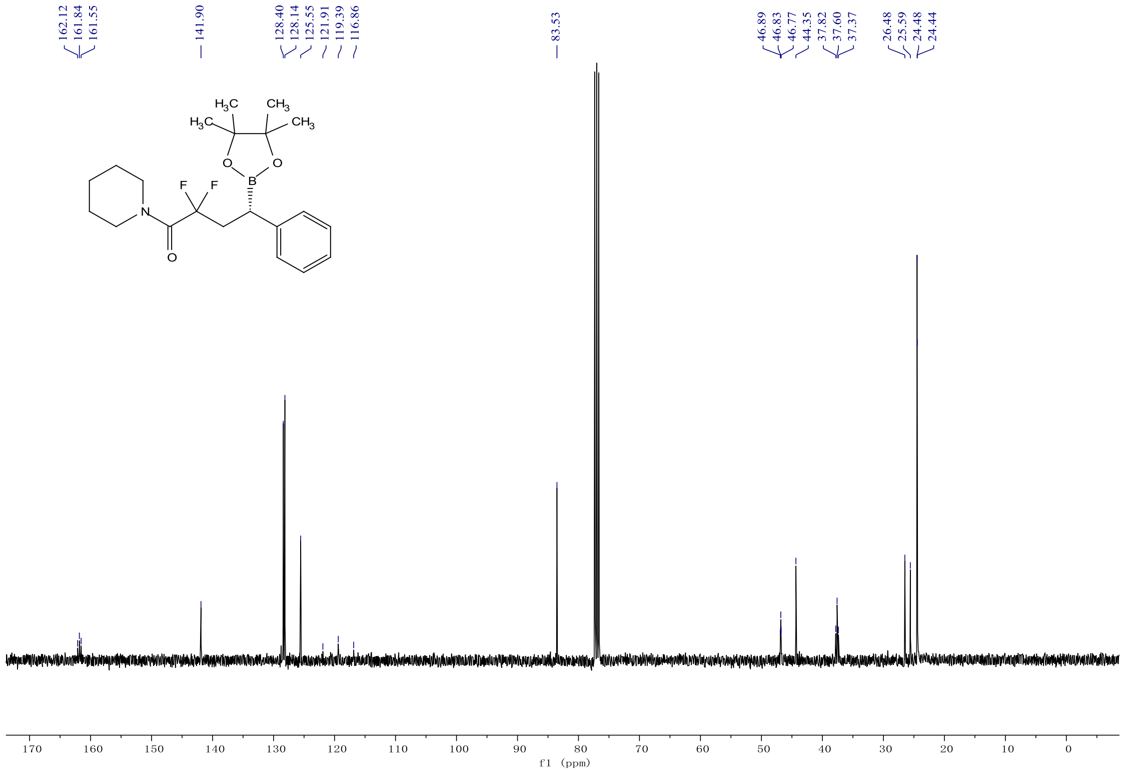

$^{13}C\{^{1}H\}$-NMR of compound **4e** (101MHz, $CDCl_3$)

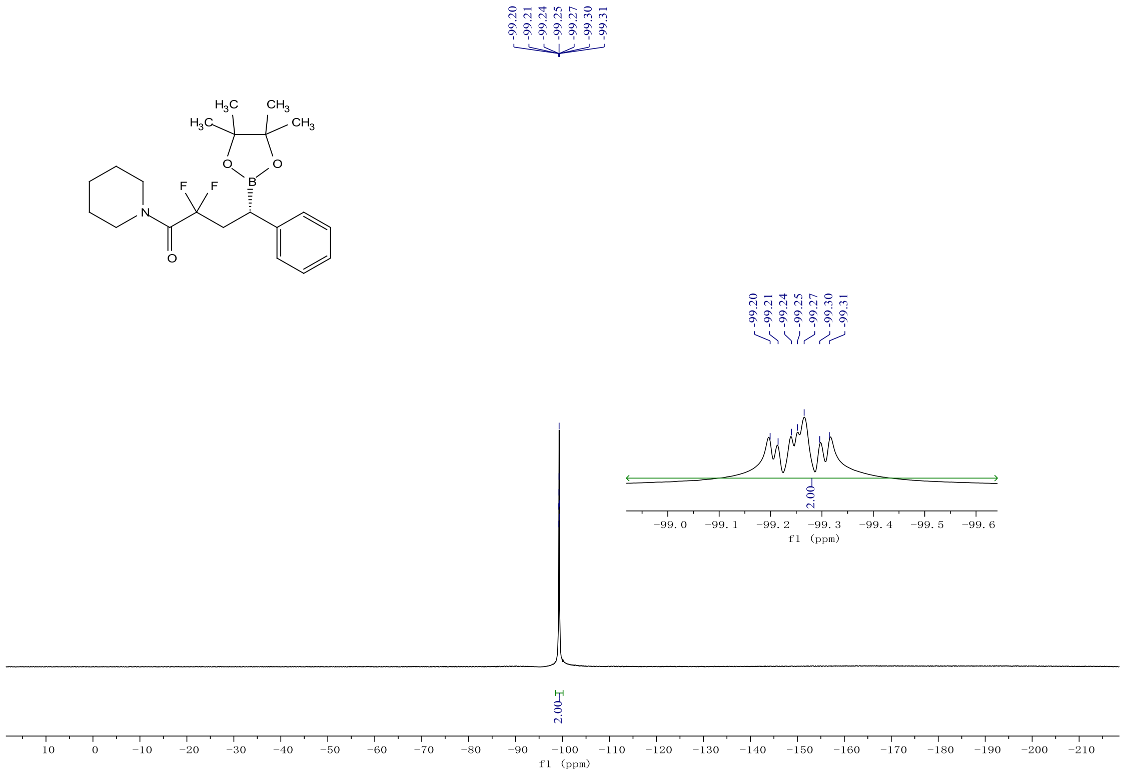

^{19}F-NMR of compound **4e** (377MHz, $CDCl_3$)

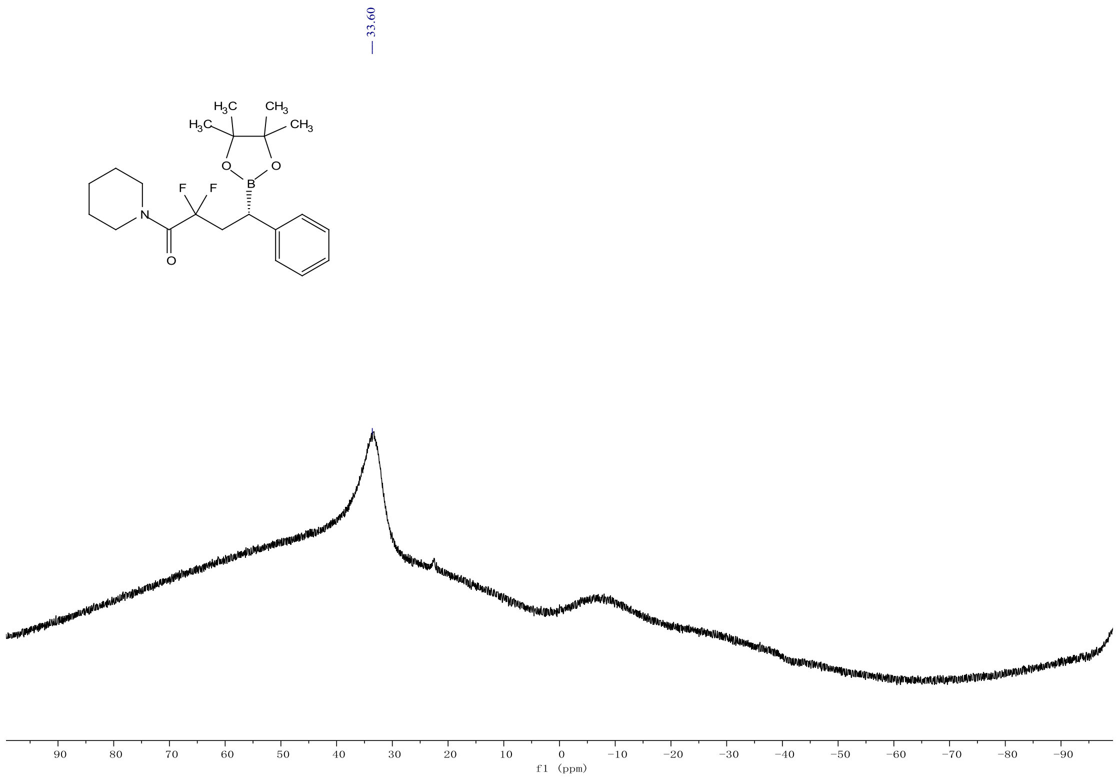

^{11}B-NMR of compound **4e** (128MHz, $CDCl_3$)

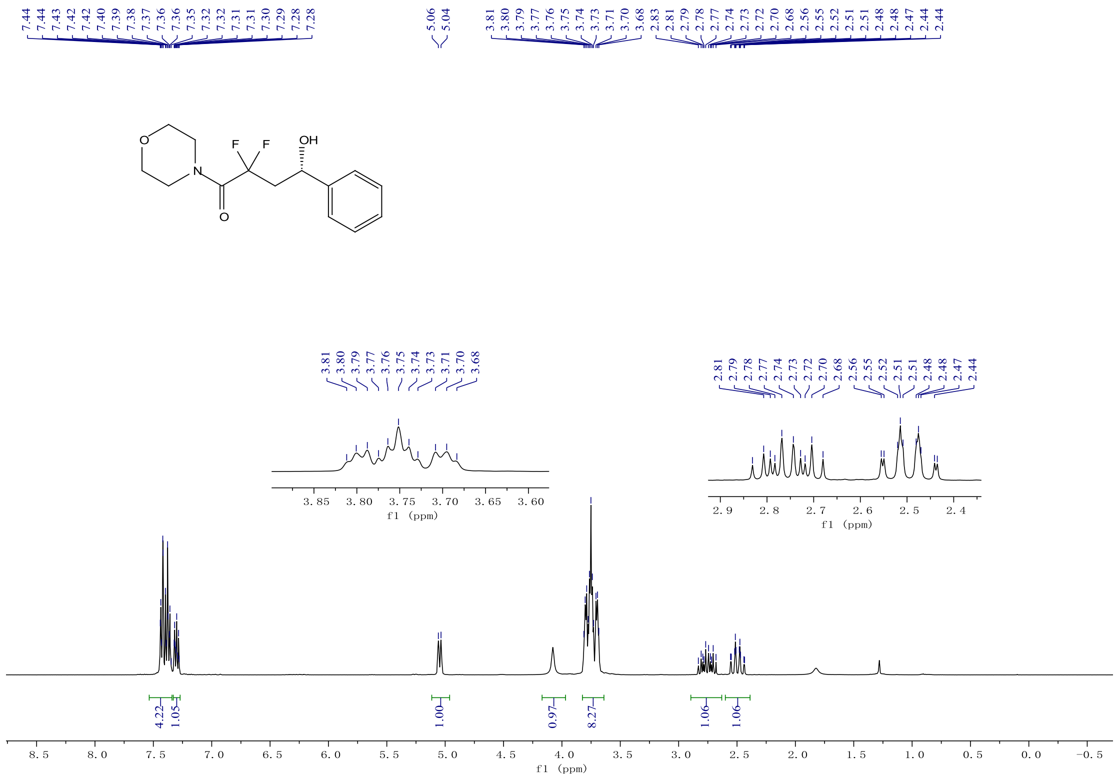

^{1}H-NMR of compound **4f** (400MHz, $CDCl_3$)

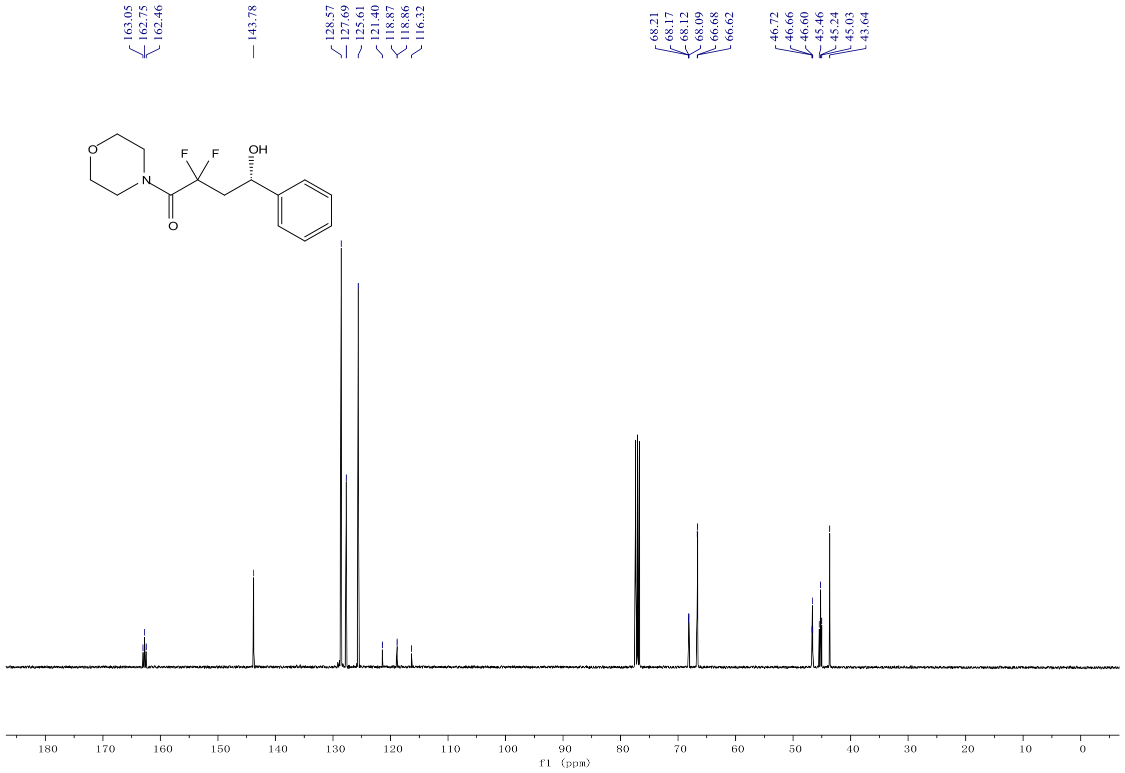

^{13}C{^{1}H}-NMR of compound **4f** (101MHz, $CDCl_3$)

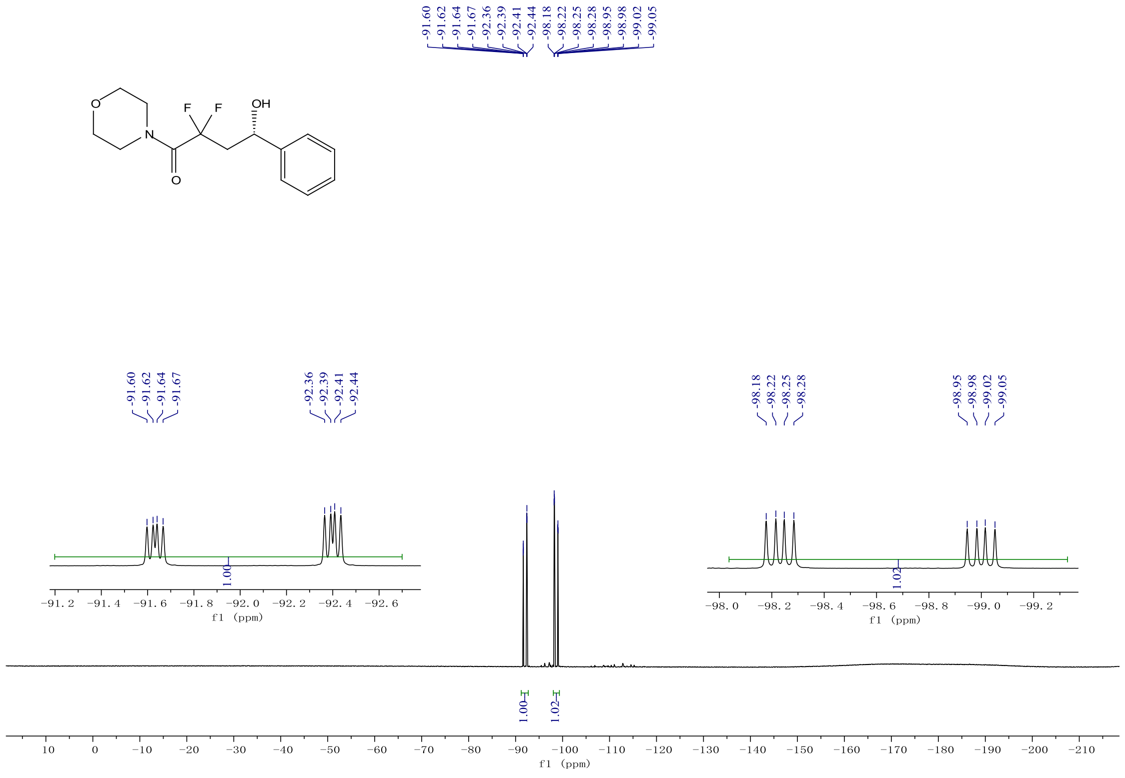

^{19}F-NMR of compound **4f** (377MHz, $CDCl_3$)

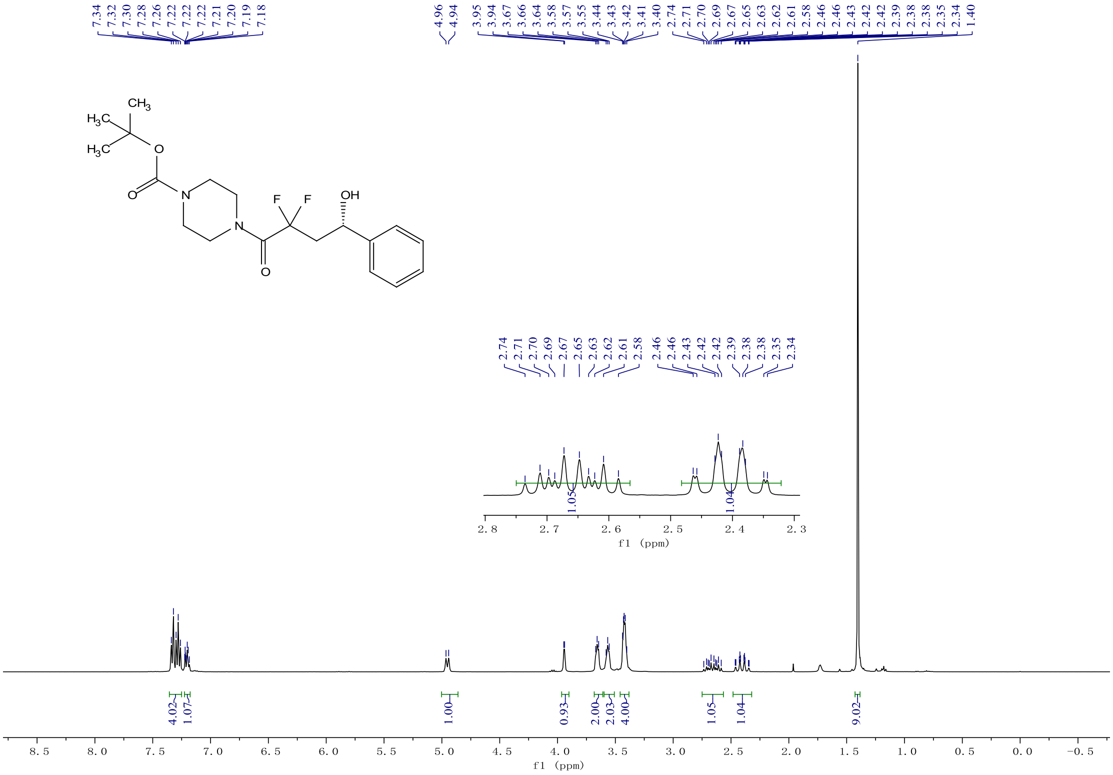

^{1}H-NMR of compound **4g** (400MHz, $CDCl_3$)

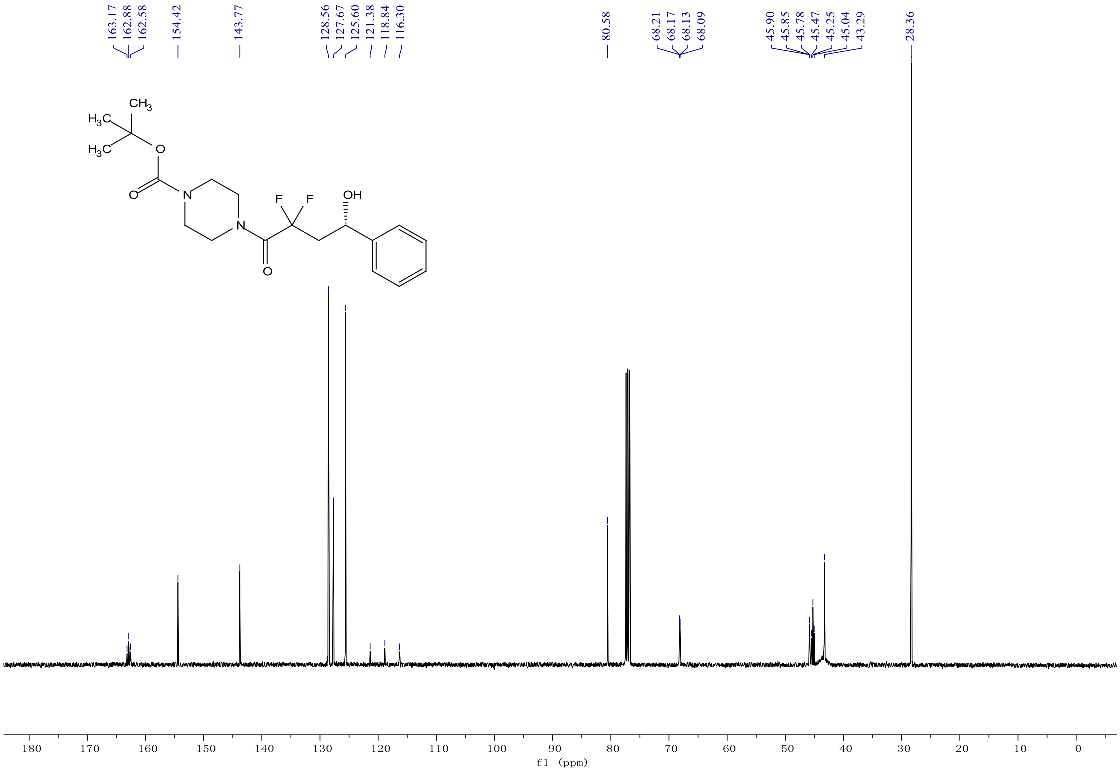

^{13}C{^{1}H}-NMR of compound **4g** (101MHz, $CDCl_3$)

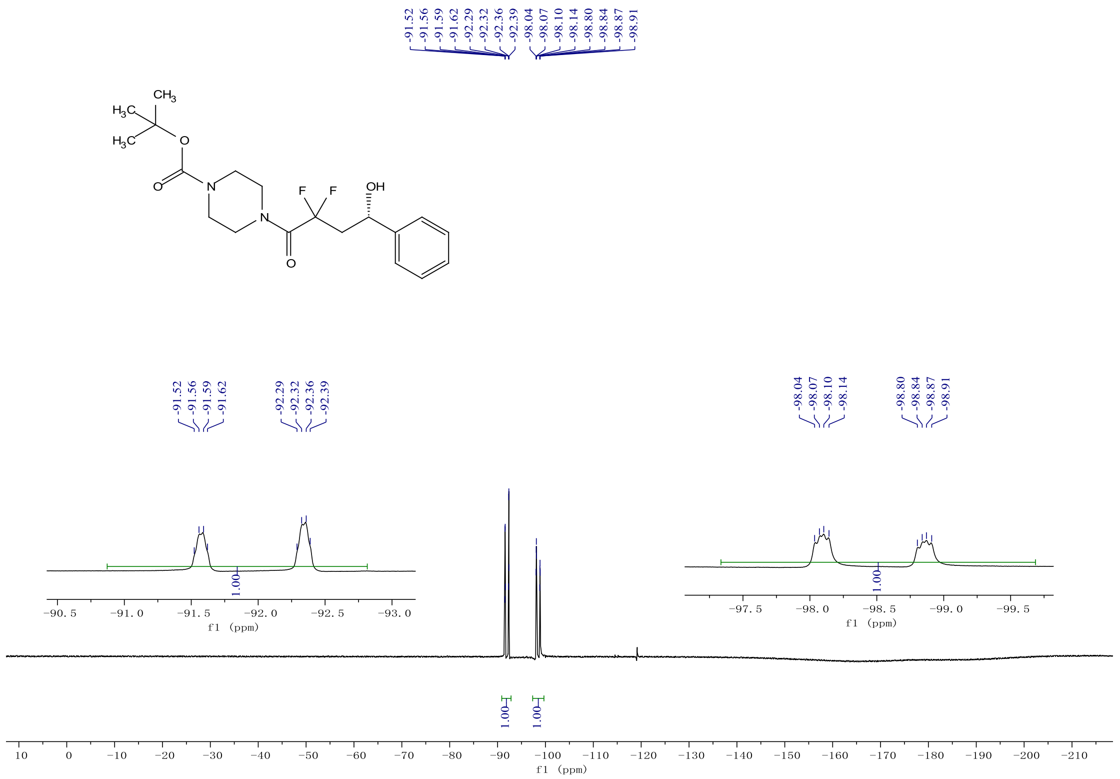

^{19}F-NMR of compound **4g** (377MHz, $CDCl_3$)

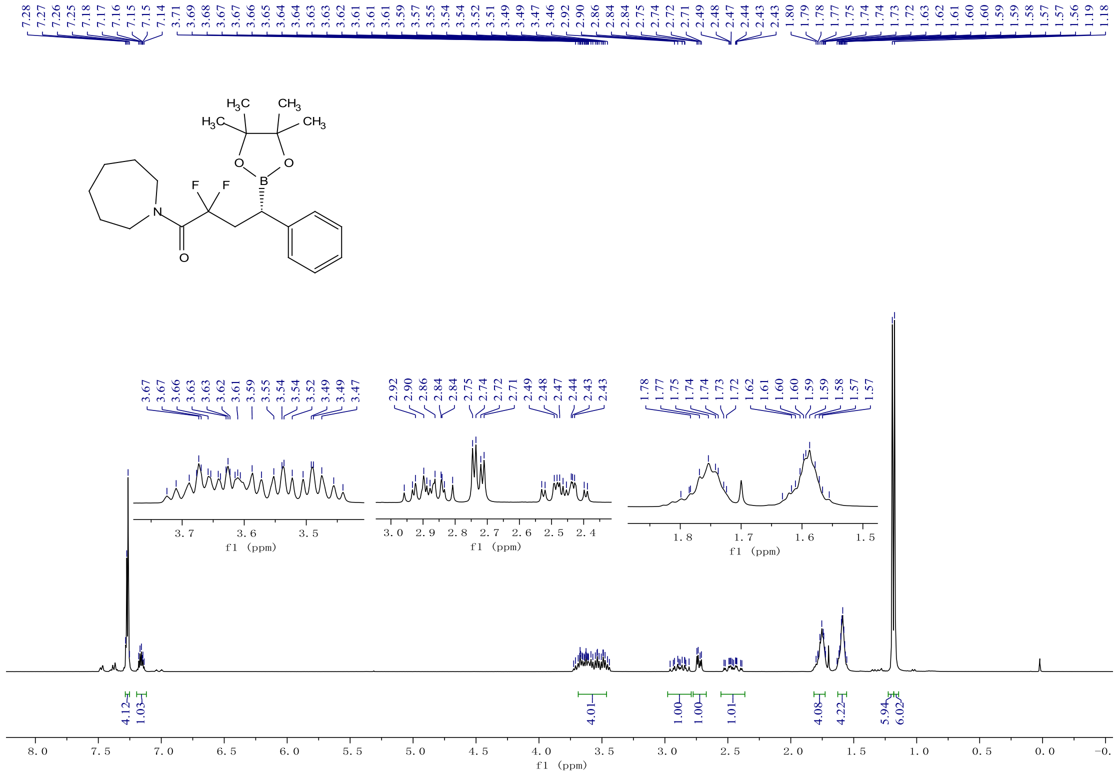

^{1}H-NMR of compound **4h** (400MHz, $CDCl_3$)

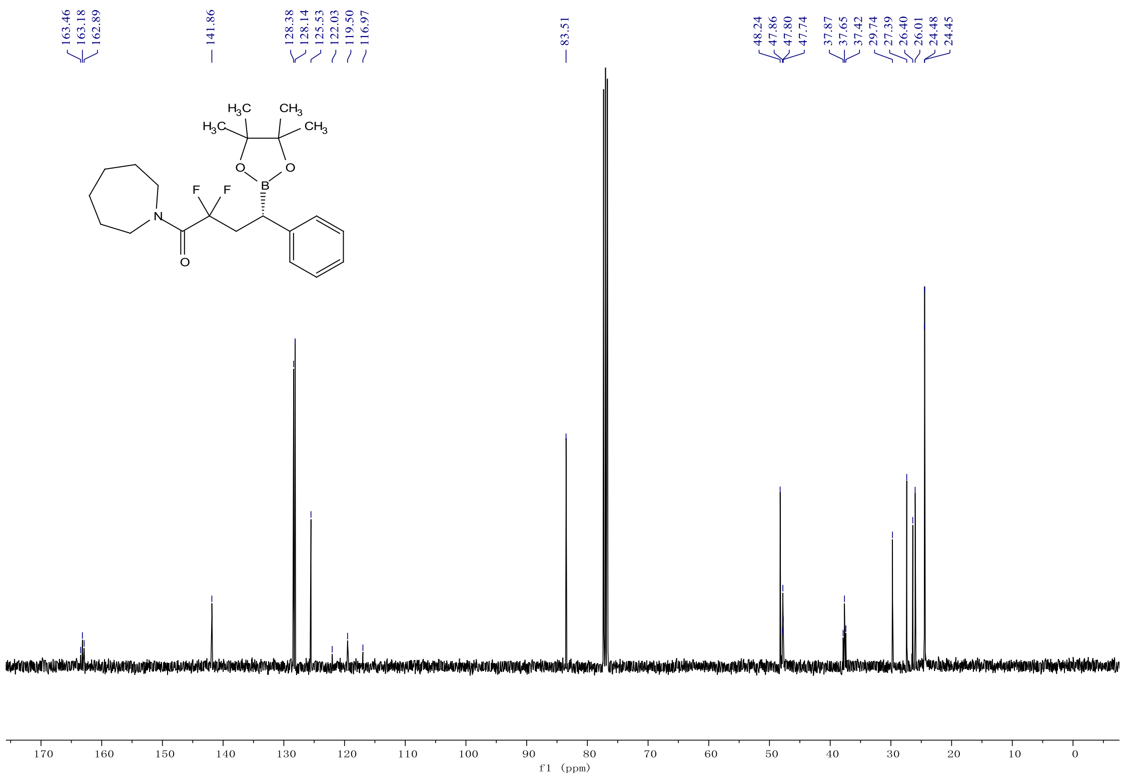

$^{13}C\{^{1}H\}$-NMR of compound **4h** (101MHz, $CDCl_3$)

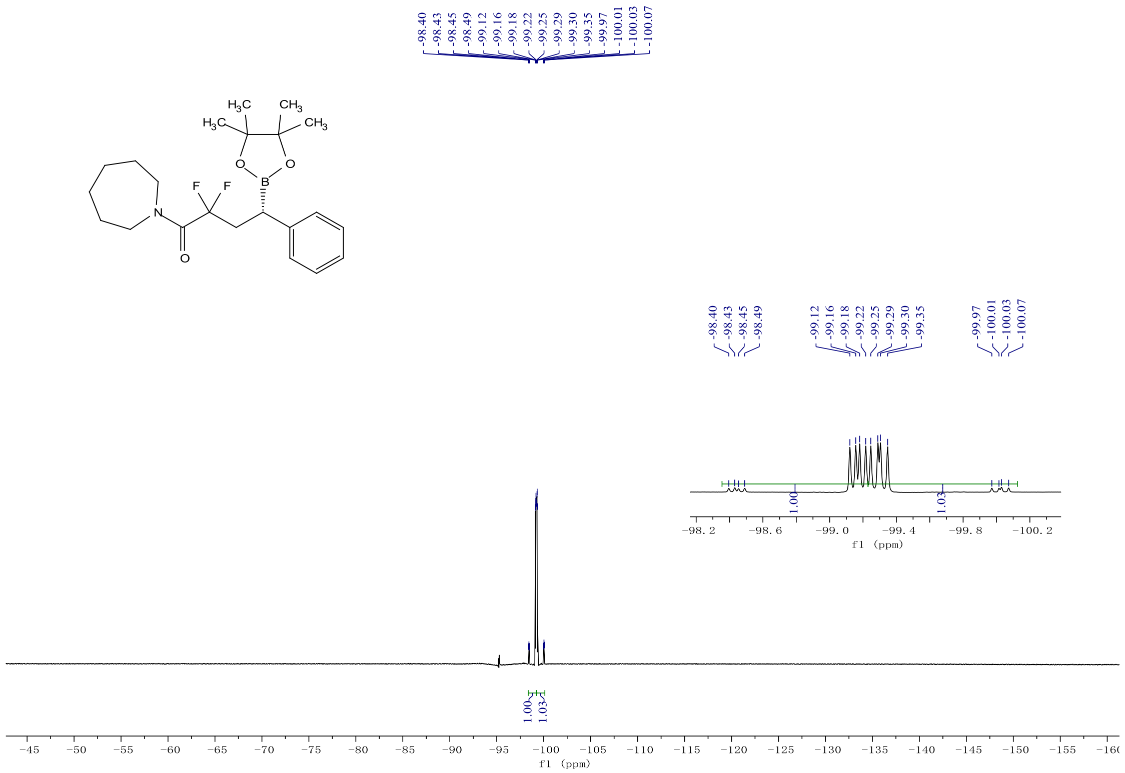

^{19}F-NMR of compound **4h** (377MHz, $CDCl_3$)

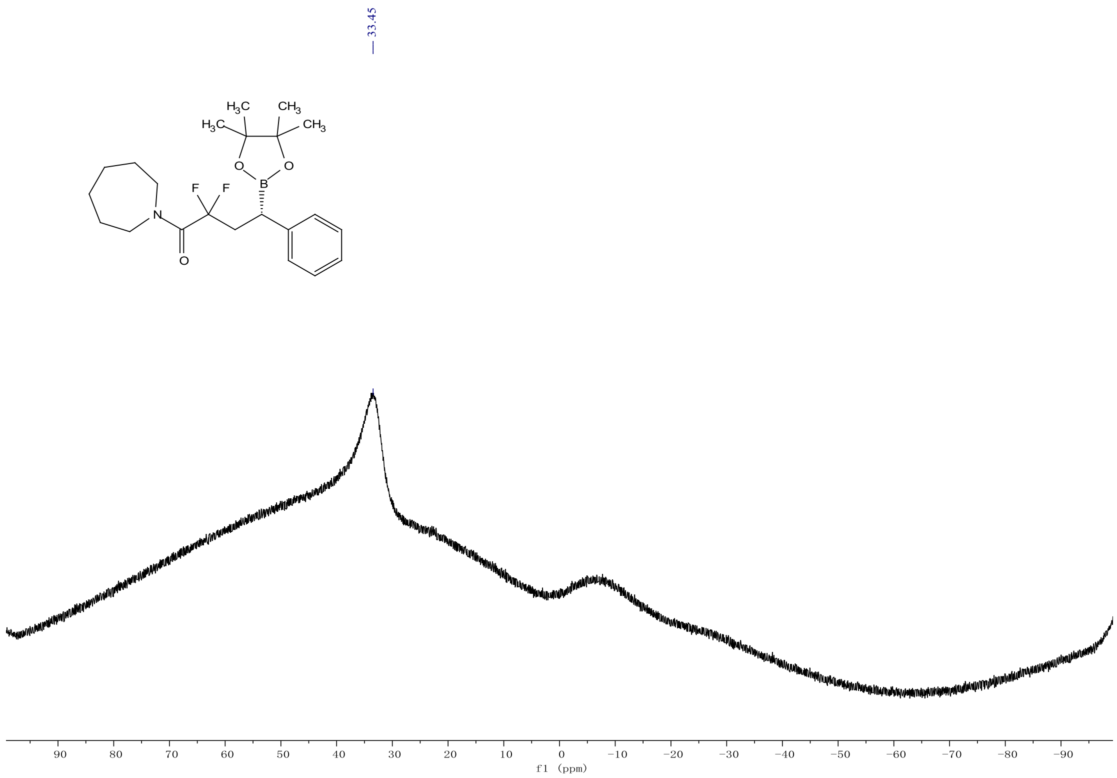

^{11}B-NMR of compound **4h** (128MHz, $CDCl_3$)

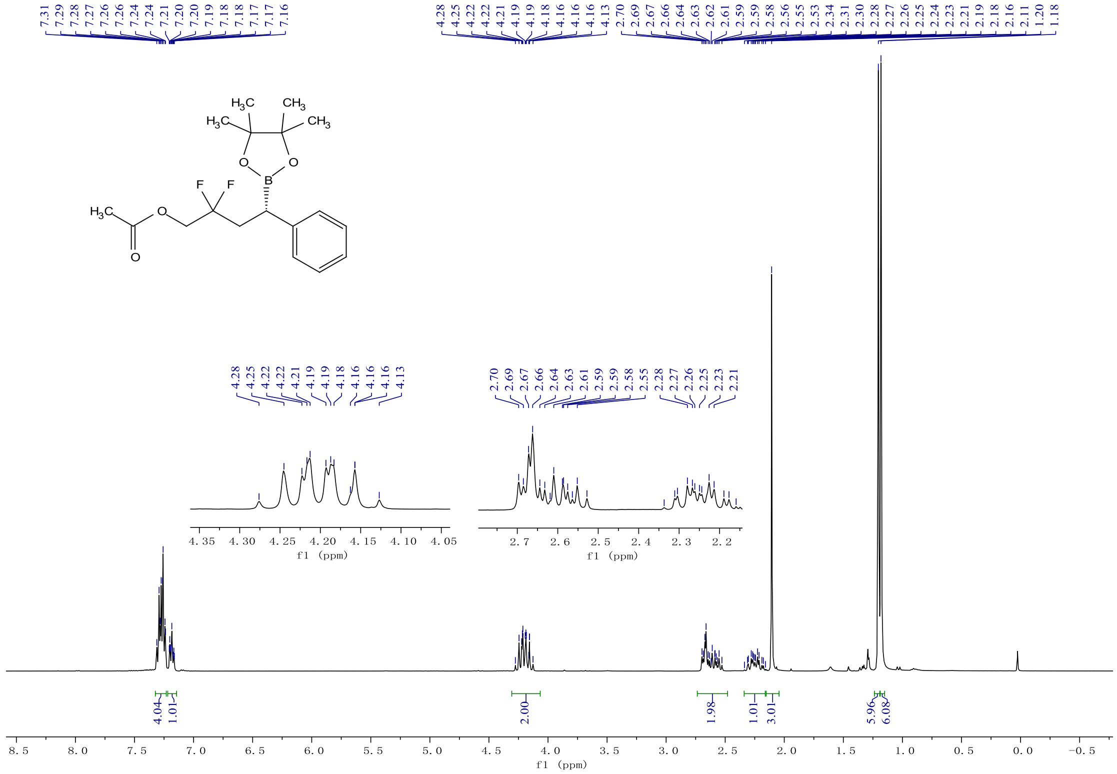

^{1}H-NMR of compound **4i** (400MHz, $CDCl_3$)

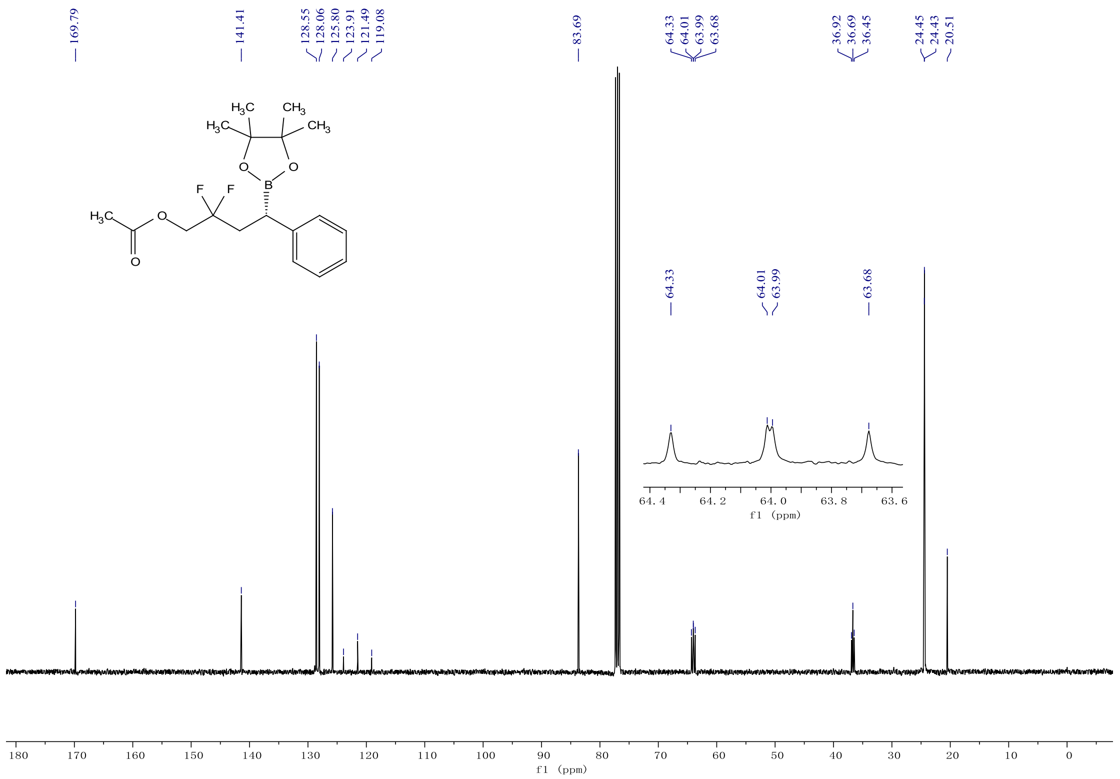

$^{13}C\{^{1}H\}$-NMR of compound **4i** (101MHz, $CDCl_3$)

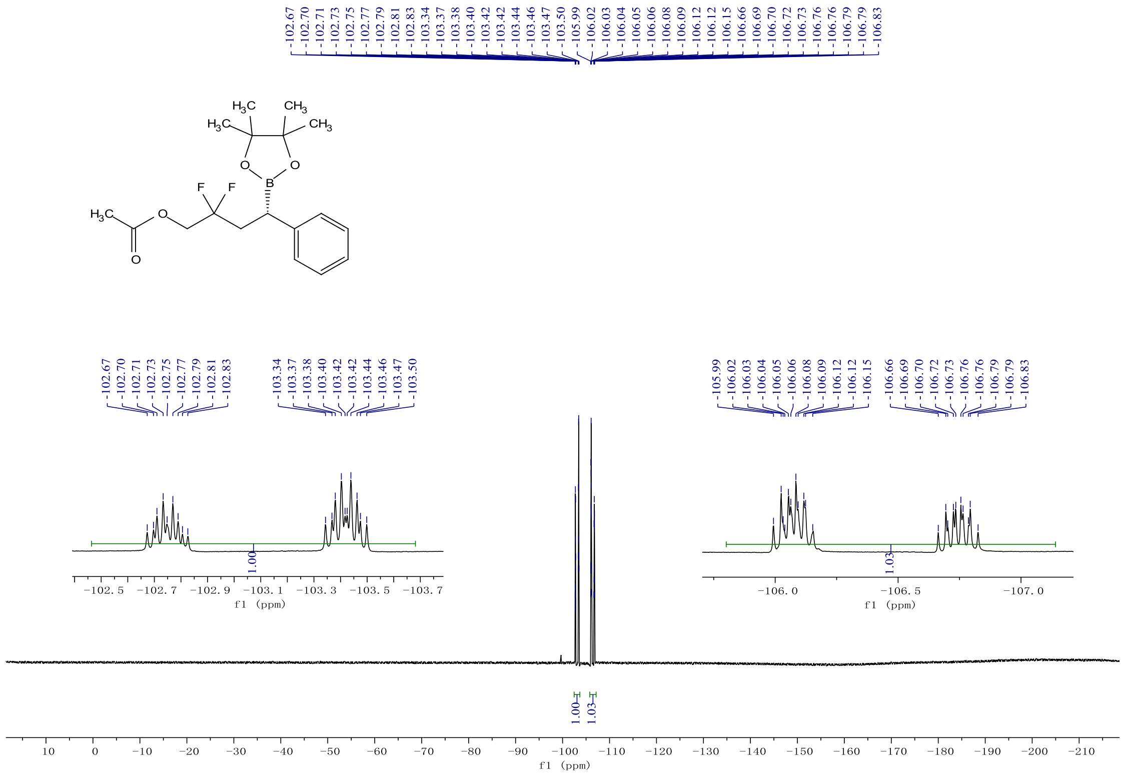

^{19}F-NMR of compound **4i** (377MHz, $CDCl_3$)

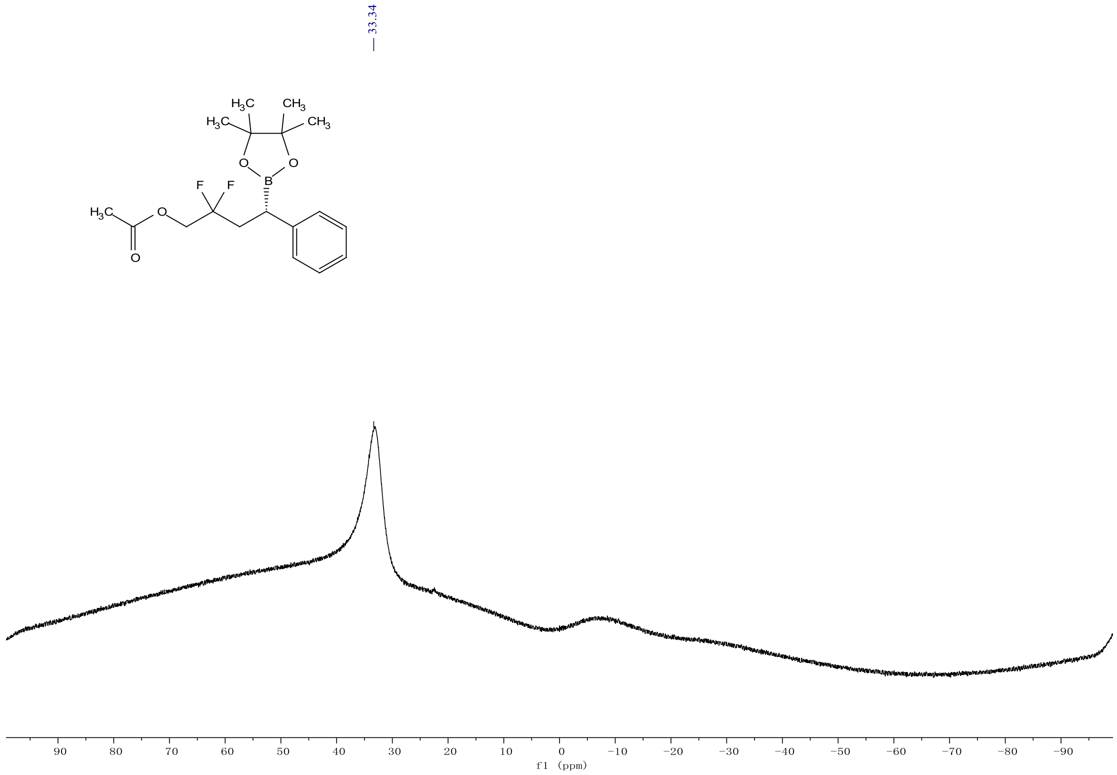

^{11}B-NMR of compound **4i** (128MHz, $CDCl_3$)

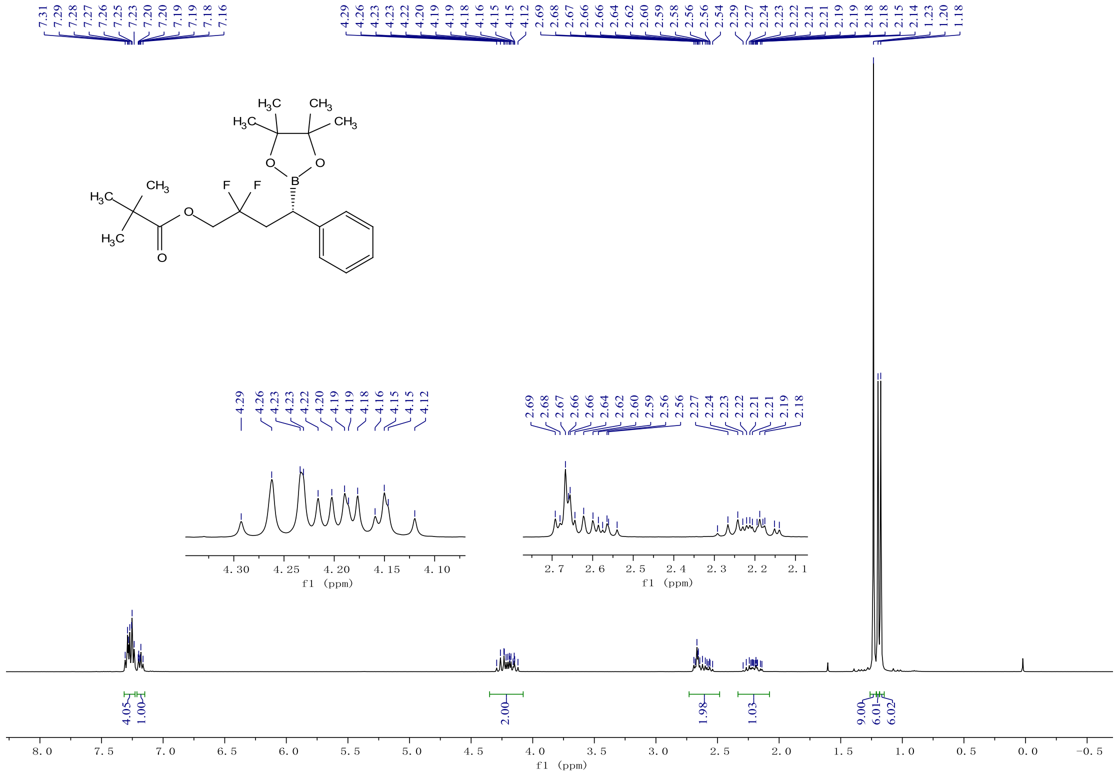

^{1}H-NMR of compound **4j** (400MHz, $CDCl_3$)

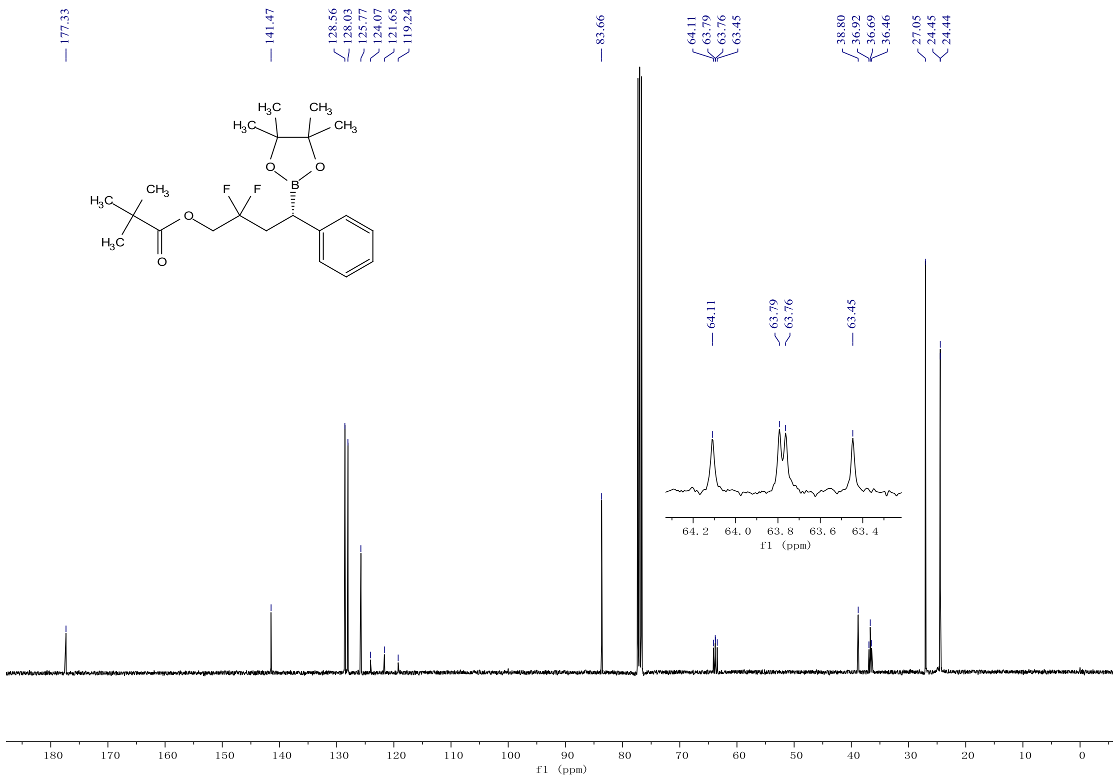

$^{13}C\{^{1}H\}$-NMR of compound **4j** (101MHz, $CDCl_3$)

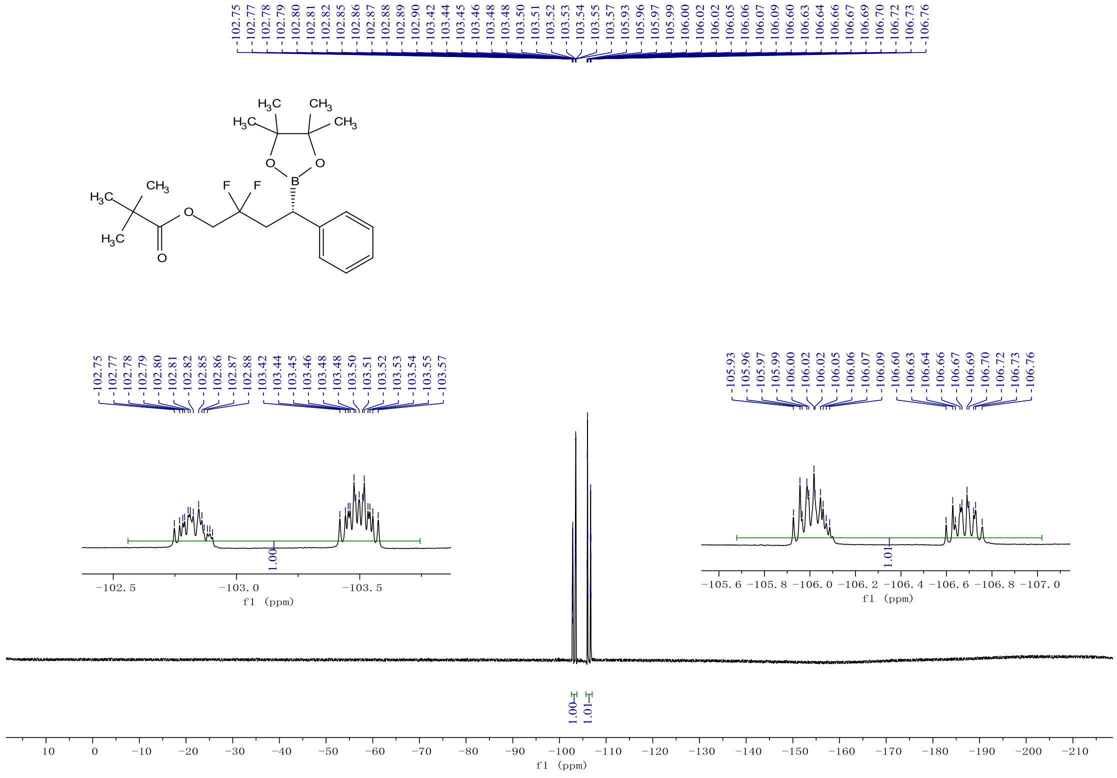

^{19}F-NMR of compound **4j** (377MHz, $CDCl_3$)

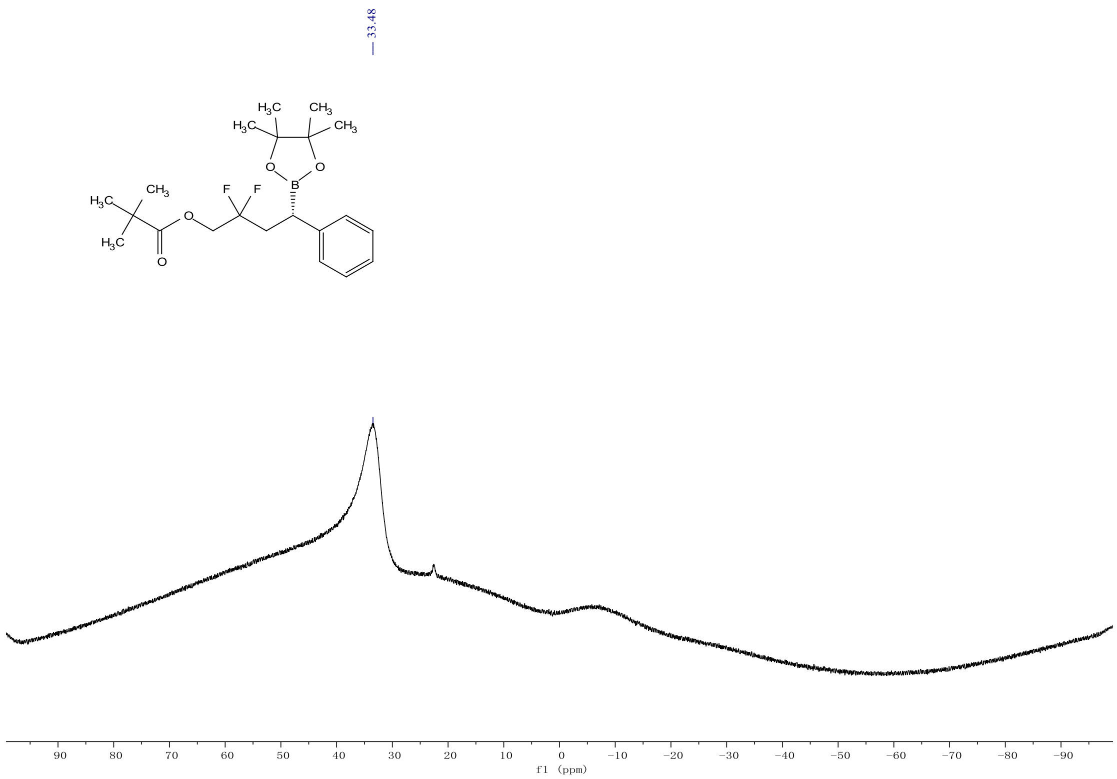

^{11}B-NMR of compound **4j** (128MHz, $CDCl_3$)

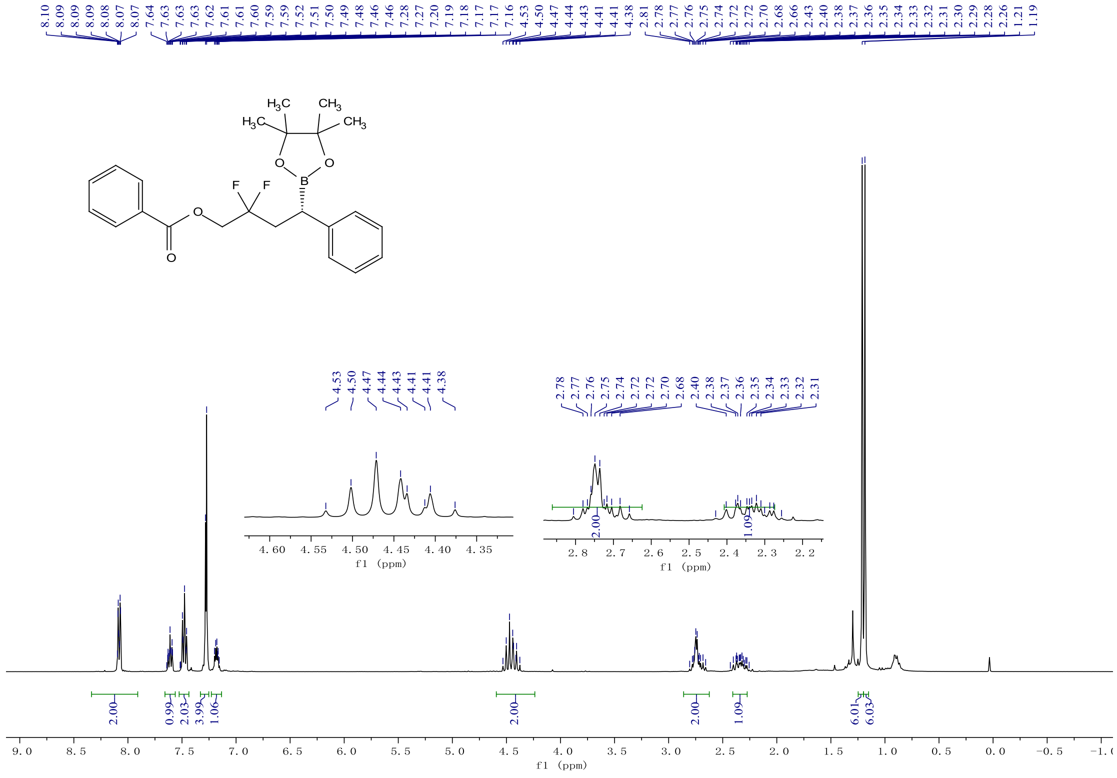

^{1}H-NMR of compound **4k** (400MHz, $CDCl_3$)

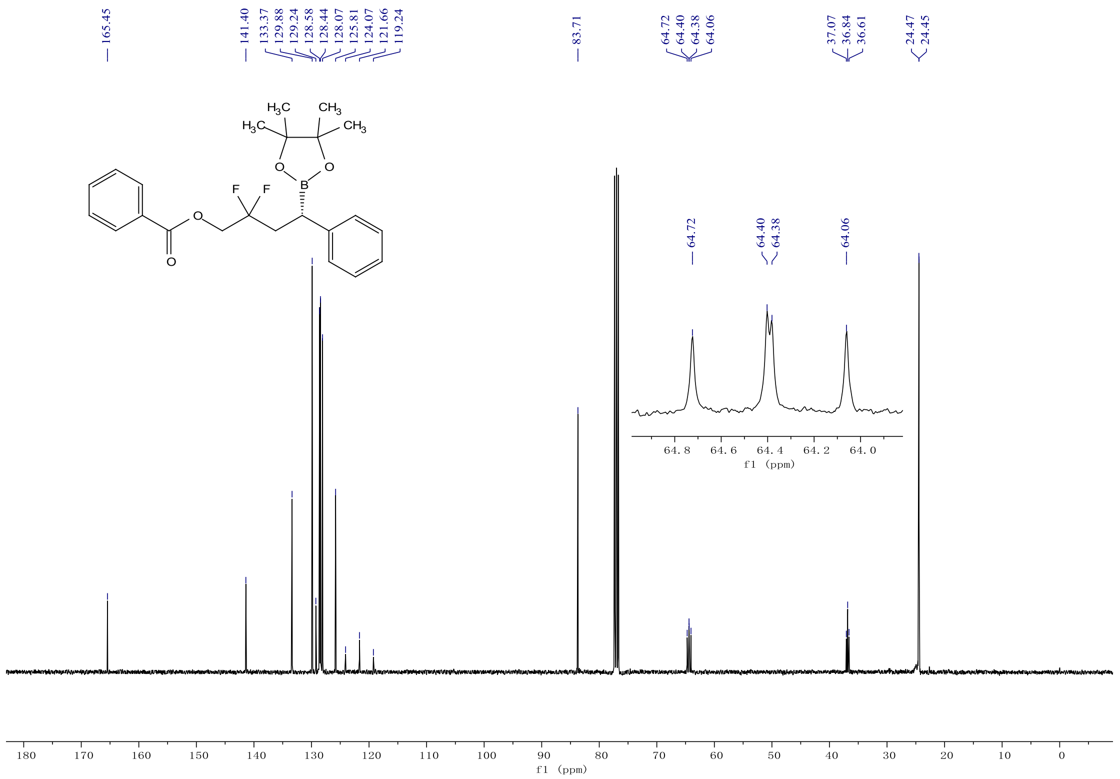

$^{13}C\{^{1}H\}$-NMR of compound **4k** (101MHz, $CDCl_3$)

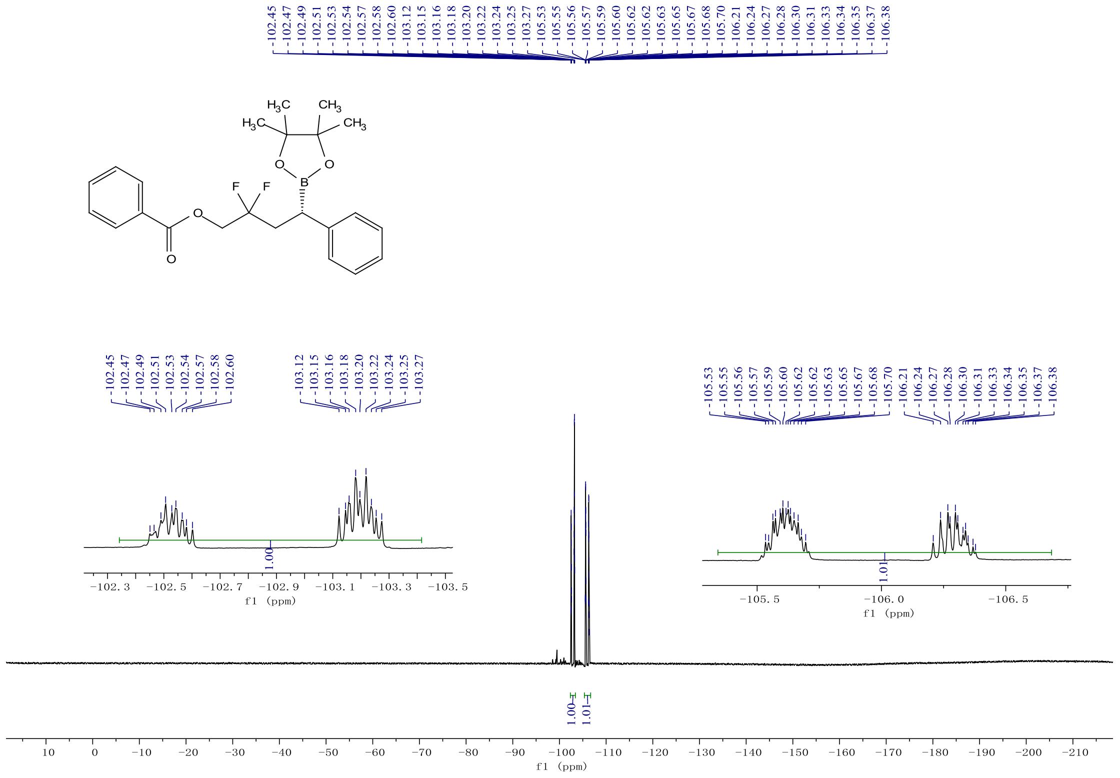

^{19}F-NMR of compound **4k** (377MHz, $CDCl_3$)

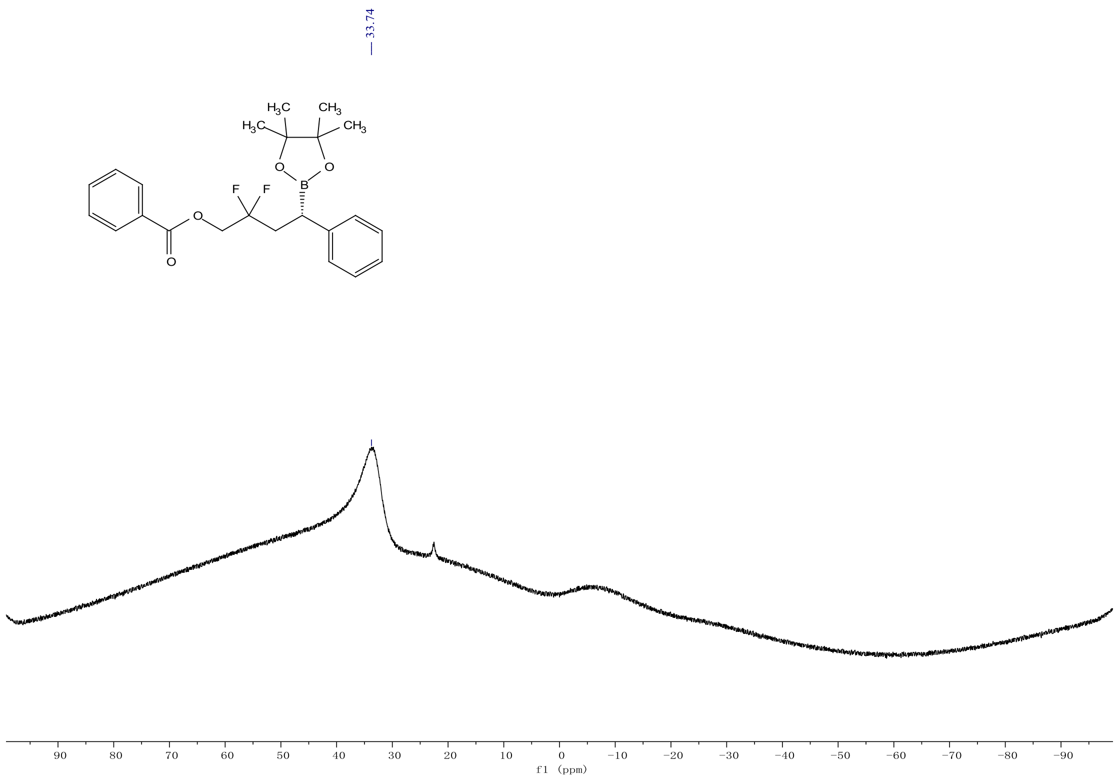

^{11}B-NMR of compound **4j** (128MHz, $CDCl_3$)

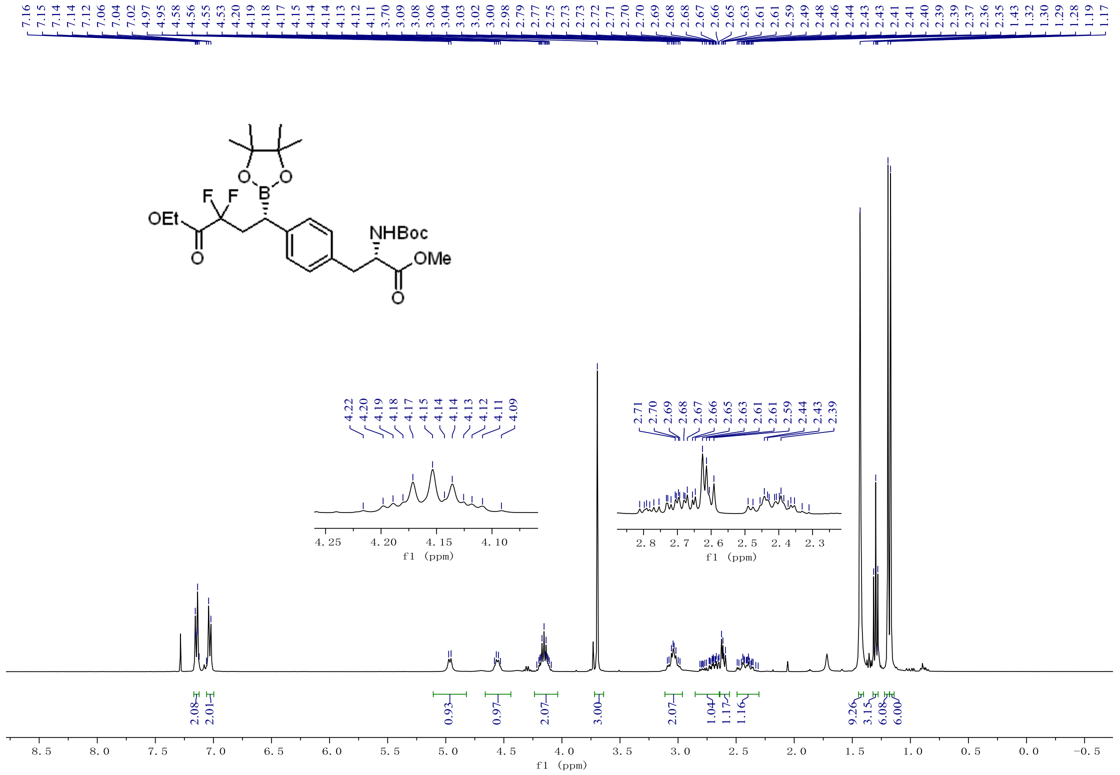

^{1}H-NMR of compound **6a** (400MHz, $CDCl_3$)

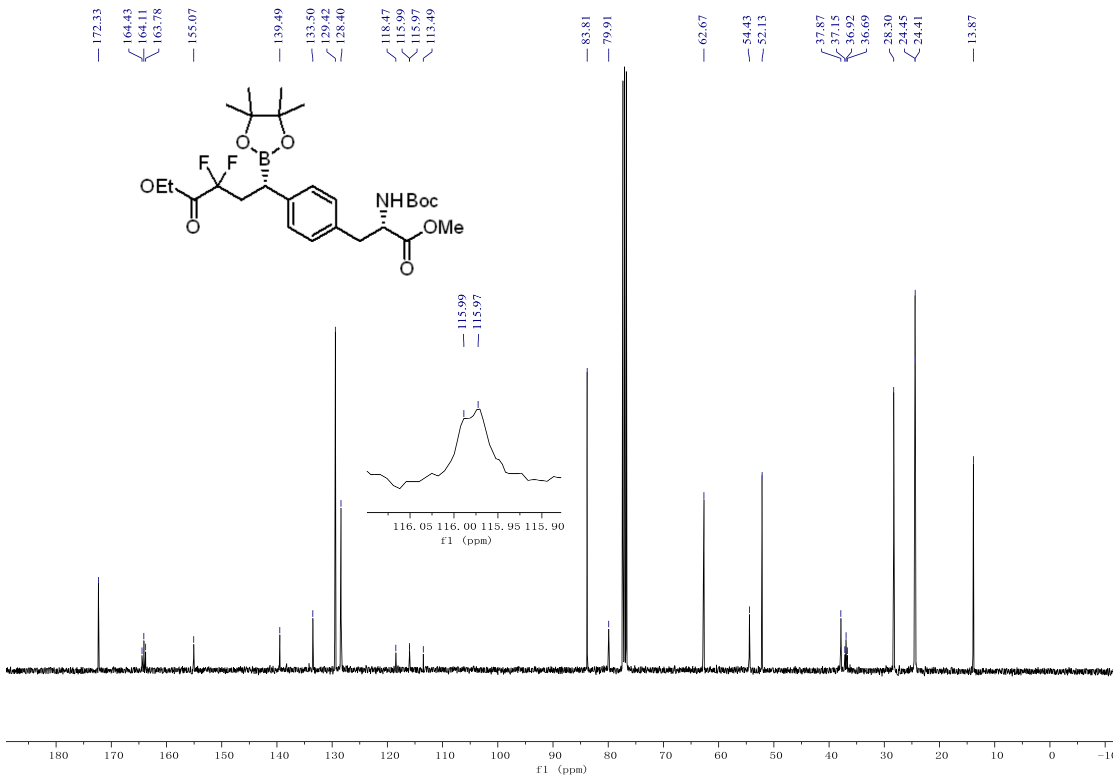

$^{13}C\{^{1}H\}$-NMR of compound **6a** (101MHz, $CDCl_3$)

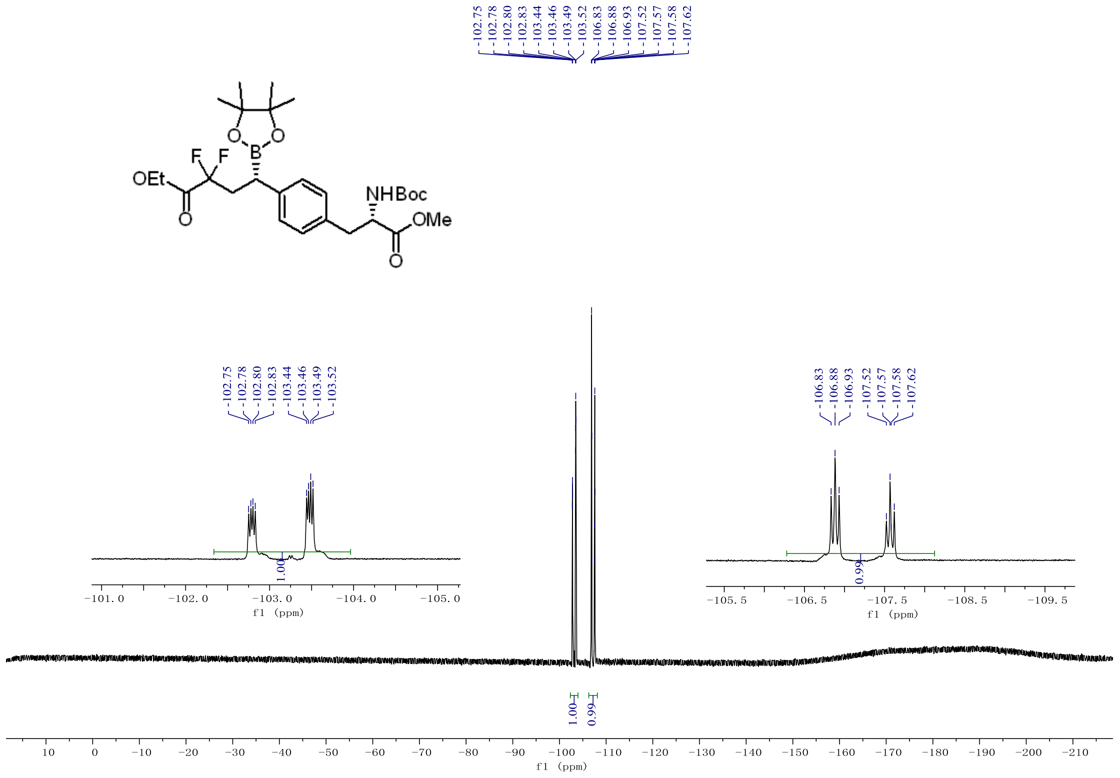

^{19}F-NMR of compound **6a** (377MHz, $CDCl_3$)

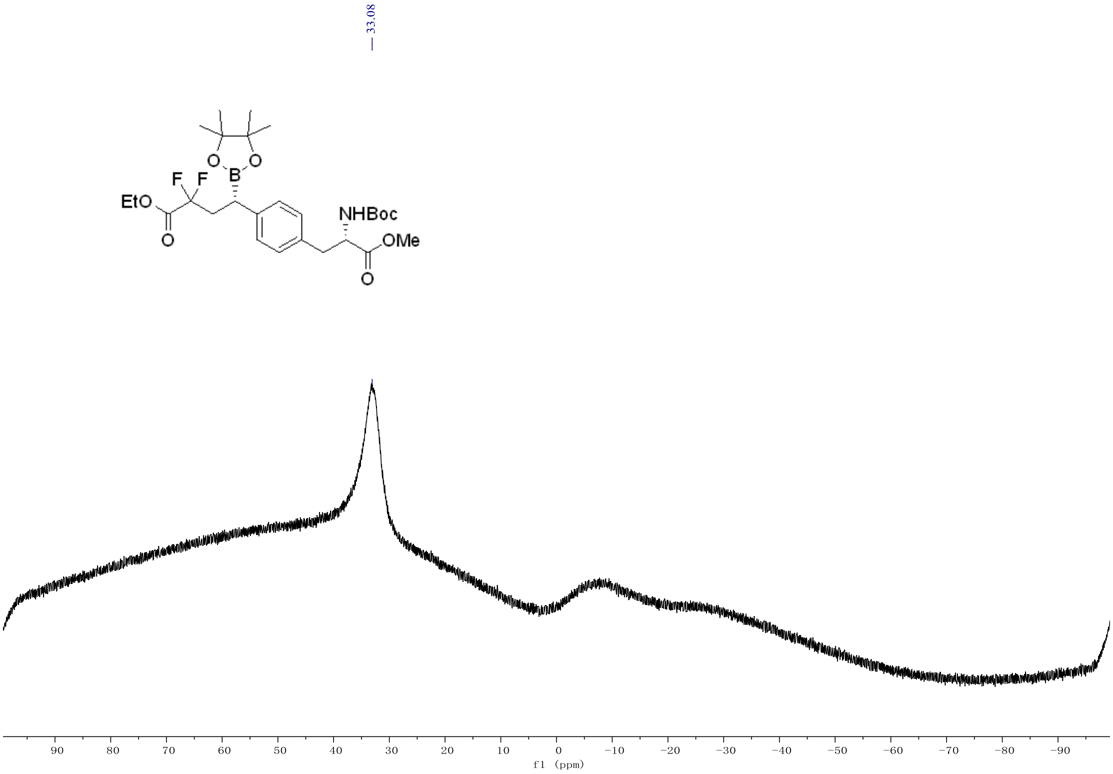

^{11}B-NMR of compound **6a** (128MHz, $CDCl_3$)

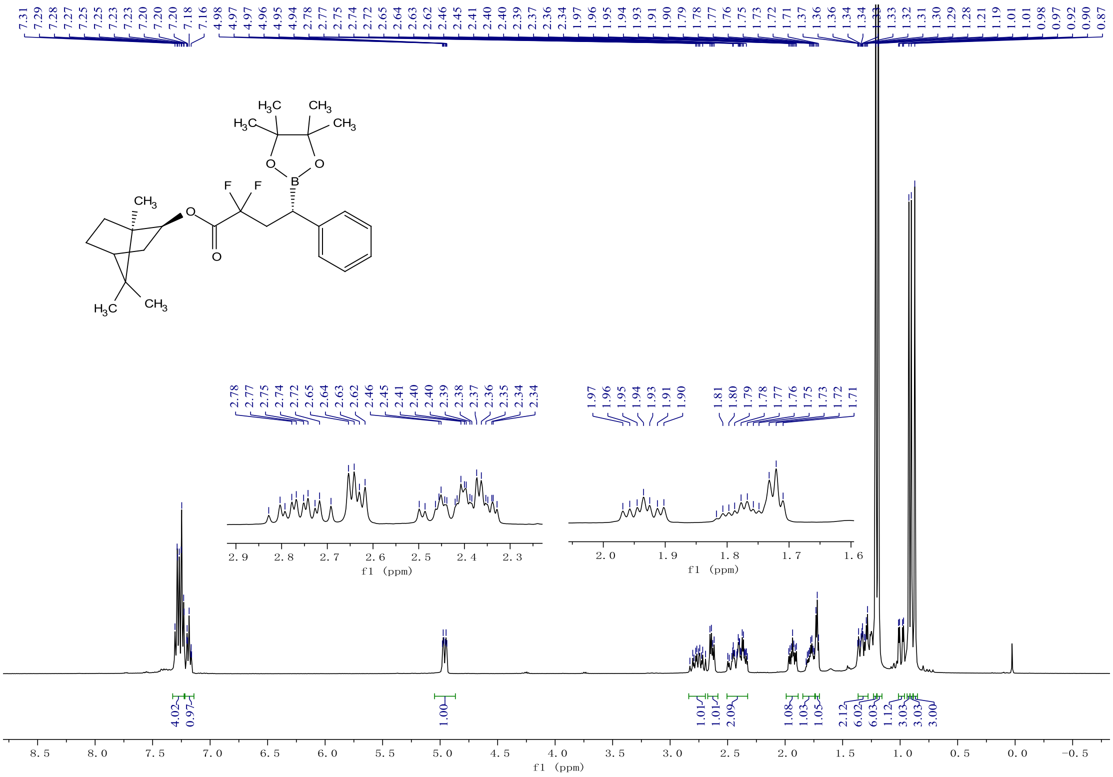

^{1}H-NMR of compound **6b** (400MHz, $CDCl_3$)

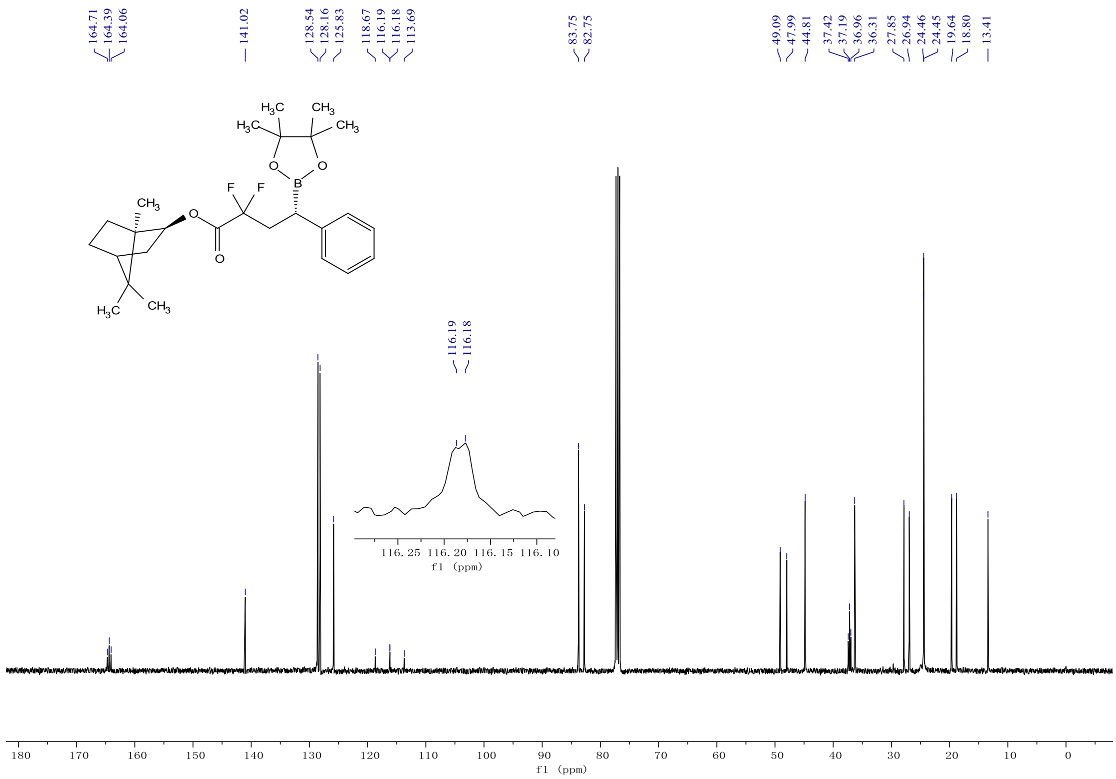

^{13}C{^{1}H}-NMR of compound **6b** (101MHz, $CDCl_3$)

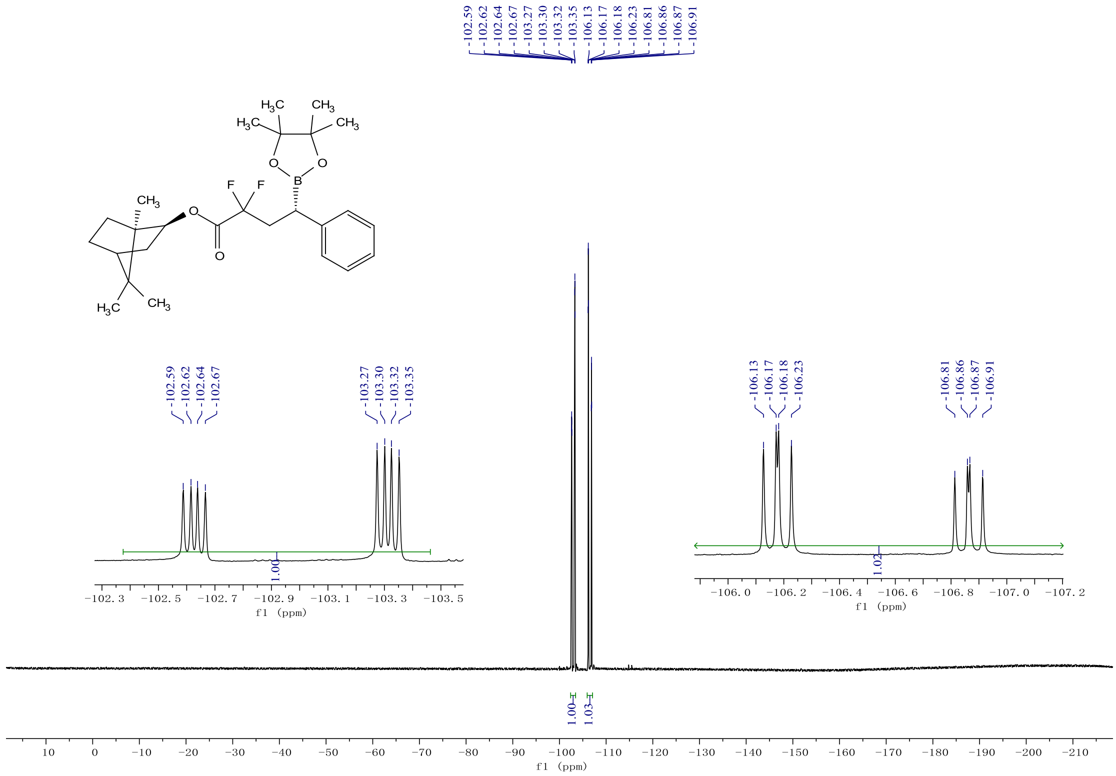

^{19}F-NMR of compound **6b** (377MHz, $CDCl_3$)

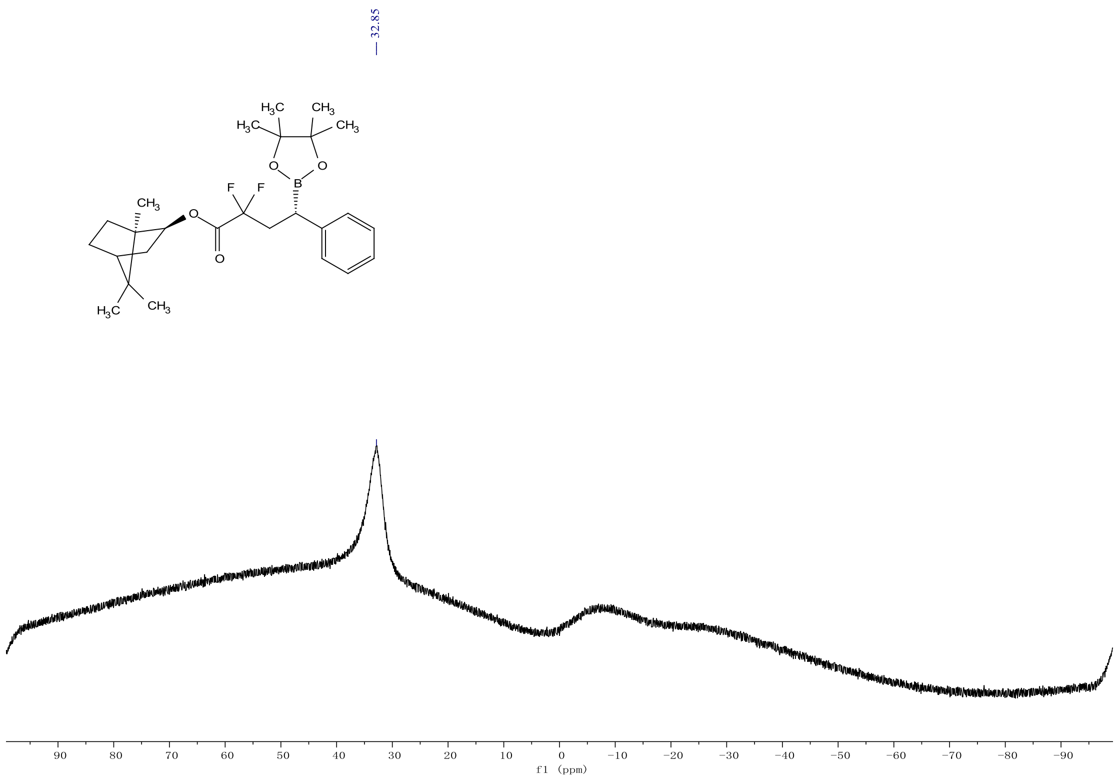

^{11}B-NMR of compound **6b** (128MHz, $CDCl_3$)

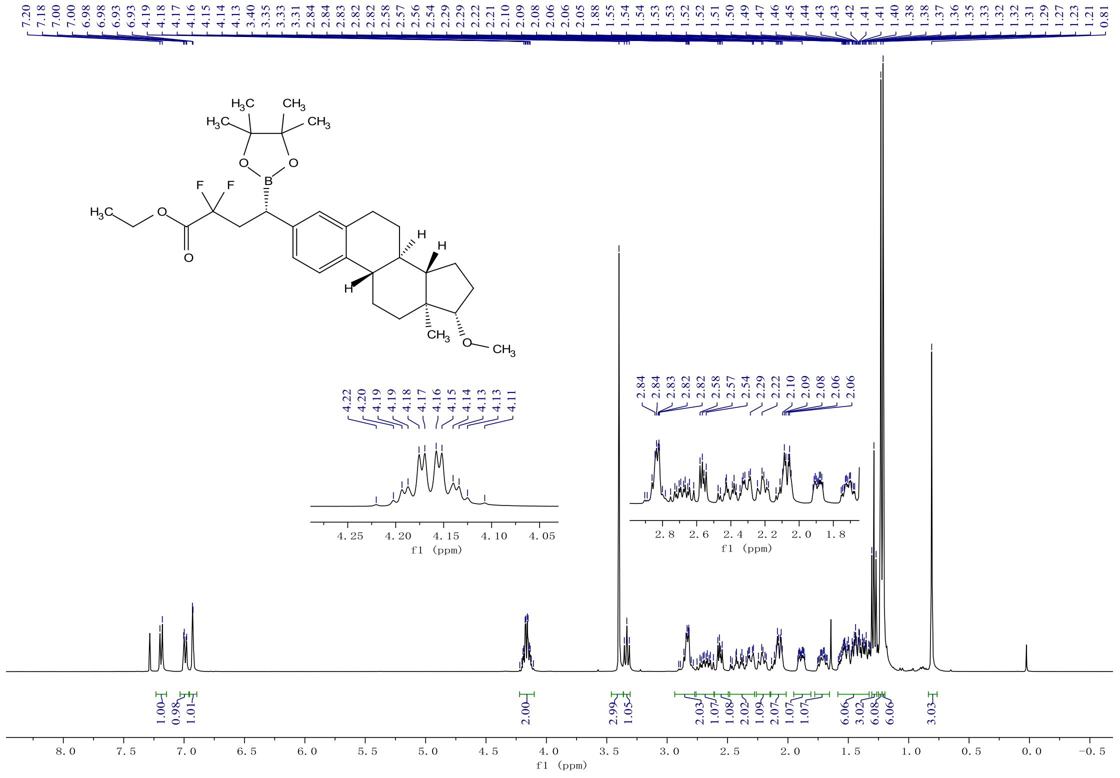

^{1}H-NMR of compound **6c** (400MHz, $CDCl_3$)

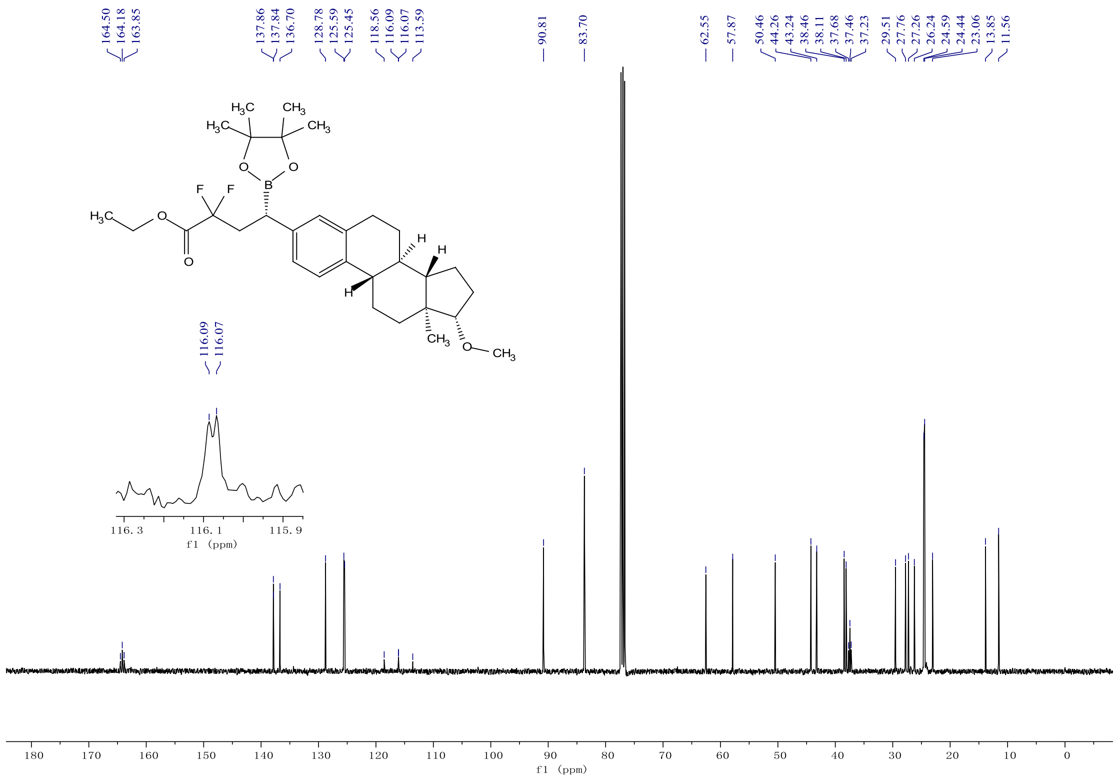

$^{13}C\{^{1}H\}$-NMR of compound **6c** (101MHz, $CDCl_3$)

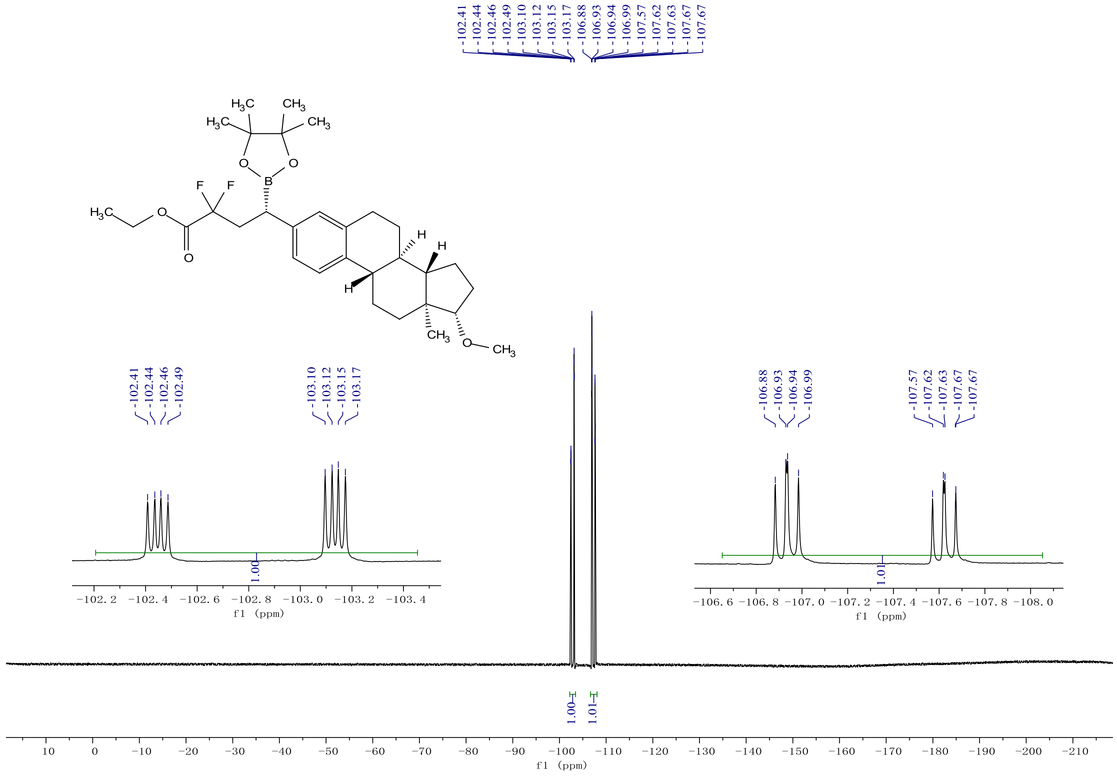

^{19}F-NMR of compound **6c** (377MHz, $CDCl_3$)

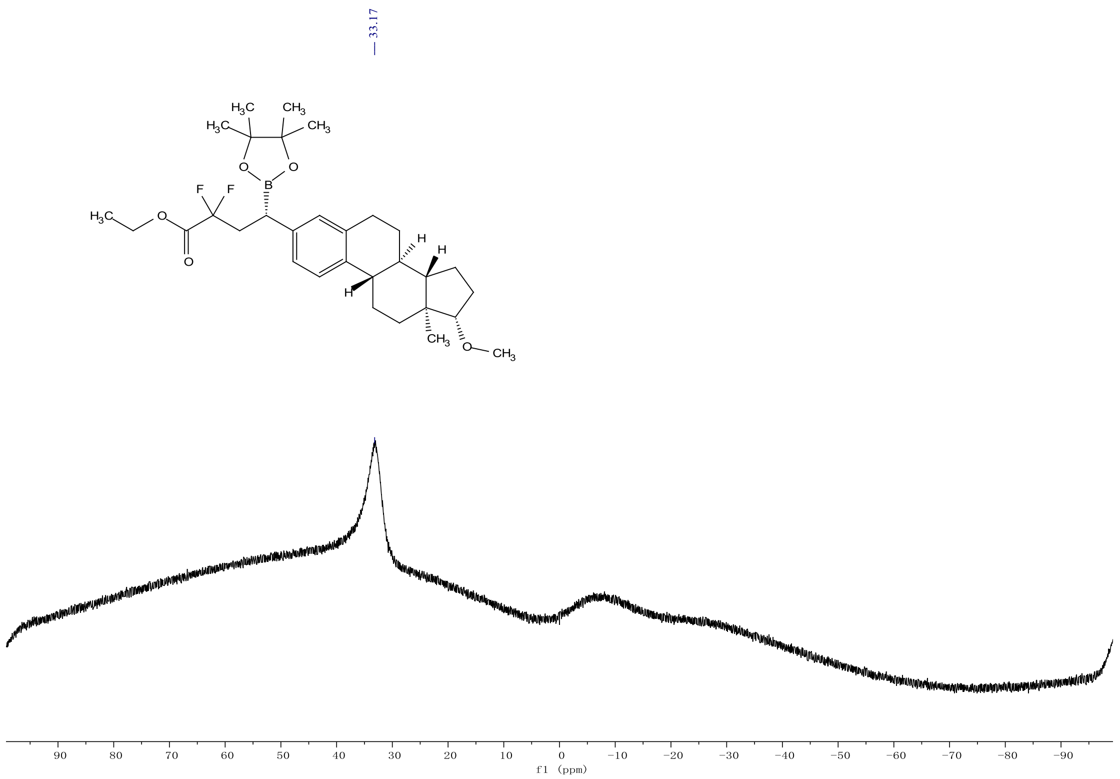

^{11}B-NMR of compound **6c** (128MHz, $CDCl_3$)

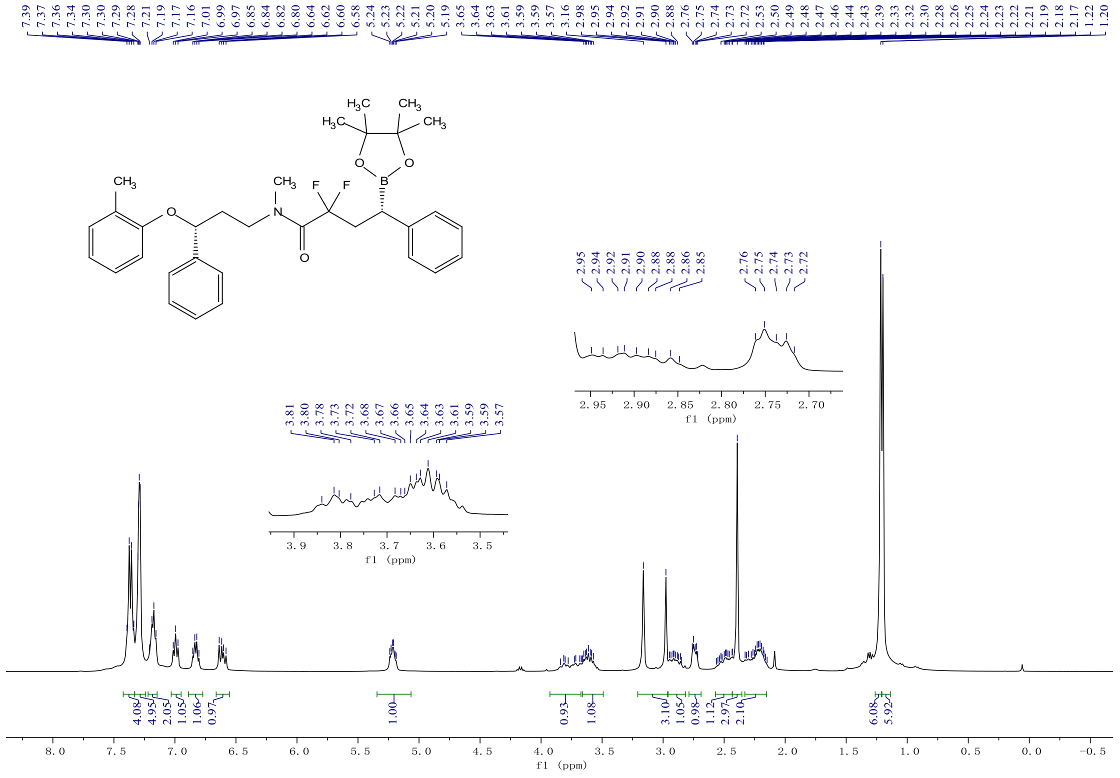

^{1}H-NMR of compound **6d** (400MHz, $CDCl_3$)

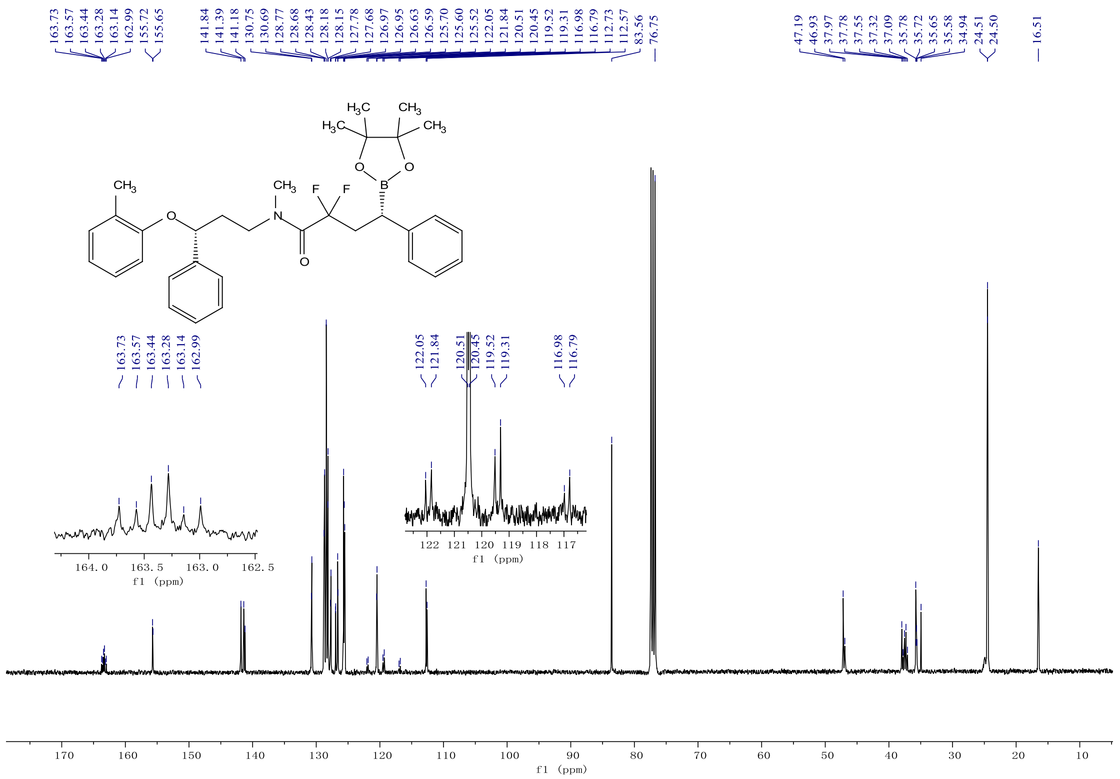

$^{13}C\{^1H\}$-NMR of compound **6d** (101MHz, $CDCl_3$)

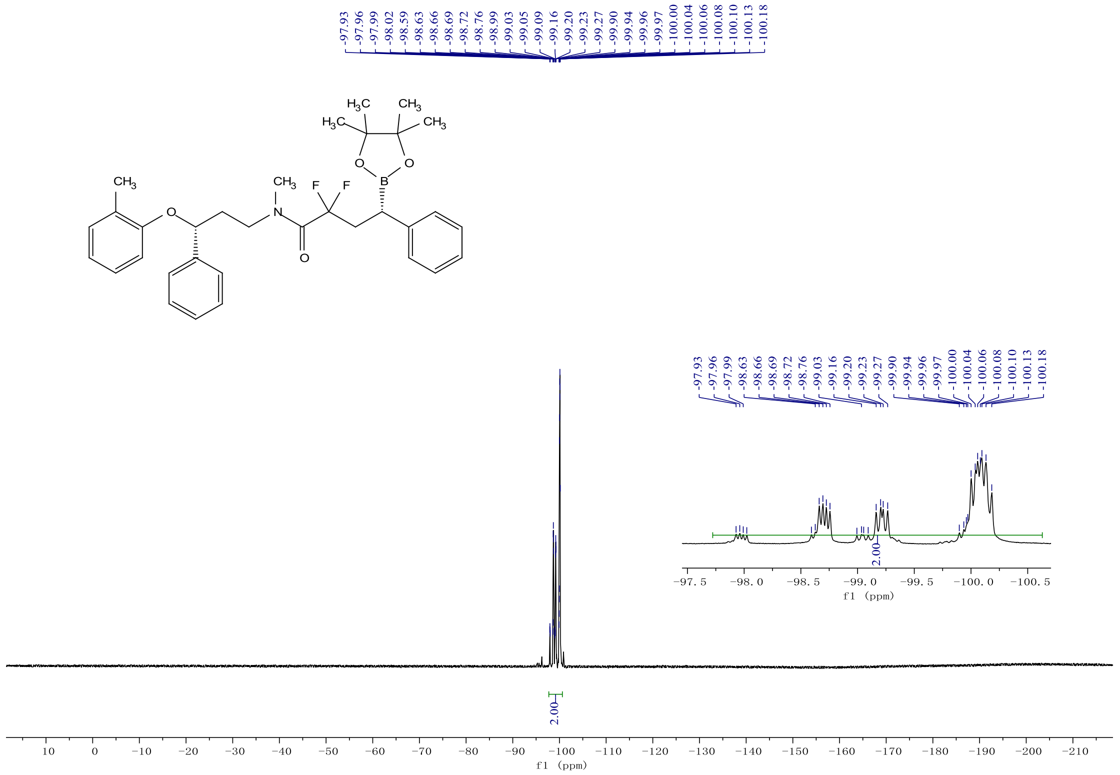

^{19}F-NMR of compound **6d** (377MHz, $CDCl_3$)

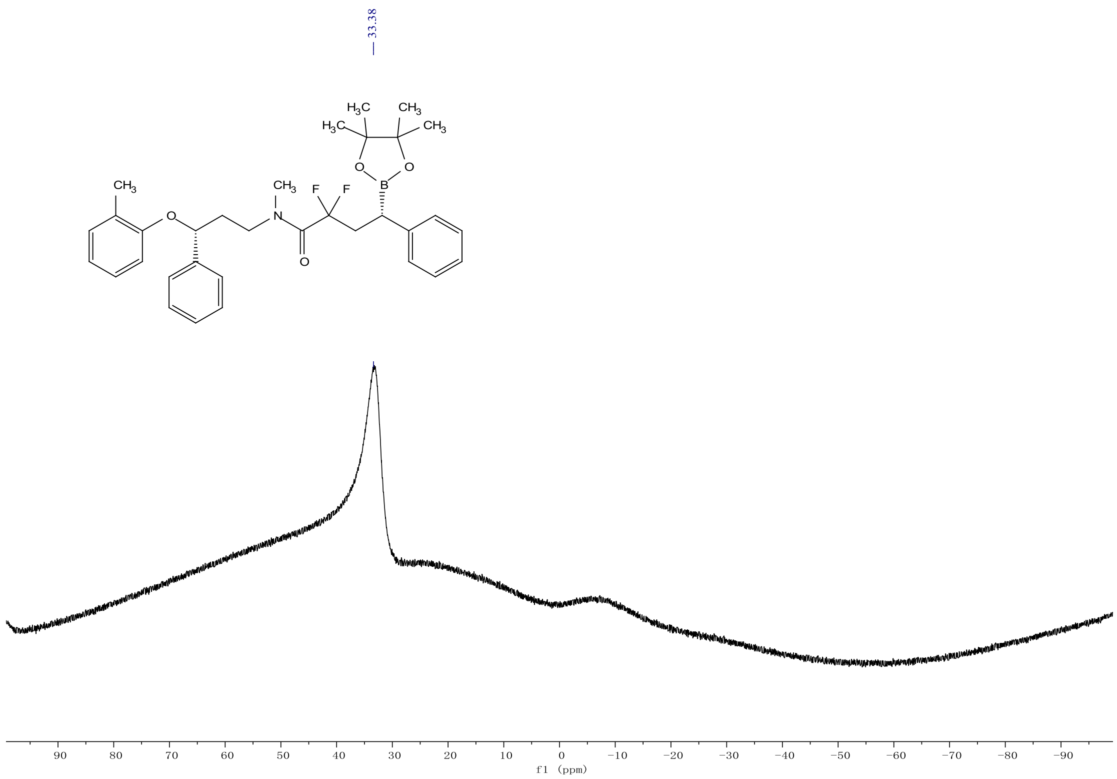

^{11}B-NMR of compound **6d** (128MHz, $CDCl_3$)

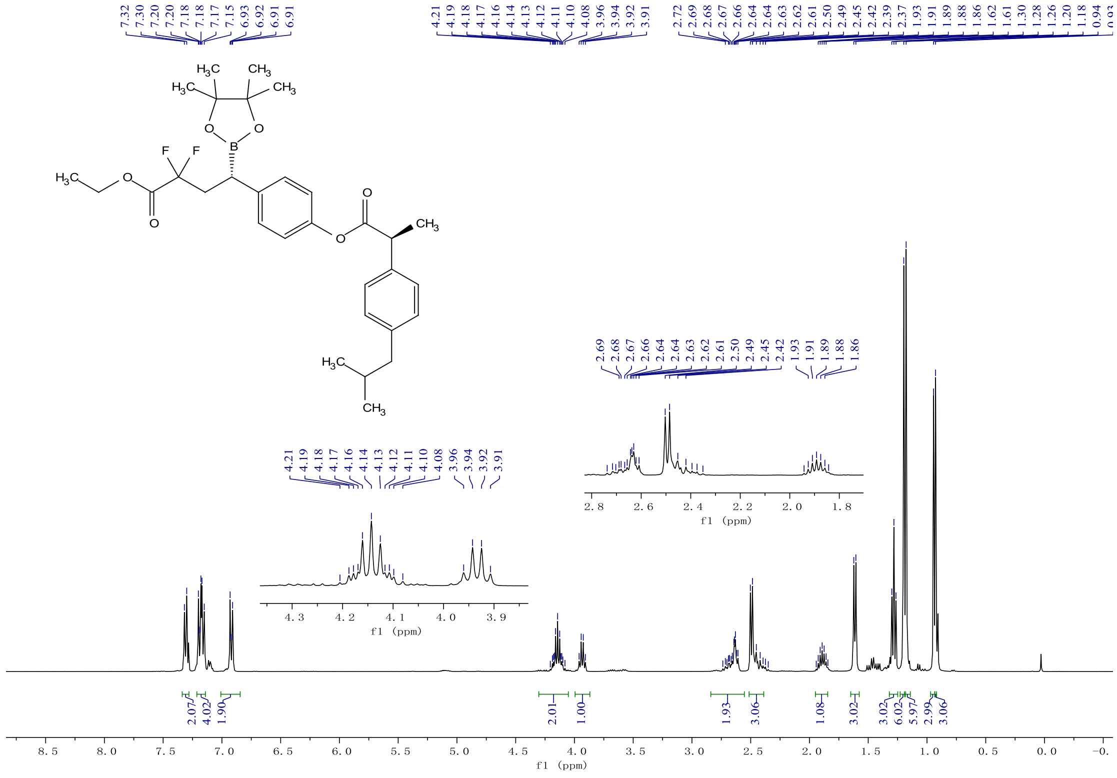

^{1}H-NMR of compound **6e** (400MHz, $CDCl_3$)

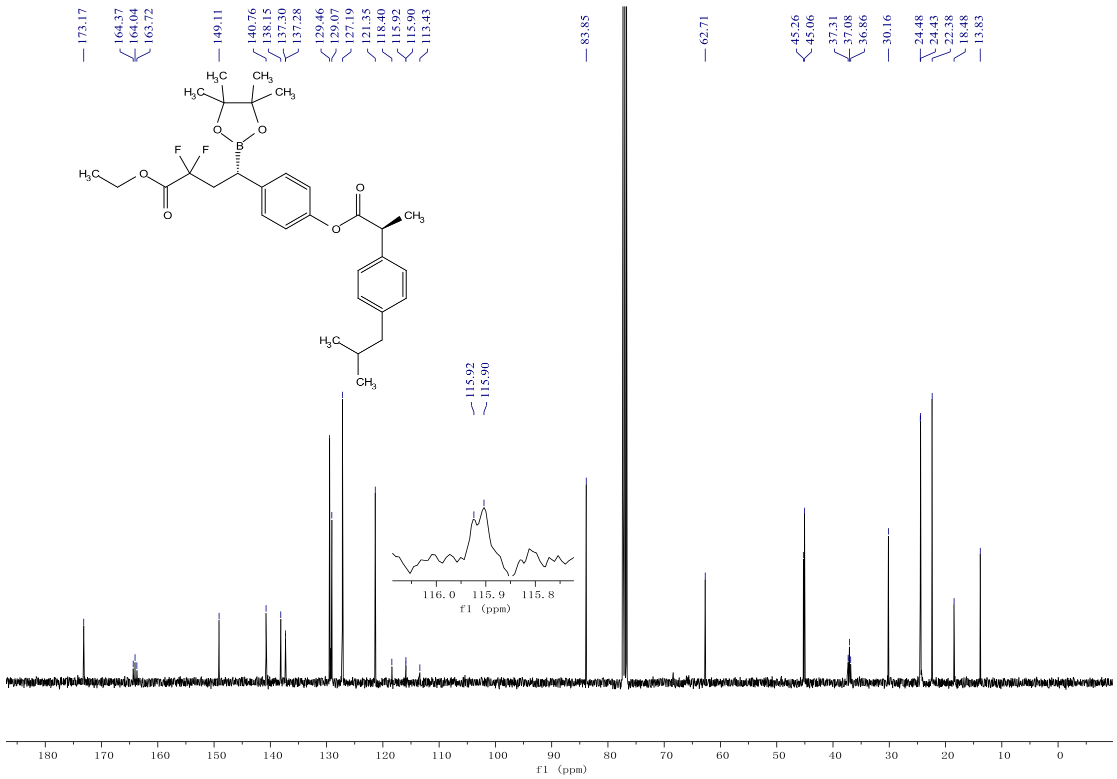

$^{13}C\{^{1}H\}$-NMR of compound **6e** (101MHz, $CDCl_3$)

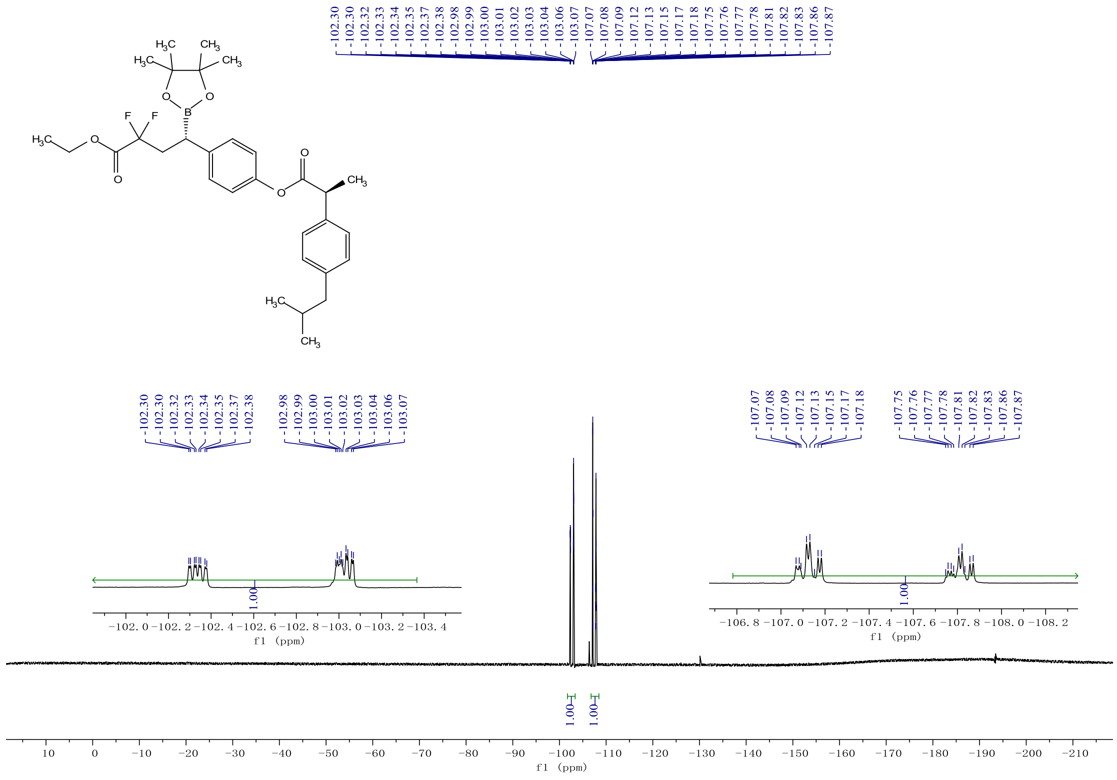

^{19}F-NMR of compound **6e** (377MHz, $CDCl_3$)

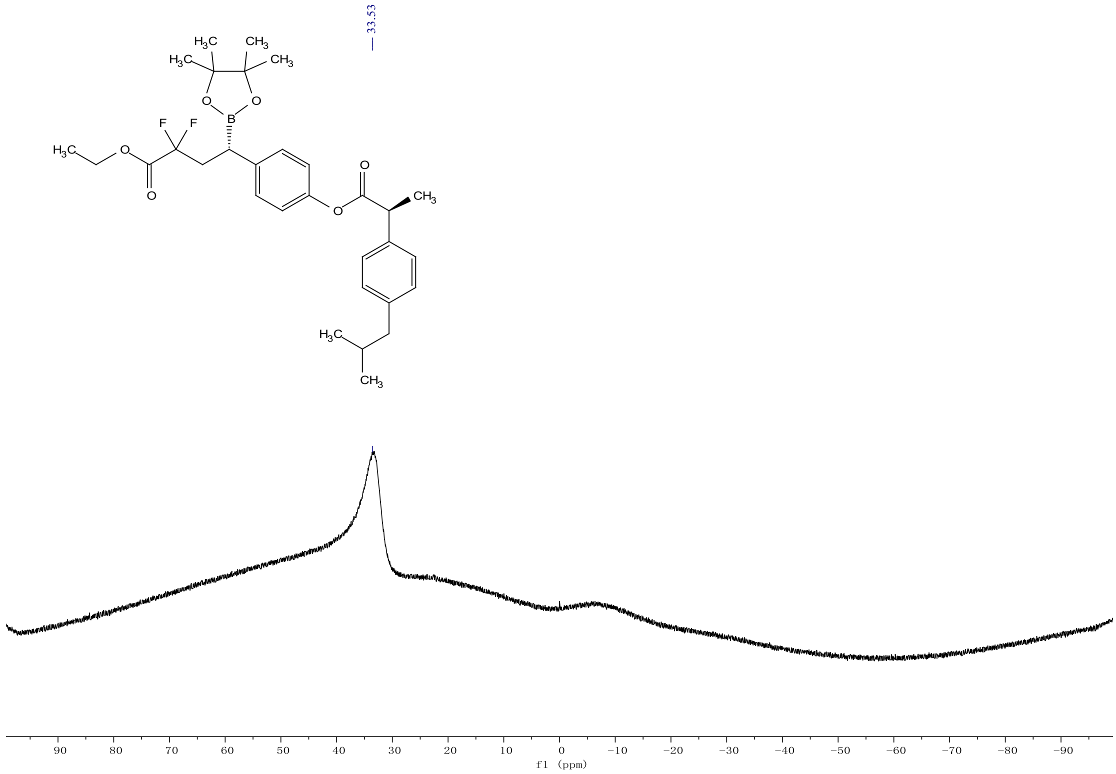

^{11}B-NMR of compound **6e** (128MHz, $CDCl_3$)

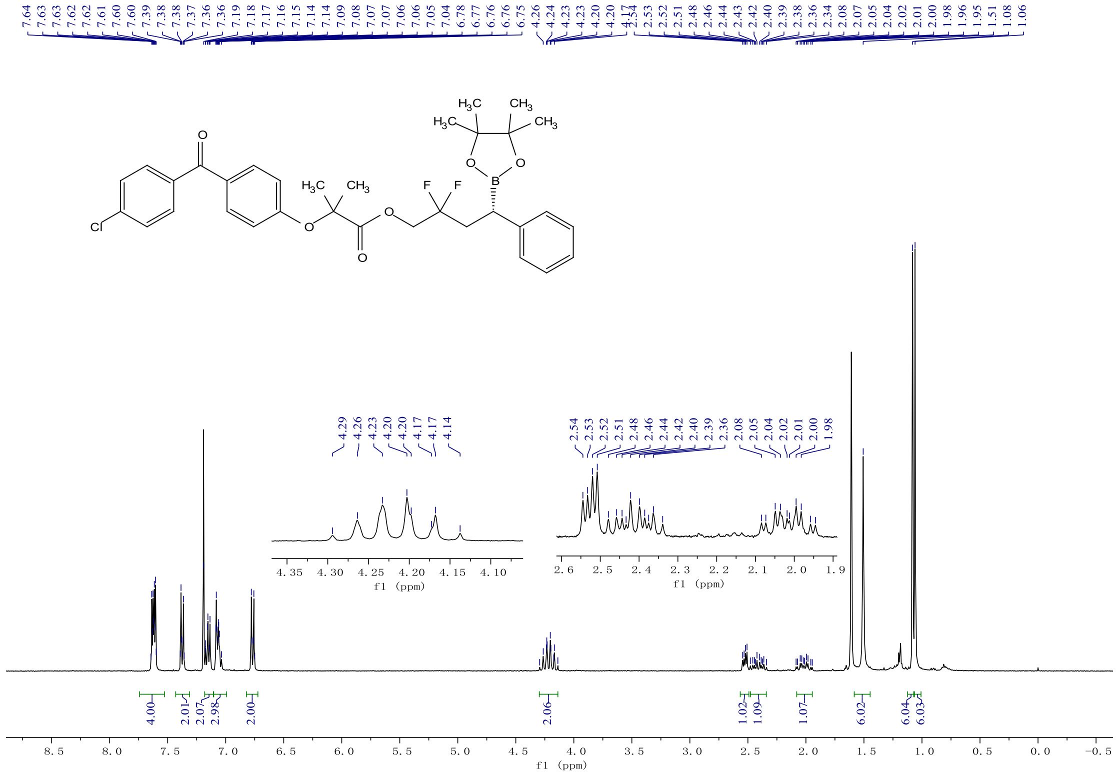

^{1}H-NMR of compound **6f** (400MHz, $CDCl_3$)

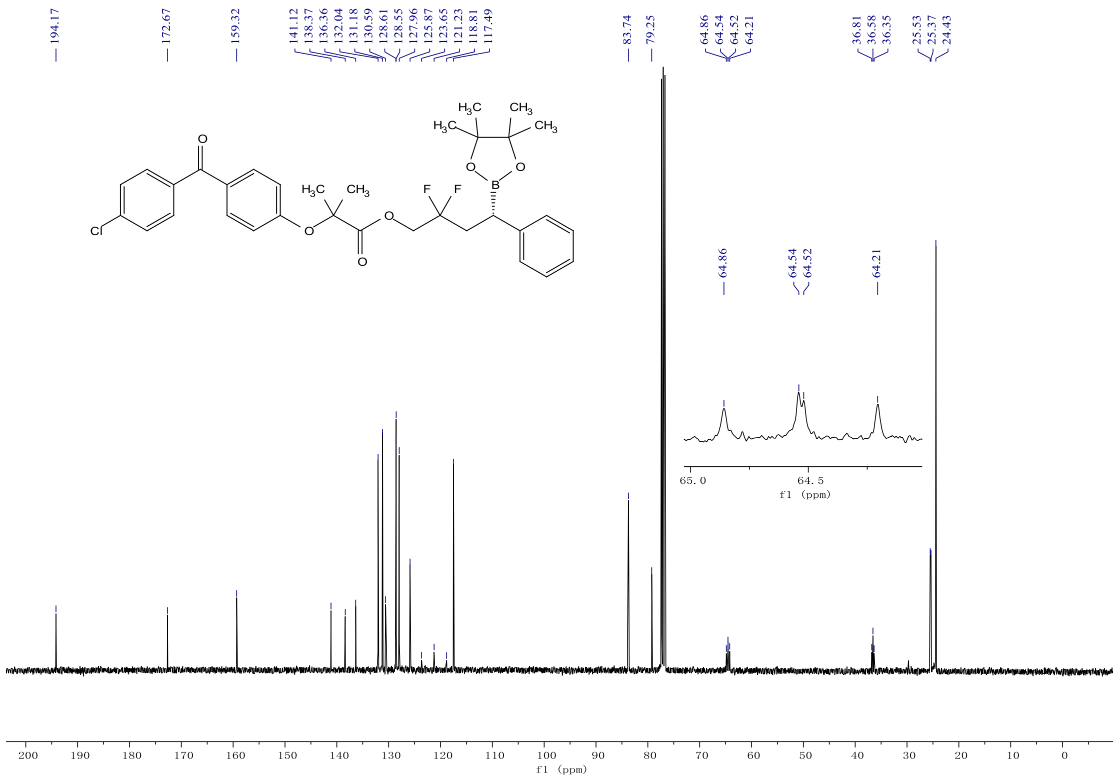

$^{13}C\{^{1}H\}$-NMR of compound **6f** (101MHz, $CDCl_3$)

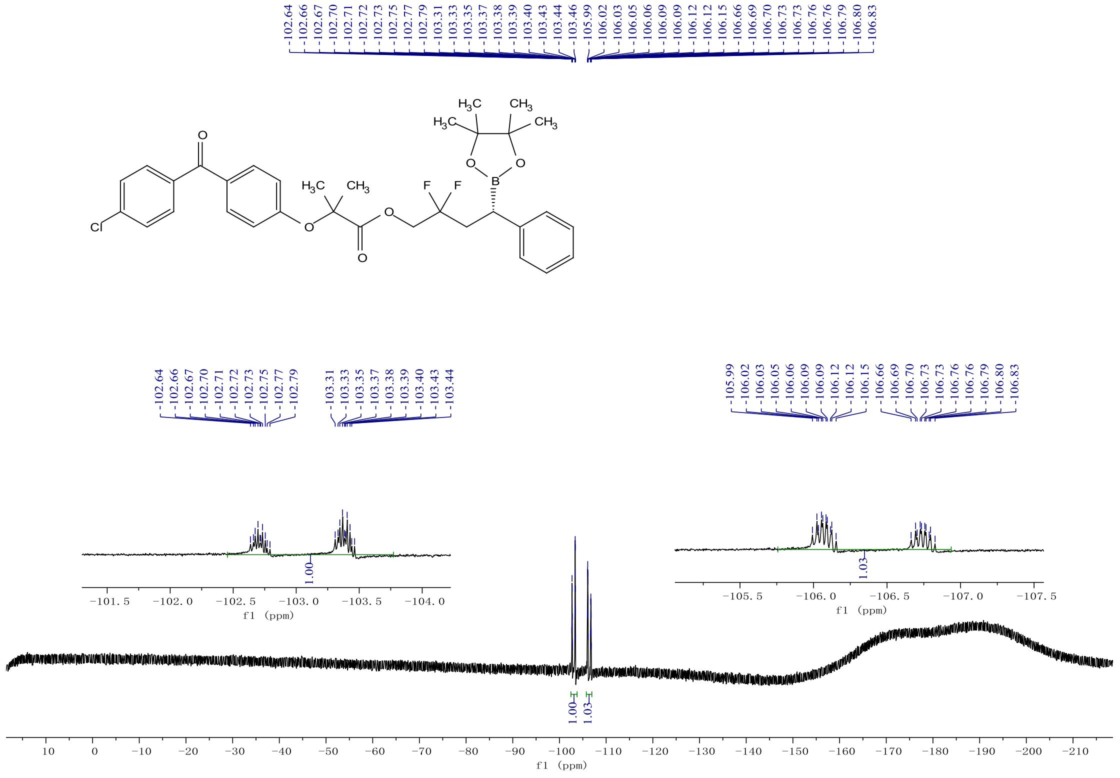

^{19}F-NMR of compound **6f** (377MHz, $CDCl_3$)

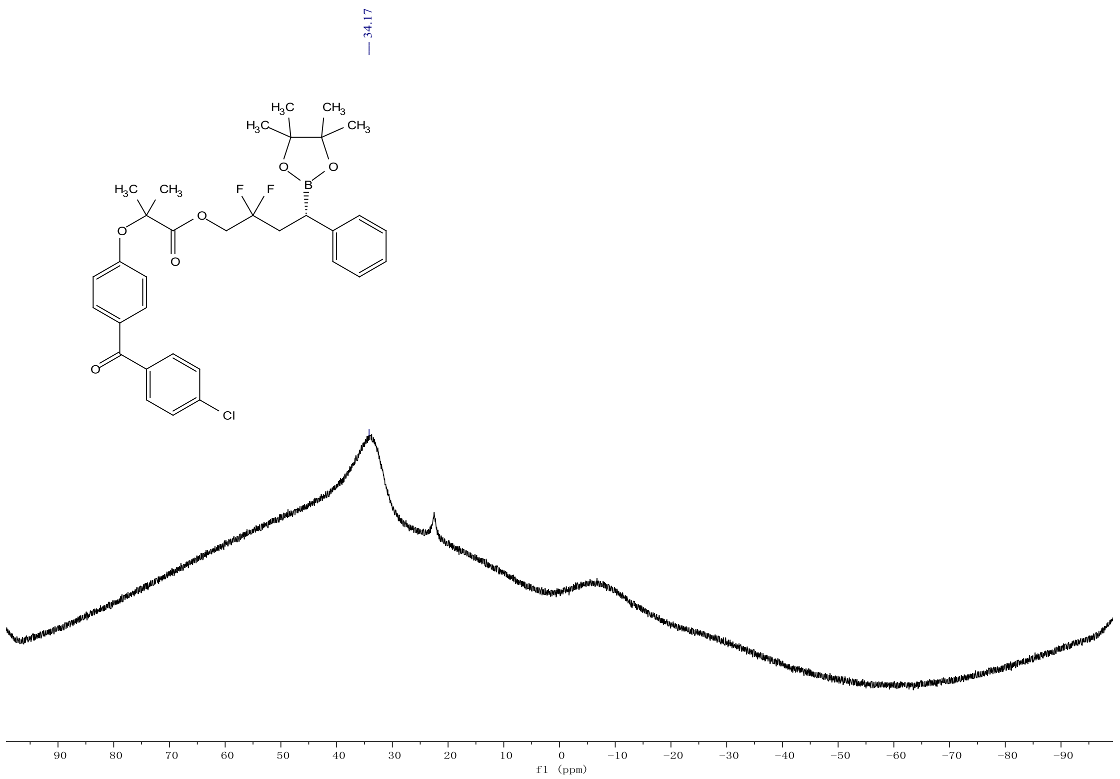

^{11}B-NMR of compound **6f** (128MHz, $CDCl_3$)

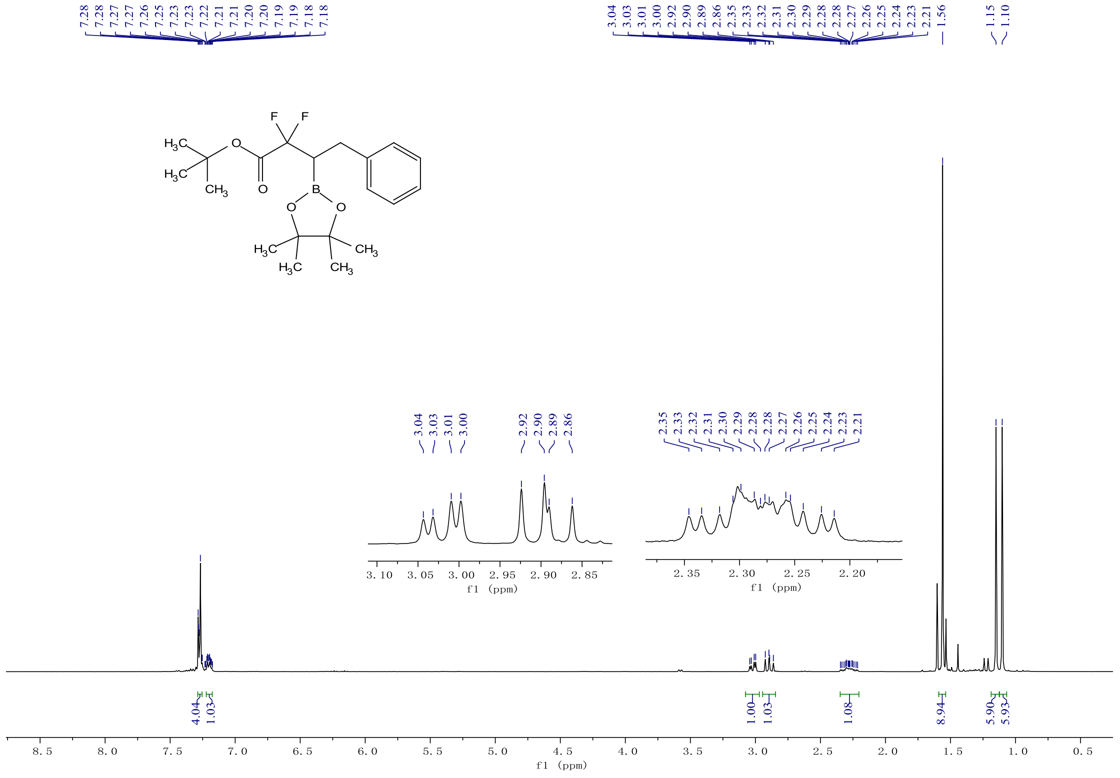

^{1}H-NMR of compound **B1a** (400MHz, $CDCl_3$)

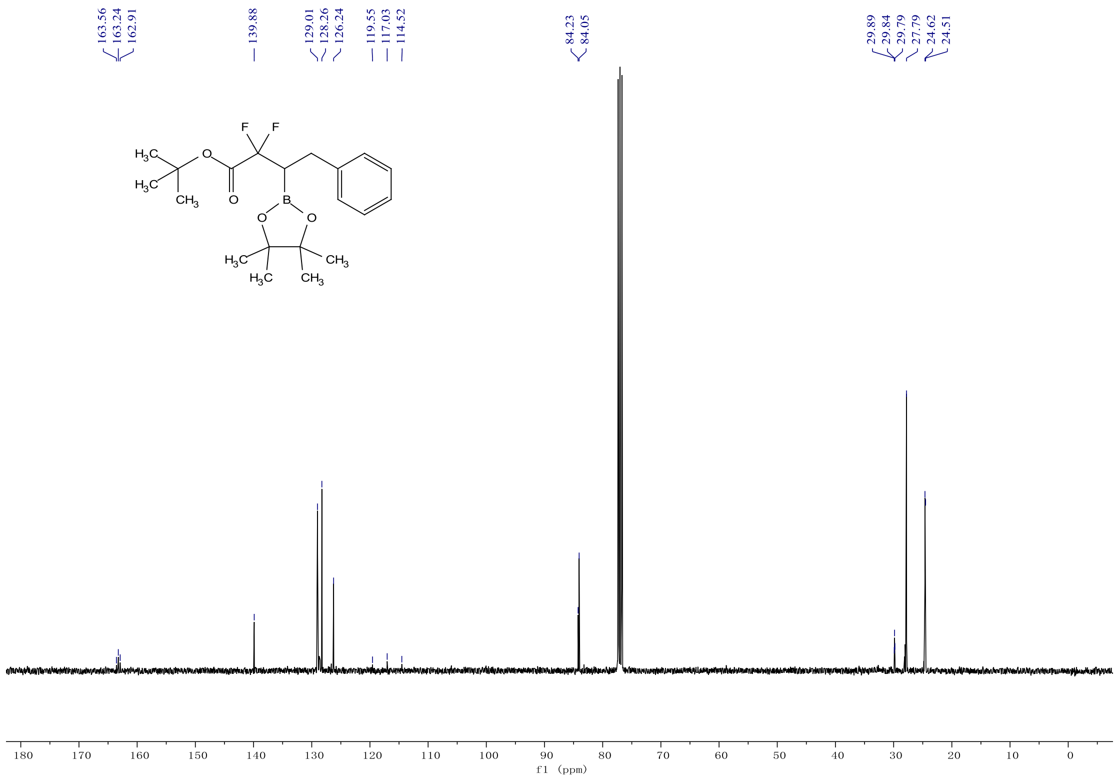

^{13}C{^{1}H}-NMR of compound **B1a** (101MHz, $CDCl_3$)

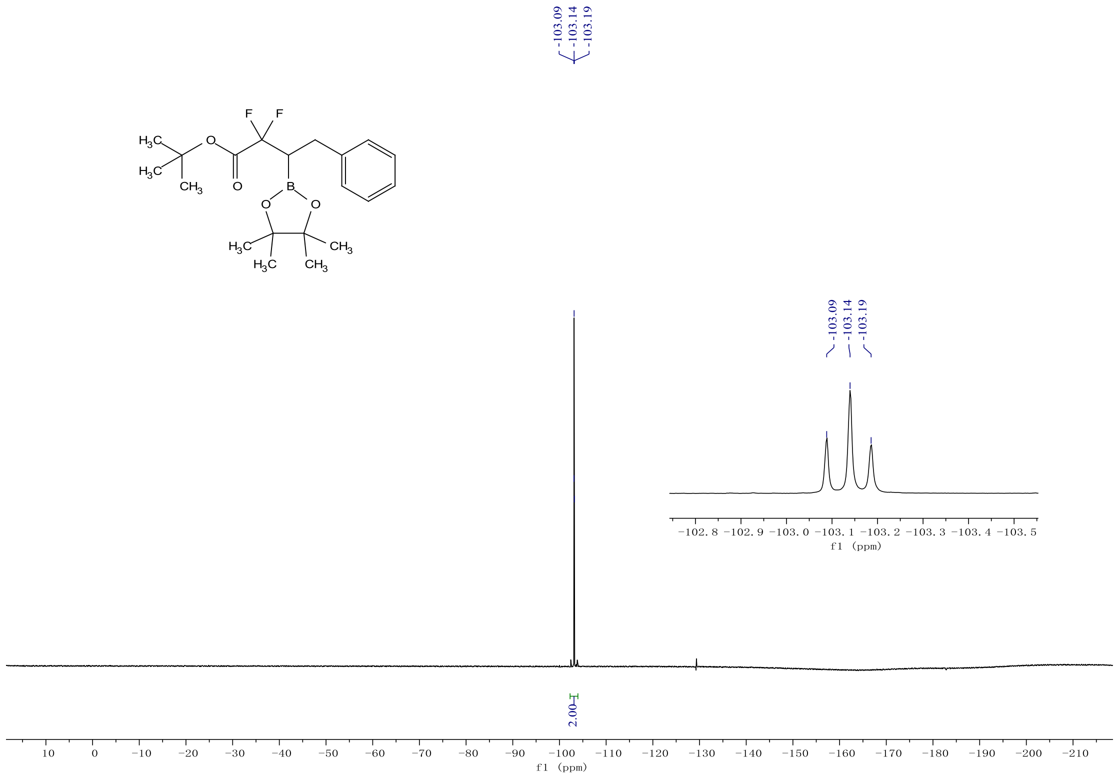

^{19}F-NMR of compound **B1a** (377MHz, $CDCl_3$)

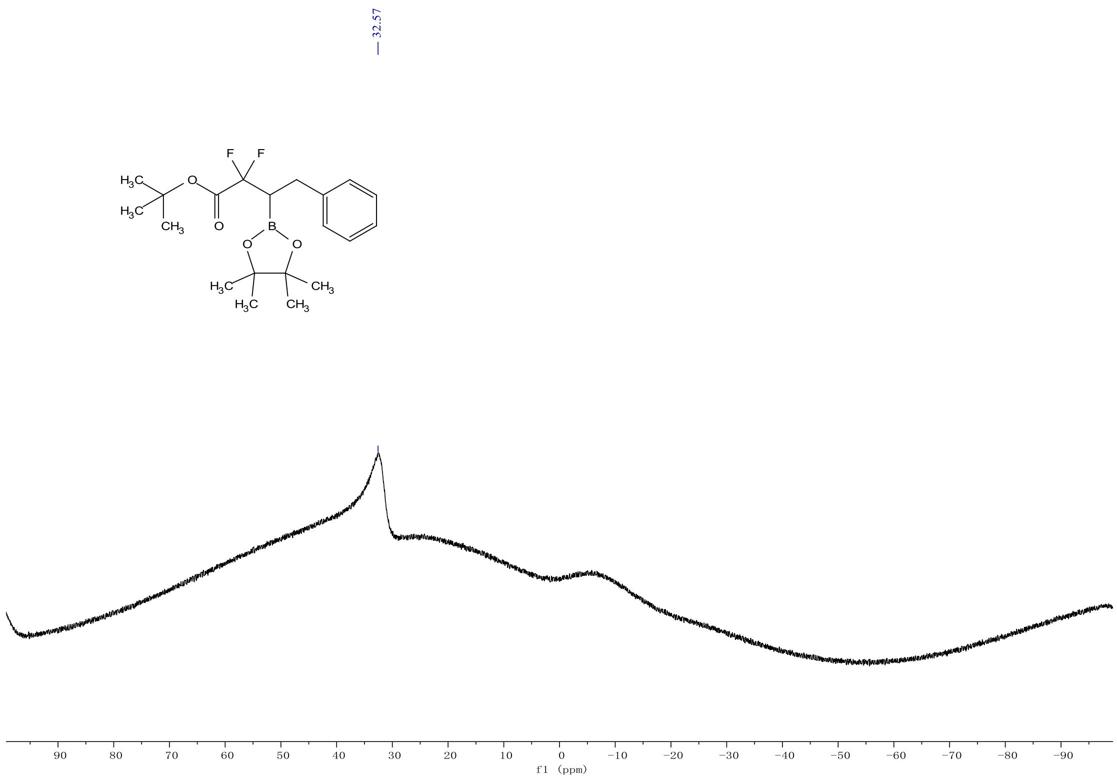

^{11}B-NMR of compound **B1a** (128MHz, $CDCl_3$)

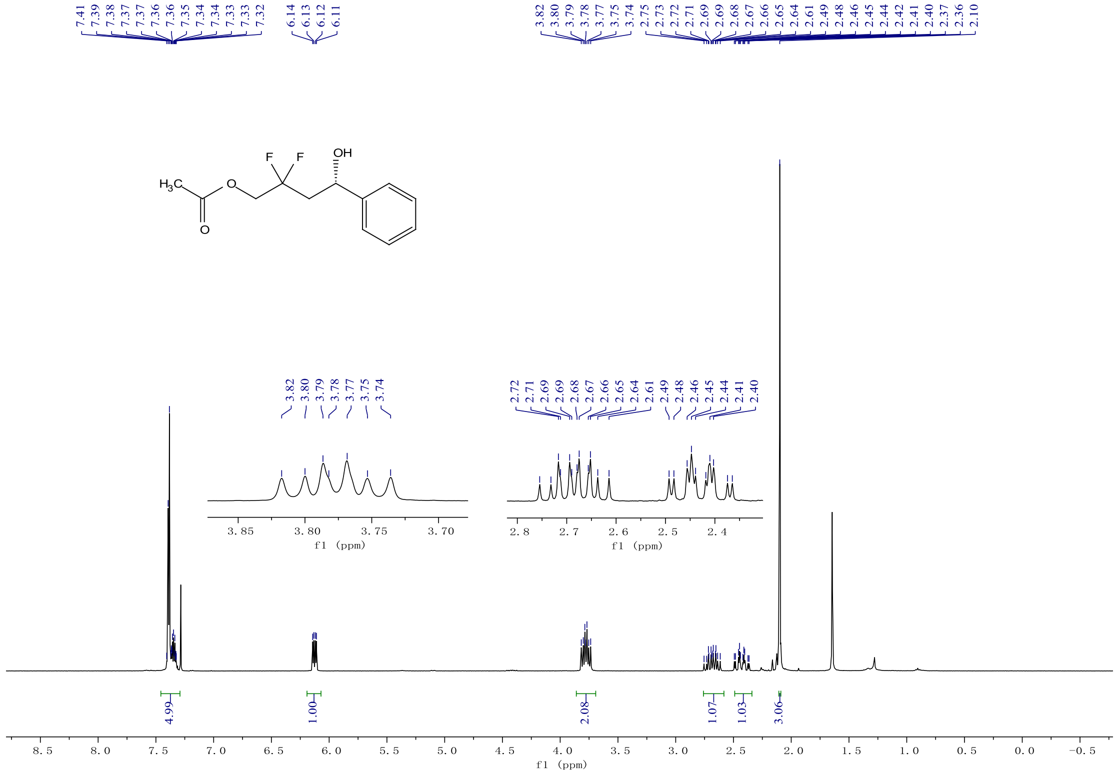

^{1}H-NMR of compound **7’** (400MHz, $CDCl_3$)

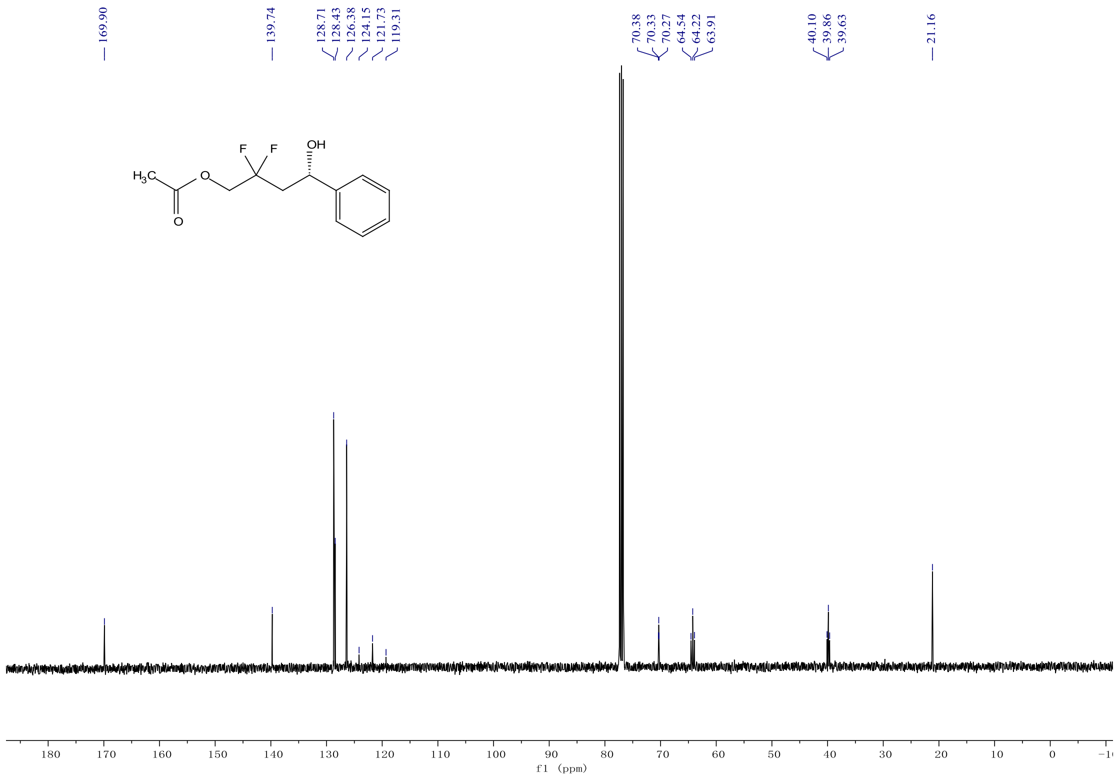

$^{13}C\{^{1}H\}$-NMR of compound **7’** (101MHz, $CDCl_3$)

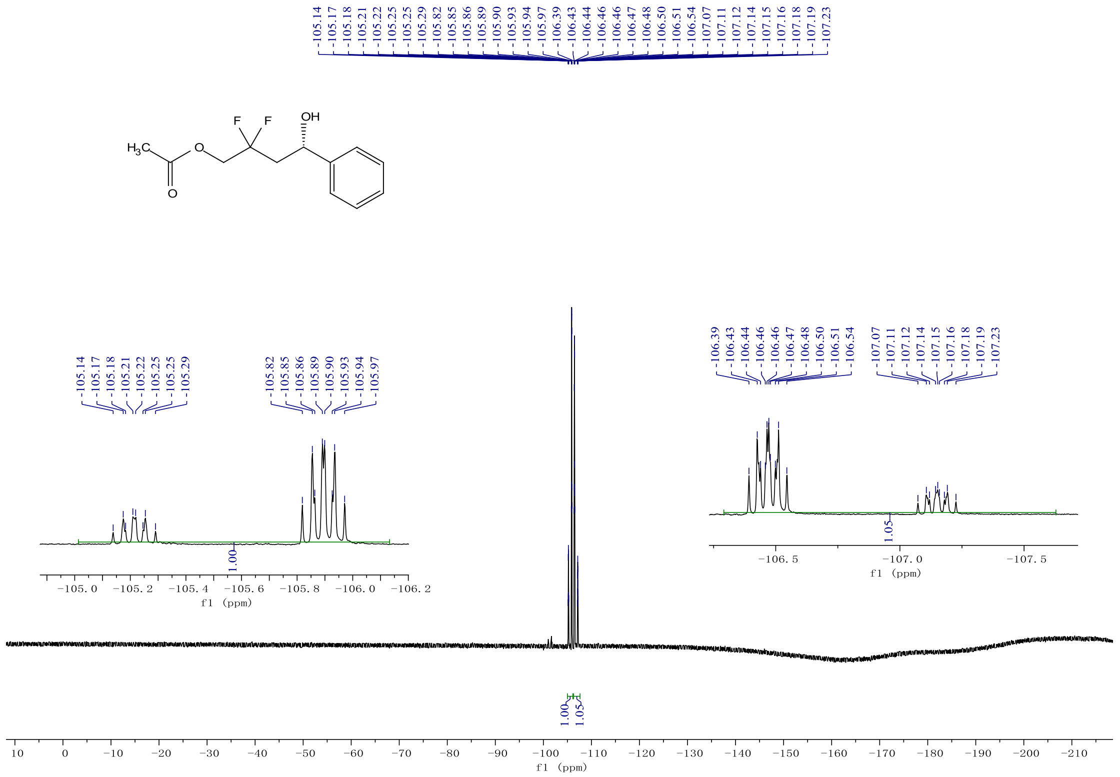

^{19}F-NMR of compound **7’** (377MHz, $CDCl_3$)

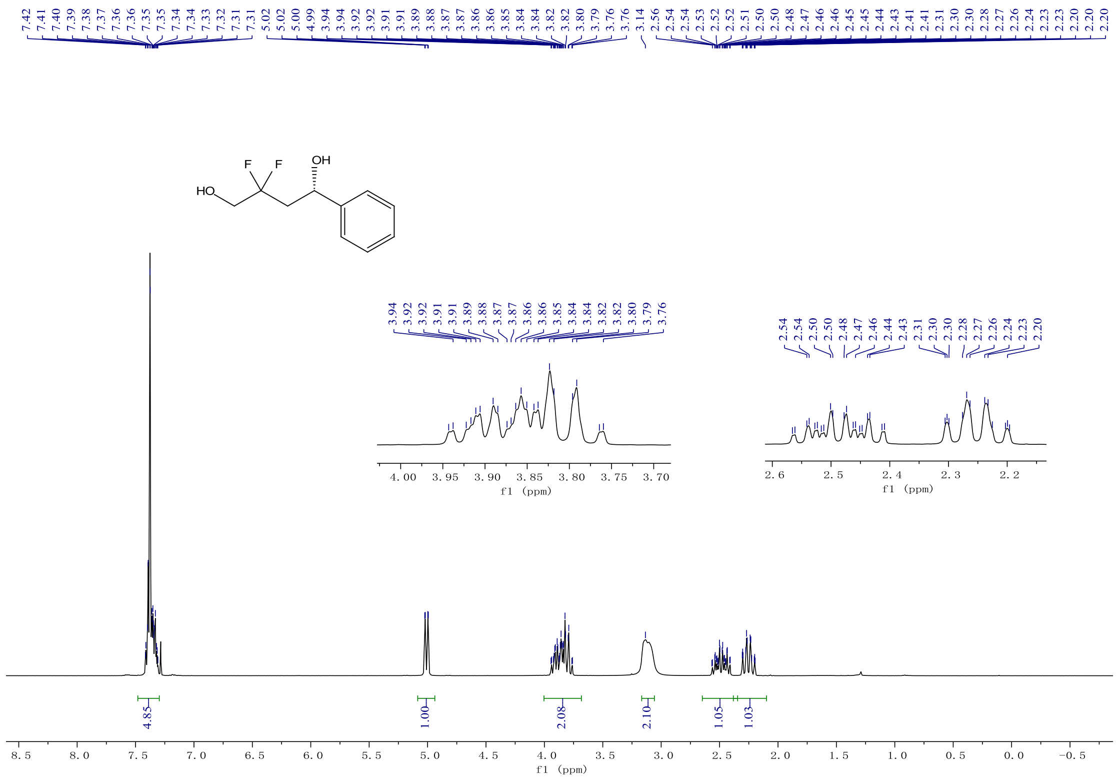

^{1}H-NMR of compound **7** (400MHz, $CDCl_3$)

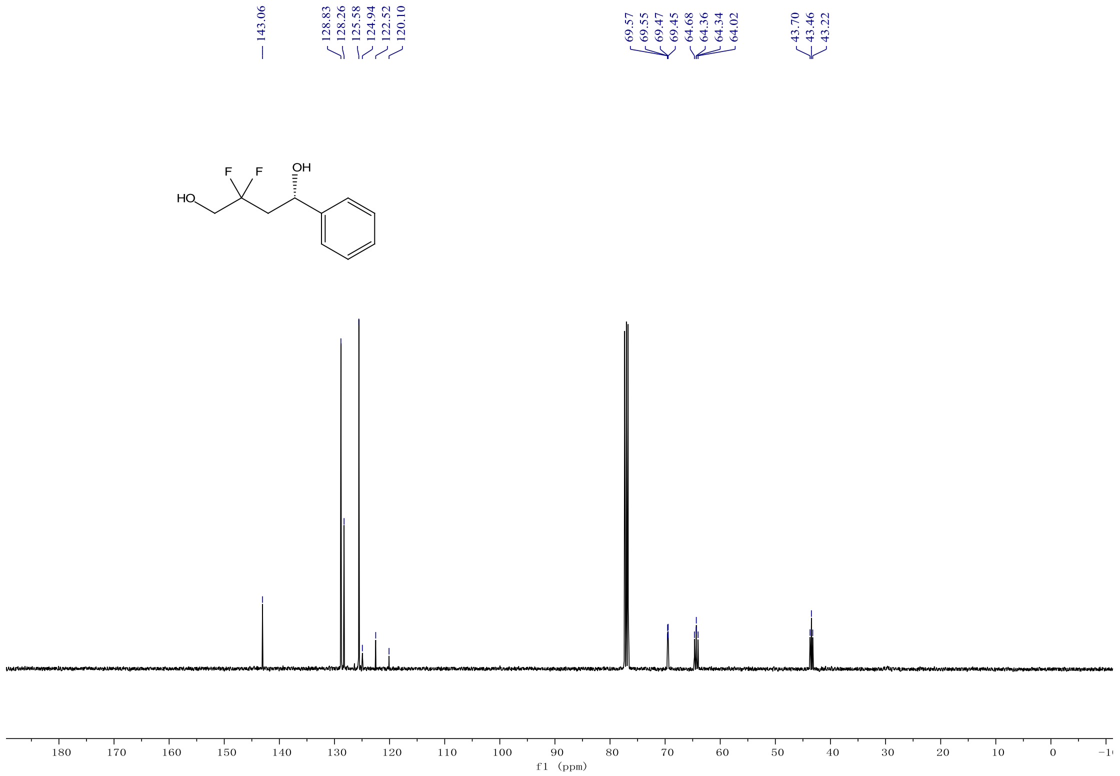

^{13}C{^{1}H}-NMR of compound **7** (101MHz, $CDCl_3$)

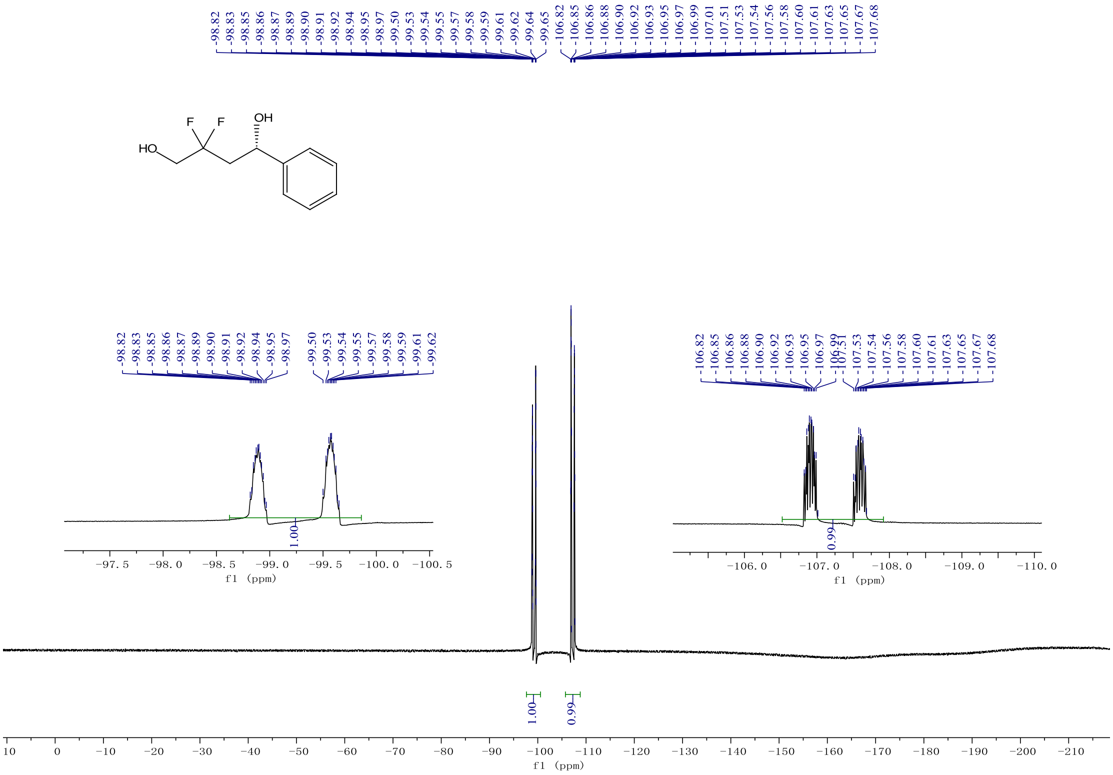

^{19}F-NMR of compound **7** (377MHz, $CDCl_3$)

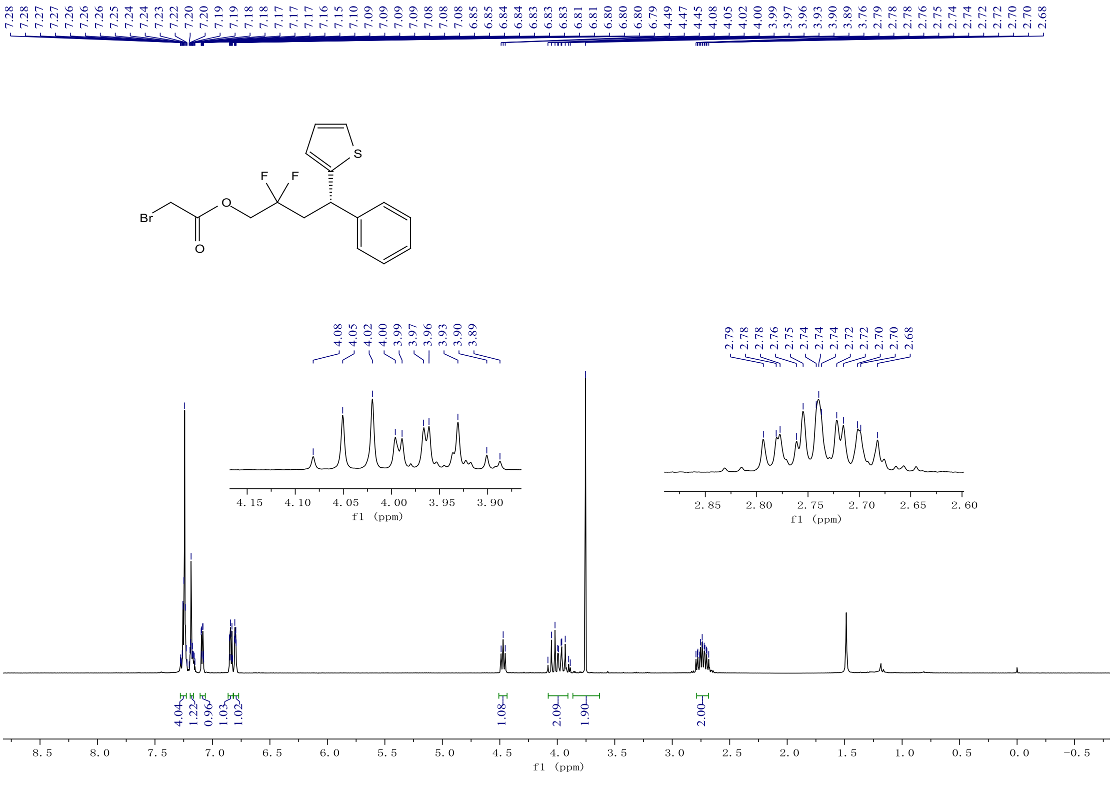

^{1}H-NMR of compound **8** (400MHz, $CDCl_3$)

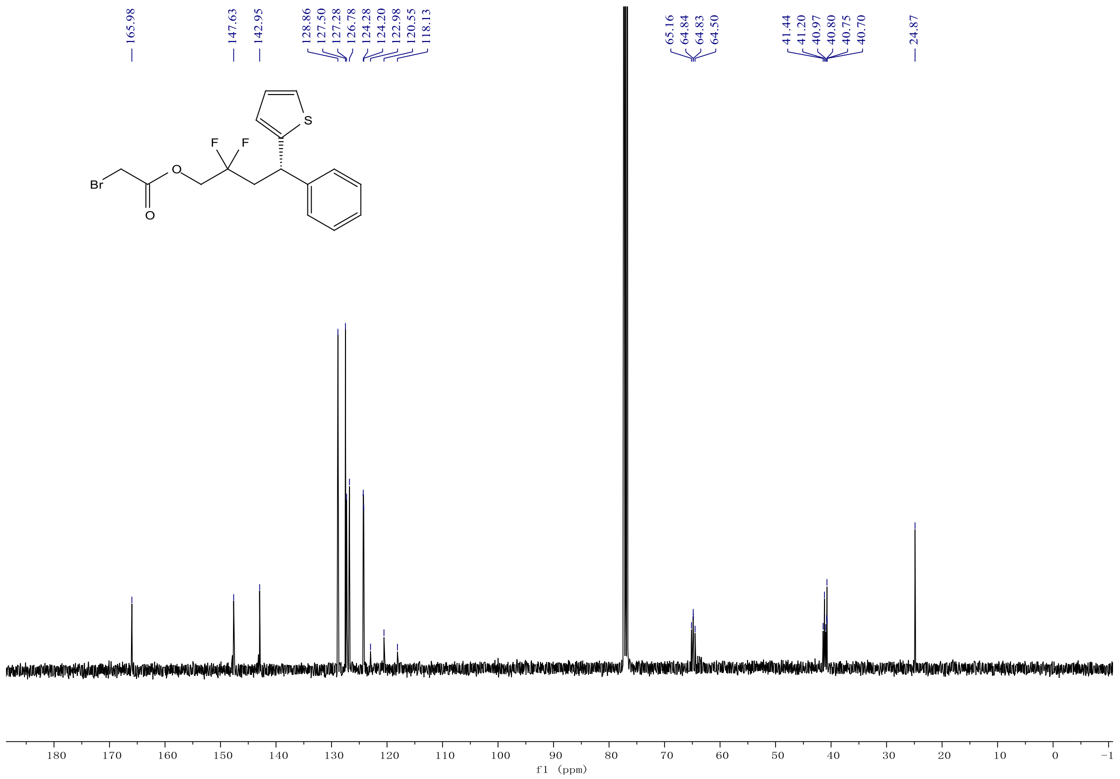

$^{13}C\{^{1}H\}$-NMR of compound **8** (101MHz, $CDCl_3$)

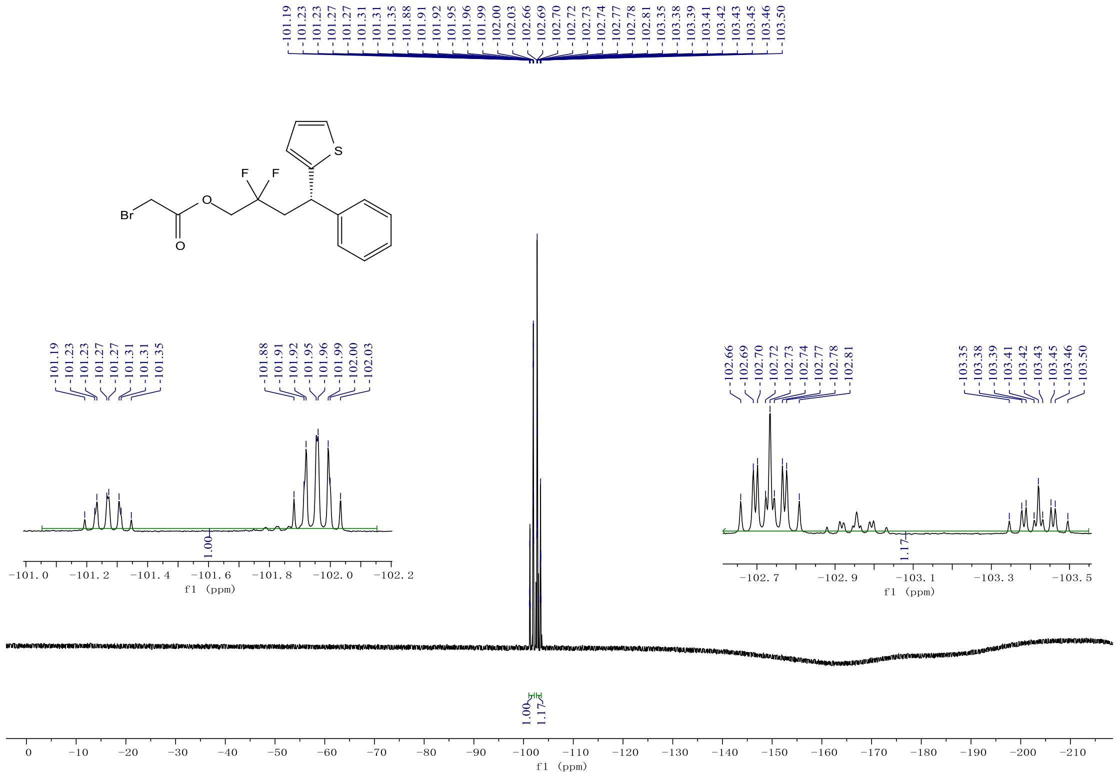

^{19}F-NMR of compound **8** (377MHz, $CDCl_3$)

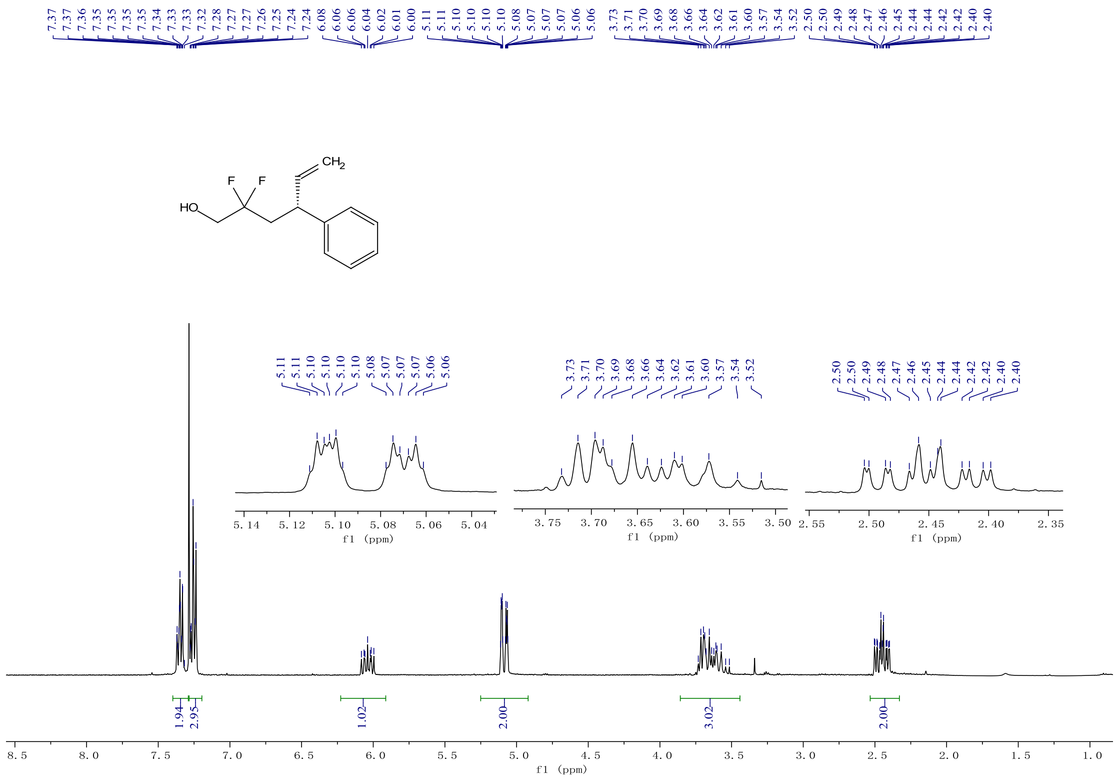

^{1}H-NMR of compound **9** (400MHz, $CDCl_3$)

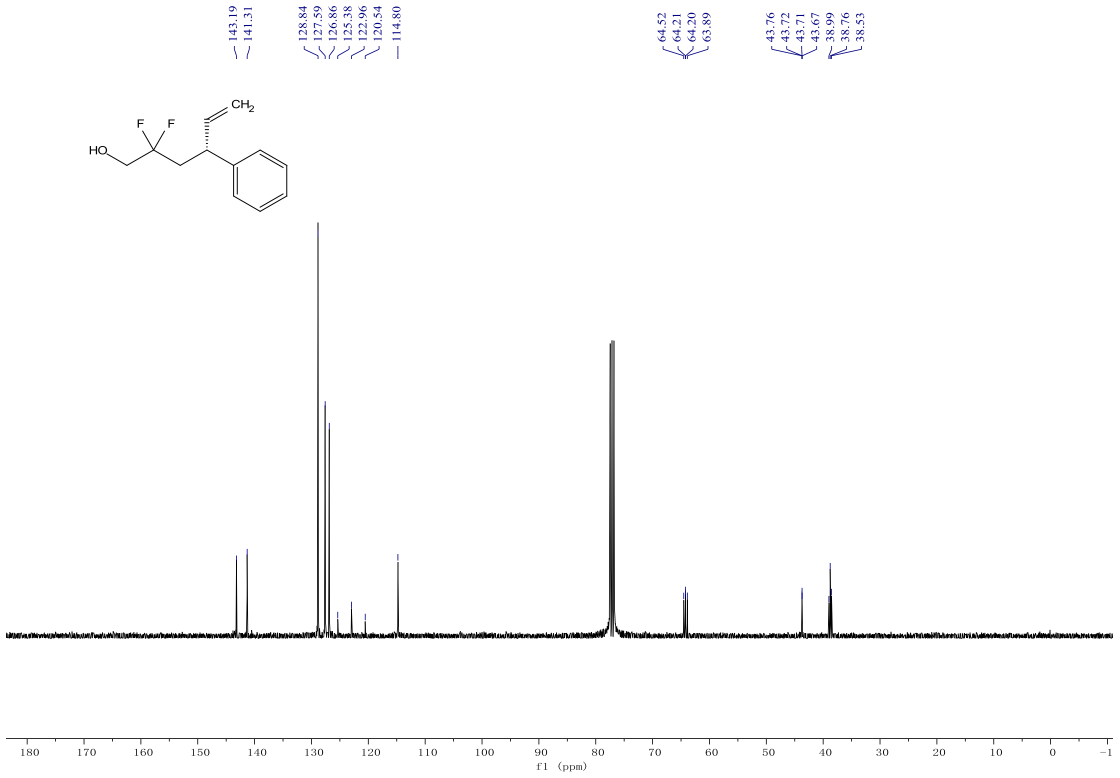

^{13}C{^{1}H}-NMR of compound **9** (101MHz, $CDCl_3$)

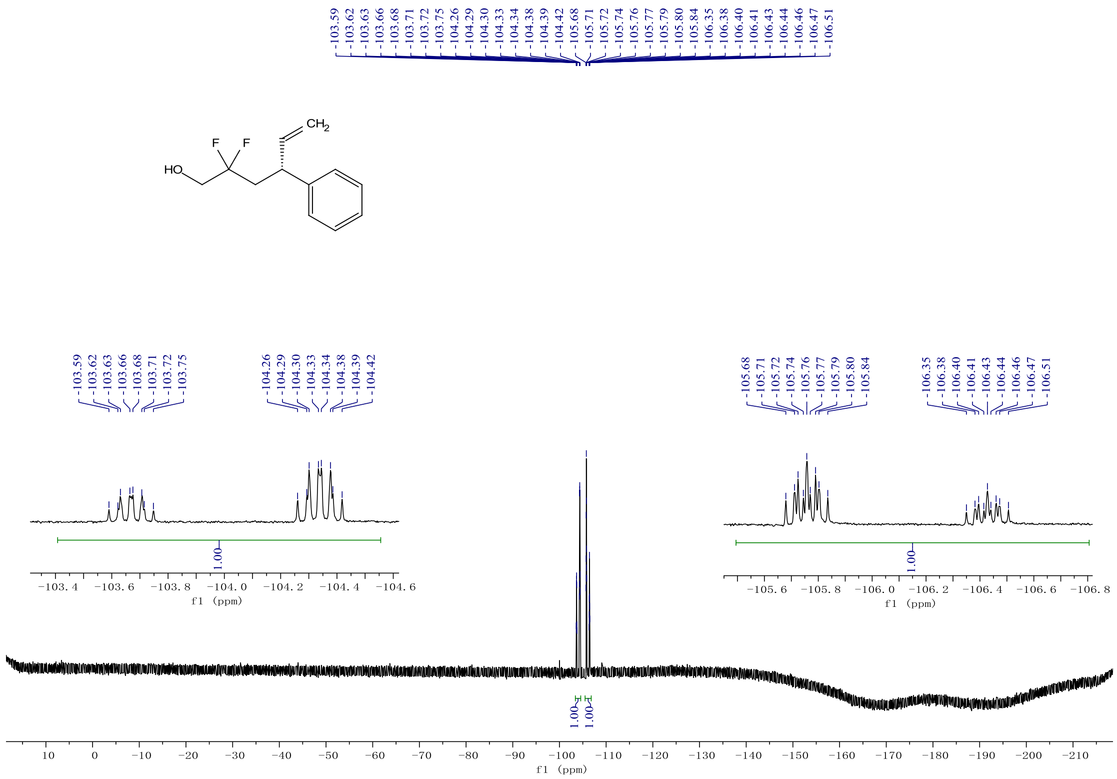

^{19}F-NMR of compound **9** (377MHz, $CDCl_3$)

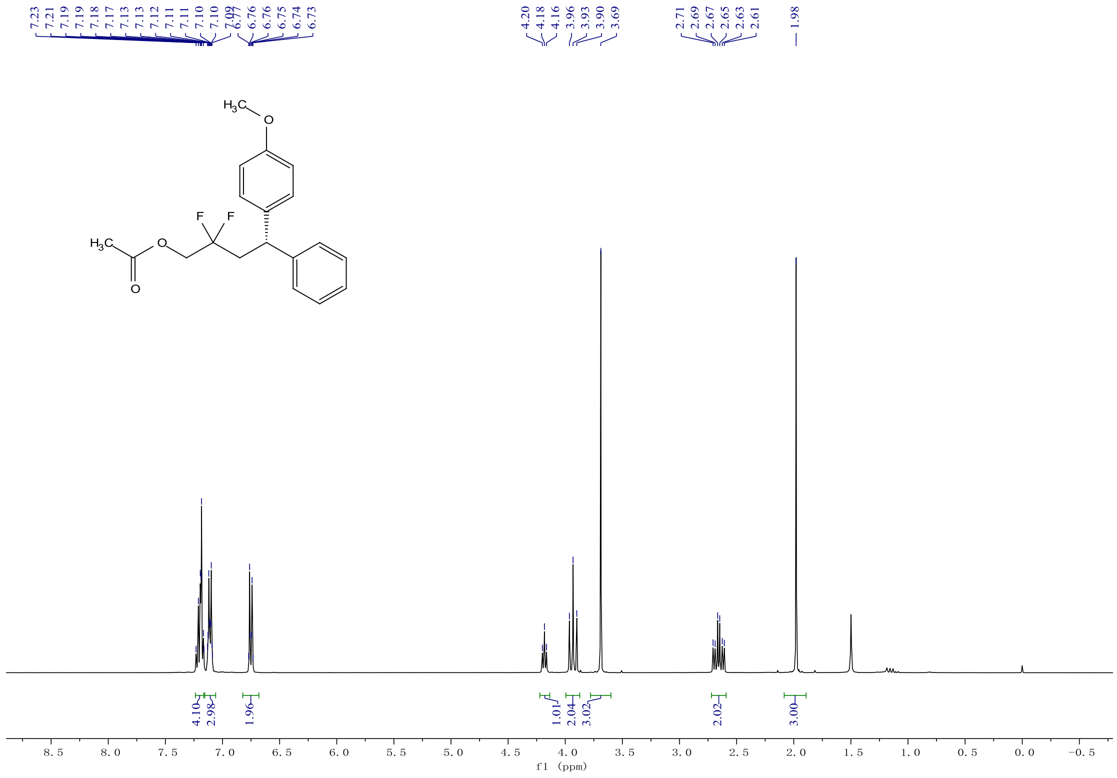

^{1}H-NMR of compound **10** (400MHz, $CDCl_3$)

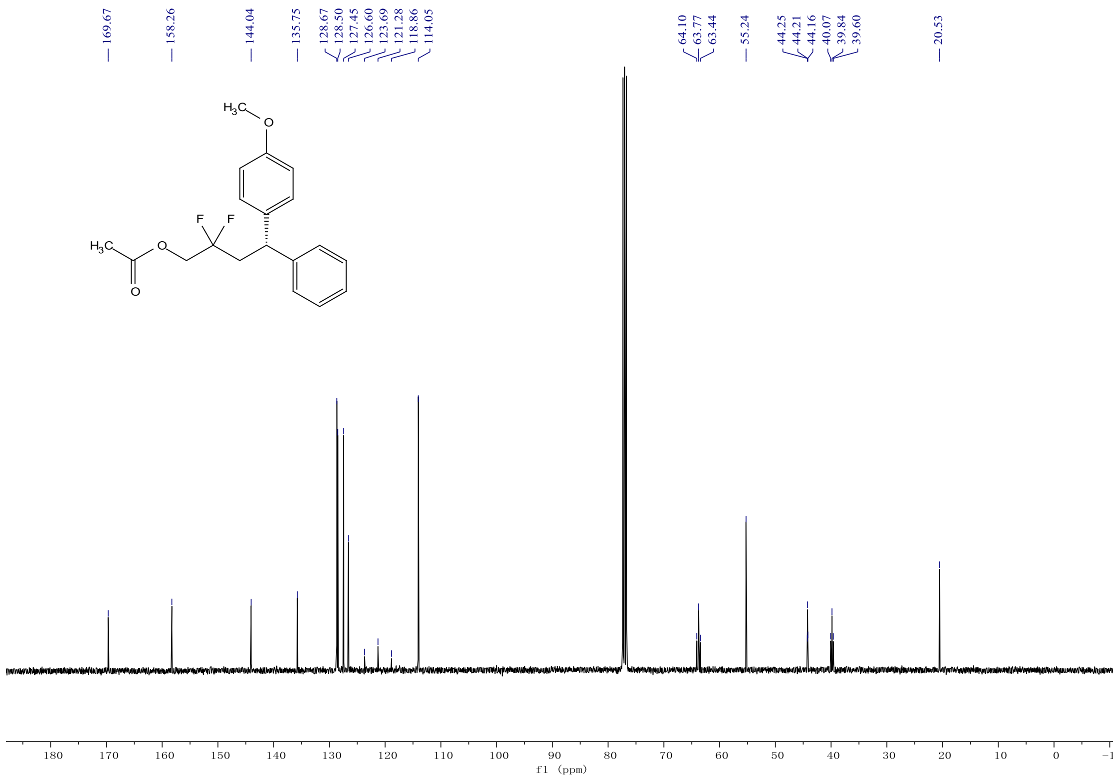

$^{13}C\{^{1}H\}$-NMR of compound **10** (101MHz, $CDCl_3$)

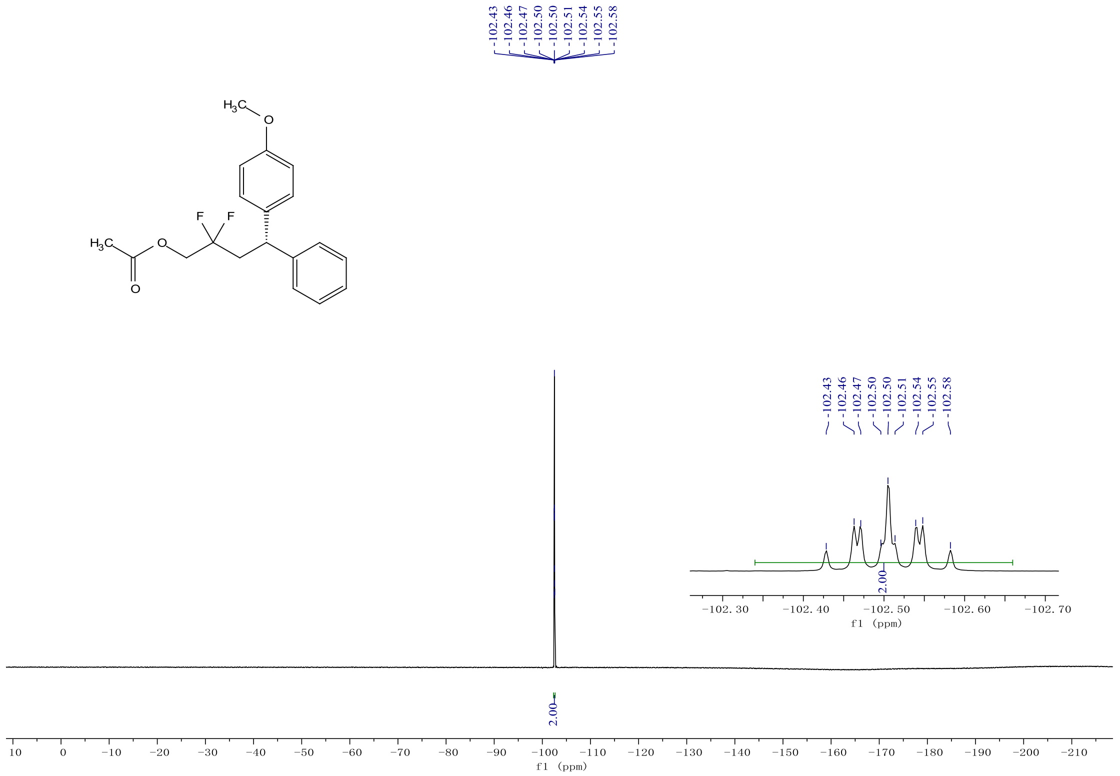

^{19}F-NMR of compound **10** (377MHz, $CDCl_3$)

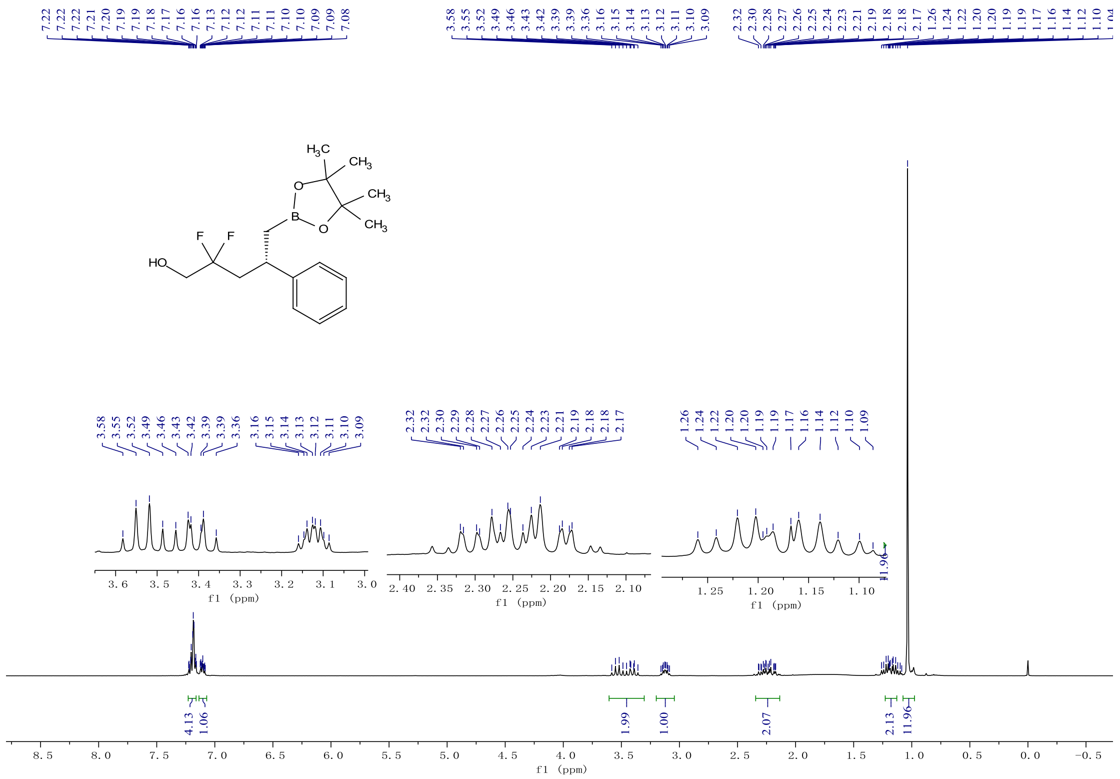

^{1}H-NMR of compound **11** (400MHz, $CDCl_3$)

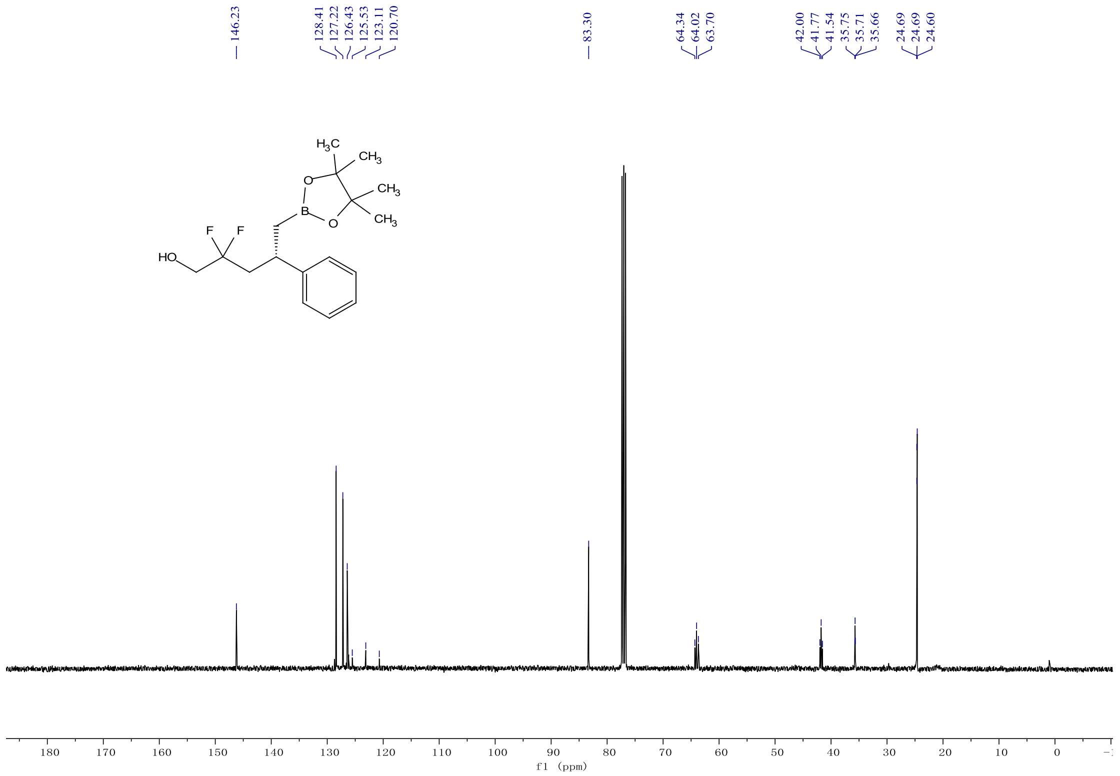

^{13}C{^{1}H}-NMR of compound **11** (101MHz, $CDCl_3$)

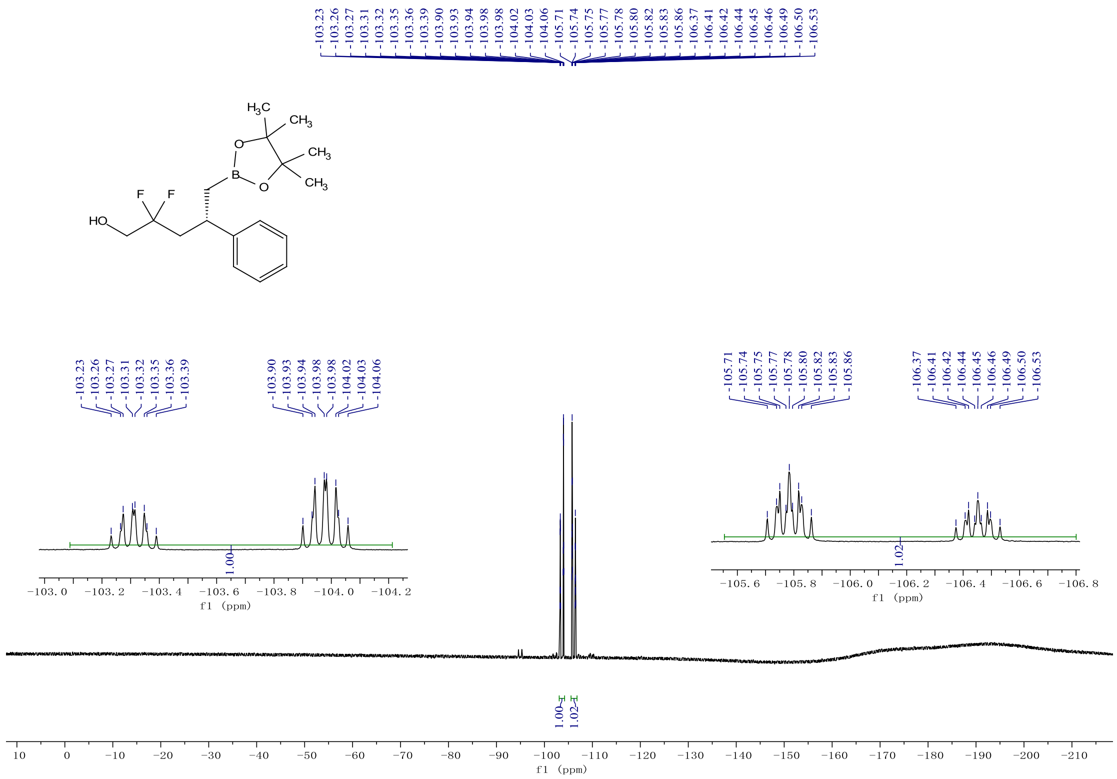

^{19}F-NMR of compound **11** (377MHz, $CDCl_3$)

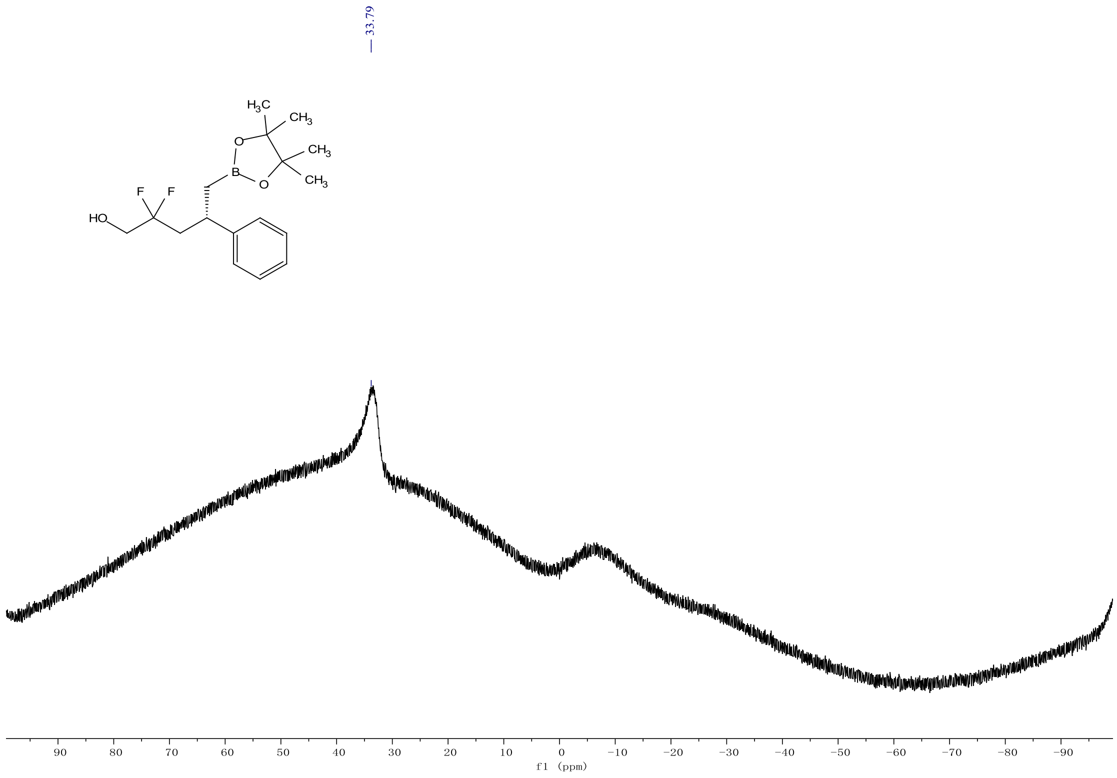

^{11}B-NMR of compound **11** (128MHz, $CDCl_3$)

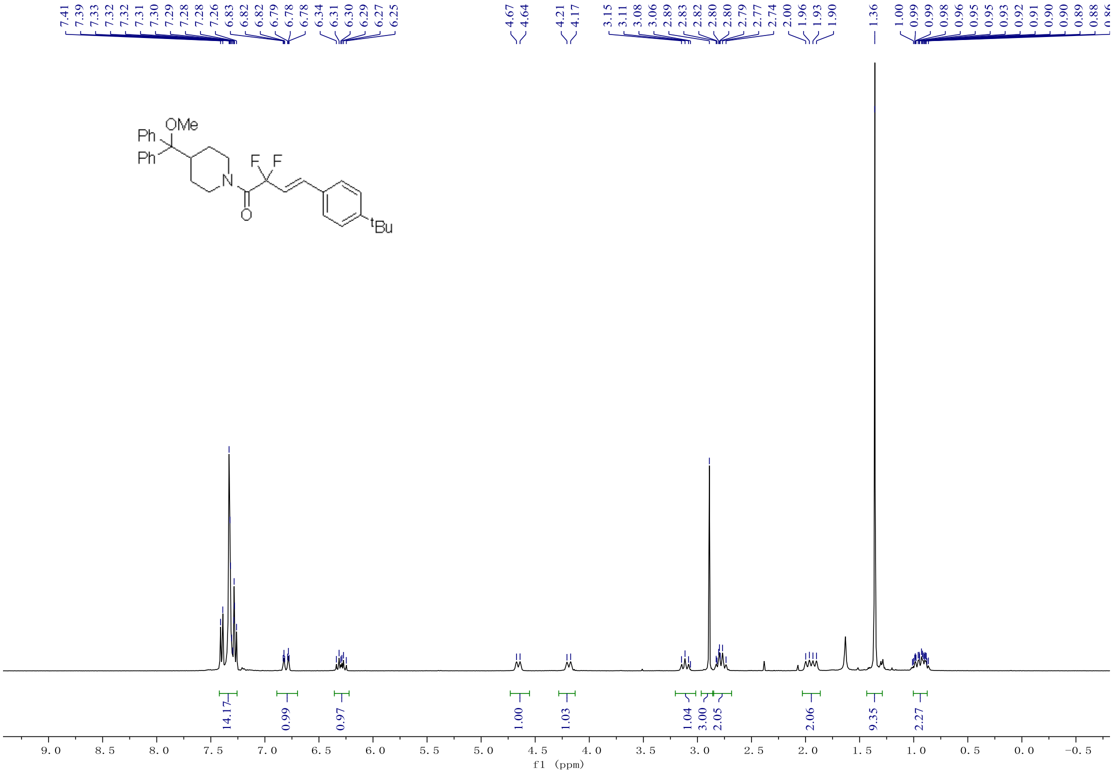

^{1}H-NMR of compound **12** (400MHz, $CDCl_3$)

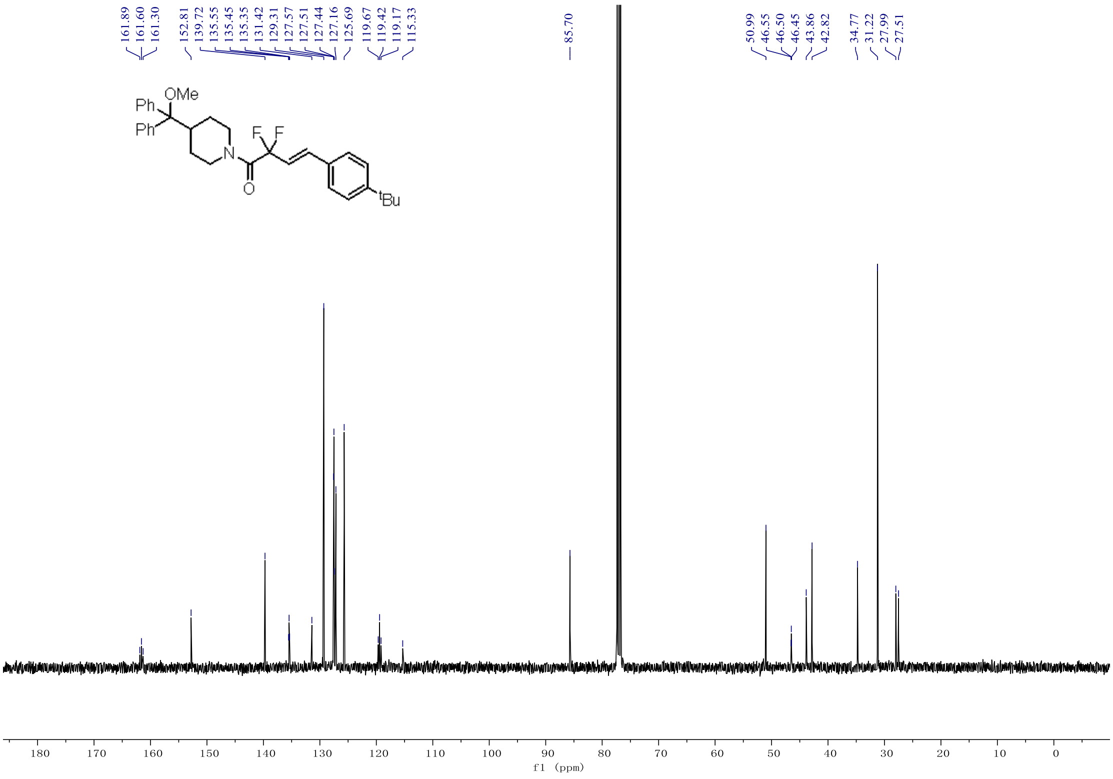

^{13}C{^{1}H}-NMR of compound **12** (400MHz, $CDCl_3$)

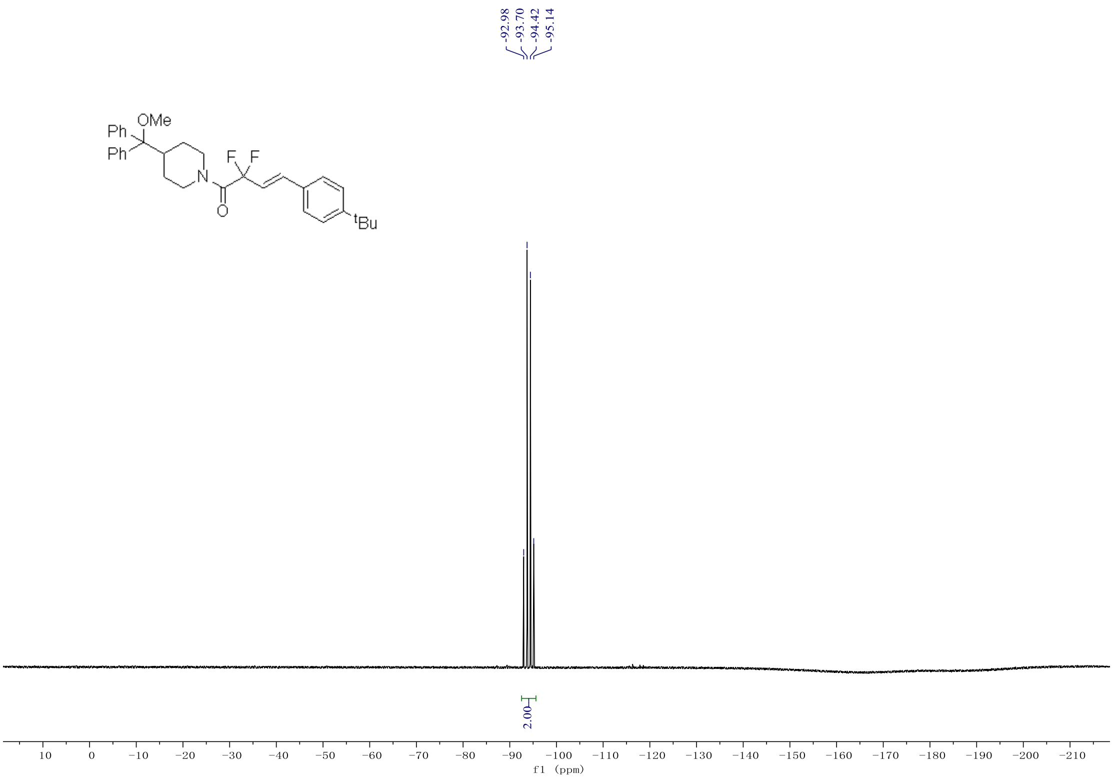

^{19}F{^{1}H}-NMR of compound **12** (377MHz, $CDCl_3$)

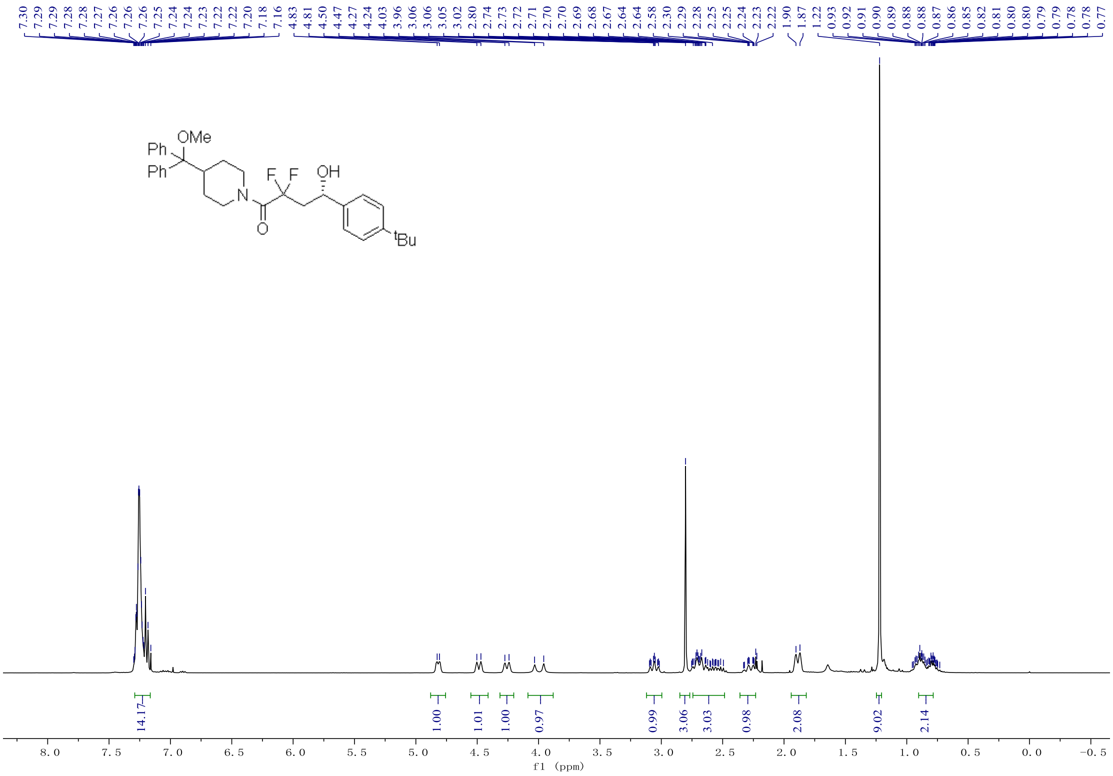

^{1}H-NMR of compound **13** (400MHz, $CDCl_3$)

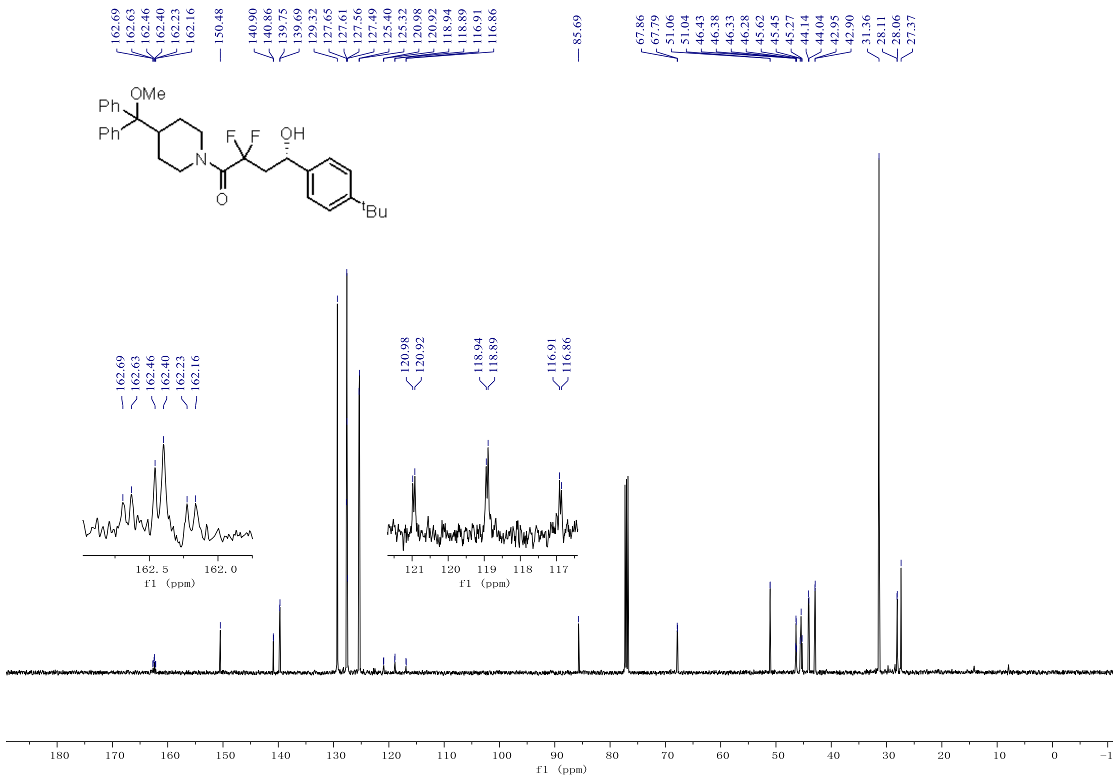

$^{13}C\{^{1}H\}$-NMR of compound **13** (126MHz, $CDCl_3$)

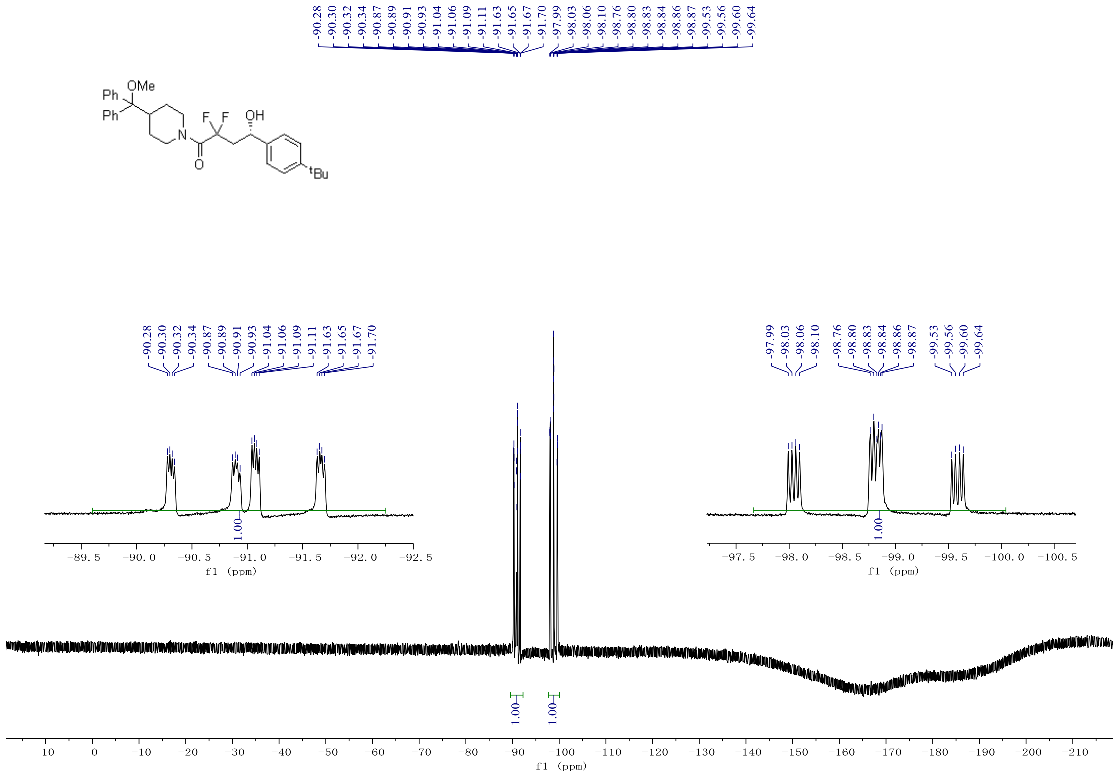

^{19}F-NMR of compound **13** (377MHz, $CDCl_3$)

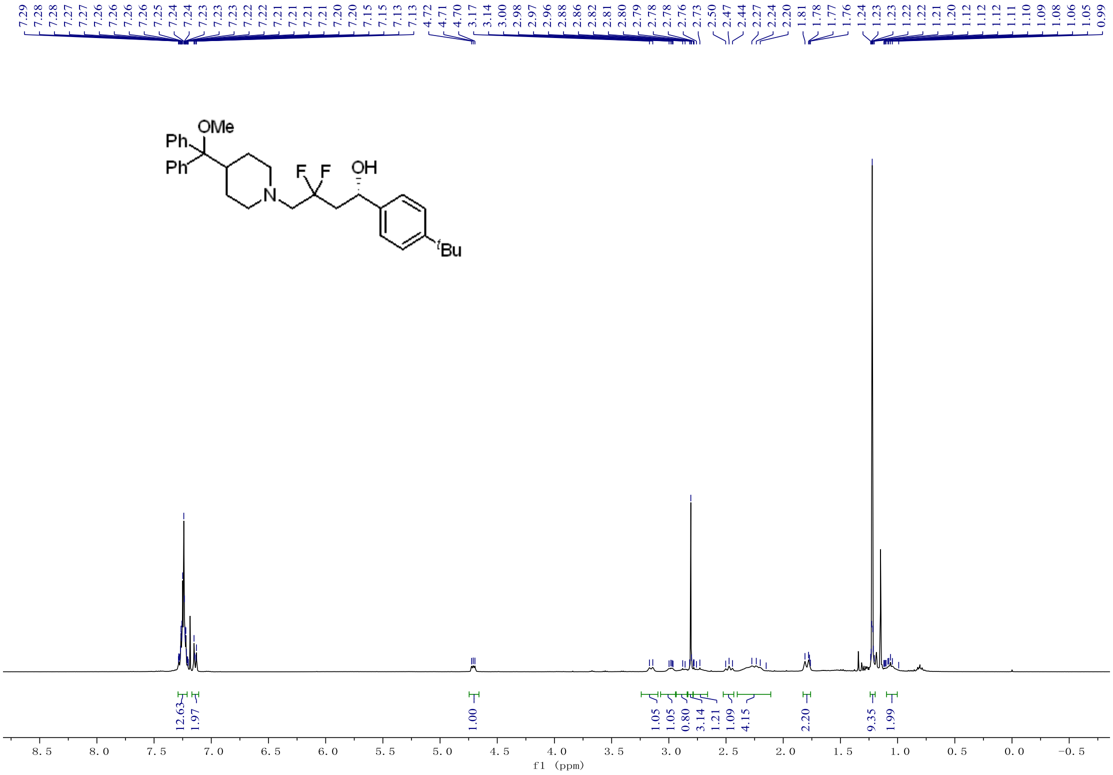

^{1}H-NMR of compound **14** (400MHz, $CDCl_3$)

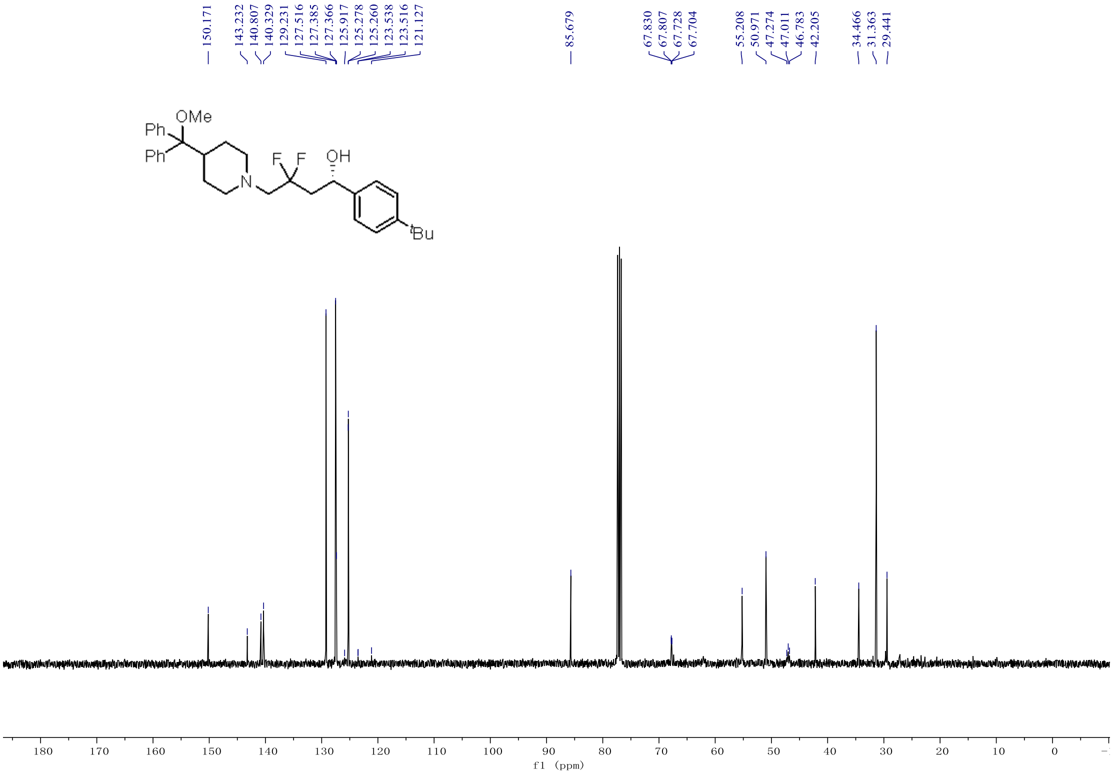

^{13}C{^{1}H}-NMR of compound **14** (101MHz, $CDCl_3$)

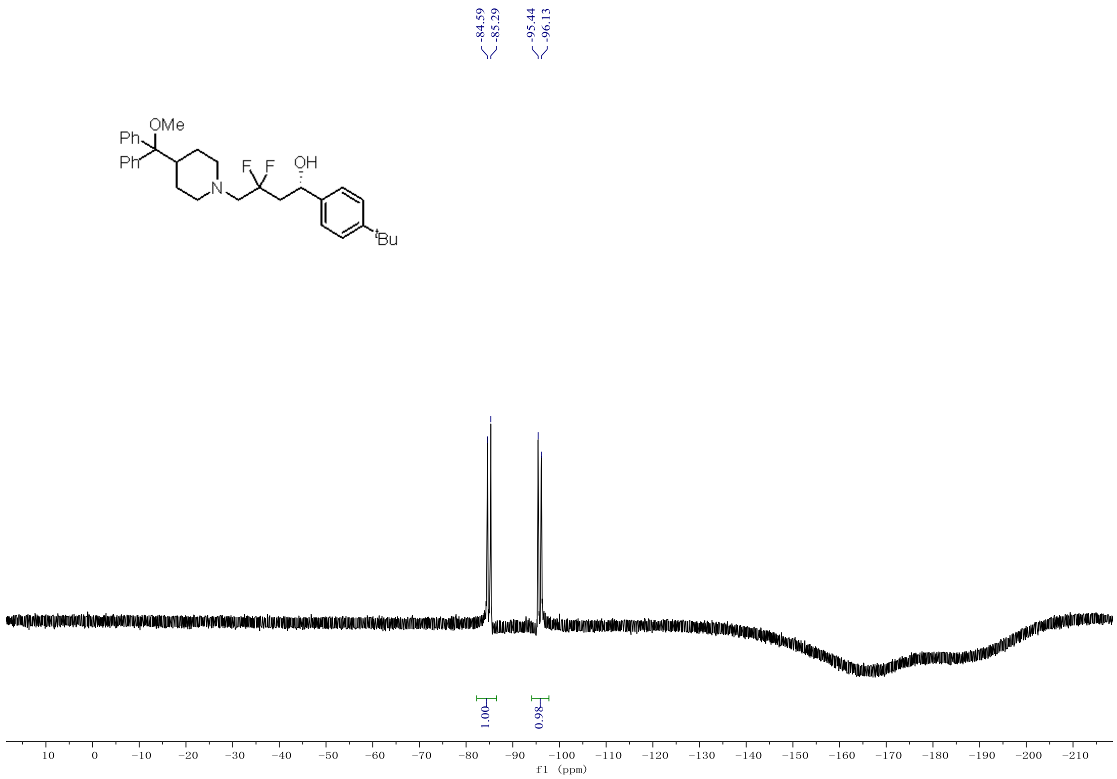

^{19}F-NMR{^{1}H} of compound **14** (377MHz, $CDCl_3$)

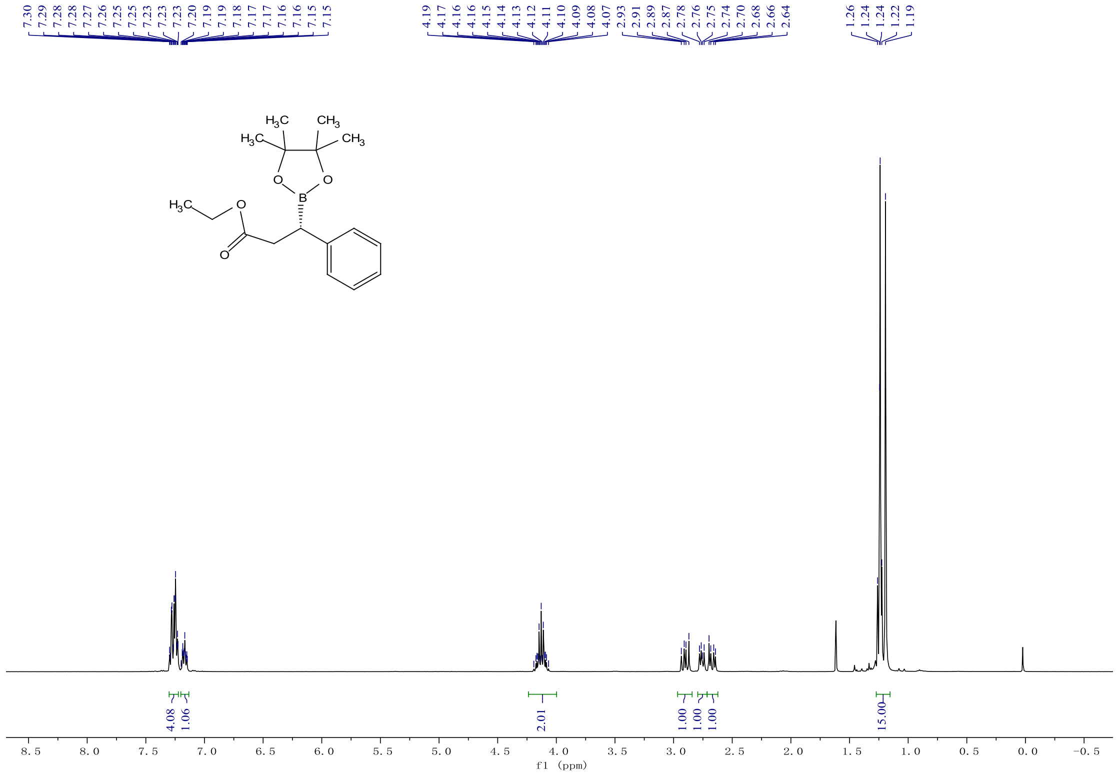

^{1}H-NMR of compound **17** (400MHz, $CDCl_3$)

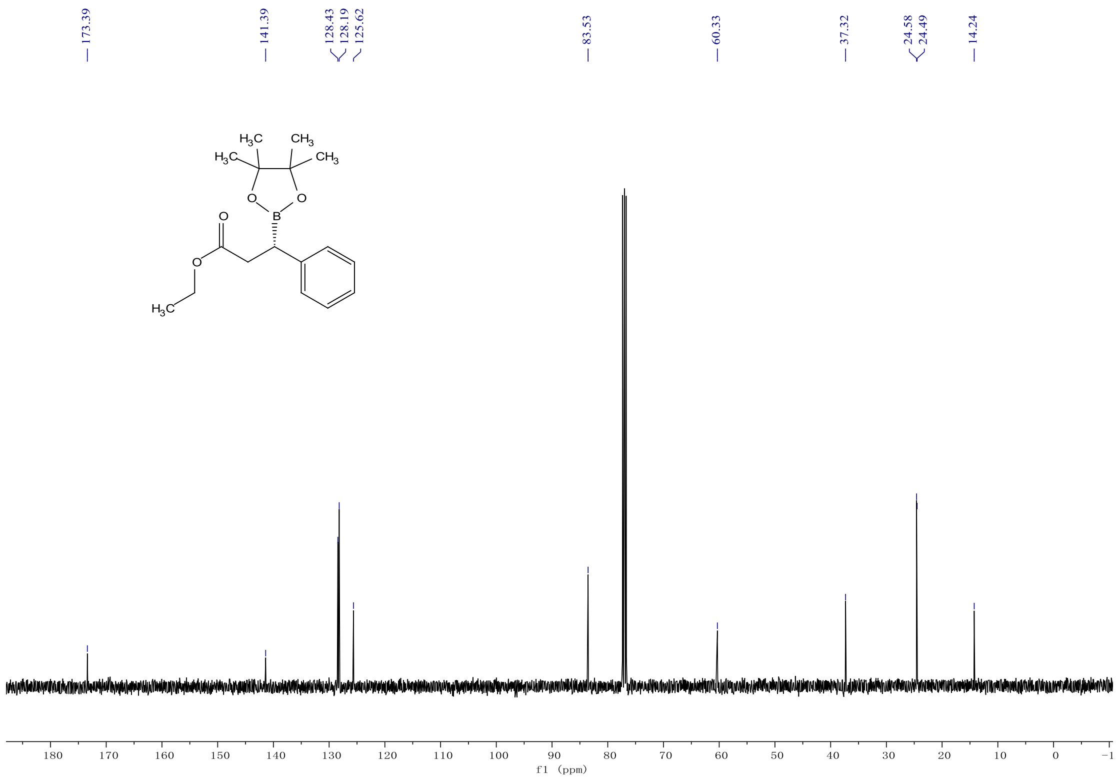

$^{13}C\{^{1}H\}$-NMR of compound **17** (101MHz, $CDCl_3$)

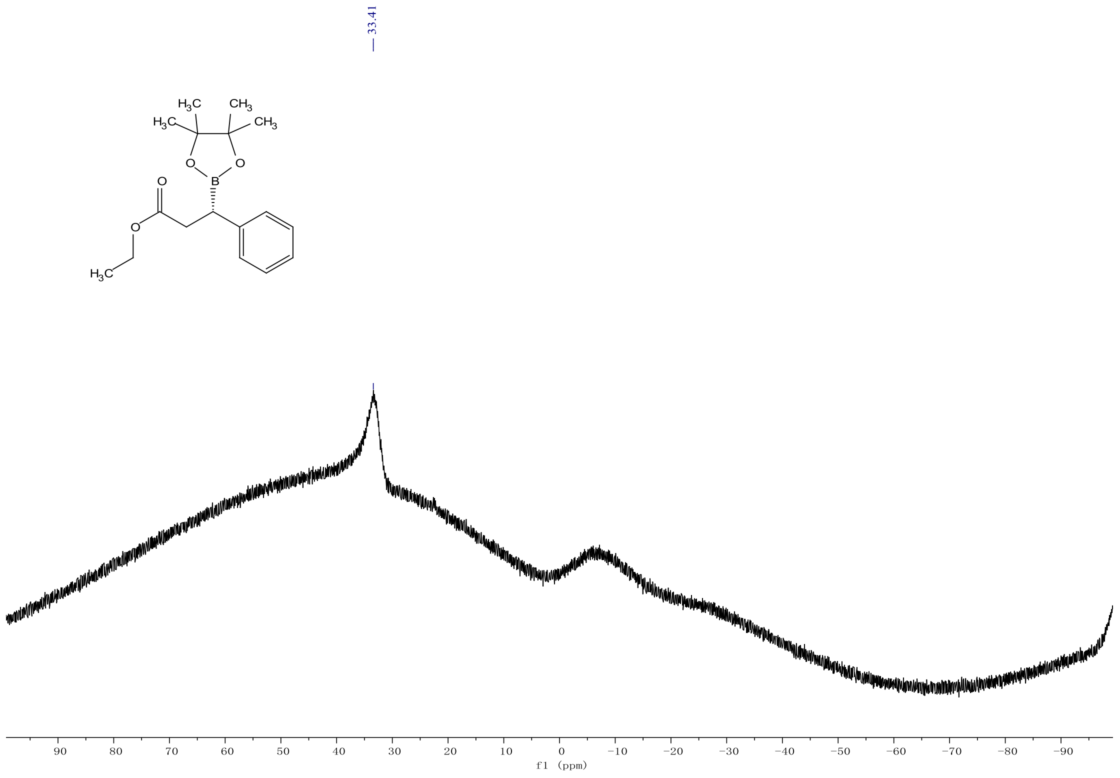

^{11}B-NMR of compound **17** (128MHz, $CDCl_3$)

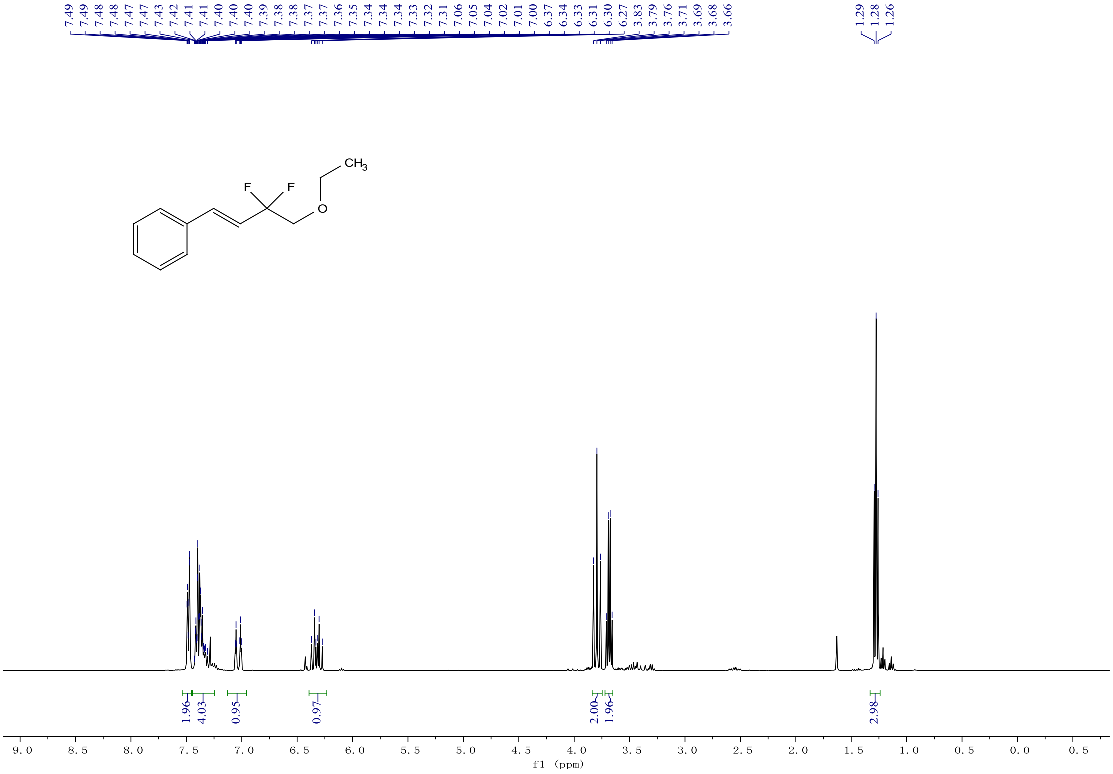

^{1}H-NMR of compound **19** (400MHz, $CDCl_3$)

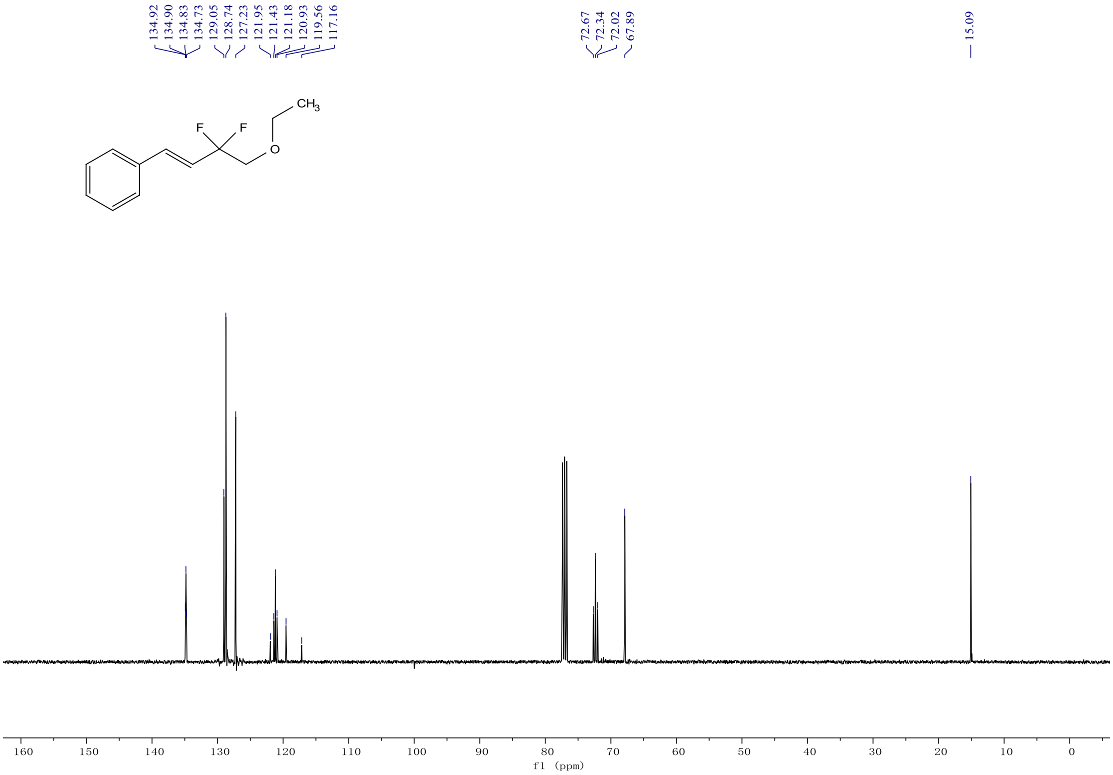

^{13}C{^{1}H}-NMR of compound **19** (101MHz, $CDCl_3$)

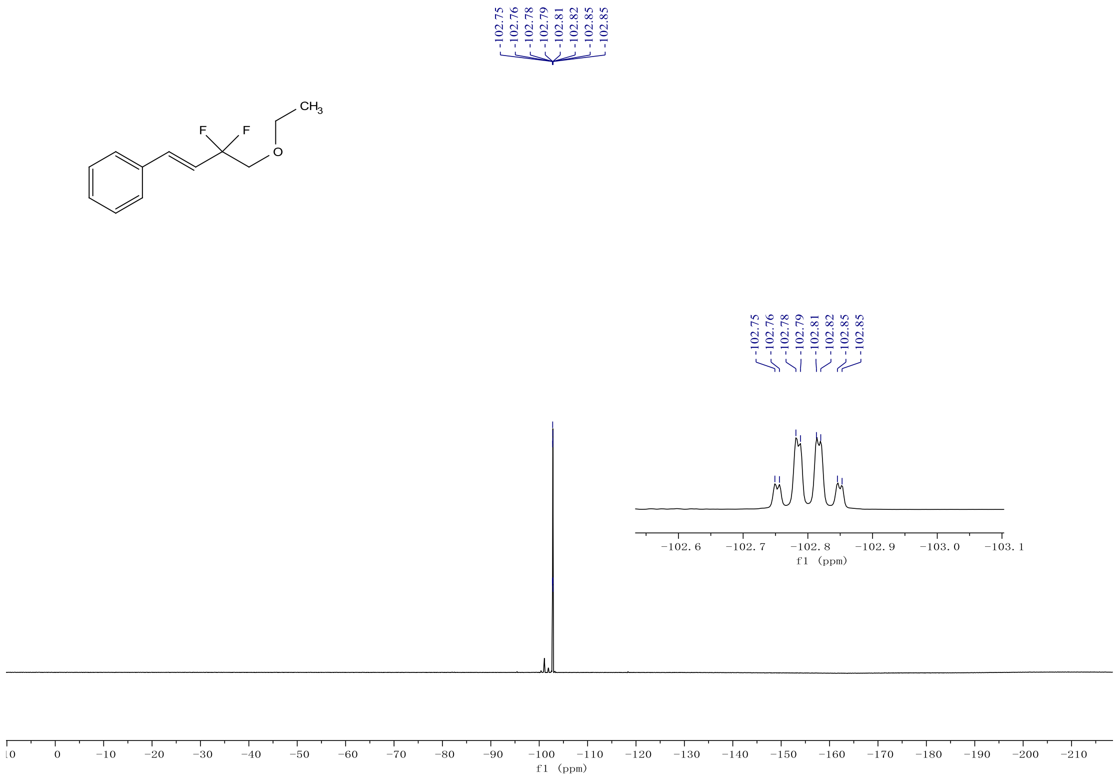

^{19}F-NMR of compound **19** (377MHz, $CDCl_3$)

8. X-Ray Crystallographic Data

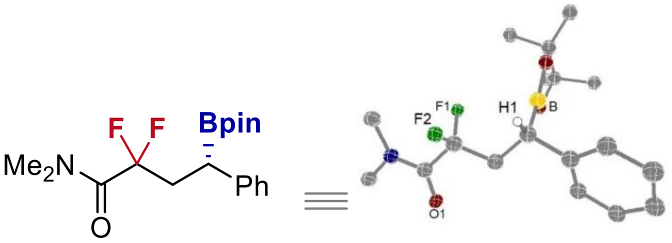

The X-ray crystallographic structures for **4b**. Crystal data have been deposited to CCDC, Deposition Number: 2262049.

Table 1 Crystal data and structure refinement for cu_1205_9_0m.

Identification code	cu_1205_9_0m
Empirical formula	$C_{18}H_{26}BF_2NO_3$
Formula weight	353.21
Temperature/K	150.00
Crystal system	triclinic
Space group	P1
a/Å	9.1428(8)
b/Å	10.6634(9)
c/Å	19.5896(16)
α/°	89.595(3)
β/°	86.148(3)
γ/°	83.389(3)
Volume/Å^3	1892.9(3)
Z	4
ρ_{calc}g/cm^3	1.239
μ/mm^{-1}	0.794
F(000)	752.0
Crystal size/mm^3	0.2 × 0.15 × 0.1
Radiation	CuKα (λ = 1.54178)
2Θ range for data collection/°	8.348 to 144.216
Index ranges	$-11 \le h \le 10$, $-13 \le k \le 13$, $-24 \le l \le 24$
Reflections collected	23146
Independent reflections	11953 [R_{int} = 0.0275, R_{sigma} = 0.0391]
Data/restraints/parameters	11953/3/913
Goodness-of-fit on F^2	1.056
Final R indexes [I>=2σ (I)]	R_1 = 0.0843, wR_2 = 0.2221
Final R indexes [all data]	R_1 = 0.0851, wR_2 = 0.2246
Largest diff. peak/hole / e Å^{-3}	0.75/-0.59
Flack parameter	0.05(4)

9. References

[1] X. Wang, S. Zhao, J. Liu, D. Zhu, M. Guo, X. Tang, G. Wang, *Org. Lett.* **2017**, *19*, 4187-4190.

[2] M.-T. Hsieh, K.-H. Lee, S.-C. Kuo, H.-C. Lin, *Adv. Syn. Catal.* **2018**, *360*, 1605-1610.

[3] C. H. Kim, R. Ramu, J. H. Ahn, M. A. Bae, Y. S. Cho, *Mol. Cell. Biochem.* **2010**, *344*, 91-98.

[4] Z. Wang, S. Bachman, A. S. Dudnik, G. C. Fu, *Angew. Chem. Int. Ed.* **2018**, *57*, 14529-14532.

[5] A. Kamal, M. Sandbhor, A. A. Shaik, *Tetrahedron: Asymmetry* **2003**, *14*, 1575-1580.

[6] M. Hu, B. B. Tan, S. Ge, *J. Am. Chem. Soc.* **2022**, *144*, 15333-15338.

[7] S. Chakrabarty, H. Palencia, M. D. Morton, R. O. Carr, J. M. Takacs, *Chem. Sci.* **2019**, *10*, 4854-4861.

[8] C. Crudden, B. Glasspoole, M. Oderinde, B. Moore, A. Antoft-Finch, *Synthesis* **2013**, *45*, 1759-1763.

[9] S. H. Cho, J. F. Hartwig, *J. Am. Chem. Soc.* **2013**, *135*, 8157-8160.

[10] J. Hao, W. Ding, Z. Zheng, L. Sun, J. Dong, M. Li, W. Wan, *J. Org. Chem.* **2022**, *87*, 13828-13836.

[11] H. Y. Zhao, Z. Feng, Z. Luo, X. Zhang, *Angew. Chem. Int. Ed.* **2016**, *55*, 10401-10405.

[12] M. Frisch, G. Trucks, H. Schlegel, G. Scuseria, M. Robb, J. Cheeseman, G. Scalmani, V. Barone, B. Mennucci, G. Petersson, *See also: URL: http://www. gaussian. com* 2009.

[13] Y. Zhao, D. G. Truhlar, *Acc. Chem. Res.* **2008**, *41*, 157-167.

[14] F. Weigend, R. Ahlrichs, *Phys. Chem. Chem. Phys.* **2005**, *7*, 3297-3305.

[15] A. V. Marenich, C. J. Cramer, D. G. Truhlar, *J. Phys. Chem. B* **2009**, *113*, 6378-6396.

[16] T. Lu, F. Chen, *J. Comput. Chem.* **2012**, *33*, 580-592.

[17] T. Lu, Q. Chen, *J. Comput. Chem.* **2022**, *43*, 539-555.

[18] W. L. DeLano, *http://www. pymol. org/* 2002.
